# Incidence and survival of interstitial lung diseases in the UK in 2010–2019

**DOI:** 10.1183/23120541.00823-2024

**Published:** 2025-03-03

**Authors:** Francesca Gonnelli, Neva Eleangovan, Ursie Smith, Heath Heatley, Vidya Navarantam, Tamera J. Corte, David B. Price, Victoria Carter, Martina Bonifazi, Caitlin C. Fermoyle, Richard Hubbard

**Affiliations:** 1 Translational Medical Sciences, University of Nottingham, Nottingham, UK; 2 Respiratory Unit, Department of Biomedical Sciences and Public Health, Polytechnic University of Marche, Ancona, Italy; 3 Observational and Pragmatic Research Institute Pte Ltd, Singapore; 4 Optimum Patient Care Global, Cambridge, UK; 5 Department of Respiratory Medicine, Sir Charles Gardiner Hospital, Perth, Australia; 6 School of Medicine, University of Queensland, Brisbane, Australia; 7 Centre for Respiratory Research, University of Western Australia, Perth, Australia; 8 Department of Respiratory and Sleep Medicine, Royal Prince Alfred Hospital, Sydney, Australia; 9 Centre of Academic Primary Care, Division of Applied Health Sciences, University of Aberdeen, Aberdeen, UK; 10 NHMRC Centre of Research Excellence in Pulmonary Fibrosis, Sydney, Australia; 11 Faculty of Medicine and Health, The University of Sydney, Sydney, Australia; 12 Interstitial Lung Disease, Pleural Disease and Adult Bronchiectasis Unit, Department of Internal Medicine, “AOU delle Marche”, Ancona, Italy

## Abstract

**Background:**

With the introduction of the antifibrotic drugs targeting progressive pulmonary fibroses, it becomes imperative to provide reliable contemporary estimates of the most common interstitial lung diseases. We aimed to provide contemporary estimates of the incidence and survival of idiopathic pulmonary fibrosis (IPF), hypersensitivity pneumonitis (HP) and connective tissue disease-associated interstitial lung disease (CTD-ILDs), and to compare their survival to that of the general population. To do this we have used data extracted from the Optimum Patient Care Research Database (OPCRD).

**Methods:**

In this matched cohort study, we extracted incident cases of HP, CTD-ILD and IPF, and age and sex matched controls for each case, for the years 2010–2019. We calculated annual incidence rates and analysed incidence trends over time using segmented regression modelling. We estimated survival for cases and controls using the Kaplan–Meier model.

**Results:**

We extracted data for 18 914 incident cases of interstitial lung diseases between 2010 and 2019 from the OPRCD. Incidence rates varied across the different diseases, with rates of 18.12, 7.96 and 2.63 per 100 000 person-years for IPF, CTD-ILD and HP, respectively. 5-year survival for IPF, CTD-ILD and HP was 40%, 54% and 66%, respectively, and this was generally ∼50% lower than that of the general population.

**Conclusion:**

Our population-based study emphasises the considerable burden of interstitial lung diseases, with >20 000 new cases diagnosed each year in the UK, many of whom will be eligible for antifibrotic drugs.

## Shareable abstract

**An overall incidence of 28.71 per 100 000 person-years with 5-year survivals ranging from 40% to 66%, half that the general population, highlights the burden of ILDs, with >20 000 new cases each year in the UK** https://bit.ly/4h7yP26

## Introduction

Previous research using primary care data sources has shown that the burden from interstitial lung disease (ILDs) is increasing in the UK and in Europe [1–5]. A more recent study by Gupta *et al*. [6] explored incidence, prevalence and mortality for idiopathic pulmonary fibrosis (IPF) in the UK, showing a recent stabilisation of these burden estimates. However, with the introduction of new drugs to treat progressive pulmonary fibrosis other than IPF, to adequately plan healthcare services, it is now timely to consider the incidence of common types of ILDs including IPF, hypersensitivity pneumonitis (HP) and connective tissue disease-associated ILDs (CTD-ILDs). In fact, providing updated estimates of their incidence and survival would provide an accurate starting point to set up ILD services.

Therefore, the primary aim of our study was to provide contemporary estimates of incidence and survival for the most common fibrosing ILDs which might be eligible for antifibrotic drugs – IPF, HP and CTD-ILDs. In addition, we compared their survival to that of age and sex matched general population controls. To do this, we have used electronic medical records from the Optimum Patient Care Research Database (OPCRD) [7, 8]. As secondary aims, we analysed incidence trends over time and explored possible burden disparities across sexes and age groups and compared survival data across the three different ILDs.

## Methods

### Data source

We extracted data from 1 January 2010 to 31 December 2019. We used this time period to give us 10 years of data to analyse, but to exclude the period of the COVID-19 pandemic. We utilised OPCRD as source of data, as it is a longitudinal healthcare database that currently comprises anonymised electronic health records for 25 million patients, from over 1000 primary care practices across the UK.

### Participants and study design

In this observational prospective national cohort study, we estimated the incidence of IPF, HP and CTD-ILDs. We also performed a matched cohort study to evaluate the survival rate of patients with these diseases compared to the general population.

We defined incident cases using our previously established method [1] as all patients with a first record of ILD (index date), and at least 1 year of disease-free registration in the current practice. We defined our HP cases as any incident case with at least one Read/SNOMED code [9] indicative of HP. We defined our CTD-ILD cases as any incident case aged >18 years with at least one Read/SNOMED code [9] indicative of CTD-ILDs (or lung fibrosis and a code for a CTD separately), with no other code suggestive for HP, following the definition proposed by Thomas *et al*. [10]. We included in the CTD group the following diseases: 1) rheumatoid arthritis; 2) systemic sclerosis; 3) inflammatory myopathies; and 4) others including rheumatic polymyalgia and Sjogren syndrome.

We defined as IPF any incident case with at least one Read/SNOMED code [9] indicative of IPF or an IPF-related prescription, with no codes for other ILDs. To define IPF we built on the system devised by Morgan *et al*. [11], which uses a narrow and broad definition. To ensure the inclusion of as many cases as possible, we used the same narrow disease band as Morgan but included additional codes for prescriptions for antifibrotic drugs (these were generally only used for IPF during the study time period). To describe IPF, we use the term IPF-clinical syndrome (IPF-CS) throughout the text [1, 2].

In case of multiple codes, we used the following order: HP → CTD-ILDs → IPF narrow definition → IPF broad definition.

We excluded from the analysis all patients aged <18 years at diagnosis, and those with missing demographics or index date. We also excluded people with sarcoidosis and/or pneumoconiosis.

The code lists are reported in the supplementary material 2 (E1–E2).

We drew controls from all individuals in OPRCD who did not have a diagnosis of ILD. We individually matched up four controls to each case based on age (±3 years), sex and general practice. Controls needed to be alive, contributing data to OPCRD at the index date of their matched case, and at least 1 year of registration before the index date of their matched case. Each control was allocated an index date corresponding to the ILD diagnosis date of their matched case.

In our cohort study, patients registered in primary care practices contributing research quality data to OPCRD were followed up from the latest of 1 January 2010 or 1 year after the current registration date in the primary care practice to the earliest of date of IPF diagnosis recorded in primary care, 31 December 2019, death and date of transfer out of the practice. For our denominators we used the total OPCRD annual mid-year populations stratified by age and sex (supplementary material E1).

In our matched cohort study, we followed up patients with IPF and matched controls from the index date to the earliest of 31 December 2019, death and date of transfer out of the practice.

### Outcomes

For the cohort study the primary outcome was a new ILD diagnosis. For the survival analysis study, the primary outcome was the all-cause death detected both in cases and controls and defined as a death record identified in the OPCRD.

### Covariates

We extracted demographic features, disease-related variables and covariates (supplementary material 1, E2). We ascertained the status of each covariate on or before the index date. In case of multiple records, we considered the latest available.

### Statistical analysis

We calculated the overall crude incidence rate for each ILD by dividing the number of incident cases by the population denominator. To ensure the comparability with previous studies [1], we considered the whole population denominator including those aged 0–17 years in our analysis. We stratified incidence rates by calendar year, sex and age group, and used Poisson regression modelling to estimate incidence rate ratios between different sex and age categories. We used multiplicative interaction terms to test for modification by age and sex.

We used the segmented regression model to analyse each ILD specific incidence rate trends over time and to calculate average annual per cent change and trends over time.

We estimated the hazard ratios (HRs) for death for each ILD group using Cox proportional hazards regressions. We used age, sex, body mass index (BMI), smoking history, comorbidities and steroids exposure as covariates.

We carried out life table analyses to estimate cumulative mortality at 5 years after incident diagnosis for each ILD category. We compared the risk of mortality between each ILD category and IPF-CS subgroup using the multivariable fully adjusted Cox regression model.

For the survival analysis, we used Kaplan–Meier and Cox regression models, both overall and stratified by sex and age. We used propensity score weighting to compare survival in cases and controls. More details regarding methodology are reported in supplementary material 1 (E2).

## Results

### Population

**FIGURE 1:**
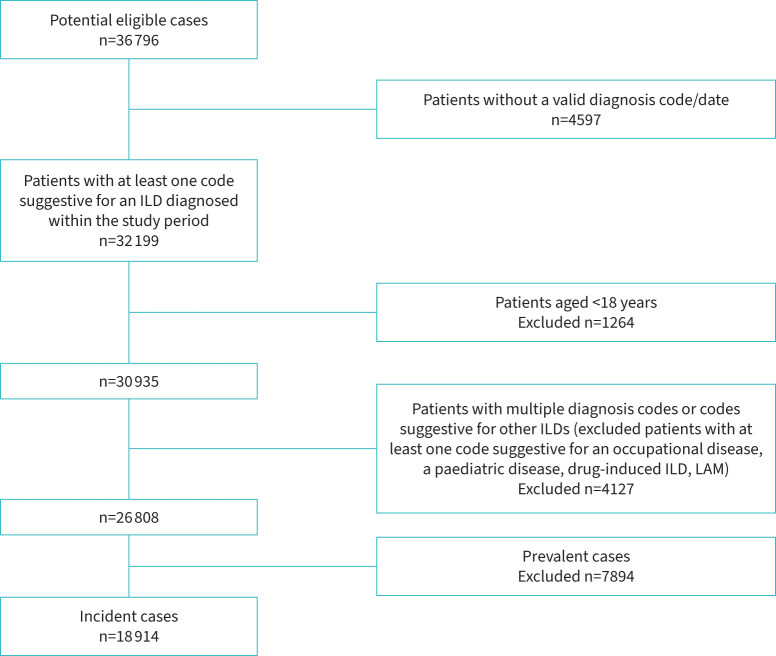
Incident cases selection of ILDs: decisional flow-chart followed to get the definitive cases cohort. ILD: interstitial lung disease; LAM: lymphangioleiomyomatosis.

After removing people with missing data (missing diagnosis date or code, missing demographics), people aged <1  years, and prevalence cases (figure 1), we identified a total of 18 914 incident cases of ILDs: 915 (4.84%) patients with an IPF-CS narrow codeset, 11 002 (58.27%) with an IPF-CS broad codeset, 5244 (27.73%) with CTD-ILD and 1733 (9.16%) with HP (table 1).

**TABLE 1:** Baseline clinical and demographic data of incident patients with ILDs included in the study

	Hypersensitivity pneumonitis	CTD-ILDs	IPF-CS narrow definition	IPF-CS broad definition
**Patients n**	1733	5244	915	11 022
**Age at diagnosis years, mean±sd**	61.9±15.0	71.1±11.8	75.7±9.6	74.9±11.1
**Sex, female, n (%)**	925 (53.4)	2789 (53.2)	294 (32.1)	3967 (36.0)
**Smoking status, n (%)**				
Nonsmoker	826 (47.7)	2163 (41.3)	351 (38.7)	3915 (35.5)
Current smoker	202 (11.7)	571 (11.0)	62 (6.8)	1136 (10.3)
Former smoker	610 (35.2)	2361 (45.1)	452 (49.6)	5605 (51.0)
Passive smoker	4 (0.2)	3 (0.1)	2 (0.2)	9 (0.1)
Missing data	89 (5.1)	141 (2.7)	43 (4.7)	354 (3.2)
**BMI category, n (%)**				
Underweight	81 (4.6)	183 (3.5)	15 (1.6)	390 (3.5)
Normal weight	441 (25.7)	1504 (28.7)	251 (28.2)	3007 (27.3)
Overweight	561 (32.8)	1941 (37.1)	377 (41.2)	4376 (39.8)
Obese	638 (36.8)	818 (30.7)	265 (29.0)	3241 (29.4)
**Cerebrovascular disease, n (%)**	139 (8.0)	544 (10.4)	123 (13.5)	1400 (12.7)
**Chronic kidney disease, n (%)**	203 (11.7)	1160 (22.1)	182 (20.0)	2575 (23.4)
**COPD, n (%)**	161 (9.3)	728 (13.9)	102 (11.2)	1833 (16.7)
**Ischaemic heart disease, n (%)**	230 (13.3)	1221 (23.3)	265 (29.1)	3140 (28.5)
**Dementia, n (%)**	22 (1.3)	108 (2.1)	21 (2.3)	352 (3.2)
**Diabetes mellitus, n (%)**	290 (16.8)	955 (18.2)	203 (22.3)	2447 (22.2)
**Liver disease, n (%)**	64 (3.7)	298 (5.7)	32 (3.5)	360 (3.3)
**Current/previous malignancy, n (%)**	284 (16.4)	701 (13.4)	135 (14.8)	1758 (16.0)
**Peptic ulcer, n (%)**	102 (5.9)	443 (8.5)	77 (8.4)	914 (8.3)
**Peripheral artery disease, n (%)**	121 (7.0)	924 (17.6)	128 (14.0)	1623 (14.8)
**Psychiatric disorders, n (%)**	29 (1.7)	41 (0.8)	5 (0.5)	117 (1.1)
**Steroid exposure, n (%)**	501 (28.9)	2187 (41.7)	144 (15.8)	2179 (19.8)

ILDs: interstitial lung diseases; CTD: connective tissue disease; IPF-CS: idiopathic pulmonary fibrosis clinical syndrome; BMI: body mass index.

The overall mean±sd age at time of diagnosis was 72.7±12.2 years and 7975 (42.2%) were female. People with HP tended to be younger at diagnosis (mean age 61.9±15.0 years) compared to the other disease groups. People with IPF-CS were predominantly male, whilst people with CTD-ILDs and HP were more likely to be female (53.2% and 53.4%, respectively) (table 1).

The mean and median registration time at the GP practice before the diagnosis of ILD were 23.34±17.78 years and 19.83 years (IQR 10.40–30.95), respectively.

We extracted a total of 60 156 matched controls (98% of cases having four controls).

The demographics of patients and controls are displayed in table 1 and supplementary material 1, E3.

### Incidence analysis

We found the crude incidence rate of 18.12 per 100 000 person-years (95% CI 17.80–18.45) for IPF-CS, 7.96 per 100 000 person-years (95% CI 7.75–8.18) for CTD-ILDs and 2.63 per 100 000 person-years (95% CI 2.51–2.76) for HP, giving an overall crude incidence rate for all ILDs of 28.71 per 100 000 person-years (95% CI 28.30–29.12) (table 2).

**TABLE 2:** Overall and annual raw incidence rates along with 95% confidence intervals for CTD-ILDs, HP, IPF-CS and each IPF-CS subgroup, and overall ILDs

	CTD-ILDs				HP				IPF-CS				IPF-CS narrow definition				IPF-CS broad definition				All ILDs			
Year	Cases	Incidence rate (per 100 000 person-years)	95% CI		Cases	Incidence rate (per 100 000 person-years)	95% CI		Cases	Incidence rate (per 100 000 person-years)	95% CI		Cases	Incidence rate (per 100 000 person-years)	95% CI		Cases	Incidence rate (per 100 000 person-years)	95% CI		Cases	Incidence rate (per 100 000 person-years)	95% CI	
			LCI	UCI			LCI	UCI			LCI	UCI			LCI	UCI			LCI	UCI			LCI	UCI
**2010**	469	8.73	7.96	9.55	152	2.83	2.40	3.32	950	17.68	16.57	18.84	63	1.17	0.90	1.50	887	16.51	15.44	17.63	1571	29.23	27.81	30.72
**2011**	468	8.24	7.51	9.02	182	3.20	2.76	3.71	977	17.20	16.14	18.32	79	1.39	1.10	1.73	898	15.81	14.79	16.88	1627	28.65	27.27	30.07
**2012**	491	8.32	7.60	9.08	158	2.68	2.27	3.13	1137	19.26	18.15	20.41	119	2.02	1.67	2.41	1018	17.24	16.20	18.33	1786	30.25	28.86	31.68
**2013**	459	7.45	6.78	8.16	148	2.40	2.03	2.82	1112	18.04	16.99	19.13	81	1.31	1.04	1.63	1031	16.73	15.72	17.78	1719	27.89	26.58	29.24
**2014**	492	7.60	6.94	8.30	156	2.41	2.05	2.82	1083	16.72	15.74	17.75	89	1.37	1.10	1.69	994	15.35	14.41	16.33	1731	26.73	25.48	28.02
**2015**	492	7.35	6.71	8.03	140	2.09	1.76	2.47	1213	18.12	17.11	19.17	95	1.42	1.15	1.73	1118	16.70	15.73	17.71	1845	27.56	26.31	28.84
**2016**	530	7.72	7.07	8.40	170	2.48	2.12	2.88	1236	18.00	17.01	19.03	105	1.53	1.25	1.85	1131	16.47	15.52	17.46	1936	28.19	26.95	29.47
**2017**	568	7.96	7.32	8.64	179	2.51	2.15	2.90	1280	17.93	16.96	18.94	74	1.04	0.81	1.30	1206	16.90	15.96	17.88	2027	28.40	27.17	29.66
**2018**	607	8.02	7.40	8.69	232	3.07	2.68	3.49	1465	19.37	18.39	20.38	95	1.26	1.02	1.54	1370	18.11	17.16	19.10	2304	30.46	29.23	31.73
**2019**	668	8.34	7.72	8.99	216	2.70	2.35	3.08	1484	18.52	17.59	19.48	115	1.43	1.18	1.72	1369	17.08	16.19	18.01	2368	29.55	28.37	30.76
**Overall**	5244	7.96	7.75	8.18	1733	2.63	2.51	2.76	11 937	18.12	17.80	18.45	915	1.39	1.30	1.48	11 022	16.73	16.42	17.05	18 914	28.71	28.30	29.12

CTD: connective tissue disease; ILD: interstitial lung disease; HP: hypersensitivity pneumonitis; IPF-CS: idiopathic pulmonary fibrosis clinical syndrome; LCI: lower confidence interval; UCI: upper confidence interval.

We estimated a crude incidence rate of 1.39 per 100 000 person-years (95% CI 1.30–1.48) in “IPF-CS narrow”, and 16.73 per 100 000 person-years (95% CI 16.42–17.05) in “IPF-CS broad” (table 2).

Incidence rates were higher in men compared with women, and rose along with increasing age in both overall IPF-CS and the broad and narrow IPF-CS subgroups (supplementary material 1, E4). The increasing incidence with age was also present in CTD-ILDs and in HP, although male predominance was not present in both these categories.

We found no multiplicative interactions between incidence and age, and sex in all the ILD categories and in each IPF-CS subgroup.

**FIGURE 2:**
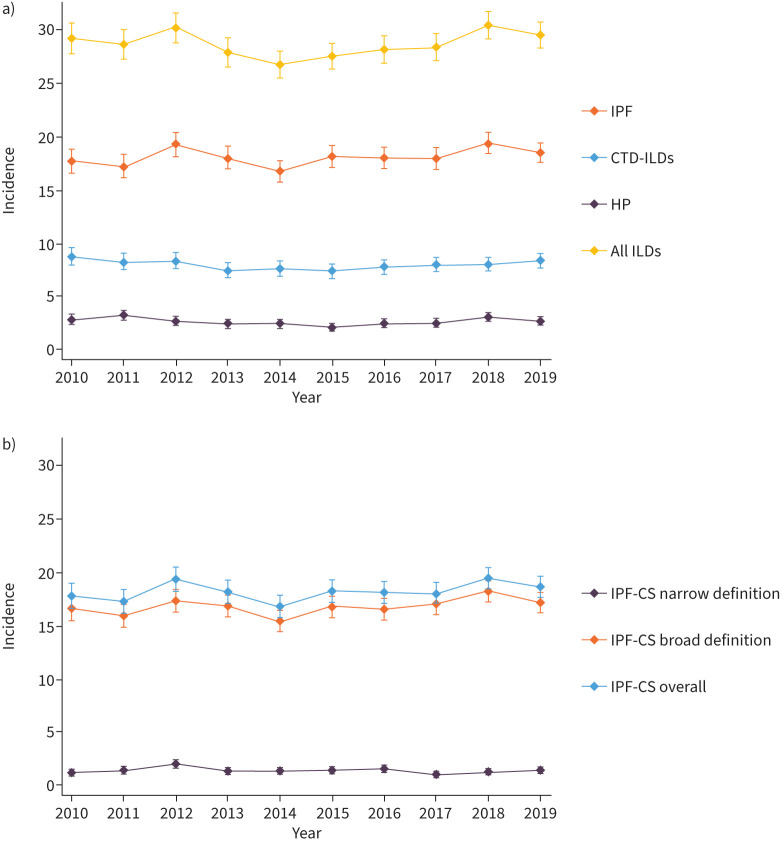
a) Incidence rates (per 100 000 person-years) over time comparing all the ILDs, overall IPF-CS, CTD-ILDs and HP; b) incidence rates (per 100 000 person-years) over time comparing overall IPF-CS definition and each IPF-CS codeset band. ILD: interstitial lung disease; IPF-CS: idiopathic pulmonary fibrosis clinical syndrome; CTD-ILD: connective tissue disease-associated interstitial lung disease; HP: hypersensitivity pneumonitis.

We found that the overall incidence rate trend for ILDs was stable between 2010 and 2019 (figure 2a). Overall IPF-CS incidence rates were also stable over time (figure 2a). Within the IPF-CS categories, the narrow code band incidence rate showed a mild non-significant decreasing trend from 2010 to 2019, whereas the broader code band maintained a flat trend (figure 2b). The incidence rate for CTD-ILDs slightly decreased until 2015, showing an increasing trend thereafter, with an overall stable trend over the study period (figure 2b). Similarly, HP incidence rate remained stable over time, slightly decreasing until 2015, then started rising (figure 2b). More detail regarding the results of the segmented regression analysis is reported in the supplementary material 1, E5.

### Survival analysis

#### ILD incident cases

The mean follow-up time in our ILD cohort after diagnosis was 3.03 person-years. The mean follow-up time was 2.77±2.44 years, 2.76±2.44 years, 2.80±2.35 years, 3.42±2.72 years and 2.69±2.88 years in overall IPF-CS, broad IPF-CS definition, narrow IPF-CS definition, CTD-ILDs and HP, respectively.

During that period, we found a total of 601 and 6949 deaths in narrow and broad IPF-CS case sets, respectively. We also detected 3029 and 791 deaths from CTD-ILDs, and HP, respectively.

We estimated the 5-year survival percentage of our cohort to be 39% in IPF-CS, 54% in CTD-ILDs and 66% in HP (table 3). Kaplan–Meier estimates are displayed in supplementary material E6.

**TABLE 3:** 5-year survival of each ILD category and IPF-CS subgroup and their matched controls

ILD category	Cases		Controls	
	Number of events/at risk	5-year survival %	Number of events/at risk	5-year survival %
**Hypersensitivity pneumonitis**	103/554	66	205/1728	89
**CTD-ILDs**	457/1411	54	841/4629	79
**IPF-CS overall**	760/2210	39	1982/9010	71
**IPF-CS narrow**	66/162	32	160/737	73
**IPF-CS broad**	694/2048	39	1822/8273	71

ILD: interstitial lung disease; IPF-CS: idiopathic pulmonary fibrosis clinical syndrome; CTD: connective tissue disease.

Within our cohort, increasing age and male sex was associated with poor prognosis in all the ILD groups (table 4, supplementary material 1, E7).

**TABLE 4:** Number of deaths and relative person-time stratified by age and sex; age- and sex- adjusted HRs for each ILD category and each IPF-CS subgroup

	Row data		Survival			
	Deaths, n	Person-time, years	HR	95% CI		p-value
ILD category				LCI	UCI	
**CTD-ILDs**						
Sex						
Female	1118	3.49	Reference			
Male	1199	3.33	1.22	1.12	1.32	<0.0001
Age at onset years						
0–54	119	4.32	Reference			
55–59	89	4.31	1.16	0.88	1.53	0.20
60–64	186	4.11	1.57	1.25	1.97	<0.0001
65–69	310	3.89	1.99	1.61	2.46	<0.0001
70–74	401	3.41	2.43	1.98	2.98	<0.0001
75–79	500	3.10	3.17	2.59	3.87	<0.0001
80–84	401	22.66	4.03	3.28	4.95	<0.0001
≥85	311	2.13	5.93	4.79	7.34	<0.0001
**Hypersensitivity pneumonitis**						
Sex						
Female	296	3.69	Reference			
Male	553	3.68	1.03	0.87	1.21	0.77
Age at onset years						
0–54	98	4.11	Reference			
55–59	58	3.96	1.70	1.23	2.35	<0.0001
60–64	51	4.06	1.41	1.00	1.97	0.05
65–69	71	3.95	1.86	1.37	2.52	<0.0001
70–74	93	3.38	2.62	1.97	3.48	<0.0001
75–79	92	3.12	3.67	2.76	4.89	<0.0001
80–84	47	2.50	3.64	2.57	5.16	<0.0001
≥85	41	2.05	6.90	4.78	9.96	<0.0001
**IPF-CS overall^#^**						
Sex						
Female	2011	2.87	Reference			
Male	4274	2.71	1.28	1.21	1.35	<0.0001
Age at onset years						
0–54	186.00	3.87	Reference			
55–59	184.00	3.61	1.36	1.11	1.67	<0.0001
60–64	340.00	3.41	1.51	1.27	1.81	<0.0001
65–69	658.00	3.22	1.91	1.62	2.25	<0.0001
70–74	1030	2.88	2.23	1.91	2.61	<0.0001
75–79	1314	2.79	2.48	2.13	2.89	<0.0001
80–84	1325	2.42	3.03	2.60	3.54	<0.0001
≥85	1248	1.88	4.30	3.69	5.02	<0.0001
**IPF-CS narrow definition**						
Sex						
Female	170	2.86	Reference			
Male	358	2.77	1.08	0.90	1.30	0.41
Age at onset years						
0–54	12	3.89	Reference			
55–59	15	3.58	1.22	0.57	2.62	0.61
60–64	26	3.42	1.51	0.76	3.00	0.24
65–69	55	3.23	1.65	0.88	3.09	0.12
70–74	96	2.87	1.64	0.90	3.00	0.11
75–79	119	2.78	1.89	1.04	3.44	0.04
80–84	112	2.42	2.34	1.29	4.26	0.01
≥85	93	1.87	3.19	1.74	5.83	<0.0001
**IPF-CS broad definition^#^**						
Sex						
Female	2126	3.18	Reference			
Male	3139	3.07	1.29	1.22	1.36	<0.0001
Age at onset years						
0–54	174	3.84	Reference			
55–59	169	3.98	1.33	1.08	1.65	<0.0001
60–64	314	3.10	1.53	1.27	1.84	<0.0001
65–69	603	2.99	1.93	1.63	2.28	<0.0001
70–74	934	2.99	2.23	1.90	2.62	<0.0001
75–79	1195	2.96	2.47	2.11	2.90	<0.0001
80–84	1213	2.50	3.02	2.58	3.54	<0.0001
≥85	1155	2.00	4.17	3.55	4.90	<0.0001

HR: hazard ratio; ILD: interstitial lung disease; IPF-CS: idiopathic pulmonary fibrosis clinical syndrome; CTD: connective tissue disease; LCI: lower confidence interval; UCI: upper confidence interval. **^#^**: to satisfy the hazard proportional assumption, in these cases we applied the Cox proportional hazard model stratified by year of diagnosis (see supplementary material E4 for details).

After adjusting for age and sex, comorbidities, BMI, smoking status and steroid therapy, we found that within people with IPF-CS, the survival was slightly worse for people in the broad coding category (HR 0.90, 95% CI 0.82–0.98, p=0.021). We found that people with CTD-ILDs and HP had better survival compared to people with IPF-CS (HR 0.80, 95% CI 0.76–0.83, p<0.0001, HR 0.74, 95% CI 68–0.81, p<0.0001).

Mortality rates for each ILD subtype and IPF-CS codeset bands are summarised in the supplementary material 1, E8.

#### Controls

The mean follow-up time in our control cohort after index data was 3.34±2.62 years, and we found an overall median survival time of 8.05 years (IQR 3.67–9.98). Overall, a diagnosis of ILD at least doubled the risk for mortality both before (crude HR 2.59, 95% CI 2.52–2.55) and after propensity score weighting (adjusted HR 2.10, 95% CI 2.04–2.16). The weighted Kaplan–Meier curve comparing cases and controls is available in the supplementary material 1, E9.

## Discussion

Our large population-based study provides contemporary national estimates of the burden of IPF-CS, CTD-ILD and HP in the UK from 2010 to 2019. These results suggest that nearly 20 000 people in the UK are diagnosed with IPF, CTD-ILD or HP with >8000 deaths each year [12]. Among them, almost all of the IPF cases and the progressive forms of CTD-ILDs and HPs would be eligible for treatment with antifibrotic drugs. This burden in terms of incidence appears now to be stable over time.

We found a change in coding practice in primary care for IPF-CS, with a marked decrease in specific codes for IPF in comparison to previous studies, and a shift towards more general codes included in the broad codeset band. We found that people with IPF-CS had a poorer prognosis compared to the other diseases, with those fulfilling the broadest IPF-CS definition doing worse. Finally, we observed that a diagnosis of ILD increased the risk of death and halved the life expectancy compared to the general population.

Our study has several strengths. First, this is the first study assessing the epidemiology of three of the most common ILDs and, to our knowledge, the very first analysing survival in ILDs other than IPF. It is also the first study evaluating ILD epidemiology in the OPCRD dataset, and it found mean IPF-CS incidence rates in line with other previous studies from similar data sources [13]. OPCRD is a unique dataset that allows the collection of a large amount of data from a single source and is representative of the UK population [7]. Studies of this nature and magnitude are not possible using only cases collected at centres in secondary care. Furthermore, the OPCRD has been used for quality improvement programmes in other diseases, which means it may offer the opportunity in the future to improve the care with further intervention studies with this resource. Second, we provided estimates not only for IPF-CS, but also for other ILDs potentially eligible for antifibrotics, and this information will be helpful for those planning ILD services and provisions of antifibrotic drugs.

Third, adding matched controls to our survival analysis, we provided evidence of the impact of a diagnosis of ILD on survival, enabling benchmarking of such patients’ burden compared to other diseases, such as cancers.

The main potential weakness of our study is related to the validity of the diagnosis codes in primary care. The validity of primary care data for IPF-CS diagnosis has been evaluated in other datasets similar to OPCRD and found to be good [11], and also CTD-ILD definition has been validated [10], but this issue has not been tackled in HP. This might have led to underestimation of some cases, as it is highly unlikely that an ILD is coded in primary care without a secondary care ascertainment. We also excluded the years of the COVID-19 pandemic because of the potential misclassification of SARS-CoV-2 pneumonia. Furthermore, although we included antifibrotic codes in the narrow definition as they were prescribed only in IPF during our study period, the ability to capture their prescription in primary care data is poor, as they are prescribed in secondary care. Finally, as we used the whole population to ensure comparability with previous studies, this may have led to an underestimate of the IPF incidence.

Our mean incidence data are consistent with previous studies carried out in the UK using similar methodology. Gupta *et al*. [6] recently reported IPF-CS incidence rates spanning from 3.0–61 to 22.4–28.6 per 100 000 person-years in 2008–2018. Navaratnam *et al*. [1] found an IPF-CS incidence rate ranging from 5.77 to 8.04 in 2001–2008, with an increasing trend over time. Strongman *et al*. [13] detected an overall incidence rate of 2.85 and 8.65 per 100 000 person-years using the narrow and the broad codeset band in 2000–2012. They also observed an increasing trend of the broad definition over time, whilst the narrow definition decreased during the analysis period. This observation is reflected in our findings, as we detected a lower incidence using the narrow definition and a higher incidence rate applying the broader codeset band. Of note the incidence rates we found for IPF are roughly double those reported in the early part of the 21st century, and the rates appear to have become stable over time. It is not clear whether the previously reported increases in disease incidence over time were due to a true increase in disease incidence, a progressive increase in disease recognition and diagnosis, or a mixture of both. However, the stabilisation of rates that we now observe suggests that the main driver of these increases has now stabilised.

There are fewer studies of the incidence of CTD-ILDs and HP, and the results are more varied, and as shown in a systematic review by Gupta *et al*. [14], is mainly due to different case selection criteria and the wide variability of clinical settings. However, Duchemann *et al*. [15] found incidence rates that are quite similar to ours.

Our results for the median survival of people with IPF-CS are comparable to previous studies. Strongman *et al*. and Navaratnam *et al*. found a median survival of around 3.0 years in people with IPF-CS [13–17]. Furthermore, in the USA, Raghu *et al*. [16] calculated a median survival time of 3.9 years for Medicare beneficiaries aged >65 years. Similarly, using primary care data, Navaratnam *et al*. [17] found that median survival times for IPF-CS and the CTD-ILDs were 3.1 years *versus* 6.5 years, respectively.

Due to different methodology and follow-up times, other previous studies from secondary care regarding CTD-ILDs and HP survival are not directly comparable with ours. In fact, Vahidy *et al*. [18] assessed the survival rates in people with CTD-ILDs and found a median survival time of 15.9 years. Similarly, WAlscher *et al*. [19] analysing the impact of comorbidities on survival in a cohort of patients with chronic HP found a median survival time of around 15 years. The longer median survival reported in these studies might partly be due to an earlier diagnosis and, possibly, of prevalent cases. In contrast, Vourlekis *et al*. [20] found a median survival time ranging from ∼7 to ∼12 years in fibrotic and non-fibrotic HP, which is close to the results of our study.

The trend we observed to use more general codes to define IPF-CS in recent years may reflect a change in secondary care clinical practice whereby the presence of lung fibrosis, and whether it is progressive or not, is more important than a specific diagnosis of IPF for treatment purposes. Certainly this shift has been noted before by Morgan *et al.* [11], who also observed the substitution of obsolete terms such as “cryptogenic fibrosing alveolitis” with more updated definitions (*i.e.* “idiopathic pulmonary fibrosis”). Nevertheless, considering the shifting in coding and that both narrow and broad IPF-CS definitions carried the same prognosis, the true estimate of new IPF cases might now be underestimated using only the more specific narrow definition.

### Conclusion

The epidemiological burden of the fibrotic ILDs is considerable with >20 000 new cases each year in UK, and many of these people will be eligible for treatment with antifibrotic drugs. The incidence of these ILDs appears to have stabilised over time. The median survival for people with these diseases remains poor and in general is half that of people of similar age and sex without the disease.

## Supplementary material

10.1183/23120541.00823-2024.Supp1 **Please note:** supplementary material is not edited by the Editorial Office, and is uploaded as it has been supplied by the author.
Supplementary material 00823-2024.SUPPLEMENT
Supplementary material 00823-2024.SUPPLEMENT2

## Data Availability

The data set supporting the conclusions of this article was derived from the Optimum Patient Care Research Database (www.opcrd.co.uk). The authors do not have permission to give public access to the study data set; researchers may request access to OPCRD data for their own purposes. Access to OPCRD can be made *via* the OPCRD website (https://opcrd.co.uk/our-database/ data-requests/) or *via* the enquiries email info@opcrd.co.uk.
