# Supplementary material for: Incidence and survival of interstitial lung diseases in the UK in 2010–2019
Source: ERJ Open Res. 2025 Mar 3;11(2):00823-2024. doi: 10.1183/23120541.00823-2024 (PMC11874205; doi:10.1183/23120541.00823-2024)
Supplement: Supplementary file 1 [file 00823-2024.SUPPLEMENT.pdf]

Supplementary Materials 1

- Supplementary material E1 page 2
- Supplementary material E2 page 3
- Supplementary material E3 page 5
- Supplementary material E4 page 6
- Supplementary material E5 page 7
- Supplementary material E6 page 8
- Supplementary material E7 page 9
- Supplementary material E8 page 10
- Supplementary material E9 page 11
- References page 12

Supplementary Material E1

Supplementary Material E1: demographic characteristics of each mid-year population used as denominator for incidence rates calculation.

**Sex**	**Age (years)**	**2010**	**2011**	**2012**	**2013**	**2014**	**2015**	**2016**	**2017**	**2018**	**2019**
Female											
	0-54	1905585	1995719	2058987	2134484	2228315	2290237	2333319	2414134	2543117	2682298
	55-59	195763	204092	210091	218036	228491	237774	244005	253805	269233	285701
	60-64	212923	213661	206311	205991	208807	209129	212259	217876	229405	242359
	65-69	158277	179148	197471	209298	216864	222082	216670	209120	212622	218036
	70-74	112873	120915	129236	138687	152636	160357	176165	194032	209362	219696
	75-79	68162	78466	86599	96044	105557	110438	114218	122227	134621	150714
	80-84	31819	38708	44276	50505	57707	63439	69856	77828	89602	101278
	≥85	14518	18693	21676	25602	31269	36043	41462	49413	61740	76999
Male											
	0-54	1992369	2092985	2169337	2256039	2360763	2438353	2495569	2584320	2718822	2855980
	55-59	184683	193796	202049	211675	223235	234764	242837	253887	270957	288832
	60-64	191797	194397	190074	191609	196945	199136	203076	210751	223878	237573
	65-69	137782	157520	175717	187793	195590	201600	198390	193368	198410	205563
	70-74	90912	98963	107198	117083	131221	139751	154859	172402	187697	198052
	75-79	49266	58225	65171	74225	82812	88034	92146	99767	111826	127996
	80-84	19597	24417	28766	33331	39492	44438	49954	56647	67087	76956
	≥85	7511	9896	11549	13814	17146	19967	23348	28382	36059	46168
Total		5366326	5669705	5892959	6150402	6459704	6675575	6844785	7109577	7528379	7968033

Supplementary Material E2

Supplementary Methods

Matching

We identified our matched controls using time-dependent incidence density sampling. This method assigned equal length of observation to cases and matched controls to ensure equal time windows of exposure[2].

Covariates

We extracted the following covariates: diagnosis date and codes, registration date, prescription dates and codes, comorbidities dates and codes (i.e. cerebrovascular diseases, including stroke and transient ischemic attack, ischemic heart disease, chronic kidney disease stage III-V, liver disease, diabetes mellitus, peptic ulcer, peripheral artery disease, chronic obstructive pulmonary disease, psychiatric disorders, dementia, and malignancy), smoking history, baseline body mass index (BMI), registry leaving date and death date, steroids (i.e. at least 3 prescriptions) exposure. Codes lists are reported in the Supplements 2 (E3-E4).

Statistical analysis

We performed an initial descriptive analysis of our cases, stratifying our results by age (in 5 year age bands from 55 to 85 years) and sex.

We used multiplicative interaction terms to test for modification by age and sex, grouping age into a binary variable (<70 years old and ≥70years old) for these analyses.

Proportional hazard model assumption verification

We tested the satisfaction of the Cox proportional hazard assumption by using the Schoenfeld and scaled Schoenfeld residuals. To do this we used the following STATA command:

```
estat phtest, detail
```

fit to the full multivariate model

→ i.e. `stcox` *varlist*

In case we obtained a significant p value (violated Cox assumptions), we then stratified the model by the non-proportional predictor, adding the option strata(year)

→ i.e. `stcox` *varlist*, `strata`(*non-proportional var*)

Segmented regression model

The model assesses the overall trends in mortality, initially with no joinpoints, and tests for significant changes in the model with sequential addition of points up to five where there is significant change in the slope of the line. The model also computes an estimated average annual percent change for each trend by fitting a regression line to the natural logarithm of the rates. The log-linear transformation allowed us to approximate normal distributions and, by estimating the annual percentage change, we were able to assess change in mortality trend at a constant percentage per year.

Propensity score weighting

To ensure the comparability of cases and controls balancing baseline features, we run a subsequent propensity score weighted analysis in our matched cohort study.

The propensity score weighting included:

1. demographic factors: age, sex, smoking status, and BMI;
2. comorbidities: all the comorbidities included in the full model;
3. exposure to steroids.

We used STATA v.18 (StataCorp, Texas, USA) for data management and statistical analyses and the Surveillance Epidemiology and End Results statistical software (Joinpoint Regression Program, version 5.0) for the segmented regression.

We wrote this manuscript in accordance with the Reporting of studies Conducted using Observational Routinely-collected health Data (RECORD) statement[3].

Supplementary Material E3

Supplementary Materials E3: Baseline clinical and demographic data of the controls included in the study

	Hypersensitivity pneumonitis	CTD-ILDs	IPF-CS narrow definition	IPF-CS broad definition
	n=5,801	n=16,738	n=2,930	n=34,699
**Age at diagnosis, mean (SD)**				
**Sex, female, n (%)**	60.9 (15.1)	70.4 (12.1)	75.4 (9.6)	74.3 (11.3)
	3,115 (53.7%)	9,233 (55.2%)	992 (33.9%)	12,957 (37.3%)
**Smoking status, n (%)**				
Non smoker	2,813 (48.5%)	8,297 (49.7%)	1,278 (43.9%)	15,839 (45.8%)
Current smoker	772 (15.2%)	1,894 (11.3%)	276 (9.5%)	3,634 (10.5%)
Former smoker	1,311 (25.8%)	5,164 (30.9%)	1,023 (35.1%)	11,979 (34.6%)
Passive smoker	6 (0.1%)	16 (0.1%)	0 (0.0%)	23 (0.1%)
Missing data	615 (10.6%)	1,321 (7.9%)	335 (11.5%)	3,113 (9.0%)
**BMI category, n (%)**				
Underweight	330 (5.7%)	669 (4.0%)	76 (2.6%)	1,146 (3.3%)
Normal weight	1,723 (29.7%)	4,937 (29.5%)	782 (26.7%)	9,819 (28.4%)
Overweight	1,647 (28.4%)	5,540 (33.1%)	1,043 (35.6%)	12,292 (35.5%)
Obese	2,106 (36.3%)	5,590 (33.4%)	1,025 (35.0%)	4,256 (32.7%)
**Cerebrovascular disease, n (%)**	273 (4.7%)	1,405 (8.4%)	308 (10.5%)	3,573 (10.3%)
**Chronic kidney disease, n (%)**	481 (8.3%)	2,327 (13.9%)	522 (17.8%)	6,072 (17.5%)
**Chronic obstructive pulmonary disease, n (%)**	232 (4.0%)	921 (5.5%)	176 (6.0%)	2,359 (6.8%)
**Ischaemic heart disease, n (%)**	516 (8.9%)	2,377 (14.2%)	607 (20.7%)	6,384 (18.4%)
**Dementia, n (%)**	104 (1.8%)	586 (3.5%)	132 (4.5%)	1,561 (4.5%)
**Diabetes mellitus, n (%)**	621 (10.7%)	2,427 (14.5%)	498 (17.0%)	5,795 (16.7%)
**Liver disease, n (%)**	99 (1.7%)	586 (3.5%)	56 (1.9%)	729 (2.1%)
**Current/previous malignancy, n (%)**	406 (7.0%)	1,925 (11.5%)	372 (12.7%)	4,372 (12.6%)
**Peptic ulcer, n (%)**	180 (3.1%)	837 (5.0%)	158 (5.4%)	2,081 (6.0%)
**Peripheral artery disease, n (%)**	278 (4.8%)	1,105 (6.6%)	267 (9.1%)	3,123 (9.0%)
**Psychiatric disorders, n (%)**	52 (0.9%)	184 (1.1%)	29 (1.0%)	347 (1.0%)
**Steroid exposure, n (%)**	464 (8.0%)	1,607 (9.6%)	267 (9.1%)	3,678 (10.6%)

BMI: body mass index; CTD: connective tissue disease; ILDs: Interstitial lung diseases; IPF-CS: idiopathic pulmonary fibrosis-clinical syndrome.

Supplementary Material E4

Supplementary Table E4: age- and sex-stratified incidence rates along with age- and sex-adjusted IRRs; age- and sex- adjusted HRs for each ILD category .

		Incidence (cases per 100.000 person-years)						
		Rate	LCI	UCI	IRR	LCI	UCI	p value
CTD-ILDs								
	Sex							
	Female	8.50	8.19	8.82	Reference			
	Male	7.42	7.13	7.72	1.06	1.00	1.12	0.03
	Age at onset (years)							
	18-54	1.11	1.02	1.21	Reference			
	55-59	7.09	6.35	7.90	6.41	5.58	7.36	<0.01
	60-64	12.70	11.64	13.82	11.38	10.08	12.84	<0.01
	65-69	19.45	18.09	20.89	17.52	15.67	19.59	<0.01
	70-74	30.58	28.63	32.62	28.02	25.16	31.21	<0.01
	75-79	50.66	47.53	53.95	46.54	41.82	51.79	<0.01
	80-84	68.12	63.26	73.26	63.54	56.75	71.16	<0.01
	≥85	82.71	75.54	90.37	78.60	69.41	89.02	<0.01
Hypersensitivity pneumonitis								
	Sex							
	Female	2.82	2.64	3.01	Reference			
	Male	2.44	2.28	2.62	0.95	0.87	1.05	0.34
	Age at onset (years)							
	18-54	1.15	1.05	1.25	Reference			
	55-59	4.13	3.56	4.75	3.59	3.04	4.23	<0.01
	60-64	4.81	4.17	5.52	4.17	3.55	4.90	<0.01
	65-69	5.55	4.84	6.34	4.82	4.11	5.64	<0.01
	70-74	7.80	6.84	8.87	6.82	5.85	7.95	<0.01
	75-79	9.29	7.97	10.76	8.12	6.85	9.62	<0.01
	80-84	10.51	8.65	12.65	9.23	7.52	11.32	<0.01
	≥85	10.66	8.19	13.63	9.41	7.24	12.22	<0.01
IPF-CS overall								
	Sex							
	Female	12.98	12.60	13.38	Reference			
	Male	23.22	22.70	23.74	2.32	2.24	2.41	<0.01
	Age at onset (years)							
	18-54	1.33	1.23	1.44	Reference			
	55-59	10.25	9.35	11.21	7.85	6.97	8.85	<0.01
	60-64	19.91	18.59	21.31	15.21	13.71	16.88	<0.01
	65-69	34.56	32.74	36.46	26.76	24.33	29.43	<0.01
	70-74	66.60	63.72	69.58	53.17	48.59	58.19	<0.01
	75-79	125.70	120.73	130.82	102.29	93.64	111.75	<0.01
	80-84	212.91	204.24	221.86	180.82	165.41	197.67	<0.01
	≥85	334.20	319.63	349.27	302.74	276.47	331.51	<0.01
IPF-CS narrow definition								
	Sex							
	Female	0.90	0.80	1.00	Reference			
	Male	1.88	1.73	2.03	2.74	2.38	3.15	<0.01
	Age at onset (years)							
	18-54	0.06	0.04	0.09	Reference			
	55-59	0.58	0.38	0.84	9.53	5.64	16.09	<0.01
	60-64	1.24	0.93	1.62	20.21	12.83	31.83	<0.01
	65-69	2.67	2.18	3.24	44.38	29.40	66.98	<0.01
	70-74	6.08	5.23	7.02	105.18	71.08	155.65	<0.01
	75-79	10.44	9.04	11.99	184.91	125.24	273.03	<0.01
	80-84	16.89	14.51	19.55	316.33	213.57	468.54	<0.01
	≥85	23.68	19.92	27.94	481.19	322.13	718.81	<0.01
IPF-CS broad definition								
	Sex							
	Female	12.09	11.72	12.47	Reference			
	Male	21.34	20.84	21.84	2.29	2.20	2.38	<0.01
	Age at onset (years)							
	18-54	1.27	1.17	1.37	Reference			
	55-59	9.67	8.80	10.61	7.77	6.87	8.78	<0.01
	60-64	18.68	17.39	20.03	14.97	13.45	16.65	<0.01
	65-69	31.89	30.14	33.72	25.89	23.47	28.56	<0.01
	70-74	60.52	57.78	63.37	50.63	46.14	55.56	<0.01
	75-79	115.26	110.50	120.17	98.25	89.71	107.60	<0.01
	80-84	196.02	187.70	204.61	174.19	158.94	190.90	<0.01
	≥85	310.53	296.48	325.06	293.90	267.73	322.64	<0.01

CTD: connective tissue disease; HR: hazard ratio; ILD: interstitial lung disease; IRR: incidence rate ratio; IPF-CS: idiopathic pulmonary fibrosis clinical syndrome; LCI: lower confidence interval; UCI: upper confidence interval.

Supplementary Material E5

Segmented regression analysis – results

After running the segmented regression analysis, we found the following results:

- Overall ILDs: joinpoint 2014, left AAPC -2.25, 95%CI -5.87- -0.52, right AAPC 1.95, 95%CI 0.84-5.00.
- Overall IPF-CS: no joinpoint, AAPC 0.61, 95%CI -0.38-1.68.
- IPF-CS narrow definition: no joinpoint, AAPC -1.53, 95%CI -7.01-4.12.
- IPF-CS broad definition: no joinpoint, AAPC 0.77, 95%CI -0.25-1.90.
- CTD-ILDs: joinpoint 2015, left AAPC -3.14, 95%CI -6.08-1.61, right AAPC 3.28, 95%CI 1.24-7.50

Supplementary Material E6

Supplementary Material E6: Kaplan Meier survival estimates comparing different ILD categories and IPF-CS subgroups.

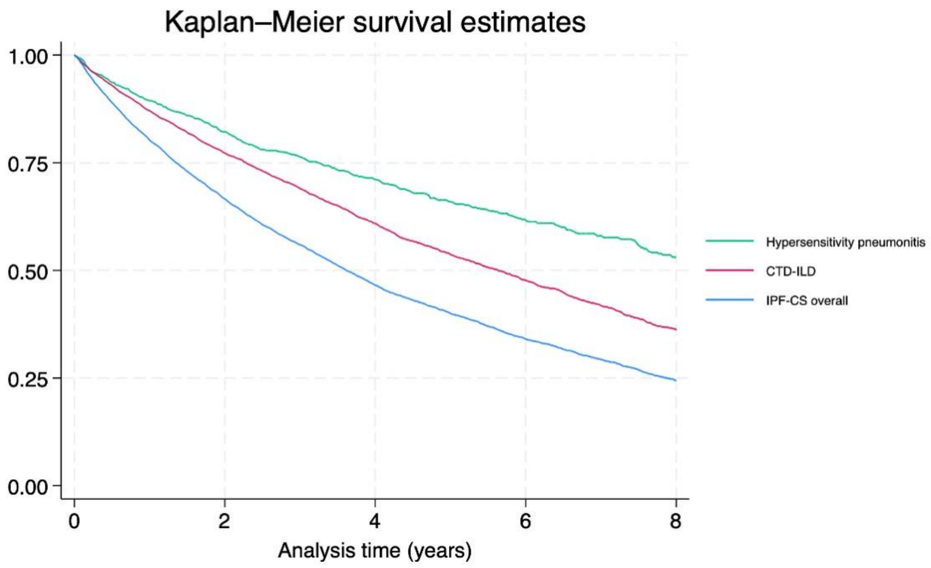

A)

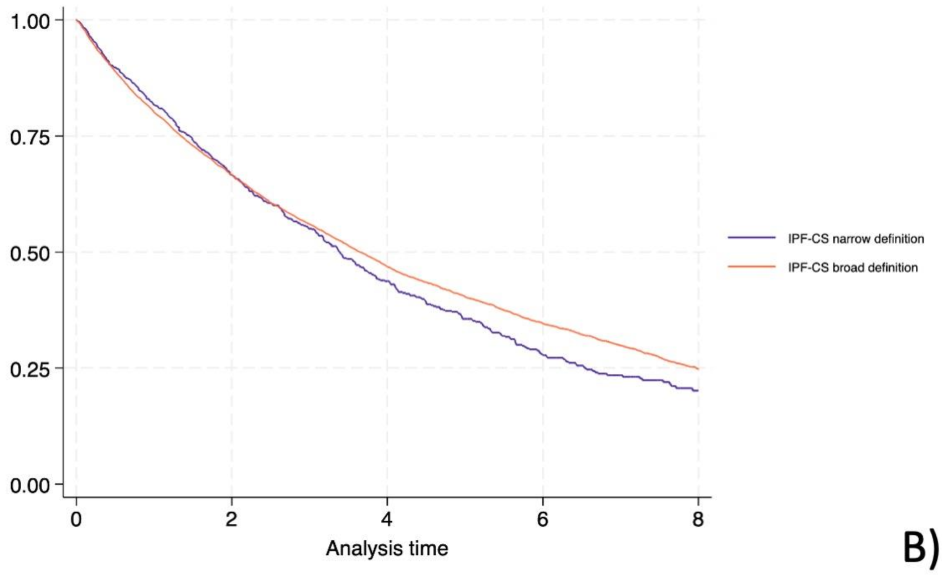

B)

Supplementary Material E7

Supplementary Material E7: number of deaths and relative person-time stratified by age and sex; age- and sex- adjusted HRs for each ILD category and each IPF-CS subgroup.

		Row data		Survival			
		Deaths (n)	*Person-time (Years)*	*HR*	*95% CI LCI*	*UCI*	*p value*
CTD-ILDs							
	Sex						
	Female	1118	3·49	Reference			
	Male	1199	3·33	1·22	1·12	1·32	<0·0001
	Age at onset (years)						
	18-54	119	4·32	Reference			
	55-59	89	4·31	1·16	0·88	1·53	0·20
	60-64	186	4·11	1·57	1·25	1·97	<0·0001
	65-69	310	3·89	1·99	1·61	2·46	<0·0001
	70-74	401	3·41	2·43	1·98	2·98	<0·0001
	75-79	500	3·10	3·17	2·59	3·87	<0·0001
	80-84	401	22·66	4·03	3·28	4·95	<0·0001
	≥85	311	2·13	5·93	4·79	7·34	<0·0001
Hypersensitivity pneumonitis							
	Sex						
	Female	296	3·69	Reference			
	Male	553	3·68	1·03	0·87	1·21	0·77
	Age at onset (years)						
	18-54	98	4·11	Reference			
	55-59	58	3·96	1·70	1·23	2·35	<0·0001
	60-64	51	4·06	1·41	1·00	1·97	0·05
	65-69	71	3·95	1·86	1·37	2·52	<0·0001
	70-74	93	3·38	2·62	1·97	3·48	<0·0001
	75-79	92	3·12	3·67	2·76	4·89	<0·0001
	80-84	47	2·50	3·64	2·57	5·16	<0·0001
	≥85	41	2·05	6·90	4·78	9·96	<0·0001
IPF-CS overall*							
	Sex						
	Female	2011	2·87	Reference			
	Male	4274	2·71	1·28	1·21	1·35	<0·0001
	Age at onset (years)						
	18-54	186·00	3·87	Reference			
	55-59	184·00	3·61	1·36	1·11	1·67	<0·0001
	60-64	340·00	3·41	1·51	1·27	1·81	<0·0001
	65-69	658·00	3·22	1·91	1·62	2·25	<0·0001
	70-74	1,030	2·88	2·23	1·91	2·61	<0·0001
	75-79	1,314	2·79	2·48	2·13	2·89	<0·0001
	80-84	1,325	2·42	3·03	2·60	3·54	<0·0001
	≥85	1,248	1·88	4·30	3·69	5·02	<0·0001
IPF-CS narrow definition							
	Sex						
	Female	170	2·86	Reference			
	Male	358	2·77	1·08	0·90	1·30	0·41
	Age at onset (years)						
	18-54	12	3·89	Reference			
	55-59	15	3·58	1·22	0·57	2·62	0·61
	60-64	26	3·42	1·51	0·76	3·00	0·24
	65-69	55	3·23	1·65	0·88	3·09	0·12
	70-74	96	2·87	1·64	0·90	3·00	0·11
	75-79	119	2·78	1·89	1·04	3·44	0·04
	80-84	112	2·42	2·34	1·29	4·26	0·01
	≥85	93	1·87	3·19	1·74	5·83	<0·0001
IPF-CS broad definition*							
	Sex						
	Female	2126	3·18	Reference			
	Male	3139	3·07	1·29	1·22	1·36	<0·0001
	Age at onset (years)						
	18-54	174	3·84	Reference			
	55-59	169	3·98	1·33	1·08	1·65	<0·0001
	60-64	314	3·10	1·53	1·27	1·84	<0·0001
	65-69	603	2·99	1·93	1·63	2·28	<0·0001
	70-74	934	2·99	2·23	1·90	2·62	<0·0001
	75-79	1,195	2·96	2·47	2·11	2·90	<0·0001
	80-84	1,213	2·50	3·02	2·58	3·54	<0·0001
	≥85	1,155	2·00	4·17	3·55	4·90	<0·0001

CTD: connective tissue disease; HR: hazard ratio; ILD: interstitial lung disease; IPF-CS: idiopathic pulmonary fibrosis clinical syndrome; LCI: lower confidence interval; UCI: upper confidence interval.

* To satisfy the hazard proportional assumption, in these cases we applied the Cox proportional hazard model stratified by year of diagnosis (Supplementary Material E4 for detail).

- HP: joinpoint 2015, left AAPC -6.14, 95%CI -19.02- -1.00, right AAPC 7.14, 95%CI 0.26-24.26.

Supplementary Material E8

Supplementary Material E8: death rates per 1000 person-years along with 95% CI for each ILD category and IPF subgroup.

	**Person-time** *(days)*	**Rate** *(per 100,000)*	**95% CI** *LCI*	*UCI*
Hypersensitivity pneumonitis	*27,359.95*	*4,579.69*	*4,333.00*	*4,840.42*
CTD-ILDs	76,175.77	7,102.00	6,915.25	7,293.79
IPF-CS narrow definition	*12,389.59*	*9,532.20*	*9,003.77*	*10,091.65*
IPF-CS broad definition	142,090.76	9,641.72	9,481.62	9,804.54
IPF-CS overall	154,480.34	9,632.94	9,479.41	9,788.96

CTD: connective tissue disease; ILD: interstitial lung disease; IPF-CS: idiopathic pulmonary fibrosis clinical syndrome LCI: lower confidence interval; UCI: upper confidence interval.

Supplementary Material E9

Supplementary Material E9a: standardized mean difference of comorbidities distribution in cases and controls before and after propensity score weighting

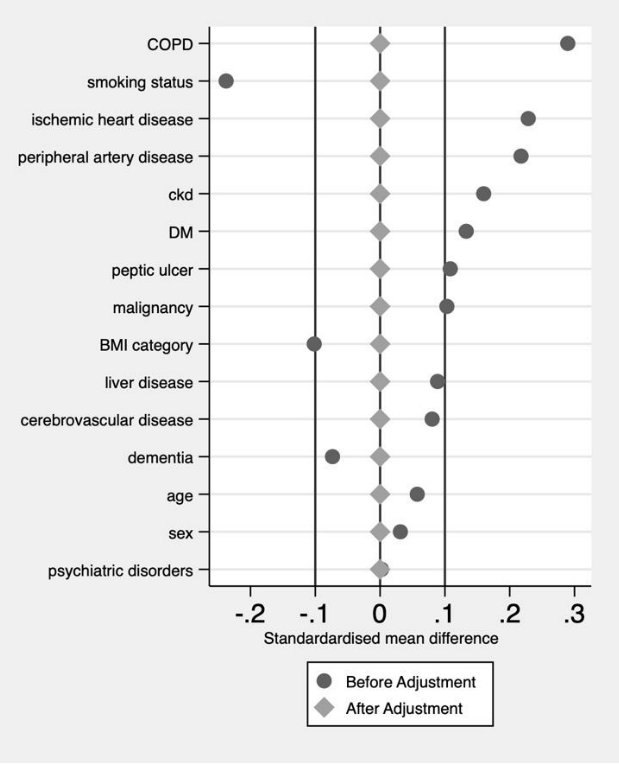

Supplementary Material E9b: Weighted Kaplan Meier survival estimates comparing ILD cases and their matched controls after propensity score weighting.

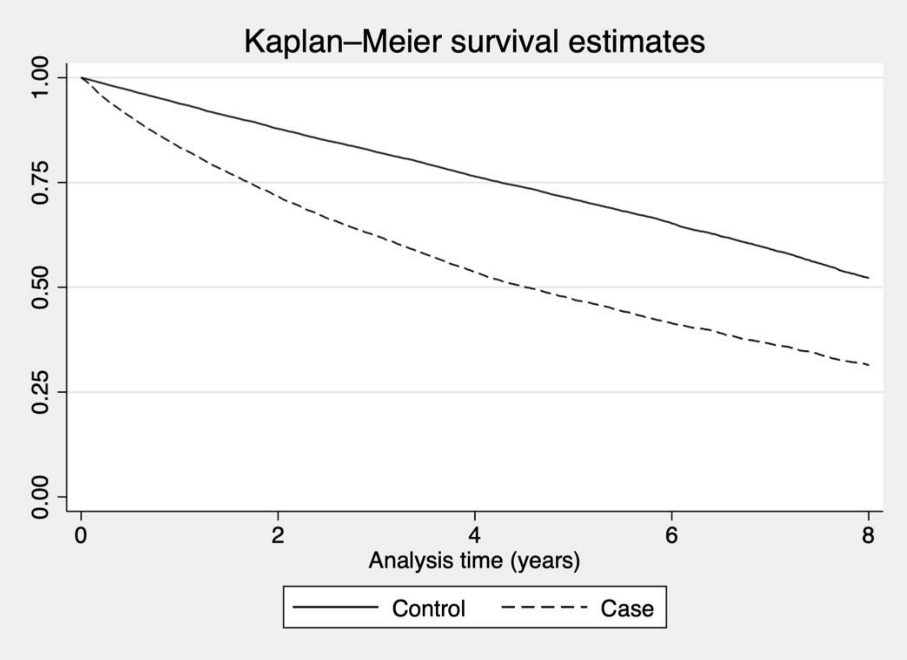

References

1 Stuart-Buttle CD, Read JD, Sanderson HF, Sutton YM. A language of health in action: Read Codes, classifications and groupings. *Proc AMIA Annu Fall Symp* 1996; : 75–9.

2 Suissa S, Dell'aniello S, Vahey S, Renoux C. Time-window bias in case-control studies: statins and lung cancer. Epidemiology. 2011 Mar;22(2):228-31.

3 Benchimol EI, Smeeth L, Guttmann A, *et al.* The REporting of studies Conducted using Observational Routinely-collected health Data (RECORD) Statement. *PLoS Med* 2015; **12**: 1–22.
