# Supplementary material for: Incidence and survival of interstitial lung diseases in the UK in 2010–2019
Source: ERJ Open Res. 2025 Mar 3;11(2):00823-2024. doi: 10.1183/23120541.00823-2024 (PMC11874205; doi:10.1183/23120541.00823-2024)
Supplement: Supplementary file 2 [file 00823-2024.SUPPLEMENT2.pdf]

Supplementary Materials 2

- Supplementary material E1 page 2
- Supplementary material E2 page 5
- Supplementary material E3 page 34
- Supplementary material E4 page 36

Supplementary Material E1

Supplementary Material E1: Read and SNOMED codes used to define HP, CTD-ILDs, IPF-CS, occupational ILDs, and drug-induced ILDs

Disease category	Code ID	Code term	Code type
Hypersensitivity pneumonitis			
	196144009	(Emphysema [interstitial]/[mediastinal])/(pneumomediastinum)	SNOMED
	266399001	(Extrinsic allergic alveolitis) or (farmers lung) or (pneumonitis, allergic NOS)	SNOMED
	205237003	[X]Hypersensitivity pneumonitis due to other organic dusts	SNOMED
	Hyu43	[X]Hypersensitivity pneumonitis due to other organic dusts	Read
	195990006	Air-conditioner and humidifier lung	SNOMED
	48347002	Air-conditioner and humidifier lung	SNOMED
	80550016	Air-conditioner and humidifier lung	SNOMED-DescriptionID
	H35y8	Air-conditioner and humidifier lung	Read
	H35z.	Allergic alveolitis and pneumonitis NOS	Read
	H35z0	Allergic extrinsic alveolitis NOS	Read
	H351.	Bagassosis	Read
	69339004	Bird-fanciers lung	SNOMED
	H352.	Bird-fanciers lung	Read
	H352z	Bird-fanciers lung NOS	Read
	12088005	Budgerigar-fanciers disease	SNOMED
	H3520	Budgerigar-fanciers lung	Read
	85761009	Byssinosis (disorder)	SNOMED
	404807005	Cheese-washers disease	SNOMED
	H35y0	Cheese-washers lung	Read
	16623004	Coffee-workers disease	SNOMED
	H35y1	Coffee-workers lung	Read
	41553006	Detergent workers' lung	SNOMED
	H35..	Extrinsic allergic alveolitis	Read
	18690003	Farmers lung	SNOMED
	H350.	Farmers lung	Read
	189221016	Hypersensitivity pneumonitis	SNOMED-DescriptionID
	37471005	Hypersensitivity pneumonitis	SNOMED
	H35z1	Hypersensitivity pneumonitis NOS	Read
	25897000	Malt workers lung	SNOMED
	H354.	Malt workers lung	Read
	52333004	Mushroom workers lung	SNOMED
	H355.	Mushroom workers lung	Read
	H35y.	Other allergic alveolitis	Read
	H35yz	Other allergic alveolitis NOS	Read
	85407005	Pigeon-fanciers disease	SNOMED
	H3521	Pigeon-fanciers lung	Read
	195989002	Pituitary snuff-takers disease	SNOMED
	H35y5	Pituitary snuff-takers disease	Read
	23315001	Sequoiosis	SNOMED
	H35y6	Sequoiosis	Read
	67242002	Sugar cane workers hypersensitivity pneumonitis	SNOMED
	233702002	Summer-type hypersensitivity pneumonitis	SNOMED
	X102T	Summer-type hypersensitivity pneumonitis	Read
	56968009	Wood dust pneumonitis	SNOMED
CTD-ILDs			
	196144009	(Emphysema [interstitial]/[mediastinal])/(pneumomediastinum)	SNOMED
	201813004	(Rheumatoid lung) or (Caplans syndrome) or (fibrosing alveolitis associated with rheumatoid arthritis)	SNOMED
	10713006	Fibrosing alveolitis associated with rheumatoid arthritis	SNOMED

Overall	Narrow definition	Intermediate definition	Broad definition	Code	Description	Coding system
				311496011	Fibrosing alveolitis associated with rheumatoid arthritis	SNOMED-DescriptionID
				X701k	Fibrosing alveolitis associated with rheumatoid arthritis	Read
				427123006	Interstitial lung disease due to collagen vascular disease	SNOMED
				XaXlF	Interstitial lung disease due to collagen vascular disease	Read
				711379004	Interstitial lung disease due to connective tissue disease	SNOMED
				XaXlJ	Interstitial lung disease due to connective tissue disease	Read
				737181009	Interstitial lung disease due to systemic disease	SNOMED
IPF-CS						
Overall	Narrow definition	Intermediate definition	Broad definition			
X	X			196144009	(Emphysema [interstitial]/[mediastinal])/(pneumomediastinum)	SNOMED
X	X			XE0Yb	Cryptogenic fibrosing alveolitis	Read
X	X			155624004	Fibrosing alveolitis-idiopath.	SNOMED
X	X			XE0Zr	Idiopath. Fibrosing alveolitis (& Hamman-Rich syndrome)	Read
X	X			H563z	Idiopathic fibrosing alveolitis NOS	Read
X	X			H563.	Idiopathic fibrosing alveolitis/Cryptogenic fibrosing alveolitis/Hamman-Rich syndrome/Acute interstitial pneumonia	Read
X	X			28168000	Idiopathic pulmonary fibrosis	SNOMED
X	X			2989702012	Idiopathic pulmonary fibrosis	SNOMED-DescriptionID
X	X			2989718018	Idiopathic pulmonary fibrosis	SNOMED-DescriptionID
X	X			2989760019	Idiopathic pulmonary fibrosis	SNOMED-DescriptionID
X	X			700250006	Idiopathic pulmonary fibrosis	SNOMED
X	X			236302005	Idiopathic pulmonary fibrosis, acute fatal form	SNOMED
X	X			hhq..	NINTEDANIB	Read
X	X			1.25444E+16	NINTEDANIB 100mg capsules	SNOMED
X	X			hhq3.	NINTEDANIB 100mg capsules	Read
X	X			1.25445E+16	NINTEDANIB 150mg capsules	SNOMED
X	X			hhq4.	NINTEDANIB 150mg capsules	Read
X	X			715613009	Nintedanib-containing product	SNOMED
X	X			777222000	Pirfenidone	SNOMED
X	X			h8M..	PIRFENIDONE	Read
X	X			1.20021E+16	PIRFENIDONE 267mg capsules	SNOMED
X	X			h8M2.	PIRFENIDONE 267mg capsules	Read
X	X			776919004	Product containing only nintedanib (medicinal product)	SNOMED
X	X			H5633	Usual interstitial pneumonitis	Read
X		X		196144009	(Emphysema [interstitial]/[mediastinal])/(pneumomediastinum)	SNOMED
X		X		45157009	Diffuse idiopathic pulmonary fibrosis	SNOMED
X		X		196125002	Diffuse interstitial pulmonary fibrosis	SNOMED
X		X		H5631	Diffuse pulmonary fibrosis	Read
X		X		2475607017	DIPF – Diffuse interstitial pulmonary fibrosis	SNOMED-DescriptionID
X		X		XE0Ya	Post-inflammatory pulmonary fibrosis	Read
X		X		266368002	Postinflammatory pulmonary fibrosis	SNOMED
X		X		H55..	Postinflammatory pulmonary fibrosis	Read
X			X	36599006	Chronic fibrosis of lung	SNOMED
X			X	51615001	Fibrosis of lung	SNOMED
X			X	85957013	Fibrosis of lung NOS	SNOMED-DescriptionID
X			X	85960018	Fibrosis of lung NOS	SNOMED-DescriptionID
X			X	233703007	ILD – Interstitial lung disease	SNOMED
X			X	350186017	Interstitial lung disease	SNOMED-DescriptionID
X			X	64667001	Interstitial lung disease	SNOMED
X			X	X102V	Interstitial lung disease	Read
X			X	H58y3	Interstitial lung disease NEC	Read
X			X	233726000	Localised pulmonary fibrosis	SNOMED
X			X	X1032	Localised pulmonary fibrosis	Read
X			X	350224012	Localized pulmonary fibrosis (disorder)	SNOMED-DescriptionID
X			X	162974009	O/E – fibrosis of lung	SNOMED
X			X	23E5.	O/E – fibrosis of lung present	Read

X	X	254002014	On examination – fibrosis of lung present (context-dependent category)	SNOMED-DescriptionID
X	X	708030004	Pulmonary emphysema co-occurrent with fibrosis of lung	SNOMED
X	X	155613001	Pulmonary fibrosis	SNOMED
X	X	H5632	Pulmonary fibrosis	Read
X	X	X102u	Pulmonary fibrosis	Read

Supplementary Material E2

Supplementary Material E2: Read, SNOMED, ICD10, and LOINC codes used to define CTDs

Disease category	Code ID	Code term
Reumatoid arthritis		
		name
	1.01007E+15	Rheumatoid arthritis particle agglutination test (observable entity)
	1.02927E+15	Rheumatoid arthritis latex test
	1.04828E+15	RAID (Rheumatoid Arthritis Impact of Disease) questionnaire
	1.04829E+15	Rheumatoid Arthritis Impact of Disease questionnaire
	1.0483E+15	Assessment using Rheumatoid Arthritis Impact of Disease questionnaire
	1.04831E+15	Rheumatoid Arthritis Impact of Disease questionnaire score (observable entity)
	1.05951E+14	Rheumatoid arthritis particle agglutination test
	1.06699E+15	Rheumatoid arthritis monitoring invitation by short message service text messaging
	1.067E+15	Rheumatoid arthritis monitoring SMS (short message service) text message first invitation
	1.06701E+15	Rheumatoid arthritis monitoring short message service text message second invitation
	1.06702E+15	Rheumatoid arthritis monitoring short message service text message third invitation
	10713006	Fibrosing alveolitis associated with rheumatoid arthritis
	1.0736E+15	Seropositive rheumatoid arthritis of joint of right foot
	1.07361E+15	Seropositive rheumatoid arthritis of joint of left foot
	1.0737E+15	Rheumatoid arthritis of left foot
	1.07371E+15	Rheumatoid arthritis of left hand
	1.07372E+15	Rheumatoid arthritis of left hip
	1.07373E+15	Rheumatoid arthritis of left knee
	1.07374E+15	Rheumatoid arthritis of left shoulder
	1.07375E+15	Rheumatoid arthritis of left wrist
	1.07378E+15	Rheumatoid arthritis of right foot
	1.07379E+15	Rheumatoid arthritis of right hand
	1.0738E+15	Rheumatoid arthritis of right hip
	1.07381E+15	Rheumatoid arthritis of right knee
	1.07382E+15	Rheumatoid arthritis of right shoulder
	1.07383E+15	Rheumatoid arthritis of right wrist
	1.07384E+15	Deformity of joint of right wrist region due to rheumatoid arthritis
	1.07385E+15	Deformity of joint of left wrist region due to rheumatoid arthritis
	1.07386E+15	Deformity of right hand co-occurrent and due to rheumatoid arthritis
	1.07387E+15	Deformity of left hand co-occurrent and due to rheumatoid arthritis
	1.07388E+15	Deformity of right foot co-occurrent and due to rheumatoid arthritis
	1.07389E+15	Deformity of left foot co-occurrent and due to rheumatoid arthritis
	1.08217E+15	Salford Rheumatoid Arthritis Foot Evaluation Instrument (assessment scale)
	1.08218E+15	Assessment using Salford Rheumatoid Arthritis Foot Evaluation Instrument
	1.08219E+15	SAFE (Salford Rheumatoid Arthritis Foot Evaluation) Instrument Part A: Fixed Scale – Impairment total score
	1.0822E+15	Salford Rheumatoid Arthritis Foot Evaluation Instrument Part A: Fixed Scale – Disability total score (observable entity)
	1.08221E+15	SAFE (Salford Rheumatoid Arthritis Foot Evaluation) Instrument Part A: Fixed Scale – Footwear total score
	1.08222E+15	Salford Rheumatoid Arthritis Foot Evaluation Instrument Part B: Patient Preference Scale – Foot and Ankle Symptoms domain total score (observable entity)
	1.08223E+15	SAFE (Salford Rheumatoid Arthritis Foot Evaluation) Instrument Part B: Patient Preference Scale – External factors that affect foot symptoms domain total score
	1.08224E+15	SAFE (Salford Rheumatoid Arthritis Foot Evaluation) Instrument Part B: Patient Preference Scale – Impact on everyday functioning domain total score
	1.08225E+15	SAFE (Salford Rheumatoid Arthritis Foot Evaluation) Instrument Part B: Patient Preference Scale – Effect on family life domain total score
	1.08226E+15	SAFE (Salford Rheumatoid Arthritis Foot Evaluation) Instrument Part B: Patient Preference Scale – Experiences with footwear domain total score
	1.08227E+15	SAFE (Salford Rheumatoid Arthritis Foot Evaluation) Instrument Part B: Patient Preference Scale – Personal feelings domain total score
	1.08228E+15	SAFE (Salford Rheumatoid Arthritis Foot Evaluation) Instrument Part B: Patient Preference Scale – Impact on social life domain total score
	1.08229E+15	SAFE (Salford Rheumatoid Arthritis Foot Evaluation) Instrument Part B: Patient Preference Scale – Personal adaptation domain total score

1.0823E+15	SAFE (Salford Rheumatoid Arthritis Foot Evaluation) Instrument Part B: Patient Preference Scale – Visual impact domain total score
1.08231E+15	Salford Rheumatoid Arthritis Foot Evaluation Instrument Part B: Patient Preference Scale – Impact on career domain total score (observable entity)
1.08308E+15	Rheumatoid arthritis monitoring invitation email
1.10552E+16	Seropositive rheumatoid arthritis of multiple joints
111218008	Rheumatoid arthritis with other visceral or systemic involvement
111280008	Caplans syndrome
1149218009	Rheumatoid arthritis with isolated nailfold vasculitis
1149219001	Rheumatoid arthritis with systemic vasculitis
11571-7	Rheumatoid arthritis nuclear Ab [Units/volume] in Serum
116082011	Rheumatoid arthritis
1162303002	Seronegative erosive rheumatoid arthritis
1162313005	Rheumatoid factor and anti-citrullinated protein antibody positive rheumatoid arthritis
1162319009	Rheumatoid factor and anti-citrullinated protein antibody positive erosive rheumatoid arthritis
1162336001	Anti-citrullinated protein antibody positive erosive rheumatoid arthritis
1162362005	Felty syndrome with seronegative erosive rheumatoid arthritis
1162364006	Felty syndrome with seronegative rheumatoid arthritis
1162677006	Rheumatoid arthritis with rheumatoid lung disease
117216004	Rheumatoid arthritis nuclear antibody
122161003	Rheumatoid arthritis nuclear antibody assay
1233202011	RA – Rheumatoid arthritis
1233204012	RhA – Rheumatoid arthritis
129563009	Rheumatoid arthritis with osteoperiostitis
138835002	H/O: rheumatoid arthritis
143243002	Rheumatoid factor positive (finding)
143245009	Rheumatoid arthritis latex test
1.43441E+14	Rheumatoid arthritis in remission
1489971016	Rheumatoid arthritis – ankle and/or foot
14G1.	H/O: rheumatoid arthritis
155621007	Rheumatoid lung (disorder)
156471009	Rheumatoid arthritis
156472002	Rheumatoid arthritis – multiple joint
156473007	Rheumatoid arthritis of shoulder
156474001	Rheumatoid arthritis of elbow
156475000	Rheumatoid arthritis of wrist
156476004	Rheumatoid arthritis – hand joint
156477008	Rheumatoid arthritis of hip
156478003	Rheumatoid arthritis of knee
156479006	Rheumatoid arthritis – ankle/foot (disorder)
156480009	Rheumatoid arthritis – other joint
156481008	Rheumatoid arthritis NOS
156483006	Juvenile rheumatoid a.
1.56863E+16	Rheumatoid arthritis of bilateral feet
1.56872E+16	Rheumatoid arthritis of bilateral knees
1.56873E+16	Rheumatoid arthritis of bilateral hands
1.56911E+16	Seronegative rheumatoid arthritis of joint of bilateral feet
1.56911E+16	Seronegative rheumatoid arthritis of joint of both elbows
1.56912E+16	Bilateral seronegative rheumatoid arthritis of knees
1.56913E+16	Seronegative rheumatoid arthritis of both wrists
1.56918E+16	Seropositive rheumatoid arthritis of joint of both shoulder regions
1.56919E+16	Seropositive rheumatoid arthritis of bilateral knees
1.56919E+16	Seropositive rheumatoid arthritis of bilateral hip joints
1.5692E+16	Bilateral seropositive rheumatoid arthritis of ankle joints
1.5692E+16	Seropositive rheumatoid arthritis of joint of bilateral elbows
1.5692E+16	Seropositive rheumatoid arthritis of joint of bilateral wrist regions
1.57444E+16	Bilateral deformity of hands due to rheumatoid arthritis

1.57445E+16	Bilateral deformity of wrists due to rheumatoid arthritis
1.5745E+16	Deformity of both feet due to rheumatoid arthritis
1.60501E+16	Seropositive rheumatoid arthritis of bilateral feet
161567008	H/O: rheumatoid arthritis
165839004	Rheumatoid factor positive (finding)
165841003	Rheumatoid arthritis latex test
1.66067E+16	Seronegative rheumatoid arthritis in remission
1.66067E+16	Seropositive rheumatoid arthritis in remission
16840014	Rheumatoid arthritis of spine
17532-3	Rheumatoid arthritis nuclear Ab [Presence] in Serum
17533-1	Rheumatoid arthritis nuclear Ab [Presence] in Serum by Immunofluorescence
1.80179E+15	Exception reporting – rheumatoid arthritis quality indicators
1.80183E+15	Excepted from rheumatoid arthritis quality indicators – patient unsuitable
1.80187E+15	Excepted from rheumatoid arthritis quality indicators – informed dissent
193180002	Polyneuropathy in rheumatoid arthritis
193250002	Myopathy due to rheumatoid arthritis
195138003	Rheumatoid carditis
196132006	Rheumatoid lung
201764007	Rheumatoid arthritis of cervical spine
201765008	Other rheumatoid arthritis of spine (disorder)
201766009	Rheumatoid arthritis of shoulder
201767000	Rheumatoid arthritis of sternoclavicular joint
201768005	Rheumatoid arthritis of acromioclavicular joint
201769002	Rheumatoid arthritis of elbow
201770001	Rheumatoid arthritis of distal radioulnar joint
201771002	Rheumatoid arthritis of wrist
201772009	Rheumatoid arthritis of metacarpophalangeal joint
201773004	Rheumatoid arthritis of proximal interphalangeal joint of finger
201774005	Rheumatoid arthritis of distal interphalangeal joint of finger
201775006	Rheumatoid arthritis of hip
201776007	Rheumatoid arthritis of sacroiliac joint
201777003	Rheumatoid arthritis of knee
201778008	Rheumatoid arthritis of tibiofibular joint
201779000	Rheumatoid arthritis of ankle (disorder)
201780002	Rheumatoid arthritis of subtalar joint
201781003	Rheumatoid arthritis of talonavicular joint
201782005	Rheumatoid arthritis of other tarsal joint
201783000	Rheumatoid arthritis of first metatarsophalangeal joint
201784006	Rheumatoid arthritis of lesser metatarsophalangeal joint
201785007	Rheumatoid arthritis of interphalangeal joint of toe
201786008	Rheumatoid vasculitis (disorder)
201787004	Seronegative rheumatoid arthritis
201788009	Rheumatoid bursitis
201789001	Rheumatoid nodule (disorder)
201790005	Rheumatoid arthritis – multiple joint
201791009	Flare of rheumatoid arthritis
201793007	Rheumatic carditis (?AND/OR [Rheumatoid carditis])
201794001	Rheumatoid lung (disorder)
201796004	Systemic onset juvenile rheumatoid arthritis
201797008	Juvenile rheumatoid arthropathy unspecified
201798003	Acute polyarticular juvenile rheumatoid arthritis
201799006	Monarticular juvenile rheumatoid arthritis
201800005	Juvenile rheumatoid arthritis NOS (disorder)
201808003	Juvenile rheumatoid arthritis
201810001	Seropositive errosive rheumatoid arthritis

201811002	[X]Seropositive rheumatoid arthritis, unspecified (disorder)
201813004	Fibrosing alveolitis associated with rheumatoid arthritis
203729008	[X]Rheumatoid arthritis with involvement of other organs or systems
203730003	[X]Other seropositive rheumatoid arthritis
203732006	[X]Other specified rheumatoid arthritis
203746006	[X]Seropositive rheumatoid arthritis, unspecified
21430002	Dilated cardiomyopathy due to rheumatoid arthritis
2159844013	Rheumatoid arthritis particle agglutination test
2.19679E+15	Rheumatoid arthritis annual review
2.2755E+15	Rheumatoid arthritis monitoring invitation
2.27567E+15	Rheumatoid arthritis monitoring invitation first letter
2.27571E+15	Rheumatoid arthritis monitoring invitation second letter
2.27575E+15	Rheumatoid arthritis monitoring invitation third letter
2.27579E+15	Rheumatoid arthritis monitoring verbal invitation
2.27583E+15	Rheumatoid arthritis monitoring telephone invitation
2.31041E+14	Delivery of rehabilitation for rheumatoid arthritis (regime/therapy)
23685000	Rheumatic carditis
239791005	Seropositive rheumatoid arthritis
239792003	Seronegative rheumatoid arthritis
239794002	Fibrosing alveolitis associated with rheumatoid arthritis
239795001	Rheumatoid arthritis with multisystem involvement
239801005	JRA – Juvenile rheumatoid arthritis
239943002	Rheumatoid arthritis with 8ubule8ing8 vasculitis
251794010	H/O: rheumatoid arthritis
257898011	Rheumatoid arthritis latex test
2.58232E+15	Rheumatoid arthritis particle agglutination test
2.60263E+15	Rheumatoid arthritis latex test
2.64082E+15	Rheumatoid Arthritis Impact of Disease questionnaire
267887009	Rheumatoid arthritis and other inflammatory polyarthropathies
2.68001E+15	Rheumatoid arthritis monitoring invitation by SMS (short message service) text messaging
2.68004E+15	Rheumatoid arthritis monitoring SMS (short message service) text message first invitation
2.68006E+15	Rheumatoid arthritis monitoring SMS (short message service) text message second invitation
2.68008E+15	Rheumatoid arthritis monitoring SMS (short message service) text message third invitation
268052008	Juvenile rheumatoid a.
2692387019	Rheumatoid arthritis of multiple joints
2696026017	Rheumatoid arthritis of foot
2.71409E+15	Rheumatoid arthritis monitoring invitation email
2.72451E+14	Delivery of rehabilitation for rheumatoid arthritis
275902004	Rheumatoid arthrit. Monitoring
2838687017	DAS (disease activity score) in rheumatoid arthritis
2839290010	DAS (disease activity score) 28 joint in rheumatoid arthritis
2839291014	Disease activity score 28 joint in rheumatoid arthritis
2842168015	Disease activity score in rheumatoid arthritis
2842339011	Progressive systemic sclerosis
287006005	Rheumatoid arthritis of multiple joints
287007001	Rheumatoid arthritis of hand joint
287008006	Rheumatoid arthritis – ankle and/or foot
287009003	Rheumatoid arthritis – other joint
287010008	Rheumatoid arthritis NOS
28880005	Rheumatoid arthritis with carditis
2.93281E+14	Disease activity score in rheumatoid arthritis
297544012	Polyneuropathy in rheumatoid arthritis
297641010	Myopathy due to rheumatoid arthritis
308143008	Seropositive erosive rheumatoid arthritis
309787016	Rheumatoid arthritis of cervical spine

309789018	Rheumatoid arthritis of shoulder
309790010	Rheumatoid arthritis of sternoclavicular joint
309791014	Rheumatoid arthritis of acromioclavicular joint
309792019	Rheumatoid arthritis of elbow
309793012	Rheumatoid arthritis of distal radioulnar joint
309794018	Rheumatoid arthritis of wrist
309795017	Rheumatoid arthritis of metacarpophalangeal joint
309796016	Rheumatoid arthritis of proximal interphalangeal joint of finger
309797013	Rheumatoid arthritis of distal interphalangeal joint of finger
309798015	Rheumatoid arthritis of hip
309799011	Rheumatoid arthritis of sacroiliac joint
309800010	Rheumatoid arthritis of knee
309801014	Rheumatoid arthritis of tibiofibular joint
309802019	Rheumatoid arthritis of ankle
309803012	Rheumatoid arthritis of subtalar joint
309804018	Rheumatoid arthritis of talonavicular joint
309806016	Rheumatoid arthritis of first metatarsophalangeal joint
309807013	Rheumatoid arthritis of 1st metatarsophalangeal joint
309808015	Rheumatoid arthritis of lesser metatarsophalangeal joint
309809011	Rheumatoid arthritis of interphalangeal joint of toe
309816012	Flare of rheumatoid arthritis
311496011	Fibrosing alveolitis associated with rheumatoid arthritis
3.18841E+14	Seropositive rheumatoid arthritis of left ankle
3.18851E+14	Seropositive rheumatoid arthritis of joint of left elbow
3.18861E+14	Seropositive rheumatoid arthritis of left hand
3.18891E+14	Seropositive rheumatoid arthritis of left shoulder
3.18901E+14	Seropositive rheumatoid arthritis of left wrist
3.18911E+14	Seropositive rheumatoid arthritis of joint of right ankle
3.18921E+14	Right elbow seropositive rheumatoid arthritis
3.18931E+14	Seropositive rheumatoid arthritis of right hand
3.18961E+14	Seropositive rheumatoid arthritis of joint of right shoulder region
3.18971E+14	Seropositive rheumatoid arthritis of joint of right wrist region
3.19011E+14	Seronegative rheumatoid arthritis of joint of left ankle
3.19041E+14	Seronegative rheumatoid arthritis of joint of left hip
3.19051E+14	Seronegative rheumatoid arthritis of left knee joint
3.19061E+14	Seronegative rheumatoid arthritis of joint of left shoulder region
3.19071E+14	Seronegative rheumatoid arthritis of joint of left wrist region
3.19081E+14	Seronegative rheumatoid arthritis of multiple joints
3.19091E+14	Seronegative rheumatoid arthritis of joint of right ankle
3.19121E+14	Seronegative rheumatoid arthritis of joint of right hip
3.19131E+14	Seronegative rheumatoid arthritis of right knee joint
3.19141E+14	Seronegative rheumatoid arthritis of joint of right shoulder region
3.19151E+14	Right wrist region seronegative rheumatoid arthritis
3.19841E+14	Rheumatoid lung disease with rheumatoid arthritis
3470F	Rheumatoid arthritis (RA) disease activity, low (RA)
3471F	Rheumatoid arthritis (RA) disease activity, moderate (RA)
3472F	Rheumatoid arthritis (RA) disease activity, high (RA)
3475F	Disease prognosis for rheumatoid arthritis assessed, poor prognosis documented (RA)
3476F	Disease prognosis for rheumatoid arthritis assessed, good prognosis documented (RA)
3517964017	Seropositive erosive rheumatoid arthritis
359291017	Seropositive rheumatoid arthritis
359292012	Seronegative rheumatoid arthritis
359293019	Rheumatoid arthritis with organ / system involvement
359295014	Rheumatoid arthritis with multisystem involvement
3.71261E+14	Delivery of rehabilitation for rheumatoid arthritis

38877003	Rheumatoid arthritis with aortitis
38DZ.	Disease activity score in rheumatoid arthritis
38DZ0	Disease activity score 28 joint in rheumatoid arthritis
38Vs.	Rheumatoid Arthritis Impact of Disease questionnaire
398640008	Rheumatoid pneumoconiosis (disorder)
398726004	Rheumatoid lung disease (disorder)
399112009	Seronegative arthritis (disorder)
399923009	Rheumatoid arthritis with arteritis
400054000	Rheumatoid vasculitis (disorder)
402431009	Cutaneous atrophy due to rheumatoid arthritis
402432002	Rheumatoid arthritis with neutrophilic dermatitis
402433007	Rheumatoid arthritis with nailfold/finger-pulp infarcts
408276001	Rheumatoid arthritis particle agglutination test
4.08751E+14	[X]Other seropositive rheumatoid arthritis (disorder)
410502007	Juvenile rheumatoid arthritis
410795001	Juvenile rheumatoid arthritis (disorder)
411871019	Rheumatoid arthritis monitoring
4.14821E+14	[X]Seropositive rheumatoid arthritis, unspecified (disorder)
416666007	Anterior uveitis in juvenile rheumatoid arthritis
4.25091E+14	[X]Rheumatoid arthritis with involvement of other organs or systems
4.25481E+14	[X]Other specified rheumatoid arthritis (disorder)
426510015	Rheumatoid arthritis – multiple joint
426511016	Rheumatoid arthritis – hand joint
426512011	Rheumatoid arthritis – ankle/foot
427770001	Rheumatoid arthritis of temporomandibular joint
429192004	Rheumatoid arthritis of foot
431707004	Deformity of wrist due to rheumatoid arthritis
433228003	Deformity of foot due to rheumatoid arthritis
4385018	Chronic polyarticular juvenile rheumatoid arthritis
43b9.	Rheumatoid arthritis particle agglutination test
43F1.	Rheumatoid factor positive
43F3.	Rheumatoid arthritis latex test
441870009	Disease activity score 28 joint in rheumatoid arthritis
442194005	Swollen joint count for rheumatoid arthritis assessment (observable entity)
442277000	Rheumatoid arthritis disease activity score using C-reactive protein
442521001	Tender joint count for rheumatoid arthritis assessment
443349002	DAS (disease activity score) in rheumatoid arthritis
443539004	DAS (disease activity score) in rheumatoid arthritis using C-reactive protein
443728000	Disease activity score 28 joint in rheumatoid arthritis
444009006	Rheumatoid arthritis assessment
444060007	Disease activity score in rheumatoid arthritis using C-reactive protein
445985006	Assessment using rheumatoid arthritis work instability scale
446043004	RA-WIS (rheumatoid arthritis work instability scale) score
451461014	Seropositive errosive rheumatoid arthritis
4.71971E+14	[X]Seropositive rheumatoid arthritis, unspecified (disorder)
4.93221E+14	Rheumatoid arthritis assessment
4.93231E+14	Assessment of rheumatoid arthritis using disease activity score – original (regime/therapy)
4.93241E+14	Assessment of rheumatoid arthritis using disease activity score 28 joint count
4.93251E+14	Assessment of rheumatoid arthritis using disease activity score using C-reactive protein
4.93291E+14	Disease activity score in rheumatoid arthritis using C-reactive protein (assessment scale)
4.93301E+14	Disease activity score 28 joint in rheumatoid arthritis (assessment scale)
4.93311E+14	Disease activity score in rheumatoid arthritis – original
4.94441E+14	Rheumatoid arthritis work instability scale
5.07081E+14	Disease activity score 28 joint in rheumatoid arthritis
5.07091E+14	Disease activity score in rheumatoid arthritis using C-reactive protein

50782009	Rheumatoid nodule (morphologic abnormality)
547307019	Rheumatoid arthritis latex test
54867000	Rheumatoid arthritis with fibrosing alveolitis
5.67021E+14	Rheumatoid arthritis and other inflammatory polyarthropathies
56977-2	Rheumatoid arthritis nuclear Ab [Titer] in Serum by Immunofluorescence
57160007	Rheumatoid arthritis, leukopenia and splenadenomegaly
59165007	Rheumatoid arthritis with scleritis
6.46871E+14	Rheumatoid arthritis – other joint
6.46881E+14	Rheumatoid arthritis NOS
6.56651E+14	Rheumatoid arthritis of other tarsal joint
6.56671E+14	Juvenile rheumatoid arthritis NOS
66HB0	Rheumatoid arthritis annual review
6.89621E+14	Juvenile rheumatoid arthropathy unspecified
69896004	Rheumatoid arthritis
7.06431E+14	Disease activity score in rheumatoid arthritis
714	Rheumatoid arthritis and other inflammatory polyarthropathies
714	Rheumatoid arthritis
714.2	Other rheumatoid arthritis with visceral or systemic involvement
7.26751E+14	Assessment using rheumatoid arthritis work instability scale
7.29921E+14	Rheumatoid arthritis work instability scale score (observable entity)
735599007	Rheumatoid arthritis with erosion of joint
735600005	Rheumatoid arthritis without erosion
74391003	Pauciarticular juvenile rheumatoid arthritis (disorder)
75633-8	Rheumatoid arthritis disease activity score Calculated by VectraDA
75634-6	Rheumatoid arthritis disease activity score level Qualitative by VectraDA
75635-3	Rheumatoid arthritis disease activity panel – Serum or Plasma by VectraDA
75822003	Acute juvenile rheumatoid arthritis
76064-5	Rheumatoid arthritis disease activity score confidence interval by VectraDA
7607008	Rheumatoid arthritis with pericarditis
764528008	Assessment using Salford Rheumatoid Arthritis Foot Evaluation Instrument
764529000	Salford Rheumatoid Arthritis Foot Evaluation Instrument
764894008	SAFE (Salford Rheumatoid Arthritis Foot Evaluation) Instrument Part A: Fixed Scale – Footwear total score
764895009	Salford Rheumatoid Arthritis Foot Evaluation Instrument Part A: Fixed Scale – Disability total score
764896005	SAFE (Salford Rheumatoid Arthritis Foot Evaluation) Instrument Part B: Patient Preference Scale – Personal feelings domain total score
764897001	Salford Rheumatoid Arthritis Foot Evaluation Instrument Part B: Patient Preference Scale – Impact on career domain total score
764898006	Salford Rheumatoid Arthritis Foot Evaluation Instrument Part B: Patient Preference Scale – Effect on family life domain total score
764899003	SAFE (Salford Rheumatoid Arthritis Foot Evaluation) Instrument Part B: Patient Preference Scale – External factors that affect foot symptoms domain total score
764900008	SAFE (Salford Rheumatoid Arthritis Foot Evaluation) Instrument Part B: Patient Preference Scale – Visual impact domain total score
764901007	Salford Rheumatoid Arthritis Foot Evaluation Instrument Part A: Fixed Scale – Impairment total score
764902000	SAFE (Salford Rheumatoid Arthritis Foot Evaluation) Instrument Part B: Patient Preference Scale – Impact on everyday functioning domain total score
764903005	Salford Rheumatoid Arthritis Foot Evaluation Instrument Part B: Patient Preference Scale – Experiences with footwear domain total score (observable entity)
764904004	Salford Rheumatoid Arthritis Foot Evaluation Instrument Part B: Patient Preference Scale – Impact on social life domain total score (observable entity)
764905003	Salford Rheumatoid Arthritis Foot Evaluation Instrument Part B: Patient Preference Scale – Personal adaptation domain total score (observable entity)
764906002	Salford Rheumatoid Arthritis Foot Evaluation Instrument Part B: Patient Preference Scale – Foot and Ankle Symptoms domain total score (observable entity)
76521009	Systemic lupus erythematosus glomerulonephritis syndrome, World Health Organization class III
77522006	Rheumatoid arthritis with episcleritis
781206002	Rheumatoid arthritis of joint of spine
7P203	Delivery of rehabilitation for rheumatoid arthritis
80172006	Rheumatoid arteritis
8.05941E+14	Exception reporting – rheumatoid arthritis quality indicators
8.05951E+14	Exception reporting: rheumatoid arthritis quality indicators
8.05961E+14	Excepted from rheumatoid arthritis quality indicators – patient unsuitable
8.05971E+14	Excepted from rheumatoid arthritis quality indicators: patient unsuitable
8.05981E+14	Excepted from rheumatoid arthritis quality indicators – informed dissent
8.05991E+14	Excepted from rheumatoid arthritis quality indicators: informed dissent

8.06451E+14	Disease activity score 28 joint in rheumatoid arthritis
8.06461E+14	Disease activity score 28 joint in rheumatoid arthritis
836472001	Spindling of fingers of both hands due to rheumatoid arthritis
8.47261E+14	Rheumatoid arthritis annual review (regime/therapy)
8.56181E+14	Rheumatoid arthritis annual review
86219005	Uveitis-rheumatoid arthritis syndrome
8.82321E+14	Rheumatoid arthritis monitoring invitation (procedure)
8.82331E+14	Rheumatoid arthritis monitoring invitation
8.82401E+14	Rheumatoid arthritis monitoring invitation first letter (procedure)
8.82411E+14	Rheumatoid arthritis monitoring invitation first letter
8.82421E+14	Rheumatoid arthritis monitoring invitation second letter (procedure)
8.82431E+14	Rheumatoid arthritis monitoring invitation second letter
8.82441E+14	Rheumatoid arthritis monitoring invitation third letter
8.82451E+14	Rheumatoid arthritis monitoring invitation third lettter
8.82461E+14	Rheumatoid arthritis monitoring verbal invitation (procedure)
8.82471E+14	Rheumatoid arthritis monitoring verbal invitation
8.82481E+14	Rheumatoid arthritis monitoring telephone invitation
88982-4	Rheumatoid arthritis disease activity level [CDAI]
8.98501E+14	[X]Other seropositive rheumatoid arthritis
8.98511E+14	[X]Other specified rheumatoid arthritis
90094-4	Rheumatoid arthritis disease severity level [RAPID3]
9.01821E+14	[X]Seropositive rheumatoid arthritis, unspecified
9.22451E+14	[X]Rheumatoid arthritis with involvement of other organs or systems
9631008	Rheumatoid arthritis of spine
9.6531E+13	Deformity of hand due to rheumatoid arthritis
9.6941E+13	Rheumatoid arthritis particle agglutination test
9839-2	Rheumatoid arthritis nuclear Ab [Presence] in Serum by Immune diffusion (ID)
9hR..	Exception reporting: rheumatoid arthritis quality indicators
9hR0.	Except rheumatoid arthritis quality indicator: pt unsuitable
9hR1.	Except rheumatoid arthritis qual indicator: informed dissent
9mM..	Rheumatoid arthritis monitoring invitation
9mM0.	Rheumatoid arthritis monitoring invitation first letter
9mM1.	Rheumatoid arthritis monitoring invitation second letter
9mM2.	Rheumatoid arthritis monitoring invitation third letter
9mM3.	Rheumatoid arthritis monitoring verbal invitation
9mM4.	Rheumatoid arthritis monitoring telephone invitation
F3712	Polyneuropathy in rheumatoid arthritis
F3964	Myopathy due to rheumatoid arthritis
G5yA.	Rheumatoid carditis
H570.	Rheumatoid lung
LA14307-5	Rheumatoid arthritis, doctor diagnosis
LA15161-5	Rheumatoid Arthritis
LA17779-2	Rheumatoid arthritis – rheumatism
LA27402-9	Arthritis- rheumatoid arthritis
LG23002-5	Rheumatoid arthritis nuclear Ab|Titr|Pt|ANYBldSerPl
LG5480-1	Rheumatoid arthritis nuclear Ab|PrThr|Pt|ANYBldSerPl
LP18024-7	Rheumatoid arthritis nuclear
LP185753-3	Rheumatoid arthritis disease activity score
LP185754-1	Rheumatoid arthritis disease activity score level
LP185755-8	Rheumatoid arthritis disease activity score confidence interval
LP185759-0	Rheumatoid arthritis disease activity panel
LP187192-2	Rheumatoid arthritis disease activity
LP187616-0	Rheumatoid arthritis disease activity score | Bld-Ser-Plas
LP187617-8	Rheumatoid arthritis disease activity score level | Bld-Ser-Plas
LP187714-3	Rheumatoid arthritis disease activity score confidence interval | Bld-Ser-Plas

LP256137-3	Rheumatoid arthritis nuclear | Bld-Ser-Plas
LP266756-8	Rheumatoid arthritis disease activity level
LP268475-3	Rheumatoid arthritis disease severity level
LP385476-9	Rheumatoid arthritis disease activity score | Serum or Plasma | Chemistry – non-challenge
LP385477-7	Rheumatoid arthritis disease activity score level | Serum or Plasma | Chemistry – non-challenge
LP385478-5	Rheumatoid arthritis disease activity score confidence interval | Serum or Plasma | Chemistry – non-challenge
LP387049-2	Rheumatoid arthritis disease activity panel | Serum or Plasma | Chemistry Panels
LP39559-7	Rheumatoid arthritis nuclear Ab
LP403915-4	Rheumatoid arthritis nuclear Ab | Serum | Serology – non-micro
LP41610-4	Rheumatoid Arthritis Nuclear Ab | Bld-Ser-Plas
M05	Seropositive rheumatoid arthritis
M05.1	Rheumatoid lung disease with rheumatoid arthritis
M05.10	Rheumatoid lung disease with rheumatoid arthritis of unspecified site
M05.11	Rheumatoid lung disease with rheumatoid arthritis of shoulder
M05.111	Rheumatoid lung disease with rheumatoid arthritis of right shoulder
M05.112	Rheumatoid lung disease with rheumatoid arthritis of left shoulder
M05.119	Rheumatoid lung disease with rheumatoid arthritis of unspecified shoulder
M05.12	Rheumatoid lung disease with rheumatoid arthritis of elbow
M05.121	Rheumatoid lung disease with rheumatoid arthritis of right elbow
M05.122	Rheumatoid lung disease with rheumatoid arthritis of left elbow
M05.129	Rheumatoid lung disease with rheumatoid arthritis of unspecified elbow
M05.13	Rheumatoid lung disease with rheumatoid arthritis of wrist
M05.131	Rheumatoid lung disease with rheumatoid arthritis of right wrist
M05.132	Rheumatoid lung disease with rheumatoid arthritis of left wrist
M05.139	Rheumatoid lung disease with rheumatoid arthritis of unspecified wrist
M05.14	Rheumatoid lung disease with rheumatoid arthritis of hand
M05.141	Rheumatoid lung disease with rheumatoid arthritis of right hand
M05.142	Rheumatoid lung disease with rheumatoid arthritis of left hand
M05.149	Rheumatoid lung disease with rheumatoid arthritis of unspecified hand
M05.15	Rheumatoid lung disease with rheumatoid arthritis of hip
M05.151	Rheumatoid lung disease with rheumatoid arthritis of right hip
M05.152	Rheumatoid lung disease with rheumatoid arthritis of left hip
M05.159	Rheumatoid lung disease with rheumatoid arthritis of unspecified hip
M05.16	Rheumatoid lung disease with rheumatoid arthritis of knee
M05.161	Rheumatoid lung disease with rheumatoid arthritis of right knee
M05.162	Rheumatoid lung disease with rheumatoid arthritis of left knee
M05.169	Rheumatoid lung disease with rheumatoid arthritis of unspecified knee
M05.17	Rheumatoid lung disease with rheumatoid arthritis of ankle and foot
M05.171	Rheumatoid lung disease with rheumatoid arthritis of right ankle and foot
M05.172	Rheumatoid lung disease with rheumatoid arthritis of left ankle and foot
M05.179	Rheumatoid lung disease with rheumatoid arthritis of unspecified ankle and foot
M05.19	Rheumatoid lung disease with rheumatoid arthritis of multiple sites
M05.2	Rheumatoid vasculitis with rheumatoid arthritis
M05.20	Rheumatoid vasculitis with rheumatoid arthritis of unspecified site
M05.21	Rheumatoid vasculitis with rheumatoid arthritis of shoulder
M05.211	Rheumatoid vasculitis with rheumatoid arthritis of right shoulder
M05.212	Rheumatoid vasculitis with rheumatoid arthritis of left shoulder
M05.219	Rheumatoid vasculitis with rheumatoid arthritis of unspecified shoulder
M05.22	Rheumatoid vasculitis with rheumatoid arthritis of elbow
M05.221	Rheumatoid vasculitis with rheumatoid arthritis of right elbow
M05.222	Rheumatoid vasculitis with rheumatoid arthritis of left elbow
M05.229	Rheumatoid vasculitis with rheumatoid arthritis of unspecified elbow
M05.23	Rheumatoid vasculitis with rheumatoid arthritis of wrist
M05.231	Rheumatoid vasculitis with rheumatoid arthritis of right wrist
M05.232	Rheumatoid vasculitis with rheumatoid arthritis of left wrist

M05.239	Rheumatoid vasculitis with rheumatoid arthritis of unspecified wrist
M05.24	Rheumatoid vasculitis with rheumatoid arthritis of hand
M05.241	Rheumatoid vasculitis with rheumatoid arthritis of right hand
M05.242	Rheumatoid vasculitis with rheumatoid arthritis of left hand
M05.249	Rheumatoid vasculitis with rheumatoid arthritis of unspecified hand
M05.25	Rheumatoid vasculitis with rheumatoid arthritis of hip
M05.251	Rheumatoid vasculitis with rheumatoid arthritis of right hip
M05.252	Rheumatoid vasculitis with rheumatoid arthritis of left hip
M05.259	Rheumatoid vasculitis with rheumatoid arthritis of unspecified hip
M05.26	Rheumatoid vasculitis with rheumatoid arthritis of knee
M05.261	Rheumatoid vasculitis with rheumatoid arthritis of right knee
M05.262	Rheumatoid vasculitis with rheumatoid arthritis of left knee
M05.269	Rheumatoid vasculitis with rheumatoid arthritis of unspecified knee
M05.27	Rheumatoid vasculitis with rheumatoid arthritis of ankle and foot
M05.271	Rheumatoid vasculitis with rheumatoid arthritis of right ankle and foot
M05.272	Rheumatoid vasculitis with rheumatoid arthritis of left ankle and foot
M05.279	Rheumatoid vasculitis with rheumatoid arthritis of unspecified ankle and foot
M05.29	Rheumatoid vasculitis with rheumatoid arthritis of multiple sites
M05.3	Rheumatoid arthritis with involvement of other organs and systems
M05.30	Rheumatoid arthritis with involvement of other organs and systems, multiple sites
M05.31	Rheumatoid arthritis with involvement of other organs and systems, shoulder region
M05.311	Rheumatoid heart disease with rheumatoid arthritis of right shoulder
M05.312	Rheumatoid heart disease with rheumatoid arthritis of left shoulder
M05.319	Rheumatoid heart disease with rheumatoid arthritis of unspecified shoulder
M05.32	Rheumatoid arthritis with involvement of other organs and systems, upper arm
M05.321	Rheumatoid heart disease with rheumatoid arthritis of right elbow
M05.322	Rheumatoid heart disease with rheumatoid arthritis of left elbow
M05.329	Rheumatoid heart disease with rheumatoid arthritis of unspecified elbow
M05.33	Rheumatoid arthritis with involvement of other organs and systems, forearm
M05.331	Rheumatoid heart disease with rheumatoid arthritis of right wrist
M05.332	Rheumatoid heart disease with rheumatoid arthritis of left wrist
M05.339	Rheumatoid heart disease with rheumatoid arthritis of unspecified wrist
M05.34	Rheumatoid arthritis with involvement of other organs and systems, hand
M05.341	Rheumatoid heart disease with rheumatoid arthritis of right hand
M05.342	Rheumatoid heart disease with rheumatoid arthritis of left hand
M05.349	Rheumatoid heart disease with rheumatoid arthritis of unspecified hand
M05.35	Rheumatoid arthritis with involvement of other organs and systems, pelvic region and thigh
M05.351	Rheumatoid heart disease with rheumatoid arthritis of right hip
M05.352	Rheumatoid heart disease with rheumatoid arthritis of left hip
M05.359	Rheumatoid heart disease with rheumatoid arthritis of unspecified hip
M05.36	Rheumatoid arthritis with involvement of other organs and systems, lower leg
M05.361	Rheumatoid heart disease with rheumatoid arthritis of right knee
M05.362	Rheumatoid heart disease with rheumatoid arthritis of left knee
M05.369	Rheumatoid heart disease with rheumatoid arthritis of unspecified knee
M05.37	Rheumatoid arthritis with involvement of other organs and systems, ankle and foot
M05.371	Rheumatoid heart disease with rheumatoid arthritis of right ankle and foot
M05.372	Rheumatoid heart disease with rheumatoid arthritis of left ankle and foot
M05.379	Rheumatoid heart disease with rheumatoid arthritis of unspecified ankle and foot
M05.38	Rheumatoid arthritis with involvement of other organs and systems, other
M05.39	Rheumatoid arthritis with involvement of other organs and systems, site unspecified
M05.4	Rheumatoid myopathy with rheumatoid arthritis
M05.40	Rheumatoid myopathy with rheumatoid arthritis of unspecified site
M05.41	Rheumatoid myopathy with rheumatoid arthritis of shoulder
M05.411	Rheumatoid myopathy with rheumatoid arthritis of right shoulder
M05.412	Rheumatoid myopathy with rheumatoid arthritis of left shoulder

M05.419	Rheumatoid myopathy with rheumatoid arthritis of unspecified shoulder
M05.42	Rheumatoid myopathy with rheumatoid arthritis of elbow
M05.421	Rheumatoid myopathy with rheumatoid arthritis of right elbow
M05.422	Rheumatoid myopathy with rheumatoid arthritis of left elbow
M05.429	Rheumatoid myopathy with rheumatoid arthritis of unspecified elbow
M05.43	Rheumatoid myopathy with rheumatoid arthritis of wrist
M05.431	Rheumatoid myopathy with rheumatoid arthritis of right wrist
M05.432	Rheumatoid myopathy with rheumatoid arthritis of left wrist
M05.439	Rheumatoid myopathy with rheumatoid arthritis of unspecified wrist
M05.44	Rheumatoid myopathy with rheumatoid arthritis of hand
M05.441	Rheumatoid myopathy with rheumatoid arthritis of right hand
M05.442	Rheumatoid myopathy with rheumatoid arthritis of left hand
M05.449	Rheumatoid myopathy with rheumatoid arthritis of unspecified hand
M05.45	Rheumatoid myopathy with rheumatoid arthritis of hip
M05.451	Rheumatoid myopathy with rheumatoid arthritis of right hip
M05.452	Rheumatoid myopathy with rheumatoid arthritis of left hip
M05.459	Rheumatoid myopathy with rheumatoid arthritis of unspecified hip
M05.46	Rheumatoid myopathy with rheumatoid arthritis of knee
M05.461	Rheumatoid myopathy with rheumatoid arthritis of right knee
M05.462	Rheumatoid myopathy with rheumatoid arthritis of left knee
M05.469	Rheumatoid myopathy with rheumatoid arthritis of unspecified knee
M05.47	Rheumatoid myopathy with rheumatoid arthritis of ankle and foot
M05.471	Rheumatoid myopathy with rheumatoid arthritis of right ankle and foot
M05.472	Rheumatoid myopathy with rheumatoid arthritis of left ankle and foot
M05.479	Rheumatoid myopathy with rheumatoid arthritis of unspecified ankle and foot
M05.49	Rheumatoid myopathy with rheumatoid arthritis of multiple sites
M05.5	Rheumatoid polyneuropathy with rheumatoid arthritis
M05.50	Rheumatoid polyneuropathy with rheumatoid arthritis of unspecified site
M05.51	Rheumatoid polyneuropathy with rheumatoid arthritis of shoulder
M05.511	Rheumatoid polyneuropathy with rheumatoid arthritis of right shoulder
M05.512	Rheumatoid polyneuropathy with rheumatoid arthritis of left shoulder
M05.519	Rheumatoid polyneuropathy with rheumatoid arthritis of unspecified shoulder
M05.52	Rheumatoid polyneuropathy with rheumatoid arthritis of elbow
M05.521	Rheumatoid polyneuropathy with rheumatoid arthritis of right elbow
M05.522	Rheumatoid polyneuropathy with rheumatoid arthritis of left elbow
M05.529	Rheumatoid polyneuropathy with rheumatoid arthritis of unspecified elbow
M05.53	Rheumatoid polyneuropathy with rheumatoid arthritis of wrist
M05.531	Rheumatoid polyneuropathy with rheumatoid arthritis of right wrist
M05.532	Rheumatoid polyneuropathy with rheumatoid arthritis of left wrist
M05.539	Rheumatoid polyneuropathy with rheumatoid arthritis of unspecified wrist
M05.54	Rheumatoid polyneuropathy with rheumatoid arthritis of hand
M05.541	Rheumatoid polyneuropathy with rheumatoid arthritis of right hand
M05.542	Rheumatoid polyneuropathy with rheumatoid arthritis of left hand
M05.549	Rheumatoid polyneuropathy with rheumatoid arthritis of unspecified hand
M05.55	Rheumatoid polyneuropathy with rheumatoid arthritis of hip
M05.551	Rheumatoid polyneuropathy with rheumatoid arthritis of right hip
M05.552	Rheumatoid polyneuropathy with rheumatoid arthritis of left hip
M05.559	Rheumatoid polyneuropathy with rheumatoid arthritis of unspecified hip
M05.56	Rheumatoid polyneuropathy with rheumatoid arthritis of knee
M05.561	Rheumatoid polyneuropathy with rheumatoid arthritis of right knee
M05.562	Rheumatoid polyneuropathy with rheumatoid arthritis of left knee
M05.569	Rheumatoid polyneuropathy with rheumatoid arthritis of unspecified knee
M05.57	Rheumatoid polyneuropathy with rheumatoid arthritis of ankle and foot
M05.571	Rheumatoid polyneuropathy with rheumatoid arthritis of right ankle and foot
M05.572	Rheumatoid polyneuropathy with rheumatoid arthritis of left ankle and foot

M05.579	Rheumatoid polyneuropathy with rheumatoid arthritis of unspecified ankle and foot
M05.59	Rheumatoid polyneuropathy with rheumatoid arthritis of multiple sites
M05.6	Rheumatoid arthritis with involvement of other organs and systems
M05.60	Rheumatoid arthritis of unspecified site with involvement of other organs and systems
M05.61	Rheumatoid arthritis of shoulder with involvement of other organs and systems
M05.611	Rheumatoid arthritis of right shoulder with involvement of other organs and systems
M05.612	Rheumatoid arthritis of left shoulder with involvement of other organs and systems
M05.619	Rheumatoid arthritis of unspecified shoulder with involvement of other organs and systems
M05.62	Rheumatoid arthritis of elbow with involvement of other organs and systems
M05.621	Rheumatoid arthritis of right elbow with involvement of other organs and systems
M05.622	Rheumatoid arthritis of left elbow with involvement of other organs and systems
M05.629	Rheumatoid arthritis of unspecified elbow with involvement of other organs and systems
M05.63	Rheumatoid arthritis of wrist with involvement of other organs and systems
M05.631	Rheumatoid arthritis of right wrist with involvement of other organs and systems
M05.632	Rheumatoid arthritis of left wrist with involvement of other organs and systems
M05.639	Rheumatoid arthritis of unspecified wrist with involvement of other organs and systems
M05.64	Rheumatoid arthritis of hand with involvement of other organs and systems
M05.641	Rheumatoid arthritis of right hand with involvement of other organs and systems
M05.642	Rheumatoid arthritis of left hand with involvement of other organs and systems
M05.649	Rheumatoid arthritis of unspecified hand with involvement of other organs and systems
M05.65	Rheumatoid arthritis of hip with involvement of other organs and systems
M05.651	Rheumatoid arthritis of right hip with involvement of other organs and systems
M05.652	Rheumatoid arthritis of left hip with involvement of other organs and systems
M05.659	Rheumatoid arthritis of unspecified hip with involvement of other organs and systems
M05.66	Rheumatoid arthritis of knee with involvement of other organs and systems
M05.661	Rheumatoid arthritis of right knee with involvement of other organs and systems
M05.662	Rheumatoid arthritis of left knee with involvement of other organs and systems
M05.669	Rheumatoid arthritis of unspecified knee with involvement of other organs and systems
M05.67	Rheumatoid arthritis of ankle and foot with involvement of other organs and systems
M05.671	Rheumatoid arthritis of right ankle and foot with involvement of other organs and systems
M05.672	Rheumatoid arthritis of left ankle and foot with involvement of other organs and systems
M05.679	Rheumatoid arthritis of unspecified ankle and foot with involvement of other organs and systems
M05.69	Rheumatoid arthritis of multiple sites with involvement of other organs and systems
M05.7	Rheumatoid arthritis with rheumatoid factor without organ or systems involvement
M05.70	Rheumatoid arthritis with rheumatoid factor of unspecified site without organ or systems involvement
M05.71	Rheumatoid arthritis with rheumatoid factor of shoulder without organ or systems involvement
M05.711	Rheumatoid arthritis with rheumatoid factor of right shoulder without organ or systems involvement
M05.712	Rheumatoid arthritis with rheumatoid factor of left shoulder without organ or systems involvement
M05.719	Rheumatoid arthritis with rheumatoid factor of unspecified shoulder without organ or systems involvement
M05.72	Rheumatoid arthritis with rheumatoid factor of elbow without organ or systems involvement
M05.721	Rheumatoid arthritis with rheumatoid factor of right elbow without organ or systems involvement
M05.722	Rheumatoid arthritis with rheumatoid factor of left elbow without organ or systems involvement
M05.729	Rheumatoid arthritis with rheumatoid factor of unspecified elbow without organ or systems involvement
M05.73	Rheumatoid arthritis with rheumatoid factor of wrist without organ or systems involvement
M05.731	Rheumatoid arthritis with rheumatoid factor of right wrist without organ or systems involvement
M05.732	Rheumatoid arthritis with rheumatoid factor of left wrist without organ or systems involvement
M05.739	Rheumatoid arthritis with rheumatoid factor of unspecified wrist without organ or systems involvement
M05.74	Rheumatoid arthritis with rheumatoid factor of hand without organ or systems involvement
M05.741	Rheumatoid arthritis with rheumatoid factor of right hand without organ or systems involvement
M05.742	Rheumatoid arthritis with rheumatoid factor of left hand without organ or systems involvement
M05.749	Rheumatoid arthritis with rheumatoid factor of unspecified hand without organ or systems involvement
M05.75	Rheumatoid arthritis with rheumatoid factor of hip without organ or systems involvement
M05.751	Rheumatoid arthritis with rheumatoid factor of right hip without organ or systems involvement
M05.752	Rheumatoid arthritis with rheumatoid factor of left hip without organ or systems involvement
M05.759	Rheumatoid arthritis with rheumatoid factor of unspecified hip without organ or systems involvement

M05.76	Rheumatoid arthritis with rheumatoid factor of knee without organ or systems involvement
M05.761	Rheumatoid arthritis with rheumatoid factor of right knee without organ or systems involvement
M05.762	Rheumatoid arthritis with rheumatoid factor of left knee without organ or systems involvement
M05.769	Rheumatoid arthritis with rheumatoid factor of unspecified knee without organ or systems involvement
M05.77	Rheumatoid arthritis with rheumatoid factor of ankle and foot without organ or systems involvement
M05.771	Rheumatoid arthritis with rheumatoid factor of right ankle and foot without organ or systems involvement
M05.772	Rheumatoid arthritis with rheumatoid factor of left ankle and foot without organ or systems involvement
M05.779	Rheumatoid arthritis with rheumatoid factor of unspecified ankle and foot without organ or systems involvement
M05.79	Rheumatoid arthritis with rheumatoid factor of multiple sites without organ or systems involvement
M05.7°	Rheumatoid arthritis with rheumatoid factor of other specified site without organ or systems involvement
M05.8	Other seropositive rheumatoid arthritis
M05.80	Other seropositive rheumatoid arthritis, multiple sites
M05.81	Other seropositive rheumatoid arthritis, shoulder region
M05.811	Other rheumatoid arthritis with rheumatoid factor of right shoulder
M05.812	Other rheumatoid arthritis with rheumatoid factor of left shoulder
M05.819	Other rheumatoid arthritis with rheumatoid factor of unspecified shoulder
M05.82	Other seropositive rheumatoid arthritis, upper arm
M05.821	Other rheumatoid arthritis with rheumatoid factor of right elbow
M05.822	Other rheumatoid arthritis with rheumatoid factor of left elbow
M05.829	Other rheumatoid arthritis with rheumatoid factor of unspecified elbow
M05.83	Other seropositive rheumatoid arthritis, forearm
M05.831	Other rheumatoid arthritis with rheumatoid factor of right wrist
M05.832	Other rheumatoid arthritis with rheumatoid factor of left wrist
M05.839	Other rheumatoid arthritis with rheumatoid factor of unspecified wrist
M05.84	Other seropositive rheumatoid arthritis, hand
M05.841	Other rheumatoid arthritis with rheumatoid factor of right hand
M05.842	Other rheumatoid arthritis with rheumatoid factor of left hand
M05.849	Other rheumatoid arthritis with rheumatoid factor of unspecified hand
M05.85	Other seropositive rheumatoid arthritis, pelvic region and thigh
M05.851	Other rheumatoid arthritis with rheumatoid factor of right hip
M05.852	Other rheumatoid arthritis with rheumatoid factor of left hip
M05.859	Other rheumatoid arthritis with rheumatoid factor of unspecified hip
M05.86	Other seropositive rheumatoid arthritis, lower leg
M05.861	Other rheumatoid arthritis with rheumatoid factor of right knee
M05.862	Other rheumatoid arthritis with rheumatoid factor of left knee
M05.869	Other rheumatoid arthritis with rheumatoid factor of unspecified knee
M05.87	Other seropositive rheumatoid arthritis, ankle and foot
M05.871	Other rheumatoid arthritis with rheumatoid factor of right ankle and foot
M05.872	Other rheumatoid arthritis with rheumatoid factor of left ankle and foot
M05.879	Other rheumatoid arthritis with rheumatoid factor of unspecified ankle and foot
M05.88	Other seropositive rheumatoid arthritis, other
M05.89	Other seropositive rheumatoid arthritis, site unspecified
M05.8°	Other rheumatoid arthritis with rheumatoid factor of other specified site
M05.9	Seropositive rheumatoid arthritis, unspecified
M05.90	Seropositive rheumatoid arthritis, unspecified, multiple sites
M05.91	Seropositive rheumatoid arthritis, unspecified, shoulder region
M05.92	Seropositive rheumatoid arthritis, unspecified, upper arm
M05.93	Seropositive rheumatoid arthritis, unspecified, forearm
M05.94	Seropositive rheumatoid arthritis, unspecified, hand
M05.95	Seropositive rheumatoid arthritis, unspecified, pelvic region and thigh
M05.96	Seropositive rheumatoid arthritis, unspecified, lower leg
M05.97	Seropositive rheumatoid arthritis, unspecified, ankle and foot
M05.98	Seropositive rheumatoid arthritis, unspecified, other
M05.99	Seropositive rheumatoid arthritis, unspecified, site unspecified
M06	Other rheumatoid arthritis

M06.0	Seronegative rheumatoid arthritis
M06.00	Seronegative rheumatoid arthritis, multiple sites
M06.01	Seronegative rheumatoid arthritis, shoulder region
M06.011	Rheumatoid arthritis without rheumatoid factor, right shoulder
M06.012	Rheumatoid arthritis without rheumatoid factor, left shoulder
M06.019	Rheumatoid arthritis without rheumatoid factor, unspecified shoulder
M06.02	Seronegative rheumatoid arthritis, upper arm
M06.021	Rheumatoid arthritis without rheumatoid factor, right elbow
M06.022	Rheumatoid arthritis without rheumatoid factor, left elbow
M06.029	Rheumatoid arthritis without rheumatoid factor, unspecified elbow
M06.03	Seronegative rheumatoid arthritis, forearm
M06.031	Rheumatoid arthritis without rheumatoid factor, right wrist
M06.032	Rheumatoid arthritis without rheumatoid factor, left wrist
M06.039	Rheumatoid arthritis without rheumatoid factor, unspecified wrist
M06.04	Seronegative rheumatoid arthritis, hand
M06.041	Rheumatoid arthritis without rheumatoid factor, right hand
M06.042	Rheumatoid arthritis without rheumatoid factor, left hand
M06.049	Rheumatoid arthritis without rheumatoid factor, unspecified hand
M06.05	Seronegative rheumatoid arthritis, pelvic region and thigh
M06.051	Rheumatoid arthritis without rheumatoid factor, right hip
M06.052	Rheumatoid arthritis without rheumatoid factor, left hip
M06.059	Rheumatoid arthritis without rheumatoid factor, unspecified hip
M06.06	Seronegative rheumatoid arthritis, lower leg
M06.061	Rheumatoid arthritis without rheumatoid factor, right knee
M06.062	Rheumatoid arthritis without rheumatoid factor, left knee
M06.069	Rheumatoid arthritis without rheumatoid factor, unspecified knee
M06.07	Seronegative rheumatoid arthritis, ankle and foot
M06.071	Rheumatoid arthritis without rheumatoid factor, right ankle and foot
M06.072	Rheumatoid arthritis without rheumatoid factor, left ankle and foot
M06.079	Rheumatoid arthritis without rheumatoid factor, unspecified ankle and foot
M06.08	Seronegative rheumatoid arthritis, other
M06.09	Seronegative rheumatoid arthritis, site unspecified
M06.0A	Rheumatoid arthritis without rheumatoid factor, other specified site
M06.8	Other specified rheumatoid arthritis
M06.80	Other specified rheumatoid arthritis, multiple sites
M06.81	Other specified rheumatoid arthritis, shoulder region
M06.811	Other specified rheumatoid arthritis, right shoulder
M06.812	Other specified rheumatoid arthritis, left shoulder
M06.819	Other specified rheumatoid arthritis, unspecified shoulder
M06.82	Other specified rheumatoid arthritis, upper arm
M06.821	Other specified rheumatoid arthritis, right elbow
M06.822	Other specified rheumatoid arthritis, left elbow
M06.829	Other specified rheumatoid arthritis, unspecified elbow
M06.83	Other specified rheumatoid arthritis, forearm
M06.831	Other specified rheumatoid arthritis, right wrist
M06.832	Other specified rheumatoid arthritis, left wrist
M06.839	Other specified rheumatoid arthritis, unspecified wrist
M06.84	Other specified rheumatoid arthritis, hand
M06.841	Other specified rheumatoid arthritis, right hand
M06.842	Other specified rheumatoid arthritis, left hand
M06.849	Other specified rheumatoid arthritis, unspecified hand
M06.85	Other specified rheumatoid arthritis, pelvic region and thigh
M06.851	Other specified rheumatoid arthritis, right hip
M06.852	Other specified rheumatoid arthritis, left hip
M06.859	Other specified rheumatoid arthritis, unspecified hip

M06.86	Other specified rheumatoid arthritis, lower leg
M06.861	Other specified rheumatoid arthritis, right knee
M06.862	Other specified rheumatoid arthritis, left knee
M06.869	Other specified rheumatoid arthritis, unspecified knee
M06.87	Other specified rheumatoid arthritis, ankle and foot
M06.871	Other specified rheumatoid arthritis, right ankle and foot
M06.872	Other specified rheumatoid arthritis, left ankle and foot
M06.879	Other specified rheumatoid arthritis, unspecified ankle and foot
M06.88	Other specified rheumatoid arthritis, other
M06.89	Other specified rheumatoid arthritis, site unspecified
M06.8°	Other specified rheumatoid arthritis, other specified site
M06.9	Rheumatoid arthritis, unspecified
M06.90	Rheumatoid arthritis, unspecified, multiple sites
M06.91	Rheumatoid arthritis, unspecified, shoulder region
M06.92	Rheumatoid arthritis, unspecified, upper arm
M06.93	Rheumatoid arthritis, unspecified, forearm
M06.94	Rheumatoid arthritis, unspecified, hand
M06.95	Rheumatoid arthritis, unspecified, pelvic region and thigh
M06.96	Rheumatoid arthritis, unspecified, lower leg
M06.97	Rheumatoid arthritis, unspecified, ankle and foot
M06.98	Rheumatoid arthritis, unspecified, other
M06.99	Rheumatoid arthritis, unspecified, site unspecified
N04..	Rheumatoid arthritis and other inflammatory polyarthropathy
N040.	Rheumatoid arthritis
N0400	Rheumatoid arthritis of cervical spine
N0401	Other rheumatoid arthritis of spine
N0402	Rheumatoid arthritis of shoulder
N0403	Rheumatoid arthritis of sternoclavicular joint
N0404	Rheumatoid arthritis of acromioclavicular joint
N0405	Rheumatoid arthritis of elbow
N0406	Rheumatoid arthritis of distal radioulnar joint
N0407	Rheumatoid arthritis of wrist
N0408	Rheumatoid arthritis of metacarpophalangeal joint
N0409	Rheumatoid arthritis of PIP joint of finger
N040A	Rheumatoid arthritis of DIP joint of finger
N040B	Rheumatoid arthritis of hip
N040C	Rheumatoid arthritis of sacroiliac joint
N040D	Rheumatoid arthritis of knee
N040E	Rheumatoid arthritis of tibiofibular joint
N040F	Rheumatoid arthritis of ankle
N040G	Rheumatoid arthritis of subtalar joint
N040H	Rheumatoid arthritis of talonavicular joint
N040J	Rheumatoid arthritis of other tarsal joint
N040K	Rheumatoid arthritis of first metatarsophalangeal joint
N040L	Rheumatoid arthritis of lesser metatarsophalangeal joint
N040M	Rheumatoid arthritis of interphalangeal joint of toe
N040N	Rheumatoid vasculitis
N040P	Seronegative rheumatoid arthritis
N040Q	Rheumatoid bursitis
N040R	Rheumatoid nodule
N040S	Rheumatoid arthritis – multiple joint
N040T	Flare of rheumatoid arthritis
N041.	Feltys syndrome
N042.	Other rheumatoid arthropathy + visceral/systemic involvement
N0420	Rheumatic carditis

N0421	Rheumatoid lung disease
N0422	Rheumatoid nodule
N042z	Rheumatoid arthropathy + visceral/systemic involvement NOS
N043.	Juvenile rheumatoid arthritis – Stills disease
N0430	Juvenile rheumatoid arthropathy unspecified
N0431	Acute polyarticular juvenile rheumatoid arthritis
N0432	Pauciarticular juvenile rheumatoid arthritis
N0433	Monarticular juvenile rheumatoid arthritis
N043z	Juvenile rheumatoid arthritis NOS
N044.	Chronic post-rheumatic arthropathy
N045.	Other juvenile arthritis
N0455	Juvenile rheumatoid arthritis
N047.	Seropositive errosive rheumatoid arthritis
N04X.	Seropositive rheumatoid arthritis, unspecified
N04y0	Rheumatoid lung
Nyu10	[X]Rheumatoid arthritis+involvement/other organs or systems
Nyu11	[X]Other seropositive rheumatoid arthritis
Nyu12	[X]Other specified rheumatoid arthritis
Nyu1G	[X]Seropositive rheumatoid arthritis, unspecified
U50.4	Delivery of rehabilitation for rheumatoid arthritis
X701h	Seropositive rheumatoid arthritis
X701i	Seronegative rheumatoid arthritis
X701j	Rheumatoid arthritis with organ / system involvement
X701k	Fibrosing alveolitis associated with rheumatoid arthritis
X701l	Rheumatoid vasculitis
X701m	Rheumatoid arthritis with multisystem involvement
X701t	Juvenile rheumatoid arthritis
Xa3gL	Rheumatoid arthritis – multiple joint
Xa3gM	Rheumatoid arthritis – hand joint
Xa3gN	Rheumatoid arthritis – ankle/foot
Xa3gO	Rheumatoid arthritis – other joint
Xa3gP	Rheumatoid arthritis NOS
Xa3wk	Stills disease – juvenile rheumatoid arthritis
XaaWt	Rheumatoid arthritis monitoring invitation first letter
XaaWu	Rheumatoid arthritis monitoring invitation second letter
XaaWv	Rheumatoid arthritis monitoring invitation third letter
XaaWw	Rheumatoid arthritis monitoring verbal invitation
XaaWx	Rheumatoid arthritis monitoring telephone invitation
XaBMO	Seropositive errosive rheumatoid arthritis
XaEQw	Pm-1 – Polymyositis-scleroderma antibody level
Xaex7	Rheumatoid Arthritis Impact of Disease questionnaire
Xafj9	Rheumatoid arthritis 20ubule20ing SMS text msg first invitation
XafjA	Rheumatoid arthritis monitoring SMS text mesg 2nd invitation
XafjB	Rheumatoid arthritis monitoring SMS text mesg 3rd invitation
XagLZ	Rheumatoid arthritis monitoring invitation email
XaJUC	Rheumatoid arthritis particle agglutination test
XaLsj	Delivery of rehabilitation for rheumatoid arthritis
XaN2K	Disease activity score in rheumatoid arthritis
XaYSO	Exception reporting: rheumatoid arthritis quality indicators
XaYSP	Except rheumatoid arthritis quality indicator: pt unsuitable
XaYSR	Except rheumatoid arthritis qual indicator: informed dissent
XaYT6	Disease activity score 28 joint in rheumatoid arthritis
XaZdB	Rheumatoid arthritis annual review
XE1DU	Rheumatoid arthritis and other inflammatory polyarthropathy
XE1Gi	Juvenile rheumatoid arthritis &/or Stills disease

	XM1XV	Rheumatoid arthritis monitoring
Systemic sclerosis		
	1144925008	Diffuse systemic sclerosis of childhood
	1197016009	Gingival disease due to systemic sclerosis
	128457007	Scleroderma
	128459005	Systemic sclerosis, limited
	128460000	Systemic sclerosis, diffuse
	128461001	Systemic sclerosis sine scleroderma
	147833014	Systemic sclerosis
	156451000	Scleroderma (& [systemic sclerosis])
	166184008	Pm-1 – Polymyositis-scleroderma antibody level
	193252005	Myopathy due to systemic sclerosis
	196133001	Lung disease with systemic sclerosis
	201440007	Systemic sclerosis
	201443009	Systemic sclerosis induced by drugs and chemicals
	203786003	[X]Other forms of systemic sclerosis
	268049000	Systemic sclerosis
	2837073016	Systemic sclerosis due to chemical
	2840902011	PSS – Progressive systemic sclerosis
	2841088016	Family history of systemic sclerosis
	2842233014	Systemic sclerosis due to chemical
	298285004	Systemic sclerosis with limited cutaneous involvement
	299276009	Limited systemic sclerosis
	309416016	Systemic sclerosis induced by drugs and chemicals
	31848007	CREST syndrome (disorder)
	3.22461E+14	Polyneuropathy due to systemic sclerosis
	35719004	Dilated cardiomyopathy due to systemic sclerosis
	402713007	Cutaneous complication of systemic sclerosis
	403514006	Calcinosis cutis due to systemic sclerosis
	403515007	Hypermelanosis due to systemic sclerosis
	403516008	Telangiectasia due to systemic sclerosis
	403517004	Telangiectasia of nailfolds due to systemic sclerosis
	403519001	Poikiloderma due to systemic sclerosis
	403520007	Nail dystrophy due to systemic sclerosis
	409532019	Systemic lupus erythematosus disease activity index
	4.24881E+14	[X]Other forms of systemic sclerosis
	43aA.	Pm-1 – Polymyositis-scleroderma antibody level
	443872005	Systemic sclerosis caused by chemical
	444133002	PSS – Progressive systemic sclerosis
	508375011	SS – Systemic sclerosis
	5.09461E+14	PSS – Progressive systemic sclerosis
	517.2	Lung involvement in systemic sclerosis
	63115005	Hypothyroidism due to systemic sclerosis
	87442008	Pericarditis due to systemic sclerosis
	88702-6	Systemic sclerosis panel – Serum by Immunoassay
	89155008	SS – Systemic sclerosis
	9.05371E+14	Polymyositis/scleroderma overlap syndrome
	9.23421E+14	[X]Other forms of systemic sclerosis
	H572.	Lung disease with systemic sclerosis
	M34	Systemic sclerosis
	M34.0	Progressive systemic sclerosis
	M34.2	Systemic sclerosis induced by drugs and chemicals
	M34.8	Other forms of systemic sclerosis
	M34.81	Systemic sclerosis with lung involvement
	M34.82	Systemic sclerosis with myopathy

	M34.83	Systemic sclerosis with polyneuropathy
	M34.89	Other systemic sclerosis
	M34.9	Systemic sclerosis, unspecified
	N001.	Scleroderma (& [acrosclerosis] or [systemic sclerosis])
	N0010	Progressive systemic sclerosis
	N0011	CREST syndrome
	N0012	Systemic sclerosis induced by drugs and chemicals
	Nyu45	[X]Other forms of systemic sclerosis
	X50FL	Sclerodermatomyositis
	XaQbV	Progressive systemic sclerosis
	XE1Gc	Scleroderma (& [systemic sclerosis])
Inflammatory miopathies-related ILDs		
	1144944001	Polymyositis overlap syndrome
	1144974009	Antisynthetase syndrome due to polymyositis
	1153408000	Calcification of muscle due to adult dermatomyositis
	1153428001	Calcification of muscle due to juvenile dermatomyositis
	116959007	Polymyositis/scleroderma extractable nuclear antibody
	122228002	Polymyositis-scleroderma (PM-Scl) extractable nuclear antibody assay
	143547002	Pm-1 – Polymyositis-scleroderma antibody level
	156456005	Dermatomyositis
	156457001	Polymyositis
	1776211019	Dermatomyositis
	1785514012	DM – Dermatomyositis
	196136009	Lung disease with polymyositis
	201445002	Dermatomyositis
	201446001	Juvenile dermatomyositis
	201447005	Dermatomyositis with malignant disease
	201448000	[X]Dermatopolymyositis, unspecified
	203110007	Myositis unspecified
	203111006	Fibromyositis NOS (disorder)
	203113009	Myalgia or myositis NOS
	203785004	[X]Other dermatomyositis
	203795006	[X]Dermatopolymyositis, unspecified
	238935002	Dermatomyositis sine myositis
	238936001	Sclerodermatomyositis
	239898008	Polymyositis with malignant disease
	239899000	Polymyositis associated with autoimmune disease
	239901009	Dermatopolymyositis in neoplastic disease
	240120003	Eosinophilic polymyositis
	24693007	Fibromyositis
	2.56582E+15	Pm-1 – Polymyositis-scleroderma antibody level
	26889001	Myositis
	281357005	Idiopathic polymyositis
	281358000	Idiopathic dermatomyositis
	301719013	Lung disease with polymyositis
	30701005	Undifferentiated inflammatory polyarthritis
	309422013	Dermatomyositis (& [Poikilodermatomyositis])
	31384009	PM – Polymyositis
	3.17941E+14	Myopathy co-occurrent and due to polymyositis
	32660008	Pm-1 – Polymyositis-scleroderma antibody level
	3458000	Polymyositis ossificans
	351390015	Acute sarcoid polymyositis
	358091015	Dermatomyositis sine myositis
	358092010	Sclerodermatomyositis
	359456011	Polymyositis with malignant disease

359457019	Polymyositis associated with autoimmune disease
359459016	Dermatopolymyositis in neoplastic disease
359460014	Dermatomyositis with malignant disease
359738012	Eosinophilic polymyositis
387119010	Pm-Scl – Polymyositis-scleroderma antibody
387120016	Pm-1 – Polymyositis-scleroderma antibody
38826005	Polymyositis with skin involvement
396229003	Adult type dermatomyositis
396230008	Polymyositis with skin involvement
402425006	Adult onset dermatomyositis
4.02621E+14	[X]Dermatopolymyositis, unspecified
419345010	Idiopathic polymyositis
419346011	Idiopathic dermatomyositis
4.19921E+14	[X]Dermatopolymyositis, unspecified
4.62101E+14	[X]Other dermatomyositis
46696008	Dilated cardiomyopathy due to dermatomyositis
484857016	PM – Polymyositis
485880017	Myositis ossificans associated with dermato / polymyositis
52463013	Polymyositis
586317016	Dermatomyositis (& [Poikilodermatomyositis])
6.67611E+14	Myalgia and myositis unspecified
6.67631E+14	Myalgia or myositis NOS
6.67941E+14	Myositis unspecified (disorder)
6.69031E+14	Fibromyositis NOS (disorder)
6839012	Polymyositis ossificans
710.3	Dermatomyositis
710.4	Polymyositis
838312001	Calcinosis due to childhood type dermatomyositis
838366009	Anti-synthetase syndrome due to dermatomyositis
9.39011E+14	[X]Dermatopolymyositis, unspecified
9.51121E+14	[X]Other dermatomyositis
H57y1	Lung disease with polymyositis
M33	Dermatopolymyositis
M33.1	Other dermatomyositis
M33.10	Other dermatomyositis, organ involvement unspecified
M33.11	Other dermatomyositis with respiratory involvement
M33.12	Other dermatomyositis with myopathy
M33.13	Other dermatomyositis without myopathy
M33.19	Other dermatomyositis with other organ involvement
M33.2	Polymyositis
M33.20	Polymyositis, organ involvement unspecified
M33.21	Polymyositis with respiratory involvement
M33.22	Polymyositis with myopathy
M33.29	Polymyositis with other organ involvement
M33.9	Dermatopolymyositis, unspecified
M33.90	Dermatopolymyositis, unspecified, organ involvement unspecified
M33.91	Dermatopolymyositis, unspecified with respiratory involvement
M33.92	Dermatopolymyositis, unspecified with myopathy
M33.93	Dermatopolymyositis, unspecified without myopathy
M33.99	Dermatopolymyositis, unspecified with other organ involvement
N003.	Dermatomyositis (& [Poikilodermatomyositis])
N0030	Juvenile dermatomyositis
N0031	Dermatopolymyositis in neoplastic disease
N003X	Dermatopolymyositis, unspecified
N004.	Polymyositis

	N241.	Myalgia and myositis unspecified
	N2411	Myositis unspecified
	N2412	Fibromyositis NOS
	N241z	Myalgia or myositis NOS
	Nyu44	[X]Other dermatomyositis
	Nyu4E	[X]Dermatopolymyositis, unspecified
	X20GU	Acute sarcoid polymyositis
	X50FJ	Dermatomyositis sine myositis
	X704p	Polymyositis with malignant disease
	X704q	Polymyositis associated with autoimmune disease
	X704t	Dermatomyositis with malignant disease
	X704u	Juvenile dermatomyositis
	Xa1jK	Idiopathic polymyositis
	Xa1jL	Idiopathic dermatomyositis
	XE1DH	Dermatomyositis
LES-related ILDs		
	11013005	Systemic lupus erythematosus glomerulonephritis syndrome, World Health Organization class VI
	1144921004	Disorder of heart due to systemic lupus erythematosus
	1144923001	Disorder of immune function due to systemic lupus erythematosus
	1144927000	Disorder of gastrointestinal tract due to systemic lupus erythematosus
	1144942002	Neuropsychiatric disorder due to systemic lupus erythematosus
	1144970000	Joint disorder due to systemic lupus erythematosus
	1144973003	Disorder of kidney due to systemic lupus erythematosus
	1231480012	SLE – Systemic lupus erythematosus
	13902000	Discoid lupus erythematosus (disorder) [Ambiguous]
	156365002	Discoid lupus erythematosus
	156450004	Systemic lupus erythematosus
	158442019	Systemic lupus erythematosus encephalitis
	197608009	Nephrotic syndrome in systemic lupus erythematosus
	197746009	Renal 24ubule-interstitial disorder in systemic lupus erythematosus
	200936003	Lupus erythematosus
	200938002	DLE – Discoid lupus erythematosus
	200943009	Subacute cutaneous lupus erythematosus
	200944003	Lupus erythematosus NOS (disorder)
	201435004	Systemic lupus erythematosus
	201437007	Systemic lupus erythematosus with organ/system involvement
	201438002	Systemic lupus erythematosus with pericarditis
	201439005	Systemic lupus erythematosus NOS
	203784000	[X]Other forms of systemic lupus erythematosus
	230307005	Chorea in systemic lupus erythematosus
	239887007	Systemic lupus erythematosus with organ/system involvement
	239889005	Bullous systemic lupus erythematosus
	239890001	Systemic lupus erythematosus with multisystem involvement
	239891002	SCLE – Subacute cutaneous lupus erythematosus
	25380002	Pericarditis co-occurrent and due to systemic lupus erythematosus
	273862004	Systemic lupus activity measure (assessment scale)
	273863009	Systemic lupus erythematosus disease activity index
	2.95101E+14	Nephropathy co-occurrent and due to systemic lupus erythematosus
	2.95111E+14	Systemic lupus erythematosus co-occurrent and due to nephrotic syndrome
	2.95121E+14	Nephrosis with systemic lupus erythematosus
	301721015	Lung disease with systemic lupus erythematosus
	307755009	Renal 24ubule-interstitial disorder in systemic lupus erythematosus
	3.08751E+14	Glomerular disease due to systemic lupus erythematosus
	309762007	Systemic lupus erythematosus with pericarditis
	345133010	Chorea in systemic lupus erythematosus

359443016	Systemic lupus erythematosus with organ/system involvement
359445011	Bullous systemic lupus erythematosus
359446012	Systemic lupus erythematosus with multisystem involvement
36402006	Systemic lupus erythematosus glomerulonephritis syndrome, World Health Organization class IV
36471000	Dilated cardiomyopathy due to systemic lupus erythematosus
397856003	Systemic lupus erythematosus-related syndrome
402865003	Systemic lupus erythematosus-associated antiphospholipid syndrome
403486000	Acute systemic lupus erythematosus
403487009	Fulminating systemic lupus erythematosus
403488004	Systemic lupus erythematosus of childhood
4.09421E+14	[X]Other forms of systemic lupus erythematosus
409529017	Systemic lupus activity measure
409530010	SLAM – Systemic lupus activity measure
409531014	SLEDAI – Systemic lupus erythematosus disease activity index
417303004	Retinal vasculitis due to systemic lupus erythematosus
451122013	Renal 25ubule-interstitial disorder in systemic lupus erythematosus
453243010	Systemic lupus erythematosus with pericarditis
4676006	Systemic lupus erythematosus glomerulonephritis syndrome, World Health Organization class II
512235016	Cerebral systemic lupus erythematosus
52042003	Systemic lupus erythematosus glomerulonephritis syndrome, World Health Organisation class V
55464009	Systemic lupus erythematosus
5.90791E+14	Lupus erythematosus NOS
5.93481E+14	Systemic lupus erythematosus NOS
68815009	Systemic lupus erythematosus glomerulonephritis syndrome
698694005	Systemic lupus erythematosus in remission
710	Systemic lupus erythematosus
710.1	Systemic sclerosis
7.2181E+13	Endocarditis due to systemic lupus erythematosus
724767000	Chorea co-occurrent and due to systemic lupus erythematosus
724781003	Demyelination of central nervous system co-occurrent and due to systemic lupus erythematosus
73286009	Systemic lupus erythematosus glomerulonephritis syndrome, World Health Organization class I
732960002	Hemolytic anemia associated with systemic lupus erythematosus
773333003	Autosomal SLE (systemic lupus erythematosus)
9.05381E+14	Systemic lupus erythematosus/Sjogren's overlap syndrome
92208011	Systemic lupus erythematosus
9.41171E+14	[X]Other forms of systemic lupus erythematosus
95332009	Rash of systemic lupus erythematosus
95408003	Systemic lupus erythematosus arthritis
95644001	Systemic lupus erythematosus encephalitis
H57y4	Lung disease with systemic lupus erythematosus
K01x4	Nephrotic syndrome in systemic lupus erythematosus
LA14306-7	SLE (systemic lupus)
LA15300-9	Systemic lupus erythematosus (SLE)
LP116304-9	PhenX – systemic lupus erythematosus protocol 171001
M154.	Lupus erythematosus
M1541	Discoid lupus erythematosus
M1547	Subacute cutaneous lupus erythematosus
M154z	Lupus erythematosus NOS
M32	Systemic lupus erythematosus
M32.1	Systemic lupus erythematosus with organ or system involvement
M32.10	Systemic lupus erythematosus, organ or system involvement unspecified
M32.11	Endocarditis in systemic lupus erythematosus
M32.12	Pericarditis in systemic lupus erythematosus
M32.13	Lung involvement in systemic lupus erythematosus
M32.14	Glomerular disease in systemic lupus erythematosus

	M32.15	Tubulo-interstitial nephropathy in systemic lupus erythematosus
	M32.19	Other organ or system involvement in systemic lupus erythematosus
	M32.8	Other forms of systemic lupus erythematosus
	M32.9	Systemic lupus erythematosus, unspecified
	N000.	Systemic lupus erythematosus
	N0000	Disseminated lupus erythematosus
	N0003	Systemic lupus erythematosus with organ or sys involv
	N0004	Systemic lupus erythematosus with pericarditis
	N000z	Systemic lupus erythematosus NOS
	Nyu43	[X]Other forms of systemic lupus erythematosus
	X0046	Chorea in systemic lupus erythematosus
	X50Ex	Chronic discoid lupus erythematosus
	X704b	Bullous systemic lupus erythematosus
	X704c	Systemic lupus erythematosus with multisystem involvement
	X704h	Subacute cutaneous lupus erythematosus
	X704X	Systemic lupus erythematosus with organ/system involvement
	XaBE1	Renal 26ubule-interstitial disord in systemic lupus erythemat
	XaC1J	Systemic lupus erythematosus with pericarditis
	XM0ji	Systemic lupus activity measure
	XM0jj	Systemic lupus erythematosus disease activity index
Miscellaneous		
	0062U	Autoimmune (systemic lupus erythematosus), IgG and IgM analysis of 80 biomarkers, utilizing serum, algorithm reported with a risk score
	1.01127E+15	Serum SSA 60 (Sjogren’s syndrome A 60 kiloDalton) antibody concentration
	1.01131E+15	Serum SSA52 (Sjogren’s syndrome A 52 kiloDalton) antibody concentration
	1.01673E+15	Pm-1 – Polymyositis-scleroderma antibody level
	105969002	Connective tissue diseases
	1144911007	Calcinosis due to adult type dermatomyositis
	1144915003	Paraneoplastic juvenile polymyositis
	1144931006	Dermatomyositis overlap syndrome
	1144977002	Hypomyopathic juvenile dermatomyositis
	1212005	Childhood type dermatomyositis
	1219184017	Connective tissue disease
	1227887018	SS-B – Sjogren’s syndrome – B antibody
	1227888011	Sjogren’s syndrome – B antibody
	1230069014	Sjogren’s syndrome – A antibody
	1230070010	SS-A – Sjogren’s syndrome – A antibody
	126766000	Myoepithelial sialadenitis in Sjogren’s syndrome
	139131019	Sjogren’s syndrome
	139132014	Sjogren’s disease
	148071003	Rheumatology disorder monitoring (& arthritis [& rheumatoid])
	14GC.	History of connective tissue disease
	156484000	Polyarthropathy (& [inflammatory]) NOS
	156485004	Seronegative arthritis
	156618002	Inflammatory spondylopathies (finding)
	156656001	Polymyalgia rheumatica (disorder)
	170845006	Rheumatology disorder monitoring (& arthritis [& rheumatoid])
	1.74771E+15	Interstitial lung disease due to connective tissue disease
	17583-6	Sjogrens syndrome-A extractable nuclear Ab [Presence] in Serum by Immunofluorescence
	17584-4	Sjogrens syndrome-A extractable nuclear Ab [Units/volume] in Serum by Immunofluorescence
	17585-1	Sjogrens syndrome-A extractable nuclear IgG Ab [Presence] in Serum by Immunoassay
	17586-9	Sjogrens syndrome-B extractable nuclear Ab [Presence] in Serum by Immunofluorescence
	17587-7	Sjogrens syndrome-B extractable nuclear Ab [Units/volume] in Serum by Immunofluorescence
	17588-5	Sjogrens syndrome-B extractable nuclear IgG Ab [Presence] in Serum by Immunoassay
	17791-5	Sjogrens syndrome-B extractable nuclear Ab [Units/volume] in Serum
	17792-3	Sjogrens syndrome-A extractable nuclear Ab [Units/volume] in Serum

1786074016	Mixed connective tissue disease
1786075015	MCTD – Mixed connective tissue disease
1.87391E+14	Pm-1 – Polymyositis-scleroderma antibody level
193177003	Neuropathy in vasculitis and connective tissue disease
193253000	Myopathy due to Sjogren’s disease
1961000	Chronic polyarticular juvenile rheumatoid arthritis
196137000	Lung disease with Sjogren’s disease
196138005	Lung disease with systemic lupus erythematosus
200956002	Psoriatic arthritis with spine involvement
200959009	Psoriatic arthropathy NOS
201049004	Morphea
201432001	Musculoskeletal and connective tissue diseases
201444003	Sicca (Sjogren’s) syndrome
201763001	Inflammatory polyarthropathy: (& [rheumatoid arthritis] or [other])
201801009	Other juvenile arthritis
201802002	Juvenile ankylosing spondylitis
201805000	Juvenile arthritis in Crohn disease
201807008	Juvenile arthritis in ulcerative colitis
201809006	Pauciarticular onset juvenile chronic arthritis
201812009	Other specified inflammatory polyarthropathy
201814005	Seronegative arthritis
201816007	Other specified inflammatory polyarthropathy NOS
201817003	Inflammatory polyarthropathy NOS (disorder)
202020005	(Polyarthropathy: [unspecified] or [NEC]) or (polyarthritis)
202649003	Inflammatory spondylopathy (disorder)
202834009	Polymyalgia
203108005	Myalgia and myositis unspecified (disorder)
203720007	[X]Additional musculoskeletal and connective tissue disease classification terms
203735008	[X]Other juvenile arthritis
203921002	Musculoskeletal and connective tissue diseases NOS
234529004	Acute sarcoid polymyositis
238881001	Panniculitis of atrophic connective tissue disease
238927000	Chronic discoid lupus erythematosus
239793008	Rheumatoid arthritis with organ / system involvement
239796000	Juvenile chronic arthritis
239803008	Juvenile arthritis in psoriasis
239805001	AS – Juvenile ankylosing spondylitis
239912009	Primary Sjogren’s syndrome
239913004	Primary Sjogren’s syndrome with organ/system involvement
239914005	Primary Sjogren’s syndrome with multisystem involvement
239915006	Secondary Sjogren’s syndrome
239916007	Secondary Sjogren’s syndrome with organ/system involvement
239917003	Secondary Sjogren’s syndrome with multisystem involvement
239918008	Undifferentiated connective tissue disease
239920006	Adult onset Still’s disease
2.41045E+15	Serum SSA 60 (Sjogren’s syndrome A 60 kiloDalton) antibody concentration measurement
2.4105E+15	Serum SSA 52 (Sjogren’s syndrome A 52 kiloDalton) antibody concentration measurement
258128000	Sjogren hand technique (qualifier value)
2.60743E+15	Serum SSA52 (Sjogren’s syndrome A 52 kiloDalton) antibody concentration
2.60744E+15	Serum SSA 60 (Sjogren’s syndrome A 60 kiloDalton) antibody concentration
267988003	Polymyalgia rheumatica
268050000	Sicca (Sjogren’s) syndrome
268053003	Polyarthropathy (& [inflammatory]) NOS (disorder)
270506005	Unspecified polyarthropathy or polyarthritis (disorder)
2.76681E+14	[X]Mixed connective tissue disease

2.92691E+14	Mixed connective tissue disease
2955491013	History of connective tissue disease
2.96241E+14	Disorder of respiratory system due to dermatomyositis
297541016	Neuropathy in vasculitis and connective tissue disease
297645018	Myopathy due to Sjogren's disease
29948-7	Sjogrens syndrome-A extractable nuclear Ab [Titer] in Serum by Immune diffusion (ID)
29949-5	Sjogrens syndrome-B extractable nuclear Ab [Titer] in Serum by Immune diffusion (ID)
29964-4	Sjogrens syndrome-A extractable nuclear IgG Ab [Units/volume] in Serum by Immunoassay
29965-1	Sjogrens syndrome-B extractable nuclear IgG Ab [Units/volume] in Serum by Immunoassay
301716018	Lung disease with systemic sclerosis
301720019	Lung disease with Sjogren's disease
3078136017	Interstitial lung disease due to connective tissue disease
309399013	Musculoskeletal &/or connective tissue diseases
312225001	Musculoskeletal and connective tissue diseases
31625-7	Sjogrens syndrome-A extractable nuclear IgG Ab [Presence] in Serum
31626-5	Sjogrens syndrome-B extractable nuclear IgG Ab [Presence] in Serum
31960007	SS-B – Sjogren's syndrome – B antibody
32041-6	Sjogrens syndrome-A extractable nuclear Ab+Sjogrens syndrome-B extractable nuclear Ab [Units/volume] in Serum
3.20681E+14	Tubulointerstitial nephropathy due to Sjogrens syndrome
32716-3	Sjogrens syndrome-A extractable nuclear Ab [Titer] in Serum
33110008	Connective tissue disease overlap syndrome
33339001	Psoriasis with arthropathy (disorder)
33566-1	Sjogrens syndrome-A extractable nuclear Ab [Units/volume] in Serum by Immunoblot
33569-5	Sjogrens syndrome-A extractable nuclear Ab [Units/volume] in Serum by Immunoassay
33570-3	Sjogrens syndrome-B extractable nuclear Ab [Units/volume] in Serum by Immunoblot
33583-6	Sjogrens syndrome-A extractable nuclear IgG Ab [Units/volume] in Serum by Immunoblot
33598-4	Sjogrens syndrome-B extractable nuclear IgG Ab [Units/volume] in Serum by Immunoblot
33610-7	Sjogrens syndrome-A extractable nuclear IgG Ab [Units/volume] in Serum
33613-1	Sjogrens syndrome-B extractable nuclear IgG Ab [Units/volume] in Serum
33719002	Subcutaneous rheumatoid nodule
358016014	Panniculitis of atrophic connective tissue disease
359173000	Spondarthropathy
359473013	Primary Sjogren's syndrome
359474019	Primary Sjogren's syndrome with organ/system involvement
359475018	Primary Sjogren's syndrome with multisystem involvement
359476017	Secondary Sjogren's syndrome
359477014	Secondary Sjogren's syndrome with organ/system involvement
359478016	Secondary Sjogren's syndrome with multisystem involvement
359479012	Undifferentiated connective tissue disease
36186002	Polyarthropathy
384169011	Sjogren hand technique
3.90181E+14	[X]Additional musculoskeletal and connective tissue disease classification terms
398021003	Connective tissue disease overlap syndrome
398049005	Mixed connective tissue disease
402657003	Necrotizing vasculitis secondary to connective tissue disease
403408001	Poikiloderma due to connective tissue disease
410793008	Chronic arthritis of juvenile onset
410797009	Juvenile seronegative polyarthritis (disorder)
416956002	Polyarthritis
417373000	Inflammatory polyarthritis
43100-7	Sjogrens syndrome-A and B extractable nuclear Ab panel – Serum
44212003	Sjogren's syndrome – A antibody
44833003	Pseudolymphoma of lung in Sjogren's disease
45142-7	Sjogrens syndrome-B extractable nuclear Ab [Units/volume] in Serum by Immunoassay
455899017	Musculoskeletal and connective tissue diseases

472961001	History of connective tissue disease
4.78451E+14	[X]Other juvenile arthritis (disorder)
5.12761E+14	History of connective tissue disease
5.12771E+14	History of connective tissue disease
53016-2	Sjogrens syndrome-A extractable nuclear 52kD Ab [Presence] in Serum
53017-0	Sjogrens syndrome-A extractable nuclear 52kD Ab [Units/volume] in Serum
53018-8	Sjogrens syndrome-A extractable nuclear 60kD Ab [Presence] in Serum
53019-6	Sjogrens syndrome-A extractable nuclear 60kD Ab [Units/volume] in Serum
5351-2	Sjogrens syndrome-A extractable nuclear Ab [Presence] in Serum by Immunoassay
5352-0	Sjogrens syndrome-A extractable nuclear Ab [Presence] in Serum by Immune diffusion (ID)
5353-8	Sjogrens syndrome-B extractable nuclear Ab [Presence] in Serum by Immunoassay
5354-6	Sjogrens syndrome-B extractable nuclear Ab [Presence] in Serum by Immune diffusion (ID)
56549-9	Sjogrens syndrome-A extractable nuclear 52kD Ab [Units/volume] in Serum by Immunoassay
56724-8	Sjogrens syndrome-A extractable nuclear Ab [Presence] in Serum by Immunoblot
56725-5	Sjogrens syndrome-B extractable nuclear Ab [Presence] in Serum by Immunoblot
586303013	Musculoskeletal &/or connective tissue diseases
59014-1	Sjogrens syndrome-A extractable nuclear 60kD Ab [Presence] in Serum by Immunoblot
63339-6	Sjogrens syndrome-A extractable nuclear 52kD Ab [Presence] in Body fluid by Immunoassay
63340-4	Sjogrens syndrome-A extractable nuclear 52kD Ab [Titer] in Body fluid by Immunoassay
63341-2	Sjogrens syndrome-A extractable nuclear 52kD Ab [Presence] in Cerebral spinal fluid by Immunoassay
63342-0	Sjogrens syndrome-A extractable nuclear 52kD Ab [Presence] in Serum by Immunoassay
63343-8	Sjogrens syndrome-A extractable nuclear 60kD Ab [Presence] in Body fluid by Immunoassay
63344-6	Sjogrens syndrome-A extractable nuclear 60kD Ab [Titer] in Body fluid by Immunoassay
63345-3	Sjogrens syndrome-A extractable nuclear Ab [Presence] in Body fluid by Flow cytometry (FC)
63346-1	Sjogrens syndrome-A extractable nuclear Ab [Presence] in Body fluid by Immune diffusion (ID)
63347-9	Sjogrens syndrome-A extractable nuclear Ab [Presence] in Body fluid by Immunoassay
63348-7	Sjogrens syndrome-A extractable nuclear Ab [Units/volume] in Body fluid by Immunoassay
63349-5	Sjogrens syndrome-B extractable nuclear Ab [Presence] in Body fluid by Immune diffusion (ID)
63350-3	Sjogrens syndrome-B extractable nuclear Ab [Presence] in Body fluid by Immunoassay
63351-1	Sjogrens syndrome-B extractable nuclear Ab [Presence] in Cerebral spinal fluid by Immunoassay
63401-4	Sjogrens syndrome-A extractable nuclear Ab [Units/volume] in Cerebral spinal fluid by Immunoassay
63402-2	Sjogrens syndrome-A extractable nuclear Ab [Presence] in Cerebral spinal fluid by Immunoassay
63403-0	Sjogrens syndrome-B extractable nuclear Ab [Units/volume] in Body fluid by Immunoassay
63404-8	Sjogrens syndrome-B extractable nuclear Ab [Units/volume] in Cerebral spinal fluid by Immunoassay
63411-3	Sjogrens syndrome-A extractable nuclear 60kD Ab [Presence] in Serum by Immunoassay
63445-1	Sjogrens syndrome-A extractable nuclear 52kD Ab [Presence] in Serum by Immunoblot
63543-3	Sjogrens syndrome-A extractable nuclear 52kD Ab [Presence] in Body fluid
63544-1	Sjogrens syndrome-A extractable nuclear 60kD Ab [Presence] in Body fluid
63545-8	Sjogrens syndrome-B extractable nuclear Ab [Presence] in Body fluid
65323003	Polymyalgia rheumatica
6.53491E+14	Other specified inflammatory polyarthropathy
6.53521E+14	Other specified inflammatory polyarthropathy NOS (disorder)
6.53531E+14	Inflammatory polyarthropathy NOS (disorder)
6.56641E+14	Other rheumatoid arthritis of spine
6.61371E+14	Musculoskeletal and connective tissue diseases NOS
6.89661E+14	Other juvenile arthritis
6.91671E+14	Unspecified polyarthropathy or polyarthritis
6.92631E+14	Psoriatic arthropathy NOS
69746005	Hemolytic anemia associated with systemic lupus erythematosus
697903007	Pulmonary arterial hypertension associated with connective tissue disease
70257-1	Sjogrens syndrome-A extractable nuclear 52kD IgG Ab [Units/volume] in Serum by Immunoassay
703119002	Carditis due to rheumatic fever (disorder)
703333006	Pericarditis due to vasculitis and connective tissue disease
710.9	Unspecified diffuse connective tissue disease
711379004	Interstitial lung disease due to connective tissue disease

724782005	Demyelination of central nervous system co-occurrent and due to Sjogren disease
7441009	Juvenile chronic polyarthritis
7.81711E+14	Interstitial lung disease due to connective tissue disease
78946008	Keratoconjunctivitis sicca, in Sjogren’s syndrome
8.07891E+14	Interstitial lung disease due to connective tissue disease
8093-7	Sjogrens syndrome-A extractable nuclear Ab [Presence] in Serum
8094-5	Sjogrens syndrome-B extractable nuclear Ab [Presence] in Serum
82725007	Diffuse progressive ossifying polymyositis
82933-3	Sjogrens syndrome-A extractable nuclear 52kD IgG Ab [Presence] in Serum by Line blot
83901003	Sicca (Sjogren’s) syndrome
840455002	Cutaneous vasculitis due to childhood type dermatomyositis
84801008	Jaccoud’s syndrome
860837007	Cardiomyopathy due to connective tissue disease
86365006	Poikilodermatomyositis
865924008	CDASI – Cutaneous Dermatomyositis Disease Area and Severity Index
866103007	Interstitial lung disease due to juvenile polymyositis
87555-9	Sjogrens syndrome-A and B extractable nuclear IgG panel – Serum
8.88161E+14	Carditis due to rheumatic fever (disorder)
89526-8	Sjogrens syndrome-B extractable nuclear Ab [Presence] in Cerebral spinal fluid by Line blot
89527-6	Sjogrens syndrome-A extractable nuclear Ab [Presence] in Cerebral spinal fluid by Line blot
89532-6	Sjogrens syndrome-A extractable nuclear 52kD Ab [Presence] in Cerebral spinal fluid by Line blot
8.96021E+14	[X]Additional musculoskeletal and connective tissue disease classification terms
9.41981E+14	Serum SSA 60 (Sjogren’s syndrome A 60 kiloDalton) antibody concentration
9.42001E+14	Serum SSA 52 (Sjogren’s syndrome A 52 kiloDalton) antibody concentration
95415006	Polymyalgia, NOS
99139-8	Sjogrens syndrome-A extractable nuclear 52kD Ab [Units/volume] in Serum by Line blot
F3967	Myopathy due to Sjogren’s disease
H57y3	Lung disease with Sjogren’s disease
H58y7	Interstitial lung disease due to connective tissue disease
LA31455-1	Connective tissue disease
LG11788-3	Sjogrens syndrome-A extractable nuclear Ab|Titr|Pt|ANYBldSerPl
LG11802-2	Sjogrens syndrome-A extractable nuclear Ab.IgG|Acnc|Pt|ANYBldSerPl
LG11803-0	Sjogrens syndrome-B extractable nuclear Ab.IgG|Acnc|Pt|ANYBldSerPl
LG21387-2	Sjogrens syndrome-A extractable nuclear 52kD Ab|PrThr|Pt|ANYBldSerPl
LG21388-0	Sjogrens syndrome-A extractable nuclear 52kD Ab|Acnc|Pt|ANYBldSerPl
LG21389-8	Sjogrens syndrome-A extractable nuclear 60kD Ab|PrThr|Pt|ANYBldSerPl
LG26775-3	Sjogrens syndrome-A extractable nuclear 52kD Ab|PrThr|Pt|Body fld
LG26777-9	Sjogrens syndrome-A extractable nuclear 52kD Ab|PrThr|Pt|CSF
LG26778-7	Sjogrens syndrome-A extractable nuclear 60kD Ab|PrThr|Pt|Body fld
LG26780-3	Sjogrens syndrome-A extractable nuclear Ab|PrThr|Pt|Body fld
LG29266-0	Sjogrens syndrome-B extractable nuclear Ab|PrThr|Pt|Body fld
LG29267-8	Sjogrens syndrome-B extractable nuclear Ab|PrThr|Pt|CSF
LG29303-1	Sjogrens syndrome-A extractable nuclear Ab|PrThr|Pt|CSF
LG5157-5	Sjogrens syndrome-A extractable nuclear Ab|PrThr|Pt|ANYBldSerPl
LG5158-3	Sjogrens syndrome-A extractable nuclear Ab|Acnc|Pt|ANYBldSerPl
LG5159-1	Sjogrens syndrome-A extractable nuclear Ab.IgG|PrThr|Pt|ANYBldSerPl
LG5160-9	Sjogrens syndrome-B extractable nuclear Ab|PrThr|Pt|ANYBldSerPl
LG5161-7	Sjogrens syndrome-B extractable nuclear Ab|Acnc|Pt|ANYBldSerPl
LG5162-5	Sjogrens syndrome-B extractable nuclear Ab.IgG|PrThr|Pt|ANYBldSerPl
LP113660-7	Sjogrens syndrome-A extractable nuclear Ab | Body fluid
LP113661-5	Sjogrens syndrome-B extractable nuclear Ab | Body fluid
LP113662-3	Sjogrens syndrome-B extractable nuclear Ab | Cerebral spinal fluid
LP113675-5	Sjogrens syndrome-A extractable nuclear 52kD Ab | Cerebral spinal fluid
LP113676-3	Sjogrens syndrome-A extractable nuclear 52kD Ab | Body fluid
LP113677-1	Sjogrens syndrome-A extractable nuclear 60kD Ab | Body fluid

LP113708-4	Sjogrens syndrome-A extractable nuclear Ab | Cerebral spinal fluid
LP116303-1	Phenx measure – systemic lupus erythematosus
LP146027-0	Sjogrens syndrome-A extractable nuclear 52kD IgG
LP146630-1	Sjogrens syndrome-A extractable nuclear 52kD IgG | Bld-Ser-Plas
LP16760-8	Sjogrens syndrome-A extractable nuclear
LP16761-6	Sjogrens syndrome-B extractable nuclear
LP256831-1	Sjogrens syndrome-A extractable nuclear | Bld-Ser-Plas
LP256832-9	Sjogrens syndrome-B extractable nuclear | Bld-Ser-Plas
LP257836-9	Sjogrens syndrome-A extractable nuclear Ab+Sjogrens syndrome-B extractable nuclear | Bld-Ser-Plas
LP259288-1	Sjogrens syndrome-A extractable nuclear 52kD | Bld-Ser-Plas
LP259289-9	Sjogrens syndrome-A extractable nuclear 60kD | Bld-Ser-Plas
LP259704-7	Sjogrens syndrome-A extractable nuclear 52kD | Body fluid
LP259705-4	Sjogrens syndrome-A extractable nuclear 52kD | Cerebral spinal fluid
LP259706-2	Sjogrens syndrome-A extractable nuclear 60kD | Body fluid
LP259707-0	Sjogrens syndrome-A extractable nuclear | Body fluid
LP259709-6	Sjogrens syndrome-B extractable nuclear | Body fluid
LP259710-4	Sjogrens syndrome-B extractable nuclear | Cerebral spinal fluid
LP259722-9	Sjogrens syndrome-A extractable nuclear | Cerebral spinal fluid
LP263204-2	Sjogrens syndrome-A and B extractable nuclear IgG panel
LP265606-6	Systemic sclerosis panel
LP28892-5	Sjogrens syndrome-A extractable nuclear Ab+Sjogrens syndrome-B extractable nuclear
LP36354-6	Sjogrens syndrome-A and B extractable nuclear Ab panel
LP39751-0	Sjogrens syndrome-A extractable nuclear Ab
LP39752-8	Sjogrens syndrome-A extractable Nuclear IgG
LP39753-6	Sjogrens syndrome-A extractable nuclear Ab+Sjogrens syndrome-B extractable nuclear Ab
LP39754-4	Sjogrens syndrome-B extractable nuclear Ab
LP39755-1	Sjogrens syndrome-B extractable nuclear IgG
LP403379-3	Sjogrens syndrome-A extractable nuclear Ab | Body fluid | Serology – non-micro
LP403380-1	Sjogrens syndrome-A extractable nuclear Ab | Cerebral spinal fluid | Serology – non-micro
LP403381-9	Sjogrens syndrome-A extractable nuclear Ab | Serum | Serology – non-micro
LP403382-7	Sjogrens syndrome-A extractable Nuclear IgG | Serum | Serology – non-micro
LP403383-5	Sjogrens syndrome-A extractable nuclear 52kD Ab | Body fluid | Serology – non-micro
LP403384-3	Sjogrens syndrome-A extractable nuclear 52kD Ab | Cerebral spinal fluid | Serology – non-micro
LP403385-0	Sjogrens syndrome-A extractable nuclear 52kD Ab | Serum | Serology – non-micro
LP403386-8	Sjogrens syndrome-A extractable nuclear 52kD IgG | Serum | Serology – non-micro
LP403387-6	Sjogrens syndrome-A extractable nuclear 60kD Ab | Body fluid | Serology – non-micro
LP403388-4	Sjogrens syndrome-A extractable nuclear 60kD Ab | Serum | Serology – non-micro
LP403389-2	Sjogrens syndrome-A extractable nuclear Ab+Sjogrens syndrome-B extractable nuclear Ab | Serum | Serology – non-micro
LP403390-0	Sjogrens syndrome-B extractable nuclear Ab | Body fluid | Serology – non-micro
LP403391-8	Sjogrens syndrome-B extractable nuclear Ab | Cerebral spinal fluid | Serology – non-micro
LP403392-6	Sjogrens syndrome-B extractable nuclear Ab | Serum | Serology – non-micro
LP403393-4	Sjogrens syndrome-B extractable nuclear IgG | Serum | Serology – non-micro
LP404125-9	Sjogrens syndrome-A and B extractable nuclear Ab panel | Serum | Serology Panels
LP404126-7	Sjogrens syndrome-A and B extractable nuclear IgG panel | Serum | Serology Panels
LP404128-3	Systemic sclerosis panel | Serum | Serology Panels
LP45489-9	Sjogrens Syndrome-a Extractable Nuclear Ab | Bld-Ser-Plas
LP45490-7	Sjogrens Syndrome-a Extractable Nuclear IgG | Bld-Ser-Plas
LP45491-5	Sjogrens Syndrome-b Extractable Nuclear Ab | Bld-Ser-Plas
LP45492-3	Sjogrens Syndrome-b Extractable Nuclear IgG | Bld-Ser-Plas
LP69659-8	Sjogrens syndrome-A Extractable Nuclear Ab+Sjogrens syndrome-B Extractable Nuclear Ab | Bld-Ser-Plas
LP69892-5	Sjogrens syndrome-A extractable nuclear 52kD Ab
LP69893-3	Sjogrens syndrome-A extractable nuclear 60kD Ab
LP69895-8	Sjogrens syndrome-A extractable nuclear 52kD
LP69896-6	Sjogrens syndrome-A extractable nuclear 60kD
LP69943-6	Sjogrens syndrome-A extractable nuclear 52kD Ab | Bld-Ser-Plas

LP69944-4	Sjogrens syndrome-A extractable nuclear 60kD Ab | Bld-Ser-Plas
M160.	Psoriatic arthropathy
M1600	Psoriasis spondylitica
M160z	Psoriatic arthropathy NOS
M35.0	Sjogren syndrome
M35.00	Sjogren syndrome, unspecified
M35.01	Sjogren syndrome with keratoconjunctivitis
M35.02	Sjogren syndrome with lung involvement
M35.03	Sjogren syndrome with myopathy
M35.04	Sjogren syndrome with 32ubule-interstitial nephropathy
M35.05	Sjogren syndrome with inflammatory arthritis
M35.06	Sjogren syndrome with peripheral nervous system involvement
M35.07	Sjogren syndrome with central nervous system involvement
M35.08	Sjogren syndrome with gastrointestinal involvement
M35.09	Sjogren syndrome with other organ involvement
M35.0A	Sjogren syndrome with glomerular disease
M35.0B	Sjogren syndrome with vasculitis
M35.0C	Sjogren syndrome with dental involvement
N….	Musculoskeletal &/or connective tissue diseases
N0450	Juvenile ankylosing spondylitis
N0451	Juvenile seronegative polyarthritis
N0452	Juvenile arthritis in psoriasis
N0453	Juvenile arthritis in Crohns disease
N0454	Juvenile arthritis in ulcerative colitis
N0456	Pauciarticular onset juvenile chronic arthritis
N04y.	Other specified inflammatory polyarthropathy
N04y1	Sero negative arthritis
N04y2	Adult-onset Stills disease
N04yz	Other specified inflammatory polyarthropathy NOS
N04z.	Inflammatory polyarthropathy NOS
N065.	Unspecified polyarthropathy or polyarthritis
N10..	Inflammatory spondylopathies
N20..	Polymyalgia rheumatica
N2314	Polymyositis ossificans
Ny…	Musculoskeletal or connective tissue diseases OS
Nyu15	[X]Other juvenile arthritis
Nyu4F	[X]Mixed connective tissue disease
Nz…	Musculoskeletal and connective tissue diseases NOS
X701x	Juvenile ankylosing spondylitis
X702I	Undifferentiated inflammatory polyarthritis
X705A	Primary Sjogren’s syndrome
X705B	Primary Sjogren’s syndrome with organ/system involvement
X705C	Primary Sjogren’s syndrome with multisystem involvement
X705D	Secondary Sjogren’s syndrome
X705E	Secondary Sjogren’s syndrome with organ/system involvement
X705F	Secondary Sjogren’s syndrome with multisystem involvement
X705G	Undifferentiated connective tissue disease
X705H	Mixed connective tissue disease
X70A0	Eosinophilic polymyositis
X7A2D	Sjogren hand technique
X80e1	Sjogren’s syndrome – A antibody
X80e2	Sjogren’s syndrome – B antibody
Xa0hF	Sjogren’s syndrome
Xa1jE	Systemic sclerosis
XaDmf	Musculoskeletal and connective tissue diseases

XaQk8	History of connective tissue disease
XaXlJ	Interstitial lung disease due to connective tissue disease
XE1DE	Connective tissue disease
XE1FJ	Polymyalgia rheumatica
XE2Qc	Unspecified polyarthropathy or polyarthritis

Supplementary Material E3

Supplementary Material E3: Read and SNOMED codes used to define steroid exposure

code ID	Code term	Code type
325448000	Prednisolone 2.5mg tablet	SNOMED
fe11.	Bentelan 500mcg tablet	Read
fe12.	Betnesol 500mcg soluble tablet	Read
fe1x.	Betamethasone 500micrograms soluble tablet	Read
fe1y.	Betamethasone 500micrograms tablet	Read
fe2..	Cortisone acetate product	Read
fe21.	Cortisone acetate 5mg tablet	Read
fe22.	Cortisone acetate 25mg tablet	Read
fe3..	DEXAMETHASONE [ENDOCRINE]	Read
fe31.	DEXAMETHASONE 500micrograms tablets	Read
fe32.	DEXAMETHASONE 2mg tablets	Read
fe33.	DECADRON 500micrograms tablets	Read
fe36.	*ORADEXON 500microgram tablets	Read
fe37.	*ORADEXON 2mg tablets	Read
fe3A.	DEXSOL 2mg/5mL oral solution	Read
fe3B.	DEXAMETHASONE 10mg/5mL oral solution	Read
fe3C.	MARTAPAN 2mg/5mL oral solution	Read
fe3r.	DEXAMETHASONE 500micrograms/5mL solution	Read
fe3s.	DEXAMETHASONE 2mg/5mL sugar free solution	Read
fe3u.	DEXAMETHASONE 2mg/5mL liquid	Read
fe4..	HYDROCORTISONE	Read
fe41.	HYDROCORTISONE 10mg tablets	Read
fe42.	HYDROCORTISONE 20mg tablets	Read
fe43.	*HYDROCORTISTAB 20mg tablets	Read
fe44.	*HYDROCORTONE 10mg tablets	Read
fe45.	*HYDROCORTONE 20mg tablets	Read
fe4e.	PLENADREN 5mg m/r tablets	Read
fe4f.	HYDROCORTISONE 5mg m/r tablets	Read
fe4g.	PLENADREN 20mg m/r tablets	Read
fe4h.	HYDROCORTISONE 20mg m/r tablets	Read
fe5..	METHYLPREDNISOLONE [ENDOCRINE]	Read
fe51.	MEDRONE 2mg tablets	Read
fe52.	MEDRONE 4mg tablets	Read
fe53.	MEDRONE 16mg tablets	Read
fe5f.	MEDRONE 100mg tablets	Read
fe5m.	METHYLPREDNISOLONE 100mg tablets	Read
fe5n.	METHYLPREDNISOLONE 2mg tablets	Read
fe5o.	METHYLPREDNISOLONE 4mg tablets	Read
fe5p.	METHYLPREDNISOLONE 16mg tablets	Read
fe6..	PREDNISOLONE [ENDOCRINE]	Read
fe61.	PREDNISOLONE 1mg tablets	Read
fe62.	PREDNISOLONE 5mg tablets	Read
fe64.	*DELTA-PHORICOL 5mg tablets	Read
fe65.	DELTACORTRIL ENTERIC 2.5mg tablets	Read
fe66.	DELTACORTRIL ENTERIC 5mg tablets	Read
fe67.	*DELTALONE 1mg tablets	Read
fe68.	*DELTALONE 5mg tablets	Read
fe69.	*DELTASTAB 1mg tablets	Read
fe6a.	*DELTASTAB 5mg tablets	Read
fe6c.	*PRECORTISYL 1mg tablets	Read
fe6d.	*PRECORTISYL 5mg tablets	Read
fe6e.	PRECORTISYL FORTE 25mg tablets	Read
fe6f.	*PREDNESOL 5mg tablets	Read
fe6g.	*SINTISONE 5mg tablets	Read
fe6h.	PREDNISOLONE 2.5mg e/c tablets	Read
fe6i.	PREDNISOLONE 5mg e/c tablets	Read
fe6j.	PREDNISOLONE 5mg soluble tablets	Read
fe6k.	PREDNISOLONE 50mg tablets	Read
fe6l.	DILACORT 5mg gastro-resistant tablets	Read
fe6m.	DILACORT 2.5mg gastro-resistant tablets	Read
fe6n.	PEVANTI 2.5mg tablets	Read
fe6o.	PEVANTI 25mg tablets	Read
fe6p.	PEVANTI 5mg tablets	Read
fe6q.	PEVANTI 10mg tablets	Read
fe6r.	PEVANTI 20mg tablets	Read
fe6s.	PREDNISOLONE 20mg tablets	Read
fe6t.	PREDNISOLONE 10mg tablets	Read
fe6v.	*PREDNISOLONE 2.5mg tablets	Read
fe6w.	*PREDNISOLONE 2.5mg tablets	Read
fe6z.	PREDNISOLONE 25mg tablets	Read
fe7..	PREDNISONE	Read
fe71.	*PREDNISONE 1mg tablets	Read
fe72.	*PREDNISONE 5mg tablets	Read
fe73.	*DECORTISYL 5mg tablets	Read
fe74.	*ECONOSONE 1mg tablets	Read
fe75.	*ECONOSONE 5mg tablets	Read
fe76.	Prednisone 20mg tablet	Read

fe77.	LODOTRA 2mg m/r tablets	Read
fe78.	LODOTRA 5mg m/r tablets	Read
fe79.	LODOTRA 1mg m/r tablets	Read
fe7x.	PREDNISONE 5mg m/r tablets	Read
fe7y.	PREDNISONE 2mg m/r tablets	Read
fe7z.	PREDNISONE 1mg m/r tablets	Read
fe81.	Triamcinolone 2mg tablet	Read
fe82.	Triamcinolone 4mg tablet	Read
fe9..	Deflazacort	Read
fe91.	Deflazacort 6mg tablet	Read
fe92.	Calcort 6mg tablet	Read
fe93.	Deflazacort 30mg tablet	Read
fe94.	Calcort 30mg tablet	Read
fe95.	Deflazacort 1mg tablet	Read
fe96.	Calcort 1mg tablet	Read
x00yP	Oral prednisolone	Read
x01Mh	Oral dexamethasone	Read
x01MW	Oral betamethasone	Read
x01Na	Oral hydrocortisone	Read
x01Nb	Oral methylprednisolone	Read
x01Nq	Oral triamcinolone	Read
x02iV	Medrone	Read
x02Vd	Betnelan	Read
x02Y5	Corlan	Read
x02Yv	Deltacortril	Read
x03jK	Calcort	Read
x049g	Hydrocortone	Read
x04AS	Deltacortril Enteric	Read
x0595	Dexsol	Read

Supplementary Material E4

Supplementary Material E4: Read and SNOMED codes used to define comorbidities

Code Type	Code term	Code ID
Ischaemic heart disease		
SNOMED	Endarterectomy of coronary artery NEC	5.38891E+14
SNOMED	Attends coronary heart disease monitoring	3.8541E+13
SNOMED	Acute ST segment elevation myocardial infarction of lateral wall (disorder)	1.57129E+16
SNOMED	Other specified chronic ischaemic heart disease	6.21611E+14
SNOMED	Non-obstructive atherosclerosis of coronary artery (disorder)	719678003
SNOMED	Acute myocardial infarction of basal-lateral wall (disorder)	282006
SNOMED	Coronary artery operations NOS	5.37011E+14
SNOMED	Acute ST segment elevation myocardial infarction of septum (disorder)	1.57132E+16
SNOMED	Acute myocardial infarction due to right coronary artery occlusion (disorder)	2.3311E+13
SNOMED	Acute Q wave myocardial infarction (disorder)	304914007
SNOMED	Mural thrombus of right ventricle following acute myocardial infarction (disorder)	736978009
SNOMED	Angina, class IV (disorder)	89323001
SNOMED	Attends coronary heart disease monitoring	4.1021E+13
SNOMED	Emergency percutaneous coronary intervention	8.90631E+14
SNOMED	Acute myocardial infarction of inferolateral wall (disorder)	65547006
SNOMED	Other acute and subacute ischaemic heart disease NOS	6.43891E+14
SNOMED	Coronary heart disease monitoring verbal invitation	3.8271E+13
SNOMED	Acute coronary artery occlusion not resulting in myocardial infarction (disorder)	7.8741E+13
SNOMED	Diabetes mellitus insulin-glucose infusion in acute myocardial infarction (procedure)	315287002
SNOMED	Mitral valve regurgitation due to acute myocardial infarction with papillary muscle and chordal rupture (disorder)	703330009
SNOMED	Acute ST segment elevation myocardial infarction	8.3361E+13
SNOMED	Coronary heart disease monitoring verbal invitation (finding)	401253006
SNOMED	Attends coronary heart disease monitoring	3.8751E+13
SNOMED	Angina control NOS	5.65751E+14
SNOMED	Percutaneous transluminal atherectomy of coronary artery	2.59481E+14
SNOMED	Subsequent non-ST segment elevation myocardial infarction (disorder)	703360004
SNOMED	Posterior myocardial infarction NOS	5.81531E+14
SNOMED	Myocardial ischemia (disorder)	414795007
SNOMED	Acute myocardial infarction of apical-lateral wall (disorder)	59063002
SNOMED	Angina self-management plan agreed	8.11911E+14
SNOMED	Septal infarction by electrocardiogram (finding)	1077002
SNOMED	Subsequent myocardial infarction of anterior wall (disorder)	194857001
SNOMED	Other specified allograft replacement of coronary artery	5.89941E+14
SNOMED	Syncope anginosa (disorder)	21470009
SNOMED	Ischaemic heart disease NOS	6.62031E+14
SNOMED	Other specified ischaemic heart disease	6.46451E+14
SNOMED	Coronary heart disease monitoring 3rd letter	4.5291E+13
SNOMED	Postoperative nontransmural myocardial infarction (disorder)	1.08943E+15
SNOMED	Myocardial ischemia during surgery (disorder)	1.0971E+13
SNOMED	Acute ST segment elevation myocardial infarction of anterior wall (disorder)	703164000
SNOMED	Coronary artery atheroma (disorder)	67682002
SNOMED	Recurrent angina status post coronary stent placement (disorder)	371809004
SNOMED	Coronary heart disease monitoring verbal invitation	4.5301E+13

SNOMED	Admit ischaemic heart disease emergency	3.20351E+14
SNOMED	Atherosclerosis of coronary artery (disorder)	443502000
SNOMED	Status anginosus (disorder)	19057007
SNOMED	Coronary heart disease monitoring 1st letter	3.8241E+13
SNOMED	Single anastomosis of mammary artery to coronary artery NEC	6.91621E+14
SNOMED	Progressive angina (disorder)	371806006
SNOMED	Subsequent myocardial infarction of other sites	6.23361E+14
SNOMED	Other autograft replacement of coronary artery	5.89871E+14
SNOMED	Other specified other open operation on coronary artery	5.62881E+14
SNOMED	Coronary arteriosclerosis in native artery (disorder)	1.641E+12
SNOMED	Acute ST segment elevation myocardial infarction involving left anterior descending coronary artery (disorder)	2.85981E+14
SNOMED	Coronary arteriosclerosis following coronary artery bypass graft (disorder)	1.39011E+14
SNOMED	Acute myocardial infarction of septum (disorder)	79009004
SNOMED	Postoperative myocardial infarction, unspecified	6.77131E+14
SNOMED	Percutaneous coronary intervention	8.41991E+14
SNOMED	Coronary heart disease monitoring 1st letter	4.1051E+13
SNOMED	Silent coronary vasospastic disease (disorder)	703214003
SNOMED	Acute ST segment elevation myocardial infarction of posterobasal wall (disorder)	1.57132E+16
SNOMED	Ventricular aneurysm due to and following acute myocardial infarction (disorder)	723858002
SNOMED	History of acute ST segment elevation myocardial infarction (situation)	2.85721E+14
SNOMED	Coronary heart disease monitoring third letter (finding)	401252001
SNOMED	Preinfarction syndrome (disorder)	4557003
SNOMED	Significant coronary bypass graft disease (disorder)	371805005
SNOMED	Lateral myocardial infarction NOS	6.03901E+14
SNOMED	Other chronic ischaemic heart disease NOS	6.53041E+14
SNOMED	History of placement of stent in coronary artery bypass graft (situation)	1.30541E+14
SNOMED	Acute myocardial infarction of high lateral wall (disorder)	64627002
SNOMED	Acute non-Q wave infarction (disorder)	307140009
SNOMED	Unstable angina co-occurrent and due to coronary arteriosclerosis (disorder)	1.59601E+16
SNOMED	Connection of other thoracic artery to coronary artery	5.81151E+14
SNOMED	Insertion of drug-eluting coronary artery stent	2.11371E+14
SNOMED	Anterior myocardial infarction NOS	5.81521E+14
SNOMED	Other specified prosthetic replacement of coronary artery	5.89961E+14
SNOMED	Chronic myocardial ischemia (disorder)	413844008
SNOMED	Coronary heart disease monitoring 2nd letter	3.8791E+13
SNOMED	Generalized ischemic myocardial dysfunction (disorder)	194849004
SNOMED	Angina pectoris NOS	6.21581E+14
SNOMED	Frequency of angina	7.83611E+14
SNOMED	Coronary heart disease monitoring verbal invitation	4.3441E+13
SNOMED	Coronary occlusion (disorder)	63739005
SNOMED	Inferior myocardial infarction NOS	6.03911E+14
SNOMED	History of myocardial infarction	7.07421E+14
SNOMED	Resting ischemia co-occurrent and due to ischemic heart disease (disorder)	712866001
SNOMED	Subsequent ST segment elevation myocardial infarction of anterior wall (disorder)	703210007
SNOMED	Acute ST segment elevation myocardial infarction of posterior wall (disorder)	1.5713E+16
SNOMED	Saphenous vein graft replacement coronary artery NOS	5.94421E+14
SNOMED	Acute myocardial infarction due to left coronary artery occlusion (disorder)	1.7531E+13
SNOMED	Arteriosclerosis of internal mammary artery coronary artery bypass graft (disorder)	444856008
SNOMED	Angina decubitus (disorder)	59021001
SNOMED	Acute coronary syndrome (disorder)	394659003
SNOMED	Acute Q wave infarction - lateral (disorder)	233833005
SNOMED	Single coronary vessel disease (disorder)	194842008

SNOMED	Coronary heart disease care plan	7.93911E+14
SNOMED	Coronary heart disease monitoring 3rd letter	3.8591E+13
SNOMED	Acute nontransmural myocardial infarction (disorder)	1.08945E+15
SNOMED	Coronary heart disease monitoring default	3.8231E+13
SNOMED	Other acute and subacute ischaemic heart disease	6.43861E+14
SNOMED	Acute infarction of papillary muscle (disorder)	10273003
SNOMED	Hibernating myocardium (disorder)	281093002
SNOMED	Angina (disorder)	194828000
SNOMED	Unstable angina co-occurrent and due to arteriosclerosis of coronary artery bypass graft (disorder)	1.59607E+16
SNOMED	Ischemic myocardial dysfunction (disorder)	281091000
SNOMED	Acute myocardial infarction (disorder)	57054005
SNOMED	Old myocardial infarction (disorder)	1755008
SNOMED	History of myocardial infarction (situation)	399211009
SNOMED	Coronary microvascular disease	8.31761E+14
SNOMED	Coronary arteriosclerosis after percutaneous coronary angioplasty (disorder)	1.10187E+16
SNOMED	Pericarditis due to acute myocardial infarction (disorder)	71023004
SNOMED	Percutaneous coronary intervention	8.41981E+14
SNOMED	Other specified operations on coronary artery	5.82911E+14
SNOMED	Replacement of coronary artery NOS	5.36991E+14
SNOMED	ECG: myocardial infarct NOS	5.78421E+14
SNOMED	Acute myocardial infarction of inferior wall (disorder)	73795002
SNOMED	Ischemic dilated cardiomyopathy due to coronary artery disease (disorder)	472100003
SNOMED	Arteriosclerosis of coronary artery bypass graft (disorder)	429673002
SNOMED	Coronary heart disease monitoring default	3.8561E+13
SNOMED	Acute ST segment elevation myocardial infarction due to right coronary artery occlusion (disorder)	1.57131E+16
SNOMED	Subsequent ST segment elevation myocardial infarction (disorder)	703211006
SNOMED	Acute myocardial infarction of anterior wall (disorder)	54329005
SNOMED	Coronary artery bypass graft operation planned (situation)	698378009
SNOMED	Atrial septal defect due to and following acute myocardial infarction (disorder)	194863005
SNOMED	Autograft replacement of four of more coronary arteries NEC	5.89921E+14
SNOMED	Repair of coronary artery NOS	5.55721E+14
SNOMED	History of myocardial infarction in last year (situation)	308065005
SNOMED	Acute non-Q wave infarction - anterolateral (disorder)	233828006
SNOMED	Subsequent ST segment elevation myocardial infarction of inferior wall (disorder)	703209002
SNOMED	Other specified replacement of coronary artery	5.36981E+14
SNOMED	Referral to Angina Plan self-management programme declined	8.53601E+14
SNOMED	Other specified repair of coronary artery	5.32881E+14
SNOMED	Acute anteroapical myocardial infarction (disorder)	52035003
SNOMED	History of myocardial infarction	5.12751E+14
SNOMED	Coronary heart disease monitoring check done	4.3461E+13
SNOMED	Other specified anterior myocardial infarction	5.81511E+14
SNOMED	[V]Presence of aortocoronary bypass graft	4.69031E+14
SNOMED	Coronary heart disease monitoring verbal invitation	3.8601E+13
SNOMED	Referral to Angina Plan self-management programme	8.53561E+14
SNOMED	Acute non-ST segment elevation myocardial infarction	4.6001E+13
SNOMED	Coronary heart disease monitoring 1st letter	4.5271E+13
SNOMED	History of myocardial infarction in last eight weeks (situation)	4.61E+11
SNOMED	Postoperative subendocardial myocardial infarction (disorder)	311796008
SNOMED	Postmyocardial infarction syndrome (disorder)	66189004
SNOMED	Angina self-management plan review	8.11701E+14
SNOMED	Coronary heart disease monitoring 2nd letter	3.8581E+13
SNOMED	Coronary heart disease monitoring 2nd letter	4.5281E+13

SNOMED	Mechanical complication due to coronary bypass graft (disorder)	78717006
SNOMED	Old inferior myocardial infarction (disorder)	233840006
SNOMED	Deep venous thrombosis associated with coronary artery bypass graft (disorder)	428781001
SNOMED	Mitral valve regurgitation due to and following acute myocardial infarction (disorder)	703326006
SNOMED	Arteriosclerosis of coronary artery bypass graft of transplanted heart (disorder)	444855007
SNOMED	Coronary artery bypass graft occlusion	1.04351E+14
SNOMED	Other therapeutic transluminal operations on coronary artery	5.86371E+14
SNOMED	Coronary angioplasty planned (situation)	7.52181E+14
SNOMED	Postoperative transmural myocardial infarction of other sites	6.77111E+14
SNOMED	Acute ST segment elevation myocardial infarction	4.4011E+13
SNOMED	Acute myocardial infarction with rupture of ventricle (disorder)	30277009
SNOMED	Prosthetic replacement of coronary artery NOS	5.83961E+14
SNOMED	Acute myocardial infarction NOS	6.23341E+14
SNOMED	Acute Q wave infarction - anteroseptal (disorder)	233825009
SNOMED	Coronary heart disease monitoring 2nd letter	4.3421E+13
SNOMED	Acute ST segment elevation myocardial infarction of inferolateral wall (disorder)	1.22381E+16
SNOMED	Other replacement of coronary artery	5.36971E+14
SNOMED	Old lateral myocardial infarction (disorder)	233841005
SNOMED	Coronary artery bypass graft operation planned (situation)	7.09791E+14
SNOMED	Disorder of coronary artery (disorder)	414024009
SNOMED	Acute ST segment elevation myocardial infarction of anterolateral wall (disorder)	1.57129E+16
SNOMED	Coronary heart disease monitoring verbal invitation	4.1081E+13
SNOMED	Postoperative transmural myocardial infarction of inferior wall (disorder)	311793000
SNOMED	Postoperative transmural myocardial infarction of unspecified site	6.77121E+14
SNOMED	Asymptomatic coronary heart disease (disorder)	315348000
SNOMED	Arteriosclerosis of arterial coronary artery bypass graft (disorder)	442421004
SNOMED	Acute Q wave infarction - inferolateral (disorder)	233831007
SNOMED	Ischemic chest pain (finding)	225566008
SNOMED	Recurrent angina status post coronary artery bypass graft (disorder)	371810009
SNOMED	Obliterative coronary artery disease (disorder)	420006002
SNOMED	Acute ST segment elevation myocardial infarction of anterior wall involving right ventricle (disorder)	703165004
SNOMED	Postoperative transmural myocardial infarction (disorder)	1.08944E+15
SNOMED	Post infarct angina (disorder)	314116003
SNOMED	Postoperative transmural myocardial infarction of anterior wall (disorder)	311792005
SNOMED	Coronary heart disease monitoring 3rd letter	3.8261E+13
SNOMED	Thrombosis of atrium, auricular appendage, and ventricle due to and following acute myocardial infarction (disorder)	194868001
SNOMED	Coronary heart disease monitoring 1st letter	4.3411E+13
SNOMED	Post-infarction ventricular septal defect (disorder)	233846000
SNOMED	Acute myocardial infarction of inferior wall involving right ventricle (disorder)	703251009
SNOMED	Attends coronary heart disease monitoring	3.8211E+13
SNOMED	Other autograft replacement of coronary artery NOS	5.95941E+14
SNOMED	Acute atrial infarction (disorder)	194809007
SNOMED	Arteriosclerosis of nonautologous coronary artery bypass graft (disorder)	442240008
SNOMED	Acute non-Q wave infarction - anteroseptal (disorder)	233826005
SNOMED	Coronary heart disease monitoring default	4.1041E+13
SNOMED	Atherosclerosis of non-autologous coronary artery bypass graft (disorder)	723862008
SNOMED	Acute ST segment elevation myocardial infarction involving left main coronary artery (disorder)	2.85991E+14
SNOMED	Other bypass of coronary artery NOS	5.82901E+14
SNOMED	Acute ST segment elevation myocardial infarction (disorder)	401303003
SNOMED	Mural thrombus of left ventricle following acute myocardial infarction (disorder)	1.5961E+16
SNOMED	Acute ST segment elevation myocardial infarction of inferior wall (disorder)	703213009
SNOMED	Coronary heart disease monitoring second letter (finding)	401251008

SNOMED	Other acute myocardial infarction NOS	5.83001E+14
SNOMED	Allograft replacement of coronary artery NOS	5.89951E+14
SNOMED	Acute non-ST segment elevation myocardial infarction	4.4001E+13
SNOMED	Repair of coronary artery NEC	5.38881E+14
SNOMED	Cardiogenic shock unrelated to mechanical complications as current complication following acute myocardial infarction (disorder)	723861001
SNOMED	Acute myocardial infarction of lateral wall (disorder)	58612006
SNOMED	Autograft replacement of three coronary arteries NEC	5.89911E+14
SNOMED	Frequency of angina	7.83601E+14
SNOMED	Angina, class II (disorder)	41334000
SNOMED	Angina self-management plan review	8.11711E+14
SNOMED	Recurrent angina status post directional coronary atherectomy (disorder)	371812001
SNOMED	Recent myocardial infarction (situation)	428752002
SNOMED	Left main coronary artery disease (disorder)	371804009
SNOMED	Right coronary artery occlusion (disorder)	123642008
SNOMED	Coronary heart disease monitoring check done	3.8621E+13
SNOMED	Acute myocardial infarction of posterobasal wall (disorder)	70998009
SNOMED	Angina associated with type II diabetes mellitus (disorder)	7.91E+11
SNOMED	Ischemic contracture of left ventricle syndrome (disorder)	82522008
SNOMED	Coronary heart disease monitoring 1st letter	3.8571E+13
SNOMED	Coronary angioplasty planned (situation)	698377004
SNOMED	Rupture of cardiac wall without hemopericardium as current complication following acute myocardial infarction (disorder)	194865003
SNOMED	Mitral valve regurgitation due to acute myocardial infarction without papillary muscle and chordal rupture (disorder)	703328007
SNOMED	Acute myocardial infarction of inferoposterior wall (disorder)	76593002
SNOMED	Quality and Outcomes Framework secondary prevention of coronary heart disease quality indicator-related care invitation (procedure)	1.11088E+15
SNOMED	Angina, class III (disorder)	85284003
SNOMED	Chronic total occlusion of coronary artery (disorder)	1.17051E+14
SNOMED	Subsequent myocardial infarction of inferior wall (disorder)	194858006
SNOMED	Coronary artery stenosis (disorder)	233970002
SNOMED	Rupture of papillary muscle as current complication following acute myocardial infarction (disorder)	194867006
SNOMED	Acute ST segment elevation myocardial infarction of inferior wall involving right ventricle (disorder)	703253007
SNOMED	Angina decubitus NOS	6.21571E+14
SNOMED	Other open operation on coronary artery NOS	5.69961E+14
SNOMED	Acute myocardial infarction of anterolateral wall (disorder)	70211005
SNOMED	Certain current complications following acute myocardial infarction (disorder)	194861007
SNOMED	Rupture of chordae tendinae due to and following acute myocardial infarction (disorder)	194866002
SNOMED	Referral to Angina Plan self-management programme	8.53571E+14
SNOMED	Ischemic heart disease (disorder)	414545008
SNOMED	Preinfarction syndrome NOS	6.43871E+14
SNOMED	Autograft replacement of two coronary arteries NEC	5.89901E+14
SNOMED	Nocturnal angina (disorder)	35928006
SNOMED	Past myocardial infarction diagnosed on electrocardiogram AND/OR other special investigation, but currently presenting no symptoms (disorder)	32574007
SNOMED	True posterior myocardial infarction (disorder)	194802003
SNOMED	Referral to Angina Plan self-management programme declined	8.53591E+14
SNOMED	Acute coronary insufficiency (disorder)	194823009
SNOMED	Subendocardial ischemia (disorder)	46109009
SNOMED	Impending infarction (disorder)	25106000
SNOMED	Coronary heart disease monitoring 1st letter	3.8781E+13
SNOMED	Acute ischemic heart disease (disorder)	413439005
SNOMED	Double coronary vessel disease (disorder)	194843003
SNOMED	Coronary heart disease monitoring 1st letter (finding)	401250009
SNOMED	Non-Q wave myocardial infarction (disorder)	314207007
SNOMED	Other specified chronic ischaemic heart disease NOS	6.53031E+14

SNOMED	Transluminal balloon angioplasty of coronary artery NOS	5.86361E+14
SNOMED	Coronary heart disease monitoring 2nd letter	3.8251E+13
SNOMED	Coronary heart disease monitoring 3rd letter	4.1071E+13
SNOMED	Coronary heart disease monitoring check done	3.8291E+13
SNOMED	Acute/subacute ischaemic heart disease NOS	6.71571E+14
SNOMED	Coronary arteriosclerosis in patient with history of previous myocardial infarction (situation)	1.03011E+14
SNOMED	Acute ST segment elevation myocardial infarction	4.5881E+13
SNOMED	Coronary microvascular disease (disorder)	8.10681E+14
SNOMED	Coronary heart disease monitoring verbal invitation	3.8811E+13
SNOMED	Accelerated coronary artery disease in transplanted heart (disorder)	233844002
SNOMED	Attends coronary heart disease monitoring	4.5241E+13
SNOMED	Other chronic ischaemic heart disease	6.21601E+14
SNOMED	Acute Q wave infarction - inferior (disorder)	233829003
SNOMED	Arteriosclerosis of autologous coronary artery bypass graft (disorder)	2.85151E+14
SNOMED	Coronary heart disease monitoring 3rd letter	4.3431E+13
SNOMED	[V]Presence of coronary artery bypass graft	4.00941E+14
SNOMED	Exercise-induced angina (disorder)	300995000
SNOMED	Stunned myocardium (disorder)	281092007
SNOMED	Acute ST segment elevation myocardial infarction due to left coronary artery occlusion (disorder)	1.57131E+16
SNOMED	Acute non-ST segment elevation myocardial infarction (disorder)	401314000
SNOMED	Angina self-management plan agreed	8.11901E+14
SNOMED	Coronary heart disease monitoring telephone invite	2.51581E+14
SNOMED	Recurrent coronary arteriosclerosis after percutaneous transluminal coronary angioplasty (disorder)	429245005
SNOMED	Angina co-occurrent and due to arteriosclerosis of coronary artery bypass graft (disorder)	1.59604E+16
SNOMED	Coronary heart disease monitoring telephone invitation (regime/therapy)	2.48461E+14
SNOMED	[V]Presence of coronary artery bypass graft	4.69611E+14
SNOMED	Cardiac rupture due to and following acute myocardial infarction (disorder)	233847009
SNOMED	Coronary heart disease monitoring default	3.8771E+13
SNOMED	Coronary artery thrombosis (disorder)	398274000
SNOMED	Silent myocardial infarction (disorder)	233843008
SNOMED	Microinfarct of heart (disorder)	42531007
SNOMED	Coronary heart disease risk clinical management plan	1.17021E+14
SNOMED	Acute subendocardial infarction (disorder)	70422006
SNOMED	Old anterior myocardial infarction (disorder)	233839009
SNOMED	Acute posterior myocardial infarction (disorder)	233838001
SNOMED	Acute transmural myocardial infarction (disorder)	1.08947E+15
SNOMED	Emergency percutaneous coronary intervention	8.81331E+14
SNOMED	Acute widespread myocardial infarction (disorder)	233835003
SNOMED	Coronary heart disease monitoring check done	4.1101E+13
SNOMED	Attends coronary heart disease monitoring	4.3381E+13
SNOMED	Acute non-Q wave infarction - inferolateral (disorder)	233832000
SNOMED	Coronary arteriosclerosis caused by radiation (disorder)	427919004
SNOMED	Left coronary artery occlusion (disorder)	123641001
SNOMED	Ischemic congestive cardiomyopathy (disorder)	426856002
SNOMED	Autograft replacement of one coronary artery NEC	5.89881E+14
SNOMED	Acute Q wave infarction - anterolateral (disorder)	233827001
SNOMED	Myocardial infarction with complication (disorder)	371068009
SNOMED	Insertion of drug-eluting coronary artery stent	2.03741E+14
SNOMED	Recurrent angina status post rotational atherectomy (disorder)	371811008
SNOMED	Chronic ischaemic heart disease NOS	6.71581E+14
SNOMED	Stable angina (disorder)	233819005
SNOMED	Coronary artery bypass graft occlusion (disorder)	408546009

SNOMED	Coronary heart disease monitoring check done	4.5321E+13
SNOMED	Silent myocardial ischemia (disorder)	233823002
SNOMED	Coronary heart disease monitoring telephone invite	2.74561E+14
SNOMED	Coronary heart disease monitoring default	4.3401E+13
SNOMED	History of myocardial infarct at age greater than sixty (situation)	161503005
SNOMED	Microvascular ischemia of myocardium (disorder)	697976003
SNOMED	Subacute ischemic heart disease (disorder)	713405002
SNOMED	Congenital coronary artery sclerosis (disorder)	42866003
SNOMED	Cardiac syndrome X	2.12721E+14
SNOMED	Postoperative myocardial infarction (disorder)	129574000
SNOMED	Typical angina (disorder)	429559004
SNOMED	Single anastomosis of thoracic artery to coronary artery NEC	5.63331E+14
SNOMED	Other specified revision of bypass for coronary artery	5.95931E+14
SNOMED	Hemopericardium due to and following acute myocardial infarction (disorder)	194862000
SNOMED	Acute anteroseptal myocardial infarction (disorder)	62695002
SNOMED	Coronary artery operations	31413008
SNOMED	Aortocoronary artery bypass graft repeated (situation)	61236006
SNOMED	Acute myocardial infarction of anterior wall involving right ventricle (disorder)	703252002
SNOMED	Myocardial infarction (disorder)	22298006
SNOMED	Pulmonary embolism due to and following acute myocardial infarction (disorder)	723859005
SNOMED	Other specified other bypass of coronary artery	5.85651E+14
SNOMED	Acute non-Q wave infarction - lateral (disorder)	233834004
SNOMED	Other acute myocardial infarction	5.82991E+14
SNOMED	Revision of bypass for coronary artery NOS	5.40201E+14
SNOMED	Acute coronary syndrome	1.76331E+14
SNOMED	Percutaneous transluminal atherectomy of coronary artery	232726007
SNOMED	New myocardial infarction compared to prior study (finding)	429391004
SNOMED	Connection of other thoracic artery to coronary artery NOS	5.96331E+14
SNOMED	Acute myocardial infarction during procedure (disorder)	703212004
SNOMED	Coronary heart disease monitoring check done	3.8831E+13
SNOMED	Angina co-occurrent and due to arteriosclerosis of autologous vein coronary artery bypass graft (disorder)	1.59606E+16
SNOMED	Coronary heart disease monitoring default	4.5261E+13
SNOMED	Connection of mammary artery to coronary artery NOS	5.88811E+14
SNOMED	History of myocardial infarct at age less than sixty (situation)	161502000
SNOMED	Multi vessel coronary artery disease (disorder)	371803003
SNOMED	Calcific coronary arteriosclerosis (disorder)	92517006
SNOMED	Angina, class I (disorder)	61490001
SNOMED	Acute ST segment elevation myocardial infarction of anteroseptal wall (disorder)	1.5713E+16
SNOMED	Subsequent myocardial infarction (disorder)	194856005
SNOMED	Chronic ischemic heart disease (disorder)	413838009
SNOMED	Coronary heart disease monitoring 2nd letter	4.1061E+13
SNOMED	Coronary heart disease monitoring invitation email (procedure)	1.08313E+15
SNOMED	Other bypass of coronary artery	5.37001E+14
SNOMED	Coronary spasm	8.6241E+13
SNOMED	Acute deep venous thrombosis of lower limb due to coronary artery bypass grafting (disorder)	1.32111E+14
SNOMED	Mixed myocardial ischemia and infarction (disorder)	428196007
SNOMED	Angina co-occurrent and due to coronary arteriosclerosis (disorder)	1.59601E+16
SNOMED	History of coronary artery bypass grafting (situation)	399261000
SNOMED	Atypical angina (disorder)	371807002
SNOMED	Acute ST segment elevation myocardial infarction of posterolateral wall (disorder)	1.57128E+16
SNOMED	Coronary graft stenosis (disorder)	251024009
SNOMED	Arteriosclerosis of autologous vein coronary artery bypass graft (disorder)	442224005

SNOMED	Triple vessel disease of the heart (disorder)	233817007
SNOMED	Coronary heart disease care plan	7.93921E+14
SNOMED	Quality and Outcomes Framework secondary prevention of coronary heart disease quality indicator-related care invitation using preferred method of communication (procedure)	1.43451E+14
SNOMED	History of non-ST segment elevation myocardial infarction (situation)	698593009
SNOMED	Acute myocardial ischemia (disorder)	413444003
SNOMED	Mechanical breakdown of coronary artery bypass graft (disorder)	2.85951E+14
SNOMED	Other open operations on coronary artery	5.91161E+14
SNOMED	Acute non-Q wave infarction - widespread (disorder)	233837006
SNOMED	Arteriosclerosis of autologous arterial coronary artery bypass graft (disorder)	2.85141E+14
SNOMED	Acute myocardial infarction of posterolateral wall (disorder)	15990001
SNOMED	Coronary heart disease monitoring invitation (procedure)	1.08312E+15
SNOMED	Arrhythmia due to and following acute myocardial infarction (disorder)	723860000
SNOMED	Acute posterolateral myocardial infarction	1.22701E+14
SNOMED	Acute ST segment elevation myocardial infarction of inferoposterior wall (disorder)	1.22382E+16
SNOMED	Myocardial infarction in recovery phase (disorder)	418044006
SNOMED	Coronary heart disease risk clinical management plan	1.21761E+14
SNOMED	Acute non-ST segment elevation myocardial infarction	8.3351E+13
SNOMED	[V]Presence of coronary angioplasty implant and graft	4.53291E+14
SNOMED	New onset angina (disorder)	233821000
SNOMED	Quality and Outcomes Framework quality indicator-related care invitation (procedure)	1.10992E+15
SNOMED	Coronary heart disease monitoring 3rd letter	3.8801E+13
SNOMED	Refractory angina (disorder)	315025001
SNOMED	Acute Q wave infarction - widespread (disorder)	233836002
SNOMED	Acute non-Q wave infarction - inferior (disorder)	233830008
SNOMED	Old posterior myocardial infarction (disorder)	233842003
SNOMED	Coronary arteriosclerosis (disorder)	53741008
SNOMED	First myocardial infarction (disorder)	394710008
SNOMED	Coronary thrombosis not resulting in myocardial infarction (disorder)	194821006
SNOMED	Chronic deep venous thrombosis of lower limb due to coronary artery bypass grafting (disorder)	1.32091E+14
SNOMED	Recurrent angina status post percutaneous transluminal coronary angioplasty (disorder)	371808007
SNOMED	Transient myocardial ischemia (disorder)	315026000
Read	Transluminal operations internal mammary artery side branch	793K.
Read	Revascularisation of wall of heart	790H3
Read	[V]Presence of coronary angioplasty implant and graft	ZV458
Read	Referral to Angina Plan self-management programme	8T04.
Read	Transluminal operations internal mammary art side branch NOS	XaLgm
Read	PERCUT TRANSLUMINAL BALLOON ANGIOPLASTY ONE CORONARY ARTERY	79280
Read	Heart disease: [arteriosclerotic] or [chronic ischaemic NOS]	XE0WE
Read	Myocardial infarction (& [acute]) or coronary thrombosis	XE0WA
Read	Coronary heart disease review	6A4..
Read	Angina pectoris NOS	G33z.
Read	Angina self-management plan agreed	XaYb7
Read	Chronic myocardial ischaemia	G34y1
Read	Coronary heart disease risk clinical management plan	8CR6.
Read	Acute myocardial infarction	XE0Uh
Read	Exc myocar infarction quality indicators: patient unsuitable	XaRFP
Read	OTHER SPECIFIED OTHER OPEN OPERATION ON CORONARY ARTERY	7927y
Read	Coronary heart disease care plan	8CMP.
Read	Repair of coronary artery NOS	792Bz
Read	Acute Q wave infarction - inferolateral	X200N
Read	Perc translumin balloon angioplasty stenting coronary artery	XaLgU

Read	OS transluminal operations internal mammary art side branch	XaLgl
Read	Acute anterior myocardial infarction	Xa0YL
Read	Ischaemic myocardial dysfunction	Xa1dP
Read	H/O: angina pectoris	14A5.
Read	SINGLE ANASTOMOSIS OF MAMMARY ARTERY TO CORONARY ARTERY NEC	79253
Read	OTHER REPLACEMENT OF CORONARY ARTERY	792C.
Read	Coronary heart disease monitoring 3rd letter	XaIvM
Read	New onset angina	X200A
Read	Acute widespread myocardial infarction	X200S
Read	Postoperative myocardial infarction, unspecified	G38z.
Read	Acute non-ST segment elevation myocardial infarction	G3071
Read	REVISION OF BYPASS FOR CORONARY ARTERY NOS	7924z
Read	REVISION OF BYPASS FOR THREE CORONARY ARTERIES	79242
Read	TRANSLUMINAL BALLOON ANGIOPLASTY OF CORONARY ARTERY NOS	7928z
Read	Acute Q wave infarction - widespread	X200T
Read	Coronary heart disease monitoring administration	XaIQp
Read	Exception reporting: myocardial infarction quality indicator	XaRFL
Read	Postoperative transmural myocardial infarction of anterior wall	XaD2d
Read	Antianginal therapy	XaBLr
Read	Exc myocard infarction quality indicators: informed dissent	9hM0.
Read	Perc translum balloon angioplasty insert 1-2 stents cor art	793G2
Read	Acute Q wave infarction - anteroseptal	X200G
Read	Coronary heart disease monitoring 2nd letter	9Ob4.
Read	DOUBLE ANASTOM THORACIC ARTERIES TO CORONARY ARTERIES NEC	79260
Read	Old posterior myocardial infarction	X200Z
Read	Refractory angina	XaFsG
Read	Coronary heart disease monitoring default	9Ob2.
Read	Worsening angina	G3114
Read	Old lateral myocardial infarction	X200Y
Read	Angina self-management plan review	XaYae
Read	Silent myocardial infarction	X200a
Read	Acute Q wave infarction - inferior	X200L
Read	Silent myocardial ischaemia	X200D
Read	Coronary heart disease monitoring check done	XaIvP
Read	Coronary spasm	X200B
Read	Acute non-Q wave infarction - inferolateral	X200O
Read	Triple vessel disease of the heart	X2006
Read	SINGLE ANAST MAMMARY ART TO LEFT ANT DESCEND CORONARY ART	79252
Read	Acute transmural myocardial infarction of unspecified site	Gyu34
Read	Open angioplasty of coronary artery	79275
Read	Acute posterior myocardial infarction	X200V
Read	Subsequent myocardial infarction of other sites	Gyu35
Read	OTHER SPECIFIED REPLACEMENT OF CORONARY ARTERY	792Cy
Read	Ischaemic heart diseases	Gyu3.
Read	Coronary heart disease monitoring administration	9Ob..
Read	Ventric septal defect/curr comp fol acut myocardal infarctn	G362.
Read	Coronary heart disease monitoring 1st letter	9Ob3.
Read	Percutaneous coronary intervention	XaZSy
Read	Acute inferoposterior infarction	G303.
Read	Other specified chronic ischaemic heart disease NOS	G34yz
Read	Postoperative transmural myocardial infarction of anterior wall	G380.
Read	Acute non-Q wave infarction	XaAzi

Read	OTHER SPECIFIED OTHER BYPASS OF CORONARY ARTERY	792Dy
Read	Single anastomosis of mammary artery to coronary artery NEC	XE2Px
Read	Acute myocardial infarction NOS	G30z.
Read	Ischaemic heart disease NOS	G3z..
Read	Atrial septal defect/curr comp folow acut myocardal infarct	G361.
Read	Percutaneous cor balloon angioplasty 3 more stents cor art NEC	793G3
Read	SAPHENOUS VEIN GRAFT REPLACEMENT OF FOUR+ CORONARY ARTERIES	79203
Read	CONNECTION OF MAMMARY ARTERY TO CORONARY ARTERY	7925
Read	Acute Q wave infarction - anterolateral	X200I
Read	Postoperative subendocardial myocardial infarction	XaD2h
Read	Postoperative transmural myocardial infarction of inferior wall	G381.
Read	Other specified chronic ischaemic heart disease	G34y.
Read	Single coronary vessel disease	G3400
Read	PERCUT TRANSLUM CUTTING BALLOON ANGIOPLASTY CORONARY ARTERY	79283
Read	Certain current complication follow acute myocardial infarct	G36..
Read	Cardiac syndrome X	G37..
Read	Coronary heart disease care plan	XaY6p
Read	H/O: myocardial infarct <60	14A3.
Read	Angina on effort	G33z3
Read	Posterior myocardial infarction NOS	G304.
Read	Referral to Angina Plan self-management programme	XaZlp
Read	ALLOGRAFT REPLACEMENT OF CORONARY ARTERY NOS	7922z
Read	Non-Q wave myocardial infarction	XaEgZ
Read	Nocturnal angina	G3300
Read	Post infarct angina	G33z5
Read	Angina pectoris	G33..
Read	Acute lateral myocardial infarction	X200P
Read	Acute non-Q wave infarction - anterolateral	X200J
Read	Coronary artery operations	792..
Read	OTHER AUTOGRAFT REPLACEMENT OF CORONARY ARTERY NOS	7921z
Read	Perc translum balloon angioplasty stenting coronary art NOS	XaLga
Read	Other acute myocardial infarction	G30y.
Read	Coronary heart disease monitoring verbal invitation	9Ob6.
Read	Angina at rest	G3112
Read	Myocardial infarction aborted	G3110
Read	Preinfarction syndrome	G311.
Read	OTHER SPECIFIED PROSTHETIC REPLACEMENT OF CORONARY ARTERY	7923y
Read	Acute septal infarction	G30y2
Read	Other acute and subacute ischaemic heart disease	G31y.
Read	AUTOGRAFT REPLACEMENT OF FOUR OF MORE CORONARY ARTERIES NEC	79213
Read	ALLOGRAFT REPLACEMENT OF TWO CORONARY ARTERIES	79221
Read	Acute posterolateral myocardial infarction	G30B.
Read	Postoperative transmural myocardial infarction of inferior wall	XaD2e
Read	Acute Q-wave infarct	G309.
Read	Acute coronary syndrome	XaINF
Read	REPLACEMENT OF CORONARY ARTERY NOS	792Cz
Read	Angina control - good	662K0
Read	SINGLE ANASTOMOSIS OF THORACIC ARTERY TO CORONARY ARTERY NEC	79262
Read	Exception reporting: myocardial infarction quality indicator	9hM..
Read	Coronary artery atheroma	XSDT6
Read	ECG: myocardial infarction	323..
Read	Acute anteroseptal infarction	G3011

Read	Post infarct angina	XaEXt
Read	OTHER SPECIFIED REVISION OF BYPASS FOR CORONARY ARTERY	7924y
Read	Stable angina	G33z7
Read	Coronary heart disease monitoring check done	9Ob8.
Read	Angina self-management plan agreed	661M0
Read	Coronary heart disease review	XaIOW
Read	OTHER THERAPEUTIC TRANSLUMINAL OPERATIONS ON CORONARY ARTERY	7929
Read	Ischaemic chest pain	G33z4
Read	Post infarction pericarditis	G501.
Read	First myocardial infarction	XaIf1
Read	Postoperative transmural myocardial infarction of other sites	XaD2f
Read	Transluminal operations internal mammary art side branch NOS	793Kz
Read	Ruptur cardiac wall without haemopericard/cur comp fol ac MI	G363.
Read	DOUBLE IMPLANT THORACIC ARTERIES INTO CORONARY ARTERIES NEC	79261
Read	Status anginosus	G33z0
Read	Coronary artery bypass graft occlusion	SP076
Read	Cardiac rupture after acute myocardial infarction	X200e
Read	Other specified anterior myocardial infarction	G301.
Read	Post-infarction ventricular septal defect	X200d
Read	Subsequent myocardial infarction of inferior wall	G351.
Read	Postoperative myocardial infarction	G38..
Read	Angina control NOS	662Kz
Read	Coronary heart disease medication review	XaIfL
Read	Coronary artery bypass graft occlusion	XaJIU
Read	OS transluminal operations internal mammary art side branch	793Ky
Read	Transluminal operations internal mammary artery side branch	XaLgj
Read	Perc translum balloon angioplasty ins 3 or more drug elut stents cor art	793G1
Read	OTHER SPECIFIED OPERATIONS ON CORONARY ARTERY	792y.
Read	Acute anteroapical infarction	G3010
Read	OTHER BYPASS OF CORONARY ARTERY	792D.
Read	Coronary atherosclerosis	XM0rN
Read	Acute non-Q wave infarction - widespread	X200U
Read	PERCUT TRANSLUM BALLOON ANGIOPLASTY BYPASS GRAFT CORONARY A	79282
Read	Subsequent myocardial infarction of unspecified site	G35X.
Read	Transluminal occlusion left internal mammary artery side branch	793K0
Read	Prinzmetals angina	G331.
Read	AUTOGRAFT REPLACEMENT OF ONE CORONARY ARTERY NEC	79210
Read	Thrombosis atrium,auric append&vent/curr comp foll acute MI	G366.
Read	Subendocardial ischaemia	G31y2
Read	Myocardial infarction	X200E
Read	[V]Presence of coronary artery bypass graft	ZV45K
Read	Angina decubitus	G330.
Read	Admit ischaemic heart disease emergency	XaNxN
Read	Asymptomatic coronary heart disease	G34z0
Read	OTHER THERAPEUTIC TRANSLUMINAL OP ON CORONARY ARTERY NOS	7929z
Read	H/O: angina in last year	14AJ.
Read	Double coronary vessel disease	G3401
Read	SAPHENOUS VEIN GRAFT REPLACEMENT OF CORONARY ARTERY	7920
Read	Asymptomatic coronary heart disease	XaG1Q
Read	Acute Q wave myocardial infarction	XaAC3
Read	REVISION OF CONNECTION OF THORACIC ARTERY TO CORONARY ARTERY	79244
Read	Percutaneous coronary intervention	792E.

Read	Exercise-induced angina	Xa7nH
Read	PROSTHETIC REPLACEMENT OF FOUR+ CORONARY ARTERIES	79233
Read	REVISION OF BYPASS FOR CORONARY ARTERY	7924
Read	Exc myocard infarction quality indicators: informed dissent	XaRFO
Read	Frequency of angina	XaXnr
Read	Ischaemic heart disease	XE2uV
Read	AUTOGRAFT REPLACEMENT OF TWO CORONARY ARTERIES NEC	79211
Read	SAPHENOUS VEIN GRAFT REPLACEMENT OF CORONARY ARTERY OS	7920y
Read	Unstable angina	G3111
Read	Old anterior myocardial infarction	X200W
Read	SAPHENOUS VEIN GRAFT REPLACEMENT OF THREE CORONARY ARTERIES	79202
Read	Transient myocardial ischaemia	G31y3
Read	Other acute and subacute ischaemic heart disease NOS	G31yz
Read	Double anastomosis of mammary arteries to coronary arteries	XE0En
Read	PERCUTANEOUS TRANSLUMINAL ATHERECTOMY OF CORONARY ARTERY	79296
Read	Coronary heart disease medication review	8B3k.
Read	Angina pectoris NOS	G33zz
Read	[V]Presence of aortocoronary bypass graft	ZV457
Read	Acute non-ST segment elevation myocardial infarction	XaIwY
Read	Endarterectomy of coronary artery NEC	792B0
Read	CHD monitoring	662N.
Read	H/O: Angina in last year	XaBL2
Read	Coronary heart disease monitoring default	XaIvJ
Read	OTHER AUTOGRAFT REPLACEMENT OF CORONARY ARTERY	7921
Read	Postoperative myocardial infarction, unspecified	XaD2i
Read	H/O: myocardial infarct >60	14A4.
Read	Perc translum balloon angioplasty stenting coronary art NOS	793Gz
Read	Subsequent myocardial infarction of anterior wall	G350.
Read	Admit ischaemic heart disease emergency	8H2V.
Read	Subsequent myocardial infarction of other sites	G353.
Read	OS perc translum balloon angioplasty stenting coronary art	793Gy
Read	PROSTHETIC REPLACEMENT OF THREE CORONARY ARTERIES	79232
Read	OTHER BYPASS OF CORONARY ARTERY NOS	792Dz
Read	Atherosclerotic cardiovascular disease	G342.
Read	Coronary atherosclerosis	G340.
Read	Other acute myocardial infarction NOS	G30yz
Read	Acute Q wave infarction - lateral	X200Q
Read	Attends coronary heart disease monitoring	XaIvH
Read	ECG: myocardial ischaemia	322..
Read	Angina control - improving	662K2
Read	Hibernating myocardium	Xa1dR
Read	PERCUTANEOUS TRANSLUMINAL LASER CORONARY ANGIOPLASTY	79290
Read	AUTOGRAFT REPLACEMENT OF THREE CORONARY ARTERIES NEC	79212
Read	Syncope anginosa	G33z2
Read	DOUBLE ANASTOMOSIS OF MAMMARY ARTERIES TO CORONARY ARTERIES	79250
Read	Coronary heart disease monitoring telephone invite	9Ob9.
Read	PERCUTAN. TRANSLUM. INJECT THERAP SUBST TO CORONARY ARTERY NEC	79292
Read	Acute posterolateral myocardial infarction	XaJX0
Read	Coronary artery operations NOS	792z.
Read	Angina at rest	X2007
Read	Preinfarction syndrome NOS	G311z
Read	Percut transluminal coronary thrombolysis with streptokinase	79291

Read	Ischaemic chest pain	Ua1eH
Read	SAPHENOUS VEIN GRAFT REPLACEMENT OF ONE CORONARY ARTERY	79200
Read	SINGLE IMPLANTATION THORACIC ARTERY INTO CORONARY ARTERY NEC	79263
Read	Acute inferior myocardial infarction	X200K
Read	Perc translumin balloon angioplasty stenting coronary artery	793G.
Read	Frequency of angina	187..
Read	Coronary microvascular disease	XaYYq
Read	REVISION OF IMPLANTATION OF THORACIC ARTERY INTO HEART	79245
Read	Acute ST segment elevation myocardial infarction	G30X0
Read	PROSTHETIC REPLACEMENT OF ONE CORONARY ARTERY	79230
Read	Stable angina	X2008
Read	REVISION OF BYPASS FOR FOUR+ CORONARY ARTERIES	79243
Read	Acute non-Q wave infarction - lateral	X200R
Read	Perc translum balloon angioplasty insert 1-2 drug elut stents cor art	793G0
Read	Other chronic ischaemic heart disease NOS	G34z.
Read	OTHER THERAPEUTIC TRANSLUMINAL OP ON CORONARY ARTERY OS	7929y
Read	New onset angina	G33z6
Read	Referral to Angina Plan self-management programme declined	XaZlr
Read	Acute transmural myocardial infarction of unspecified site	G30X.
Read	Ruptur chordae tendinae/curr comp fol acute myocard infarct	G364.
Read	Acute non-Q wave infarction - inferior	X200M
Read	Referral to Angina Plan self-management programme declined	8IEY.
Read	Emergency percutaneous coronary intervention	XaaUg
Read	INSERTION OF DRUG-ELUTING CORONARY ARTERY STENT	79295
Read	Microinfarction of heart	G31y1
Read	Coronary artery spasm	G332.
Read	MECHANICAL COMPLICATION OF CORONARY BYPASS	SP003
Read	Coronary heart disease risk clinical management plan	XaJYk
Read	ROTARY BLADE CORONARY ANGIOPLASTY	79293
Read	OTHER OPEN OPERATIONS ON CORONARY ARTERY	7927
Read	Insertion of coronary artery stent	X00tU
Read	Acute coronary syndrome	G3115
Read	Inferior myocardial infarction NOS	G308.
Read	Acute subendocardial infarction	G307.
Read	Transient myocardial ischaemia	XaFsH
Read	CONNECTION OF OTHER THORACIC ARTERY TO CORONARY ARTERY OS	7926y
Read	Perc translum balloon angioplasty insert 1-2 stents cor art	XaLgX
Read	Postoperative myocardial infarction	XaD2b
Read	ALLOGRAFT REPLACEMENT OF THREE CORONARY ARTERIES	79222
Read	Acute atrial infarction	G30y0
Read	Acute non-Q wave infarction	G3070
Read	Other chronic ischaemic heart disease	G34..
Read	ALLOGRAFT REPLACEMENT OF FOUR+ CORONARY ARTERIES	79223
Read	Emergency percutaneous coronary intervention	7.92E+02
Read	Chronic ischaemic heart disease NOS	XE0WG
Read	Other forms of acute ischaemic heart disease	Gyu32
Read	Mural thrombosis	G30A.
Read	Haemopericardium/current comp folow acut myocard infarct	G360.
Read	Old inferior myocardial infarction	X200X
Read	Anterior myocardial infarction NOS	G301z
Read	Other forms of angina pectoris	Gyu30
Read	[V]Presence of coronary artery bypass graft	XaBEB

Read	OTHER SPECIFIED ALLOGRAFT REPLACEMENT OF CORONARY ARTERY	7922y
Read	INSERTION OF CORONARY ARTERY STENT	79294
Read	Angina control	662K.
Read	Silent myocardial ischaemia	G344.
Read	Exc myocar infarction quality indicators: patient unsuitable	9hM1.
Read	Coronary heart disease annual review	XaI9h
Read	Angina decubitus NOS	G330z
Read	Coronary heart disease monitoring verbal invitation	XaIvN
Read	ALLOGRAFT REPLACEMENT OF ONE CORONARY ARTERY	79220
Read	Angina control - worsening	662K3
Read	Repair of coronary artery NEC	792B.
Read	Other open operation on coronary artery NOS	7927z
Read	ECG: myocardial infarct NOS	323Z.
Read	Postoperative subendocardial myocardial infarction	G384.
Read	Refractory angina	G3113
Read	TRANSLUMINAL BALLOON ANGIOPLASTY OF CORONARY ARTERY OS	7928y
Read	Angina self-management plan review	661N0
Read	Coronary heart disease monitoring telephone invite	XaMGk
Read	Antianginal therapy	8B27.
Read	Other specified repair of coronary artery	792By
Read	Chronic coronary insufficiency	G34y0
Read	Attends coronary heart disease monitoring	9Ob0.
Read	History of myocardial infarction	XaQk7
Read	Acute/subacute ischaemic heart disease NOS	XE0WC
Read	Stenocardia	G33z1
Read	Coronary thrombosis not resulting in myocardial infarction	G312.
Read	Acute anterolateral infarction	G300.
Read	REVISION OF BYPASS FOR ONE CORONARY ARTERY	79240
Read	Angina control - poor	662K1
Read	Postoperative transmural myocardial infarction of other sites	G382.
Read	Old myocardial infarction	G32..
Read	Lateral myocardial infarction NOS	G305.
Read	SAPHENOUS VEIN GRAFT REPLACEMENT OF TWO CORONARY ARTERIES	79201
Read	Subsequent myocardial infarction	G35..
Read	Insertion of drug-eluting coronary artery stent	XaLSD
Read	Coronary heart disease monitoring 1st letter	XaIvK
Read	REPLACEMENT OF CORONARY ARTERIES USING MULTIPLE METHODS	792C0
Read	H/O: Myocardial infarction in last year	14AH.
Read	REVISION OF BYPASS FOR TWO CORONARY ARTERIES	79241
Read	Old myocardial infarction	XE2aA
Read	Acute inferolateral infarction	G302.
Read	CONNECTION OF MAMMARY ARTERY TO CORONARY ARTERY OS	7925y
Read	Other acute and subacute ischaemic heart disease	G31..
Read	Acute papillary muscle infarction	G30y1
Read	PERCUT TRANSLUM BALLOON ANGIOPLASTY MULT CORONARY ARTERIES	79281
Read	Postmyocardial infarction syndrome	G310.
Read	SINGLE IMPLANTATION OF MAMMARY ARTERY INTO CORONARY ARTERY	79254
Read	CONNECTION OF OTHER THORACIC ARTERY TO CORONARY ARTERY NOS	7926z
Read	PROSTHETIC REPLACEMENT OF CORONARY ARTERY	7923
Read	Unstable angina	X2009
Read	Acute coronary insufficiency	G31y0
Read	Postoperative transmural myocardial infarction of unspecified site	XaD2g

Read	Subsequent myocardial infarction of unspecified site	Gyu36
Read	ECG: old myocardial infarction	3232
Read	CONNECTION OF OTHER THORACIC ARTERY TO CORONARY ARTERY	7926
Read	Ischaemic heart disease	G3...
Read	CONNECTION OF MAMMARY ARTERY TO CORONARY ARTERY NOS	7925z
Read	History of myocardial infarction	14AT.
Read	Coronary heart disease monitoring 2nd letter	XaIvL
Read	H/O: Myocardial infarction in last year	XaBL1
Read	Other forms of chronic ischaemic heart disease	Gyu33
Read	Coronary heart disease monitoring 3rd letter	9Ob5.
Read	Ischaemic cardiomyopathy	G343.
Read	Stunned myocardium	Xa1dQ
Read	Myocardial ischaemia	X200C
Read	Rupture papillary muscle/curr comp fol acute myocard infarct	G365.
Read	Other specified ischaemic heart disease	G3y..
Read	True posterior myocardial infarction	G306.
Read	Worsening angina	XE0Ui
Read	DIAGNOSTIC TRANSLUMINAL OPERATIONS ON CORONARY ARTERY	792A.
Read	ALLOGRAFT REPLACEMENT OF CORONARY ARTERY	7922
Read	PROSTHETIC REPLACEMENT OF TWO CORONARY ARTERIES	79231
Read	Coronary heart disease annual review	6A2..
Read	Acute myocardial infarction	G30..
Read	Postoperative transmural myocardial infarction of unspecified site	G383.
Read	Acute ST segment elevation myocardial infarction	XaIwM
Read	SAPHENOUS VEIN GRAFT REPLACEMENT CORONARY ARTERY NOS	7920z
Read	Cardiac syndrome X	X200c
Read	Acute non-Q wave infarction - anteroseptal	X200H
Read	Coronary microvascular disease	G39..
Read	PROSTHETIC REPLACEMENT OF CORONARY ARTERY NOS	7923z
Read	DOUBLE IMPLANT OF MAMMARY ARTERIES INTO CORONARY ARTERIES	79251
Read	TRANSLUMINAL BALLOON ANGIOPLASTY OF CORONARY ARTERY	7928
Read	OTHER AUTOGRAFT REPLACEMENT OF CORONARY ARTERY OS	7921y
Read	Percut translum cutting balloon angioplasty coronary artery	XaMKE
Venous thrombo-embolism		
SNOMED	Pulmonary embolism with infarction	1.001E+12
SNOMED	Chronic pulmonary embolism	1.33971E+14
SNOMED	(Pulmonary embolism) or (pulmonary infarct)	155326007
SNOMED	Acute cor pulmonale co-occurrent and due to saddle embolus of pulmonary artery	1.59647E+16
SNOMED	Pulmonary embolism	194882001
SNOMED	PE - Pulmonary thromboembolism	233935004
SNOMED	Acute massive pulmonary embolism	233936003
SNOMED	SAMPE - Subacute massive pulmonary embolism	233937007
SNOMED	Thromboembolic pulmonary hypertension (disorder)	233947005
SNOMED	(Pulmonary embolism) or (pulmonary infarct)	266292008
SNOMED	Saddle embolus of pulmonary artery	3.28511E+14
SNOMED	Recurrent pulmonary embolism	3.39601E+14
SNOMED	Recurrent pulmonary embolism	3.68861E+14
SNOMED	Recurrent pulmonary embolism	438773007
SNOMED	PE - Pulmonary embolism	59282003
SNOMED	Acute pulmonary embolism	706870000
SNOMED	Chronic pulmonary thromboembolism	707412000
SNOMED	Acute pulmonary thromboembolism	707414004

SNOMED	Pulmonary embolism on long-term anticoagulation therapy	713078005
SNOMED	Pulmonary embolism as current complication following acute myocardial infarction	723859005
SNOMED	Thromboembolic pulmonary hypertension	7.78191E+14
SNOMED	Deep vein thrombosis of peroneal vein	5.03701E+14
SNOMED	Postoperative deep vein thrombosis	213220000
SNOMED	(Deep vein phlebitis and thrombophlebitis of the leg) or (deep vein thrombosis)	195403006
SNOMED	Acute deep vein thrombosis of bilateral upper limbs following coronary artery bypass graft	1.57113E+16
SNOMED	Acute deep vein thrombosis of left iliac vein	2.93491E+14
SNOMED	Acute deep vein thrombosis of right lower limb following procedure	1.57112E+16
SNOMED	On deep vein thrombosis care pathway	8.69611E+14
SNOMED	Recurrent deep vein thrombosis	8.24081E+14
SNOMED	Acute deep vein thrombosis of left lower limb as a late effect of coronary artery bypass graft	1.57084E+16
SNOMED	Bilateral acute deep vein thrombosis of femoral veins	2.85381E+14
SNOMED	Postnatal deep vein thrombosis	156269006
SNOMED	Puerperal DVT (deep vein thrombosis)	56272000
SNOMED	Deep vein thrombosis - enhanced services administration	4.92171E+14
SNOMED	Advised risk of deep vein thrombosis in air travel	1.74681E+14
SNOMED	[V] Personal history deep vein thrombosis (context-dependent category)	308158000
SNOMED	Acute deep vein thrombosis of bilateral upper extremities following procedure	1.57083E+16
SNOMED	Acute deep vein thrombosis of right iliac vein	2.93481E+14
SNOMED	Deep vein thrombosis enhanced services administration stage 2 service level	5.03451E+14
SNOMED	Deep vein thrombosis of lower extremity due to intravenous drug use	860699005
SNOMED	Deep vein thrombosis stage 1 service level - enhanced services administration	5.03421E+14
SNOMED	Deep vein thrombosis of lower limb	366912000
SNOMED	[V] Personal history deep vein thrombosis	315760002
SNOMED	DVT (deep vein thrombosis) prevention	3.23631E+14
SNOMED	Unprovoked deep vein thrombosis	9.78421E+14
SNOMED	Acute deep vein thrombosis of lower limb	6.51E+11
SNOMED	Deep vein thrombosis stage 1 service level - enhanced services administration	5.03441E+14
SNOMED	[V] Personal history deep vein thrombosis	4.43131E+14
SNOMED	Deep vein thrombosis enhanced services administration stage 3 service level	5.03481E+14
SNOMED	Deep vein thrombosis screening	4.4611E+13
SNOMED	History of deep vein thrombosis	161508001
SNOMED	Recurrent DVT (deep vein thrombosis)	710167004
SNOMED	Advised risk of deep vein thrombosis in air travel	413459009
SNOMED	Iliofemoral deep vein thrombosis	234044007
SNOMED	Acute deep vein thrombosis of right upper limb following procedure	1.57082E+16
SNOMED	Obstetric venous problems (& [deep vein thrombosis] or [varicose vein])	267361004
SNOMED	Provoked DVT (deep vein thrombosis)	9.78441E+14
SNOMED	Chronic deep vein thrombosis of pelvic vein	709687000
SNOMED	Postnatal deep vein thrombosis with postnatal complication	200238005
SNOMED	Antenatal deep vein thrombosis unspecified	6.05811E+14
SNOMED	DVT - Deep vein thrombosis	128053003
SNOMED	Acute deep vein thrombosis of bilateral lower limbs following procedure	1.57113E+16
SNOMED	Acute deep vein thrombosis of left upper limb following procedure	1.57084E+16
SNOMED	History of recurrent deep vein thrombosis	1.41911E+14
SNOMED	Chronic deep vein thrombosis during postpartum period	1.72735E+17
SNOMED	Deep vein thrombosis of portal vein	17920008
SNOMED	Postnatal deep vein thrombosis NOS	200239002
SNOMED	Acute deep vein thrombosis of right lower limb as a sequela of coronary artery bypass graft	1.57082E+16
SNOMED	Deep vein thrombosis of bilateral lower extremities	444325005
SNOMED	[V] Personal history deep vein thrombosis (context-dependent category)	4.29311E+14

SNOMED	Deep vein thrombosis	128057002
SNOMED	Deep vein thrombosis of leg related to air travel	134399007
SNOMED	Deep vein thrombosis enhanced services administration	4.92161E+14
SNOMED	DVT - Deep vein thrombosis of lower limb	404223003
SNOMED	Provision of written information about reducing risk of DVT (deep vein thrombosis)	8.60631E+14
SNOMED	Deep vein thrombosis stage 2 service level - enhanced services administration	5.03471E+14
SNOMED	Chronic deep vein thrombosis of right upper extremity following procedure	1287896006
SNOMED	Antenatal DVT (deep vein thrombosis)	49956009
SNOMED	Postnatal deep vein thrombosis unspecified	200236009
SNOMED	Chronic deep vein thrombosis during pregnancy	1.04759E+17
SNOMED	Acute DVT (deep vein thrombosis) of left peroneal vein	4.6557E+16
SNOMED	Antenatal deep vein thrombosis - delivered	200232006
SNOMED	Antenatal deep vein thrombosis NOS	200234007
SNOMED	Provoked deep vein thrombosis	9.78451E+14
SNOMED	Antenatal deep vein thrombosis NOS	6.15861E+14
SNOMED	Postpartum acute deep vein thrombosis	1.64354E+17
SNOMED	Antenatal deep vein thrombosis	156268003
SNOMED	Deep vein thrombosis clinic	702845003
SNOMED	Antenatal deep vein thrombosis with antenatal complication	200233001
SNOMED	Deep vein thrombosis-non obst.	155454004
SNOMED	Deep vein thrombosis - enhanced services administration	4.92181E+14
SNOMED	Postnatal deep vein thrombosis unspecified	6.15871E+14
SNOMED	Unprovoked deep vein thrombosis	9.78431E+14
SNOMED	Chronic deep vein thrombosis of left iliac vein	2.93461E+14
SNOMED	Acute deep vein thrombosis of left lower limb following procedure	1.57114E+16
SNOMED	Deep vein thrombosis stage 3 service level - enhanced services administration	5.03501E+14
SNOMED	Deep vein thrombosis of peroneal vein	443210003
SNOMED	Chronic deep vein thrombosis of right iliac vein	2.93451E+14
SNOMED	Chronic deep vein thrombosis of bilateral upper limbs following procedure	1.57086E+16
SNOMED	Antenatal deep vein thrombosis unspecified	200231004
SNOMED	Referral to deep vein thrombosis clinic	3.26441E+14
SNOMED	Chronic deep vein thrombosis of left upper limb following procedure	1287895005
SNOMED	Deep vein thrombosis stage 2 service level - enhanced services administration	5.03461E+14
SNOMED	Deep vein thrombosis of leg related to intravenous drug use	413956008
SNOMED	Deep vein thrombosis of splenic vein	14534009
SNOMED	Deep vein thrombosis stage 1 service level - enhanced services administration	5.03431E+14
SNOMED	Deep vein thrombosis of leg related to air travel	366830006
SNOMED	Acute deep vein thrombosis during pregnancy	2.92834E+17
SNOMED	Deep vein thrombosis of leg related to intravenous drug use	1.76351E+14
SNOMED	Deep vein thrombosis of leg related to air travel	366911007
SNOMED	Acute DVT (deep vein thrombosis) of right peroneal vein	8.43434E+17
SNOMED	Deep vein thrombosis-non obst.	266328001
SNOMED	Postnatal deep vein thrombosis	200235008
SNOMED	Recurrent deep vein thrombosis	8.34941E+14
SNOMED	Non-pyogenic venous sinus thrombosis	195229008
SNOMED	Intracranial sinus thrombosis, embolism AND/OR inflammation	106016005
SNOMED	Cerebral venous sinus thrombosis	192759008
SNOMED	Sagittal sinus thrombosis	70607008
SNOMED	Cerebral venous sinus thrombosis in pregnancy	1156027008
SNOMED	Postoperative cerebral venous sinus thrombosis	1078223005
SNOMED	Neonatal noninfectious cerebral venous sinus thrombosis	702374000
SNOMED	Cerebral venous sinus thrombosis in puerperium	1156029006

Read	Pulmonary embolism	G401.
Read	Recurrent pulmonary embolism	G4011
Read	Thromboembolic pulmonary hypertension	G41y1
Read	Pulmonary thromboembolism	X202x
Read	Acute massive pulmonary embolism	X202y
Read	Subacute massive pulmonary embolism	X202z
Read	Thromboembolic pulmonary hypertension	X203A
Read	Recurrent pulmonary embolism	XaOYV
Read	Pulmonary embolus	XE0Um
Read	(Pulmonary embolism) or (pulmonary infarct)	XE0WI
Read	Deep vein thrombosis - enhanced services administration	9kg..
Read	Postnatal deep vein thrombosis	L414.
Read	Referral to deep vein thrombosis clinic	8HTm.
Read	On deep vein thrombosis care pathway	8CMWA
Read	Deep vein thrombosis of lower limb	G801D
Read	Deep vein thrombosis of leg related to intravenous drug use	G801E
Read	Deep vein thrombosis of leg related to air travel	G801C
Read	Deep vein thrombosis of peroneal vein	G801F
Read	Recurrent deep vein thrombosis	G801G
Read	[V] Personal history deep vein thrombosis	ZV128
Read	Unprovoked deep vein thrombosis	G801H
Read	Provoked deep vein thrombosis	G801J
Read	Postoperative deep vein thrombosis	SP122
Read	H/O: Deep Vein Thrombosis	14A81
Read	Postnatal deep vein thrombosis NOS	L414z
Read	Postnatal deep vein thrombosis unspecified	L4140
Read	Postnatal deep vein thrombosis - delivered with p/n comp	L4141
Read	Postnatal deep vein thrombosis with postnatal complication	L4142
Read	Deep vein thrombosis screening	XaIu9
Read	On deep vein thrombosis care pathway	XaaBG
Read	Deep vein thrombosis prevention	Ua2EC
Read	Suspected deep vein thrombosis	XaNfd
Read	Referral to deep vein thrombosis clinic	XaO8G
Read	Recurrent deep vein thrombosis	XaZ43
Read	Ileofemoral deep vein thrombosis	X205n
Read	Deep vein thrombosis of leg related to intravenous drug use	XaJxo
Read	Deep vein thrombosis enhanced services administration	XaQ8U
Read	[V] Personal history deep vein thrombosis	XaBMc
Read	Unprovoked deep vein thrombosis	Xacvd
Read	Deep vein thrombosis of lower limb	Xa9Bs
Read	Deep vein thrombosis of leg related to air travel	XaIIo
Read	Postnatal deep vein thrombosis	XE0xL
Read	Provoked deep vein thrombosis	Xacve
Read	Cerebral venous sinus thrombosis	F051.
Read	Non-pyogenic venous sinus thrombosis	G676.
Atrial fibrillation		
SNOMED	Chronic atrial fibrillation	1.0327E+15
SNOMED	Chronic atrial fibrillation	1.03271E+15
SNOMED	Typical atrial flutter	1.03272E+15
SNOMED	Typical atrial flutter	1.03273E+15
SNOMED	Atypical atrial flutter	1.03274E+15
SNOMED	Atypical atrial flutter	1.03275E+15

SNOMED	Atrial fibrillation detected	1.06683E+15
SNOMED	Atrial fibrillation with rapid ventricular response	1.20041E+14
SNOMED	Atrial fibrillation monitoring	134377004
SNOMED	H/O: atrial fibrillation	138790004
SNOMED	ECG: atrial fibrillation	142049000
SNOMED	ECG: atrial flutter	142050000
SNOMED	Evaluation of AF (atrial fibrillation) burden	1.43601E+14
SNOMED	(Atrial fibrillation) or (atrial flutter)	155364009
SNOMED	Atypical atrial flutter	1.59649E+16
SNOMED	H/O: atrial fibrillation	161521008
SNOMED	ECG: atrial fibrillation	164889003
SNOMED	ECG: atrial flutter	164890007
SNOMED	Implantation of intravenous pacemaker for atrial fibrillation	175146007
SNOMED	Atrial fibrillation and flutter	195080001
SNOMED	Paroxysmal atrial fibrillation	195081002
SNOMED	Atrial fibrillation and flutter NOS	195082009
SNOMED	Lone atrial fibrillation	233910005
SNOMED	Non-rheumatic atrial fibrillation	233911009
SNOMED	Atrial fibrillation monitoring administration	2.48111E+14
SNOMED	Atrial fibrillation annual review	2.48411E+14
SNOMED	Atrial fibrillation monitoring first letter	2.49021E+14
SNOMED	Atrial fibrillation monitoring second letter	2.49031E+14
SNOMED	Atrial fibrillation monitoring third letter	2.49041E+14
SNOMED	Atrial fibrillation monitoring verbal invite	2.49051E+14
SNOMED	Atrial fibrillation monitoring telephone invite	2.49101E+14
SNOMED	Atrial fibrillation annual review	2.50591E+14
SNOMED	Atrial fibrillation monitoring administration	2.51701E+14
SNOMED	Atrial fibrillation monitoring first letter	2.51711E+14
SNOMED	Atrial fibrillation monitoring second letter	2.51721E+14
SNOMED	Atrial fibrillation monitoring third letter	2.51731E+14
SNOMED	Atrial fibrillation monitoring verbal invite	2.51741E+14
SNOMED	Atrial fibrillation monitoring telephone invite	2.51751E+14
SNOMED	Atrial fibrillation annual review	2.53221E+14
SNOMED	(Atrial fibrillation) or (atrial flutter)	266306001
SNOMED	Atrial fibrillation monitoring administration	2.74681E+14
SNOMED	Atrial fibrillation monitoring first letter	2.74691E+14
SNOMED	Atrial fibrillation monitoring second letter	2.74701E+14
SNOMED	Atrial fibrillation monitoring third letter	2.74711E+14
SNOMED	Atrial fibrillation monitoring verbal invite	2.74721E+14
SNOMED	Atrial fibrillation monitoring telephone invite	2.74731E+14
SNOMED	Percutaneous transluminal ablation of atrial wall for atrial flutter	2.81471E+14
SNOMED	AF - Paroxysmal atrial fibrillation	282825002
SNOMED	Percutaneous transluminal ablation of conducting system of heart for atrial flutter NEC	2.83251E+14
SNOMED	Percutaneous transluminal ablation of atrial wall for atrial flutter	2.89871E+14
SNOMED	Percutaneous transluminal ablation of conducting system of heart for atrial flutter NEC	2.89891E+14
SNOMED	Controlled atrial fibrillation	300996004
SNOMED	History of atrial flutter	3.05671E+14
SNOMED	History of atrial flutter	3.05681E+14
SNOMED	History of atrial flutter	3.05691E+14
SNOMED	H/O: atrial fibrillation	312442005
SNOMED	Rapid atrial fibrillation	314208002
SNOMED	Atrial fibrillation monitoring	367037002

SNOMED	Atrial fibrillation monitoring	367266005
SNOMED	Permanent atrial fibrillation	3.68881E+14
SNOMED	Persistent atrial fibrillation	3.68891E+14
SNOMED	Non-rheumatic atrial fibrillation	4.1241E+13
SNOMED	Chronic atrial flutter	425615007
SNOMED	Chronic atrial fibrillation	426749004
SNOMED	Transient cerebral ischaemia due to atrial fibrillation	426814001
SNOMED	Paroxysmal atrial flutter	427665004
SNOMED	History of atrial flutter	428076002
SNOMED	Maze procedure for atrial fibrillation	429211003
SNOMED	History of maze procedure for atrial fibrillation	429218009
SNOMED	Permanent atrial fibrillation	440028005
SNOMED	Persistent atrial fibrillation	440059007
SNOMED	Non-rheumatic atrial fibrillation	4.4021E+13
SNOMED	Insertion of pacemaker for control of atrial fibrillation	449863006
SNOMED	AF - Atrial fibrillation	49436004
SNOMED	Atrial flutter	5370000
SNOMED	Atrial fibrillation and flutter NOS	6.08471E+14
SNOMED	Longstanding persistent atrial fibrillation	706923002
SNOMED	Percutaneous transluminal ablation of atrial wall for atrial flutter	707832008
SNOMED	Postoperative fluttering heart	709065005
SNOMED	Atrial fibrillation monitoring invitation	7.11411E+14
SNOMED	Atrial fibrillation monitoring administration	7.13801E+14
SNOMED	Familial atrial fibrillation	715395008
SNOMED	Atrial fibrillation monitoring third letter	7.16181E+14
SNOMED	Atrial fibrillation monitoring telephone invite	7.16721E+14
SNOMED	Atrial fibrillation monitoring second letter	7.16981E+14
SNOMED	Atrial fibrillation monitoring verbal invite	7.17011E+14
SNOMED	Atrial fibrillation monitoring first letter	7.17221E+14
SNOMED	Atrial flutter type 1	720448006
SNOMED	Referral to atrial fibrillation clinic	758600000
SNOMED	Preexcited atrial fibrillation	762247006
SNOMED	Referral to atrial fibrillation clinic	7.85111E+14
SNOMED	Referral to atrial fibrillation clinic	7.85121E+14
SNOMED	Non-rheumatic atrial fibrillation	7.8591E+13
SNOMED	Atrial fibrillation and flutter	81216002
SNOMED	Non-rheumatic atrial fibrillation	8.3371E+13
SNOMED	Atrial fibrillation care pathway	8.47611E+14
SNOMED	Following atrial fibrillation care pathway protocol	8.47621E+14
SNOMED	Paroxysmal atrial flutter	8.81041E+14
SNOMED	Paroxysmal atrial flutter	8.90571E+14
Read	ECG: atrial fibrillation	3272
Read	ECG: atrial flutter	3273
Read	Atrial fibrillation monitoring	662S.
Read	Atrial fibrillation annual review	6A9..
Read	Implantation of intravenous pacemaker for atrial fibrillation	7936A
Read	Referral to atrial fibrillation clinic	8HTy.
Read	Atrial fibrillation monitoring administration	9Os..
Read	Atrial fibrillation monitoring first letter	9Os0.
Read	Atrial fibrillation monitoring second letter	9Os1.
Read	Atrial fibrillation monitoring third letter	9Os2.
Read	Atrial fibrillation monitoring verbal invite	9Os3.

Read	Atrial fibrillation monitoring telephone invite	9Os4.
Read	Atrial fibrillation and flutter	G573.
Read	Atrial fibrillation	G5730
Read	Atrial flutter	G5731
Read	Paroxysmal atrial fibrillation	G5732
Read	Non-rheumatic atrial fibrillation	G5733
Read	Permanent atrial fibrillation	G5734
Read	Persistent atrial fibrillation	G5735
Read	Paroxysmal atrial flutter	G5736
Read	Chronic atrial fibrillation	G5737
Read	Typical atrial flutter	G5738
Read	Atypical atrial flutter	G5739
Read	Atrial fibrillation and flutter NOS	G573z
Read	Lone atrial fibrillation	X202R
Read	Non-rheumatic atrial fibrillation	X202S
Read	Paroxysmal atrial fibrillation	Xa2E8
Read	Controlled atrial fibrillation	Xa7nI
Read	Paroxysmal atrial flutter	XaaUH
Read	Rapid atrial fibrillation	XaEga
Read	Chronic atrial fibrillation	XaeUP
Read	Typical atrial flutter	XaeUQ
Read	Atypical atrial flutter	XaeUR
Read	Atrial fibrillation monitoring	XaIIT
Read	Atrial fibrillation monitoring administration	XaMDF
Read	Atrial fibrillation monitoring first letter	XaMDG
Read	Atrial fibrillation monitoring second letter	XaMDH
Read	Atrial fibrillation monitoring third letter	XaMDI
Read	Atrial fibrillation monitoring verbal invite	XaMDK
Read	Atrial fibrillation monitoring telephone invite	XaMFn
Read	Atrial fibrillation annual review	XaMGD
Read	Persistent atrial fibrillation	XaOfa
Read	Permanent atrial fibrillation	XaOft
Read	Referral to atrial fibrillation clinic	XaXrZ
Read	(Atrial fibrillation) or (atrial flutter)	XE0Wk
Cerebrovascular desease		
SNOMED	Paralytic syndrome of both lower limbs as sequela of stroke	102831000119104
SNOMED	Paralytic syndrome of all four limbs as sequela of stroke	103761000119107
SNOMED	Spasticity as sequela of stroke	108691000119102
SNOMED	Cerebral infarction due to occlusion of cerebral artery (disorder)	1089411000000100
SNOMED	Cerebral infarction due to stenosis of cerebral artery (disorder)	1089421000000100
SNOMED	Nonparalytic stroke	111297002
SNOMED	Sequela of cerebrovascular accident	1131000119105
SNOMED	Paralytic stroke	116288000
SNOMED	CI - Cerebral infarction	1222398015
SNOMED	Weakness of left facial muscle due to and following cerebrovascular accident	12242711000119100
SNOMED	Weakness of right facial muscle due to and following cerebrovascular accident (disorder)	1224275100011

		9100
SNOMED	Paraplegia as sequela of cerebrovascular accident	12367511000119100
SNOMED	Cerebral infarction due to occlusion of precerebral artery	125081000119106
SNOMED	Alteration of sensation as late effect of stroke	134771000119108
SNOMED	Acute cerebrovascular accident due to occlusion of right posterior cerebral artery	137592291000119000
SNOMED	Seizure disorder as sequela of stroke	137991000119103
SNOMED	History of transient ischaemic attack due to embolism	140221000119109
SNOMED	Ischemic stroke with coma	140911000119109
SNOMED	Ischemic stroke without coma	140921000119102
SNOMED	History of ischemic cerebrovascular accident with residual deficit	141821000119104
SNOMED	Weakness as a late effect of cerebrovascular accident	148871000119109
SNOMED	Cerebral infarction due to carotid artery occlusion	149821000119103
SNOMED	CVA - Cerebrovascular accident due to intracerebral haemorrhage	1508000
SNOMED	Transient ischaemic attacks	155404005
SNOMED	Cerebrovascular accident	155405006
SNOMED	Cerebrovascular accident due to occlusion of left posterior cerebral artery	16000351000119100
SNOMED	Cerebrovascular accident due to occlusion of right posterior cerebral artery (disorder)	16000391000119100
SNOMED	Cerebrovascular accident due to occlusion of right middle cerebral artery (disorder)	16000431000119100
SNOMED	Cerebrovascular accident due to occlusion of left middle cerebral artery (disorder)	16000511000119100
SNOMED	Cerebrovascular accident due to thrombus of right middle cerebral artery	16002031000119100
SNOMED	Cerebrovascular accident due to thrombus of left middle cerebral artery	16002111000119100
SNOMED	Cerebrovascular accident due to occlusion of right carotid artery (disorder)	16023911000119100
SNOMED	Cerebrovascular accident due to occlusion of left pontine artery (disorder)	16023991000119100
SNOMED	Cerebrovascular accident due to occlusion of right pontine artery	16024031000119100
SNOMED	Cerebrovascular accident due to occlusion of left carotid artery (disorder)	16024111000119100
SNOMED	Cerebrovascular accident due to occlusion of left cerebellar artery	16024151000119100
SNOMED	Cerebrovascular accident due to stenosis of right carotid artery	16026951000119100
SNOMED	Cerebrovascular accident due to stenosis of left carotid artery (disorder)	16026991000119100

SNOMED	History of transient ischaemic attack	161511000
SNOMED	Dysphasia due to and following cerebrovascular accident (disorder)	16260551000119100
SNOMED	Cerebellar stroke	16371781000119100
SNOMED	Cerebrovascular accident due to occlusion of bilateral pontine arteries	16644681000119100
SNOMED	Cerebrovascular accident due to stenosis of bilateral vertebral arteries	16661931000119100
SNOMED	Cerebrovascular accident due to stenosis of bilateral carotid arteries	16661971000119100
SNOMED	[V]Personal history of transient ischaemic attack	1667741000000110
SNOMED	Memory deficit due to and following cerebrovascular accident (disorder)	16703661000119100
SNOMED	Memory deficit as sequela of embolic cerebrovascular accident	16703711000119100
SNOMED	Memory deficit as a sequela of ischaemic cerebrovascular accident	16703761000119100
SNOMED	Memory deficit due to and following haemorrhagic cerebrovascular accident	16703821000119100
SNOMED	History of embolic cerebrovascular accident	16896851000119100
SNOMED	History of cerebrovascular accident due to ischaemia	16896891000119100
SNOMED	Apraxia due to and following cerebrovascular accident	186831000119104
SNOMED	Cerebral infarction due to embolism of precerebral arteries (disorder)	195186005
SNOMED	CVA - cerebrovascular accident due to cerebral artery occlusion	195188006
SNOMED	Cerebral infarction due to thrombosis of cerebral arteries	195189003
SNOMED	Cerebral infarction due to embolism of cerebral arteries	195190007
SNOMED	Cerebral infarction NOS	195191006
SNOMED	Left sided cerebral infarction	195194003
SNOMED	Right sided cerebral infarction (disorder)	195195002
SNOMED	Transient ischaemic attack	195196001
SNOMED	Stroke and cerebrovascular accident unspecified	195208004
SNOMED	Brainstem stroke syndrome	195212005
SNOMED	Cerebellar stroke syndrome	195213000
SNOMED	Right sided cerebral hemisphere cerebrovascular accident (disorder)	195217004
SNOMED	Cerebral infarction due to cerebral venous thrombosis, non-pyogenic (disorder)	195230003
SNOMED	Occlusion and stenosis of cerebral arteries, not resulting in cerebral infarction	195231004
SNOMED	Sequelae of cerebral infarction	195243003
SNOMED	[X]Cerebral infarction due to unspecified occlusion or stenosis of precerebral arteries	195246006
SNOMED	[X]Cerebral infarction due to unspecified occlusion or stenosis of cerebral arteries	195247002
SNOMED	[X]Cerebral infarction due to unspecified occlusion or stenosis of cerebral arteries	195599001
SNOMED	[X]Other cerebral infarction	195600003
SNOMED	[X]Cerebral infarction due to unspecified occlusion or stenosis of precerebral arteries	195612004
SNOMED	Cerebrovascular disorders in the puerperium (& [CVA - cerebrovascular accident]) (disorder)	200328002
SNOMED	CI - Cerebral infarction	20059004
SNOMED	Cerebrovascular accident	230690007
SNOMED	Cerebrovascular accident due to occlusion of cerebral artery (disorder)	230691006
SNOMED	Total anterior cerebral circulation infarction	230694003

SNOMED	Partial anterior cerebral circulation infarction	230695002
SNOMED	Posterior cerebral circulation infarction	230696001
SNOMED	Lacunar infarction	230698000
SNOMED	Stroke of uncertain pathology	230713003
SNOMED	Anterior circulation stroke of uncertain pathology	230714009
SNOMED	Posterior circulation stroke of uncertain pathology	230715005
SNOMED	Carotid territory transient ischaemic attack	230716006
SNOMED	Vertebrobasilar territory transient ischaemic attack	230717002
SNOMED	Sequela of ischemic cerebral infarction	23671000119107
SNOMED	Old cerebral infarction on imaging	2436731000000110
SNOMED	History of - cerebrovascular accident	2476091017
SNOMED	Completed stroke	25133001
SNOMED	History of transient ischaemic attack	251692018
SNOMED	Cerebral infarction NOS (disorder)	266256009
SNOMED	TIA - Transient ischaemic attack	266257000
SNOMED	Transient ischaemic attacks	266314007
SNOMED	Cerebrovascular accident	266315008
SNOMED	Stroke and cerebrovascular accident unspecified	270883006
SNOMED	Stroke in the puerperium	275434003
SNOMED	History of - cerebrovascular accident (situation)	275526006
SNOMED	Occipital cerebral infarction	276219001
SNOMED	Cerebral infarction	2770034014
SNOMED	Extension of cerebrovascular accident	281240008
SNOMED	Ataxia due to and following cerebrovascular accident	290581000119101
SNOMED	Dysarthria due to and following cerebrovascular accident	290631000119103
SNOMED	Fluency disorder due to and following cerebrovascular accident	290791000119105
SNOMED	Monoplegia of lower limb due to and following cerebrovascular accident (disorder)	290931000119108
SNOMED	Monoplegia of left nondominant upper limb due to and following cerebrovascular accident	291091000119102
SNOMED	Monoplegia of right nondominant upper limb due to and following cerebrovascular accident	291111000119105
SNOMED	Monoplegia of upper limb due to and following cerebrovascular accident	291121000119103
SNOMED	Cerebrovascular accident due to stenosis of right vertebral artery (disorder)	292661000119105
SNOMED	Cerebrovascular accident due to occlusion of right vertebral artery (disorder)	292681000119101
SNOMED	Cerebrovascular accident due to occlusion of left vertebral artery (disorder)	292691000119103
SNOMED	Cerebral infarction due to vertebral artery stenosis (disorder)	293811000119100
SNOMED	Cerebral infarction due to stenosis of precerebral artery (disorder)	293831000119105
SNOMED	History of cerebrovascular accident (situation)	2986886017
SNOMED	Intracerebral haemorrhage (& [cerebrovascular accident due to])	300259018
SNOMED	Cerebral infarction due to embolism of precerebral arteries	300313017

SNOMED	CVA - cerebrovascular accident due to cerebral artery occlusion	300315012
SNOMED	Cerebral infarction due to thrombosis of cerebral arteries (disorder)	300321011
SNOMED	Cerebral infarction due to embolism of cerebral arteries (disorder)	300322016
SNOMED	Cerebral infarction NOS	300323014
SNOMED	Transient ischaemic attack	300339019
SNOMED	Left sided cerebral hemisphere cerebrovascular accident (disorder)	300369014
SNOMED	Left sided cerebral hemisphere cerebrovascular accident (disorder)	300370010
SNOMED	Right sided cerebral hemisphere cerebrovascular accident (disorder)	300371014
SNOMED	Right sided cerebral hemisphere cerebrovascular accident (disorder)	300372019
SNOMED	Cerebral infarction due to cerebral venous thrombosis, non-pyogenic	300393016
SNOMED	Occlusion and stenosis of cerebral arteries, not resulting in cerebral infarction (disorder)	300394010
SNOMED	Sequelae of cerebral infarction (disorder)	300411016
SNOMED	Cerebral infarction due to occlusion of precerebral artery (disorder)	3042974014
SNOMED	Left sided cerebral infarction (disorder)	307766002
SNOMED	Right sided cerebral infarction (disorder)	307767006
SNOMED	CVA - cerebrovascular accident in the puerperium	307875011
SNOMED	Transient ischaemic attacks	313242003
SNOMED	Cerebrovascular accident due to occlusion of left middle cerebral artery by embolus	329371000119101
SNOMED	Cerebrovascular accident due to occlusion of left posterior cerebral artery by embolus	329431000119105
SNOMED	Cerebrovascular accident due to occlusion of left cerebellar artery by embolus	329461000119102
SNOMED	Cerebrovascular accident due to thrombus of basilar artery	329641000119104
SNOMED	Cerebrovascular accident due to thrombus of right carotid artery	329651000119102
SNOMED	Cerebrovascular accident due to thrombus of left carotid artery	330791000119108
SNOMED	Sequela of cardioembolic stroke	33301000119105
SNOMED	Sequela of lacunar stroke	33331000119103
SNOMED	Cerebral infarction	33759015
SNOMED	CVA - Cerebral infarction	33762017
SNOMED	Cerebral infarction due to occlusion of basilar artery	34181000119102
SNOMED	Cerebral infarction due to vertebral artery occlusion	34191000119104
SNOMED	Cerebrovascular accident (disorder)	345635016
SNOMED	CVA - Cerebrovascular accident	345636015
SNOMED	Cerebrovascular accident (disorder)	345637012
SNOMED	Cerebrovascular accident due to occlusion of cerebral artery (disorder)	345638019
SNOMED	Anterior circulation transient ischaemic attack	345683018
SNOMED	Anterior circulation transient ischaemic attack	345684012
SNOMED	Posterior circulation transient ischaemic attack	345689019
SNOMED	Vertebrobasilar territory transient ischaemic attack	345690011
SNOMED	Acute cerebrovascular accident due to thrombosis of left middle cerebral artery	346410121000119000
SNOMED	Thrombotic stroke	371040005
SNOMED	Embolic stroke	371041009
SNOMED	Occlusive stroke	373606000

SNOMED	Transient ischaemic attack	38609002
SNOMED	TIA - Transient ischaemic attack	395781014
SNOMED	Transient ischaemic attack	395783012
SNOMED	TIA - Transient ischaemic attack	395784018
SNOMED	Transient ischaemic attack	395785017
SNOMED	Transient ischaemic attack	395788015
SNOMED	Cerebrovascular accident	395967015
SNOMED	Transient ischaemic attacks	395972012
SNOMED	(Cerebral infarct) or (cerebrovascular accident) or (undefined stroke/CVA) or (stroke NOS) (disorder)	395987019
SNOMED	(Cerebral infarct) or (cerebrovascular accident) or (undefined stroke/CVA) or (stroke NOS) (disorder)	395992017
SNOMED	Acute cerebrovascular accident due to occlusion of left posterior cerebral artery	40076141000119100
SNOMED	Weakness of face muscles as sequela of stroke	40161000119102
SNOMED	Acute cerebrovascular accident due to occlusion of right carotid artery	407573811000119000
SNOMED	History of - cerebrovascular accident	411518010
SNOMED	Occipital cerebral infarction	412270012
SNOMED	Infarction of basal ganglia	413102000
SNOMED	Cardioembolic stroke	413758000
SNOMED	[X]Cerebral infarction due to unspecified occlusion or stenosis of precerebral arteries	415151000000100
SNOMED	Extension of cerebrovascular accident	419212013
SNOMED	Extension of cerebrovascular accident	419213015
SNOMED	Extension of cerebrovascular accident (disorder)	419214014
SNOMED	[X]Cerebral infarction due to unspecified occlusion or stenosis of cerebral arteries (disorder)	419691000000103
SNOMED	[X]Other cerebral infarction	419701000000103
SNOMED	Ischemic stroke	422504002
SNOMED	Monoplegia of dominant lower limb as a late effect of cerebrovascular accident	425642008
SNOMED	Paralytic syndrome as late effect of stroke	425882004
SNOMED	Dysphagia as a late effect of cerebrovascular accident	426033005
SNOMED	[X]Cerebral infarction due to unspecified occlusion or stenosis of precerebral arteries (disorder)	426661000000105
SNOMED	Vertigo as late effect of stroke	426788002
SNOMED	Infarction of medulla oblongata	426983002
SNOMED	Thalamic infarction	427296003
SNOMED	Paralytic syndrome as late effect of thalamic stroke	427432001
SNOMED	[V]Personal history of cerebrovascular accident (CVA)	428011000000100
SNOMED	Apraxia due to cerebrovascular accident	428668000
SNOMED	History of cerebrovascular accident without residual deficits	429993008
SNOMED	Paralytic syndrome of nondominant side as late effect of stroke	430947007
SNOMED	Paralytic syndrome of dominant side as late effect of stroke	430959006
SNOMED	Cerebral infarction	432504007
SNOMED	Neurogenic bladder as late effect of cerebrovascular accident	433183000
SNOMED	History of occlusion of cerebral artery without cerebral infarction (situation)	433807000
SNOMED	History of cerebrovascular accident with residual deficit (situation)	440140008
SNOMED	Infarct of cerebrum due to iatrogenic cerebrovascular accident (disorder)	441526008
SNOMED	Monoplegia of nondominant lower limb as a late effect of cerebrovascular accident (disorder)	441894009
SNOMED	Speech and language deficit as late effect of cerebrovascular accident	441960006

SNOMED	Hemiparesis as late effect of cerebrovascular accident	441991000
SNOMED	Monoplegia of nondominant upper limb as a late effect of cerebrovascular accident	442181008
SNOMED	Residual cognitive deficit as late effect of cerebrovascular accident (disorder)	442212003
SNOMED	Aphasia as late effect of cerebrovascular accident	442617003
SNOMED	[X]Cerebral infarction due to unspecified occlusion or stenosis of cerebral arteries (disorder)	444151000000109
SNOMED	Left sided cerebral infarction	451133011
SNOMED	Right sided cerebral infarction (disorder)	451134017
SNOMED	Behavior disorder as sequela of cerebral infarction (disorder)	46421000119102
SNOMED	CVA - Cerebral infarction	480288019
SNOMED	Cerebral infarction	480289010
SNOMED	CVA - Cerebral infarction	480290018
SNOMED	Paralytic syndrome on one side of the body as effect of cerebrovascular accident	48601000119107
SNOMED	Acute cerebrovascular accident due to occlusion of left carotid artery	502836591000119000
SNOMED	CVA - cerebrovascular accident due to cerebral artery occlusion	579254017
SNOMED	Transient ischaemic attack	579263015
SNOMED	Left sided cerebral hemisphere cerebrovascular accident (disorder)	579285014
SNOMED	Progressing stroke	57981008
SNOMED	Cerebrovascular disorders in the puerperium (& [CVA - cerebrovascular accident]) (disorder)	585058012
SNOMED	Acute cerebrovascular accident of basal ganglia (disorder)	595899961000119000
SNOMED	Acute cerebrovascular accident due to occlusion of left middle cerebral artery	63230211000119100
SNOMED	Acute cerebrovascular accident of brainstem (disorder)	652287331000119000
SNOMED	Cerebrovascular accident	658791018
SNOMED	(Transient ischaemic attacks) or (vertebro-basilar insufficiency) or (drop attacks)	658793015
SNOMED	Cerebrovascular accident	658794014
SNOMED	Hemiplegia of nondominant side due to and following ischemic cerebrovascular accident	672441000119103
SNOMED	Hemiplegia of dominant side due to and following ischemic cerebrovascular accident	672461000119104
SNOMED	Dysarthria due to and following ischemic cerebrovascular accident	672501000119104
SNOMED	Dysphasia due to and following ischemic cerebrovascular accident	672521000119108
SNOMED	Aphasia due to and following ischemic cerebrovascular accident	672541000119102
SNOMED	Aphasia due to and following haemorrhagic cerebrovascular accident	672551000119100
SNOMED	Cognitive deficit due to and following ischemic cerebrovascular accident	672561000119103
SNOMED	Cerebrovascular accident due to thrombus of right middle cerebral artery	672571000119109
SNOMED	Vertigo due to and following ischemic cerebrovascular accident	674091000119108
SNOMED	Ataxia due to and following ischemic cerebrovascular accident	674111000119100
SNOMED	Ataxia due to and following haemorrhagic cerebrovascular accident	6741210001191

		07
SNOMED	Monoplegia of upper limb due to and following ischemic cerebrovascular accident	674161000119102
SNOMED	Apraxia due to and following ischemic cerebrovascular accident	674361000119104
SNOMED	Weakness of facial muscle due to and following ischemic cerebrovascular accident	674381000119108
SNOMED	Speech and language deficit due to and following ischemic cerebrovascular accident	674401000119108
SNOMED	Cerebral infarction NOS (disorder)	682621000000105
SNOMED	Stroke and cerebrovascular accident unspecified	685631000000102
SNOMED	Weakness of facial muscle due to and following embolic cerebrovascular accident	690171000119105
SNOMED	Ataxia as sequela of embolic cerebrovascular accident	690201000119109
SNOMED	Hemiplegia of nondominant side due to and following embolic cerebrovascular accident	690271000119104
SNOMED	Dysarthria due to and following embolic cerebrovascular accident	690311000119104
SNOMED	Aphasia due to and following embolic cerebrovascular accident	690321000119106
SNOMED	Speech and language deficit due to and following embolic cerebrovascular accident	690331000119109
SNOMED	Cognitive deficit due to and following embolic cerebrovascular accident	690341000119100
SNOMED	Dysphasia due to and following embolic cerebrovascular accident	690351000119103
SNOMED	Acute cerebrovascular accident due to stenosis of right carotid artery	693158221000119000
SNOMED	Post-cerebrovascular accident epilepsy	698767004
SNOMED	Cerebral infarction due to embolism of middle cerebral artery (disorder)	705128004
SNOMED	Cerebral infarction due to thrombosis of middle cerebral artery	705130002
SNOMED	Transient ischaemic attack due to embolism	710575003
SNOMED	Cerebrovascular accident during surgery (disorder)	716051003
SNOMED	Livedo reticularis and cerebrovascular accident syndrome (disorder)	716745004
SNOMED	Late effects of cerebral ischemic stroke	723083001
SNOMED	Cerebral ischemic stroke due to small artery occlusion	724424009
SNOMED	Cerebral ischemic stroke due to intracranial large artery atherosclerosis	724425005
SNOMED	Cerebral ischemic stroke due to extracranial large artery atherosclerosis	724426006
SNOMED	Stroke co-occurrent with migraine	724429004
SNOMED	Epilepsy due to perinatal stroke	724785007
SNOMED	Epilepsy due to cerebrovascular accident (disorder)	724787004
SNOMED	Cerebral ischemic stroke due to occlusion of extracranial large artery	724993002
SNOMED	Cerebral ischemic stroke due to stenosis of extracranial large artery	724994008
SNOMED	Ischemic stroke without residual deficits	725132001
SNOMED	Multifocal cerebral infarction due to and following procedure on cardiovascular system (disorder)	733199002
SNOMED	Acute cerebrovascular accident due to occlusion of right cerebellar artery (disorder)	738478141000119000
SNOMED	Personal history of transient ischaemic attack (situation)	751371000000107

SNOMED	[V]Personal history of transient ischaemic attack	757411000000101
SNOMED	Acute cerebrovascular accident due to ischemia	762005171000119000
SNOMED	Acute cerebrovascular accident due to occlusion of left cerebellar artery	788310011000119000
SNOMED	Cerebral ischemic stroke due to dissection of artery	788880006
SNOMED	Cerebral ischemic stroke due to aortic arch embolism	788881005
SNOMED	Cerebral ischemic stroke due to global hypoperfusion with watershed infarct	788882003
SNOMED	Cerebral ischemic stroke due to hypercoagulable state	788883008
SNOMED	Cerebrovascular accident, NOS	82797006
SNOMED	Carotid territory transient ischaemic attack	857261000000101
SNOMED	[X]Cerebral infarction due to unspecified occlusion or stenosis of precerebral arteries (disorder)	882261000000110
SNOMED	[V]Personal history of cerebrovascular accident (CVA)	898491000000116
SNOMED	[X]Cerebral infarction due to unspecified occlusion or stenosis of cerebral arteries	899921000000119
SNOMED	[X]Other cerebral infarction (disorder)	900181000000117
SNOMED	Acute cerebrovascular accident due to embolism of basilar artery	915141931000119000
SNOMED	Sequela of thrombotic stroke	91601000119109
SNOMED	Weakness of extremities as sequela of stroke	92341000119107
SNOMED	Acute cerebrovascular accident due to embolism of right middle cerebral artery	931164671000119000
SNOMED	Acute cerebrovascular accident due to embolism of left middle cerebral artery	936648941000119000
SNOMED	Brain stem infarction	95457000
SNOMED	Old cerebral infarction on imaging	955491000000106
SNOMED	Old cerebral infarction on imaging	965091000000107
SNOMED	History of parietal cerebrovascular accident	97531000119106
SNOMED	Occlusion of cerebral artery with stroke	9901000119100
SNOMED	History of lacunar cerebrovascular accident (situation)	99051000119101
SNOMED	Cerebral infarction due to stenosis of carotid artery	99451000119105
SNOMED	[V]Personal history of cerebrovascular accident (CVA)	996341000000110
Read	Pure sensory lacunar syndrome	G666.
Read	Spontaneous subarachnoid haemorrhage	XE2bF
Read	Cerebral arterial occlusion	G64..
Read	Lobar cerebral haemorrhage	X00DM
Read	Cerebral atherosclerosis	XE0VL
Read	Non-traumatic intracranial subdural haematoma	Xa1uU
Read	[X]Other cerebral infarction	Gyu64

Read	Multiple unruptured cerebral aneurysms	Xa00W
Read	Other specified cerebrovascular disease	G6y..
Read	Unruptured aneurysm of basilar artery	Xa01Z
Read	Cerebellar infarction	Xa00J
Read	Cerebral lupus	X00Dx
Read	Anterior circulation stroke of uncertain pathology	X00DS
Read	Transient cerebral ischaemia NOS	G65zz
Read	Transient cerebral ischaemia NOS	G65z.
Read	Brainstem infarction	G64z0
Read	Stroke/transient ischaemic attack monitoring telephone invte	XaMGv
Read	Subarachnoid haemorrhage from intracranial artery, unspecified	G60X.
Read	Thrombosis of central nervous system venous sinus NOS	F051z
Read	Benedict syndrome	Xa00L
Read	Occlusion and stenosis of middle cerebral artery	G6770
Read	Multi-infarct state	X00DG
Read	Right sided cerebral infarction	XaBED
	Wallenberg syndrome	Xa00M
Read	Cerebral venous thrombosis of lateral sinus	F0512
Read	Intracerebral haemorrhage NOS	G61z.
Read	Cerebral embolism	G641.
Read	Sequelae of intracerebral haemorrhage	G681.
Read	Precerebral arterial occlusion	XE0VG
Read	[X]Other cerebrovascular disorders in diseases classified elsewhere	Gyu6A
Read	Unruptured aneurysm of posterior communicating artery	Xa01Y
Read	Hypertensive encephalopathy	G672.
Read	Brain stem stroke syndrome	G663.
Read	Infarction of basal ganglia	XaJgQ
Read	Intracranial haemorrhage NOS	G62z.
Read	Diffuse cerebrovascular disease	Xa9Bq
Read	Posterior cerebral circulation infarction	X00D8
Read	Claude syndrome	Xa00Q
Read	Late effects of cerebrovascular disease	G68..
Read	Sequelae of other and unspecified cerebrovascular diseases	G68W.
Read	Cerebral infarct due to thrombosis of precerebral arteries	G63y0
Read	Sequelae of subarachnoid haemorrhage	G680.
Read	Top of basilar syndrome	Xa00R
Read	Transient cerebral ischaemia	G65..
Read	Ruptured aneurysm of posterior inferior cerebellar artery	Xa0NS
Read	Posterior cerebral circulation haemorrhagic infarction	X00DK
Read	Subdural haemorrhage - nontraumatic	G621.
Read	Unruptured aneurysm of posterior inferior cerebellar artery	Xa01a
Read	Unruptured aneurysm of anterior communicating artery	Xa01X
Read	Left sided intracerebral haemorrhage, unspecified	XaBM4
Read	Sequelae of cerebral infarction	G683.
Read	Total anterior cerebral circulation infarction	X00D6
Read	Transient ischaemic attacks	XaEGK
Read	Angiogram-negative subarachnoid haemorrhage	Xa0N7
Read	Right sided intracerebral haemorrhage, unspecified	XaBM5
Read	[X]Occlusion and stenosis of other precerebral arteries	Gyu65
Read	Ruptured aneurysm of posterior communicating artery	Xa0NQ
Read	Cerebral infarction due to thrombosis of cerebral arteries	G6400
Read	Ruptured aneurysm of posterior cerebral artery	Xa0NN

Read	External capsule haemorrhage	G616.
Read	Asymptomatic cerebrovascular disease	X00E4
Read	Nonpyogenic venous sinus thrombosis	G676.
Read	Unruptured internal carotid-anterior communicating artery zone aneurysm	Xa0NH
Read	Cerebral infarction due to embolism of precerebral arteries	G63y1
Read	Multiple lacunar infarcts	XaB4Z
Read	Multi-infarct dementia	Xa0lH
Read	Subdural haemorrhage NOS	G623.
Read	Brainstem infarction NOS	X00D9
Read	Pure motor lacunar syndrome	XaQbK
Read	Hypertensive encephalopathy	XE0VM
Read	Cerebral arterial occlusion	XE0VI
Read	Lacunar ataxic hemiparesis	X00DE
Read	Vertebral artery occlusion	G632.
Read	Cerebral infarction	Xa0kZ
Read	Unruptured aneurysm of anterior cerebral artery	Xa01T
Read	Subarachnoid haemorrhage	G60..
Read	Cerebral venous sinus thrombosis	F051.
Read	Cerebrovascular disease	G6...
Read	Brainstem infarction	Xa00K
Read	Weber syndrome	Xa00P
Read	Stroke/transient ischaemic attack monitoring third letter	XaJuY
Read	Generalised ischaemic cerebrovascular disease NOS	G671.
Read	Retinal microembolism	F4233
Read	Isolated angiitis of central nervous system	X00Dz
Read	Subarachnoid haemorrhage from vertebral artery	G606.
Read	Subarachnoid haemorrhage	Xa1uW
Read	Cerebellar stroke syndrome	G664.
Read	Stroke / transient ischaemic attack referral	8HBJ.
Read	Stroke/transient ischaemic attack monitoring first letter	9Om0.
Read	Intracerebral haemorrhage, multiple localized	G618.
Read	Posterior cerebral artery syndrome	G662.
Read	[V]Personal history of transient ischaemic attack	XaX16
Read	Thrombosis of superior longitudinal sinus	F0511
Read	Millard-Gubler syndrome	Xa00O
Read	Cerebellar haemorrhage	G613.
Read	Ruptured internal carotid-anterior communicating artery zone aneurysm	Xa0NV
Read	Cerebral infarction NOS	XE0VJ
Read	Retinal partial arterial occlusion NOS	F4235
Read	Unruptured internal carotid bifurcation aneurysm	Xa0NI
Read	Uncomplicated arteriosclerotic dementia	E0040
Read	Cerebral thrombosis	G640.
Read	Mixed cortical and subcortical vascular dementia	X003V
Read	Cerebral vein thrombosis	Xa6Yt
Read	Cerebral haemorrhage NOS	XE0Wy
Read	Cerebrovascular disease NOS	G6z..
Read	Cerebral parenchymal haemorrhage	XE0VF
Read	[X]Other vascular syndromes of the brain in cerebrovascular diseases	Fyu57
Read	[V]Personal history of transient ischaemic attack	ZV12D
Read	Cerebral amyloid angiopathy	X00Do
Read	Unruptured aneurysm of posterior cerebral artery	Xa01V
Read	Subcortical cerebral haemorrhage	X00DN

Read	Transient ischaemic deafness	F5802
Read	Arteriosclerotic dementia with depression	E0043
Read	Extradural haemorrhage - nontraumatic	G620.
Read	Cerebral infarction due to embolism of cerebral arteries	G6410
Read	Cerebrovascular accident	X00D1
Read	[X]Occlusion and stenosis of other cerebral arteries	Gyu66
Read	Bulbar haemorrhage	G615.
Read	Multiple and bilateral precerebral arterial occlusion	G633.
Read	Ruptured cerebral aneurysm	X204F
Read	Vertebral artery dissection	G6733
Read	Stroke / transient ischaemic attack referral	XaJkS
Read	Infarction of basal ganglia	G64z4
Read	Transient global amnesia	X00E2
Read	Cerebral atherosclerosis	G670.
Read	Other cerebrovascular disease OS	G67y.
Read	Anterior cerebral circulation infarction	X00D5
Read	H/O amaurosis fugax	14AB0
Read	Other and unspecified intracranial haemorrhage	G62..
Read	Granulomatous angiitis of central nervous system	X00E0
Read	H/O: TIA	14AB.
Read	Occlusion and stenosis of cerebral arteries, not resulting in cerebral infarction	G677.
Read	Berry aneurysm - disorder	Xa0L0
Read	Cerebral anoxia complication	SP101
Read	Intermittent cerebral ischaemia	G65z1
Read	[X]Cerebral arteritis in other diseases classified elsewhere	Gyu69
Read	Stroke/transient ischaemic attack monitoring first letter	XaJuU
Read	Suspected transient ischaemic attack	1JK..
Read	Impending cerebral ischaemia	G65z0
Read	Obstetric cerebral venous thrombosis	L417.
Read	Subarachnoid haemorrhage from carotid siphon and bifurcation	G601.
Read	H/O amaurosis fugax	XaZIY
Read	Other transient cerebral ischaemia	G65y.
Read	Occipital cerebral infarction	Xa00I
Read	Ruptured internal carotid-posterior communicating artery zone aneurysm	Xa0NW
Read	Transient ischaemic attack clinical management plan	XaQve
Read	Arteriosclerotic dementia with paranoia	E0042
Read	[X]Other intracerebral haemorrhage	Gyu62
Read	Lacunar infarction	X00DA
Read	Transient ischaemic attack	XE0VK
Read	Middle cerebral artery syndrome	G660.
Read	Wallenberg syndrome	G64z1
Read	Stroke/transient ischaemic attack monitoring verbal invitati	9Om3.
Read	Stroke/transient ischaemic attack monitoring telephone invte	9Om4.
Read	Listerial cerebral arteritis	A2703
Read	Arteriosclerotic dementia (including [multi infarct dementia])	E004.
Read	Congenital brain aneurysm NEC	P7y02
Read	Arteriosclerotic dementia with delirium	E0041
Read	Cerebral venous thrombosis in pregnancy	L4170
Read	Carotid territory transient ischaemic attack	X00DU
Read	Carotid artery dissection	G6732
Read	Cerebral haemorrhage	XM0rV
Read	Sequelae of stroke, not specified as haemorrhage or infarction	G68X.

Read	Basilar artery occlusion	G630.
Read	[X]Cerebral infarction due to unspecified occlusion or stenosis of cerebral arteries	Gyu63
Read	Stroke/transient ischaemic attack monitoring administration	XaJuH
Read	Ruptured aneurysm of middle cerebral artery	Xa0NK
Read	Occlusion and stenosis of posterior cerebral artery	G6772
Read	Thrombosis transverse sinus	F0513
Read	Anterior cerebral circulation haemorrhagic infarction	X00DJ
Read	Binswanger's disease	F21y2
Read	Ruptured internal carotid bifurcation aneurysm	Xa0NX
Read	Vertebral artery dissection	X00Dv
Read	Ruptured aneurysm of anterior communicating artery	Xa0NO
Read	Anterior cerebral artery syndrome	G661.
Read	Amaurosis fugax	F4236
Read	Infarction - precerebral	X00D4
Read	Cerebral palsy, not congenital or infantile, acute	G669.
Read	Carotid territory transient ischaemic attack	G657.
Read	Cerebral arteriosclerosis	Xa3fW
Read	Subcortical vascular dementia	X003T
Read	[X]Other transient cerebral ischaemic attacks and related syndromes	Fyu55
Read	Transient global amnesia	1B1S.
Read	Brainstem haemorrhage	X00DQ
Read	Left sided intracerebral haemorrhage, unspecified	G61X0
Read	Unruptured internal carotid-posterior communicating artery zone aneurysm	Xa0NF
Read	Subarachnoid haemorrhage from anterior communicating artery	G603.
Read	Subarachnoid haemorrhage from basilar artery	G605.
Read	Right sided CVA	G668.
Read	Vertebral artery syndrome	G651.
Read	Subarachnoid haemorrhage NOS	G60z.
Read	Occlusion and stenosis of multiple and bilateral cerebral arteries	G6774
Read	Cortical haemorrhage	G610.
Read	Internal capsule haemorrhage	G611.
Read	Cerebral aneurysm, nonruptured	G673.
Read	Arteriosclerotic dementia NOS	E004z
Read	Pontine haemorrhage	G614.
Read	Syphilitic cerebral arteritis	X00E1
Read	Cerebral palsy, not congenital or infantile, acute	XaBE2
Read	Cerebral lupus	N0006
Read	Reversible cerebral vasoconstriction syndrome	G67B.
Read	Pure sensory lacunar infarction	X00DC
Read	Reversible cerebral vasoconstriction syndrome	XaaJd
Read	Other cerebrovascular disease	G67..
Read	Right sided intracerebral haemorrhage, unspecified	G61X1
Read	Stroke/transient ischaemic attack monitoring second letter	XaJuX
Read	Cerebral amyloid angiopathy	G6740
Read	Transient ischaemic attack clinical management plan	8CRB.
Read	Occlusion and stenosis of anterior cerebral artery	G6771
Read	Subdural haematoma - nontraumatic	G622.
Read	(Transient ischaemic attacks) or (vertebro-basilar insufficiency) or (drop attacks)	XE0X0
Read	Pure sensory lacunar syndrome	XaQbM
Read	Haemorrhagic cerebral infarction	X00DI
Read	Stroke/transient ischaemic attack monitoring verbal invitati	XaKba
Read	Carotid artery occlusion	G631.

Read	Reversible ischaemic neurological defect	X00DY
Read	Pure sensorimotor lacunar infarction	X00DD
Read	Left sided cerebral infarction	G64z2
Read	Acute cerebrovascular insufficiency NOS	G6710
Read	Basilar artery syndrome	G650.
Read	Embolus of circle of Willis	Xa6YV
Read	Precerebral artery occlusion NOS	G63z.
Read	CVA - cerebrovascular accident due to cerebral artery occlusion	X00D3
Read	Subarachnoid haemorrhage from middle cerebral artery	G602.
Read	Foville syndrome	Xa00N
Read	Dissection of cerebral arteries, nonruptured	G6730
Read	Anoxic encephalopathy	F281.
Read	Cerebral infarction NOS	G64z.
Read	Cerebral venous thrombosis of cavernous sinus	F0510
Read	Carotid artery syndrome hemispheric	G653.
Read	Subarachnoid haemorrhage from posterior communicating artery	G604.
Read	[X]Intracerebral haemorrhage in hemisphere, unspecified	Gyu6F
Read	Stroke of uncertain pathology	X00DR
Read	Sequelae of other nontraumatic intracranial haemorrhage	G682.
Read	Ruptured aneurysm of anterior cerebral artery	Xa0NJ
Read	Massive supratentorial cerebral haemorrhage	X00DL
Read	Vertebro-basilar artery syndrome	G6510
Read	Pure motor lacunar infarction	X00DB
Read	Multiple and bilateral precerebral artery syndromes	G654.
Read	Vertebrobasilar insufficiency	G656.
Read	Transient global amnesia	G655.
Read	Unruptured aneurysm of middle cerebral artery	Xa01U
Read	Cerebral arteritis	G674.
Read	Precerebral arterial occlusion	G63..
Read	Drop attacks in middle-aged woman	X00E3
Read	Stroke/transient ischaemic attack monitoring administration	9Om..
Read	Ruptured berry aneurysm	G600.
Read	Stroke/transient ischaemic attack monitoring third letter	9Om2.
Read	[X]Other vascular dementia	Eu01y
Read	Subdural haemorrhage NOS	XaKK3
Read	Hollenhorst plaque	F4234
Read	Extension of cerebrovascular accident	Xa1hE
Read	Small vessel cerebrovascular disease	G679.
Read	Basal nucleus haemorrhage	G612.
Read	Pure motor lacunar syndrome	G665.
Read	Moyamoya disease	G675.
Read	Right sided cerebral infarction	G64z3
Read	Carotid artery occlusion	XE0VH
Read	[X]Vascular dementia, unspecified	Eu01z
Read	Lobar cerebral haemorrhage	G619.
Read	Stroke and cerebrovascular accident unspecified	G66..
Read	Carotid artery stenosis	G634.
Read	Cerebral venous thrombosis in the puerperium	L4171
Read	Chronic cerebral ischaemia	G6711
Read	Cerebrovascular: [disease] or [accident]	XE0Ww
Read	Cerebral autosomal dominant arteriopathy with subcortical infarcts and leukoencephalopathy	XaIRJ
Read	Other precerebral artery occlusion	G63y.

Read	Vascular dementia	XE1Xs
Read	Left sided cerebral infarction	XaBEC
Read	[X]Cerebral infarction due to unspecified occlusion or stenosis of precerebral arteries	Gyu6G
Read	Subclavian steal syndrome	G652.
Read	Cerebral aneurysm	X204A
Read	Left sided CVA	G667.
Read	Lacunar haemorrhage	X00DP
Read	Small vessel cerebrovascular disease	XaQZG
Read	Cerebral infarction due to cerebral venous thrombosis, nonpyogenic	G6760
Read	[X]Other specified cerebrovascular diseases	Gyu67
Read	Vascular dementia of acute onset	X003R
Read	Stroke NOS	XaEGq
Read	Intracerebral haemorrhage, intraventricular	G617.
Read	Cerebral vein thrombosis	G67A.
Read	Other cerebrovascular disease NOS	G67z.
Read	Cerebral autosomal dominant arteriopathy with subcortical infarcts and leukoencephalopathy	G678.
Read	Stroke and cerebrovascular accident unspecified	XE2aB
Read	Retinal transient arterial occlusion NOS	F4237
Read	Dysarthria-clumsy hand syndrome	X00DF
Read	Stroke/transient ischaemic attack monitoring second letter	9Om1.
Read	Generalised ischaemic cerebrovascular disease NOS	G671z
Read	Cerebral infarction due to unspecified occlusion or stenosis of cerebral arteries	G6X..
Read	Occlusion and stenosis of cerebellar arteries	G6773
Read	Vertebrobasilar insufficiency	X00DW
Read	Vertebrobasilar territory transient ischaemic attack	X00DV
Read	(Cerebral infarct) or (cerebrovascular accident) or (undefined stroke/CVA) or (stroke NOS)	XE0X2
Read	Carotico-cavernous sinus fistula	G6731
Read	Carotid artery dissection	X00Du
Read	Cerebral infarction due to unspecified occlusion or stenosis of precerebral arteries	G6W..
Read	Intracerebral haemorrhage in hemisphere, unspecified	G61X.
Read	Intracerebral haemorrhage	G61..
Read	Non-traumatic extradural intracranial haematoma	Xa1uV
Read	Posterior circulation stroke of uncertain pathology	X00DT
Read	Partial anterior cerebral circulation infarction	X00D7
Read	[X]Dementia: [multi-infarct] or [predominantly cortical]	Eu011
Read	Ruptured aneurysm of basilar artery	Xa0NR
Read	Suspected transient ischaemic attack	XaXNG
Read	Anoxic brain damage complication	SP100
Read	[X]Cerebral arteritis in infectious and parasitic diseases	Gyu68
Read	Thalamic haemorrhage	X00DO
Read	Intracerebellar and posterior fossa haemorrhage	Xa0Bj
Read	Cerebral aneurysm (& [non-ruptured]) or cerebrovascular insufficiency	XE0X4
Gastro-oesophageal reflux disease		
Read	Reflux cough	171J.
Read	Indigestion symptoms	195..
Read	Indigestion	1954
Read	Heartburn	1955
Read	Gastric reflux	1957
Read	Undiagnosed dyspepsia	1958
Read	Indigestion symptom NOS	195Z.
Read	Transthor antireflux fundoplicn (& [Belsey] or [Nissen])	760L0
Read	Transabdominal antireflux fundoplication (& Nissen)	760L2

Read	Antireflux operatn: [gastropexy (& Hill)] or [gastroplasty]	760L3
Read	Antireflux procedure and gastroplasty HFQ	760L4
Read	Discharged from care of dyspepsia specialist nurse	8Hg5.
Read	Referral to dyspepsia specialist nurse	8Hl0.
Read	Under care of dyspepsia specialist nurse	9NNK.
Read	Psychogenic dyspepsia	E2644
Read	Acid reflux &/or oesophagitis	J1011
Read	Gastro-oesophageal reflux disease with ulceration	J1020
Read	Oesophageal reflux (& [without mention of oesophagitis])	J10y4
Read	Laryngopharyngeal reflux	J10y5
Read	Dyspepsia (& [flatulent] or [NOS])	J16y4
Read	Non-ulcer dyspepsia	J16yA
Read	[D]Heartburn	R071.
Read	[D]Heartburn NOS	R071z
Read	Gastric reflux	Ua1kQ
Read	Pepcid AC indigestion tablet	x02AL
Read	Galpharm Heartburn Relief	x05uG
Read	Antireflux prosthesis procedure	X20UY
Read	Replacement of implanted antireflux prosthesis	X20UZ
Read	Gastro-oesophageal reflux disease	X3003
Read	Disorder of implanted oesophageal antireflux device	X301A
Read	Infection of oesophageal antireflux device	X301C
Read	Non-ulcer dyspepsia	X301d
Read	Duodenogastric reflux	X302S
Read	Extent of reflux	X75PS
Read	Oesophageal reflux observation	X76cn
Read	Indigestion NOS	X76d4
Read	Flatulent dyspepsia	X76d5
Read	Gastro-oesophageal reflux status	X77H0
Read	Acid reflux status	X77H1
Read	Venous reflux flow	X77Ug
Read	Drug-induced dyspepsia	Xa7MX
Read	Moderate gastric reflux	Xa7Ta
Read	Minimal gastric reflux	Xa7Tb
Read	No gastric reflux	Xa7TY
Read	Excessive gastric reflux	Xa7TZ
Read	Adjustment of implanted antireflux prosthesis	Xa8bS
Read	Removal of implanted antireflux prosthesis	Xa8bT
Read	Insertion of antireflux prosthesis	XaDt8
Read	Laryngopharyngeal reflux	XaK99
Read	Discharged from care of dyspepsia specialist nurse	XaKbv
Read	Under care of dyspepsia specialist nurse	XaKbw
Read	Reflux cough	XaLCS
Read	Undiagnosed dyspepsia	XaLRu
Read	Referral to dyspepsia specialist nurse	XaMDx
Read	Gastro-oesophageal reflux disease with oesophagitis	XE0aL
Read	Gastro-oesophageal reflux disease without oesophagitis	XE0aO
Read	Alkaline reflux gastritis	XE0aT
Read	Oesophagitis (& [reflux]) or oesophageal reflux	XE0bv
Read	Antireflux fundoplication using abdominal approach	XE0Ca
Read	Antireflux gastropexy	XE0Cb
Read	Antireflux fundoplication using thoracic approach	XE0CZ

Chronic kidney disease		
Read	[EDTA]Cong renal dysplasia+or-urin tract malform+ren failure	XM19J
Read	CKD with GFR category G4 & albuminuria category A1	1Z1a.
Read	[EDTA] Cystinosis associated with renal failure	XM18x
Read	[EDTA]Pyelo/interst nephritis+urolithiasis+renal failure	XM1AS
Read	[EDTA]Pyelo/interst nephritis due to OS cause+renal failure	XM1AR
Read	CKD with GFR category G2 & albuminuria category A2	1Z1R.
Read	[EDTA]Wegener granulomatosis associated with renal failure	XM19u
Read	End stage renal failure	K050.
Read	[EDTA]Renal vasc dis-classified associated+renal failure	XM19B
Read	Chronic kidney disease stage 3A with proteinuria	1Z1D.
Read	[EDTA]Cystic kidney dis-other specified type+renal failure	XM19b
Read	[EDTA]Other identified specified renal disorders+renal fail	XM19W
Read	Compensation for renal failure	XE0Jf
Read	Chronic kidney disease stage 2	XaLHH
Read	[EDTA] Fabrys disease associated with renal failure	XM19t
Read	Chronic kidney disease stage 3A without proteinuria	XaO3w
Read	[EDTA] Kidney tumour associated with renal failure	XM19q
Read	CKD with GFR category G1 & albuminuria category A1	1Z1M.
Read	CKD with GFR category G2 & albuminuria category A1	XacA4
Read	[EDTA]Congenital renal hypoplasia-unspecified+renal failure	XM19I
Read	CKD with GFR category G3a & albuminuria category A2	XacAN
Read	Chronic kidney disease stage 1	K051.
Read	[EDTA] Balkan nephropathy associated with renal failure	XM19e
Read	Post-renal renal failure	Xa85t
Read	Dialysis disequilibrium	X30Lr
Read	[EDTA]Polycystic kidneys,infantile (recessive)+renal failure	XM19K
Read	Renal failure-associated hyperphosphataemia	C3536
Read	CKD with GFR category G4 & albuminuria category A1	XacAb
Read	[EDTA]Pyelo/interst nephrit due to ves-ureter reflux+RF	XM1AT
Read	Chronic kidney disease stage 3B	XaNbo
Read	[EDTA]Multi-system disease-oth spec with renal failure	XM19h
Read	Chronic kidney disease stage 5	XaLHK
Read	EDTA Renal vascular disease-type unspecified&renal failure	XM19C
Read	[V]Renal dialysis status	ZV451
Read	[EDTA]Nephropathy due to cyclosporin A with renal failure	XM19p
Read	[EDTA]Glomerulonephritis,histolog exam OS+renal failure	XM1AL
Read	[EDTA] Chronic renal failure, aetiology uncertain	XM19A
Read	Hypertensive renal disease with renal failure	G222.
Read	Intestinal dialysis	8882
Read	Chronic kidney disease stage 3 with proteinuria	1Z1B.
Read	Hypertensive heart and renal disease with renal failure	G233.
Read	Chronic kidney disease stage 5 with proteinuria	1Z1K.
Read	[EDTA]Cortical or tubular necrosis associated+renal failure	XM199
Read	[EDTA]Renal vasc dis due to malig hypertension+renal failure	XM19E
Read	Prerenal renal failure	XM08q
Read	Disorder associated with dialysis	SP0H.
Read	[EDTA]Renal vascular dis due to hypertension+renal failure	XM19D
Read	[EDTA] Lead-induced nephropathy associat with renal failure	Xa33f
Read	Chronic kidney disease stage 4 without proteinuria	1Z1J.
Read	Chronic kidney disease stage 3A	XaNbn
Read	Chronic kidney disease stage 3 without proteinuria	XaO3u

Read	Chronic kidney disease stage 3B without proteinuria	XaO3y
Read	Congenital renal failure	Q48y0
Read	Chronic kidney disease stage 3A with proteinuria	XaO3v
Read	[EDTA]Tubulo-interstitial nephritis (not PN)+renal failure	Xa33e
Read	Chronic kidney disease stage 1 with proteinuria	1Z17.
Read	Chronic kidney disease stage 2 without proteinuria	XaO3s
Read	CKD with GFR category G1 & albuminuria category A3	1Z1P.
Read	Renal impairment	K060.
Read	Chronic kidney disease stage 2	1Z11.
Read	[EDTA] Diabetes Type II associated with renal failure	XM19j
Read	Chronic kidney disease stage 3	XaLHI
Read	Chronic kidney disease stage 1	XaLHG
Read	Renal failure as a complication of care	XE1pB
Read	[EDTA]Sev nephr syndrome+focal scler (paed only)+renal fail	XM196
Read	End stage renal failure on dialysis	X30J2
Read	CKD with GFR category G3b & albuminuria category A1	XacAV
Read	[EDTA]Cryoglobulineme glomerulonephritis+renal failure	Xa33h
Read	CKD with GFR category G5 & albuminuria category A1	XacAf
Read	CKD with GFR category G5 & albuminuria category A3	XacAi
Read	Chronic kidney disease stage 4	1Z13.
Read	Chronic kidney disease stage 3B with proteinuria	XaO3x
Read	CKD with GFR category G4 & albuminuria category A2	XacAd
Read	CKD with GFR category G3a & albuminuria category A1	XacAM
Read	CKD with GFR category G2 & albuminuria category A2	XacA6
Read	[X]Renal failure	Kyu2.
Read	CKD with GFR category G1 & albuminuria category A1	Xac9y
Read	CKD with GFR category G2 & albuminuria category A3	1Z1S.
Read	Chronic kidney disease stage 4	K054.
Read	[EDTA] Lupus erythematosus associated with renal failure	XM197
Read	Chronic kidney disease stage 3 with proteinuria	XaO3t
Read	CKD with GFR category G1 & albuminuria category A2	1Z1N.
Read	CKD with GFR category G4 & albuminuria category A2	1Z1b.
Read	CKD with GFR category G3a & albuminuria category A2	1Z1V.
Read	Renal failure following abortive pregnancy	L093.
Read	Chronic kidney disease stage 1 with proteinuria	XaO3p
Read	Chronic kidney disease stage 5	1Z14.
Read	CKD with GFR category G5 & albuminuria category A2	XacAh
Read	Anaemia secondary to chronic renal failure	XaCLy
Read	H/O: chronic kidney disease	XaXTz
Read	Hyperten heart&renal dis+both(congestv)heart and renal fail	G234.
Read	CKD with GFR category G5 & albuminuria category A3	1Z1f.
Read	Renal failure-associated hyperphosphataemia	X40RM
Read	Chronic kidney disease stage 3 without proteinuria	1Z1C.
Read	Congenital renal failure	XE1fE
Read	[EDTA]IgA nephrop excl Henoch-Schonlein purpur+renal failure	XM19X
Read	[EDTA] Myelomatosis associated with renal failure	XM19m
Read	CKD with GFR category G2 & albuminuria category A3	XacA9
Read	CKD with GFR category G3b & albuminuria category A2	XacAW
Read	Chronic kidney disease stage 1 without proteinuria	XaO3q
Read	[EDTA]Dense deposit dis,memb-prolif GN type II+renal failure	XM19Z
Read	Chronic kidney disease stage 2 with proteinuria	1Z19.
Read	CKD with GFR category G3a & albuminuria category A1	1Z1T.

Read	Renal failure unspecified	K06..
Read	CKD with GFR category G1 & albuminuria category A3	XacA2
Read	CKD with GFR category G2 & albuminuria category A1	1Z1Q.
Read	Acute-on-chronic renal failure	X30Iz
Read	Renal function impairment with growth failure	K08y3
Read	[EDTA] Scleroderma associated with renal failure	XM19k
Read	CKD with GFR category G3a & albuminuria category A3	1Z1W.
Read	CKD with GFR category G3b & albuminuria category A1	1Z1X.
Read	Chronic kidney disease stage 3A	1Z15.
Read	[EDTA] Primary oxalosis associated with renal failure	XM18y
Read	CKD with GFR category G5 & albuminuria category A2	1Z1e.
Read	[EDTA]Pyelo/interstit nephritis due to acq obstr+renal fail	XM1AP
Read	CKD with GFR category G1 & albuminuria category A2	Xac9z
Read	[EDTA]Pyelo/interst nephritis+cong+obstr+uropathy +/-VUR+RF	XM1AQ
Read	[EDTA]Diabetes Type I associated with renal failure	XM19i
Read	Chronic kidney disease stage 4 with proteinuria	XaO3z
Read	[EDTA]Nephropathy due to cis-plat with renal failure	XM19o
Read	[X]Other chronic renal failure	Kyu21
Read	CKD with GFR category G5 & albuminuria category A1	1Z1d.
Read	[EDTA]Nephrocalcinosis+hypercalcaemic nephrop+renal failure	XM19f
Read	Compensation for renal failure	7L1A.
Read	Dialysis disequilibrium	SP0H0
Read	End stage renal failure with renal transplant	X30J3
Read	[EDTA]Heredit nephritis+nerve deaf (Alports)+ renal failure	XM19O
Read	Chronic kidney disease stage 3B	1Z16.
Read	[EDTA]Hereditary nephropathy OS assoc with renal failure	XM19r
Read	[EDTA] Goodpastures syndrome associated with renal failure	XM194
Read	End stage renal failure	X30J0
Read	Chronic kidney disease stage 3B with proteinuria	1Z1F.
Read	CKD with GFR category G3a & albuminuria category A3	XacAO
Read	Chronic kidney disease stage 3B without proteinuria	1Z1G.
Read	Chronic kidney disease stage 4 with proteinuria	1Z1H.
Read	Dependence on renal dialysis	8DD..
Read	Dependence on renal dialysis	Xa9zl
Read	Renal impairment	X30Il
Read	CKD with GFR category G3b & albuminuria category A2	1Z1Y.
Read	[EDTA] Membranous nephropathy associated with renal failure	XM198
Read	Renal failure as a complication of care	SP154
Read	Chronic kidney disease stage 3	1Z12.
Read	[EDTA]Glomerulonephritis,histol not exam with renal failure	XM1AM
Read	CKD with GFR category G4 & albuminuria category A3	XacAe
Read	Chronic kidney disease stage 5 with proteinuria	XaO41
Read	Chronic kidney disease stage 4	XaLHJ
Read	[EDTA]Tuberculosis of renal tract associated+renal failure	XM18w
Read	[EDTA]Oligomeganephr hypoplas associated with renal failure	XM1AE
Read	[EDTA]Pyelonephrit/interst nephrit cause not spec+renal fail	XM1AO
Read	[EDTA]Nephropathy caused by other spec drug+renal failure	XM19n
Read	End stage renal failure, untreated by RRT	X30J1
Read	CKD with GFR category G3b & albuminuria category A3	1Z1Z.
Read	Chronic renal impairment	1Z1..
Read	[EDTA]Prune belly syndrome associated with renal failure	XM19N
Read	[EDTA]Henoch-Schonlein purpura with renal failure	XM19l

Read	[EDTA]Medullary cyst dise,incl nephronophthis+renal failure	XM19M
Read	Hypertensive heart&renal dis wth (congestive) heart failure	G232.
Read	Chronic renal failure	K05..
Read	[EDTA]Cresc glomerulonephritis (type I,II,III)+renal failure	Xa33d
Read	Chronic kidney disease stage 5 without proteinuria	1Z1L.
Read	Chronic kidney disease stage 2	K052.
Read	Chronic kidney disease stage 3	K053.
Read	Chronic kidney disease stage 5	K055.
Read	Chronic kidney disease stage 5 without proteinuria	XaO42
Read	Acute-on-chronic renal failure	K0E..
Read	Chronic renal impairment	X30In
Read	[EDTA]Ischaemic renal disease/cholest embolism+renal failure	Xa33g
Read	Chronic renal failure	XE0df
Read	[EDTA]Cystic kidney disease-type unspecified+renal failure	XM19c
Read	Chronic kidney disease stage 1	1Z10.
Read	Chronic kidney disease stage 4 without proteinuria	XaO40
Read	Chronic kidney disease stage 1 without proteinuria	1Z18.
Read	Chronic kidney disease stage 3A without proteinuria	1Z1E.
Read	[EDTA]Polycystic kidneys,adult type (dominant)+renal failure	XM19L
Read	[EDTA]Haemolyt uraemic synd incl Moschcowitz syn+renal fail	XM190
Read	[EDTA]Hereditary/familial nephropathy unspec+renal failure	XM19s
Read	CKD with GFR category G4 & albuminuria category A3	1Z1c.
Read	Chronic kidney disease stage 2 without proteinuria	1Z1A.
Read	Disorder associated with dialysis	X30Lp
Read	[EDTA]Renal vascular dise due to polyarteritis+renal failure	XM19v
Read	[EDTA] Pyelonephritis/interstitial nephritis+renal failure	Xa1uE
Read	[EDTA]Focal seg glomerulosc+nephr synd,adults+renal failure	Xa33c
Read	Anaemia secondary to chronic renal failure	D2150
Read	CKD with GFR category G3b & albuminuria category A3	XacAX
Read	[EDTA]Pyeloneph/interst nephritis+neurog bladder+renal fail	XM1AN
Read	Chronic kidney disease stage 2 with proteinuria	XaO3r
Read	Renal failure unspecified	XE0dg
SNOMED	Chronic kidney disease stage 1 with proteinuria (disorder)	3.24121E+14
SNOMED	Chronic kidney disease with glomerular filtration rate category G3a and albuminuria category A3 (disorder)	9.49921E+14
SNOMED	Chronic kidney disease stage 4 due to type 1 diabetes mellitus (disorder)	9.0751E+13
SNOMED	Chronic kidney disease stage 2 (disorder)	431856006
SNOMED	Chronic kidney disease stage 1 (disorder)	431855005
SNOMED	Chronic kidney disease stage 5 without proteinuria (disorder)	3.24541E+14
SNOMED	Chronic kidney disease with glomerular filtration rate category G4 and albuminuria category A2 (disorder)	9.50211E+14
SNOMED	Hypertensive heart AND chronic kidney disease stage 4 (disorder)	9.6721E+13
SNOMED	Chronic kidney disease with glomerular filtration rate category G3a and albuminuria category A1 (disorder)	9.49881E+14
SNOMED	Chronic kidney disease with glomerular filtration rate category G5 and albuminuria category A3 (disorder)	9.50311E+14
SNOMED	Chronic kidney disease stage 1 due to hypertension (disorder)	1.17681E+14
SNOMED	Chronic kidney disease stage 3 due to benign hypertension (disorder)	2.84991E+14
SNOMED	Chronic kidney disease with glomerular filtration rate category G1 and albuminuria category A1 (disorder)	9.49401E+14
SNOMED	Chronic kidney disease stage 2 due to hypertension (disorder)	1.29181E+14
SNOMED	Chronic kidney disease with glomerular filtration rate category G3b and albuminuria category A2 (disorder)	9.50081E+14
SNOMED	Chronic kidney disease stage 1 due to type 2 diabetes mellitus (disorder)	7.51E+11
SNOMED	Chronic kidney disease with glomerular filtration rate category G2 and albuminuria category A3 (disorder)	9.49621E+14
SNOMED	Hypertensive heart AND chronic kidney disease stage 2 (disorder)	9.6741E+13
SNOMED	Chronic kidney disease with glomerular filtration rate category G3b and albuminuria category A1 (disorder)	9.50061E+14
SNOMED	Malignant hypertensive chronic kidney disease stage 4 (disorder)	2.85881E+14

SNOMED	Chronic kidney disease stage 4 due to hypertension (disorder)	1.29151E+14
SNOMED	Chronic kidney disease stage 2 due to benign hypertension (disorder)	2.84981E+14
SNOMED	Chronic kidney disease stage 4 with proteinuria (disorder)	3.24441E+14
SNOMED	Chronic kidney disease stage 3B without proteinuria (disorder)	3.24411E+14
SNOMED	Hypertensive heart AND chronic kidney disease stage 5 (disorder)	9.6711E+13
SNOMED	Chronic kidney disease stage 5 due to benign hypertension (disorder)	2.85011E+14
SNOMED	Hypertension in chronic kidney disease stage 4 due to type 2 diabetes mellitus (disorder)	1.40111E+14
SNOMED	Chronic kidney disease stage 1 due to benign hypertension (disorder)	2.84971E+14
SNOMED	Chronic kidney disease with glomerular filtration rate category G3b and albuminuria category A3 (disorder)	9.50101E+14
SNOMED	Chronic kidney disease stage 5 with proteinuria (disorder)	3.24501E+14
SNOMED	Chronic kidney disease with glomerular filtration rate category G4 and albuminuria category A3 (disorder)	9.50231E+14
SNOMED	Chronic kidney disease stage 3B with proteinuria (disorder)	3.24371E+14
SNOMED	Chronic kidney disease stage 4 due to benign hypertension (disorder)	2.85001E+14
SNOMED	Chronic kidney disease stage 3A without proteinuria (disorder)	3.24341E+14
SNOMED	Chronic kidney disease stage 1 without proteinuria (disorder)	3.24151E+14
SNOMED	Hypertension in chronic kidney disease stage 2 due to type 2 diabetes mellitus (disorder)	1.40131E+14
SNOMED	Chronic kidney disease stage 3 due to type 2 diabetes mellitus (disorder)	7.31E+11
SNOMED	Chronic kidney disease with glomerular filtration rate category G3a and albuminuria category A2 (disorder)	9.49901E+14
SNOMED	Chronic kidney disease with glomerular filtration rate category G2 and albuminuria category A2 (disorder)	9.49561E+14
SNOMED	Chronic kidney disease with glomerular filtration rate category G5 and albuminuria category A1 (disorder)	9.50251E+14
SNOMED	Chronic kidney disease stage 5 on dialysis (disorder)	714152005
SNOMED	Hypertensive heart AND chronic kidney disease stage 1 (disorder)	9.6751E+13
SNOMED	Chronic kidney disease stage 2 due to type 1 diabetes mellitus (disorder)	9.0731E+13
SNOMED	Chronic kidney disease stage 2 with proteinuria (disorder)	3.24181E+14
SNOMED	Chronic kidney disease stage 5 due to type 2 diabetes mellitus (disorder)	7.11E+11
SNOMED	Chronic kidney disease with glomerular filtration rate category G1 and albuminuria category A3 (disorder)	9.49481E+14
SNOMED	Chronic kidney disease with glomerular filtration rate category G1 and albuminuria category A2 (disorder)	9.49421E+14
SNOMED	Chronic kidney disease stage 3A (disorder)	700378005
SNOMED	Malignant hypertensive chronic kidney disease stage 5 (disorder)	1.53851E+14
SNOMED	Chronic kidney disease stage 5 due to type 1 diabetes mellitus (disorder)	9.0761E+13
SNOMED	Chronic kidney disease stage 3 (disorder)	433144002
SNOMED	Chronic kidney disease stage 5 with transplant (disorder)	714153000
SNOMED	Chronic kidney disease with glomerular filtration rate category G2 and albuminuria category A1 (disorder)	9.49521E+14
SNOMED	Chronic kidney disease stage 5 (disorder)	433146000
SNOMED	Malignant hypertensive chronic kidney disease stage 3 (disorder)	2.85871E+14
SNOMED	Chronic kidney disease stage 3 due to type 1 diabetes mellitus (disorder)	9.0741E+13
SNOMED	Anemia co-occurrent and due to chronic kidney disease stage 3 (disorder)	6.91421E+14
SNOMED	Chronic kidney disease stage 2 without proteinuria (disorder)	3.24211E+14
SNOMED	Hypertension in chronic kidney disease stage 5 due to type 2 diabetes mellitus (disorder)	1.40101E+14
SNOMED	Chronic kidney disease stage 2 due to type 2 diabetes mellitus (disorder)	7.41E+11
SNOMED	Chronic kidney disease stage 3A with proteinuria (disorder)	3.24311E+14
SNOMED	Chronic kidney disease stage 1 due to type 1 diabetes mellitus (disorder)	9.0721E+13
SNOMED	Chronic kidney disease stage 4 without proteinuria (disorder)	3.24471E+14
SNOMED	Chronic kidney disease stage 3B (disorder)	700379002
SNOMED	Chronic kidney disease stage 3 with proteinuria (disorder)	3.24251E+14
SNOMED	Hypertension in chronic kidney disease stage 3 due to type 2 diabetes mellitus (disorder)	1.40121E+14
SNOMED	Chronic kidney disease stage 4 due to type 2 diabetes mellitus (disorder)	7.21E+11
SNOMED	Malignant hypertensive chronic kidney disease stage 2 (disorder)	2.85861E+14
SNOMED	Malignant hypertensive chronic kidney disease stage 1 (disorder)	2.85851E+14
SNOMED	Chronic kidney disease with glomerular filtration rate category G5 and albuminuria category A2 (disorder)	9.50291E+14
SNOMED	Chronic kidney disease stage 4 (disorder)	431857002
SNOMED	Chronic kidney disease stage 3 without proteinuria (disorder)	3.24281E+14

SNOMED	Chronic kidney disease with glomerular filtration rate category G4 and albuminuria category A1 (disorder)	9.50181E+14
SNOMED	Hypertensive heart AND chronic kidney disease stage 3 (disorder)	9.6731E+13
SNOMED	Chronic kidney disease stage 3 due to hypertension (disorder)	1.29171E+14
SNOMED	Chronic kidney disease stage 5 due to hypertension (disorder)	1.29161E+14
Chronic obstructive pulmonary disease		
SNOMED	Asthma-chronic obstructive pulmonary disease overlap syndrome (disorder)	1.07E+16
SNOMED	End stage chronic obstructive airways disease (disorder)	1.36E+08
SNOMED	Chronic obstructive lung disease (disorder)	13645005
SNOMED	Giant bullous emphysema (disorder)	16003001
SNOMED	Chronic obstructive bronchitis (disorder)	1.85E+08
SNOMED	Chronic asthmatic bronchitis (disorder)	1.96E+08
SNOMED	Mixed simple and mucopurulent chronic bronchitis (disorder)	1.96E+08
SNOMED	Chronic bullous emphysema (disorder)	1.96E+08
SNOMED	Segmental bullous emphysema (disorder)	1.96E+08
SNOMED	Zonal bullous emphysema (disorder)	1.96E+08
SNOMED	Acute vesicular emphysema (disorder)	1.96E+08
SNOMED	Chronic emphysema caused by chemical fumes (disorder)	1.96E+08
SNOMED	Pulmonary emphysema in alpha-1 primary immunodeficiency deficiency (disorder)	2.34E+08
SNOMED	Toxic emphysema (disorder)	2.34E+08
SNOMED	Scar emphysema (disorder)	2.34E+08
SNOMED	Bullous emphysema with collapse (disorder)	2.66E+08
SNOMED	Atrophic (senile) emphysema (disorder)	2.66E+08
SNOMED	Very severe chronic obstructive pulmonary disease (disorder)	2.94E+14
SNOMED	Mild chronic obstructive pulmonary disease (disorder)	3.13E+08
SNOMED	Moderate chronic obstructive pulmonary disease (disorder)	3.13E+08
SNOMED	Severe chronic obstructive pulmonary disease (disorder)	3.13E+08
SNOMED	Unilateral emphysema (situation)	45145000
SNOMED	Congenital emphysema (disorder)	47895001
SNOMED	Occupational bronchitis (disorder)	49691004
SNOMED	Panacinar emphysema (disorder)	4981000
SNOMED	Chronic tracheobronchitis (disorder)	52571006
SNOMED	Chronic bronchitis (disorder)	63480004
SNOMED	Congenital lobar emphysema (disorder)	66987001
SNOMED	Centriacinar emphysema (disorder)	68328006
SNOMED	Mucopurulent chronic bronchitis (disorder)	74417001
SNOMED	Fetid chronic bronchitis (disorder)	84409004
SNOMED	Pulmonary emphysema (disorder)	87433001
Dementia		
Read	GDS level 4 - moderate cognitive decline	1.00901E+14
Read	[X]Unspecified dementia	XE1Z6
Read	Senile and presenile organic psychotic conditions	XE1Xr
Read	Acquired immune deficiency syndrome dementia complex	X003P
Read	Dementia monitoring third letter	XaMGI
Read	Dementia monitoring second letter	XaMGG
Read	Dementia monitoring first letter	XaMG0
Read	Dementia monitoring administration	XaMFy
Read	Lewy body disease	345095019
Read	GDS level 6 - severe cognitive decline	XaJBW
Read	Dementia annual review	4.11341E+14
Read	[X]Subcortical vascular dementia	Eu012
Read	Dementia monitoring second letter	9Ou2.
Read	Dementia advance care plan	XacLx

Read	Dementia monitoring verbal invite	XaMGJ
Read	[X]Dementia: [multi-infarct] or [predominantly cortical]	295675012
Read	Dementia monitoring telephone invite	4.65071E+14
Read	[X]Dementia: [multi-infarct] or [predominantly cortical]	Eu011
Read	Antipsychotic drug therapy for dementia	XaaiW
Read	[X]Vascular dementia of acute onset	Eu010
Read	Subcortical vascular dementia	X003T
Read	Alzheimers disease	F110.
Read	Dementia of frontal lobe type	Xa0sE
Read	Frontal lobe degeneration	Xa0sC
Read	[X]Dementia: [multi-infarct] or [predominantly cortical]	575948016
Read	Senile dementia	X00R2
Read	Semantic dementia	X003W
Read	Cognitive impairment	28E3.
Read	Language disorder of dementia	Ub1T6
Read	Non-familial Alzheimers disease of late onset	X0032
Read	Non-familial Alzheimers disease of early onset	X002z
Read	Dementia annual review	4.11351E+14
Read	Frontotemporal dementia	X0034
Read	Presenile dementia with delirium	E0011
Read	Alzheimers disease with late onset	F1101
Read	Vascular dementia of acute onset	575947014
Read	[X]Dementia in Alzheimers dis, atypical or mixed type	Eu002
Read	[X]Lewy body dementia	3.23261E+14
Read	[X]Dementia in other diseases classified elsewhere	Eu02.
Read	Lewy body disease	X003A
Read	[X]Vascular dementia	Eu01.
Read	Other antidementia drugs	1.3034E+16
Read	[X]Dementia in Alzheimers disease with early onset	Eu000
Read	Senile dementia	401917016
Read	GDS level 4 - moderate cognitive decline	XaJBU
Read	Dementia monitoring verbal invite	4.65041E+14
Read	Dementia in Alzheimers disease with early onset	X002x
Read	Senile dementia with depression	E0021
Read	Senile dementia with paranoia	E0020
Read	Senile dementia of the Lewy body type	XaE74
Read	Alcoholic dementia	Xa25J
Read	Senile dementia with delirium	E003.
Read	Binswangers disease	F21y2
Read	Assessment of psychotic and behavioural symptoms of dementia	38C13
Read	GDS level 6 - severe cognitive decline	3AE5.
Read	Other alcoholic dementia	XE1Xu
Read	Dementia monitoring first letter	9Ou1.
Read	Amyotrophic lateral sclerosis with dementia	X002m
Read	Other antidementia drugs	1.33116E+16
Read	Alzheimers disease with early onset	XaIKB
Read	Dementia monitoring	66h..
Read	[X]Vascular dementia, unspecified	Eu01z
Read	Dementia monitoring telephone invite	4.65061E+14
Read	Moderate cognitive impairment	28E1.
Read	Dementia monitoring telephone invite	9Ou5.
Read	Presenile dementia with depression	E0013

Read	Other senile/presenile dementia	535116010
Read	Senile dementia with depressive or paranoid features NOS	E002z
Read	Dementia care plan reviewed	XacIy
Read	GDS level 6 - severe cognitive decline	1.48621E+14
Read	[X]Dementia in other specified diseases classif elsewhere	Eu02y
Read	Presenile dementia NOS	294643015
Read	Dementia monitoring administration	4.64961E+14
Read	[X]Lewy body dementia	XaKyY
Read	Dementia monitoring	XaMJC
Read	Antidementia drug	473238019
Read	[X]Dementia in Alzheimers disease, unspecified	Eu00z
Read	Focal Alzheimers disease	X0033
Read	Other alcoholic dementia	401760017
Read	Dementia monitoring second letter	4.08421E+14
Read	Frontal lobe degeneration	415879012
Read	Epileptic dementia	X003Y
Read	Dementia monitoring	4.11081E+14
Read	Dementia care plan	XaaBZ
Read	Cerebral degeneration presenting primarily with dementia	Xa1GB
Read	Alcoholic dementia: [other] or [NOS]	E012.
Read	Antidementia drug	x052K
Read	Post-traumatic dementia	X003I
Read	[X]Dementia in Alzheimers disease	Eu00.
Read	Frontal lobe degeneration	278855005
Read	Vascular dementia	661373016
Read	[X]Dementia in Alzheimers disease with late onset	Eu001
Read	Dementia monitoring verbal invite	9Ou4.
Read	Subcortical vascular dementia	575949012
Read	Dementia monitoring first letter	4.08361E+14
Read	[X] Dementia: [unspecified] or [named variants (& NOS)]	Eu02z
Read	Presenile dementia NOS	E001z
Read	Dementia medication review	8BM02
Read	Alzheimers disease with late onset	XaIKC
Read	Dementia advance care plan	8CMe0
Read	Vascular dementia of acute onset	X003R
Read	Familial Alzheimers disease of late onset	X0031
Read	[X]Lewy body dementia	3.36991E+14
Read	Mixed cortical and subcortical vascular dementia	X003V
Read	[X]Lewy body dementia	Eu025
Read	Progressive aphasia in Alzheimers disease	X003G
Read	Senile dementia with depressive or paranoid features	E002.
Read	GDS level 4 - moderate cognitive decline	9.6041E+13
Read	Dementia monitoring second letter	4.65011E+14
Read	Dementia care plan	2.24803E+15
Read	GDS level 7 - very severe cognitive decline	3AE6.
Read	Dementia monitoring administration	4.64971E+14
Read	[X]Other Alzheimers disease	Fyu30
Read	Dementia annual review	4.16771E+14
Read	Alcoholic dementia: [other] or [NOS]	294665015
Read	Arteriosclerotic dementia with paranoia	E0042
Read	Dementia care plan agreed	8CMZ0
Read	Dementia care plan reviewed	2.43965E+15

Read	Other alcoholic dementia	294667011
Read	[X]Dementia in Picks disease	Eu020
Read	[X]Dementia in Parkinsons disease	Eu023
Read	[X]Dementia in Huntingtons disease	Eu022
Read	Mixed cortical and subcortical vascular dementia	575950012
Read	Severe cognitive impairment	Xaagk
Read	Senile and presenile organic psychot conditions (& dementia)	E00..
Read	Dementia monitoring	4.16511E+14
Read	Lewy body disease	345096018
Read	Frontotemporal degeneration	F118.
Read	Frontal lobe degeneration with motor neurone disease	X0039
Read	Dementia monitoring third letter	9Ou3.
Read	Dementia monitoring administration	9Ou..
Read	Dementia advance care plan	2.44549E+15
Read	Dementia advance care plan agreed	8CSA.
Read	Familial Alzheimers disease of early onset	X002y
Read	Dementia care plan reviewed	2.43964E+15
Read	GDS level 5 - moderately severe cognitive decline	3AE4.
Read	GDS level 4 - moderate cognitive decline	3AE3.
Read	Dementia (& [presenile] or [senile])	XE1aG
Read	Alcoholic dementia NOS	346929012
Read	Senile and presenile organic psychotic conditions	401757012
Read	Uncomplicated senile dementia	E000.
Read	Lewy body disease	297008013
Read	GDS level 7 - very severe cognitive decline	1.48631E+14
Read	Acquired immune deficiency syndrome dementia complex	474150013
Read	Review of dementia advance care plan	XabEl
Read	Dementia monitoring telephone invite	4.13961E+14
Read	GDS level 5 - moderately severe cognitive decline	1.44511E+14
Read	Other senile/presenile dementia	Xa3ez
Read	Wernicke-Korsakov syndrome	575175012
Read	Review of dementia advance care plan	8CMG2
Read	H/O: dementia	1461
Read	Other antidementia drugs	dB...
Read	Senile and presenile organic psychotic conditions	294633018
Read	Frontal lobe degeneration with motor neurone disease	345093014
Read	Frontotemporal degeneration	2.16754E+15
Read	Parkinsons disease - dementia complex on Guam	X003l
Read	Moderate cognitive impairment	Xaagj
Read	Arteriosclerotic dementia NOS	E004z
Read	Uncomplicated presenile dementia	E0010
Read	Dementia (& [presenile] or [senile])	535110016
Read	[X]Dementia in human immunodef virus [HIV] disease	Eu024
Read	Dementia (& [presenile] or [senile])	240874011
Read	Dementia care plan	8CMZ.
Read	Presenile dementia	575148017
Read	Dementia (& [presenile] or [senile])	401914011
Read	[X]Mixed cortical and subcortical vascular dementia	Eu013
Read	Picks disease with Pick cells and no Pick bodies	X0036
Read	Assessment of psychotic and behavioural symptoms of dementia	XaaeA
Read	Patchy dementia	X003X
Read	[X]Dementia in other specified diseases classif elsewhere	295690011

Read	Punch drunk syndrome	X003J
Read	Mixed cortical and subcortical vascular dementia	295679018
Read	Senile dementia with depressive or paranoid features NOS	294647019
Read	GDS level 4 - moderate cognitive decline	1.58781E+14
Read	Dementia care plan reviewed	8CMZ1
Read	Frontotemporal degeneration	X0037
Read	Alcoholic dementia NOS	1.47298E+15
Read	H/O: dementia	220114011
Read	Arteriosclerotic dementia with depression	E0043
Read	Wernicke-Korsakov syndrome	E0112
Read	Predominantly cortical dementia	2.7299E+15
Read	Dementia advance care plan agreed	XabEk
Read	Dementia in Alzheimers disease with late onset	X0030
Read	[X]Unspecified dementia	295703010
Read	Senile dementia	240877016
Read	Presenile dementia NOS	1.35631E+15
Read	Picks disease	F111.
Read	Dementia annual review	6AB..
Read	Dementia (& [presenile] or [senile])	661521018
Read	Dementia care plan agreed	2.4396E+15
Read	Multi-infarct dementia	9.5511E+13
Read	Uncomplicated senile dementia	240879018
Read	Presenile dementia with paranoia	E0012
Read	Dementia annual review	XaMGF
Read	Dementia in conditions EC	E041.
Read	Dementia monitoring verbal invite	4.13941E+14
Read	Dementia	X002w
Read	Drug-induced dementia	E02y1
Read	Picks disease	3785039014
Read	Participates in Butterfly Scheme for dementia	XaZWz
Read	Dementia care plan	2.24804E+15
Read	Alcoholic dementia NOS	X00Rk
Read	Antipsychotic drug therapy for dementia	8BPa.
Read	Dementia medication review	2.40312E+15
Read	GDS level 7 - very severe cognitive decline	XaJBX
Read	Dementia medication review	XabtQ
Read	Picks disease with Pick bodies	X0035
Read	Alcoholic dementia: [other] or [NOS]	575177016
Read	Arteriosclerotic dementia with delirium	E0041
Read	Anti-dementia drug therapy	XaJPy
Read	Dementia care plan agreed	XacIx
Read	Other senile/presenile dementia	426421014
Read	[X]Other vascular dementia	Eu01y
Read	Lewy body disease	F116.
Read	Vascular dementia	XE1Xs
Read	Predominantly cortical dementia	Xaghb
Read	Arteriosclerotic dementia	E004.
Read	Frontotemporal degeneration	345092016
Read	Dementia monitoring telephone invite	XaMGK
Read	Dementia monitoring verbal invite	4.65051E+14
Read	Presenile dementia	E001.
Read	Multi-infarct dementia	Xa0lH

Read	Dementia monitoring second letter	4.13911E+14
Read	[X]Dementia in Creutzfeldt-Jakob disease	Eu021
Read	[X]Delirium superimposed on dementia	Eu041
Read	GDS level 4 - moderate cognitive decline	1.76941E+14
Read	GDS level 7 - very severe cognitive decline	1.44521E+14
Read	Severe cognitive impairment	28E2.
Read	Multi-infarct dementia	9.5521E+13
Read	Dementia monitoring first letter	4.64981E+14
Read	Punch drunk syndrome	345104014
Read	Uncomplicated arteriosclerotic dementia	E0040
Read	GDS level 5 - moderately severe cognitive decline	XaJBV
Read	Alzheimers disease with early onset	F1100
SNOMED	Lewy body disease	192808003
SNOMED	Primary degenerative dementia of the Alzheimer type, presenile onset, with depression (disorder)	10532003
SNOMED	Senile or presenile psychoses NOS	E00z.
SNOMED	[X]Dementia in other specified diseases classif elsewhere (disorder)	192179008
SNOMED	Inhalant-induced persisting dementia (disorder)	764210011
SNOMED	Dementia due to prion disease (disorder)	3636472011
SNOMED	Presenile dementia with psychosis (disorder)	2.72974E+15
SNOMED	Senile dementia with depressive or paranoid features (disorder)	191457008
SNOMED	Dementia of frontal lobe type (disorder)	672068018
SNOMED	Altered behavior in dementia due to Huntington chorea (disorder)	8.2361E+13
SNOMED	[X]Lewy body dementia	2.14031E+14
SNOMED	Dementia monitoring first letter	2.51841E+14
SNOMED	Severe cognitive impairment	702956004
SNOMED	Dementia caused by volatile inhalant (disorder)	3786107010
SNOMED	Dementia paralytica	86446018
SNOMED	Dementia monitoring telephone invite	2.74861E+14
SNOMED	Psychological symptom due to dementia (finding)	3788712011
SNOMED	Dementia of frontal lobe type (disorder)	278857002
SNOMED	Dementia annual review	4.16781E+14
SNOMED	Dementia due to carbon monoxide poisoning (disorder)	3902073013
SNOMED	Senile or presenile psychoses NOS	1.37718E+15
SNOMED	Impaired cognition (finding)	386806002
SNOMED	GDS level 5 - moderately severe cognitive decline	407633008
SNOMED	Dementia due to primary malignant neoplasm of brain (disorder)	3498955014
SNOMED	Dementia monitoring second letter	7.17471E+14
SNOMED	Uncomplicated arteriosclerotic dementia (disorder)	575164013
SNOMED	[X]Dementia in Creutzfeldt-Jakob disease (disorder)	8.23481E+14
SNOMED	Predominantly cortical dementia	1.08958E+15
SNOMED	Drug-induced dementia (disorder)	575197014
SNOMED	Presenile dementia associated with acquired immunodeficiency syndrome (disorder)	2971122014
SNOMED	[X]Dementia in Creutzfeldt-Jakob disease	4.14351E+14
SNOMED	Other senile/presenile dementia (disorder)	286932008
SNOMED	GDS level 6 - severe cognitive decline	1.00921E+14
SNOMED	Primary degenerative dementia (disorder)	1.78971E+14
SNOMED	Arteriosclerotic dementia NOS (disorder)	575169015
SNOMED	Dementia monitoring verbal invite	4.08481E+14
SNOMED	[X]Other vascular dementia	4.12631E+14
SNOMED	Antipsychotic drug therapy for dementia	8.89271E+14
SNOMED	Dementia monitoring	4.11091E+14
SNOMED	Severe cognitive impairment	2.28829E+15

SNOMED	Moderate cognitive impairment	702955000
SNOMED	Mild dementia (disorder)	4.28051E+14
SNOMED	[X]Vascular dementia, unspecified (disorder)	192171006
SNOMED	Cognitive impairment	80216008
SNOMED	Alcoholic dementia NOS	294666019
SNOMED	Vascular dementia (disorder)	268613002
SNOMED	Dementia monitoring	2.48711E+14
SNOMED	[X]Dementia in Alzheimers disease, unspecified (disorder)	192164001
SNOMED	Presenile dementia (disorder)	154847008
SNOMED	Antipsychotic drug therapy for dementia	2.29044E+15
SNOMED	Aggression due to dementia (finding)	788861009
SNOMED	Subcortical vascular dementia (disorder)	230286002
SNOMED	[X]Vascular dementia, unspecified	4.13281E+14
SNOMED	Predominantly cortical dementia (disorder)	2.72978E+15
SNOMED	[X]Delirium superimposed on dementia	295714013
SNOMED	Mixed dementia (disorder)	7.9341E+13
SNOMED	Moderate cognitive impairment	8.88301E+14
SNOMED	Global Deterioration Scale level 6 - severe cognitive decline	407634002
SNOMED	Other alcoholic dementia (disorder)	1.21478E+15
SNOMED	[X]Dementia in Creutzfeldt-Jakob disease (disorder)	192175002
SNOMED	Dementia monitoring verbal invite	2.74851E+14
SNOMED	Dementia due to primary malignant neoplasm of brain (disorder)	733190003
SNOMED	Presenile dementia with psychosis (disorder)	1.0895E+15
SNOMED	AIDS with presenile dementia (disorder)	62102009
SNOMED	Moderate cognitive impairment	2.28826E+15
SNOMED	GDS level 5 - moderately severe cognitive decline	1.85391E+14
SNOMED	Dementia associated with cerebral anoxia (disorder)	2981289019
SNOMED	[X]Alcoholic dementia NOS	8.97091E+14
SNOMED	Organic dementia associated with acquired immunodeficiency syndrome (disorder)	420614009
SNOMED	Dementia monitoring second letter	2.74831E+14
SNOMED	[X]Dementia in Parkinsons disease	4.79161E+14
SNOMED	Presenile dementia with delusions (disorder)	2989150017
SNOMED	[X]Dementia in Picks disease (disorder)	192174003
SNOMED	Lewy body disease	618061019
SNOMED	Vascular dementia (disorder)	192165000
SNOMED	Focal Alzheimer disease	2839833018
SNOMED	Dementia monitoring third letter	2.51861E+14
SNOMED	Multi-infarct dementia	7.8511E+13
SNOMED	[X]Dementia in Alzheimers disease	4.43491E+14
SNOMED	Predominantly cortical vascular dementia (disorder)	2.7298E+15
SNOMED	Frontotemporal dementia with gene located on 3p11	702393003
SNOMED	Other alcoholic dementia	5.44871E+14
SNOMED	[X]Dementia in Alzheimers dis, atypical or mixed type	4.19261E+14
SNOMED	Right temporal atrophy variant frontotemporal dementia	3307152013
SNOMED	Dementia associated with multiple sclerosis (disorder)	2975065017
SNOMED	Familial Alzheimers disease of late onset (disorder)	230267005
SNOMED	Senile dementia with delusion (disorder)	371024007
SNOMED	Other alcoholic dementia (disorder)	661375011
SNOMED	Presenile dementia co-occurrent with human immunodeficiency virus infection (disorder)	713488003
SNOMED	Lewy body disease	230276003
SNOMED	Behavioral and psychological symptoms of dementia	1.0171E+13
SNOMED	Dementia care plan reviewed	9.56861E+14

SNOMED	Dementia due to iron deficiency (disorder)	840465008
SNOMED	IBMPFD - Inclusion body myopathy with early onset Paget disease and frontotemporal dementia	703544004
SNOMED	Senile dementia with depressive or paranoid features NOS	1.35632E+15
SNOMED	Dementia of the Alzheimer type with behavioral disturbance (disorder)	1.2571E+13
SNOMED	Dementia associated with cerebral lipidosis (disorder)	2975096014
SNOMED	Dementia due to pellagra (disorder)	788899002
SNOMED	Epileptic dementia (disorder)	618076010
SNOMED	Ischemic vascular dementia (disorder)	3334660016
SNOMED	Dementia in Alzheimers disease with early onset (disorder)	192802002
SNOMED	Rapidly progressive dementia (disorder)	3424365012
SNOMED	Anxiety due to dementia (finding)	788866004
SNOMED	Dementia in Alzheimers disease with late onset	192162002
SNOMED	Other alcoholic dementia (disorder)	268615009
SNOMED	Dementia due to and following injury of head (disorder)	762351006
SNOMED	Lewy body disease	230277007
SNOMED	Familial Alzheimers disease of early onset (disorder)	230265002
SNOMED	Dementia monitoring	2.53111E+14
SNOMED	AIDS with organic dementia	90194001
SNOMED	Frontotemporal degeneration	2.16509E+15
SNOMED	Wernicke-Korsakov syndrome	191472007
SNOMED	Dementia in Alzheimers disease with late onset (disorder)	192803007
SNOMED	Behavioral disturbance co-occurrent and due to late onset Alzheimer dementia (disorder)	1.62192E+16
SNOMED	Dementia monitoring telephone invitation	7.16991E+14
SNOMED	Moderate cognitive impairment	8.88291E+14
SNOMED	Frontotemporal dementia with parkinsonism-17	702429008
SNOMED	Agitation due to dementia (finding)	788862002
SNOMED	Early onset Alzheimers disease with behavioral disturbance (disorder)	1.05421E+14
SNOMED	Senile dementia with depressive or paranoid features (disorder)	575157011
SNOMED	Dementia co-occurrent and due to Down syndrome (disorder)	3498964016
SNOMED	Dementia following injury caused by exposure to ionizing radiation (disorder)	3512305017
SNOMED	Lewy body disease	345094015
SNOMED	Dementia monitoring verbal invitation	1.15392E+15
SNOMED	Apathetic behavior due to dementia (finding)	3786024019
SNOMED	Dementia advance care plan review	9.12811E+14
SNOMED	Vascular dementia without behavioral disturbance (disorder)	3447363018
SNOMED	Semantic dementia	230288001
SNOMED	Arteriosclerotic dementia with delirium (disorder)	191464005
SNOMED	Protein kinase cAMP-dependent type I regulatory subunit beta-related neurodegenerative dementia with intermediate filaments (disorder)	3727869010
SNOMED	Global Deterioration Scale level 4 - moderate cognitive decline	407632003
SNOMED	Other senile/presenile dementia	1.53201E+15
SNOMED	AIDS with organic dementia	149508014
SNOMED	Cognitive impairment co-occurrent and due to human immunodeficiency virus infection	3745708015
SNOMED	Dementia monitoring verbal invite	4.13951E+14
SNOMED	Dementia due to chromosomal anomaly (disorder)	3334075013
SNOMED	[X]Delirium superimposed on dementia (disorder)	192184002
SNOMED	Uncomplicated senile dementia (disorder)	191449005
SNOMED	Antipsychotic drug therapy for dementia	2.29045E+15
SNOMED	Dementia monitoring administration	4.13861E+14
SNOMED	Dementia associated with normal pressure hydrocephalus (disorder)	698625002
SNOMED	GDS level 6 - severe cognitive decline	8.5701E+13
SNOMED	Dementia care plan agreed	9.56841E+14
SNOMED	GDS level 7 - very severe cognitive decline	9.6061E+13

SNOMED	Acquired immune deficiency syndrome dementia complex (disorder)	2728688016
SNOMED	Dementia associated with Parkinson Disease	425390006
SNOMED	Dementia due to Huntington chorea (disorder)	2820376013
SNOMED	Alzheimers neurofibrillary degeneration	85775002
SNOMED	Frontotemporal dementia with parkinsonism-17	2995187016
SNOMED	Dementia monitoring telephone invite	4.13971E+14
SNOMED	Severe cognitive impairment	8.88311E+14
SNOMED	Antipsychotic drug therapy for dementia (procedure)	2989637017
SNOMED	[X]Dementia in Alzheimers disease (disorder)	192160005
SNOMED	GDS level 7 - very severe cognitive decline	1.73371E+14
SNOMED	Patchy dementia (disorder)	618075014
SNOMED	Dementia monitoring third letter	4.65031E+14
SNOMED	Other antidementia drugs	4.3129E+15
SNOMED	Dementia advance care plan agreed	1.09512E+15
SNOMED	[X]Dementia in Huntingtons disease (disorder)	192176001
SNOMED	AIDS with dementia (disorder)	12741002
SNOMED	Familial dementia with neuroserpin inclusion bodies	3009601017
SNOMED	Dementia due to carbon monoxide poisoning (disorder)	840464007
SNOMED	Presenile dementia with bone cysts	702347001
SNOMED	[X] Unspecified dementia	9.71611E+14
SNOMED	Other senile/presenile dementia	7.00911E+14
SNOMED	Primary degenerative dementia of the Alzheimer type, senile onset (disorder)	416975007
SNOMED	Presenile dementia NOS (disorder)	575156019
SNOMED	Organic dementia associated with acquired immunodeficiency syndrome (disorder)	2971089017
SNOMED	[X] Unspecified dementia	268675002
SNOMED	[X]Dementia in other diseases classified elsewhere	295684012
SNOMED	Dementia co-occurrent and due to neurocysticercosis (disorder)	722977005
SNOMED	Diffuse Lewy body disease with spongiform cortical change	71362016
SNOMED	Drug-induced dementia (disorder)	191493005
SNOMED	Alzheimer type II glial cell (body structure)	29209006
SNOMED	Senile dementia of the Lewy body type (disorder)	312991009
SNOMED	Dementia monitoring first letter	2.74821E+14
SNOMED	Subcortical leukoencephalopathy (disorder)	90099008
SNOMED	Multi infarct dementia with delirium	2923435018
SNOMED	Lewy body dementia with behavioural disturbance	2983848019
SNOMED	Antidementia agent	413562009
SNOMED	Behavioral and psychological symptoms of dementia	3672963018
SNOMED	Subcortical vascular dementia (disorder)	618071017
SNOMED	[X]Dementia: [multi-infarct] or [predominantly cortical] (disorder)	192167008
SNOMED	Non-amnestic Alzheimer disease (disorder)	3332845017
SNOMED	Senile dementia with psychosis (disorder)	371026009
SNOMED	Dementia monitoring first letter	4.13881E+14
SNOMED	Dementia associated with alcoholism (disorder)	281004
SNOMED	Wernicke-Korsakov syndrome	294663010
SNOMED	Dementia monitoring second letter	2.49191E+14
SNOMED	Presenile dementia with delusions (disorder)	3.1081E+13
SNOMED	Post-traumatic dementia with behavioral change (disorder)	2975127014
SNOMED	Predominantly cortical dementia (disorder)	1.08952E+15
SNOMED	Senile dementia with delirium (disorder)	191461002
SNOMED	Presenile dementia NOS (disorder)	191456004
SNOMED	[X]Dementia in Huntingtons disease	4.14361E+14
SNOMED	Antidementia agent	358926001

SNOMED	Subcortical dementia (disorder)	3635491015
SNOMED	Mixed cortical and subcortical vascular dementia (disorder)	230287006
SNOMED	Dementia caused by heavy metal exposure (disorder)	3498954013
SNOMED	Dementia due to infectious disease (disorder)	3445728013
SNOMED	[X]Other vascular dementia	295680015
SNOMED	Vascular dementia (disorder)	2756042016
SNOMED	Dementia co-occurrent and due to Down syndrome (disorder)	733194007
SNOMED	Multi-infarct dementia with depression	23960010
SNOMED	Uncomplicated senile dementia (disorder)	575147010
SNOMED	Epileptic dementia with behavioral disturbance (disorder)	3290070014
SNOMED	Punch drunk syndrome (disorder)	618068013
SNOMED	Anxiety due to dementia (finding)	3786020011
SNOMED	Dementia pugilistica	51996004
SNOMED	Presenile dementia NOS	6.13591E+14
SNOMED	Severe dementia (disorder)	4.28351E+14
SNOMED	Arteriosclerotic dementia NOS	294656010
SNOMED	Behavioural variant of frontotemporal dementia	3308115011
SNOMED	Assessment of psychotic and behavioural symptoms of dementia	8.86431E+14
SNOMED	Subcortical vascular dementia (disorder)	192168003
SNOMED	Senile dementia with psychosis (disorder)	1196322019
SNOMED	Moderate cognitive impairment	2.28825E+15
SNOMED	Senile dementia with depressive or paranoid features NOS (disorder)	191460001
SNOMED	[X]Other vascular dementia (disorder)	8.14701E+14
SNOMED	Uncomplicated arteriosclerotic dementia (disorder)	191463004
SNOMED	Subcortical dementia (disorder)	762707000
SNOMED	Severe cognitive impairment	8.88321E+14
SNOMED	Dementia monitoring verbal invitation	7.16221E+14
SNOMED	Presenile dementia (disorder)	12348006
SNOMED	Alcoholic dementia: [other] or [NOS] (disorder)	191474008
SNOMED	Cortical vascular dementia (disorder)	833326008
SNOMED	Dementia due to chronic subdural hematoma (disorder)	3498959015
SNOMED	Inhalant-induced persisting dementia (disorder)	32875003
SNOMED	Dementia associated with alcoholism (disorder)	758743015
SNOMED	Senile and presenile organic psychotic conditions (& dementia) (disorder)	191448002
SNOMED	Disinhibited behavior due to dementia (disorder)	3787503014
SNOMED	Senile dementia with paranoia (disorder)	575159014
SNOMED	Dementia monitoring administration	2.48401E+14
SNOMED	[X]Other vascular dementia (disorder)	575951011
SNOMED	Senile dementia of the Lewy body type (disorder)	709724016
SNOMED	Dementia monitoring third letter	4.13931E+14
SNOMED	Wandering due to dementia (finding)	789062005
SNOMED	Dementia advance care plan	9.59371E+14
SNOMED	Dementia (& [presenile] or [senile]) (disorder)	2715415018
SNOMED	Primary degenerative dementia of the Alzheimer type, senile onset, with delirium (disorder)	4817008
SNOMED	Nuchal dystonia-dementia syndrome	48514014
SNOMED	[X]Delirium superimposed on dementia (disorder)	8.84041E+14
SNOMED	Dementia care plan agreed	9.56851E+14
SNOMED	Dementia (disorder)	52448006
SNOMED	Other senile/presenile dementia (disorder)	681102010
SNOMED	Dementia monitoring verbal invitation	2.49221E+14
SNOMED	Dementia associated with cerebral lipidosis (disorder)	698624003
SNOMED	Dementia caused by heavy metal exposure (disorder)	733184002

SNOMED	Dementia associated with acquired immunodeficiency syndrome (disorder)	421529006
SNOMED	Multi-infarct dementia (disorder)	56267009
SNOMED	Vascular dementia (disorder)	2760457012
SNOMED	Alcoholic dementia NOS (disorder)	231463001
SNOMED	X-linked ataxia dementia syndrome	719818007
SNOMED	Presenile dementia with delirium (disorder)	575150013
SNOMED	[X] Dementia: [unspecif] or [presenile NOS (including presenile psychosis NOS)] or [primary degenerative NOS] or [senile NOS (including senile psychosis NOS)] or [senile depressed or paranoid type] (disorder)	2731676017
SNOMED	Antipsychotic drug therapy for dementia (procedure)	2.29042E+15
SNOMED	Protein kinase cAMP-dependent type I regulatory subunit beta-related neurodegenerative dementia with intermediate filaments (disorder)	774069007
SNOMED	GDS level 7 - very severe cognitive decline	1.00931E+14
SNOMED	Senile and presenile organic psychotic conditions (& dementia) (disorder)	2714191011
SNOMED	Senile dementia with depression (disorder)	575160016
SNOMED	[X]Dementia: [multi-infarct] or [predominantly cortical] (disorder)	2713854010
SNOMED	Alzheimers dementia	2987126011
SNOMED	Dementia (& [presenile] or [senile]) (disorder)	268744003
SNOMED	Arteriosclerotic dementia with delirium (disorder)	575166010
SNOMED	Dementia care plan agreed	2.43961E+15
SNOMED	Senile dementia with paranoia (disorder)	191458003
SNOMED	Primary degenerative dementia of the Alzheimer type, senile onset, with behavioral disturbance (disorder)	4.29161E+14
SNOMED	Participates in Butterfly Scheme for dementia	8.43991E+14
SNOMED	Dementia advance care plan review	2.34595E+15
SNOMED	Presenile dementia (disorder)	726463015
SNOMED	Mixed cortical and subcortical vascular dementia (disorder)	192169006
SNOMED	Dementia associated with viral encephalitis (disorder)	698726009
SNOMED	Assessment of psychotic and behavioral symptoms of dementia	2990782017
SNOMED	Cortical vascular dementia (disorder)	3884947018
SNOMED	Dementia due to Creutzfeldt Jakob disease (disorder)	2690145018
SNOMED	Dementia monitoring invitation	7.11441E+14
SNOMED	[X]Lewy body dementia	1.98681E+14
SNOMED	Alzheimers dementia	1.42811E+14
SNOMED	Dementia with behavioral disturbance (disorder)	1.2651E+13
SNOMED	Ischemic vascular dementia (disorder)	723123001
SNOMED	Sedative, hypnotic AND/OR anxiolytic-induced persisting dementia (disorder)	59651006
SNOMED	GDS level 6 - severe cognitive decline	1.70671E+14
SNOMED	Other senile/presenile dementia (disorder)	1.53202E+15
SNOMED	Dementia (& [presenile] or [senile]) (disorder)	2716700013
SNOMED	Lewy body disease	618059011
SNOMED	Antidementia agent	2534708013
SNOMED	Vascular dementia of acute onset (disorder)	2728589017
SNOMED	GDS level 5 - moderately severe cognitive decline	1.00911E+14
SNOMED	Senile dementia with delirium (disorder)	154849006
SNOMED	Antidementia agent	3727032013
SNOMED	Dementia due to prion disease (disorder)	762350007
SNOMED	Dementia with behavioral disturbance (disorder)	1.591E+12
SNOMED	Behavioural variant of frontotemporal dementia	716994006
SNOMED	Behavioral disturbance co-occurrent and due to late onset Alzheimer dementia (disorder)	3512936017
SNOMED	Subcortical vascular dementia (disorder)	2722994016
SNOMED	Dementia management (regime/therapy)	1460436013
SNOMED	[X]Dementia in Alzheimers disease, unspecified	4.43541E+14
SNOMED	Dementia due to iron deficiency (disorder)	3902076017
SNOMED	Frontotemporal degeneration	8.33921E+14

SNOMED	Mixed dementia (disorder)	6.34651E+14
SNOMED	Arteriosclerotic dementia with depression (disorder)	191466007
SNOMED	Dementia associated with multiple sclerosis (disorder)	698626001
SNOMED	Dementia due to multiple sclerosis with altered behavior (disorder)	6.58891E+14
SNOMED	Dementia due to Creutzfeldt Jakob disease (disorder)	429458009
SNOMED	[X]Dementia in Alzheimers dis, atypical or mixed type (disorder)	192163007
SNOMED	Dementia due to Retts syndrome (disorder)	1.30121E+14
SNOMED	[X]Delirium superimposed on dementia (disorder)	575968010
SNOMED	Dialysis dementia (disorder)	837187013
SNOMED	Other senile/presenile dementia	240884012
SNOMED	Dementia due to pellagra (disorder)	3786109013
SNOMED	[X] Unspecified dementia	295699012
SNOMED	[X]Other vascular dementia (disorder)	192170007
SNOMED	Dementia due to metabolic abnormality (disorder)	3334073018
SNOMED	Alzheimer type II glial cell (body structure)	760078011
SNOMED	[X]Dementia in other specified diseases classified elsewhere	3.97791E+14
SNOMED	Delusions in Alzheimers disease (disorder)	1.41991E+14
SNOMED	Dementia associated with neurosyphilis (disorder)	698725008
SNOMED	Hallucinations co-occurrent and due to late onset dementia (disorder)	2.421E+12
SNOMED	Dementia associated with normal pressure hydrocephalus (disorder)	2974985014
SNOMED	Epilepsy co-occurrent and due to dementia (disorder)	3498222013
SNOMED	Dementia arising in the senium AND/OR presenium (disorder)	88339003
SNOMED	Antipsychotic drug therapy for dementia (procedure)	700214004
SNOMED	Assessment of psychotic and behavioral symptoms of dementia	700464008
SNOMED	Dementia due to multiple sclerosis with altered behavior (disorder)	8.2371E+13
SNOMED	Epileptic dementia with behavioral disturbance (disorder)	8.2381E+13
SNOMED	History of dementia (situation)	2986503016
SNOMED	Familial dementia with neuroserpin inclusion bodies	702421006
SNOMED	[X] Unspecified dementia	4.71341E+14
SNOMED	Uncomplicated senile dementia (disorder)	154846004
SNOMED	[X] (Mental and behavioural disorders due to use of alcohol: residual and late-onset psychotic disorder) or (chronic alcoholic brain syndrome [& dementia NOS]) (disorder)	192214004
SNOMED	Diffuse Lewy body disease with spongiform cortical change	42769004
SNOMED	Familial Alzheimers disease	79358009
SNOMED	Multi infarct dementia, uncomplicated	70936005
SNOMED	Dementia management (regime/therapy)	386257007
SNOMED	Non-amnestic Alzheimer disease (disorder)	722600006
SNOMED	Dementia due to infectious disease (disorder)	724777003
SNOMED	Multi-infarct dementia	1.16861E+14
SNOMED	Dementia monitoring administration	7.13821E+14
SNOMED	Dementia co-occurrent with human immunodeficiency virus infection (disorder)	713844000
SNOMED	Dementia annual review	2.53241E+14
SNOMED	Dementia monitoring first letter	7.15821E+14
SNOMED	Dementia monitoring administration	2.74811E+14
SNOMED	Right temporal atrophy variant frontotemporal dementia	716667005
SNOMED	Post-traumatic dementia with behavioral change (disorder)	698687007
SNOMED	Senile or presenile psychoses NOS	191469000
SNOMED	Dementia monitoring third letter	2.74841E+14
SNOMED	Senile dementia with depressive or paranoid features NOS (disorder)	575161017
SNOMED	Patchy dementia (disorder)	230289009
SNOMED	Lewy body disease	618060018
SNOMED	Dementia monitoring	4.16521E+14

SNOMED	Alcoholic dementia NOS (disorder)	619398019
SNOMED	Dementia associated with Parkinson Disease	2842012010
SNOMED	Dementia caused by toxin (disorder)	722978000
SNOMED	Presenile dementia with depression (disorder)	575155015
SNOMED	AIDS with presenile dementia (disorder)	801238010
SNOMED	Lewy body disease	576629011
SNOMED	Dementia co-occurrent and due to progressive multifocal leukoencephalopathy (disorder)	3474221016
SNOMED	Moderate dementia (disorder)	4.30771E+14
SNOMED	IBMPFD - Inclusion body myopathy with early onset Paget disease and frontotemporal dementia	3009710019
SNOMED	Parkinson-dementia complex of Guam	103435012
SNOMED	Delirium co-occurrent with dementia (disorder)	725898002
SNOMED	Global Deterioration Scale level 6 - severe cognitive decline	2914648017
SNOMED	Dementia monitoring second letter	4.65001E+14
SNOMED	Multi-infarct dementia	4.1161E+13
SNOMED	Primary degenerative dementia of the Alzheimer type, senile onset, with depression (disorder)	26852004
SNOMED	Vascular dementia (disorder)	2722993010
SNOMED	Focal Alzheimer disease	230269008
SNOMED	Anti-dementia drug therapy	9.2001E+13
SNOMED	Senile dementia with depressive or paranoid features NOS	6.13601E+14
SNOMED	Dementia associated with viral encephalitis (disorder)	2975213012
SNOMED	Lewy bodies	71948019
SNOMED	Primary degenerative dementia of the Alzheimer type, presenile onset (disorder)	416780008
SNOMED	Cortical Lewy body disease	80098002
SNOMED	Dementia monitoring third letter	7.16671E+14
SNOMED	Alzheimers disease (disorder)	26929004
SNOMED	Uncomplicated senile dementia (disorder)	2741257012
SNOMED	Multi infarct dementia with delusions	2951830014
SNOMED	Frontotemporal dementia	230270009
SNOMED	Global Deterioration Scale level 7 - very severe cognitive decline	407635001
SNOMED	[X]Dementia in other diseases classified elsewhere (disorder)	575955019
SNOMED	Familial Alzheimer-like prion disease	721219005
SNOMED	Senile dementia with depressive or paranoid features NOS (disorder)	1.35633E+15
SNOMED	Other alcoholic dementia	1.21477E+15
SNOMED	Multi infarct dementia with delusions	25772007
SNOMED	H/O: dementia	138736007
SNOMED	Vascular dementia of acute onset (disorder)	230285003
SNOMED	Dementia monitoring second letter	2.51851E+14
SNOMED	Dementia advance care plan agreed	9.12781E+14
SNOMED	Dementia monitoring telephone invite	2.51881E+14
SNOMED	Dementia co-occurrent and due to neurocysticercosis (disorder)	3334501014
SNOMED	Presenile dementia co-occurrent with human immunodeficiency virus infection (disorder)	3289553015
SNOMED	Cerebral degenerations NOS &/or Alzheimers disease &/or Picks disease &/or Reyes syndrome	267688001
SNOMED	Psychoactive substance-induced organic dementia (disorder)	631976012
SNOMED	[X] Dementia: [unspecif] or [presenile NOS (including presenile psychosis NOS)] or [primary degenerative NOS] or [senile NOS (including senile psychosis NOS)] or [senile depressed or paranoid type] (disorder)	192180006
SNOMED	Dementia annual review	2.50611E+14
SNOMED	Dementia monitoring	2.50481E+14
SNOMED	Dementia monitoring telephone invite (administrative concept)	2.49231E+14
SNOMED	Dementia care plan	8.69801E+14
SNOMED	Dementia monitoring third letter	2.49211E+14
SNOMED	Dementia medication review	9.38551E+14
SNOMED	[X]Dementia in other diseases classified elsewhere	4.64291E+14

SNOMED	Dementia monitoring first letter	2.49161E+14
SNOMED	Presenile dementia with bone cysts	2995442014
SNOMED	Parkinson-dementia complex of Guam	62239001
SNOMED	Antipsychotic drug therapy for dementia	8.89281E+14
SNOMED	Lewy bodies	43127003
SNOMED	[X] Unspecified dementia	4.13681E+14
SNOMED	Epilepsy, dementia and amelogenesis imperfecta	109478007
SNOMED	Vascular dementia of acute onset (disorder)	618070016
SNOMED	GRN-related frontotemporal dementia	702426001
SNOMED	Multi-infarct dementia (disorder)	794760011
SNOMED	Alcoholic dementia NOS	6.71511E+14
SNOMED	Binswangers disease	390000004
SNOMED	Dementia associated with another disease (disorder)	2545384017
SNOMED	Amyotrophic lateral sclerosis, parkinsonism, dementia complex (disorder)	838276009
SNOMED	Presenile dementia with delirium (disorder)	191452002
SNOMED	Cognitive impairment co-occurrent and due to human immunodeficiency virus infection	1.59281E+16
SNOMED	[X] Dementia: [unspecif] or [presenile NOS (including presenile psychosis NOS)] or [primary degenerative NOS] or [senile NOS (including senile psychosis NOS)] or [senile depressed or paranoid type] (disorder)	8.22131E+14
SNOMED	Dementia annual review	2.49181E+14
SNOMED	Dementia advance care plan review	2.34594E+15
SNOMED	Dementia due to herpes encephalitis (disorder)	3498961012
SNOMED	Delirium co-occurrent with dementia (disorder)	3446227014
SNOMED	[X]Dementia in other diseases classified elsewhere (disorder)	8.14721E+14
SNOMED	Senile dementia with delirium (disorder)	575162012
SNOMED	Dementia monitoring telephone invite	4.08501E+14
SNOMED	Dementia paralytica	51928006
SNOMED	Amyotrophic lateral sclerosis with dementia	230258005
SNOMED	Alzheimers disease with late onset	134423002
SNOMED	Psychological symptom due to dementia (finding)	789011007
SNOMED	Progressive aphasia in Alzheimers disease (disorder)	230280008
SNOMED	GRN-related frontotemporal dementia	2995747016
SNOMED	Dementia monitoring invitation	1.5559E+15
SNOMED	Lewy body disease	230275004
SNOMED	Arteriosclerotic dementia NOS (disorder)	1.32485E+15
SNOMED	Mixed cortical and subcortical vascular dementia (disorder)	2722963016
SNOMED	Predominantly cortical dementia (disorder)	2.72989E+15
SNOMED	Epilepsy co-occurrent and due to dementia (disorder)	724992007
SNOMED	Subcortical leukoencephalopathy (disorder)	833131018
SNOMED	Mixed cortical and subcortical vascular dementia (disorder)	618072012
SNOMED	Dementia due to chromosomal anomaly (disorder)	722980006
SNOMED	Vascular dementia with behavioral disturbance (disorder)	3290107010
SNOMED	Primary degenerative dementia of the Alzheimer type, senile onset, with delusions (disorder)	55009008
SNOMED	Post-traumatic dementia (disorder)	618067015
SNOMED	Dementia due to and following injury of head (disorder)	3636473018
SNOMED	Severe cognitive impairment	2.2883E+15
SNOMED	Dementia of the Alzheimer type with behavioral disturbance (disorder)	1.581E+12
SNOMED	Arteriosclerotic dementia (including [multi infarct dementia]) (disorder)	191462009
SNOMED	[X]Other Alzheimers disease	194481000
SNOMED	Assessment of psychotic symptoms of dementia	2.28439E+15
SNOMED	Assessment of psychotic symptoms of dementia	2.28438E+15
SNOMED	[X]Alcoholic dementia NOS	295795013
SNOMED	Alcoholic dementia: [other] or [NOS] (disorder)	2717315015

SNOMED	Disinhibited behavior due to dementia (disorder)	789170003
SNOMED	GDS level 5 - moderately severe cognitive decline	9.6051E+13
SNOMED	Presenile dementia NOS (disorder)	1.3563E+15
SNOMED	X-linked ataxia dementia syndrome	3318023015
SNOMED	Multi-infarct dementia	1.16851E+14
SNOMED	Acquired immune deficiency syndrome dementia complex (disorder)	192178000
SNOMED	[X]Dementia in Creutzfeldt-Jakob disease (disorder)	575958017
SNOMED	Vascular dementia of acute onset (disorder)	192166004
SNOMED	Non-familial Alzheimers disease of late onset (disorder)	230268000
SNOMED	Primary degenerative dementia of the Alzheimer type, presenile onset, with delusions (disorder)	54502004
SNOMED	Dementia co-occurrent and due to progressive multifocal leukoencephalopathy (disorder)	733193001
SNOMED	Dementia monitoring administration	4.13871E+14
SNOMED	Dementia due to Huntington chorea (disorder)	442344002
SNOMED	Dementia arising in the senium AND/OR presenium (disorder)	831001018
SNOMED	Apathetic behavior due to dementia (finding)	788867008
SNOMED	Vascular dementia (disorder)	2763302010
SNOMED	Alcoholic dementia NOS (disorder)	1.47297E+15
SNOMED	Arteriosclerotic dementia with depression (disorder)	575168011
SNOMED	Dementia co-occurrent with human immunodeficiency virus infection (disorder)	3297847015
SNOMED	Wandering due to dementia (finding)	3786566011
SNOMED	[X]Dementia in other diseases classified elsewhere (disorder)	192173009
SNOMED	Parkinsonism co-occurrent with dementia of Guadeloupe (disorder)	3303569017
SNOMED	Post-traumatic dementia (disorder)	230282000
SNOMED	Primary degenerative dementia of the Alzheimer type, presenile onset, uncomplicated (disorder)	6475002
SNOMED	Assessment of psychotic symptoms of dementia	8.86441E+14
SNOMED	On anti-dementia drug therapy	1.06704E+15
SNOMED	Alzheimers disease	73768007
SNOMED	History of dementia (situation)	161465002
SNOMED	Epileptic dementia (disorder)	230290000
SNOMED	Dialysis dementia (disorder)	9345005
SNOMED	Familial dementia Danish type (disorder)	3758316015
SNOMED	Dementia in Alzheimers disease with early onset (disorder)	192161009
SNOMED	Dementia monitoring telephone invite (administrative concept)	4.08491E+14
SNOMED	Presenile dementia (disorder)	2710469019
SNOMED	Familial dementia Danish type (disorder)	783258000
SNOMED	Alzheimers disease with early onset	134422007
SNOMED	Uncomplicated presenile dementia (disorder)	575149013
SNOMED	Uncomplicated presenile dementia (disorder)	191451009
SNOMED	Arteriosclerotic dementia NOS (disorder)	191467003
SNOMED	Arteriosclerotic dementia NOS	5.97951E+14
SNOMED	Arteriosclerotic dementia with paranoia (disorder)	191465006
SNOMED	Dementia due to Parkinsons disease (disorder)	1.01421E+14
SNOMED	Dementia due to chronic subdural hematoma (disorder)	733191004
SNOMED	Presenile dementia (disorder)	191450005
SNOMED	Familial dementia British type (disorder)	783161005
SNOMED	Depressed mood in Alzheimers disease (disorder)	1.42001E+14
SNOMED	Senile dementia with depression (disorder)	191459006
SNOMED	On anti-dementia drug therapy	2.68011E+15
SNOMED	Familial dementia British type (disorder)	3757895010
SNOMED	Dementia management: bathing (regime/therapy)	406165004
SNOMED	Presenile dementia with depression (disorder)	191455000
SNOMED	Hallucinations co-occurrent and due to late onset dementia (disorder)	3449649013

SNOMED	Dementia advance care plan	2.44548E+15
SNOMED	AIDS with dementia (disorder)	731497018
SNOMED	Rapidly progressive dementia (disorder)	723390000
SNOMED	Dementia medication review	9.38561E+14
SNOMED	GDS level 5 - moderately severe cognitive decline	1.59541E+14
SNOMED	Agitation due to dementia (finding)	3786009011
SNOMED	Punch drunk syndrome (disorder)	230283005
SNOMED	GDS level 7 - very severe cognitive decline	1.76961E+14
SNOMED	Dementia monitoring verbal invite	2.51871E+14
SNOMED	Presenile dementia (disorder)	2741255016
SNOMED	Presenile dementia with paranoia (disorder)	575154016
SNOMED	Dementia caused by volatile inhalant (disorder)	788898005
SNOMED	Parkinsonism co-occurrent with dementia of Guadeloupe (disorder)	715737004
SNOMED	Psychoactive substance-induced organic dementia (disorder)	111480006
SNOMED	Vascular dementia with behavioral disturbance (disorder)	2.88631E+14
SNOMED	Other senile/presenile dementia (disorder)	154851005
SNOMED	Dementia (disorder)	790458016
SNOMED	Review of dementia advance care plan	9.12801E+14
SNOMED	Dementia co-occurrent and due to Picks disease (disorder)	2.1921E+13
SNOMED	Senile dementia (disorder)	15662003
SNOMED	[X]Dementia in Parkinsons disease	192177005
SNOMED	Wernicke-Korsakov syndrome	69482004
SNOMED	Review of dementia advance care plan	2.74299E+15
SNOMED	Vascular dementia (disorder)	154850006
SNOMED	Altered behavior in Alzheimers disease (disorder)	9.7751E+13
SNOMED	Senile dementia with depressive or paranoid features (disorder)	154848003
SNOMED	[X]Dementia in Picks disease	4.55381E+14
SNOMED	Primary degenerative dementia of the Alzheimer type, presenile onset, with delirium (disorder)	65096006
SNOMED	Dementia monitoring third letter	4.13921E+14
SNOMED	Senile dementia with depressive or paranoid features (disorder)	2740355014
SNOMED	Primary degenerative dementia of the Alzheimer type, senile onset in remission (disorder)	698954005
SNOMED	Review of dementia advance care plan	1.09511E+15
SNOMED	Senile dementia with delusion (disorder)	1196320010
SNOMED	Dementia due to herpes encephalitis (disorder)	733192006
SNOMED	Aggression due to dementia (finding)	3786007013
SNOMED	Dementia associated with another disease (disorder)	191519005
SNOMED	Senile dementia (disorder)	742529013
SNOMED	[X]Other Alzheimers disease	4.43781E+14
SNOMED	Arteriosclerotic dementia with paranoia (disorder)	575167018
SNOMED	Vascular dementia without behavioral disturbance (disorder)	1.62764E+16
SNOMED	Altered behavior in dementia due to Huntington chorea (disorder)	2987466016
SNOMED	Alzheimers disease co-occurrent with delirium (disorder)	1.42011E+14
SNOMED	Senile or presenile psychoses NOS	6.23941E+14
SNOMED	Dementia care plan	736371006
SNOMED	Dementia caused by toxin (disorder)	3334071016
SNOMED	Dementia due to metabolic abnormality (disorder)	722979008
SNOMED	Vascular dementia (disorder)	429998004
SNOMED	Lewy body dementia with behavioural disturbance	1.35811E+14
SNOMED	Global Deterioration Scale level 7 - very severe cognitive decline	2914082012
SNOMED	[X]Dementia in other specified diseases classif elsewhere (disorder)	575962011
SNOMED	Dementia (& [presenile] or [senile]) (disorder)	154845000
SNOMED	[X]Delirium superimposed on dementia	4.02771E+14

SNOMED	Presenile dementia with paranoia (disorder)	191454001
SNOMED	Dementia care plan	8.69791E+14
SNOMED	Presenile dementia associated with acquired immunodeficiency syndrome (disorder)	421023003
SNOMED	Dementia advance care plan	9.59361E+14
SNOMED	Primary degenerative dementia of the Alzheimer type, senile onset, uncomplicated (disorder)	66108005
SNOMED	Arteriosclerotic dementia NOS	1.32484E+15
SNOMED	Arteriosclerotic dementia (including [multi infarct dementia]) (disorder)	2713931017
SNOMED	Dementia due to disorder of central nervous system (disorder)	3445726012
SNOMED	Dementia medication review	2.40311E+15
SNOMED	Cerebral degenerations NOS &/or Alzheimers disease &/or Picks disease &/or Reyes syndrome	154998003
SNOMED	Multi-infarct dementia with depression	14070001
SNOMED	Dementia monitoring administration	4.06841E+14
SNOMED	Primary degenerative dementia (disorder)	2.2381E+13
SNOMED	Non-familial Alzheimers disease of early onset	230266001
SNOMED	Dementia care plan reviewed	9.56871E+14
SNOMED	Senile or presenile psychoses NOS	294660013
SNOMED	Dementia monitoring administration	2.51831E+14
SNOMED	Dementia associated with cerebral anoxia (disorder)	698781002
SNOMED	Senile dementia with delirium (disorder)	2740645019
SNOMED	Frontotemporal dementia with gene located on 3p11	2995083019
SNOMED	Dementia due to disorder of central nervous system (disorder)	724776007
SNOMED	Dementia associated with acquired immunodeficiency syndrome (disorder)	2971994019
SNOMED	On anti-dementia drug therapy	735141001
SNOMED	Dementia following injury caused by exposure to ionizing radiation (disorder)	733185001
SNOMED	Predominantly cortical vascular dementia (disorder)	1.08953E+15
SNOMED	Cerebral degeneration presenting primarily with dementia	279982005
SNOMED	Familial Alzheimer-like prion disease	3324650018
SNOMED	Language disorder of dementia	229672009
SNOMED	Multi infarct dementia with atherosclerosis	1.06021E+14
SNOMED	Multi infarct dementia with atherosclerosis	3306368015
SNOMED	On anti-dementia drug therapy	3513263016
SNOMED	Multi infarct dementia with delirium	10349009
SNOMED	[X]Alcoholic dementia NOS	4.32111E+14
SNOMED	GDS level 6 - severe cognitive decline	1.35491E+14
SNOMED	Other senile/presenile dementia (disorder)	2758678015
SNOMED	Dementia monitoring first letter	4.64991E+14
SNOMED	Dementia associated with neurosyphilis (disorder)	2975441013
SNOMED	Nuchal dystonia-dementia syndrome	28978003
Diabetes Mellitus		
Read	Maturity onset diabetes of the young type 5	C10Q.
Read	Discharged from diabetes shared care programme	XaQGH
Read	Diabetes mellitus due to insulin receptor antibodies	XSETp
Read	Type II diabetes mellitus with persistent proteinuria	XaIzQ
Read	Diabetes structured education programme completed	9OLF.
Read	Diabetes mellitus with unspecified complication	C10z.
Read	Referral to community diabetes service	8Hlc.
Read	Diabetes mellitus with neurological manifestation	XE10H
Read	Type I diabetes mellitus with arthropathy	XaFmL
Read	Lifestyle education for diabetes	XabZm
Read	DM induced by non-steroid drugs without complication	XaJlM
Read	Referral to community diabetes service	XaYaB
Read	H/O: diabetes mellitus type 1	XaXZv

Read	Advised ([?AND/OR advice ?AND/OR eductn]) diabetes driving	XaX8e
Read	Diabetes mellitus, adult, + peripheral circulatory disorder	C1071
Read	Referral to community diabetes specialist nurse	XaMhQ
Read	Declined consent for diabetes year of care programme	XaQjt
Read	Seen by diabetes specialist nurse	9NlP1
Read	Type 2 diabetes mellitus with gastroparesis	C10FR
Read	Did not attend DAFNE diabetes structured education programme	XaNU1
Read	Insulin dependent diabetes maturity onset	C1089
Read	Referral to DAFNE diabetes structured education programme	XaNTQ
Read	Did not complet XPERT diabetes structured education programm	XaNTg
Read	Diabetes mellitus with neurological manifestation	C106.
Read	DAFNE diabetes structured education programme completed	XaNTB
Read	Attended XPERT diabetes structured education programme	XaNT8
Read	Diabetes mellitus, adult onset, no mention of complication	XE10F
Read	Non-insulin depend diabetes mellitus with diabetic cataract	C109E
Read	Diabetes type 1 review	XaMhJ
Read	Type 1 diabetes mellitus with gastroparesis	XaKyW
Read	Diabetes clinical management plan	XaJYg
Read	Type II diabetes mellitus with exudative maculopathy	XaJQp
Read	Diabetes monitoring check done	XE2Ne
Read	Type 2 diabetes mellitus - poor control	C10F7
Read	Fibrocalculous pancreatopathy without complication	XaJlP
Read	Hyperosmolar non-ketotic state in type 2 diabetes mellitus	C109K
Read	IDDM with peripheral circulatory disorder	C1073
Read	Diabetes mellitus, adult onset, + neurological manifestation	C1061
Read	Type II diabetes mellitus with persistent microalbuminuria	XaIzR
Read	Referral to community diabetes clinic	8HTE1
Read	Type 1 diabetes mellitus with persistent proteinuria	XaIzM
Read	Soft tissue complication of diabetes mellitus	X50GO
Read	Diabetes medication review	XaIfM
Read	Declined consent for diabetes year of care programme	66AQ1
Read	Insulin dependent diab mell with neuropathic arthropathy	C108J
Read	Diabetes monitoring third letter	9OL6.
Read	Type II diabetes mellitus with hypoglycaemic coma	XaFWI
Read	Referral to diabetes nurse	XaE46
Read	Diabetes resolved	XaFsp
Read	Insulin dependent diab mell with peripheral angiopathy	C108G
Read	Referral to XPERT diabetes structured education programme	XaNTT
Read	Eating disorder co-occurrent with diabetes mellitus type 1	XafjT
Read	Diabetes mellitus, adult onset, with renal manifestation	C1041
Read	Asymptomatic diabetic neuropathy	F3722
Read	Type 1 diabetes mellitus with gangrene	C10E6
Read	Type 2 diabetes mellitus risk assessment inv third letter	Xaffj
Read	Type I diabetes mellitus in remission	Xaage
Read	X-PERT First Steps diabetes self-management programme complt	XaeWH
Read	Type II diabetes mellitus with mononeuropathy	XaEnp
Read	Type I diabetes mellitus with mononeuropathy	XaEnn
Read	Type II diabetes mellitus without complication	XaELQ
Read	Attending diabetes clinic	XaE4q
Read	Patient on maximal tolerated therapy for diabetes	8BL2.
Read	Type I diabetes mellitus without complication	XaELP
Read	Malnutrition-related diabetes mellitus with renal complicatn	C10A2

Read	Type 1 diabetes mellitus without complication	C10EA
Read	Type I diabetes mellitus with nephropathy	XaF04
Read	Provision written information abt diabetes & high HbA1c levl	Xac27
Read	Type I diabetes mellitus with diabetic cataract	XaFm8
Read	Dietary advice for type I diabetes	Xa2h9
Read	Provision of written information about diabetes and driving	XaZpJ
Read	Diabetes Distress Scale 2 item	XabAQ
Read	Conversion to insulin by diabetes specialist nurse	XaaJK
Read	Type II diabetes mellitus in remission	Xaagf
Read	Unspecified diabetes mellitus with multiple complications	C108z
Read	Erectile dysfunction due to diabetes mellitus	Xaa8r
Read	Insulin autoimmune syndrome without complication	XaJlN
Read	Secondary diabetes mellitus	C10N.
Read	Lipoatrophic diabetes	XSETe
Read	Secondary diabetes mellitus	X40JA
Read	Hyperproinsulinemia	X40JS
Read	Hypoglycaemic event in diabetes	X40Jq
Read	Did not attend XPERT diabetes structured education programme	9NiE.
Read	Congenital lipoatrophic diabetes	X40JO
Read	Did not complete diabetes structured education programme	XaNTd
Read	Diabetes mellitus NOS with neurological manifestation	C106z
Read	Diabetes mellitus (& [ketoacidosis])	XE128
Read	Malnutrition-related diabetes mellitus - protein-deficient	X40J9
Read	Non-insulin dependent diabetes mellitus with mononeuropathy	C109A
Read	H/O: diabetes mellitus	1434
Read	Renal diabetes	X30Hd
Read	Diabetic mononeuropathy	X00Al
Read	Pre-existing diabetes mellitus, unspecified	L180X
Read	Pre-existing malnutrition-related diabetes mellitus	L1807
Read	Diabetes monitoring short msg service text msg 3rd invitaton	Xafj2
Read	Insulin treated Type 2 diabetes mellitus	C10FJ
Read	Diabetes mellitus with other specified manifestation	C10y.
Read	High risk non proliferative diabetic retinopathy	F4208
Read	Advanced diabetic retinal disease	F4205
Read	Secondary diabetes mellitus without complication	C10N0
Read	Diabetes mellitus with hyperosmolar coma	C102.
Read	Diabetes mellitus NOS with no mention of complication	C100z
Read	Joint consultation GP & community diabetes specialist nurse	9Na52
Read	Refuses diabetes monitoring	9OL2.
Read	Fibrocalculous pancreatopathy	C10L.
Read	Type 1 diabetes mellitus with peripheral angiopathy	C10EG
Read	Other specified diabetes mellitus with unspecified comps	C10zy
Read	Education about lifestyle for risk of diabetes	Xaafx
Read	Diabetes mellitus, adult, + other specified manifestation	C10y1
Read	H/O: Admission in last year for diabetes foot problem	14F4.
Read	Type 2 diabetes mellitus with persistent microalbuminuria	C10FM
Read	Type 2 diabetes mellitus with gangrene	C10F5
Read	Malnutrit-related diabetes mellitus wth ophthalmic complicat	C10A3
Read	Non-insulin dependent diabetes mellitus	C109.
Read	Maternally inherited diabetes mellitus	C10FS
Read	[X]Diabetes mellitus	Cyu2.
Read	Insulin autoimmune syndrome without complication	C10J0

Read	Insulin autoimmune syndrome	C10J.
Read	Secondary pancreatic diabetes mellitus without complication	C10G0
Read	Type 2 diabetes mellitus with persistent proteinuria	C10FL
Read	Type 2 diabetes mellitus with neuropathic arthropathy	C10FH
Read	Diabetes mellitus, juvenile, + neurological manifestation	C1060
Read	Type 2 diabetes mellitus with polyneuropathy	C10FB
Read	Diabetes mellitus, adult onset, + unspecified complication	C10z1
Read	Type 2 diabetes mellitus without complication	C10F9
Read	High risk non proliferative diabetic retinopathy	XaIW8
Read	Latent autoimmune diabetes mellitus in adult	C10ER
Read	Type 1 diabetes mellitus with ketoacidotic coma	C10EN
Read	Malnutrition-related diabetes mellitus	C10A.
Read	Diabetes clinic satisfaction questionnaire	XM0cX
Read	Latent autoimmune diabetes mellitus in adult	XaOPu
Read	Referral to diabetes structured education programme	XaKGy
Read	Type 1 diabetes mellitus - poor control	C10E8
Read	Insulin dependent diabetes mellitus with polyneuropathy	C108C
Read	Understands diet - diabetes	66A9.
Read	Referral to diabetes preconception counselling clinic	8HTe.
Read	Diabetes mellitus autosomal dominant type 2	C10D.
Read	Diabetes mellitus autosomal dominant	C10C.
Read	Steroid induced diabetes mellitus without complication	C10B0
Read	Diabetes mellitus, juvenile type, with ketoacidotic coma	C1030
Read	Cystic fibrosis related diabetes mellitus	C10N1
Read	Malnutrition-related diabetes mellitus with coma	C10A0
Read	Erectile dysfunction due to diabetes mellitus	K27y7
Read	Diabetic cataract	F4640
Read	Referral to diabetes nurse	8H7f.
Read	Type 1 diabetes mellitus with ketoacidosis	C10EM
Read	Non-insulin dependent diabetes mellitus with arthropathy	C109G
Read	Non-insulin-dependent d m with peripheral angiopath	C109F
Read	Pre-existing type 1 diabetes mellitus in pregnancy	L180A
Read	Non-insulin dependent diabetes mellitus with nephropathy	C109C
Read	Non-insulin dependent diabetes mellitus with polyneuropathy	C109B
Read	Non-insulin-dependent diabetes mellitus without complication	C1099
Read	Referral to DESMOND diabetes structured education programme	8Hj4.
Read	Insulin dependent diabetes mellitus with arthropathy	C108H
Read	Insulin dependent diabetes mellitus with diabetic cataract	C108F
Read	Type 2 diabetes mellitus with ketoacidosis	C10FN
Read	Unstable insulin dependent diabetes mellitus	C1084
Read	Insulin-dependent diabetes mellitus with neurological comps	C1082
Read	Provision of diabetes identity card	8OAL.
Read	Background diabetic retinopathy	F4200
Read	Diabetes mellitus, juvenile +peripheral circulatory disorder	C1070
Read	Diabetes: [peripheral circulatory disease] or [gangrene]	XE12K
Read	Proliferative diabetic retinopathy	F4201
Read	Health education - diabetes	XaJ7D
Read	Diabetes mellitus, adult onset, + ophthalmic manifestation	C1051
Read	Diabetes mellitus with ophthalmic manifestation	C105.
Read	Diabetes mellitus, juvenile type, with renal manifestation	C1040
Read	Diabetes mellitus NOS with ketoacidotic coma	C103z
Read	Type 2 diabetes mellitus risk assessment verbal invitation	Xaffk

Read	Malnutrition-related diabetes mellitus wth neuro complicatns	C10A4
Read	Lifestyle education for diabetes	67HA.
Read	Diabetes monitoring second letter	9OL5.
Read	Myasthenic syndrome due to diabetic amyotrophy	F3813
Read	Referral to children's diabetes nurse specialist	XaP4P
Read	Diabetes Year of Care annual review	66AS0
Read	DAFNE diabetes structured education programme completed	9OLJ.
Read	Diabetes mellitus, adult with gangrene	C1072
Read	Type 1 diabetes mellitus with ophthalmic complications	C10E1
Read	Diabetes monitored (& check done)	9OLA.
Read	Referral to type I diabetes structured education programme	XaX49
Read	Diabetes monitoring first letter	9OL4.
Read	Diabetes monitoring default	9OL3.
Read	Under care of hospital-based diabetes specialist nurse	9NN90
Read	Diabetes dietitian	XaBqu
Read	Steroid induced diabetes	C11y0
Read	Referral to DAFNE diabetes structured educn prog declined	XaZuQ
Read	Joint consultn pracse nurse & comm diabetes specialist nurse	XacIu
Read	Type 2 diabetes mellitus with ulcer	C10F4
Read	Lipoatrophic diabetes mellitus without complication	C10M0
Read	Type A insulin resistance without complication	XaJlO
Read	Diabetic hyperosmolar non-ketotic state	XaCJ2
Read	XPERT diabetes structured education programme completed	9OLL.
Read	Pan retinal photocoagulation for diabetes	XaJlI
Read	Type 2 diabetes mellitus risk assessment inv first letter	Xaffh
Read	Type A insulin resistance	C10K.
Read	Insulin dependent diabetes mellitus with ulcer	C1085
Read	Diabetes mellitus with persistent proteinuria	XaIz0
Read	Insulin dependent diabetes mellitus	C108.
Read	Provsn written information about diabetes & high cholesterol	8OAK.
Read	Provision of written information about diabetes and driving	8OA3.
Read	Other specified diabetes mellitus with multiple comps	C108y
Read	Did not complete DESMOND diabetes structured educat program	8I83.
Read	Attended DAFNE diabetes structured education programme	9OLH.
Read	Type 1 diabetes mellitus with renal complications	C10E0
Read	Referral to community diabetes specialist nurse	8Hl4.
Read	Discharged from diabetes shared care programme	8HgC.
Read	Referral to diabetes structured education programme	8Hj0.
Read	Type 1 diabetes mellitus with neuropathic arthropathy	C10EJ
Read	Dietary advice for diabetes mellitus	Xa2h7
Read	Joint consultation GP & community diabetes specialist nurse	XacJ1
Read	Diabetes care plan agreed	8CS0.
Read	Diabetes clinical management plan	8CR2.
Read	Hyperosmolar non-ketotic state in type 2 diabetes mellitus	C10FK
Read	Diabetes + eye manifestation (& [cataract] or [retinopathy])	XE12G
Read	Diabetic severe hyperglycaemia	X40Jb
Read	Diabetes treatment satisfaction questionnaire	3883
Read	Referral to diabetes preconception counselling clinic	XaKGq
Read	Type 1 diabetes mellitus with persistent microalbuminuria	C10EL
Read	Did not attend DESMOND diabetes structured education program	9NiD.
Read	Diabetes mellitus, juvenile type, + ophthalmic manifestation	C1050
Read	Type 1 diabetes mellitus with exudative maculopathy	C10EP

Read	Diabetes mellitus with gangrene	XM1Qx
Read	Advanced diabetic retinal disease	XaD2T
Read	Acute painful diabetic neuropathy	F3720
Read	Insulin dependent diabetes mellitus with hypoglycaemic coma	C108E
Read	Diabetes monitoring admin.NOS	9OLZ.
Read	Pre-existing diabetes mellitus, non-insulin-dependent	L1806
Read	Patient on maximal tolerated therapy for diabetes	XaJ5j
Read	Type 1 diabetes mellitus	C10E.
Read	Diabetes care plan declined	8IE2.
Read	Diabetes structured education programme declined	9OLM.
Read	Type II diabetes mellitus with gastroparesis	XaKyX
Read	Diabetes mellitus with peripheral circulatory disorder	C107.
Read	X-PERT First Steps diabetes self-management programme complt	9OLP.
Read	Other specified diabetes mellitus with other spec comps	C10yy
Read	Diabetes treatment satisfaction questionnaire	XM0cY
Read	Secondary endocrine diabetes mellitus	X40JC
Read	Diabetic on oral treatment	66A4.
Read	Diabetes mellitus NOS with unspecified complication	C10zz
Read	Diabetes mellitus	C10..
Read	Unstable diabetes	XM1Xk
Read	Type 2 diabetes mellitus with mononeuropathy	C10FA
Read	Discharged from care of diabetes specialist nurse	8Hg4.
Read	Insulin resistance in diabetes	X40KG
Read	Diabetes care by hospital only	XaIIe
Read	Pan retinal photocoagulation for diabetes	7276
Read	Diabetes type II review	XaMhK
Read	Insulin-dependent diabetes mellitus with renal complications	C1080
Read	Other specified diabetes mellitus with periph circ comps	C107y
Read	Type 2 diabetes mellitus	C10F.
Read	Referral to diabetes special interest general practitioner	8H4e.
Read	Non proliferative diabetic retinopathy	F4206
Read	Type 1 diabetes mellitus with persistent proteinuria	C10EK
Read	Type I diabetes mellitus	X40J4
Read	Diabetes mellitus NOS with other specified manifestation	C10yz
Read	Type 2 diabetes mellitus with ketoacidotic coma	C10FP
Read	Hyperosmolar non-ketotic state in type II diabetes mellitus	XaIrf
Read	Diabetes mellitus in remission	C10P.
Read	Insulin dependent diabetes mellitus with gangrene	C1086
Read	Diabetes monitoring short msg service text msg 2nd invitaton	Xafj1
Read	Type 1 diabetes mellitus maturity onset	C10E9
Read	Type 1 diabetes mellitus with polyneuropathy	C10EC
Read	Did not attend XPERT diabetes structured education programme	XaNU3
Read	Diabetes management plan given	66AR.
Read	Referral to XPERT diabetes structured education programme	8Hj5.
Read	Diabetes + nephropathy (& [Kimmelstiel-Wilson syndrome])	XE12E
Read	Lipoatrophic diabetes mellitus without complication	XaJlQ
Read	Diabetes mellitus, adult onset, with hyperosmolar coma	C1021
Read	Acrorenal field defect,ectodys,lipoat diabetes	X40JP
Read	Insulin dependent diabetes mellitus - poor control	C1088
Read	Diabetic maculopathy	X00dG
Read	Drug-induced diabetes mellitus	XSETK
Read	Diabetes care by hospital only	66AU.

Read	Type 2 diabetes mellitus with neurological complications	C10F2
Read	Attending diabetes clinic	9NM0.
Read	Attended diabetes structured education programme	XaKH0
Read	Brittle diabetes	66AJ1
Read	Maturity onset diabetes of the young type 5	XacoB
Read	Maturity onset diabetes mellitus in young	XSETH
Read	Diabetes wellbeing questionnaire	XM0cZ
Read	Seen in community diabetes specialist clinic	XaKaM
Read	Did not attend diabetes structured education programme	XaNTa
Read	Attended XPERT diabetes structured education programme	9OLG.
Read	Lipoatrophic diabetes mellitus	C10M.
Read	Diabetes structured education programme not available	8I94.
Read	Diabetes monitoring deleted	9OL9.
Read	Diabetes mellitus with persistent microalbuminuria	XaIyz
Read	Preproliferative diabetic retinopathy	F4202
Read	Diabetes care plan declined	XaXv9
Read	Type 2 diabetes mellitus with renal complications	C10F0
Read	Polyglandular autoimmune syndrome - type II	X40JK
Read	Type II diabetes on insulin	XaIfG
Read	Provision of diabetes clinical summary	67D8.
Read	Patient offered diabetes structured education programme	679R.
Read	Health education - diabetes	679L.
Read	Has diabetes identity card	66o3.
Read	Seen by diabetes specialist nurse	XaeBx
Read	Type 1 diabetes mellitus with arthropathy	C10EH
Read	Retinal abnormality - diabetes-related	XaBul
Read	Diabetes mellitus, juvenile type, + unspecified complication	C10z0
Read	Has seen dietitian - diabetes	66A8.
Read	Diabetes self-management plan agreed	661M4
Read	[EDTA] Diabetes Type II associated with renal failure	XM19j
Read	Provsn written information about diabetes & high cholesterol	XacSK
Read	Maternally inherited diabetes mellitus	XaOPt
Read	Diabetes self-management plan review	XaYZP
Read	Referral to community diabetes specialist nurse declined	XaZNW
Read	Joint consultn pracse nurse & comm diabetes specialist nurse	9Na51
Read	Type 2 diabetes mellitus with diabetic cataract	C10FE
Read	Type 2 diabetes mellitus with nephropathy	C10FC
Read	Type 2 diabetes mellitus with exudative maculopathy	C10FQ
Read	Myasthenic syndrome due to diabetic amyotrophy	XE15n
Read	Type I diabetes mellitus in remission	C10P0
Read	Type 2 diabetes mellitus risk assessment inv second letter	Xaffi
Read	Diabetes mellitus NOS with peripheral circulatory disorder	C107z
Read	Diabetes mellitus with renal manifestation	C104.
Read	Type 1 diabetes mellitus with retinopathy	C10E7
Read	Did not attend diabetes structured education programme	9NiA.
Read	Malnutrit-relat diabetes mellitus with other spec comps	C10AX
Read	DM induced by non-steroid drugs without complication	C10H0
Read	Type 2 diabetes mellitus risk assessment declined	Xafak
Read	Seen by general practitioner special interest in diabetes	XaNHp
Read	NIDDM with peripheral circulatory disorder	C1074
Read	Education about diabetes and driving	679L2
Read	Other specified diabetes mellitus with ketoacidosis	C101y

Read	Diabetes structured education programme not available	XaadP
Read	Insulin treated Type 2 diabetes mellitus	C109J
Read	Attends diabetes monitoring	9OL1.
Read	Type II diabetes mellitus with diabetic cataract	XaFmA
Read	Diabetes mellitus, juvenile type, no mention of complication	XE10E
Read	Referral to DAFNE diabetes structured education programme	8Hj3.
Read	Patient offered diabetes structured education programme	XaKSp
Read	Type 2 diabetes mellitus with ophthalmic complications	C10F1
Read	Diabetes mellitus NOS with ketoacidosis	C101z
Read	Diabetes mellitus NOS with ophthalmic manifestation	C105z
Read	[X]Unspecified diabetes mellitus with renal complications	Cyu23
Read	Discharged from care of diabetes specialist nurse	XaKbu
Read	Diabetes mellitus: [adult onset] or [noninsulin dependent]	XE12A
Read	Transition of diabetes care options discussed	8CP2.
Read	Diabetes clinical pathway	XaZig
Read	Foot abnormality - diabetes related	2G5C.
Read	Retinal abnormality - non-diabetes	XaBum
Read	Type II diabetes mellitus with neuropathic arthropathy	XaFn9
Read	Under care of community-based diabetes specialist nurse	9NN91
Read	Diabetes monitoring call	XaBU8
Read	Did not complete DAFNE diabetes structured education program	XaNTe
Read	Type I diabetes mellitus with neuropathic arthropathy	XaFmM
Read	Non-insulin dependent diabetes mellitus with ulcer	C1094
Read	Seen in community diabetes specialist clinic	9N0n.
Read	Diabetes self-management plan agreed	XaYbB
Read	Recommendation self-refer for diabetes structured education	Xacu2
Read	[X]Glomerular disorders in diabetes mellitus	Kyu03
Read	Type 1 diabetes mellitus with nephropathy	C10ED
Read	Type 2 diabetes mellitus with multiple complications	C10F3
Read	Type II diabetes mellitus with polyneuropathy	XaEnq
Read	Diabetes mellitus with renal manifestation	XE10G
Read	Insulin-dependent diabetes without complication	C108A
Read	Diabetes Distress Scale 2 item	38QW.
Read	Under care of diabetes specialist nurse	9NN9.
Read	Diabetes monitoring invitation email	XagLc
Read	Diabetes mellitus with ketoacidosis	C101.
Read	Advanced diabetic maculopathy	F4203
Read	Insulin dependent diabetes mellitus with mononeuropathy	C108B
Read	[X]Pre-existing diabetes mellitus, unspecified	Lyu29
Read	Secondary pancreatic diabetes mellitus	X40JB
Read	Secondary pancreatic diabetes mellitus without complication	XaJlL
Read	Diabetes monitor invitation by SMS (short message service)	9OLN.
Read	Patient diabetes education review	66Af.
Read	Provision of written information about diabetes and high BP	8OAH.
Read	Diabetes mellitus, juvenile type, with ketoacidosis	C1010
Read	Type I diabetes mellitus with hypoglycaemic coma	XaFWG
Read	Genetic syndromes of diabetes mellitus	X40JG
Read	Diabetes monitoring telephone invite	9OL8.
Read	[X]Malnutrit-related diabetes mellitus with unspec complics	Cyu22
Read	Diabetic retinopathy NOS	F420z
Read	Education in self management of diabetes	679L0
Read	Fibrocalculous pancreatopathy without complication	C10L0

Read	Insulin-dependent diabetes mellitus secretory diarrhoea synd	X40JY
Read	Insulin dependent diabetes mellitus with nephropathy	C108D
Read	Autonomic neuropathy due to diabetes	F1711
Read	Other specified diabetes mellitus with coma	C103y
Read	Referral to DAFNE diabetes structured educn prog declined	8IEa.
Read	Malnutrition-related diabetes mellitus without complications	C10A7
Read	Insulin dependent diabetes mellitus with retinopathy	C1087
Read	Diabetic retinopathy	F420.
Read	Diabetes mellitus, juvenile, + other specified manifestation	C10y0
Read	Unstable type 1 diabetes mellitus	C10E4
Read	[EDTA]Diabetes Type I associated with renal failure	XM19i
Read	Fibrocalculous pancreatic diabetes	XSETI
Read	Referral to diabetes special interest general practitioner	XaON1
Read	Did not attend DAFNE diabetes structured education programme	9NiC.
Read	Foot abnormality - diabetes related	2G510
Read	Type I diabetes mellitus with polyneuropathy	XaEno
Read	Diabetic maculopathy	F4204
Read	Diabetes-deafness syndrome maternally transmitted	X40JZ
Read	Diabetes + neuropathy (& [amyotrophy])	XE12I
Read	Diabetes mellitus induced by non-steroid drugs	XaJUI
Read	Diabetes mellitus NOS with hyperosmolar coma	C102z
Read	Secondary pancreatic diabetes mellitus	C10G.
Read	Provision of diabetes clinical summary	XaZCO
Read	Type 2 diabetes mellitus risk assessment invitation	Xaffg
Read	Type II diabetes mellitus with peripheral angiopathy	XaFn7
Read	Provision of diabetes identity card	XacXr
Read	Provision written information abt diabetes & high HbA1c levl	8OAJ.
Read	Type 2 diabetes mellitus risk assessment inv SMS text msg	Xaffm
Read	Diabetes Year of Care annual review	Xaag1
Read	H/O: autosomal dominant diabetes mellitus	XaXZB
Read	Diabetes mellitus, juvenile type, with hyperosmolar coma	C1020
Read	Diabetes quality of life questionnaire	XaZMQ
Read	Diabetes: practice programme	66AP.
Read	Type II diabetes mellitus with arthropathy	XaFn8
Read	Attended diabetes structured education programme	9OLB.
Read	Retinal abnormality - diabetes related	2BBF.
Read	Patient diabetes education review	XaKGs
Read	Diabetes mellitus: [juvenile] or [insulin dependent]	XE12C
Read	Type 1 diabetes mellitus with multiple complications	C10E3
Read	Diabetes mellitus in remission	Xaagd
Read	Perceived control of insulin-dependent diabetes	XaA6b
Read	Malnutrition-related diabetes mellitus with ketoacidosis	C10A1
Read	Diabetes mellitus, adult onset, with ketoacidosis	C1011
Read	Non-insulin-dependent diabetes mellitus with retinopathy	C1096
Read	Diabetes type 2 review	66Ao.
Read	Discharge from secondary care diabetes service	8Hgd.
Read	High risk proliferative diabetic retinopathy	F4207
Read	Skin ulcer associated with diabetes mellitus	Xagk3
Read	Type A insulin resistance	X40KH
Read	Type 1 diabetes mellitus with ulcer	C10E5
Read	Retinal abnormality - non-diabetes	2BBG.
Read	Transition of diabetes care options discussed	XaKHD

Read	Type 1 diabetes mellitus with neurological complications	C10E2
Read	Diabetes mellitus autosomal dominant	X40JI
Read	Type 1 diabetes mellitus with persistent microalbuminuria	XaIzN
Read	Discharge from secondary care diabetes service	Xabhn
Read	Malnutritn-relat diabetes melitus wth periph circul complctn	C10A5
Read	Diabetes mellitus with ketoacidotic coma	C103.
Read	Type 2 diabetes mellitus with retinopathy	C10F6
Read	Hypoglycaemic state in diabetes	X40Jr
Read	Non-insulin dependent diabetes mellitus with gangrene	C1095
Read	Unstable type I diabetes mellitus	Xa4g7
Read	Abnormal metabolic state in diabetes mellitus	X40Ja
Read	Under care of hospital-based diabetes specialist nurse	XaeBz
Read	Type A insulin resistance without complication	C10K0
Read	Diabetes mellitus with no mention of complication	C100.
Read	Malnutrit-related diabetes mellitus with unspec complics	C10AW
Read	Type 1 diabetes mellitus with hypoglycaemic coma	C10EE
Read	Education about lifestyle for risk of diabetes	67H9.
Read	Nephrotic syndrome in diabetes mellitus	K01x1
Read	Type II diabetes on diet only	XaIfI
Read	Did not complete diabetes structured education programme	8I81.
Read	Diabetes mellitus induced by steroids	C10B.
Read	Insulin autoimmune syndrome	XaJUH
Read	Diabetes medication review	8B3l.
Read	Diabetes resolved	21263
Read	Dietary advice for type II diabetes	Xa2hA
Read	Diabetes with other complications	XE12M
Read	Diabetes well being questionnaire	3882
Read	Diabetes monitoring status	XaBU9
Read	Education about diabetes and driving	XaaaR
Read	Foot abnormality - diabetes-related	XaBun
Read	Diabetes care plan agreed	XaKSn
Read	Non proliferative diabetic retinopathy	XaIP5
Read	Chronic painful diabetic neuropathy	F3721
Read	Pre-existing type 2 diabetes mellitus in pregnancy	L180B
Read	Non-insulin-dependent diabetes mellitus with ophthalm comps	C1091
Read	Conversion to insulin by diabetes specialist nurse	66AH2
Read	[X]Malnutrit-relat diabetes mellitus with other spec comps	Cyu21
Read	Other drugs used in diabetes	ft...
Read	Diabetes clinical pathway	8CMW7
Read	DESMOND diabetes structured education programme completed	9OLK.
Read	Type II diabetes mellitus	X40J5
Read	Diabetes resolved	212H.
Read	Diabetic iritis	F4407
Read	Referral to community diabetes clinic	XaZRZ
Read	Other specified diabetes mellitus with renal complications	C104y
Read	Education in self management of diabetes	XaR8L
Read	Non-insulin dependent d m with neuropathic arthropathy	C109H
Read	Diabetic mononeuritis multiplex	F3450
Read	Under care of community-based diabetes specialist nurse	XaeC0
Read	Pre-existing diabetes mellitus, insulin-dependent	L1805
Read	Under care of diabetes specialist nurse	XaJO4
Read	H/O: diabetes mellitus type 2	XaXZR

Read	Malnutrition-related diabetes mellitus - fibrocalculous	X40J8
Read	Diabetic mononeuritis NOS	F35z0
Read	Type 1 diabetes mellitus with exudative maculopathy	XaJSr
Read	Diabetes mellitus with nephropathy NOS	C104z
Read	Secondary diabetes mellitus without complication	XaJlR
Read	Type II diabetes mellitus with nephropathy	XaF05
Read	[X]Other specified diabetes mellitus	Cyu20
Read	Diabetes monitoring verbal invite	9OL7.
Read	H/O: Admission in last year for diabetes foot problem	XaBwj
Read	Has diabetes identity card	XacY8
Read	Did not complete DAFNE diabetes structured education program	8I82.
Read	Type 2 diabetes mellitus risk assessment telephone inv	Xaffl
Read	Cystic fibrosis related diabetes mellitus	XaMzI
Read	Non-insulin dependent diabetes mellitus - poor control	C1097
Read	Diabetes Distress Scale 17 item	XabAX
Read	Referral to community diabetes specialist nurse declined	8IEQ.
Read	Diabetes mellitus autosomal dominant type 2	X40JJ
Read	Diabetes mellitus, adult onset, no mention of complication	C1001
Read	Diabetes monitoring short msg service text msg 1st invitaton	Xafj0
Read	Malnutrition-related diabetes mellitus with multiple comps	C10A6
Read	Diabetes monitor invitation by SMS (short message service)	XaX1n
Read	Type 2 diabetes mellitus with arthropathy	C10FG
Read	Non-insulin dependent diabetes mellitus with hypoglyca coma	C109D
Read	Provision of written information about diabetes and high BP	Xac26
Read	Diabetes self management plan	XaQdT
Read	Non-insulin-dependent diabetes mellitus with multiple comps	C1093
Read	Diabetes monitoring invitation	XaCGh
Read	Type I diabetes mellitus with peripheral angiopathy	XaFmK
Read	Other specified diabetes mellitus with ophthalmic complicatn	C105y
Read	Recommendation self-refer for diabetes structured education	67W1.
Read	Type II diabetes mellitus in remission	C10P1
Read	Seen by general practitioner special interest in diabetes	9Nl4.
Read	Diabetes Distress Scale 17 item	38QX.
Read	Diabetic mononeuropathy	F3y0.
Read	Diabetes monitoring admin.	XE2Nd
Read	Malnutrition-related diabetes mellitus	X40J7
Read	Diabetes insipidus, diabetes mellitus, optic atrophy and deafness	X008t
Read	Polyneuropathy in diabetes	F372.
Read	Non-insulin-dependent diabetes mellitus with renal comps	C1090
Read	Other specified diabetes mellitus with neurological comps	C106y
Read	Type 2 diabetes mellitus with peripheral angiopathy	C10FF
Read	Diabetes self-management plan review	661N4
Read	Did not complete XPERT diabetes structured education program	8I84.
Read	Insulin-dependent diabetes mellitus with ophthalmic comps	C1081
Read	Diabetes administration: [monitoring] or [clinic]	9OL..
Read	Diabetes mellitus induced by non-steroid drugs	C10H.
Read	Type 1 diabetes mellitus with gastroparesis	C10EQ
Read	Insulin dependent diabetes mellitus with multiple complicatn	C1083
Read	Diabetes mellitus with peripheral circulatory disorder	XE10I
Read	Diabetes structured education programme completed	XaX5D
Read	Diabetes type 1 review	66An.
Read	Diabetes structured education programme declined	XaNTH

Read	Diabetes clinic administration	XM1Rs
Read	Diabetes mellitus, adult onset, with ketoacidotic coma	C1031
Read	Diabetes with ketoacidosis - no coma	Xa3ee
Read	Type 1 diabetes mellitus with mononeuropathy	C10EB
Read	Insulin treated Type 2 diabetes mellitus	X40J6
Read	Type 1 diabetes mellitus with diabetic cataract	C10EF
Read	Diabetes mellitus, juvenile type, no mention of complication	C1000
Read	H/O: secondary diabetes mellitus	XaXZG
Read	Type 2 diabetes mellitus with hypoglycaemic coma	C10FD
Read	Referral to DESMOND diabetes structured programme	XaNTS
Read	Non-insulin-dependent diabetes mellitus with neuro comps	C1092
Read	Diabetes insipidus, diabetes mellitus, optic atrophy and deafness	PKyP.
SNOMED	Atherosclerosis, deafness, diabetes, epilepsy, nephropathy syndrome	720519003
SNOMED	Proteinuria due to type 2 diabetes mellitus	1.57141E+14
SNOMED	Did not complete dose adjustment for normal eating diabetes structured education programme	3.06981E+14
SNOMED	Did not complete DESMOND (diabetes education and self management for ongoing and newly diagnosed) structured programme	3.06991E+14
SNOMED	Diabetes self-monitoring health education	398773005
SNOMED	Type I diabetes mellitus with neuropathic arthropathy	4.3711E+13
SNOMED	Did not complete diabetes education and self management for ongoing and newly diagnosed structured programme	3.07001E+14
SNOMED	Insulin dependent diabetes mellitus with peripheral angiopathy	314892000
SNOMED	Diabetes mellitus	267467004
SNOMED	Unstable type I diabetes mellitus	8.2881E+13
SNOMED	Severe nonproliferative retinopathy with venous beading of retina due to diabetes mellitus	870420005
SNOMED	Proliferative retinopathy with type 2 diabetes mellitus	1.501E+12
SNOMED	Diabetes education and self management for ongoing and newly diagnosed structured programme completed	3.06521E+14
SNOMED	Referral to diabetes special interest general practitioner	3.34711E+14
SNOMED	Diabetes mellitus with ophthalmic manifestation	190343002
SNOMED	High risk proliferative retinopathy with clinically significant macula edema due to diabetes mellitus	399874002
SNOMED	Diabetes monitoring telephone invite (procedure)	185760009
SNOMED	Did not attend expert patient education versus routine treatment diabetes structured education programme	3.07221E+14
SNOMED	Mild nonproliferative retinopathy co-occurrent and due to secondary diabetes mellitus (disorder)	3.68711E+14
SNOMED	Did not attend diabetes structured education programme	3.06881E+14
SNOMED	Type I diabetes mellitus without complication (disorder)	313435000
SNOMED	Diabetes mellitus NOS with ketoacidosis	5.93661E+14
SNOMED	Type I diabetes mellitus in remission	8.88201E+14
SNOMED	Diabetes mellitus, adult onset, with other specified manifestation	6.58031E+14
SNOMED	Diabetes mellitus: [juvenile] or [insulin dependent]	154673001
SNOMED	Brittle diabetes mellitus (finding)	11530004
SNOMED	History of admission in last year for diabetes foot problem	309635005
SNOMED	Diabetes mellitus, adult onset, with peripheral circulatory disorder	190356009
SNOMED	Secondary pancreatic diabetes mellitus	1.04031E+14
SNOMED	Hyperosmolar non-ketotic state due to diabetes mellitus	310505005
SNOMED	Diabetes care by hospital only	367269003
SNOMED	Peripheral circulatory disorder associated with type I diabetes mellitus (disorder)	421365002
SNOMED	Diabetes monitored	185762001
SNOMED	Expert patient education versus routine treatment diabetes structured education programme completed	3.06541E+14
SNOMED	Advanced retinal disease co-occurrent and due to diabetes mellitus	311782002
SNOMED	Diabetes: [peripheral circulatory disease] or [gangrene]	154687001
SNOMED	Type II diabetes mellitus with neurological complications	8.3071E+13
SNOMED	Referral to community diabetes specialist nurse declined	8.38731E+14
SNOMED	Type II diabetes mellitus with gangrene (disorder)	190390000
SNOMED	Quiescent proliferative retinopathy due to diabetes mellitus	312908007

SNOMED	Diabetes mellitus induced by non-steroid drugs	1.22421E+14
SNOMED	Vitreous haemorrhage due to diabetes mellitus	312910009
SNOMED	Macroalbuminuric nephropathy due to diabetes mellitus	445170001
SNOMED	At risk of ulcer of foot due to diabetes mellitus	714664001
SNOMED	Diabetes mellitus due to insulin receptor antibodies	9.9161E+13
SNOMED	Diabetes resolved	315051004
SNOMED	Diabetic foot ulcer associated with type 2 diabetes mellitus	1.521E+12
SNOMED	Diabetes-pancreatic exocrine dysfunction syndrome	609575003
SNOMED	Referral to diabetes nurse	312856000
SNOMED	Did not complete dose adjustment for normal eating diabetes structured education programme	3.06961E+14
SNOMED	Mild nonproliferative retinopathy of left eye due to diabetes mellitus type 1	1.67459E+16
SNOMED	Bronzed diabetes	86781004
SNOMED	Macular oedema due to type 2 diabetes mellitus	769220000
SNOMED	Diabetes mellitus type 2 in nonobese [Ambiguous]	55626003
SNOMED	Non-insulin dependent diabetes mellitus	190323008
SNOMED	Under care of diabetes specialist nurse	1.05491E+14
SNOMED	Optic papillopathy due to diabetes mellitus	314537004
SNOMED	Type I diabetes mellitus with hypoglycemic coma (disorder)	314771006
SNOMED	Other specified diabetes mellitus with peripheral circulatory complications	190360007
SNOMED	Dyslipidemia due to type 2 diabetes mellitus	7.61E+11
SNOMED	Post hypoglycemic hyperglycemia due to diabetes mellitus	398140007
SNOMED	Type 1 diabetes mellitus with persistent microalbuminuria	8.3021E+13
SNOMED	Type II diabetes mellitus with nephropathy	190398007
SNOMED	Type II diabetes mellitus with ophthalmic complications	4.3741E+13
SNOMED	Proliferative retinopathy with diabetes mellitus	59276001
SNOMED	Maturity onset diabetes of the young, type 1 (disorder)	609562003
SNOMED	Fibrocalculous pancreatic diabetes	9.9261E+13
SNOMED	Did not attend diabetes education and self management for ongoing and newly diagnosed structured programme	3.07181E+14
SNOMED	Mastopathy due to diabetes mellitus	724136006
SNOMED	Moderate nonproliferative retinopathy of right eye due to diabetes mellitus type 1	1.66975E+16
SNOMED	Small vessel disease due to type 1 diabetes mellitus	426907004
SNOMED	Hypertension in chronic kidney disease stage 4 due to type II diabetes mellitus	1.40111E+14
SNOMED	Bilateral mild nonproliferative retinopathy due to diabetes mellitus type 2	1.67459E+16
SNOMED	Type I diabetes mellitus with diabetic cataract	4.3681E+13
SNOMED	Asymmetric polyneuropathy co-occurrent and due to diabetes mellitus	230576004
SNOMED	Stable treated proliferative retinopathy of left eye due to diabetes mellitus	816962002
SNOMED	Erectile dysfunction due to diabetes mellitus	8.67891E+14
SNOMED	Stasis ulcer due to type 2 diabetes mellitus	9.7621E+13
SNOMED	Joint consultation with practice nurse and community diabetes specialist nurse	9.56791E+14
SNOMED	Blindness co-occurrent and due to type 2 diabetes mellitus	6.0951E+13
SNOMED	Type II diabetes mellitus with peripheral angiopathy	8.3191E+13
SNOMED	Diabetes care plan agreed	417317008
SNOMED	Type 2 diabetes mellitus risk assessment invitation SMS (short message service) text message	1.06551E+15
SNOMED	Diabetes mellitus diet education	284350006
SNOMED	Mononeuropathy multiplex due to diabetes mellitus	35777006
SNOMED	Diabetes + neuropathy	154683002
SNOMED	Health education - diabetes	407584001
SNOMED	Diabetes, hypogonadism, deafness, intellectual disability syndrome	816067005
SNOMED	Patient offered diabetes structured education programme	1.87831E+14
SNOMED	Moderate nonproliferative retinopathy due to type 1 diabetes mellitus	1.38891E+14
SNOMED	Maturity onset diabetes mellitus in young	400971001
SNOMED	Intellectual disability, craniofacial dysmorphism, hypogonadism, diabetes mellitus syndrome	722454003

SNOMED	Neuropathic ulcer of midfoot due to type 2 diabetes mellitus	789570006
SNOMED	Type I diabetes mellitus - poor control	4.3611E+13
SNOMED	Other specified diabetes mellitus with multiple complications	6.58001E+14
SNOMED	Insulin dependent diabetes mel	267469001
SNOMED	Hyperosmolar non-ketotic state in type 2 diabetes mellitus	394511005
SNOMED	Diabetes foot care clinic	702848001
SNOMED	Ulcer of midfoot due to type 1 diabetes mellitus	789572003
SNOMED	Diabetes monitoring administration (administrative concept)	3.341E+12
SNOMED	Retinal microaneurysm co-occurrent and due to diabetes mellitus	25412000
SNOMED	Infection of foot due to diabetes mellitus	419100001
SNOMED	Moderate nonproliferative retinopathy of left eye co-occurrent and due to diabetes mellitus	769186002
SNOMED	Diabetes mellitis with nephropathy NOS	190342007
SNOMED	Proliferative retinopathy of left eye with diabetes mellitus	770766000
SNOMED	Pre-existing type 2 diabetes mellitus in pregnancy	609567009
SNOMED	Chronic kidney disease stage 3 due to drug induced diabetes mellitus	3.68441E+14
SNOMED	Transition of diabetes care options discussed	1.70061E+14
SNOMED	Other specified diabetes mellitus with coma	6.30521E+14
SNOMED	Retinal abnormality - non-diabetes	141197003
SNOMED	Attended DESMOND diabetes structured education programme	2.76661E+14
SNOMED	Cheiroarthropathy due to diabetes mellitus type 1	3.68541E+14
SNOMED	Dyslipidemia due to type 1 diabetes mellitus	3.68551E+14
SNOMED	Attended expert patient education versus routine treatment structured diabetes programme	3.06411E+14
SNOMED	Peripheral neuropathy co-occurrent and due to type 2 diabetes mellitus	1.511E+12
SNOMED	Cheiroarthropathy due to diabetes mellitus type 2	3.68591E+14
SNOMED	Diabetes monitoring first letter (regime/therapy)	153935008
SNOMED	Insulin-dependent diabetes mellitus with neurological complications	190365002
SNOMED	Type II diabetes mellitus with mononeuropathy	314370005
SNOMED	Diabetes-deafness syndrome maternally transmitted (disorder)	237619009
SNOMED	Sensory neuropathy due to diabetes mellitus	127011001
SNOMED	Type I diabetes mellitus with ketoacidosis	371055001
SNOMED	Type II diabetes mellitus with complication	371056000
SNOMED	Provision of written information about gestational diabetes	8.37811E+14
SNOMED	Ulcer of foot due to diabetes mellitus	371087003
SNOMED	Diabetic dermopathy associated with diabetes mellitus type 2	1.531E+12
SNOMED	Pancreatic hypoplasia, diabetes mellitus, congenital heart disease syndrome	722206009
SNOMED	Under care of hospital-based diabetes specialist nurse	1.02456E+15
SNOMED	Diarrhea co-occurrent and due to diabetes mellitus	38205001
SNOMED	Rubeosis faciei due to diabetes mellitus	238984005
SNOMED	Congenital lipoatrophic diabetes	35824007
SNOMED	Severe nonproliferative retinopathy of bilateral eyes due to diabetes mellitus type 2	1.6747E+16
SNOMED	Type II diabetes mellitus with ulcer	4.3771E+13
SNOMED	Dermopathy due to diabetes mellitus	238982009
SNOMED	[X]Glomerular disorders in diabetes mellitus	198493003
SNOMED	Attends diabetes monitoring	185753003
SNOMED	Lumbosacral radiculoplexus neuropathy due to diabetes mellitus	39058009
SNOMED	Retinal ischemia due to type 2 diabetes mellitus	1.04961E+14
SNOMED	Has diabetes identity card	9.90751E+14
SNOMED	Diabetes mellitus with persistent proteinuria	401088000
SNOMED	Symmetric proximal motor neuropathy due to diabetes mellitus	39127005
SNOMED	Cranial nerve palsy with type I diabetes mellitus	770098001
SNOMED	Declined to register for access to online diabetes self-management application	1.43941E+14
SNOMED	Non-insulin-dependent diabetes mellitus with retinopathy	190391001

SNOMED	Diabetes Distress Scale 17 item	9.09441E+14
SNOMED	Persistent macular oedema due to diabetes mellitus	870529009
SNOMED	Latent autoimmune diabetes mellitus in adult (disorder)	426875007
SNOMED	[X]Unspecified diabetes mellitus with renal complications	3.97201E+14
SNOMED	Type 1 diabetes mellitus with mononeuropathy	314368001
SNOMED	Mild nonproliferative retinopathy of both eyes due to diabetes mellitus type 1	1.67454E+16
SNOMED	Other specified diabetes mellitus with other specified complications	190420007
SNOMED	Type 2 diabetes mellitus controlled by diet	1.64971E+14
SNOMED	Retinopathy due to unstable diabetes mellitus type 1	706894000
SNOMED	Neuropathy due to diabetes mellitus	230572002
SNOMED	Type I diabetes mellitus with renal complications	4.3531E+13
SNOMED	Diabetes mellitus with other specified manifestation	6.58011E+14
SNOMED	Intraretinal microvascular anomaly due to diabetes mellitus	399868002
SNOMED	Complication due to diabetes mellitus	74627003
SNOMED	Type 1 diabetes mellitus with persistent proteinuria	8.3011E+13
SNOMED	Visually threatening retinopathy due to diabetes mellitus	399871005
SNOMED	Discharged from care of diabetes specialist nurse	1.91781E+14
SNOMED	Provision of diabetes identity card	719570003
SNOMED	Emergency hospital admission to paediatric diabetes service	1.07861E+15
SNOMED	Hypoglycemia due to type 1 diabetes mellitus	8.4371E+13
SNOMED	Provision of written information about diabetes	8.37801E+14
SNOMED	DDS17 (Diabetes Distress Scale 17) score	9.10931E+14
SNOMED	Diabetic oculopathy associated with type I diabetes mellitus (disorder)	421165007
SNOMED	Photomyoclonus, diabetes mellitus, deafness, nephropathy and cerebral dysfunction	237612000
SNOMED	Provision of written information about diabetes and high blood pressure	9.44571E+14
SNOMED	Type 1 diabetes mellitus with persistent proteinuria	401109007
SNOMED	Did not attend diabetes education and self management for ongoing and newly diagnosed structured programme	3.07171E+14
SNOMED	Agreeing on diabetes care plan	703040004
SNOMED	Hypoglycaemic state in diabetes	8.3681E+13
SNOMED	Steroid-induced diabetes	9.9141E+13
SNOMED	Diabetes self-management behavior (observable entity)	405096004
SNOMED	Cranial nerve palsy co-occurrent and due to type 2 diabetes mellitus	8.7921E+13
SNOMED	Diabetes type (observable entity)	405751000
SNOMED	Diabetes mellitus, juvenile type, with renal manifestation	190339001
SNOMED	Diabetes monitoring second letter (regime/therapy)	153936009
SNOMED	Other specified diabetes mellitus with neurological complications	190352006
SNOMED	Diabetes mellitus in remission	8.88181E+14
SNOMED	Refuses diabetes monitoring	185754009
SNOMED	Dwarfism-hepatomegaly-obesity-juvenile diabetes syndrome	80660001
SNOMED	Neuropathic ulcer of foot due to diabetes mellitus	201251005
SNOMED	Severe nonproliferative retinopathy due to diabetes mellitus	312905005
SNOMED	Amyotrophy due to type 2 diabetes mellitus	427027005
SNOMED	Discharge from secondary care diabetes service	9.27821E+14
SNOMED	Type I diabetes mellitus maturity onset (disorder)	190372001
SNOMED	Disorder of eye co-occurrent and due to type 2 diabetes mellitus (disorder)	422099009
SNOMED	Panretinal photocoagulation for diabetes	413180006
SNOMED	Diabetes mellitus caused by non-steroid drugs without complication (disorder)	413183008
SNOMED	Referral to XPERT diabetes structured education programme	3.06771E+14
SNOMED	Education about lifestyle for risk of diabetes	8.87781E+14
SNOMED	Patient diabetes education review (regime/therapy)	415042006
SNOMED	Type I diabetes mellitus in remission (disorder)	703137001
SNOMED	Provision of diabetes clinical summary	8.31021E+14

SNOMED	Other specified diabetes mellitus with renal complications	5.93681E+14
SNOMED	Seen in community diabetes specialist clinic	416554009
SNOMED	Type 2 diabetes mellitus with nephropathy	314378003
SNOMED	Type I diabetes mellitus with hypoglycaemic coma	190377007
SNOMED	Referral to community diabetes specialist nurse	2.79531E+14
SNOMED	Diabetes mellitus in remission	8.88171E+14
SNOMED	Dermatitis due to drug induced diabetes mellitus	3.68171E+14
SNOMED	Assessment using diabetes quality of life questionnaire	8.38231E+14
SNOMED	Diabetes with peripheral circulatory disorders	982001
SNOMED	Preproliferative retinopathy of left eye co-occurrent and due to diabetes mellitus	769182000
SNOMED	Referral to diabetes support worker for diabetics of Asian origin	4.91731E+14
SNOMED	Nocturnal hypoglycemia due to diabetes mellitus	237635002
SNOMED	Acquired lipodystrophic diabetes	86907008
SNOMED	Did not attend DAFNE diabetes structured education programme	3.07141E+14
SNOMED	Diabetes dietitian	309417009
SNOMED	Type 1 diabetes mellitus with multiple complications	190366001
SNOMED	Other specified diabetes mellitus with ketoacidosis	190327009
SNOMED	Secondary diabetes mellitus without complication	1.13001E+14
SNOMED	Neuropathic ulcer of heel due to type 2 diabetes mellitus	789569005
SNOMED	Disorder of kidney co-occurrent and due to diabetes mellitus (disorder)	127013003
SNOMED	Acute painful polyneuropathy co-occurrent and due to diabetes mellitus	230574001
SNOMED	Diabetes with other complications	267474009
SNOMED	Unspecified diabetes mellitus with multiple complications	190383005
SNOMED	Small vessel disease due to type 2 diabetes mellitus	427134009
SNOMED	Congenital lipodystrophic diabetes	286289004
SNOMED	Retinal oedema due to type 2 diabetes mellitus	2.8331E+13
SNOMED	Diabetes monitoring invitation email (procedure)	1.08311E+15
SNOMED	Peripheral sensory neuropathy due to type 2 diabetes mellitus	1.10181E+14
SNOMED	Ischemic ulcer of foot due to diabetes mellitus	201250006
SNOMED	Severe nonproliferative retinopathy without macular edema co-occurrent and due to diabetes mellitus	399873008
SNOMED	End stage renal disease on dialysis due to type 2 diabetes mellitus	9.0791E+13
SNOMED	Foot ulcer due to drug induced diabetes mellitus	3.68181E+14
SNOMED	Ulcer of heel due to diabetes mellitus	789562001
SNOMED	Nonproliferative retinopathy co-occurrent and due to type 1 diabetes mellitus	6.0961E+13
SNOMED	Neuropathy with neurologic complication due to diabetes mellitus	359611005
SNOMED	Attended diabetes structured education programme	1.70841E+14
SNOMED	Type I diabetes mellitus with arthropathy (disorder)	314893005
SNOMED	Exudative maculopathy co-occurrent and due to type 2 diabetes mellitus (disorder)	421779007
SNOMED	Chronic kidney disease stage 2 due to type 1 diabetes mellitus	9.0731E+13
SNOMED	Renal disorder associated with type I diabetes mellitus (disorder)	421893009
SNOMED	Diabetic chronic renal impairment due to type 2 diabetes mellitus	7.71E+11
SNOMED	Diabetes mellitus, adult onset, with ketoacidotic coma	190335007
SNOMED	[X]Other specified diabetes mellitus	191045007
SNOMED	History of proliferative retinopathy due to diabetes mellitus	1.68909E+16
SNOMED	Diabetes care by hospital only	7.04471E+14
SNOMED	Insulin dependent diabetes mellitus type IA	23045005
SNOMED	Megaloblastic anaemia, thiamine-responsive, with diabetes mellitus and sensorineural deafness	237617006
SNOMED	Diabetes mellitus due to insulin receptor antibodies	408542006
SNOMED	Diabetes monitoring higher risk albumin excretion	1.99491E+14
SNOMED	Persistent proteinuria associated with type II diabetes mellitus (disorder)	421986006
SNOMED	Polyneuropathy associated with juvenile-onset diabetes mellitus	422297002
SNOMED	Femoral mononeuropathy co-occurrent and due to diabetes mellitus	361216007

SNOMED	Gangrene associated with diabetes mellitus	422275004
SNOMED	Type I diabetes mellitus with ulcer	4.3581E+13
SNOMED	Hyperglycemic crisis in diabetes mellitus	441656006
SNOMED	Fibrocalculous pancreatic diabetes	1.22481E+14
SNOMED	Motor polyneuropathy co-occurrent and due to diabetes mellitus	126535008
SNOMED	Bilateral severe nonproliferative retinopathy of eyes due to diabetes mellitus type 1	1.67471E+16
SNOMED	Type I diabetes mellitus with nephropathy	4.3661E+13
SNOMED	Nonproliferative retinopathy co-occurrent and due to diabetes mellitus	390834004
SNOMED	[X]Pre-existing diabetes mellitus, unspecified	4.49851E+14
SNOMED	Microalbuminuric nephropathy due to diabetes mellitus	236499007
SNOMED	Drug-induced diabetes mellitus	408544007
SNOMED	[X]Pre-existing diabetes mellitus, unspecified	200505002
SNOMED	At moderate risk of ulcer of left foot due to diabetes mellitus	863881000
SNOMED	Diabetes mellitus, juvenile type, with unspecified complication	190423009
SNOMED	Moderate nonproliferative retinopathy due to secondary diabetes mellitus	3.68741E+14
SNOMED	Pre-existing diabetes mellitus in childbirth	1.06281E+14
SNOMED	Severe hyperglycaemia due to diabetes mellitus	237621004
SNOMED	Retinal microaneurysm of right eye with diabetes mellitus	770581008
SNOMED	Sensory polyneuropathy due to diabetes mellitus	789585000
SNOMED	Chronic kidney disease stage 5 due to drug induced diabetes mellitus	3.68461E+14
SNOMED	Diabetes self-management plan agreed	8.11981E+14
SNOMED	Thoracic radiculopathy due to diabetes mellitus	230579006
SNOMED	Moderate nonproliferative retinopathy of left eye due to diabetes mellitus type 2	1.67461E+16
SNOMED	Type I diabetes mellitus	4.3521E+13
SNOMED	Referral to diabetes structured education programme	1.70311E+14
SNOMED	Type I diabetes mellitus with ophthalmic complications	4.3541E+13
SNOMED	Osteomyelitis due to type 1 diabetes mellitus	7.2041E+13
SNOMED	Type II diabetes mellitus with persistent microalbuminuria	401112005
SNOMED	Attended diabetes structured education program	413597006
SNOMED	Polyneuropathy co-occurrent and due to type 1 diabetes mellitus (disorder)	713705003
SNOMED	Protein-deficient diabetes mellitus	57886004
SNOMED	Type I diabetes mellitus with retinopathy	4.3601E+13
SNOMED	Referral to diabetes special interest general practitioner	3.34731E+14
SNOMED	Retinal abnormality - diabetes-related	309595004
SNOMED	Did not complete expert patient education versus routine treatment diabetes structured education programme	3.07041E+14
SNOMED	Neuropathy due to brittle type I diabetes mellitus	706891008
SNOMED	Acute painful neuropathy due to diabetes mellitus	193183000
SNOMED	Disorder due to well controlled type 2 diabetes mellitus	2.01E+11
SNOMED	Ulcer of right foot due to diabetes mellitus	860977000
SNOMED	Developmental delay, epilepsy, neonatal diabetes syndrome	721088003
SNOMED	Type I diabetes mellitus with peripheral angiopathy	4.3691E+13
SNOMED	No evidence of disorder of kidney due to diabetes mellitus	874908001
SNOMED	Diabetes mellitus, juvenile type, with unspecified complication	6.58081E+14
SNOMED	Type II diabetes mellitus	4.3721E+13
SNOMED	Diabetes mellitus autosomal dominant type 2	390716007
SNOMED	Clinically significant macular oedema of right eye due to diabetes mellitus	769221001
SNOMED	Type 2 diabetes mellitus with ketoacidosis	8.3251E+13
SNOMED	Ulcer of heel due to type 1 diabetes mellitus	789571005
SNOMED	Diabetic autonomic neuropathy associated with type 2 diabetes mellitus	423263001
SNOMED	Referral to diabetes clinic	8.09391E+14
SNOMED	[X]Glomerular disorders in diabetes mellitus	4.25911E+14
SNOMED	Attended diabetes structured education programme	1.75501E+14

SNOMED	Perceived control of insulin-dependent diabetes	304752002
SNOMED	Type II diabetes mellitus with mononeuropathy	4.3831E+13
SNOMED	Referral to diabetes education and self management for ongoing and newly diagnosed diabetes structured programme	3.06761E+14
SNOMED	Type II diabetes mellitus with polyneuropathy	8.3151E+13
SNOMED	Type 1 diabetes mellitus well controlled	444074000
SNOMED	Traction retinal detachment with type 2 diabetes mellitus	8.2541E+13
SNOMED	Referral to diabetes preconception counselling clinic	1.74941E+14
SNOMED	Proliferative retinopathy of right eye due to diabetes mellitus type 1	1.67487E+16
SNOMED	Diabetes treatment satisfaction questionnaire	142605003
SNOMED	Insulin treated Type 2 diabetes mellitus	4.3911E+13
SNOMED	Diabetes clinical management plan	736284000
SNOMED	Cataract due to diabetes mellitus	43959009
SNOMED	Type II diabetes mellitus with arthropathy	4.3891E+13
SNOMED	Type II diabetes mellitus with diabetic cataract	190400006
SNOMED	Understands diet - diabetes	147984007
SNOMED	Insulin-treated non-insulin-dependent diabetes mellitus	154688006
SNOMED	Diabetes structured education programme declined	3.06591E+14
SNOMED	Persistent proteinuria associated with type I diabetes mellitus (disorder)	420514000
SNOMED	Mixed sensorimotor polyneuropathy co-occurrent and due to diabetes mellitus	126534007
SNOMED	End stage renal disease on dialysis due to drug induced diabetes mellitus	3.68471E+14
SNOMED	Diabetes mellitus, adult onset, with no mention of complication	5.32411E+14
SNOMED	Type II diabetes mellitus with neurological complications	190387006
SNOMED	Other specified diabetes mellitus with unspecified complications	6.58101E+14
SNOMED	Hyperosmolarity co-occurrent and due to drug induced diabetes mellitus	3.67261E+14
SNOMED	Referral to community diabetes service	8.11401E+14
SNOMED	Nephrotic syndrome due to type 2 diabetes mellitus	7.1441E+13
SNOMED	Other drugs used in diabetes	326036003
SNOMED	Malnutrition related diabetes mellitus	75524006
SNOMED	Ulcer of ankle due to type 2 diabetes mellitus	8.7441E+13
SNOMED	Diabetes Type II (non-insulin-dependent) associated with renal failure - European Dialysis and Transplant Association	4.53171E+14
SNOMED	[X]Malnutrition-related diabetes mellitus with other specified complications	4.54441E+14
SNOMED	Referral to diabetes structured education programme	1.74961E+14
SNOMED	Type II diabetes mellitus with multiple complications	4.3761E+13
SNOMED	Peripheral circulatory disorder associated with non-insulin dependent diabetes mellitus	422166005
SNOMED	Proliferative retinopathy with optic disc neovascularisation due to diabetes mellitus	232021008
SNOMED	Nonproliferative retinopathy of both eyes due to diabetes mellitus type 1	1.67467E+16
SNOMED	Type 2 diabetes mellitus with peripheral angiopathy (disorder)	314902007
SNOMED	Diabetes wellbeing questionnaire	273413007
SNOMED	Diabetes monitoring admin.NOS	3.2911E+13
SNOMED	Retinopathy co-occurrent and due to diabetes mellitus (disorder)	4855003
SNOMED	Type II diabetes mellitus with exudative maculopathy	408417004
SNOMED	Ulcer of right foot co-occurrent and due to diabetes mellitus type 2	1.06617E+16
SNOMED	Mild nonproliferative retinopathy due to type 2 diabetes mellitus	1.38911E+14
SNOMED	Hyperglycaemia due to diabetes mellitus	822995009
SNOMED	Malnutrition-related diabetes mellitus with multiple complications	190411003
SNOMED	Transition of diabetes care options discussed	1.74721E+14
SNOMED	Diabetes care plan	1.36234E+15
SNOMED	Unstable diabetes	147994002
SNOMED	Type II diabetes mellitus with diabetic cataract	4.3871E+13
SNOMED	Hyperosmolality due to uncontrolled type 1 diabetes mellitus	428896009
SNOMED	Autonomic neuropathy due to diabetes	50620007
SNOMED	Stable treated proliferative retinopathy of right eye due to diabetes mellitus	816961009

SNOMED	Diabetes self management plan (qualifier value)	5.10741E+14
SNOMED	Declined consent for diabetes year of care programme	5.12581E+14
SNOMED	At risk of ulcer of left foot due to diabetes mellitus	863882007
SNOMED	Very severe nonproliferative retinopathy of left eye due to diabetes mellitus	769191001
SNOMED	Type I diabetes mellitus with polyneuropathy	8.2951E+13
SNOMED	Severe nonproliferative retinopathy of right eye due to diabetes mellitus type 2	1.67471E+16
SNOMED	Diabetes + periph.circulat.dis	267473003
SNOMED	Attended XPERT (expert patient education versus routine treatment) diabetes structured education programme	3.06401E+14
SNOMED	Diabetes clinic administration	2.3111E+13
SNOMED	Did not complete diabetes structured education programme	3.06951E+14
SNOMED	Neuropathy due to type 2 diabetes mellitus	3.68581E+14
SNOMED	MODY - Maturity onset diabetes in youth type I	28453007
SNOMED	Ischaemic maculopathy with diabetes mellitus	314014002
SNOMED	Did not attend dose adjustment for normal eating diabetes structured education programme	3.07161E+14
SNOMED	Diabetes mellitus associated with hormonal aetiology	59079001
SNOMED	Proliferative retinopathy with iris neovascularization due to diabetes mellitus	312909004
SNOMED	Type I diabetes mellitus with mononeuropathy	4.3641E+13
SNOMED	Insulin treated Type 2 diabetes mellitus	5.0241E+13
SNOMED	Maculopathy of left eye due to diabetes mellitus	769245002
SNOMED	Diabetes mellitus with nephropathy NOS	5.93691E+14
SNOMED	Type 1 diabetes mellitus with renal complications	190363009
SNOMED	Ischemic foot ulcer due to type 2 diabetes mellitus	1.40521E+14
SNOMED	Posttransplant diabetes mellitus	445260006
SNOMED	Insulin receptor defect with insulin-resistant diabetes mellitus and acanthosis nigricans	237606005
SNOMED	Forefoot ulcer due to type 1 diabetes mellitus	8.7491E+13
SNOMED	Addison's disease, struma lymphomatosa and insulin-dependent diabetes mellitus	74263009
SNOMED	Attended DAFNE (dose adjustment for normal eating) diabetes structured education programme	3.06441E+14
SNOMED	Ulcer of left foot due to diabetes mellitus	860978005
SNOMED	Skin ulcer associated with diabetes mellitus	422183001
SNOMED	Hypogonadism, diabetes mellitus, alopecia, mental retardation and electrocardiographic abnormalities	237616002
SNOMED	Joint consultation with General Practitioner and community diabetes specialist nurse	9.56931E+14
SNOMED	Permanent neonatal diabetes mellitus	609565001
SNOMED	Retinal abnormality - non-diabetes	309596003
SNOMED	Diabetes mellitus, adult onset, with ophthalmic manifestation	190346005
SNOMED	Proximal tubulopathy, diabetes mellitus, cerebellar ataxia syndrome	724062000
SNOMED	Maturity-onset diabetes of the young, type 3	609570008
SNOMED	On subcutaneous insulin for diabetes mellitus	473189005
SNOMED	Diabetes care by hospital only	367040002
SNOMED	Diabetes treatment satisfaction questionnaire	273412002
SNOMED	Provision of written information about diabetes and high hemoglobin A1c level	719943000
SNOMED	H/O: Admission in last year for diabetes foot problem	138832004
SNOMED	Diabetes mellitus type 2 in nonobese	359642000
SNOMED	Diabetes mellitus, juvenile type, with neurological manifestation	190350003
SNOMED	Glaucoma suspect due to diabetes mellitus type 2	1003605004
SNOMED	Heel AND/OR midfoot ulcer due to type 2 diabetes mellitus	8.7451E+13
SNOMED	Proliferative retinopathy with type 1 diabetes mellitus	6.0971E+13
SNOMED	Pre-existing diabetes mellitus, insulin-dependent	199229001
SNOMED	Hypoglycemia due to type 2 diabetes mellitus	1.20731E+14
SNOMED	Renal diabetes	236367002
SNOMED	Did not attend diabetes structured education programme	3.06871E+14
SNOMED	Diabetes monitoring default	185755005
SNOMED	Nonproliferative retinopathy of right eye due to diabetes mellitus	816178004

SNOMED	Discharged from care of diabetes specialist nurse	417467000
SNOMED	Diabetes monitoring administration NOS (record artifact)	7.13681E+14
SNOMED	Type II diabetes mellitus with renal complications	4.3731E+13
SNOMED	Vitreous haemorrhage of right eye due to diabetes mellitus	770361008
SNOMED	Did not complete diabetes structured education programme	3.06931E+14
SNOMED	Advice about diabetes and driving	8.84071E+14
SNOMED	Diabetic erectile dysfunction associated with type 1 diabetes mellitus	6.91E+11
SNOMED	Foot abnormality - diabetes-related	164495006
SNOMED	Other specified diabetes mellitus with other specified complications	6.58051E+14
SNOMED	Intracapillary glomerulosclerosis due to diabetes mellitus	310387003
SNOMED	Diabetes mellitus with unspecified complication	6.58071E+14
SNOMED	Mild nonproliferative retinopathy of right eye co-occurrent and due to diabetes mellitus	769183005
SNOMED	Armanni-Ebstein kidney due to diabetes mellitus	792926007
SNOMED	Erectile dysfunction due to diabetes mellitus	8.67901E+14
SNOMED	Health education - diabetes	1.06861E+14
SNOMED	Type II diabetes mellitus with multiple complications	8.3081E+13
SNOMED	Has seen dietitian - diabetes	147983001
SNOMED	Education in self management of diabetes	5.21391E+14
SNOMED	Type II diabetes mellitus with neuropathic arthropathy	8.3211E+13
SNOMED	Diabetes mellitus NOS with no mention of complication	6.30481E+14
SNOMED	Did not complete dose adjustment for normal eating diabetes structured education programme	3.06971E+14
SNOMED	History of diabetes mellitus in child of subject	444094009
SNOMED	Diabetes type 1 review	2.79301E+14
SNOMED	Did not attend diabetes education and self management for ongoing and newly diagnosed structured programme	3.07191E+14
SNOMED	Ulcer of lower limb due to type 2 diabetes mellitus	1.10171E+14
SNOMED	Lipoatrophic diabetes mellitus without complication	1.22501E+14
SNOMED	DDS2 (Diabetes Distress Scale 2) score	9.09421E+14
SNOMED	End stage renal disease on dialysis due to type 1 diabetes mellitus	9.0771E+13
SNOMED	Type II diabetes mellitus with peripheral angiopathy	4.3881E+13
SNOMED	Education about lifestyle for risk of diabetes	8.87791E+14
SNOMED	Type II diabetes mellitus without complication	8.3131E+13
SNOMED	Type II diabetes mellitus with neuropathic arthropathy (disorder)	314904008
SNOMED	Education about self management of diabetes	698610002
SNOMED	Referral to dose adjustment for normal eating diabetes structured education programme	3.06721E+14
SNOMED	Diabetes mellitus with other specified manifestation	190417004
SNOMED	Epiphyseal dysplasia, multiple, with early onset diabetes mellitus	254066006
SNOMED	Type I diabetes mellitus with nephropathy	190376003
SNOMED	Cystoid macular edema due to diabetes mellitus	870421009
SNOMED	Chronic painful polyneuropathy due to diabetes mellitus	230575000
SNOMED	Hypoglycemic unawareness in type 1 diabetes mellitus	1.20711E+14
SNOMED	Gangrene associated with type I diabetes mellitus (disorder)	420825003
SNOMED	Insulin-dependent diabetes mellitus - poor control	190371008
SNOMED	Cellulitis of foot due to diabetes mellitus	200687002
SNOMED	Diabetes mellitus with persistent proteinuria	7.8431E+13
SNOMED	Renal disorder due to type 2 diabetes mellitus (disorder)	420279001
SNOMED	Retinal microaneurysm of left eye with diabetes mellitus	770582001
SNOMED	Referral to HeLP-Diabetes (Healthy Living for People with Diabetes) online self-management programme	1.10874E+15
SNOMED	X-PERT (expert patient education versus routine treatment) First Steps diabetes self-management programme completed	1.03329E+15
SNOMED	Type II diabetes mellitus with retinopathy	4.3791E+13
SNOMED	Loss of hypoglycemic warning due to diabetes mellitus	170766006
SNOMED	History of diabetes mellitus type 2	472969004
SNOMED	Moderate nonproliferative retinopathy of left eye due to diabetes mellitus type 1	1.66974E+16

SNOMED	Chronic painful neuropathy due to diabetes mellitus	193184006
SNOMED	Non-high-risk proliferative retinopathy with no macular oedema due to diabetes mellitus	399870006
SNOMED	Type I diabetes mellitus	4.7481E+13
SNOMED	Iritis of right eye co-occurrent and due to diabetes mellitus	768797001
SNOMED	Diabetes: practice programme	170774007
SNOMED	Glomerulosclerosis of kidney due to diabetes mellitus	707221002
SNOMED	Congenital lipoatrophic diabetes	237607001
SNOMED	Provision of written information about diabetes and high haemoglobin A1c level	9.44581E+14
SNOMED	Diabetic stage 5 chronic renal impairment associated with type 2 diabetes mellitus	7.11E+11
SNOMED	Autonomic neuropathy co-occurrent and due to type 1 diabetes mellitus	712882000
SNOMED	Provision of written information about diabetes and high cholesterol	715879000
SNOMED	Retinal venous beading of right eye co-occurrent and due to diabetes mellitus	770599000
SNOMED	Diffuse exudative maculopathy co-occurrent and due to diabetes mellitus	314010006
SNOMED	Mononeuropathy co-occurrent and due to diabetes mellitus (disorder)	230577008
SNOMED	Diabetes clinic administration (record artifact)	7.13691E+14
SNOMED	Referral to community diabetes specialist nurse	2.79521E+14
SNOMED	Diabetes with renal manifestations	21858001
SNOMED	Type 2 diabetes mellitus with hypoglycaemic coma	314772004
SNOMED	Type 2 diabetes mellitus in remission	8.88211E+14
SNOMED	Diabetes mellitus with ketoacidotic coma	190333000
SNOMED	Polyneuropathy co-occurrent and due to type 2 diabetes mellitus (disorder)	713706002
SNOMED	Chronic kidney disease stage 4 due to drug induced diabetes mellitus	3.68451E+14
SNOMED	Diabetes type 2 review	2.79341E+14
SNOMED	Cranial nerve palsy with diabetes mellitus	770095003
SNOMED	Diabetes administration: [monitoring] or [clinic]	153931004
SNOMED	Bird-headed dwarfism with progressive ataxia, insulin-resistant diabetes, goitre, and primary gonadal insufficiency	237614004
SNOMED	Nonproliferative retinopathy of right eye due to diabetes mellitus type 2	1.67451E+16
SNOMED	Maturity-onset diabetes of the young (disorder)	609561005
SNOMED	Gingival disease co-occurrent with diabetes mellitus	716362006
SNOMED	H/O: secondary diabetes mellitus	7.71571E+14
SNOMED	Type I diabetes mellitus with ophthalmic complications	190364003
SNOMED	Diabetic neuropathic arthropathy due to type 1 diabetes mellitus (disorder)	7.1771E+13
SNOMED	Pre-existing malnutrition-related diabetes mellitus	199231005
SNOMED	Referral to diabetes nurse	151850005
SNOMED	Secondary pancreatic diabetes mellitus without complication	1.22961E+14
SNOMED	Joint consultation with practice nurse and community diabetes specialist nurse	9.56781E+14
SNOMED	Referral to diabetes preconception counselling clinic	1.70281E+14
SNOMED	Diabetes monitoring verbal invite (procedure)	185759004
SNOMED	Type I diabetes mellitus with arthropathy	4.3701E+13
SNOMED	Diabetes wellbeing questionnaire	142604004
SNOMED	[EDTA] Diabetes Type I (insulin dependent) associated with renal failure	274589008
SNOMED	Osteomyelitis due to type 2 diabetes mellitus	7.2061E+13
SNOMED	Provision of written information about diabetes and high haemoglobin A1c level	9.44591E+14
SNOMED	Bilateral iritis due to diabetes mellitus	768799003
SNOMED	Multiple complications of type I diabetes mellitus (disorder)	422228004
SNOMED	Complication due to diabetes mellitus type 2	1.2811E+13
SNOMED	Refuses diabetes monitoring (regime/therapy)	153933001
SNOMED	Diabetes + eye manifestation (& [cataract] or [retinopathy])	154678005
SNOMED	Radiculoplexoneuropathy due to diabetes mellitus	724810001
SNOMED	Mild nonproliferative retinopathy co-occurrent and due to diabetes mellitus	312903003
SNOMED	[X]Malnutrition-related diabetes mellitus with other specified complications	190414006
SNOMED	Diabetes monitoring telephone invite	153939002

SNOMED	Advanced maculopathy co-occurrent and due to diabetes mellitus	193350004
SNOMED	Permanent neonatal diabetes mellitus with cerebellar agenesis syndrome	724067006
SNOMED	[X]Other specified diabetes mellitus	4.42541E+14
SNOMED	Maturity-onset diabetes of the young, type 10	609577006
SNOMED	Neuropathic ulcer of foot due to type 2 diabetes mellitus	1.40531E+14
SNOMED	DAFNE diabetes structured education programme completed	3.06471E+14
SNOMED	Referral to community diabetes specialist clinic	8.41231E+14
SNOMED	Hyperosmolar non-ketotic state in type 2 diabetes mellitus (disorder)	395204000
SNOMED	Steroid-induced diabetes mellitus without complication	190416008
SNOMED	Diabetes mellitus (disorder)	73211009
SNOMED	Cataract of left eye co-occurrent and due to diabetes mellitus (disorder)	768793002
SNOMED	At low risk of ulcer of left foot due to diabetes mellitus	863880004
SNOMED	Maturity onset diabetes of the young type 5	9.74131E+14
SNOMED	Retinal edema co-occurrent and due to diabetes mellitus	770323005
SNOMED	Lipodystrophic diabetes with partial lipoatrophy	75659004
SNOMED	Chronic kidney disease stage 3 associated with type 2 diabetes mellitus	7.31E+11
SNOMED	Insulin resistance in diabetes	237650006
SNOMED	Type I diabetes mellitus with gangrene (disorder)	190369008
SNOMED	[V]Dietary counseling in diabetes mellitus	4.41901E+14
SNOMED	Type II diabetes mellitus with arthropathy	8.3201E+13
SNOMED	Diabetes Treatment Satisfaction Questionnaire status version (assessment scale)	739663003
SNOMED	Gastroparesis with diabetes mellitus	713704004
SNOMED	Disorder of eye co-occurrent and due to type 1 diabetes mellitus (disorder)	739681000
SNOMED	Non centrally involved macular edema due to diabetes mellitus	871781003
SNOMED	Other specified diabetes mellitus with ketoacidosis	6.30491E+14
SNOMED	High risk proliferative retinopathy not amenable to photocoagulation due to diabetes mellitus	399869005
SNOMED	Steroid-induced diabetes	190447002
SNOMED	Diabetes mellitus, adult onset, with renal manifestation	190340004
SNOMED	Diabetes self-management plan review	8.32131E+14
SNOMED	Referral to type I diabetes structured education programme	7.54461E+14
SNOMED	Diabetes monitoring third letter (procedure)	185758007
SNOMED	History of small vessel disease due to diabetes mellitus	5.9801E+13
SNOMED	Hypertension in chronic kidney disease due to type II diabetes mellitus	7.1421E+13
SNOMED	Proliferative retinopathy of left eye due to diabetes mellitus type 1	1.67477E+16
SNOMED	Fibrocalculous pancreatic diabetes	2751001
SNOMED	Ketoacidosis in type I diabetes mellitus (disorder)	420270002
SNOMED	Insulin treated Type 2 diabetes mellitus	366909003
SNOMED	Vitreous haemorrhage of left eye due to diabetes mellitus	770362001
SNOMED	Provision of patient held diabetes record	763783006
SNOMED	Severe nonproliferative retinopathy of left eye due to diabetes mellitus type 1	1.67469E+16
SNOMED	Assessment using DTSQs (Diabetes Treatment Satisfaction Questionnaire status version)	765148004
SNOMED	Diabetes monitoring short message service text message second invitation (procedure)	1.06692E+15
SNOMED	Macular edema due to diabetes mellitus	312912001
SNOMED	Type I diabetes mellitus with neurological complications	4.3551E+13
SNOMED	Ulcer of midfoot due to diabetes mellitus	789568002
SNOMED	Type 1 diabetes mellitus with arthropathy	190380008
SNOMED	Alaninuria, microcephaly, dwarfism, enamel hypoplasia, diabetes mellitus syndrome	733072002
SNOMED	Preproliferative retinopathy of right eye due to diabetes mellitus	769181007
SNOMED	Diabetes: practice program	148000001
SNOMED	Diabetes monitoring invitation by SMS (short message service) text messaging	7.52141E+14
SNOMED	Type II diabetes mellitus uncontrolled (finding)	443694000
SNOMED	Malnutrition-related diabetes mellitus with peripheral circulatory complications	190410002

SNOMED	Mild nonproliferative retinopathy of left eye due to diabetes mellitus	769184004
SNOMED	Diabetes mellitus type 2 without retinopathy (disorder)	1.481E+12
SNOMED	Moderate nonproliferative retinopathy of right eye co-occurrent and due to diabetes mellitus	769185003
SNOMED	Diabetes foot care education	9.23371E+14
SNOMED	History of diabetes related lower limb amputation	735199000
SNOMED	Diabetes clinical management plan	1.16981E+14
SNOMED	Dietary education for type II diabetes mellitus	428274007
SNOMED	Disorder associated with type I diabetes mellitus	420868002
SNOMED	Referral to diabetes service	715159005
SNOMED	Type II diabetes mellitus with gangrene	4.3781E+13
SNOMED	High risk proliferative retinopathy due to diabetes mellitus	312907002
SNOMED	Did not complete expert patient education versus routine treatment diabetes structured education programme	3.07031E+14
SNOMED	Pineal hyperplasia AND diabetes mellitus syndrome	33559001
SNOMED	Newly diagnosed diabetes	405749004
SNOMED	Diabetes mellitus due to genetic defect in beta cell function	609568004
SNOMED	Erectile dysfunction with diabetes mellitus	770096002
SNOMED	Clinically significant macular edema due to diabetes mellitus	770097006
SNOMED	Retinal abnormality - non-diabetes	163998006
SNOMED	Autonomic neuropathy due to drug induced diabetes mellitus	3.68141E+14
SNOMED	Retinal venous beading of left eye with diabetes mellitus	770600002
SNOMED	Brittle type II diabetes mellitus (finding)	445353002
SNOMED	Provision of patient held diabetes record declined	763782001
SNOMED	Diabetes with diabetic complications	372069003
SNOMED	Referral to hospital diabetes antenatal clinic	770811007
SNOMED	Diabetes structured education program	3.08421E+14
SNOMED	Pseudotabes with diabetes mellitus	19378003
SNOMED	Seen in diabetes preconception counseling clinic	770752005
SNOMED	Venous beading of retina due to diabetes mellitus	399866003
SNOMED	Diabetes management plan given	148002009
SNOMED	Diabetes resolved	162661000
SNOMED	Severe malnutrition due to type 1 diabetes mellitus	7.2031E+13
SNOMED	Diabetes Treatment Satisfaction Questionnaire status version score	765147009
SNOMED	History of autosomal dominant diabetes mellitus	7.71521E+14
SNOMED	Pre-existing type 1 diabetes mellitus in pregnancy	609564002
SNOMED	Diabetes mellitus	191044006
SNOMED	Malnutrition-related diabetes mellitus with ophthalmic complications	190408004
SNOMED	Diabetes clinical pathway	8.51511E+14
SNOMED	Diabetes structured education programme completed	7.55491E+14
SNOMED	Provision of written information about diabetes and hypertension	715759002
SNOMED	Referral to diabetes education and self management for ongoing and newly diagnosed structured education programme declined	7.81171E+14
SNOMED	Multiple complications of type II diabetes mellitus	420414003
SNOMED	Primary microcephaly, epilepsy, permanent neonatal diabetes syndrome	782825008
SNOMED	Type I diabetes mellitus with neuropathic arthropathy	190381007
SNOMED	Diabetes Type I (insulin dependent) associated with renal failure - EDTA (European Dialysis and Transplant Association)	4.16751E+14
SNOMED	NIDDM with peripheral circulatory disorder	190359002
SNOMED	Type 1 diabetes mellitus with ketoacidosis	7.8441E+13
SNOMED	Type II diabetes mellitus	7.8451E+13
SNOMED	Diabetes + eye manifestation	267471001
SNOMED	Very severe nonproliferative retinopathy without macular edema co-occurrent and due to diabetes mellitus	399863006
SNOMED	History of maturity onset diabetes mellitus in young	472972006
SNOMED	Diabetes care plan declined	7.87071E+14
SNOMED	Type I diabetes mellitus with peripheral angiopathy	190379005

SNOMED	Neuropathy due to type 1 diabetes mellitus	789542009
SNOMED	Diet controlled diabetes mellitus	170745003
SNOMED	Diabetes mellitus, Addison's disease and myxedema	83728000
SNOMED	Assessment of self management of diabetes mellitus	870422002
SNOMED	Health education - diabetes	8.6561E+13
SNOMED	Inflammatory dermatosis due to type 1 diabetes mellitus	3.67211E+14
SNOMED	Chronic ulcer of skin due to type 1 diabetes mellitus	7.2141E+13
SNOMED	Maturity-onset diabetes of the young, type 6	609573005
SNOMED	Diabetes mellitus associated with receptor abnormality	42954008
SNOMED	Provision of written information about diabetes mellitus	715894001
SNOMED	Type I diabetes mellitus with nephropathy	314377008
SNOMED	Diabetes management using closed-loop delivery system (regime/therapy)	897238003
SNOMED	Ulcer of forefoot due to type 2 diabetes mellitus	8.7461E+13
SNOMED	Has diabetes identity card	9.66371E+14
SNOMED	Skin ulcer of toe due to diabetes mellitis type 1	1.06562E+16
SNOMED	Polyneuropathy associated with type II diabetes mellitus	421707005
SNOMED	Hyperosmolar non-ketotic state in type 2 diabetes mellitus	4.3921E+13
SNOMED	Lactic acidosis co-occurrent and due to diabetes mellitus (disorder)	735538002
SNOMED	Dermopathy due to type 1 diabetes mellitus	7.2021E+13
SNOMED	Referral to diabetes preconception counselling clinic	415269004
SNOMED	Peripheral vascular disorder co-occurrent and due to diabetes mellitus	421895002
SNOMED	Hypertension in chronic kidney disease stage 2 due to type II diabetes mellitus	1.40131E+14
SNOMED	Diabetes management plan given (regime/therapy)	170776009
SNOMED	Diabetes monitoring deleted	185761008
SNOMED	Referral to DAFNE diabetes structured education programme declined	8.60981E+14
SNOMED	History of nonproliferative retinopathy due to diabetes mellitus	1.68909E+16
SNOMED	Hypertension concurrent and due to end stage renal disease on dialysis due to type 1 diabetes mellitus	1.28001E+14
SNOMED	Diabetes mellitus NOS with ketoacidotic coma	190337004
SNOMED	Adult diabetes diet	34170007
SNOMED	Nephrotic syndrome due to type 1 diabetes mellitus	7.1721E+13
SNOMED	Diabetes self-management plan agreed	8.11991E+14
SNOMED	Diabetes mellitus service (qualifier value)	444913002
SNOMED	Rubeosis iridis co-occurrent and due to type 2 diabetes mellitus	8.2551E+13
SNOMED	Diabetes monitoring second letter (procedure)	185757002
SNOMED	Lipoatrophic diabetes mellitus without complication (disorder)	1.12991E+14
SNOMED	Hyperproinsulinemia (disorder)	237613005
SNOMED	Cardiomyopathy due to diabetes mellitus	788878000
SNOMED	Type I diabetes mellitus with neurological complications	8.2861E+13
SNOMED	Attended dose adjustment for normal eating structured diabetes education programme	3.06461E+14
SNOMED	Glucoaminophosphate diabetes	44673006
SNOMED	Ophthalmoplegia with diabetes mellitus	427943001
SNOMED	Maturity-onset diabetes of the young, type 7	609574004
SNOMED	Type I diabetes mellitus with retinopathy	8.2911E+13
SNOMED	Type I diabetes mellitus maturity onset	8.2921E+13
SNOMED	Type II diabetes mellitus - poor control	4.3801E+13
SNOMED	Disorder associated with type 2 diabetes mellitus	422014003
SNOMED	Diabetes education and self management for ongoing and newly diagnosed structured programme completed	3.06511E+14
SNOMED	Provision of diabetes clinical summary	8.29521E+14
SNOMED	Neurological disorder co-occurrent and due to type 1 diabetes mellitus (disorder)	421468001
SNOMED	Type I diabetes mellitus with hypoglycaemic coma	8.2971E+13
SNOMED	Anemia of diabetes	82980005
SNOMED	Did not commence remote structured diabetes education and support programme	1.09801E+15

SNOMED	Unstable diabetes	268599005
SNOMED	Type I diabetes mellitus with neuropathic arthropathy	8.3001E+13
SNOMED	Disorder of macula of bilateral eyes due to diabetes mellitus present	860712005
SNOMED	Proliferative retinopathy with retinal oedema due to type 2 diabetes mellitus	9.7341E+13
SNOMED	Joint consultation with General Practitioner and community diabetes specialist nurse	9.56921E+14
SNOMED	Disorder of eye due to malnutrition related diabetes mellitus	421256007
SNOMED	Diabetes mellitus NOS with other specified manifestation	190421006
SNOMED	Diabetes medication review	393084006
SNOMED	Type I diabetes mellitus with multiple complications	8.2871E+13
SNOMED	Diabetes mellitus NOS with peripheral circulatory disorder	5.93731E+14
SNOMED	Under care of community-based diabetes specialist nurse	1.02457E+15
SNOMED	Diabetes mellitus, juvenile type, with no mention of complication	267379000
SNOMED	Diabetes mellitus NOS with unspecified complication	190426001
SNOMED	Type II diabetes mellitus with retinopathy	8.3111E+13
SNOMED	Gingivitis co-occurrent with diabetes mellitus	709147009
SNOMED	Discharged from diabetes shared care programme	4.94961E+14
SNOMED	Did not complete XPERT diabetes structured education programme	3.07021E+14
SNOMED	Glaucoma due to type 2 diabetes mellitus	4.1911E+13
SNOMED	Unspecified diabetes mellitus with multiple complications	6.21631E+14
SNOMED	Type II diabetes mellitus with diabetic cataract	8.3181E+13
SNOMED	Steroid-induced diabetes	190415007
SNOMED	Diabetes resolved	1.64491E+14
SNOMED	Diabetic - poor control (finding)	268519009
SNOMED	Focal exudative maculopathy due to diabetes mellitus	314011005
SNOMED	DTSQc - Diabetes Treatment Satisfaction Questionnaire change version	739664009
SNOMED	Retinal oedema co-occurrent and due to type 1 diabetes mellitus	1.09171E+14
SNOMED	Under care of community-based diabetes specialist nurse	1.02458E+15
SNOMED	Type I diabetes mellitus with renal complications	8.2841E+13
SNOMED	Diabetes self-management plan review	8.10961E+14
SNOMED	H/O: diabetes mellitus	138717002
SNOMED	Diabetes mellitus autosomal dominant	390715006
SNOMED	Rare form of diabetes mellitus, NOS	111553002
SNOMED	Type 2 diabetes mellitus with ketoacidosis	7.8461E+13
SNOMED	Hyperosmolar coma due to type 1 diabetes mellitus	190330002
SNOMED	Secondary diabetes mellitus	1.10221E+14
SNOMED	Primary autoimmune diabetes mellitus	28032008
SNOMED	Diabetes mellitus NOS with other specified manifestation	6.58061E+14
SNOMED	Diabetes mellitus, adult onset, with unspecified complication	6.58091E+14
SNOMED	Acidosis due to type 1 diabetes mellitus	721283000
SNOMED	Diabetes-nephrosis syndrome	54181000
SNOMED	Type I diabetes mellitus with exudative maculopathy	408287009
SNOMED	Cataract of bilateral eyes co-occurrent and due to diabetes mellitus (disorder)	768794008
SNOMED	Has seen dietician - diabetes	170752001
SNOMED	Diabetes quality of life questionnaire score (observable entity)	8.51991E+14
SNOMED	Provision of written information about diabetes and driving	8.57921E+14
SNOMED	Provision of written information about diabetes and driving	8.57931E+14
SNOMED	No disorder of retina of left eye due to diabetes mellitus	860706000
SNOMED	Patient diabetes education review	1.74141E+14
SNOMED	Gastroparesis co-occurrent and due to type 2 diabetes mellitus (disorder)	713703005
SNOMED	Seen in hospital diabetes antenatal clinic	1.05422E+15
SNOMED	Ulcer of heel due to type 2 diabetes mellitus	789567007
SNOMED	Cheiroarthropathy due to diabetes mellitus	201723002

SNOMED	[X]Malnutrition-related diabetes mellitus with unspecified complications	4.25691E+14
SNOMED	Radiculoplexus neuropathy due to diabetes mellitus	39181008
SNOMED	Generalised autonomic neuropathy due to diabetes mellitus	860883001
SNOMED	Patient on maximal tolerated therapy for diabetes	1.04881E+14
SNOMED	Mononeuritis multiplex co-occurrent and due to diabetes mellitus	193141005
SNOMED	Type I diabetes mellitus with mononeuropathy	8.2941E+13
SNOMED	Chronic ulcer of left foot due to diabetes mellitus	860980004
SNOMED	Understands diet - diabetes	170753006
SNOMED	Diabetes mellitus with multiple complications (disorder)	3.85041E+14
SNOMED	Diabetes mellitus with unspecified complication	190422004
SNOMED	Recommendation to self-refer for diabetes structured education	9.77201E+14
SNOMED	Seen by diabetes specialist nurse	720685006
SNOMED	[X]Malnutrition-related diabetes mellitus with unspecified complications	190413000
SNOMED	Centrally involved macular oedema due to diabetes mellitus	871778008
SNOMED	Diabetes mellitus NOS with hyperosmolar coma	5.93671E+14
SNOMED	Diabetes structured education programme not available	8.85931E+14
SNOMED	Diffuse glomerulosclerosis of kidney due to diabetes mellitus	38046004
SNOMED	Proliferative retinopathy of right eye with diabetes mellitus	770765001
SNOMED	Traction detachment of retina due to diabetes mellitus	232023006
SNOMED	No retinopathy of right eye due to diabetes mellitus	860707009
SNOMED	Maturity onset diabetes of the young type 5	9.74141E+14
SNOMED	Ulcer of toe due to type 2 diabetes mellitus	1.40391E+14
SNOMED	Coma associated with diabetes mellitus	420662003
SNOMED	Type II diabetes mellitus with peripheral angiopathy	190401005
SNOMED	Diabetes wellbeing questionnaire	165205007
SNOMED	Diabetes mellitus caused by insulin receptor antibodies (disorder)	75682002
SNOMED	Diabetes monitoring injection site not checked	1.98111E+14
SNOMED	Hypoglycaemic coma due to diabetes mellitus	421725003
SNOMED	Patient offered diabetes structured education programme (regime/therapy)	416672007
SNOMED	Seen by diabetes specialist nurse	1.02452E+15
SNOMED	Provision of written information about diabetes and hypertension	9.44561E+14
SNOMED	Type 2 diabetes mellitus with gastroparesis	2.14001E+14
SNOMED	Diabetes mellitus in remission (disorder)	703136005
SNOMED	Education for self management of diabetes	5.21401E+14
SNOMED	Ischemia of retina due to diabetes mellitus	770324004
SNOMED	Diabetes mellitus with neurological manifestation	267382005
SNOMED	Diabetes monitoring injection site check declined	1.98101E+14
SNOMED	Diabetes mellitus associated with genetic syndrome (disorder)	5969009
SNOMED	Conversion to insulin by diabetes specialist nurse	8.76381E+14
SNOMED	Coma due to malnutrition-related diabetes mellitus	420996007
SNOMED	Diabetes self management	878859007
SNOMED	Skin ulcer of toe due to diabetes mellitus type 2	1.06563E+16
SNOMED	Diabetes mellitus with skin ulcer	371086007
SNOMED	Secondary diabetes mellitus (disorder)	8801005
SNOMED	Diabetes mellitus: [with renal manifestation] or [nephropathy]	190338009
SNOMED	Education about diabetes and driving	8.84081E+14
SNOMED	Hypoglycaemic coma in type 1 diabetes mellitus	421437000
SNOMED	DTSQs (Diabetes Treatment Satisfaction Questionnaire status version) score	1.08559E+15
SNOMED	Telehealth monitoring for diabetes mellitus (regime/therapy)	879782007
SNOMED	Other specified diabetes mellitus with coma	190336008
SNOMED	Impaired glucose tolerance in and individual with a heritable form of maturity onset diabetes in the young	14052004
SNOMED	Diabetes monitoring first letter (procedure)	185756006

SNOMED	Severe nonproliferative retinopathy of left eye due to diabetes mellitus type 2	1.67469E+16
SNOMED	Seen by diabetes specialist nurse	1.02451E+15
SNOMED	Lifestyle education regarding risk of diabetes	699826006
SNOMED	Skin ulcer associated with diabetes mellitus	1.09005E+15
SNOMED	Diabetes mellitus NOS with no mention of complication	190324002
SNOMED	Nonproliferative retinopathy co-occurrent and due to type 2 diabetes mellitus	1.551E+12
SNOMED	Diabetes structured education programme not available	8.85941E+14
SNOMED	Hypoglycaemia due to diabetes mellitus	237633009
SNOMED	Diabetes clinical management plan (qualifier value)	412777005
SNOMED	Type I diabetes mellitus	190362004
SNOMED	At high risk of ulcer of left foot due to diabetes mellitus	863886005
SNOMED	Diabetes clinical pathway	8.51501E+14
SNOMED	Ketoacidotic coma in type II diabetes mellitus (disorder)	421847006
SNOMED	At low risk of ulcer of right foot due to diabetes mellitus	863879002
SNOMED	Type I diabetes mellitus with ophthalmic complications	8.2851E+13
SNOMED	Ulcer of lower limb due to type 1 diabetes mellitus	1.10141E+14
SNOMED	Diabetes clinical management plan	1.21721E+14
SNOMED	Diabetes mellitus co-occurrent and due to cystic fibrosis (disorder)	426705001
SNOMED	Did not attend expert patient education versus routine treatment diabetes structured education programme	3.07201E+14
SNOMED	Diabetes monitoring admin.	8.731E+12
SNOMED	Type II diabetes mellitus	190384004
SNOMED	Referral to community diabetes antenatal clinic	1.05423E+15
SNOMED	Diabetes type 2 with gastroparesis	424989000
SNOMED	KPDM - ketosis-prone diabetes mellitus	890171006
SNOMED	Persistent microalbuminuria associated with type I diabetes mellitus	421305000
SNOMED	Chronic ulcer of right foot due to diabetes mellitus	860979002
SNOMED	History of diabetes mellitus type II	7.71691E+14
SNOMED	Primary microcephaly, mild intellectual disability, young-onset diabetes syndrome	782755007
SNOMED	Diabetic peripheral neuropathy associated with type I diabetes mellitus	1.561E+12
SNOMED	No disorder of macula of both eyes due to diabetes mellitus	860711003
SNOMED	Diabetes care plan agreed	1.91601E+14
SNOMED	Seen by diabetes special interest general practitioner	3.00661E+14
SNOMED	Type 2 diabetes mellitus risk assessment declined	1.06454E+15
SNOMED	Referral to expert patient education versus routine treatment diabetes structured education programme	3.06781E+14
SNOMED	Referral to DESMOND (diabetes education and self management for ongoing and newly diagnosed) diabetes structured programme	3.06741E+14
SNOMED	Type I diabetes mellitus with mononeuropathy	190374000
SNOMED	Secondary diabetes mellitus	1.22511E+14
SNOMED	At high risk of ulcer of right foot due to diabetes mellitus	863884008
SNOMED	Mononeuropathy co-occurrent and due to type 1 diabetes mellitus (disorder)	420918009
SNOMED	Referral to dose adjustment for normal eating diabetes structured education programme	3.06711E+14
SNOMED	Renal cysts and diabetes syndrome	446641003
SNOMED	Hypoglycaemic coma in type 2 diabetes mellitus	421164006
SNOMED	Diabetes structured education programme declined	3.06601E+14
SNOMED	Vitreous hemorrhage due to type 1 diabetes mellitus	1.04951E+14
SNOMED	Attending diabetes clinic	185502001
SNOMED	Prader-Willi syndrome and diabetes	37355009
SNOMED	Diabetes mellitus with peripheral circulatory disorder	267383000
SNOMED	Wet gangrene of foot due to diabetes mellitus	402864004
SNOMED	Attended expert patient education versus routine treatment structured diabetes programme	3.06421E+14
SNOMED	Mononeuropathy co-occurrent and due to type 2 diabetes mellitus (disorder)	420436000
SNOMED	Type II diabetes mellitus risk assessment verbal invitation	1.06549E+15
SNOMED	History of diabetes mellitus type I	472970003

SNOMED	Unstable diabetes	170764009
SNOMED	Fibrocalculous pancreatic diabetes	408545008
SNOMED	Attended dose adjustment for normal eating structured diabetes education programme	3.06451E+14
SNOMED	Diabetes mellitus NOS with neurological manifestation	6.24691E+14
SNOMED	Type I diabetes mellitus with hypoglycaemic coma	4.3671E+13
SNOMED	Remote diabetes structured education and support programme completed	1.09714E+15
SNOMED	Type II diabetes mellitus with gastroparesis	1.98671E+14
SNOMED	Patient on maximal tolerated therapy for diabetes	1.07421E+14
SNOMED	Postpancreatectomy diabetes mellitus	303059007
SNOMED	Pineal hyperplasia, insulin-resistant diabetes mellitus and somatic abnormalities	1.22541E+14
SNOMED	Type II diabetes mellitus with hypoglycaemic coma	4.3861E+13
SNOMED	Seen by general practitioner with special interest in diabetes	3.00641E+14
SNOMED	Diabetic neuropathic arthropathy associated with type 2 diabetes mellitus	7.81E+11
SNOMED	Pre-existing type 2 diabetes mellitus	199230006
SNOMED	Diabetes structured education programme completed	3.00501E+14
SNOMED	Seen in community diabetes specialist clinic	1.92191E+14
SNOMED	Diabetes mellitus NOS with ketoacidosis	190328004
SNOMED	Hypoglycaemia unawareness in type 2 diabetes mellitus	1.19831E+14
SNOMED	Macular edema co-occurrent and due to type 1 diabetes mellitus	769219006
SNOMED	Type II diabetes mellitus	5.0231E+13
SNOMED	Education about diabetes and driving	700414001
SNOMED	Recommendation to self-refer for diabetes structured education	9.77211E+14
SNOMED	Congenital lipoatrophic diabetes [Ambiguous]	237609003
SNOMED	Microalbuminuria due to type 2 diabetes mellitus	9.0781E+13
SNOMED	Cystic fibrosis related diabetes mellitus	2.86451E+14
SNOMED	Persistent microalbuminuria associated with type II diabetes mellitus (disorder)	420715001
SNOMED	Seen in community diabetes specialist clinic	1.88691E+14
SNOMED	Type 2 diabetes mellitus with exudative maculopathy	1.04021E+14
SNOMED	Congenital total lipodystrophy (disorder)	284449005
SNOMED	Quality and Outcomes Framework diabetes mellitus quality indicator-related care invitation using preferred method of communication (procedure)	1.43401E+14
SNOMED	Lumbosacral plexopathy co-occurrent and due to diabetes mellitus	724997001
SNOMED	Hyperlipidemia due to type 2 diabetes mellitus	1.37931E+14
SNOMED	Type I diabetes mellitus with gangrene	4.3591E+13
SNOMED	Type II diabetes mellitus with neurological complications	4.3751E+13
SNOMED	Type I diabetes mellitus with polyneuropathy	190375004
SNOMED	Diabetes Distress Scale 17 item	9.09451E+14
SNOMED	Diabetes type 2 review	2.79331E+14
SNOMED	DDS2 - Diabetes Distress Scale 2	9.09401E+14
SNOMED	Moderate nonproliferative retinopathy due to diabetes mellitus	312904009
SNOMED	Type 2 diabetes mellitus with persistent proteinuria	8.3231E+13
SNOMED	Polyneuropathy in diabetes	193182005
SNOMED	Diabetes monitoring short message service text message third invitation (procedure)	1.06693E+15
SNOMED	Did not attend expert patient education versus routine treatment diabetes structured education programme	3.07211E+14
SNOMED	Disorder of macula due to diabetes mellitus	232020009
SNOMED	Unstable diabetes	275918005
SNOMED	Maternally inherited diabetes mellitus (disorder)	3.35621E+14
SNOMED	Diabetic renal disease due to drug induced diabetes mellitus	3.68201E+14
SNOMED	Type 1 diabetes mellitus with persistent microalbuminuria (disorder)	401110002
SNOMED	Type I diabetes mellitus maturity onset	4.3621E+13
SNOMED	Hypertension in chronic kidney disease due to type 1 diabetes mellitus	7.1701E+13
SNOMED	Nonproliferative retinopathy of left eye due to diabetes mellitus	816177009
SNOMED	Diabetes clinic satisfaction questionnaire (assessment scale)	273411009

SNOMED	Diabetes mellitus, juvenile type, with no mention of complication	190322003
SNOMED	Type I diabetes mellitus with gangrene	8.2901E+13
SNOMED	Diabetes mellitus type I diet education	3.00121E+14
SNOMED	Diabetes mellitus, adult onset, with no mention of complication	267380002
SNOMED	Under care of diabetes specialist nurse	1.02451E+14
SNOMED	Acanthosis nigricans due to type 2 diabetes mellitus	9859006
SNOMED	Ketoacidosis due to malnutrition related diabetes mellitus	190406000
SNOMED	Diabetes + nephropathy	267470000
SNOMED	Severe nonproliferative retinopathy with clinically significant macular oedema due to diabetes mellitus	399872003
SNOMED	Diabetes mellitus -adult onset	267468009
SNOMED	Diabetes mellitus: [adult onset] or [noninsulin dependent]	154672006
SNOMED	Diabetes mellitus without complication (disorder)	111552007
SNOMED	Other specified diabetes mellitus with neurological complications	6.19991E+14
SNOMED	Diabetes mellitus with renal manifestation	267381003
SNOMED	Proliferative retinopathy of bilateral eyes due to diabetes mellitus type 1	1.67498E+16
SNOMED	Cervical radiculoplexus neuropathy with diabetes mellitus	770094004
SNOMED	History of diabetes mellitus type 1	7.72031E+14
SNOMED	Newly diagnosed diabetes mellitus type 1	870528001
SNOMED	Referral to dose adjustment for normal eating diabetes structured education programme	3.06701E+14
SNOMED	Type II diabetes mellitus with gangrene	8.3101E+13
SNOMED	Diabetes monitored (& check done) (regime/therapy)	153941001
SNOMED	Lipoatrophic diabetes	1.12761E+14
SNOMED	Hyperosmolar coma due to secondary diabetes mellitus	3.68601E+14
SNOMED	Proteinuria due to type 1 diabetes mellitus	2.43421E+14
SNOMED	[X]Pre-existing diabetes mellitus, unspecified	4.14601E+14
SNOMED	Thick skin syndrome due to diabetes mellitus	238983004
SNOMED	Diabetes monitoring injection site not checked	8.19331E+14
SNOMED	Neovascular glaucoma due to diabetes mellitus	713457002
SNOMED	Diabetes type 2 review	2.79321E+14
SNOMED	Diabetes mellitus, juvenile type, with no mention of complication	5.32401E+14
SNOMED	Proliferative retinopathy of bilateral eyes due to diabetes mellitus type 2	1.67481E+16
SNOMED	Seen by diabetes special interest general practitioner	3.00651E+14
SNOMED	Type I diabetes mellitus with polyneuropathy	4.3651E+13
SNOMED	Type II diabetes mellitus in remission (disorder)	703138006
SNOMED	Diabetes with other complications	6.59291E+14
SNOMED	Other specified diabetes mellitus with ophthalmic complications	5.93701E+14
SNOMED	Diabetes mellitus with neurological manifestation: (& [amyotrophy] or [neuropathy] or [polyneuropathy])	190349003
SNOMED	Moderate nonproliferative retinopathy due to type 2 diabetes mellitus	1.38921E+14
SNOMED	Quality and Outcomes Framework diabetes mellitus quality indicator-related care invitation (procedure)	1.11092E+15
SNOMED	Diabetes monitoring default (regime/therapy)	153934007
SNOMED	Diabetes care by hospital only	134389002
SNOMED	Diabetes with other complications	154689003
SNOMED	Type II diabetes mellitus with polyneuropathy	190397002
SNOMED	Nonproliferative retinopathy of left eye due to diabetes mellitus type 1	1.67452E+16
SNOMED	Acrorenal field defect, ectodermal dysplasia, and lipoatrophic diabetes	237610008
SNOMED	Diabetic autonomic neuropathy associated with type 1 diabetes mellitus	425442003
SNOMED	Disorder of eye co-occurrent and due to diabetes mellitus	25093002
SNOMED	Referral to community diabetes service	8.11391E+14
SNOMED	Secondary endocrine diabetes mellitus (disorder)	237601000
SNOMED	Diabetes self management plan (qualifier value)	698360004
SNOMED	Referral to children's diabetes nurse specialist	3.44061E+14
SNOMED	Proteinuric nephropathy due to diabetes mellitus	236500003

SNOMED	Diabetes mellitus with persistent microalbuminuria	7.8421E+13
SNOMED	Expert patient education versus routine treatment diabetes structured education programme completed	3.06551E+14
SNOMED	Non-high-risk proliferative retinopathy due to diabetes mellitus	312906006
SNOMED	Proliferative retinopathy with neovascularization elsewhere than the optic disc due to diabetes mellitus	232022001
SNOMED	Provision of written information about diabetes and high cholesterol	9.62831E+14
SNOMED	Referral to dose adjustment for normal eating diabetes structured education programme declined	8.60991E+14
SNOMED	Type I diabetes mellitus with arthropathy	5.0221E+13
SNOMED	Diabetes quality of life questionnaire	8.38221E+14
SNOMED	Diabetes mellitus	154671004
SNOMED	Diabetes mellitus NOS with ophthalmic manifestation	190348006
SNOMED	Peripheral vascular disease due to type I diabetes	3.1211E+13
SNOMED	Traction retinal detachment due to type 1 diabetes mellitus	8.2571E+13
SNOMED	Discharged from diabetes shared care programme	7.07351E+14
SNOMED	Coronary artery disease due to type 1 diabetes mellitus	1.68912E+16
SNOMED	Proliferative retinopathy following surgery due to diabetes mellitus	1.03981E+14
SNOMED	Latent autoimmune diabetes mellitus in adult	3.35631E+14
SNOMED	Amyotrophy due to type 1 diabetes mellitus	427571000
SNOMED	Diabetic on oral treatment	170746002
SNOMED	Hyperosmolar coma associated with diabetes mellitus	422126006
SNOMED	Diabetic neuropathic arthropathy (disorder)	201724008
SNOMED	Diabetes type 1 review (regime/therapy)	2.79291E+14
SNOMED	Diabetes mellitus type 1 (disorder)	46635009
SNOMED	Type II diabetes mellitus with renal complications	8.3051E+13
SNOMED	Insulin treated type 2 diabetes mellitus (disorder)	237599002
SNOMED	Type II diabetes mellitus risk assessment telephone invitation	1.0655E+15
SNOMED	Type I diabetes mellitus uncontrolled (finding)	444073006
SNOMED	Diabetes mellitus type II diet education	3.00131E+14
SNOMED	Diabetes monitoring lower risk albumin excretion	1.99481E+14
SNOMED	[X]Pre-existing diabetes mellitus, unspecified	199233008
SNOMED	Pretibial pigmental patches in diabetes	62260007
SNOMED	Moderate nonproliferative retinopathy of right eye due to diabetes mellitus type 2	1.67463E+16
SNOMED	Chronic kidney disease stage 5 due to type 1 diabetes mellitus	9.0761E+13
SNOMED	Mixed maculopathy co-occurrent and due to diabetes mellitus	314015001
SNOMED	Remote diabetes structured education and support programme commenced	1.09713E+15
SNOMED	Chronic kidney disease due to type I diabetes mellitus	9.6441E+13
SNOMED	Diabetes mellitus due to genetic defect in insulin action	609569007
SNOMED	Ketoacidosis without coma due to diabetes mellitus	111556005
SNOMED	Referral to community diabetes clinic	8.41221E+14
SNOMED	Advised about diabetes and driving	7.58621E+14
SNOMED	Iritis co-occurrent and due to diabetes mellitus	193489006
SNOMED	Lipoatrophic diabetes, NOS	71325002
SNOMED	Preproliferative retinopathy co-occurrent and due to diabetes mellitus	193349004
SNOMED	Renal papillary necrosis due to diabetes mellitus	723074006
SNOMED	Diabetes mellitus with persistent microalbuminuria	401087005
SNOMED	Maternally inherited diabetes mellitus	3.38041E+14
SNOMED	Nonproliferative retinopathy of right eye due to diabetes mellitus type 1	1.67467E+16
SNOMED	Macular oedema not clinically significant co-occurrent and due to diabetes mellitus	399864000
SNOMED	Diabetes resolved	1.71771E+14
SNOMED	Transition of diabetes care options discussed	415744000
SNOMED	Malnutrition-related diabetes mellitus	190404002
SNOMED	Type 1 diabetes mellitus with complication	371054002
SNOMED	Severe nonproliferative retinopathy of left eye co-occurrent and due to diabetes mellitus	769188001

SNOMED	Completed diabetes structured education programme	3.00521E+14
SNOMED	[X]Malnutrition-related diabetes mellitus with other specified complications	191046008
SNOMED	Renal cysts and diabetes syndrome	7.29981E+14
SNOMED	Attended DESMOND (diabetes education and self management for ongoing and newly diagnosed) structured programme	2.76651E+14
SNOMED	Diabetes + nephropathy (& [Kimmelstiel-Wilson syndrome])	154677000
SNOMED	Diabetes monitoring administration NOS (administrative concept)	8.741E+12
SNOMED	Other specified diabetes mellitus with unspecified complications	190425002
SNOMED	Diabetes treatment satisfaction questionnaire (assessment scale)	165206008
SNOMED	Pre-existing diabetes mellitus	3.85051E+14
SNOMED	At risk of ulcer of right foot due to diabetes mellitus	863883002
SNOMED	Maturity onset diabetes mellitus in young	7.8471E+13
SNOMED	Lesion of skin due to diabetes mellitus	724876003
SNOMED	Dose adjustment for normal eating structured diabetes education programme completed	3.06491E+14
SNOMED	Diabetes mellitus, juvenile type, with other specified manifestation	190418009
SNOMED	Diabetes mellitus induced by non-steroid drugs	9.9181E+13
SNOMED	Type I diabetes mellitus with gastroparesis	425159004
SNOMED	Hyperosmolarity due to type 1 diabetes mellitus (disorder)	3.68561E+14
SNOMED	Diabetes type 1 review	2.79311E+14
SNOMED	Self management of diabetes	8.06961E+14
SNOMED	Malnutrition-related diabetes mellitus without complications	190412005
SNOMED	Diabetes monitoring invitation by short message service	7.52151E+14
SNOMED	Rare form of secondary diabetes mellitus, due to disorder other than malnutrition, protein deficiency, pancreatic disease, hormonal disease, drugs, receptor abnormality, OR genetic syndrome	111554008
SNOMED	Iritis of left eye due to diabetes mellitus	768798006
SNOMED	Nonproliferative retinopathy of bilateral eyes due to diabetes mellitus type 2	1.67477E+16
SNOMED	Diabetes mellitus education service	734920002
SNOMED	Referral to expert patient education versus routine treatment diabetes structured education programme	3.06791E+14
SNOMED	Mild nonproliferative retinopathy of right eye due to diabetes mellitus type 1	1.67456E+16
SNOMED	Exudative maculopathy co-occurrent and due to type 1 diabetes mellitus (disorder)	420486006
SNOMED	Acidosis due to type 2 diabetes mellitus	721284006
SNOMED	Attending diabetes clinic	312888008
SNOMED	Other specified diabetes mellitus with multiple complications	190382000
SNOMED	Non-insulin-dependent diabetes mellitus with renal complications	190385003
SNOMED	Diabetes mellitus syndrome in newborn infant	49817004
SNOMED	Diabetes mellitus, adult onset, with hyperosmolar coma	190331003
SNOMED	Lipodystrophy, partial, with Reiger anomaly, short stature, and insulinopenic diabetes mellitus	237608006
SNOMED	Type II diabetes mellitus with mononeuropathy	190396006
SNOMED	Diabetes mellitus due to pancreatic injury	1.05401E+14
SNOMED	Pre-existing diabetes mellitus in pregnancy	609563008
SNOMED	Health education - diabetes	385805005
SNOMED	Diabetes with coma	111557001
SNOMED	Autonomic neuropathy with type II diabetes mellitus	712883005
SNOMED	Type II diabetes mellitus with multiple complications (disorder)	190388001
SNOMED	Mixed hyperlipidaemia due to type 2 diabetes mellitus	7.01E+11
SNOMED	Dose adjustment for normal eating structured diabetes education programme completed	3.06481E+14
SNOMED	Referral to DESMOND (diabetes education and self management for ongoing and newly diagnosed) diabetes structured education programme declined	7.81161E+14
SNOMED	Ketoacidosis in type II diabetes mellitus (disorder)	421750000
SNOMED	Type II diabetes mellitus with arthropathy	190402003
SNOMED	Proliferative retinopathy of left eye due to diabetes mellitus type 2	1.67479E+16
SNOMED	NIDDM in nonobese	359638003
SNOMED	Other specified diabetes mellitus with peripheral circulatory complications	5.93721E+14
SNOMED	Pan retinal photocoagulation for diabetes	1.21461E+14

SNOMED	Type I diabetes mellitus with diabetic cataract	190378002
SNOMED	Referral to community diabetes specialist nurse	2.79541E+14
SNOMED	Brittle type I diabetes mellitus (finding)	290002008
SNOMED	Glaucoma due to diabetes mellitus	860798008
SNOMED	Very severe nonproliferative retinopathy with clinically significant macular oedema due to diabetes mellitus	399877009
SNOMED	Disorder of right macula co-occurrent and due to diabetes mellitus	769244003
SNOMED	Type II diabetes mellitus with neuropathic arthropathy	4.3901E+13
SNOMED	Type I diabetes mellitus in remission	8.88191E+14
SNOMED	IDDM - Insulin-dependent diabetes mellitus with retinopathy	190370009
SNOMED	Non-proliferative retinopathy due to secondary diabetes mellitus	3.68721E+14
SNOMED	Diabetes mellitus with hyperosmolar coma	190329007
SNOMED	Provision of diabetes identity card	9.90851E+14
SNOMED	Chronic kidney disease stage 2 due to drug induced diabetes mellitus	3.68431E+14
SNOMED	Type 2 diabetes mellitus with ketoacidotic coma	8.3261E+13
SNOMED	Chronic kidney disease stage 1 due to drug induced diabetes mellitus	3.68421E+14
SNOMED	Patient on maximal tolerated therapy for diabetes (finding)	407569005
SNOMED	Type 2 diabetes mellitus with ophthalmic complications	190386002
SNOMED	Gangrene associated with type II diabetes mellitus (disorder)	421631007
SNOMED	Peripheral neuropathy co-occurrent and due to type 1 diabetes mellitus	7.1791E+13
SNOMED	Type 1 diabetes mellitus with ketoacidotic coma	8.3041E+13
SNOMED	IDDM with peripheral circulatory disorder	190358005
SNOMED	Diabetes mellitus, adult with gangrene	190357000
SNOMED	Type II diabetes mellitus with nephropathy	8.3161E+13
SNOMED	Diabetes mellitus, juvenile type, with peripheral circulatory disorder	190355008
SNOMED	Type I diabetes mellitus with nephropathy	8.2961E+13
SNOMED	Type II diabetes mellitus without complication	4.3821E+13
SNOMED	Type II diabetes mellitus with ulcer (disorder)	190389009
SNOMED	Diabetes mellitus, adult onset, with neurological manifestation	190351004
SNOMED	Diabetes mellitus with ketoacidosis	420422005
SNOMED	Nephrotic syndrome due to diabetes mellitus	197605007
SNOMED	Diabetes care plan agreed	1.88101E+14
SNOMED	Diabetes monitoring call	308505000
SNOMED	Nodular glomerulosclerosis of kidney due to diabetes mellitus	63510008
SNOMED	Diabetes mellitus, juvenile type, with ophthalmic manifestation	190345009
SNOMED	Neuropathic ulcer of midfoot AND/OR heel due to type 2 diabetes mellitus	1.08781E+14
SNOMED	Type II diabetes mellitus with hypoglycaemic coma	8.3171E+13
SNOMED	Malnutrition-related diabetes mellitus with renal complications	190407009
SNOMED	Foot abnormality - diabetes-related	309597007
SNOMED	Severe nonproliferative retinopathy of right eye due to diabetes mellitus	769187006
SNOMED	Diabetes with ketoacidosis - no coma	286912007
SNOMED	Diabetes monitored	270445003
SNOMED	Chronic kidney disease stage 3 due to type 1 diabetes mellitus	9.0741E+13
SNOMED	High risk proliferative retinopathy without macular oedema due to diabetes mellitus	399862001
SNOMED	Diabetes mellitus, juvenile type, with ketoacidotic coma	190334006
SNOMED	Diabetic peripheral angiopathy (disorder)	127014009
SNOMED	Diabetes mellitus NOS with hyperosmolar coma	190332005
SNOMED	Type 1 diabetes mellitus with gastroparesis	2.13991E+14
SNOMED	Referral to community diabetes antenatal clinic	770789008
SNOMED	Retinal ischemia with type 1 diabetes mellitus	1.04941E+14
SNOMED	Chronic kidney disease stage 2 due to type 2 diabetes mellitus	7.41E+11
SNOMED	Neurological disorder co-occurrent and due to type 2 diabetes mellitus (disorder)	421326000
SNOMED	Type 1 diabetes mellitus with polyneuropathy	314369009

SNOMED	Type II diabetes mellitus with ulcer	8.3091E+13
SNOMED	KPD - ketosis-prone diabetes mellitus	1.10235E+15
SNOMED	Diabetes mellitus, adult onset, with unspecified complication	190424003
SNOMED	[X]Malnutrition-related diabetes mellitus with unspecified complications	4.66171E+14
SNOMED	Diabetes mellitus with no mention of complication	190321005
SNOMED	Non-ketotic non-hyperosmolar coma due to diabetes mellitus	421966007
SNOMED	Clinically significant macular oedema of left eye co-occurrent and due to diabetes mellitus	769222008
SNOMED	Skin ulcer associated with type II diabetes mellitus	1.541E+12
SNOMED	Referral to diabetes nurse	183615002
SNOMED	Insulin reactive hypoglycaemia in type 2 diabetes mellitus	8.4361E+13
SNOMED	Dietary education for type 1 diabetes mellitus	429094000
SNOMED	Type II diabetes mellitus with mononeuropathy	8.3141E+13
SNOMED	Provision of diabetes identity card	9.66161E+14
SNOMED	Diabetes mellitus caused by chemical (disorder)	737212004
SNOMED	Diabetes mellitus associated with pancreatic disease (disorder)	51002006
SNOMED	Diabetes mellitus, juvenile type, with ketoacidosis	190325001
SNOMED	Diabetes mellitus NOS with ophthalmic manifestation	5.93711E+14
SNOMED	Bullous disease due to diabetes mellitus	48951005
SNOMED	H/O: Admission in last year for diabetes foot problem	161564001
SNOMED	Unstable type I diabetes mellitus	4.3571E+13
SNOMED	Microalbuminuria due to type 1 diabetes mellitus	1.8521E+13
SNOMED	Diabetes mellitus AND insipidus with optic atrophy AND deafness (disorder)	70694009
SNOMED	Diabetes self management plan (record artifact)	735985000
SNOMED	Referral to diabetes special interest general practitioner	3.34721E+14
SNOMED	Diabetes with gangrene	190354007
SNOMED	Diabetes mellitus type 2 (disorder)	44054006
SNOMED	Type II diabetes on insulin	2.4471E+13
SNOMED	Diabetes monitoring invitation by short message service text messaging (procedure)	705072004
SNOMED	Diabetes monitoring status	308506004
SNOMED	Diabetes monitoring third letter (regime/therapy)	153937000
SNOMED	H/O: diabetes mellitus	161445009
SNOMED	Diabetic - cooperative patient	170769004
SNOMED	Assessment using DDS2 (Diabetes Distress Scale 2)	9.09431E+14
SNOMED	Mild nonproliferative retinopathy of left eye due to diabetes mellitus type 2	1.67455E+16
SNOMED	Non-high-risk proliferative retinopathy with clinically significant macular edema due to diabetes mellitus	399875001
SNOMED	Myopathy and diabetes mellitus	783722008
SNOMED	[X]Unspecified diabetes mellitus with renal complications	191048009
SNOMED	Asymmetric proximal motor neuropathy with diabetes mellitus	79554005
SNOMED	Very severe proliferative retinopathy due to diabetes mellitus	399865004
SNOMED	Macular oedema and retinopathy due to type 2 diabetes mellitus	9.7331E+13
SNOMED	Absence of lower extremity due to diabetes mellitus	735200002
SNOMED	Type I diabetes mellitus - poor control	5.0211E+13
SNOMED	Referral to diabetes service	9.67261E+14
SNOMED	Patient offered diabetes structured education programme	1.91271E+14
SNOMED	Disorder of nervous system due to malnutrition related diabetes mellitus	420683009
SNOMED	Diabetes mellitus type 2 in obese	81531005
SNOMED	Metabolic acidosis co-occurrent and due to diabetes mellitus (disorder)	735539005
SNOMED	Proliferative retinopathy of right eye due to diabetes mellitus type 2	1.67497E+16
SNOMED	Ankle ulcer due to type 1 diabetes mellitus	8.7471E+13
SNOMED	Lipoatrophic diabetes	9.9281E+13
SNOMED	Referral to diabetes education and self management for ongoing and newly diagnosed diabetes structured programme	3.06751E+14
SNOMED	Registration for access to online diabetes self-management application	1.43911E+14

SNOMED	Lipoatrophic diabetes	408543001
SNOMED	Foot abnormality - diabetes-related	164483006
SNOMED	ADM - atypical diabetes mellitus	5.30559E+17
SNOMED	Foot abnormality - diabetes-related	141680008
SNOMED	Blindness co-occurrent and due to type 1 diabetes mellitus	6.0991E+13
SNOMED	Did not complete diabetes education and self management for ongoing and newly diagnosed structured programme	3.07011E+14
SNOMED	Very severe nonproliferative retinopathy co-occurrent and due to diabetes mellitus	399876000
SNOMED	Neurologic disorder associated with diabetes mellitus	422088007
SNOMED	Type II diabetes mellitus with arthropathy (disorder)	314903002
SNOMED	Moderate nonproliferative retinopathy of bilateral eyes due to diabetes mellitus type 2	1.67463E+16
SNOMED	Type I diabetes mellitus with gastroparesis	1.98661E+14
SNOMED	Diabetes monitoring administration	7.13671E+14
SNOMED	Erectile dysfunction with type 2 diabetes mellitus	428007007
SNOMED	Diabetes mellitus NOS with neurological manifestation	190353001
SNOMED	Disorder of nerve co-occurrent and due to type 1 diabetes mellitus	3.68521E+14
SNOMED	Type II diabetes mellitus without complication	190395005
SNOMED	Did not complete diabetes structured education programme	3.06941E+14
SNOMED	Severe malnutrition due to type 2 diabetes mellitus	7.2051E+13
SNOMED	Diabetes administration: [monitoring] or [clinic]	3.2901E+13
SNOMED	Referral to diabetes structured education programme	415270003
SNOMED	Lifestyle education for diabetes	9.23461E+14
SNOMED	Moderate nonproliferative retinopathy of bilateral eyes due to diabetes mellitus type 1	1.66975E+16
SNOMED	Peripheral neuropathy co-occurrent and due to diabetes mellitus	424736006
SNOMED	Maturity-onset diabetes of the young, type 5 (disorder)	609572000
SNOMED	Ketoacidotic coma due to diabetes mellitus	26298008
SNOMED	Diabetes mellitus NOS with ketoacidotic coma	5.51831E+14
SNOMED	Macular oedema of left eye co-occurrent and due to diabetes mellitus	769218003
SNOMED	Hyperglycemia due to type 2 diabetes mellitus	3.68051E+14
SNOMED	Hypoglycemic coma co-occurrent and due to diabetes mellitus type II (disorder)	719216001
SNOMED	Completed diabetes structured education programme	3.00511E+14
SNOMED	Diabetes Year of Care annual review	8.87871E+14
SNOMED	Under care of diabetes specialist nurse	408392008
SNOMED	Retinopathy co-occurrent and due to type 2 diabetes mellitus (disorder)	422034002
SNOMED	Lipoatrophic diabetes (disorder)	127012008
SNOMED	Hyperosmolar non-ketotic state in type 2 diabetes mellitus	8.3221E+13
SNOMED	Cystic fibrosis related diabetes mellitus	2.92571E+14
SNOMED	Maturity-onset diabetes of the young, type 9	609576002
SNOMED	Dermatitis due to type 2 diabetes mellitus	1.59365E+16
SNOMED	Chronic kidney disease stage 1 due to type 1 diabetes mellitus	9.0721E+13
SNOMED	Diabetes monitoring injection site check declined	8.19301E+14
SNOMED	Maturity onset diabetes of the young, type 2 (disorder)	237604008
SNOMED	Type I diabetes mellitus with ulcer (disorder)	190368000
SNOMED	Ketoacidotic coma in type I diabetes mellitus (disorder)	421075007
SNOMED	Type II diabetes mellitus with polyneuropathy	4.3841E+13
SNOMED	Drug-induced diabetes mellitus (disorder)	5368009
SNOMED	Non-insulin-dependent diabetes mellitus - poor control	190392008
SNOMED	Lifestyle education regarding diabetes	9.23471E+14
SNOMED	Type I diabetes mellitus with multiple complications	4.3561E+13
SNOMED	Diabetes with ketoacidosis - no coma	154675008
SNOMED	Diabetes mellitus with no mention of complication	154674007
SNOMED	Insulin-dependent diabetes mellitus secretory diarrhea syndrome (disorder)	237618001
SNOMED	Glucose tolerance test indicates diabetes mellitus	166928007

SNOMED	Diabetes mellitus due to cystic fibrosis	427089005
SNOMED	Type II diabetes mellitus with hypoglycemic coma	190399004
SNOMED	Diabetes monitoring admin.NOS (regime/therapy)	153942008
SNOMED	Mononeuropathy simplex co-occurrent and due to diabetes mellitus	81830002
SNOMED	Diabetes with gangrene	275522008
SNOMED	Malnutrition-related diabetes mellitus with coma	190405001
SNOMED	Diabetes monitoring verbal invite	153938005
SNOMED	Type I diabetes mellitus with diabetic cataract	8.2981E+13
SNOMED	Diabetes medication review	394725008
SNOMED	Diabetes mellitus due to abnormal insulin	91352004
SNOMED	Very severe nonproliferative retinopathy of right eye due to diabetes mellitus	769190000
SNOMED	Diabetes structured education programme declined	3.06611E+14
SNOMED	Attends diabetes monitoring (regime/therapy)	153932006
SNOMED	Asymptomatic neuropathy co-occurrent and due to diabetes mellitus	193185007
SNOMED	Attending diabetes clinic	153683009
SNOMED	XPERT diabetes structured education programme completed	3.06531E+14
SNOMED	Assessment using Diabetes Distress Scale 17 item	9.10911E+14
SNOMED	[V]Dietary counselling in diabetes mellitus	316426000
SNOMED	Unstable type I diabetes mellitus	190367005
SNOMED	No maculopathy of right eye due to diabetes mellitus	860710002
SNOMED	Acute complication due to diabetes mellitus	762489000
SNOMED	Glomerulonephritis due to diabetes mellitus	425455002
SNOMED	Latent autoimmune diabetes mellitus in adult	3.38031E+14
SNOMED	Diabetes monitoring deleted	153940000
SNOMED	Type 2 diabetes mellitus with persistent microalbuminuria	8.3241E+13
SNOMED	Angina associated with type 2 diabetes mellitus	7.91E+11
SNOMED	Provision of written information about diabetes and high cholesterol	9.62841E+14
SNOMED	Diabetes Distress Scale 2 item	9.09411E+14
SNOMED	Type II diabetes mellitus well controlled	444110003
SNOMED	Type I diabetes mellitus without complication	4.3631E+13
SNOMED	Diabetes mellitus with ketoacidosis	24927004
SNOMED	Type I diabetes mellitus	5.0201E+13
SNOMED	Type II diabetes mellitus in remission	8.88221E+14
SNOMED	Polyneuropathy due to drug induced diabetes mellitus	3.68251E+14
SNOMED	Diabetes mellitus NOS with unspecified complication	6.58111E+14
SNOMED	Diabetes mellitus with no mention of complication	5.28451E+14
SNOMED	Retinal abnormality - diabetes-related	141196007
SNOMED	Conversion to insulin by diabetes specialist nurse	8.76391E+14
SNOMED	Malnutrition-related diabetes mellitus with neurological complications	190409007
SNOMED	Type 1 diabetes mellitus with diabetic cataract	314887002
SNOMED	Type II diabetes mellitus - poor control	8.4041E+13
SNOMED	Type 1 diabetes mellitus with exudative maculopathy	1.04011E+14
SNOMED	Retinopathy co-occurrent and due to type 1 diabetes mellitus (disorder)	420789003
SNOMED	Hypertension in chronic kidney disease stage 3 due to type 2 diabetes mellitus	1.40121E+14
SNOMED	Mixed hyperlipidemia associated with type 1 diabetes mellitus	1.571E+12
SNOMED	Hypertension in chronic kidney disease stage 5 due to type 2 diabetes mellitus	1.40101E+14
SNOMED	Cataract due to drug induced diabetes mellitus	3.68151E+14
SNOMED	Diabetes mellitus, adult onset, with ketoacidosis	190326000
SNOMED	Diabetes medication review	394035007
SNOMED	Mild nonproliferative retinopathy co-occurrent and due to type 1 diabetes mellitus	1.38881E+14
SNOMED	Diabetes care plan declined	7.87061E+14
SNOMED	Referral to hospital diabetes antenatal clinic	1.05424E+15

SNOMED	Hyperlipidemia due to type 1 diabetes mellitus	1.37941E+14
SNOMED	Diabetes monitoring invitation (procedure)	310425007
SNOMED	Under care of hospital-based diabetes specialist nurse	1.02455E+15
SNOMED	Diabetes mellitus NOS with peripheral circulatory disorder	190361006
SNOMED	Foot ulcer due to type 1 diabetes mellitus	1.64881E+14
SNOMED	Rubeosis iridis co-occurrent and due to type 1 diabetes mellitus (disorder)	8.2581E+13
SNOMED	Hypertension concurrent and due to end stage renal disease on dialysis due to type 2 diabetes mellitus	1.27991E+14
SNOMED	Type II diabetes mellitus with neuropathic arthropathy	190403008
SNOMED	Heel AND/OR midfoot ulcer due to type 1 diabetes mellitus	8.7481E+13
SNOMED	Retinal abnormality - diabetes-related	163997001
SNOMED	Diabetes with other coma	33248009
SNOMED	Diabetes education care plan	9.61931E+14
SNOMED	Maturity-onset diabetes of the young, type 4	609571007
SNOMED	Attended DESMOND diabetes structured education programme	2.76671E+14
SNOMED	Disorder of macula of bilateral eyes due to diabetes mellitus	860721006
SNOMED	Secondary diabetes mellitus without complication	1.22521E+14
SNOMED	Child diabetes diet	79367009
SNOMED	Chronic kidney disease stage 4 due to type I diabetes mellitus	9.0751E+13
SNOMED	Malnutrition-related diabetes mellitus	9.9151E+13
SNOMED	Diabetes mellitus induced by non-steroid drugs without complication	1.22431E+14
SNOMED	Type II diabetes mellitus with nephropathy	4.3851E+13
SNOMED	Secondary pancreatic diabetes mellitus without complication	1.22411E+14
SNOMED	Nonproliferative retinopathy of left eye due to diabetes mellitus type 2	1.67451E+16
SNOMED	Glomerulopathy due to diabetes mellitus	309426007
SNOMED	Pseudo-phlorizin diabetes	61598006
SNOMED	Mild nonproliferative retinopathy of right eye due to diabetes mellitus type 2	1.67457E+16
SNOMED	Type I diabetes mellitus without complication	8.2931E+13
SNOMED	Macular edema of right eye co-occurrent and due to diabetes mellitus	769217008
SNOMED	Polyneuropathy co-occurrent and due to diabetes mellitus (disorder)	49455004
SNOMED	Diabetes mellitus, adult onset, with other specified manifestation	190419001
SNOMED	Has diabetes identity card	720724003
SNOMED	Type II diabetes mellitus risk assessment invitation	1.06545E+15
SNOMED	Did not attend diabetes structured education programme	3.06861E+14
SNOMED	Seen in community diabetes antenatal clinic	1.05421E+15
SNOMED	Other specified diabetes mellitus with renal complications	190341000
SNOMED	Type I diabetes mellitus with ulcer	8.2891E+13
SNOMED	Cataract co-occurrent and due to diabetes mellitus type 1 (disorder)	421920002
SNOMED	Dyslipidemia with high density lipoprotein below reference range and triglyceride above reference range due to type 2 diabetes mellitus	1.11231E+14
SNOMED	Chronic kidney disease stage 1 associated with type 2 diabetes mellitus	7.51E+11
SNOMED	Type I diabetes mellitus without complication	190373006
SNOMED	Referral to online diabetes structured education programme	1.10873E+15
SNOMED	Congenital lipoatrophic diabetes	285369007
SNOMED	Abnormal metabolic state in diabetes mellitus	237620003
SNOMED	Diabetes + neuropathy	267472008
SNOMED	[X]Malnutrition-related diabetes mellitus with unspecified complications	191047004
SNOMED	Malnutrition-related diabetes mellitus - fibrocalculous	237600004
SNOMED	Type I diabetes mellitus with peripheral angiopathy	8.2991E+13
SNOMED	Type I diabetes mellitus with neuropathic arthropathy	314894004
SNOMED	Diabetes mellitus, juvenile type, with other specified manifestation	6.58021E+14
SNOMED	Cataract of right eye co-occurrent and due to diabetes mellitus (disorder)	768792007
SNOMED	Gastroparesis co-occurrent and due to type 1 diabetes mellitus (disorder)	713702000
SNOMED	Type 2 diabetes on diet only	2.4481E+13

SNOMED	Suspected glaucoma due to type 2 diabetes mellitus	1.08791E+14
SNOMED	Diabetes clinic	702706001
SNOMED	Discharged from care of diabetes specialist nurse	1.88281E+14
SNOMED	Seen in diabetes preconception counselling clinic	1.0542E+15
SNOMED	[X]Malnutrition-related diabetes mellitus with other specified complications	4.78671E+14
SNOMED	Hypoglycaemic event due to diabetes	237632004
SNOMED	Type II diabetes mellitus with diabetic cataract	314888007
SNOMED	Chronic kidney disease stage 4 associated with type 2 diabetes mellitus	7.21E+11
SNOMED	ED-DMT1 - Eating disorder-diabetes mellitus type 1	1.0672E+15
SNOMED	DESMOND diabetes structured education programme completed	3.06501E+14
SNOMED	Hyperosmolar hyperglycemic coma due to diabetes mellitus without ketoacidosis (disorder)	735537007
SNOMED	Diabetes monitoring short message service text message first invitation (procedure)	1.06691E+15
SNOMED	Bronzed diabetes	399144008
SNOMED	Cataract co-occurrent and due to diabetes mellitus type 2 (disorder)	420756003
SNOMED	Type II diabetes mellitus with ketoacidosis	408660003
SNOMED	[EDTA] Diabetes Type II (non-insulin-dependent) associated with renal failure	274590004
SNOMED	Muscular atrophy, ataxia, retinitis pigmentosa, and diabetes mellitus	237611007
SNOMED	No maculopathy of left eye due to diabetes mellitus	860709007
SNOMED	Type II diabetes mellitus without complication (disorder)	313436004
SNOMED	Type 1 diabetes mellitus with ketoacidosis	8.3031E+13
SNOMED	Type II diabetes mellitus risk assessment invitation second letter	1.06547E+15
SNOMED	Type II diabetes mellitus risk assessment invitation first letter	1.06546E+15
SNOMED	Ulcer of left foot co-occurrent and due to diabetes mellitus type 2	1.06605E+16
SNOMED	Type II diabetes mellitus with ophthalmic complications	8.3061E+13
SNOMED	Coronary artery disease due to type 2 diabetes mellitus	1.68912E+16
SNOMED	Maturity-onset diabetes of the young, type 11	609578001
SNOMED	Neuropathic ulcer of toe due to type 2 diabetes mellitus	1.40381E+14
SNOMED	Hyperosmolar nonketotic coma in diabetes	55692006
SNOMED	At moderate risk of ulcer of right foot due to diabetes mellitus	863885009
SNOMED	Patient diabetes education review	1.69561E+14
SNOMED	Disorder of soft tissue co-occurrent and due to diabetes mellitus	238981002
SNOMED	Hyperosmolar coma due to drug induced diabetes mellitus	3.67241E+14
SNOMED	Vitreous haemorrhage due to type 2 diabetes mellitus	1.491E+12
SNOMED	Diabetes Year of Care annual review	8.87861E+14
SNOMED	Assessment using Diabetes Treatment Satisfaction Questionnaire status version	1.08558E+15
SNOMED	X-PERT First Steps diabetes self-management programme completed	1.03328E+15
SNOMED	Multiple complications due to diabetes mellitus	441628001
SNOMED	Diabetes mellitus type 1 without retinopathy	3.1321E+13
SNOMED	Type 2 diabetes mellitus risk assessment declined	772789003
SNOMED	Other specified diabetes mellitus with ophthalmic complications	190347001
SNOMED	Type 2 diabetes mellitus with polyneuropathy	314371009
SNOMED	Type 2 diabetes mellitus with persistent proteinuria	401111003
SNOMED	Sensory neuropathy due to type 1 diabetes mellitus	1.02781E+14
SNOMED	Ulcer of skin co-occurrent and due to type 2 diabetes mellitus	1.02621E+14
SNOMED	Severe nonproliferative retinopathy of right eye due to diabetes mellitus type 1	1.67473E+16
SNOMED	Diabetes structured education programme offered	1.10369E+15
SNOMED	Diabetes mellitus caused by non-steroid drugs (disorder)	408540003
SNOMED	Hyperglycemia due to type 1 diabetes mellitus	3.67991E+14
SNOMED	Did not attend dose adjustment for normal eating diabetes structured education programme	3.07151E+14
SNOMED	Type II diabetes mellitus risk assessment invitation third letter	1.06548E+15
Liver disease		
Read	Secondary biliary cirrhosis	J6161

Read	Viral hepatitis without hepatic coma	A709.
Read	Glycogen synthase deficiency	X40TS
Read	Empyema with hepatopleural fistula	H5002
Read	Chronic yellow atrophy	J61y0
Read	Congenital cystic liver disease	XE1L1
Read	Unspecified viral hepatitis with coma	A706.
Read	Juvenile portal cirrhosis	XE0b8
Read	Hepatitis C genotype 6	XaZ5V
Read	Compensation for liver failure	7L1f.
Read	Hepatitis due to infection	X306Z
Read	Malignant neoplasm of intrahepatic biliary passages	B1512
Read	Biliary cirrhosis (& [primary])	XE0dJ
Read	Compensation for liver failure	XaMuG
Read	Capsular portal cirrhosis	J6156
Read	Local recurrence of malignant tumour of liver	XaFrG
Read	Chronic hepatitis (& [NOS] or [active B] or [active] or [persistent])	XE0dH
Read	Hypertrophic portal cirrhosis	J6155
Read	Hepatitis in other infectious diseases EC	J632.
Read	Alcoholic hepatic failure	X3073
Read	Hepatic glycogen phosphorylase kinase deficiency	Xa6ax
Read	Toxic liver disease with hepatic necrosis	J6351
Read	Cardituberculous cirrhosis	J615E
Read	Viral serum hepatitis B	A703.
Read	Hepatic failure as a complication of care	SP142
Read	Conjugated hyperbilirubinaemia in infancy	Xa0B2
Read	Primary carcinoma of liver	B1500
Read	Rotor syndrome	C3743
Read	Hepatitis C genotype 5	XaZ5U
Read	Angiosarcoma of liver	X78P0
Read	Alcoholic hepatic failure	J6130
Read	Infectious hepatitis	XE0TW
Read	Lysosomal alpha-1,4-glucosidase deficiency - juvenile onset	X40TY
Read	Lysosomal alpha-1,4-glucosidase deficiency	X40TW
Read	Hepatitis in infectious diseases EC NOS	J632z
Read	Multilobular portal cirrhosis	XE0b6
Read	Yellow atrophy of the liver	Xa8Df
Read	Unilobular portal cirrhosis	J6150
Read	Alcoholic hepatitis	J617.
Read	Alcoholic liver disease	X3071
Read	Hepatic failure NOS	X3078
Read	Hypoxia-associated cirrhosis	X307U
Read	Portal cirrhosis	X307R
Read	Chronic viral hepatitis B	A7073
Read	Indian childhood cirrhosis	X307P
Read	Hepatic coma	J622.
Read	Tumour of liver	X3080
Read	Hemochromatosis	X307o
Read	Liver transplant failure and rejection	SP086
Read	Cirrhosis of liver	X307L
Read	Klatskin's tumour	X77nj
Read	Fibrosis of liver	X307W
Read	Hepatocellular jaundice	X307w

Read	Crigler-Najjar syndrome type II	X307i
Read	Crigler-Najjar syndrome type I	X307h
Read	Chronic lobular hepatitis	X307G
Read	(Congenital cystic liver disease) or (congenital hepatic cyst)	PB62.
Read	Viral hepatitis C with coma	A7040
Read	Benign tumour of liver	B7150
Read	Sarcoma of liver	X78Oz
Read	Malignant neoplasm of liver unspecified	B152.
Read	Intrahepatic phlebosclerosis and fibrosis	X307c
Read	Congenital malformation of liver	X306S
Read	Wilson's disease	C3510
Read	[X]Toxic liver disease with other disorders of liver	Jyu70
Read	Hepatic haemangioma	X3070
Read	Budd-Chiari syndrome	XE0Va
Read	Glycogen storage disease	C310.
Read	Central haemorrhagic necrosis of liver	J636.
Read	Gonococcal perihepatitis	X406R
Read	Primary malignant neoplasm of liver	B150.
Read	Chronic hepatitis unspecified	J614y
Read	Alpha 1 antitry phenotype PiZZ	X772S
Read	Ca liver - primary	XE1xp
Read	Glycogenosis: [generalised] or [Pompe's disease] or [type 2]	C3101
Read	Liver transplant failure	X307z
Read	Hepatitis C genotype 3	XaZ5S
Read	Hepatorenal syndrome as a complication of care	SP143
Read	Chronic viral hepatitis B with hepatitis D	X306j
Read	Transection of liver	XA07K
Read	Hepatitis C genotype 6	A70F.
Read	Other specified glycogenosis	C310y
Read	Glucose 6-phosphate transport defect	X40TT
Read	Hepatic sclerosis	X307Z
Read	Cystic fibrosis related cirrhosis	XaXi9
Read	Muscle phosphofructokinase deficiency	X20DL
Read	Transient neonatal hyperbilirubinaemia	Q4361
Read	[X]Toxic liver disease, unspecified	Jyu76
Read	Hepatitis C genotype 1	A70A.
Read	Other specified viral hepatitis with hepatic coma NOS	A704z
Read	Mucopolysaccharidosis type II	C3752
Read	Benign tumour of intrahepatic bile duct	B7151
Read	Chronic alcoholic hepatitis	XaBE3
Read	Fibrocystic liver disease	PB621
Read	Phosphate transport defect	X40TU
Read	Toxic liver disease with chronic lobular hepatitis	J6354
Read	Sclerosing cholangitis unspecified	J6619
Read	Carcinoma in situ of liver	B8080
Read	Neonatal unconjugated hyperbilirubinaemia	Xa1op
Read	Other specified liver disorder	J63y.
Read	Unspecified viral hepatitis	A70z.
Read	Fatty portal cirrhosis	J6154
Read	Chronic viral hepatitis B	X306i
Read	Toxic liver disease with chronic persistent hepatitis	J6353
Read	Primary malignant neoplasm of liver NOS	B150z

Read	Hepatitis in yellow fever	J6314
Read	Alcoholic hepatitis	X306r
Read	Subfulminant hepatic failure	X307A
Read	Inflammatory liver disease	X306T
Read	Pipestem portal cirrhosis	J615A
Read	Liver disorder NOS	J63z.
Read	Hepatitis in late syphilis	J6321
Read	Liver transplant rejection	X307y
Read	Toxic hepatitis	J6330
Read	Biliary cirrhosis of children	J6162
Read	Hepatitis in infectious mononucleosis	J6312
Read	Congenital hepatic fibrosis	X307X
Read	Viral hepatitis without hepatic coma	XaYbq
Read	Other hepatitis	Xa3e6
Read	Agenesis of lobe of liver	PB631
Read	Branching-transferase deficiency glycogenosis	C3104
Read	Hepatorenal syndrome	J624.
Read	Generalised glycogenosis	XE11G
Read	Cirrhosis and chronic liver disease	J61..
Read	Cardiac portal cirrhosis	XE0b7
Read	Peliosis hepatis	X306x
Read	Chronic liver disease NOS	J61z.
Read	Cryptogenic cirrhosis	X307O
Read	Other specified liver disorder NOS	J63yz
Read	Hyperbilirubinemia - conjugated - type III	X307n
Read	Lysosomal alpha-1,4-glucosidase deficiency - infantile onset	X40TX
Read	Biliary cirrhosis	J616.
Read	Hepatic fibrosis with hepatic sclerosis	X307b
Read	Portal vein thrombosis	G81..
Read	Hepatitis C genotype 2	A70B.
Read	Viral hepatitis B with coma	A702.
Read	Drug-induced chronic hepatitis	X307I
Read	Abnormal liver lobulation	X784D
Read	Alcoholic fibrosis and sclerosis of liver	X3072
Read	Hepatic granulomas in sarcoidosis	J63A.
Read	Polycystic liver disease	PB620
Read	Liver hamartoma	X784C
Read	Glycogenosis NOS	C310z
Read	Hepatitis: [viral (infectious) A] or [infective]	A701.
Read	Lysosomal alpha-1,4-glucosidase deficiency - adult onset	X40TZ
Read	Hepatitis C carrier	X306o
Read	Chronic hepatitis	J614.
Read	Hepatoblastoma of liver	B1501
Read	Alcoholic cirrhosis of liver	J612.
Read	[X] Hepatic failure	J625.
Read	Alcoholic fatty liver	J610.
Read	Liver failure NOS	X3077
Read	[X]Other congenital malformations of liver	Pyu5E
Read	Fulminant hepatic failure	X3079
Read	Cerebral degeneration in Hunter's disease	F1030
Read	Alpha-1-antitrypsin hepatitis	C3761
Read	Chronic hepatic failure	X307C

Read	Congenital atrophy of left lobe of liver	PB632
Read	Cystic fibrosis related cirrhosis	C3708
Read	Muscle glycogen phosphorylase deficiency	C3100
Read	Chronic viral hepatitis C	X306k
Read	Congenital hepatomegaly	PB6y1
Read	Viral hepatitis C without mention of hepatic coma	A7050
Read	Glucose-6-phosphatase deficiency	C3102
Read	Drug-induced hepatitis	X306s
Read	Liver disease due to cystic fibrosis	C3707
Read	Carcinoma in situ of intrahepatic bile ducts	B8081
Read	Hepatitis C genotype 1	XaZ5P
Read	Toxic liver disease with cholestasis	J6350
Read	Alcoholic liver damage unspecified	J613.
Read	Malignant neoplasm of intrahepatic bile ducts NOS	B151z
Read	Malignant neoplasm of intrahepatic gall duct	B1514
Read	Malignant neoplasm of interlobular biliary canals	B1511
Read	Amylo-1,6-glucosidase deficiency	C3103
Read	[X]Unspecified viral hepatitis with coma	AyuB3
Read	Recurrent hepatitis	J6143
Read	Bacterial portal cirrhosis	J615D
Read	Viral hepatitis with hepatic coma	A708.
Read	Chronic viral hepatitis C	A7072
Read	Autoimmune hepatitis	XaJTk
Read	Mucopolysaccharidosis type II severe form	X40W0
Read	Other specified viral hepatitis without coma	A705.
Read	Cardiac glycogen phosphorylase kinase deficiency	Xa6av
Read	Combined hepatocellular carcinoma and cholangiocarcinoma	XM1FE
Read	Hepatic granulomas in berylliosis	J639.
Read	Portal cirrhosis unspecified	J615y
Read	Biliary cirrhosis NOS	J616z
Read	Hepatic coma	XE0bB
Read	Chronic non-A non-B hepatitis	X306l
Read	[X]Other specified carcinomas of liver	Byu11
Read	Serum hepatitis	XE0TY
Read	Toxic liver disease with fibrosis and cirrhosis of liver	J6356
Read	Viral hepatitis with hepatic coma	XaYbp
Read	Hepatic veno-occlusive disease	J637.
Read	Alpha-1-antitrypsin isoform Z	X80Oc
Read	Syphilitic portal cirrhosis	J615F
Read	Malignant neoplasm of intrahepatic bile ducts	B151.
Read	Hepatic fibrosis	J61y4
Read	Hepatitis C genotype 5	A70E.
Read	Sclerosing cholangitis	X3086
Read	Hepatic failure	X3076
Read	[X]Other specified inflammatory liver diseases	Jyu72
Read	Viral hepatitis	A70..
Read	Liver, biliary, pancreas + gastrointestinal diseases NEC	J6...
Read	Liver transplant disorder	X307x
Read	Hepatic glycogen phosphorylase deficiency	X40Ta
Read	Fibrolamellar hepatocellular carcinoma	X77nk
Read	Neonatal hyperbilirubinaemia	Xa1oo
Read	Peliosis hepatis	J638.

Read	Liver haematoma and contusion with open wound into cavity	S7411
Read	Compensation for liver failure NOS	7L1fz
Read	[X]Chronic viral hepatitis, unspecified	AyuB2
Read	Focal nodular hypoplasia of liver	X784G
Read	Hepatitis in viral diseases EC	J631.
Read	Chronic viral hepatitis B without delta-agent	A7071
Read	Cardiac portal cirrhosis	J6157
Read	Xanthomatous portal cirrhosis	J615C
Read	Mucopolysaccharidosis type II mild form	X40W1
Read	Compensation for liver failure NOS	XaMuI
Read	Liver necrosis	Xa8De
Read	Ca intrahepatic bile ducts	XE1xr
Read	Nodular regenerative hyperplasia of liver	X307K
Read	Alcoholic fibrosis and sclerosis of liver	J6120
Read	Neonatal conjugated hyperbilirubinaemia	Xa1oq
Read	[X]Other sarcomas of the liver	Byu10
Read	Liver disease due to cystic fibrosis	XaREa
Read	Liver, biliary, pancreas + gastrointestinal diseases NOS	J6z..
Read	Hepatocellular carcinoma	B1503
Read	Hepatitis C genotype 4	XaZ5T
Read	Hepatitis unspecified	J633.
Read	Cytomegalovirus hepatitis	A7852
Read	Focal nodular hyperplasia of liver	B7158
Read	Trilobular liver	X784E
Read	Congenital cystic liver disease NOS	PB62z
Read	[X]Unspecified viral hepatitis without coma	AyuB4
Read	Hepatitis C genotype 4	A70D.
Read	Hepatitis C genotype 3	A70C.
Read	Portal hypertension	J623.
Read	Riedel's lobe of liver	PB633
Read	Cardiac cirrhosis	Xa9C7
Read	Liver disorder due to infection	X306V
Read	Toxic liver disease	J635.
Read	Micronodular cirrhosis	X307Q
Read	Dubin-Johnson syndrome	C3741
Read	Diffuse nodular cirrhosis	J6153
Read	Liver injuries without mention of open wound into cavity, NOS	S740z
Read	Zooparasitic portal cirrhosis	J615G
Read	Hepatitis in secondary syphilis	J6322
Read	Hepatic veno-occlusive disease	X306y
Read	Intrahepatic haematoma	XA07E
Read	Liver anomaly, unspecified	PB600
Read	Autoimmune liver disease	X307J
Read	Unconjugated hyperbilirubinaemia	X307f
Read	(Cirrhosis &/or chronic liver dis) or (alcoholic liver disease)	XE0dD
Read	Primary sclerosing cholangitis	J6617
Read	Alcoholic cirrhosis of liver	XE0b4
Read	Other specified compensation for liver failure	7L1fy
Read	[X]Other and unspecified cirrhosis of liver	Jyu71
Read	GLYCOGEN STORAGE DISEASE	ida..
Read	Malignant neoplasm of intrahepatic canaliculi	B1513
Read	Other sequelae of chronic liver disease	XE0bC

Read	Transient familial neonatal hyperbilirubinaemia	Q4333
Read	Hepatitis in cytomegalic inclusion virus	J6311
Read	Vascular disorder of liver	X306u
Read	Neonatal giant cell hepatitis	X307p
Read	Malignant neoplasm of interlobular bile ducts	B1510
Read	Alcoholic liver damage NOS	XE0dF
Read	Other specified viral hepatitis without mention of hepatic coma NOS	A705z
Read	Glycogen phosphorylase kinase deficiency, autosomal recessive	Xa6au
Read	Fitz-Hugh-Curtis syndrome	X406Q
Read	Primary biliary cirrhosis	J6160
Read	Florid cirrhosis	X307N
Read	Portal and splenic vein sclerosis	X307a
Read	[X]Granulomatous hepatitis, not elsewhere classified	Jyu77
Read	Metabolic and genetic disorder affecting the liver	X307d
Read	Hepatitis + adenovirus	J6316
Read	Hepatitis C	A70z0
Read	Focal nodular hyperplasia of liver	Xa0lo
Read	Infectious cirrhosis NOS	J615H
Read	Nonspecific reactive hepatitis	J63y1
Read	Chronic aggressive hepatitis	J6142
Read	Hepatitis unspecified NOS	J633z
Read	Autoimmune hepatitis	J63B.
Read	Chronic passive liver congestion	J630.
Read	Mixed portal cirrhosis	J6152
Read	Multilobular portal cirrhosis	J6151
Read	Hepatitis C genotype 2	XaZ5Q
Read	Toxic cirrhosis	X307V
Read	Toxic portal cirrhosis	J615B
Read	Viral hepatitis A with coma	A700.
Read	Chronic persistent hepatitis	J6140
Read	Pigmentary portal cirrhosis	J6159
Read	Antichymotrypsin deficiency-alpha-1	X307t
Read	Glycogen phosphorylase kinase deficiency, X-linked	Xa6at
Read	Hepatitis D superinfection of hepatitis B carrier	X306d
Read	Pericellular fibrosis of congenital syphilis	X307Y
Read	Glucose transport defect	X40TV
Read	Toxoplasma hepatitis	AD05.
Read	Chronic viral hepatitis	X306h
Read	Secondary malignant neoplasm of liver and intrahepatic bile duct	XaYeq
Read	Hepatic sclerosis	J61y5
Read	Other sequelae of chronic liver disease	J62y.
Read	Chronic alcoholic hepatitis	J6170
Read	Granulomatous hepatitis, not elsewhere classified	J63X.
Read	Hyperbilirubinaemia	X307e
Read	Hepatic fibrosis with hepatic sclerosis	J61y6
Read	[X]Liver disorders in other diseases classified elsewhere	Jyu75
Read	Intrahepatic bile duct carcinoma	Xa97r
Read	Glycogen phosphorylase kinase deficiency	X307r
Read	Ruptured liver NOS	XA07H
Read	Hepatitis in viral diseases EC NOS	J631z
Read	Infectious cirrhosis	X307T
Read	Metastasis to liver	B577.

Read	(Budd-Chiari syndrome) or (hepatic vein thrombosis) or (Stuart-Brass syndrome)	G820.
Read	Chronic lobular hepatitis	J6144
Read	Alpha-1-antitrypsin deficiency	C3762
Read	Conjugated hyperbilirubinaemia	X307k
Read	Toxic liver disease, unspecified	J635X
Read	Cirrhosis: [juvenile portal] or [childhood function] or [Indian childhood]	J6158
Read	Other specified compensation for liver failure	XaMuH
Read	Portal fibrosis without cirrhosis	J61y3
Read	Malignant tumour of liver	Xa97q
Read	Hepatic and muscle glycogen phosphorylase kinase deficiency	Xa6aw
Read	[X]Liver disorders in infectious and parasitic diseases classified elsewhere	Jyu74
Read	Hepatitis B carrier	X306n
Read	Metastasis to liver of unknown primary	Xa3AE
Read	Toxic liver disease with chronic active hepatitis	J6355
Read	[X]Other chronic viral hepatitis	AyuB1
Read	Pulm emphysema, alpha 1 PI def	X101o
Read	Cirrhosis of liver NOS	X307M
Read	Other congenital anomaly of liver	XE1L2
Read	[X]Other specified diseases of liver	Jyu73
Read	Chronic viral hepatitis	A707.
Read	Chronic active hepatitis	J6141
Read	Chronic hepatitis NOS	J614z
Read	Other specified viral hepatitis with coma	A704.
SNOMED	Secondary malignant neoplasm of liver	94381002
SNOMED	Malignant neoplasm of liver	93870000
SNOMED	Lucey-Driscoll syndrome	47444008
SNOMED	Hunter's syndrome, severe form	73146005
SNOMED	Chronic hepatitis (& [NOS] or [active B] or [active] or [persistent])	266539002
SNOMED	Benign neoplasm of liver	92186001
SNOMED	Hypoxia-associated cirrhosis	235897005
SNOMED	Local recurrence of malignant tumor of liver	314963000
SNOMED	Secondary malignant neoplasm of liver and intrahepatic bile duct	8.32581E+14
SNOMED	Malignant neoplasm of intrahepatic canaliculi	187776004
SNOMED	Compensation for liver failure NOS	2.91441E+14
SNOMED	Hepatitis C genotype 3	8.30781E+14
SNOMED	Chronic viral hepatitis B	8.75211E+14
SNOMED	Hepatic necrosis	87248009
SNOMED	Portal pyemia	67656006
SNOMED	Fulminant hepatic failure	235884008
SNOMED	Alpha-1-antitrypsin phenotype PiZZ	250686001
SNOMED	Secondary malignant neoplasm of liver and intrahepatic bile duct	8.13671E+14
SNOMED	Hepatic fibrosis	62484002
SNOMED	Bacterial infection by site	301811001
SNOMED	Portal fibrosis without cirrhosis	197316009
SNOMED	Alpha-1-antitrypsin isoform Z	259591003
SNOMED	Primary carcinoma of liver	187769009
SNOMED	Toxic cirrhosis	123604002
SNOMED	Multilobular portal cirrhosis	266469006
SNOMED	Other specified compensation for liver failure	2.84741E+14
SNOMED	Hepatitis C genotype 1	8.30761E+14
SNOMED	Hepatitis C genotype 6	8.24901E+14
SNOMED	Hepatic coma	72836002

SNOMED	Viral hepatitis without hepatic coma	8.32391E+14
SNOMED	Hepatitis C genotype 3	8.24871E+14
SNOMED	Hepatitis C genotype 2	8.24851E+14
SNOMED	Chronic viral hepatitis B without delta-agent	186639003
SNOMED	Hepatorenal syndrome as a complication of care	213231008
SNOMED	Neonatal hemochromatosis	6160004
SNOMED	Toxic liver disease with hepatic necrosis	197356006
SNOMED	Viral hepatitis with hepatic coma	8.12271E+14
SNOMED	Liver transplant disorder	235910007
SNOMED	(Liver cirrhosis: [named variants] or [NOS]) or (hepatic fibrosis)	197309008
SNOMED	Cystic fibrosis related cirrhosis	7.76981E+14
SNOMED	[X]Other specified carcinomas of liver	4.13961E+14
SNOMED	Conjugated hyperbilirubinemia	9326001
SNOMED	Neoplasm of liver	126851005
SNOMED	Cirrhosis of liver co-occurrent and due to primary sclerosing cholangitis (disorder)	735733008
SNOMED	Toxic liver disease with cholestasis	197355005
SNOMED	Congenital cystic disease of liver	72925005
SNOMED	Intrahepatic hematoma	262798009
SNOMED	Glycogen phosphorylase kinase deficiency, X-linked	297251003
SNOMED	Chronic type B viral hepatitis	61977001
SNOMED	Newborn physiological jaundice	276549000
SNOMED	Cystic fibrosis related cirrhosis	7.78131E+14
SNOMED	Chronic viral hepatitis B with hepatitis D	235869004
SNOMED	Chronic hepatitis C	128302006
SNOMED	Portal vein thrombosis	17920008
SNOMED	Indian childhood cirrhosis	6183001
SNOMED	Compensation for liver failure	2.91411E+14
SNOMED	Disorder of digestive system	53619000
SNOMED	Florid cirrhosis	76301009
SNOMED	Zooparasitic portal cirrhosis	197306001
SNOMED	Hepatocellular jaundice	66789005
SNOMED	Hepatitis C genotype 1	8.24841E+14
SNOMED	[X]Other chronic viral hepatitis	4.19531E+14
SNOMED	Glycogen phosphorylase kinase deficiency	235908005
SNOMED	Wilson's disease	88518009
SNOMED	Viral hepatitis C	50711007
SNOMED	Alcoholic fatty liver	50325005
SNOMED	Other specified viral hepatitis with coma	6.53331E+14
SNOMED	Primary malignant neoplasm of liver	95214007
SNOMED	Alcoholic liver damage NOS	6.40881E+14
SNOMED	Liver failure support management	7.73411E+14
SNOMED	Toxic liver disease	197354009
SNOMED	Hepatitis B carrier	235871004
SNOMED	Unconjugated hyperbilirubinemia	7752002
SNOMED	Veno-occlusive disease of the liver	65617004
SNOMED	[X]Other and unspecified cirrhosis of liver	4.70971E+14
SNOMED	Chronic non-A non-B hepatitis	235870003
SNOMED	Cerebral degeneration in Hunter's disease	192795000
SNOMED	Hepatitis C genotype 6	8.30811E+14
SNOMED	Hemochromatosis	399187006
SNOMED	Other specified viral hepatitis with hepatic coma NOS	6.01981E+14
SNOMED	Crigler-Najjar syndrome, type II	68067009

SNOMED	Hepatic coma due to viral hepatitis	40946000
SNOMED	Toxic portal cirrhosis	197301006
SNOMED	Liver disease due to cystic fibrosis	7.07011E+14
SNOMED	Portal cirrhosis unspecified	5.89551E+14
SNOMED	Glucose transport defect	237966006
SNOMED	Nodular regenerative hyperplasia of liver	715140008
SNOMED	Delayed rupture of liver	262801003
SNOMED	Hepatitis C genotype 2	8.30771E+14
SNOMED	Viral hepatitis with hepatic coma	8.32381E+14
SNOMED	Micronodular cirrhosis	21861000
SNOMED	Biliary cirrhosis	1761006
SNOMED	Other congenital anomaly of liver	5.58761E+14
SNOMED	Unilobular portal cirrhosis	197291001
SNOMED	Chronic liver disease NOS	5.92431E+14
SNOMED	Hepatitis C carrier	235872006
SNOMED	Viral hepatitis C without mention of hepatic coma	5.30081E+14
SNOMED	Chronic liver disease	328383001
SNOMED	[X]Granulomatous hepatitis, not elsewhere classified	4.18391E+14
SNOMED	Juvenile portal cirrhosis	266471006
SNOMED	Recurrent hepatitis	197286002
SNOMED	Chorea co-occurrent and due to Wilson disease	724766009
SNOMED	Dubin-Johnson syndrome	44553005
SNOMED	Primary biliary cirrhosis co-occurrent with systemic scleroderma (disorder)	715401008
SNOMED	[X]Toxic liver disease with other disorders of liver	4.19121E+14
SNOMED	Alcoholic liver damage unspecified	5.89511E+14
SNOMED	Hepatitis unspecified	5.78961E+14
SNOMED	[X]Toxic liver disease, unspecified	4.62681E+14
SNOMED	Other specified viral hepatitis without mention of hepatic coma NOS	5.61641E+14
SNOMED	Sclerosing cholangitis (disorder)	235917005
SNOMED	Infectious cirrhosis	235896001
SNOMED	[X]Diseases of the liver	4.42741E+14
SNOMED	Liver anomaly, unspecified	6.96451E+14
SNOMED	Malignant neoplasm of liver unspecified	6.20431E+14
SNOMED	Alcoholic cirrhosis	420054005
SNOMED	Diffuse nodular cirrhosis	197293003
SNOMED	Alcoholic hepatic failure	235881000
SNOMED	Hunter's syndrome, mild form	5667009
SNOMED	Chronic hepatitis	76783007
SNOMED	Liver failure NOS	6.62321E+14
SNOMED	Toxic liver disease with fibrosis and cirrhosis of liver	197362001
SNOMED	Autoimmune hepatitis	408335007
SNOMED	Congenital hepatomegaly	407000
SNOMED	Combined hepatocellular carcinoma and cholangiocarcinoma	274902006
SNOMED	Hepatorenal syndrome	51292008
SNOMED	Hepatitis C genotype 4	8.30791E+14
SNOMED	Alpha-1-antitrypsin deficiency	30188007
SNOMED	Hyperbilirubinemia (& [Gilbert's syndrome])	267509000
SNOMED	Other specified compensation for liver failure	2.91431E+14
SNOMED	Hepatic sclerosis	235899008
SNOMED	Alcoholic hepatitis	235875008
SNOMED	Cirrhosis secondary to cholestasis	271440004
SNOMED	Chronic alcoholic hepatitis	307757001

SNOMED	Macronodular cirrhosis	43904005
SNOMED	Hepatic failure as a complication of care	213230009
SNOMED	Intrahepatic phlebosclerosis and fibrosis	235902006
SNOMED	Hepatitis C genotype 5	8.30801E+14
SNOMED	Hepatitis in late syphilis	197347003
SNOMED	[X]Liver disorders in other diseases classified elsewhere	4.78481E+14
SNOMED	Congenital cystic liver disease NOS	6.00331E+14
SNOMED	Primary biliary cholangitis	31712002
SNOMED	Hepatic glycogen phosphorylase kinase deficiency	297255007
SNOMED	Cardiac glycogen phosphorylase kinase deficiency	297253000
SNOMED	Liver transplant failure	235912004
SNOMED	Hepatic infarction	17890003
SNOMED	Toxic liver disease with chronic lobular hepatitis	197360009
SNOMED	[X]Unspecified viral hepatitis with coma	4.44071E+14
SNOMED	Hepatosplenomegaly	36760000
SNOMED	Metastasis to liver of unknown primary	285613005
SNOMED	Glycogen storage disease, type VI	29291001
SNOMED	Compensation for liver failure	2.84721E+14
SNOMED	Hepatic failure	59927004
SNOMED	Congenital atrophy of left lobe of liver	204788008
SNOMED	[X]Other specified diseases of liver	4.55481E+14
SNOMED	Compensation for liver failure NOS	3.03101E+14
SNOMED	Vascular disorder of liver	235878005
SNOMED	Liver transplant failure and rejection	213153001
SNOMED	Alcoholic liver damage	41309000
SNOMED	Rotor syndrome	32891000
SNOMED	Gilbert's syndrome	27503000
SNOMED	Biliary cirrhosis (& [primary])	266540000
SNOMED	Chronic passive congestion of liver	34736002
SNOMED	Cirrhosis of liver	19943007
SNOMED	[X]Liver disorders in infectious and parasitic diseases classified elsewhere	4.78471E+14
SNOMED	Generalized glycogenosis	267424007
SNOMED	Congenital anomaly of liver	89166001
SNOMED	Glycogen storage disease	29633007
SNOMED	Primary malignant neoplasm of liver NOS	6.35021E+14
SNOMED	Fatty portal cirrhosis	197294009
SNOMED	Malignant neoplasm of intrahepatic bile ducts NOS	6.35031E+14
SNOMED	Hepatic coma due to viral hepatitis B	26206000
SNOMED	Fibrolamellar hepatocellular carcinoma	253018005
SNOMED	Focal nodular hypoplasia of liver	253814006
SNOMED	Glycogen storage disease due to acid maltase deficiency	274864009
SNOMED	Hepatoblastoma	109843000
SNOMED	Benign neoplasm of intrahepatic bile ducts	92154006
SNOMED	Klatskin's tumor	253017000
SNOMED	Chronic aggressive hepatitis	68784009
SNOMED	Hepatitis C genotype 4	8.24881E+14
SNOMED	Glycogen phosphorylase kinase deficiency, autosomal recessive	297252005
SNOMED	[X]Chronic viral hepatitis, unspecified	4.19541E+14
SNOMED	Mucopolysaccharidosis, MPS-II	70737009
SNOMED	Fitz-Hugh-Curtis syndrome	237041005
SNOMED	Congenital hepatic fibrosis	79607001
SNOMED	Hepatitis in viral diseases EC	5.28391E+14

SNOMED	Hepatic granulomas in berylliosis	197367007
SNOMED	Infectious cirrhosis NOS	5.89541E+14
SNOMED	Hyperbilirubinemia - conjugated - type III	235906009
SNOMED	Chronic hepatitis NOS	5.89531E+14
SNOMED	Cryptogenic cirrhosis	89580002
SNOMED	Portal and splenic vein sclerosis	235900003
SNOMED	Yellow atrophy of the liver	302126000
SNOMED	Angiosarcoma of liver	109844006
SNOMED	Peliosis hepatis	58008004
SNOMED	Hepatoptosis	50701000
SNOMED	Other specified liver disorder NOS	6.60831E+14
SNOMED	Toxic liver disease with chronic active hepatitis	197361008
SNOMED	Hepatic and muscle glycogen phosphorylase kinase deficiency	297254006
SNOMED	Viral hepatitis without hepatic coma	8.12281E+14
SNOMED	Subfulminant hepatic failure	235885009
SNOMED	Hepatic granulomas in sarcoidosis	197368002
SNOMED	Other specified liver disorder	5.78971E+14
SNOMED	Other specified glycogenosis	6.04411E+14
SNOMED	Compensation for liver failure	3.03091E+14
SNOMED	Chronic hepatic failure	235886005
SNOMED	Autoimmune liver disease	235890007
SNOMED	Liver cell carcinoma	109841003
SNOMED	Glycogen storage disease due to acid maltase deficiency, late-onset	722343009
SNOMED	[X]Other specified inflammatory liver diseases	4.67141E+14
SNOMED	[X]Chronic viral hepatitis, unspecified	4.32261E+14
SNOMED	Pipestem portal cirrhosis	197300007
SNOMED	Pericellular fibrosis of congenital syphilis	235898000
SNOMED	Glycogen synthase deficiency	237964009
SNOMED	Chronic yellow atrophy of liver	81675001
SNOMED	Glycogenosis NOS	6.15711E+14
SNOMED	Type B viral hepatitis	66071002
SNOMED	Conjugated hyperbilirubinemia in infancy	276687002
SNOMED	Granulomatous hepatitis	86514004
SNOMED	Other specified diseases of liver, biliary tract, pancreas or gastrointestinal tract	6.06771E+14
SNOMED	Capsular portal cirrhosis	197296006
SNOMED	Sarcoma of liver	254601002
SNOMED	Focal nodular hyperplasia of liver	278527001
SNOMED	Liver disease due to cystic fibrosis	5.26101E+14
SNOMED	Chronic active hepatitis	197284004
SNOMED	Biliary cirrhosis NOS	5.89561E+14
SNOMED	Cardiac cirrhosis	74669004
SNOMED	Alcoholic fibrosis and sclerosis of liver	235880004
SNOMED	Toxic liver disease with chronic persistent hepatitis	197359004
SNOMED	Central hemorrhagic necrosis of liver	197364000
SNOMED	Autoimmune hepatitis	1.04221E+14
SNOMED	Drug-induced chronic hepatitis	235889003
SNOMED	[X]Other congenital malformations of liver	3.98151E+14
SNOMED	Glucose-6-phosphate transport defect	30102006
SNOMED	Mixed micro and macronodular cirrhosis	15999000
SNOMED	Crigler-Najjar syndrome, type I	8933000
SNOMED	Pulmonary emphysema in alpha-1 PI deficiency	233674008
SNOMED	Compensation for liver failure NOS	2.84731E+14

SNOMED	Budd-Chiari syndrome	82385007
SNOMED	Unspecified viral hepatitis with coma	5.61651E+14
SNOMED	Hepatic: [coma] or [encephalopathy]	197332007
SNOMED	Glycogen storage disease, type VII	89597008
SNOMED	Cirrhosis and chronic liver disease	197279005
SNOMED	Malignant neoplasm of intrahepatic gall duct	187777008
SNOMED	Hepatitis C genotype 5	8.24891E+14
SNOMED	Hepatitis in other infectious diseases EC	5.97861E+14
SNOMED	Portal hypertension	34742003
SNOMED	Biliary cirrhosis of children	197310003
SNOMED	Chronic lobular hepatitis	57339008
SNOMED	Phosphate transport defect	237965005
SNOMED	Bacterial portal cirrhosis	197303009
SNOMED	[X]Toxic liver disease, unspecified	4.44681E+14
SNOMED	Hepatic coma due to viral hepatitis C	186628001
SNOMED	Alpha-1-antitrypsin hepatitis	190944000
SNOMED	Hepatic fibrosis with hepatic sclerosis	235901004
SNOMED	Cirrhosis: [cardiac portal] or [congestive]	197297002
SNOMED	Cirrhosis of liver NOS	6.62341E+14
SNOMED	Other specified compensation for liver failure	3.03111E+14
SNOMED	Metabolic and genetic disorder affecting the liver	235903001
SNOMED	Other sequelae of chronic liver disease	6.82591E+14
SNOMED	Glycogen storage disease, type V	55912009
SNOMED	Liver transplant rejection	235911006
SNOMED	Chronic viral hepatitis	10295004
SNOMED	Toxic hepatitis	197352008
SNOMED	Antichymotrypsin deficiency-alpha-1	235909002
SNOMED	Hepatitis D superinfection of hepatitis B carrier	235865005
SNOMED	Syphilitic portal cirrhosis	197305002
SNOMED	Compensation for liver failure	8.43101E+14
SNOMED	Hepatic failure NOS	6.62331E+14
SNOMED	Chronic hepatitis unspecified	5.89521E+14
SNOMED	Primary sclerosing cholangitis	197441003
SNOMED	Cardiac portal cirrhosis	266470007
SNOMED	Liver: [other congenital anomaly] or [hamartoma] or [abnormal lobulation] or [trilobular]	204800009
SNOMED	Hepatitis non-A non-B	186634008
SNOMED	Pigmentary portal cirrhosis	197299004
SNOMED	Liver disease due to cystic fibrosis	427022004
SNOMED	Carcinoma in situ of liver	92644006
SNOMED	[X]Granulomatous hepatitis, not elsewhere classified	4.25491E+14
SNOMED	Cirrhosis: [juvenile portal] or [childhood function] or [Indian childhood]	197298007
SNOMED	Hepatic coma due to viral hepatitis A	16060001
SNOMED	Portal cirrhosis	419728003
SNOMED	Alpha-1-antitrypsin deficiency	154771007
SNOMED	Malignant neoplasm of interlobular bile ducts	187773007
SNOMED	Chronic aggressive hepatitis	197285003
SNOMED	Hyperbilirubinemia	14783006
SNOMED	Cirrhosis: [florid] or [alcoholic]	197280008
SNOMED	Carcinoma in situ of intrahepatic bile ducts	92618006
SNOMED	Deficiency of glucose-6-phosphatase	124437004
SNOMED	Secondary biliary cirrhosis	12368000
SNOMED	Chronic persistent hepatitis	41889008

SNOMED	Empyema with hepatopleural fistula	34286007
SNOMED	[X]Other sarcomas of the liver	4.67201E+14
SNOMED	Intrahepatic bile duct carcinoma	109842005
Malignancy		
SNOMED	Malignant neoplasm of cervical stump (disorder)	188183000
SNOMED	Primary Ewing sarcoma of bone of rib (disorder)	723887001
SNOMED	Malignant tumor involving uterine corpus by separate metastasis from ovary (disorder)	369576002
SNOMED	Malignant tumor involving uterine corpus by separate metastasis from uterine cervix (disorder)	369577006
SNOMED	Secondary malignant neoplasm of long bone of lower limb (disorder)	94382009
SNOMED	Adult T-cell leukemia (disorder)	188729005
SNOMED	Therapy related acute myeloid leukemia due to and following administration of antineoplastic agent (disorder)	762315004
SNOMED	Malignant tumor involving vagina by separate metastasis from bladder (disorder)	369581006
SNOMED	Malignant melanoma of palate (disorder)	698286006
SNOMED	Carcinosarcoma of cervix uteri (disorder)	764951002
SNOMED	Primary malignant neoplasm of trigone of urinary bladder (disorder)	94109006
SNOMED	Refractory anemia without sideroblasts so stated (disorder)	189509003
SNOMED	Primary malignant neoplasm of soft tissue of left upper extremity (disorder)	3.52231E+14
SNOMED	Primary pulmonary lymphoma (disorder)	718200007
SNOMED	Malignant tumor of middle turbinate (disorder)	254481009
SNOMED	Malignant neoplasm of junction of hard and soft palate (disorder)	187666008
SNOMED	Carcinoma of ampulla of Vater (disorder)	254609000
SNOMED	Disseminated malignancy of unknown primary (disorder)	285645000
SNOMED	Malignant neoplasm of gastrointestinal tract (disorder)	428905002
SNOMED	Immunoproliferative neoplasm (disorder)	275524009
SNOMED	Hodgkins disease lymphocytic-histiocytic predominance of lymph nodes of inguinal region AND/OR lower limb (disorder)	93498005
SNOMED	Secondary malignant neoplasm of soft tissues of shoulder (disorder)	94593007
SNOMED	Primary malignant neoplasm of blood vessel (disorder)	93714009
SNOMED	Malignant neoplasm of upper lip lipstick area (disorder)	187601000
SNOMED	Malignant tumor involving an organ by direct extension from prostate (disorder)	369598006
SNOMED	Malignant tumor involving an organ by direct extension from uterine cervix (disorder)	369599003
SNOMED	Carcinoma of prostate (disorder)	254900004
SNOMED	Malignant tumor involving left fallopian tube by separate metastasis from endometrium (disorder)	369542006
SNOMED	Malignant tumor of mediastinum (disorder)	363494000
SNOMED	Malignant tumor involving an organ by separate metastasis from endometrium (disorder)	369603003
SNOMED	Malignant tumor involving left broad ligament by metastasis from ovary (disorder)	369538008
SNOMED	Primary malignant neoplasm of body of stomach (disorder)	93717002
SNOMED	Malignant tumor involving an organ by separate metastasis from prostate (disorder)	369606006
SNOMED	Malignant tumor of corpus cavernosum (disorder)	276420005
SNOMED	Primary malignant neoplasm of soft tissues of pelvis (disorder)	94059000
SNOMED	Malignant tumor involving an organ by separate metastasis from vagina (disorder)	369609004
SNOMED	Secondary malignant neoplasm of tip and lateral border of tongue (disorder)	94637003
SNOMED	Microsatellite instability-high solid malignant tumor (disorder)	735385007
SNOMED	Primary malignant neoplasm of laryngeal aspect of aryepiglottic fold (disorder)	371994002
SNOMED	Diffuse non-Hodgkins lymphoma undifferentiated (diffuse) (disorder)	188679001
SNOMED	Malignant tumor of urinary tract proper (disorder)	363517008
SNOMED	Primary malignant neoplasm of hamate bone (disorder)	93820003
SNOMED	Sarcoma of posterior mediastinum (disorder)	448259002
SNOMED	Secondary malignant neoplasm of soft tissues of upper limb (disorder)	94596004
SNOMED	Hodgkins disease lymphocytic-histiocytic predominance of lymph nodes of head face AND/OR neck (disorder)	93497000
SNOMED	Primary malignant neoplasm of blood vessel of pelvis (disorder)	93705004
SNOMED	Carcinoma of abdominal part of esophagus (disorder)	254543002
SNOMED	Primary malignant neoplasm of anterior wall of nasopharynx (disorder)	93674007

SNOMED	Secondary malignant neoplasm of chest wall (disorder)	94253005
SNOMED	Secondary malignant neoplasm of skin of hand (disorder)	94560003
SNOMED	Malignant lymphoma of extranodal AND/OR solid organ site (disorder)	93199007
SNOMED	Carcinoma of cardia (disorder)	254553001
SNOMED	Malignant neoplasm of body of uterus (disorder)	371972005
SNOMED	Malignant neoplasm of uterus (disorder)	371973000
SNOMED	Primary malignant neoplasm of peripheral nerve (disorder)	422736007
SNOMED	Primary malignant neoplasm of blood vessel of inguinal region (disorder)	93701008
SNOMED	Primary squamous cell carcinoma of upper third of esophagus (disorder)	721618006
SNOMED	Primary malignant neoplasm of cecum (disorder)	371977004
SNOMED	Primary malignant neoplasm of cervical esophagus (disorder)	371978009
SNOMED	Acute myeloid leukemia with FMS-like tyrosine kinase-3 mutation (disorder)	734522002
SNOMED	Primary squamous cell carcinoma of upper limb (disorder)	2.4111E+13
SNOMED	Primary malignant neoplasm of commissure of lip (disorder)	371981004
SNOMED	Primary malignant neoplasm of craniopharyngeal duct (disorder)	93768004
SNOMED	Primary malignant neoplasm of endocrine gland (disorder)	371983001
SNOMED	Malignant fibrous histiocytoma of orbit (disorder)	254996003
SNOMED	Primary malignant neoplasm of eye (disorder)	371986009
SNOMED	Secondary malignant neoplasm of face (disorder)	94293008
SNOMED	[X]Melanoma and other malignant neoplasms of skin (disorder)	3.96181E+14
SNOMED	Primary malignant neoplasm of glans penis (disorder)	371989002
SNOMED	Kaposis sarcoma of lymph nodes (disorder)	109391009
SNOMED	Primary malignant neoplasm of hand (disorder)	93821004
SNOMED	Primary malignant neoplasm of intestinal tract (disorder)	371992003
SNOMED	Primary malignant neoplasm of ethmoidal sinus (disorder)	93787005
SNOMED	Malignant tumor involving left fallopian tube by separate metastasis from ovary (disorder)	369543001
SNOMED	Primary malignant neoplasm of soft tissues of inguinal region (disorder)	94056007
SNOMED	Sarcoma (disorder)	424413001
SNOMED	Primary mucinous cystadenocarcinoma of trachea (disorder)	707383000
SNOMED	Primary malignant neoplasm of lower third of esophagus (disorder)	371998004
SNOMED	Secondary malignant neoplasm of labia majora (disorder)	94361003
SNOMED	Secondary malignant neoplasm of axillary lymph nodes (disorder)	94181007
SNOMED	Acute myeloid leukemia with t(8;16)(p11;p13) translocation (disorder)	725390002
SNOMED	Secondary malignant neoplasm of paramammary lymph nodes (disorder)	94463004
SNOMED	Primary malignant neoplasm of pancreas (disorder)	372003004
SNOMED	Primary malignant neoplasm of choroid primary (disorder)	93755007
SNOMED	Secondary malignant neoplasm of blood vessel of lower leg (disorder)	400058002
SNOMED	Benign paraproteinemia	395692003
SNOMED	Secondary malignant neoplasm of skin of groin (disorder)	94559008
SNOMED	Cystadenocarcinoma of ovary (disorder)	314191009
SNOMED	Primary malignant neoplasm of scrotum (disorder)	372009000
SNOMED	Malignant melanoma arising in intradermal nevus (disorder)	254733003
SNOMED	Carcinoma of head of pancreas (disorder)	326072005
SNOMED	Acute myeloid leukemia and myelodysplastic syndrome related to alkylating agent (disorder)	766045006
SNOMED	Malignant neoplasm of connective and soft tissue of forearm (disorder)	187994003
SNOMED	Secondary malignant neoplasm of lacrimal drainage structure (disorder)	418529003
SNOMED	Mantle cell lymphoma (disorder)	443487006
SNOMED	Malignant tumor of laryngeal cartilage (disorder)	363431006
SNOMED	Primary mucoepidermoid carcinoma of hypopharynx (disorder)	707406005
SNOMED	Malignant neoplasm of thoracic vertebral column (disorder)	372018003
SNOMED	Metastasis to liver from adenocarcinoma (disorder)	1.691E+12
SNOMED	Squamous cell carcinoma of gum (disorder)	276953005

SNOMED	Malignant neoplasm of temporal lobe (disorder)	363468009
SNOMED	Malignant neoplasm of cerebral pia mater (disorder)	188315007
SNOMED	Adenoid cystic carcinoma of trachea (disorder)	254619006
SNOMED	Primary malignant neoplasm of uterine cervix (disorder)	372024009
SNOMED	Secondary malignant neoplasm of exocervix (disorder)	94290006
SNOMED	Malignant neoplasm of hand bones (& [carpal bones] or [metacarpal bones])	187936004
SNOMED	Primary malignant neoplasm of border of tongue (disorder)	371975007
SNOMED	Subacute leukemia (disorder)	302855005
SNOMED	Malignant tumor of lymphoid hemopoietic AND/OR related tissue (disorder)	269475001
SNOMED	Malignant melanoma of frontal sinus (disorder)	707361004
SNOMED	Carcinoma of breast (disorder)	254838004
SNOMED	Malignant neoplasm of female breast (disorder)	372064008
SNOMED	Primary malignant neuroepitheliomatous neoplasm of autonomic nervous system (disorder)	724806004
SNOMED	Hormone refractory prostate cancer (disorder)	427492003
SNOMED	Primary malignant neoplasm of posterior wall of oropharynx (disorder)	93971002
SNOMED	Carcinoma of descending colon (disorder)	312113007
SNOMED	Primary mucinous carcinoma of digestive organ (disorder)	733356003
SNOMED	Hodgkins disease lymphocytic depletion of lymph nodes of multiple sites (disorder)	188593003
SNOMED	Carcinoma of male breast (disorder)	372096000
SNOMED	Malignant neoplasm of ribs and/or sternum and/or clavicle (disorder)	187920001
SNOMED	Primary malignant neoplasm of abducens nerve (disorder)	93657007
SNOMED	Malignant melanoma of skin of canthus of eye (disorder)	423280002
SNOMED	Carcinoma of exocervix (disorder)	372100004
SNOMED	Carcinoma of vocal cord (disorder)	372141009
SNOMED	Secondary malignant neoplasm of intrathoracic lymph nodes (disorder)	94351005
SNOMED	Secondary malignant neoplasm of unknown site (disorder)	274088005
SNOMED	Malignant tumor of ethmoid sinus (disorder)	363426009
SNOMED	Refractory anemia with excess blasts (disorder)	398623004
SNOMED	Lymphoma of fundus of stomach (disorder)	447658000
SNOMED	Hypomelanotic mycosis fungoides (disorder)	404110001
SNOMED	Primary malignant neoplasm of clitoris (disorder)	371980003
SNOMED	Primary malignant neuroendocrine neoplasm of bronchus (disorder)	722527003
SNOMED	Primary malignant neoplasm of middle lobe bronchus or lung (disorder)	372112000
SNOMED	Adult T-cell leukemia/lymphoma (disorder)	110007008
SNOMED	Secondary malignant neoplasm of ethmoidal sinus (disorder)	94288005
SNOMED	Diffuse non-Hodgkins lymphoma of tonsil (disorder)	449173006
SNOMED	[X]Additional neoplasm classification terms (disorder)	4.25111E+14
SNOMED	Secondary malignant neoplasm of lymph nodes of neck from thyroid (disorder)	1.33514E+16
SNOMED	Secondary malignant neoplasm of lingual tonsil (disorder)	94379004
SNOMED	Secondary malignant neoplasm of main bronchus (disorder)	94399005
SNOMED	Malignant neoplasm of intermediate cuneiform (disorder)	187969004
SNOMED	Primary small cell malignant neoplasm of lung TNM stage 4 (disorder)	6.7841E+13
SNOMED	Malignant tumor involving vagina by direct extension from ovary (disorder)	369512008
SNOMED	Follicular non-Hodgkins lymphoma small cleaved cell (disorder)	109970006
SNOMED	Plasma cell disorder	277576009
SNOMED	Myxoid liposarcoma (disorder)	404069006
SNOMED	Hodgkins sarcoma of intrathoracic lymph nodes (disorder)	93549004
SNOMED	Borderline malignant melanoma (disorder)	403921000
SNOMED	Malignant neoplasm of cerebrum (excluding lobes and ventricles) (disorder)	188280007
SNOMED	Sarcoma of connective tissue (disorder)	449293008
SNOMED	Primary malignant neoplasm of muscle of pelvis (disorder)	93907002
SNOMED	SÃƒ Â©zarys disease of intra-abdominal lymph nodes (disorder)	188632001

SNOMED	Microglioma (disorder)	307649006
SNOMED	Malignant neoplasm of bone and articular cartilage (disorder)	1.09082E+15
SNOMED	Malignant neoplasm of nasopharyngeal wall (disorder)	240163000
SNOMED	Carcinoma common bile duct (disorder)	255086002
SNOMED	Malignant tumor involving left ovary by separate metastasis from vagina (disorder)	369564002
SNOMED	Primary malignant neoplasm of upper arm (disorder)	93682007
SNOMED	Primary malignant neoplasm of breast lower inner quadrant (disorder)	373090000
SNOMED	Malignant neoplasm of pharynx (disorder)	363507003
SNOMED	Primary squamous cell carcinoma of palatine tonsil (disorder)	1.8121E+13
SNOMED	Triple negative malignant neoplasm of breast (disorder)	706970001
SNOMED	Secondary malignant neoplasm of cubital lymph nodes (disorder)	94268006
SNOMED	Secondary malignant neoplasm of bone marrow (disorder)	94217008
SNOMED	Malignant tumor involving uterine corpus by direct extension from fallopian tube (disorder)	369579009
SNOMED	Secondary malignant neoplasm of cerebral meninges (disorder)	94246001
SNOMED	Secondary malignant neoplasm of sphenoidal sinus (disorder)	94599006
SNOMED	Aleukemic myeloid leukemia (disorder)	188741003
SNOMED	Small cell carcinoma of prostate (disorder)	396198006
SNOMED	Lymphoma of gastrointestinal tract (disorder)	449072004
SNOMED	Secondary malignant neoplasm of conjunctiva (disorder)	94263002
SNOMED	MiT family translocation renal cell carcinoma (disorder)	764694005
SNOMED	Secondary malignant neoplasm of ventral surface of tongue (disorder)	94672000
SNOMED	Malignant tumor of penis (disorder)	363516004
SNOMED	Non-Hodgkins lymphoma (disorder)	118601006
SNOMED	Carcinoma of penis (disorder)	372106005
SNOMED	Primary malignant neoplasm of ectopic male breast tissue (disorder)	93777006
SNOMED	Malignant peritoneal local recurrence (disorder)	277782009
SNOMED	Malignant tumor involving right fallopian tube by separate metastasis from ovary (disorder)	369555007
SNOMED	Malignant tumor involving left fallopian tube by direct extension from ovary (disorder)	369516006
SNOMED	(Aleukemic leukemia) or (leukemia - unspec cell NOS)	269633006
SNOMED	Squamous cell carcinoma of prostate (disorder)	399590005
SNOMED	Malignant tumor of intestine (disorder)	363508008
SNOMED	Secondary malignant neoplasm of lower leg (disorder)	449632009
SNOMED	Secondary malignant neoplasm of blood vessel of upper arm (disorder)	399969009
SNOMED	Metastasis from malignant tumor of uterus (disorder)	314993006
SNOMED	Malignant melanoma of skin of toe (disorder)	93650009
SNOMED	Malignant neoplasm of connective and soft tissue of pelvis (disorder)	188019007
SNOMED	Leukemic infiltrate of retina (disorder)	314418005
SNOMED	Sarcoma of omentum (disorder)	448450001
SNOMED	Secondary malignant neoplasm of intestinal lymph nodes (disorder)	94345000
SNOMED	Primary squamous cell carcinoma of lacrimal apparatus (disorder)	722666002
SNOMED	Congenital malignant melanoma (disorder)	402559007
SNOMED	Malignant neoplasm of liver (disorder)	93870000
SNOMED	Malignant melanoma of soft tissues (disorder)	402561003
SNOMED	Follicular non-Hodgkins lymphoma of tonsil (disorder)	449058008
SNOMED	Primary cutaneous CD8 positive aggressive epidermotropic cytotoxic T-cell lymphoma (disorder)	765136002
SNOMED	Metastasis to multiple lymph nodes (disorder)	303201005
SNOMED	Hodgkin lymphoma nodular lymphocyte predominance (disorder)	118605002
SNOMED	Squamous cell carcinoma (disorder)	402815007
SNOMED	T-cell hystiocyte rich large B-cell lymphoma (disorder)	724645006
SNOMED	Malignant neoplasm of cerebrum (disorder)	429033009
SNOMED	Malignant neoplasm of axial suprasellar region of brain (disorder)	448248006
SNOMED	Primary malignant neoplasm of adrenal gland (disorder)	93665005

SNOMED	Local recurrence of malignant tumor of urinary bladder (disorder)	314968009
SNOMED	Sarcomatous metastasis in skin (disorder)	404093001
SNOMED	Primary cutaneous B-cell lymphoma (disorder)	402881008
SNOMED	Overlapping malignant neoplasm of tonsil (disorder)	110013004
SNOMED	Malignant glioma of central nervous system (disorder)	8.7151E+13
SNOMED	B lymphoblastic leukemia lymphoma with t(9:22) (q34;q11.2); BCR-ABL 1 (disorder)	723889003
SNOMED	Carcinoma of lesser curve of stomach (disorder)	254563009
SNOMED	Secondary malignant neoplasm of laryngeal aspect of interarytenoid fold (disorder)	94367004
SNOMED	Malignant epithelial neoplasm of maxilla (disorder)	449077005
SNOMED	CD-30 positive anaplastic large T-cell cutaneous lymphoma (disorder)	404125001
SNOMED	Primary malignant neoplasm of mediastinum (disorder)	93891008
SNOMED	Immunoglobulin D myeloma (disorder)	285422003
SNOMED	Burkitts tumor of spleen (disorder)	92515003
SNOMED	Nephroblastoma (disorder)	302849000
SNOMED	Sarcoma of ovary (disorder)	423627007
SNOMED	Signet ring squamous cell carcinoma (disorder)	403903003
SNOMED	Primary malignant neoplasm of parotid gland (disorder)	372004005
SNOMED	Secondary malignant neoplasm of anterior cervical lymph nodes (disorder)	1.09013E+15
SNOMED	Therapy-related myelodysplastic syndrome (disorder)	702476004
SNOMED	Primary malignant neoplasm of scaphoid bone (disorder)	372008008
SNOMED	Primary malignant neoplasm of occipital lobe (disorder)	93928006
SNOMED	Primary neuroendocrine carcinoma of stomach (disorder)	721638007
SNOMED	Cutaneous CD30+ lymphoproliferative disorder (disorder)	128874001
SNOMED	Primary verrucous carcinoma of hypopharynx (disorder)	707490009
SNOMED	Primary adenocarcinoma of accessory sinus (disorder)	707337006
SNOMED	Malignant neoplasm of parametrium (disorder)	448674007
SNOMED	Primary cutaneous anaplastic large cell B-cell lymphoma (disorder)	404145009
SNOMED	Kaposis sarcoma of skin (disorder)	109386008
SNOMED	Adenocarcinoma of scrotum (disorder)	447800002
SNOMED	Squamous cell carcinoma of bladder (disorder)	255111004
SNOMED	Metastasis from malignant tumor of bone (disorder)	314990009
SNOMED	Malignant chondroid syringoma of skin (disorder)	403943008
SNOMED	Kaposis sarcoma of eyelid (disorder)	231835006
SNOMED	Perianal Pagets disease (disorder)	403947009
SNOMED	Apocrine adenocarcinoma of skin (disorder)	403949007
SNOMED	Primary adenocarcinoma of body of pancreas (disorder)	6.81621E+14
SNOMED	Follicular non-Hodgkins lymphoma mixed small cleaved cell and large cell (disorder)	109971005
SNOMED	Hodgkins disease nodular sclerosis of lymph nodes of inguinal region and lower limb (disorder)	188569008
SNOMED	Malignant tumor of major salivary gland (disorder)	363378008
SNOMED	Angiosarcoma (disorder)	403977003
SNOMED	Secondary malignant neoplasm of skin of back (disorder)	94543008
SNOMED	Secondary malignant neoplasm of skin of ankle (disorder)	94540006
SNOMED	Secondary malignant neoplasm of penis (disorder)	94481001
SNOMED	Secondary malignant neoplasm of cerebellum (disorder)	94245002
SNOMED	Retinal pigment epithelial adenocarcinoma (disorder)	312949007
SNOMED	Secondary malignant neoplasm of trunk (disorder)	94649002
SNOMED	Malignant histiocytic disorder (disorder)	127070008
SNOMED	Primary malignant neoplasm of palate (disorder)	372002009
SNOMED	Secondary malignant neoplasm of middle third of esophagus (disorder)	94414003
SNOMED	Giant cell malignant fibrous histiocytoma of skin (disorder)	404016005
SNOMED	Malignant tumor of pelviureteric junction (disorder)	188253000
SNOMED	Primary malignant neoplasm of soft tissues of head (disorder)	94054005

SNOMED	Malignant tumor of vermilion border of upper lip (disorder)	363372009
SNOMED	Malignant skin tumor with eccrine differentiation (disorder)	254707006
SNOMED	Malignant neoplasm of carpal bone - capitate (disorder)	187943005
SNOMED	Primary spindle cell squamous cell carcinoma of larynx (disorder)	707422006
SNOMED	Primary squamous cell carcinoma of sphenoidal sinus (disorder)	707355002
SNOMED	Acute myeloid leukemia in remission (disorder)	91860005
SNOMED	Squamous cell carcinoma of lip (disorder)	255071008
SNOMED	Embryonal rhabdomyosarcoma (disorder)	404051002
SNOMED	Primary malignant neoplasm of accessory nerve (disorder)	93658002
SNOMED	Primary malignant neoplasm of epicardium (disorder)	93782004
SNOMED	Secondary malignant neoplasm of lymph nodes of lower limb (disorder)	94395004
SNOMED	Malignant pinealoma (disorder)	255044008
SNOMED	Secondary malignant neoplasm of skin of elbow (disorder)	94550007
SNOMED	Mast cell malignancy of lymph nodes of axilla and upper limb (disorder)	188665009
SNOMED	Carcinoma of vermilion border of upper lip (disorder)	254389005
SNOMED	Malignant melanoma of skin of buttock (disorder)	93216007
SNOMED	Round cell liposarcoma (disorder)	404070007
SNOMED	Pleomorphic liposarcoma (disorder)	404071006
SNOMED	Dedifferentiated liposarcoma (disorder)	404072004
SNOMED	Malignant melanoma of groin (disorder)	188052001
SNOMED	Primary malignant neoplasm of female breast (disorder)	93796005
SNOMED	Primary malignant neoplasm of bone of left lower limb (disorder)	3.54651E+14
SNOMED	Primary pseudosarcomatous carcinoma of lung (disorder)	707460002
SNOMED	Malignant neoplasm of carpal bone - triquetrum (disorder)	187939006
SNOMED	Chordoma of clivus (disorder)	446939001
SNOMED	Sarcoma of vertebra (disorder)	448776002
SNOMED	Malignant neoplasm of humerus (disorder)	187932002
SNOMED	Malignant lymphoma of intra-abdominal lymph nodes (disorder)	93191005
SNOMED	SÃƒ Â©zarys disease of intrapelvic lymph nodes (disorder)	188635004
SNOMED	Primary malignant neuroendocrine neoplasm of colon (disorder)	721698005
SNOMED	Lymphoma of intestine (disorder)	276815004
SNOMED	Extrauterine adenocarcinoma (disorder)	373888000
SNOMED	Malignant tumor of tunica vaginalis (disorder)	188235006
SNOMED	Secondary malignant neoplasm of soft tissues of axilla (disorder)	94583000
SNOMED	Metastatic neuroblastoma (disorder)	704152002
SNOMED	Primary malignant neuroepitheliomatous neoplasm of nasal cavity (disorder)	735450006
SNOMED	Primary squamous cell carcinoma of urethra (disorder)	1.07591E+14
SNOMED	Poikilodermatous mycosis fungoides (disorder)	404108003
SNOMED	Follicular mucinosis type mycosis fungoides (disorder)	404109006
SNOMED	Primary malignant neoplasm of hilus of lung (disorder)	93827000
SNOMED	Malignant neoplasm of adrenal cortex (disorder)	371964008
SNOMED	Primary malignant neoplasm of conjunctiva primary (disorder)	93764002
SNOMED	Diffuse non-Hodgkins lymphoma (disorder)	109962001
SNOMED	Malignant tumor involving left ovary by direct extension from right ovary (disorder)	369526004
SNOMED	Siewert type II adenocarcinoma (disorder)	3.42511E+14
SNOMED	Malignant neoplasm of cervix uteri (& carcinoma)	188174005
SNOMED	Malignant neoplasm of vaginal vault (disorder)	188209005
SNOMED	Transitional cell carcinoma of ureter (disorder)	300988009
SNOMED	Malignant tumor involving right broad ligament by metastasis from ovary (disorder)	369540003
SNOMED	Malignant melanoma of skin of lower leg (disorder)	449636007
SNOMED	Breast cancer detected by national screening programme (disorder)	9.4361E+13
SNOMED	Leukemia disease (disorder)	93143009

SNOMED	Leukemia in remission (disorder)	93142004
SNOMED	Primary malignant neoplasm of right fallopian tube (disorder)	369520005
SNOMED	Hodgkins sarcoma of lymph nodes of axilla and upper limb (disorder)	188547001
SNOMED	Primary cutaneous immunocytoma (disorder)	404141000
SNOMED	Malignant tumor involving rectum by separate metastasis from vagina (disorder)	369461007
SNOMED	T-cell leukemic infiltration of skin (disorder)	402879006
SNOMED	Malignant tumor of upper limb (disorder)	363503004
SNOMED	Regressed malignant testicular tumor (disorder)	254912000
SNOMED	Primary carcinoma of accessory sinus (disorder)	707345001
SNOMED	Angioimmunoblastic T-cell lymphoma (disorder)	413537009
SNOMED	Primary malignant neoplasm of upper gum (disorder)	372022008
SNOMED	Non-Hodgkins lymphoma of nasopharynx (disorder)	448371005
SNOMED	Primary adenocarcinoma of urethra (disorder)	733136006
SNOMED	Aggressive natural killer-cell leukemia involving skin (disorder)	404136008
SNOMED	Primary malignant neoplasm of bone of right lower limb (disorder)	3.54681E+14
SNOMED	Malignant odontogenic neoplasm of upper jaw (disorder)	710196003
SNOMED	Primary adenocarcinoma of cystic duct (disorder)	721558004
SNOMED	Secondary malignant neoplasm of popliteal lymph nodes (disorder)	94494004
SNOMED	Secondary malignant neoplasm of great vessels (disorder)	94319009
SNOMED	Secondary malignant neoplasm of soft tissues of buttock (disorder)	94584006
SNOMED	Primary cutaneous follicular center B-cell lymphoma (disorder)	404143002
SNOMED	Malignant melanoma stage IA (finding)	9.56331E+14
SNOMED	Malignant tumor of tongue (disorder)	363375006
SNOMED	Sarcoma lower inner quadrant of female breast (disorder)	448435005
SNOMED	Secondary malignant neoplasm of vomer (disorder)	94680007
SNOMED	Peripheral neuroectodermal tumor (disorder)	253096008
SNOMED	Follicular non-Hodgkins lymphoma of extranodal site (disorder)	448561007
SNOMED	Olfactory neuroblastoma (disorder)	422886007
SNOMED	Secondary malignant neoplasm of carpal bone (disorder)	94240007
SNOMED	Malignant neoplasm of junctional region of epiglottis (disorder)	187685006
SNOMED	Overlapping squamous cell carcinoma of hypopharynx (disorder)	707628004
SNOMED	Secondary malignant neoplasm of skin of perineum (disorder)	94568005
SNOMED	Primary malignant neoplasm of blood vessel of axilla (disorder)	93692004
SNOMED	Carcinoma of extrahepatic bile duct (disorder)	372101000
SNOMED	Mixed cell type lymphosarcoma of intrapelvic lymph nodes (disorder)	94687005
SNOMED	Overlapping malignant neoplasm of lip oral cavity and/or pharynx (disorder)	109833003
SNOMED	Secondary malignant neoplasm of muscle of inguinal region (disorder)	94423000
SNOMED	Sarcoma of male breast (disorder)	448257000
SNOMED	Primary malignant neoplasm of upper outer quadrant of female breast (disorder)	94117003
SNOMED	Precursor T cell lymphoblastic leukemia/lymphoblastic lymphoma (disorder)	420890002
SNOMED	Adenocarcinoma of rectum (disorder)	254582000
SNOMED	Transitional cell carcinoma of kidney (disorder)	408642003
SNOMED	Secondary malignant neoplasm of nasal concha (disorder)	94437009
SNOMED	Malignant melanoma of floor of mouth (disorder)	698043002
SNOMED	Secondary malignant neoplasm of descending colon (disorder)	94271003
SNOMED	Adenocarcinoma of liver (disorder)	408646000
SNOMED	Primary malignant neoplasm of muscle (disorder)	93913006
SNOMED	Squamous cell carcinoma of epiglottis (disorder)	408648004
SNOMED	Malignant neoplasm of globus pallidus (disorder)	188285002
SNOMED	Primary malignant neoplasm of endometrium (disorder)	93781006
SNOMED	Malignant tumor of ectopic testis (disorder)	188220005
SNOMED	Malignant histiocytosis of intrapelvic lymph nodes (disorder)	93183001

SNOMED	Undifferentiated carcinoma of ovary (disorder)	254856004
SNOMED	Primary undifferentiated carcinoma of trachea (disorder)	707385007
SNOMED	Primary mucoepidermoid carcinoma of lung (disorder)	707465007
SNOMED	Secondary malignant neoplasm of spleen (disorder)	94603006
SNOMED	Adenocarcinoma of cecum (disorder)	413446001
SNOMED	Pleomorphic small/medium-sized cell cutaneous T-cell lymphoma (disorder)	404132005
SNOMED	Malignant neoplasm of clitoris (disorder)	371979001
SNOMED	Blastic phase chronic myeloid leukemia (disorder)	413656006
SNOMED	Intraocular non-Hodgkin malignant lymphoma (disorder)	420788006
SNOMED	Sarcoma of back (disorder)	448711001
SNOMED	Primary small cell non-keratinizing squamous cell carcinoma of lung (disorder)	707408006
SNOMED	Extrarenal rhabdoid tumor (disorder)	404089007
SNOMED	Merkel cell carcinoma (disorder)	253001006
SNOMED	Primary malignant neoplasm of endocardium (disorder)	93778001
SNOMED	Malignant melanoma of skin of scalp (disorder)	93646002
SNOMED	Low-grade fibromyxoid sarcoma (disorder)	404088004
SNOMED	Myelodysplastic syndrome unclassified by World Health Organization classification (disorder)	414791003
SNOMED	Malignant tumor of cranial nerve (disorder)	188307009
SNOMED	Malignant melanoma of left choroid (disorder)	1.08094E+15
SNOMED	Follicular malignant lymphoma - large cell (disorder)	277641001
SNOMED	Malignant neoplasm of cartilage of ear (disorder)	187987001
SNOMED	Primary malignant neoplasm of penis (disorder)	372005006
SNOMED	Primary malignant neoplasm of body of penis (disorder)	93716006
SNOMED	Secondary malignant neoplasm of undescended testis (disorder)	94651003
SNOMED	Squamous cell carcinoma of bronchus in left lower lobe (disorder)	313353007
SNOMED	Primary adenocarcinoma of distal third of esophagus (disorder)	1.28041E+14
SNOMED	Primary undifferentiated large cell malignant neoplasm of chest wall (disorder)	9.1171E+13
SNOMED	Primary malignant neoplasm of neck (disorder)	93922007
SNOMED	Primary malignant neoplasm of ilium (disorder)	93833009
SNOMED	Classic medulloblastoma (disorder)	699704002
SNOMED	Malignant tumor involving seminal vesicle by direct extension from bladder (disorder)	369489005
SNOMED	Secondary carcinoma of gastrointestinal tract (disorder)	286902000
SNOMED	Secondary malignant neoplasm of radius (disorder)	94508007
SNOMED	Malignant epithelial neoplasm of bronchus (disorder)	733144006
SNOMED	Carcinomatosis (disorder)	307593001
SNOMED	T-cell chronic lymphocytic leukemia (disorder)	277545003
SNOMED	Squamous cell carcinoma of mucous membrane of lower lip (disorder)	418372008
SNOMED	Primary squamous cell carcinoma of pyriform sinus (disorder)	707704007
SNOMED	Primary malignant neoplasm of blood vessel of hip (disorder)	93700009
SNOMED	Malignant neoplasm soft tissues of cervical spine (disorder)	187989003
SNOMED	Squamous cell carcinoma of oral mucous membrane (disorder)	419842002
SNOMED	Malignant lymphoma - centrocytic (disorder)	307625008
SNOMED	Multiple endocrine neoplasia type 1 (disorder)	30664006
SNOMED	Primary adenocarcinoma of lower third of esophagus due to Barrett esophagus (disorder)	721617001
SNOMED	Chronic myeloid leukemia in lymphoid blast crisis (disorder)	413842007
SNOMED	Widespread metastatic malignant neoplastic disease (disorder)	405843009
SNOMED	Malignant tumor involving rectum by direct extension from uterine cervix (disorder)	369452007
SNOMED	Refractory cytopenia with multilineage dysplasia and ringed sideroblasts (disorder)	415286005
SNOMED	Primary lymphoma of brain associated with acquired immunodeficiency syndrome (disorder)	421283008
SNOMED	Malignant neoplasm associated with acquired immunodeficiency syndrome (disorder)	422282000
SNOMED	Malignant tumor of vertebral column (disorder)	363438000
SNOMED	Overlapping malignant neoplasm of soft tissues (disorder)	109349007

SNOMED	Primary malignant neoplasm of oropharynx (disorder)	93933005
SNOMED	Secondary malignant neoplasm of articular cartilage (disorder)	128465005
SNOMED	Carcinoma of nasal meatus (disorder)	422758009
SNOMED	Primary malignant neoplasm of ovary with widespread metastatic disease (disorder)	422782004
SNOMED	Malignant tumor of lateral nasal wall (disorder)	255075004
SNOMED	Secondary malignant neoplasm of hypopharyngeal aspect of interarytenoid fold (disorder)	94333009
SNOMED	Primary basaloid squamous cell carcinoma of larynx (disorder)	707662004
SNOMED	Overlapping malignant neoplasm of palate (disorder)	109831001
SNOMED	Primary squamous cell carcinoma of anterior surface of epiglottis (disorder)	707537007
SNOMED	Angiosarcoma of liver (disorder)	109844006
SNOMED	Secondary malignant neoplasm of temporal lobe (disorder)	94622002
SNOMED	Secondary malignant neoplasm of myocardium (disorder)	94433008
SNOMED	Malignant neoplasm of calcaneum (disorder)	187967002
SNOMED	Spindle cell liposarcoma (disorder)	404073009
SNOMED	Malignant tumor of soft tissue of shoulder (disorder)	363363001
SNOMED	Primary malignant neuroendocrine neoplasm of cystic duct (disorder)	733348001
SNOMED	Malignant neoplasm of breast upper inner quadrant (disorder)	373082000
SNOMED	Myxoid leiomyosarcoma of skin (disorder)	404046008
SNOMED	Malignant tumor involving bladder by separate metastasis from ovary (disorder)	369478003
SNOMED	Primary malignant neuroendocrine neoplasm of rectum (disorder)	721700001
SNOMED	Malignant neoplasm of navicular (disorder)	187972006
SNOMED	Local recurrence of malignant tumor of colon (disorder)	314965007
SNOMED	Malignant tumor of kidney (disorder)	363518003
SNOMED	Secondary malignant neoplasm of multiple endocrine glands (disorder)	94417005
SNOMED	Malignant tumor of renal calyx (disorder)	188252005
SNOMED	Carcinoma of pancreas (disorder)	372142002
SNOMED	Squamous cell carcinoma of external auditory canal (disorder)	423506005
SNOMED	Malignant histiocytosis of lymph nodes of inguinal region AND/OR lower limb (disorder)	93187000
SNOMED	Large cell carcinoma of lung TNM stage 4 (disorder)	423600008
SNOMED	Secondary malignant neoplasm of clitoris (disorder)	94257006
SNOMED	Malignant tumor of small intestine (disorder)	363509000
SNOMED	Primary malignant neoplasm of lesser curvature of stomach (disorder)	93867004
SNOMED	Primary malignant neoplasm of peripheral nerve of head face and/or neck (disorder)	109921007
SNOMED	Mucoepidermoid carcinoma of skin (disorder)	254713002
SNOMED	Follicular non-Hodgkins lymphoma of nose (disorder)	448231003
SNOMED	Squamous cell carcinoma of auricle of ear (disorder)	423700001
SNOMED	Stewart-Treves syndrome (disorder)	62497000
SNOMED	Secondary malignant neoplasm of sigmoid colon (disorder)	94538001
SNOMED	Primary malignant neoplasm of lacrimal caruncle (disorder)	735918005
SNOMED	Diffuse non-Hodgkins lymphoma of oral cavity (disorder)	448468003
SNOMED	Malignant tumor of esophagus stomach and duodenum (disorder)	255078002
SNOMED	Hodgkins disease of intra-abdominal lymph nodes (disorder)	93520007
SNOMED	Malignant melanoma of skin of thigh (disorder)	93649009
SNOMED	Carcinoma of breast - axillary tail (disorder)	286897001
SNOMED	Chronic leukemia disease (disorder)	92812005
SNOMED	Primary malignant neoplasm of parametrium (disorder)	93942003
SNOMED	Malignant seminoma of mediastinum (disorder)	278043000
SNOMED	Nodular high grade B-cell lymphoma (disorder)	277627005
SNOMED	Secondary malignant neoplasm of nasopharyngeal wall (disorder)	242862004
SNOMED	Secondary malignant neoplasm of deep cervical lymph nodes (disorder)	1.09012E+15
SNOMED	Malignant neoplasm of carina of bronchus (disorder)	187857006
SNOMED	Lymphoma of pylorus of stomach (disorder)	447656001

SNOMED	Secondary malignant neoplasm of spinal cord (disorder)	94600009
SNOMED	Primary serous carcinoma of uterine adnexa (disorder)	722686003
SNOMED	Secondary malignant neoplasm of lung (disorder)	94391008
SNOMED	Malignant melanoma of oral cavity (disorder)	403926005
SNOMED	Malignant neoplasm of corpus callosum (disorder)	188301005
SNOMED	Secondary malignant neoplasm of sacral lymph nodes (disorder)	1.09017E+15
SNOMED	Secondary malignant neoplasm of maxilla (disorder)	94405008
SNOMED	Local recurrence of malignant tumor of lung (disorder)	314954002
SNOMED	Undifferentiated large cell carcinomatosis (disorder)	424954002
SNOMED	African Burkitts lymphoma (disorder)	240531002
SNOMED	Secondary malignant neoplasm of bone of lower limb (disorder)	94219006
SNOMED	Lymphomatoid papulosis with Hodgkins disease (disorder)	404106004
SNOMED	Secondary malignant neoplasm of epitrochlear lymph nodes (disorder)	94285008
SNOMED	Secondary malignant neoplasm of inner aspect of lip (disorder)	94341009
SNOMED	Overlapping malignant neoplasm of stomach (disorder)	109836006
SNOMED	Secondary malignant neoplasm of blood vessel of thigh (disorder)	94206007
SNOMED	Malignant lymphoma - small cleaved cell (disorder)	188675007
SNOMED	Primary malignant neoplasm of peripheral nerves of thorax (disorder)	109941002
SNOMED	Primary malignant neoplasm of ulna (disorder)	94112009
SNOMED	Myeloproliferative disorder (disorder)	425333006
SNOMED	Hodgkins granuloma of intra-abdominal lymph nodes (disorder)	188536008
SNOMED	Nodular lymphoma of lymph nodes of axilla and upper limb (disorder)	188612002
SNOMED	Secondary malignant neoplasm of muscle of buttock (disorder)	94419008
SNOMED	Secondary malignant neoplasm of round ligament of uterus (disorder)	94525009
SNOMED	Nodular lymphoma of lymph nodes of inguinal region and lower limb (disorder)	188613007
SNOMED	Syringotropic mycosis fungoides (disorder)	404118008
SNOMED	Malignant fibrohistiocytic tumor of skin (disorder)	402873007
SNOMED	Primary malignant neoplasm of Waldeyers ring (disorder)	94144008
SNOMED	Hodgkins disease nodular sclerosis - lymphocytic predominance (disorder)	277610002
SNOMED	Hodgkin lymphoma lymphocyte-rich (disorder)	118607005
SNOMED	Primary malignant neoplasm of anterior aspect of epiglottis (disorder)	93670003
SNOMED	Follicular non-Hodgkins lymphoma of intestine (disorder)	449419008
SNOMED	Secondary malignant neoplasm of prostate (disorder)	94503003
SNOMED	Primary malignant neoplasm of bone of upper limb (disorder)	93724001
SNOMED	Malignant neoplasm of rectosigmoid junction metastatic to brain (disorder)	9.6981E+13
SNOMED	Non-small cell lung cancer negative for epidermal growth factor receptor expression (disorder)	427038005
SNOMED	Carcinosarcoma of endometrium (disorder)	732201008
SNOMED	Primary adenocarcinoma of small intestine (disorder)	1.84891E+14
SNOMED	Carcinoma liver and/or biliary system (disorder)	286887005
SNOMED	Malignant tumor of abdominal part of esophagus (disorder)	187724003
SNOMED	Primary squamous cell carcinoma of chest wall (disorder)	9.1151E+13
SNOMED	Histiocytic sarcoma (disorder)	109988003
SNOMED	Non-small cell carcinoma of lung TNM stage 3 (disorder)	422968005
SNOMED	Pancoasts syndrome (disorder)	278065000
SNOMED	Malignant tumor of conjunctiva (disorder)	363463000
SNOMED	Malignant neoplasm of bone (disorder)	428281000
SNOMED	Malignant neoplasm of uterine adnexa (disorder)	428322007
SNOMED	Lymphoma of body of stomach (disorder)	448555009
SNOMED	Malignant infiltration of skin by underlying tumor (disorder)	404091004
SNOMED	Mucoepidermoid tumor of lacrimal gland (disorder)	254990009
SNOMED	Primary malignant neoplasm of fundus uteri (disorder)	109882003
SNOMED	Localized pagetoid reticulosis (disorder)	404120006

SNOMED	Nodular lymphoma of intrapelvic lymph nodes (disorder)	95187002
SNOMED	Primary malignant neoplasm of blood vessel of finger (disorder)	93695002
SNOMED	Secondary malignant neoplasm of stomach (disorder)	94606003
SNOMED	Primary malignant neoplasm of vermilion border of upper lip (disorder)	372027002
SNOMED	Malignant melanoma stage IB (finding)	9.56351E+14
SNOMED	Primary Kaposis sarcoma of oral cavity (disorder)	424779008
SNOMED	Primary osteosarcoma of pelvis (disorder)	440397000
SNOMED	Secondary malignant neoplasm of biliary tract (disorder)	94185003
SNOMED	Malignant mesothelioma of parietal peritoneum (disorder)	109854005
SNOMED	Primary malignant neoplasm of blood vessel of toe (disorder)	93711001
SNOMED	Malignant lymphoma follicular center cell cleaved (disorder)	303056000
SNOMED	Malignant neoplasm of digestive organs and peritoneum	269456006
SNOMED	Primary malignant neoplasm of soft tissues of neck (disorder)	94058008
SNOMED	Malignant melanoma of temple (disorder)	188042000
SNOMED	Squamous cell carcinoma of left lung (disorder)	1.2241E+16
SNOMED	Metastasis to head and neck lymph node (disorder)	303194003
SNOMED	Lymphoid leukemia in remission (disorder)	93169003
SNOMED	Secondary malignant neoplasm of blood vessel of hand (disorder)	94195005
SNOMED	Malignant neoplasm of right upper lobe of lung (disorder)	724060008
SNOMED	Primary malignant neoplasm of muscle of abdomen (disorder)	93899005
SNOMED	Primary malignant neoplasm of bone of right upper limb (disorder)	3.54671E+14
SNOMED	Malignant neoplasm of scalp AND/OR skin of neck (disorder)	188102008
SNOMED	Malignant lymphoma (disorder)	118600007
SNOMED	Megakaryocytic leukemia (disorder)	188754005
SNOMED	Acute eosinophilic leukemia (disorder)	277604002
SNOMED	Chronic myeloid leukemia in remission (disorder)	92817004
SNOMED	Germline BRCA-mutated human epidermal growth factor receptor 2 negative metastatic carcinoma of breast (disorder)	767444009
SNOMED	Malignant tumor involving rectum by direct extension from endometrium (disorder)	369448007
SNOMED	Medulloblastoma of cerebellum (disorder)	277505007
SNOMED	Invasive vulval Pagets disease (disorder)	423829008
SNOMED	Diffuse non-Hodgkins lymphoma of extranodal site (disorder)	448560008
SNOMED	Pseudomyxoma peritonei (disorder)	307601000
SNOMED	Aggressive lymphadenopathic mastocytosis with eosinophilia (disorder)	397008008
SNOMED	Seminoma of undescended testis (disorder)	313428008
SNOMED	Carcinoma of breast with ductal and lobular features (disorder)	444604002
SNOMED	Malignant neoplasm of labia majora (disorder)	363446004
SNOMED	Hodgkins disease lymphocytic depletion of lymph nodes of axilla and upper limb (disorder)	188589009
SNOMED	Primary hepatic neuroendocrine carcinoma (disorder)	716652006
SNOMED	Secondary malignant neoplasm of floor of mouth (disorder)	94304008
SNOMED	Meningeal leukemia (disorder)	722795004
SNOMED	Overlapping malignant neoplasm of female genital organs (disorder)	109878000
SNOMED	Secondary malignant neoplasm of craniopharyngeal duct (disorder)	94267001
SNOMED	Hepatosplenic T-cell lymphoma (disorder)	445406001
SNOMED	Acute myeloid leukemia with myelodysplasia-related changes (disorder)	445448008
SNOMED	Hodgkins granuloma of lymph nodes of inguinal region and lower limb (disorder)	188538009
SNOMED	Primary mucinous adenocarcinoma of appendix (disorder)	721672004
SNOMED	Secondary malignant neoplasm of nervous system (disorder)	94442001
SNOMED	Secondary malignant neoplasm of right upper lobe of lung (disorder)	94524008
SNOMED	Malignant melanoma of nail apparatus (disorder)	403927001
SNOMED	Malignant histiocytosis (disorder)	118612006
SNOMED	Secondary malignant neoplasm of inner aspect of lower lip (disorder)	94342002
SNOMED	Malignant tumor of lateral floor of mouth (disorder)	187653008

SNOMED	Primary malignant neoplasm of pyriform sinus (disorder)	93978008
SNOMED	Secondary malignant neoplasm of phalanx of hand (disorder)	94487002
SNOMED	Malignant carcinoid tumor of colon (disorder)	726654006
SNOMED	Periosteal osteosarcoma of jaw (disorder)	708504008
SNOMED	Primary malignant neoplasm of lacrimal drainage system (disorder)	423195009
SNOMED	Lymphosarcoma of intrapelvic lymph nodes (disorder)	188505000
SNOMED	Metastasis from malignant tumor of esophagus (disorder)	315003007
SNOMED	Leiomyosarcoma of scalp (disorder)	447706001
SNOMED	Secondary malignant neoplasm of blood vessel of forearm (disorder)	94194009
SNOMED	Primary malignant neoplasm of central nervous system (disorder)	93744007
SNOMED	Malignant tumor of craniopharyngeal duct (disorder)	188340000
SNOMED	Malignant melanoma of skin of anus (disorder)	447712006
SNOMED	Carcinoma of bladder (disorder)	255108000
SNOMED	Malignant neoplasm of soft tissue (disorder)	269469005
SNOMED	Secondary malignant neoplasm of eustachian tube (disorder)	94289002
SNOMED	Malignant tumor of pylorus (disorder)	187736009
SNOMED	Malignant epithelial neoplasm of female breast (disorder)	447782002
SNOMED	Malignant neoplasm of corpus uteri excluding isthmus (disorder)	188189001
SNOMED	Sarcoma of axillary tail of female breast (disorder)	447784001
SNOMED	Malignant neoplasm of foot and ankle (disorder)	8.82791E+14
SNOMED	Primary mixed subtype adenocarcinoma of lung (disorder)	707404008
SNOMED	Primary malignant neoplasm of lower inner quadrant of female breast (disorder)	93874009
SNOMED	Malignant melanoma of lower limb (disorder)	269581007
SNOMED	Primary squamous cell carcinoma of glottis (disorder)	707664003
SNOMED	Carcinoma of endocervix (disorder)	372098004
SNOMED	Carcinoma of larynx (disorder)	276975007
SNOMED	Psoralen and long-wave ultraviolet radiation therapy-associated skin malignancy (disorder)	403711001
SNOMED	Sarcoma of pelvis (disorder)	447885009
SNOMED	Adenocarcinoma of anorectum (disorder)	447886005
SNOMED	Leukemic infiltration of skin in hairy-cell leukemia (disorder)	404139001
SNOMED	Malignant tumor of optic nerve and sheath (disorder)	254972008
SNOMED	Secondary malignant neoplasm of blood vessel of finger (disorder)	94192008
SNOMED	Merkel cell carcinoma of upper limb (disorder)	1.33871E+14
SNOMED	Malignant neoplasm of cerebral cortex (disorder)	188282004
SNOMED	Malignant tumor involving vagina by direct extension from bladder (disorder)	369504000
SNOMED	Refractory anemia with excess blasts in transformation (disorder)	110000005
SNOMED	Malignant glioma of spinal cord (disorder)	276827001
SNOMED	Primary malignant neoplasm of distal bile duct (disorder)	447416008
SNOMED	Leukemic infiltration of orbit (disorder)	423032007
SNOMED	Malignant neoplasm of male breast (disorder)	372095001
SNOMED	Primary malignant neoplasm of axilla (disorder)	93684008
SNOMED	Secondary malignant neoplasm of upper third of esophagus (disorder)	94657004
SNOMED	Sarcoma of fibula (disorder)	448221005
SNOMED	Primary effusion lymphoma co-occurrent with infection caused by Human herpesvirus 8 (disorder)	714463003
SNOMED	High grade T-cell lymphoma (disorder)	277643003
SNOMED	Secondary malignant neoplasm of adenoid (disorder)	94158005
SNOMED	Primary cutaneous lymphoma (disorder)	400001003
SNOMED	Sclerosing liposarcoma (disorder)	404068003
SNOMED	Malignant neoplasm of axillary tail of female breast (disorder)	188156001
SNOMED	Secondary malignant neoplasm of trigone of urinary bladder (disorder)	94647000
SNOMED	Malignant melanoma of right choroid (disorder)	1.08098E+15
SNOMED	T-cell lymphoma (disorder)	109978004

SNOMED	Lymphoma of lesser curvature of stomach (disorder)	448269008
SNOMED	Malignant tumor of anterior floor of mouth (disorder)	187652003
SNOMED	Malignant tumor of commissure of lip (disorder)	363374005
SNOMED	Dermatofibrosarcoma protuberans of skin of chest (disorder)	448296006
SNOMED	Primary squamous cell carcinoma of maxillary sinus (disorder)	707354003
SNOMED	Chronic eosinophilic leukemia (disorder)	188733003
SNOMED	Primary malignant neoplasm of bone marrow (disorder)	93720005
SNOMED	Primary malignant neoplasm of cervical vertebral column (disorder)	93751003
SNOMED	Primary malignant neoplasm of retrocecal tissue (disorder)	372007003
SNOMED	Secondary malignant neoplasm of superior wall of nasopharynx (disorder)	94613003
SNOMED	Non-Hodgkins lymphoma of intestine (disorder)	448354009
SNOMED	Carcinoma of epididymis/spermatic cord (disorder)	286900008
SNOMED	Cutaneous/peripheral T-cell lymphoma (disorder)	277613000
SNOMED	Non-Hodgkins lymphoma of ovary (disorder)	448376000
SNOMED	Primary malignant neoplasm of lateral wall of nasopharynx (disorder)	93861003
SNOMED	Sarcoma of bone of foot (disorder)	448378004
SNOMED	Malignant tumor of head and/or neck (disorder)	255056009
SNOMED	Overlapping malignant neoplasm of heart mediastinum and pleura (disorder)	109384006
SNOMED	Rhabdomyosarcoma of orbit (disorder)	254994000
SNOMED	Sarcoma of lower outer quadrant of female breast (disorder)	448388003
SNOMED	Secondary malignant neoplasm of intercostal lymph nodes (disorder)	94344001
SNOMED	Malignant tumor involving right ovary by direct extension from vagina (disorder)	369567009
SNOMED	Primary signet ring cell carcinoma of lung (disorder)	707407001
SNOMED	Metastatic malignant neoplasm of meninges (disorder)	722671009
SNOMED	Carcinoma of glottis (disorder)	372103002
SNOMED	Primary squamous cell carcinoma of hypopharyngeal aspect of aryepiglottic fold (disorder)	707697002
SNOMED	Primary malignant neoplasm of shoulder (disorder)	94005003
SNOMED	Secondary malignant neoplasm of sacrococcygeal region (disorder)	94526005
SNOMED	Hodgkins granuloma (disorder)	118602004
SNOMED	Primary malignant neoplasm of bone of skull (disorder)	93723007
SNOMED	Malignant neoplasm of multiple endocrine glands (disorder)	1.09093E+15
SNOMED	Lymphoma of pelvis (disorder)	448553002
SNOMED	Malignant neoplasm of parietal lobe (disorder)	363469001
SNOMED	Malignant histiocytosis of lymph nodes of multiple sites (disorder)	93188005
SNOMED	Primary squamous cell carcinoma of lower third of esophagus (disorder)	721620009
SNOMED	Secondary malignant neoplasm of ileum (disorder)	94335002
SNOMED	Epstein-Barr virus positive diffuse large B-cell lymphoma of elderly (disorder)	716788007
SNOMED	Secondary malignant neoplasm of parotid lymph nodes (disorder)	94475009
SNOMED	Primary squamous cell carcinoma of supraglottis (disorder)	707575007
SNOMED	Granulomatous mycosis fungoides (disorder)	404112009
SNOMED	Local recurrence of malignant tumor of stomach (disorder)	314961003
SNOMED	Malignant tumor of jejunum (disorder)	363404008
SNOMED	Lymphosarcoma (disorder)	188498009
SNOMED	Secondary malignant neoplasm of short bone of lower limb (disorder)	94535003
SNOMED	Secondary malignant neoplasm of pyriform sinus (disorder)	94507002
SNOMED	Adenocarcinoma of gallbladder and extrahepatic biliary tract (disorder)	765741003
SNOMED	Sarcoma of dendritic cells (accessory cells) (disorder)	446643000
SNOMED	Diffuse non-Hodgkins lymphoma of nasopharynx (disorder)	448319002
SNOMED	Adenocarcinoma of duodenum (disorder)	408644002
SNOMED	Mixed epithelial tumor of ovary (disorder)	254855000
SNOMED	Primary liposarcoma of retroperitoneum (disorder)	722515001
SNOMED	Kappa light chain myeloma (disorder)	414553000

SNOMED	Secondary malignant neoplasm of deep inguinal lymph nodes (disorder)	1.0902E+15
SNOMED	Primary adenocarcinoma overlapping lesion of retroperitoneum peritoneum and omentum (disorder)	721562005
SNOMED	Primary malignant neoplasm of pelvic bone (disorder)	93951006
SNOMED	Sarcoma of radius (disorder)	448775003
SNOMED	Primary malignant neoplasm of cerebellum (disorder)	93746009
SNOMED	Richters syndrome (disorder)	277550009
SNOMED	Malignant histiocytosis of lymph nodes of head face AND/OR neck (disorder)	93186009
SNOMED	Primary malignant neoplasm of blood vessel of buttock (disorder)	93693009
SNOMED	Malignant melanoma of lower limb and hip (disorder)	188067002
SNOMED	Diffuse non-Hodgkins lymphoma of lung (disorder)	448867004
SNOMED	Malignant neoplasm of lateral wall of oropharynx (disorder)	448868009
SNOMED	Primary malignant neoplasm of branchial cleft (disorder)	109834009
SNOMED	Secondary malignant neoplasm of skin of cheek (disorder)	94546000
SNOMED	Fibrosarcoma of connective tissue (disorder)	448139005
SNOMED	Primary malignant neoplasm of cystic duct (disorder)	93770008
SNOMED	Carcinomatosis of peritoneal cavity (disorder)	255121007
SNOMED	Primary malignant neoplasm of salivary gland duct (disorder)	109828002
SNOMED	Primary malignant neoplasm of pancreatic duct (disorder)	93939009
SNOMED	Malignant neoplasm of thoracic cavity structure (disorder)	428100006
SNOMED	Hodgkins disease lymphocytic predominance - diffuse (disorder)	277609007
SNOMED	Primary malignant neoplasm of false vocal cord (disorder)	371988005
SNOMED	Primary malignant neoplasm of ovary (disorder)	93934004
SNOMED	Primary neuroendocrine carcinoma of cardia of stomach (disorder)	721639004
SNOMED	Malignant tumor involving right ovary by direct extension from fallopian tube (disorder)	369532009
SNOMED	Malignant tumor involving uterine corpus by direct extension from ovary (disorder)	369494005
SNOMED	Secondary malignant neoplasm of central nervous system (disorder)	94243009
SNOMED	Primary cutaneous gamma-delta-positive T-cell lymphoma (disorder)	733627006
SNOMED	Secondary malignant neoplasm of muscle of thorax (disorder)	94429001
SNOMED	Acute lymphoid leukemia in remission (disorder)	91856007
SNOMED	Follicular non-Hodgkins lymphoma of uterine cervix (disorder)	449059000
SNOMED	Primary malignant neoplasm of ill-defined site (disorder)	109357005
SNOMED	Malignant tumor of aryepiglottic fold - laryngeal aspect (disorder)	363487003
SNOMED	Primary malignant neoplasm of sphenoid bone (disorder)	94066004
SNOMED	Secondary malignant neoplasm of body of uterus (disorder)	94215000
SNOMED	Carcinoma of lateral part of floor of mouth (disorder)	254431000
SNOMED	Malignant neoplasm of genitourinary organ (disorder)	271468000
SNOMED	Primary adenocarcinoma of vagina (disorder)	9.9121E+13
SNOMED	Secondary malignant neoplasm of lower gum (disorder)	94384005
SNOMED	Low grade B-cell lymphoma (disorder)	277615007
SNOMED	Malignant neoplasm of respiratory system (disorder)	449096009
SNOMED	Sarcoma of bone of pelvis (disorder)	449100005
SNOMED	Mycosis fungoides of extranodal AND/OR solid organ site (disorder)	94715001
SNOMED	Philadelphia chromosome positive chronic myelogenous leukemia (disorder)	449108003
SNOMED	Secondary malignant neoplasm of pituitary gland (disorder)	94491007
SNOMED	Malignant epithelial neoplasm of floor of mouth (disorder)	449156009
SNOMED	Secondary malignant neoplasm of epicardium (disorder)	94282006
SNOMED	Primary malignant neoplasm of bronchus of right upper lobe (disorder)	93733004
SNOMED	Local recurrence of malignant tumor of kidney (disorder)	314967004
SNOMED	Secondary malignant neoplasm of glans penis (disorder)	94315003
SNOMED	Malignant tumor involving uterine cervix by separate metastasis from fallopian tube (disorder)	369500009
SNOMED	Secondary malignant neoplasm of uterine adnexa (disorder)	94664002
SNOMED	Diffuse non-Hodgkins lymphoma of soft tissue (disorder)	449216004

SNOMED	Heavy chain disease (disorder)	68979007
SNOMED	Primary malignant neoplasm of pyloric antrum (disorder)	93976007
SNOMED	Follicular non-Hodgkins lymphoma of nasopharynx (disorder)	449219006
SNOMED	Squamous cell carcinoma of vulva (disorder)	254895003
SNOMED	Primary malignant neoplasm of upper limb (disorder)	94116007
SNOMED	Malignant neoplasm of medulla oblongata (disorder)	188296005
SNOMED	Intermediate grade B-cell lymphoma (disorder)	285776004
SNOMED	Follicular non-Hodgkins lymphoma of prostate (disorder)	448217003
SNOMED	Carcinoma of esophagus (disorder)	372138000
SNOMED	Primary malignant neoplasm of blood vessel of shoulder (disorder)	93708002
SNOMED	Immunoglobulin G myeloma (disorder)	285421005
SNOMED	Squamous cell carcinoma of oropharynx (disorder)	423464009
SNOMED	Acute myeloid leukemia without maturation FAB M1 (disorder)	359640008
SNOMED	Sarcoma of mandible (disorder)	449267001
SNOMED	Primary malignant neoplasm of upper lobe of left lung (disorder)	93865007
SNOMED	Primary Ewing sarcoma of articular cartilage of rib (disorder)	723856003
SNOMED	Non-Hodgkins lymphoma of tonsil (disorder)	449292003
SNOMED	Lymphangitis carcinomatosa (disorder)	255119002
SNOMED	Carcinosarcoma of ovary (disorder)	702368000
SNOMED	Primary malignant neoplasm of blood vessel of popliteal space (disorder)	93707007
SNOMED	Malignant tumor of cardia (disorder)	187732006
SNOMED	Primary osteosarcoma of bone of limb (disorder)	723850009
SNOMED	Hodgkins disease nodular sclerosis of intrapelvic lymph nodes (disorder)	188570009
SNOMED	Cutaneous leiomyosarcoma (disorder)	254771006
SNOMED	Malignant tumor involving bladder by direct extension from uterine cervix (disorder)	369473007
SNOMED	Malignant tumor involving rectum by separate metastasis from endometrium (disorder)	369455009
SNOMED	Carcinoma of stomach (disorder)	372143007
SNOMED	Malignant epithelial neoplasm of body of uterus (disorder)	449073009
SNOMED	Secondary malignant neoplasm of tracheobronchial lymph nodes (disorder)	94642006
SNOMED	Malignant histiocytosis of lymph nodes of axilla AND/OR upper limb (disorder)	93185008
SNOMED	Secondary malignant neoplasm of muscle of neck (disorder)	94425007
SNOMED	Secondary malignant neoplasm of epididymis AND vas deferens (disorder)	188471005
SNOMED	Malignant tumor of duodenum (disorder)	363403002
SNOMED	Secondary malignant neoplasm of blood vessel of foot (disorder)	94193003
SNOMED	Malignant melanoma of buccal mucosa (disorder)	698045009
SNOMED	Secondary malignant neoplasm of skin of lower leg (disorder)	449630001
SNOMED	Malignant tumor of Eustachian tube (disorder)	187834000
SNOMED	SÃƒ Â©zarys disease of spleen (disorder)	95263006
SNOMED	Malignant tumor of adrenal gland (disorder)	363355002
SNOMED	Follicular non-Hodgkins lymphoma of central nervous system (disorder)	448995000
SNOMED	Letterer-Siwe disease of intrathoracic lymph nodes (disorder)	93135004
SNOMED	Combined hepatocellular carcinoma and cholangiocarcinoma (disorder)	274902006
SNOMED	Overlapping malignant neoplasm of mouth (disorder)	1.09283E+15
SNOMED	Hodgkins paragranuloma of intra-abdominal lymph nodes (disorder)	188526000
SNOMED	Primary myxofibrosarcoma (disorder)	723076008
SNOMED	Primary malignant neoplasm of central portion of female breast (disorder)	93745008
SNOMED	Leukemic reticuloendotheliosis of extranodal AND/OR solid organ site (disorder)	93152000
SNOMED	Secondary malignant neoplasm of broad ligament (disorder)	94226006
SNOMED	Secondary malignant neoplasm of spermatic cord (disorder)	94597008
SNOMED	Malignant neoplasm of lower lobe of left lung (disorder)	724059003
SNOMED	Oligodendroglioma (disorder)	443936004
SNOMED	Primary malignant neoplasm of exocervix (disorder)	93789008

SNOMED	Malignant tumor of fallopian tube (disorder)	363444001
SNOMED	Primary malignant neoplasm of anal canal (disorder)	93669004
SNOMED	Nonkeratinizing carcinoma of the nasopharynx (disorder)	707705008
SNOMED	Extensive stage primary small cell carcinoma of lung (disorder)	6.83991E+14
SNOMED	Primary small cell malignant neoplasm of lung TNM stage 2 (disorder)	6.7821E+13
SNOMED	Malignant tumor involving bladder by direct extension from uterus (disorder)	369474001
SNOMED	Primary malignant neoplasm of upper limb bones and scapula (disorder)	372133009
SNOMED	Diffuse large B-cell lymphoma co-occurrent with chronic inflammation caused by Epstein-Barr virus (disorder)	724647003
SNOMED	Infiltrating ductal carcinoma of central portion of left female breast (disorder)	1.08011E+15
SNOMED	Primary malignant neoplasm of sublingual gland (disorder)	94076001
SNOMED	Malignant tumor of adenoid (disorder)	187694000
SNOMED	Diffuse non-Hodgkins lymphoma of skin (disorder)	449217008
SNOMED	Primary malignant neoplasm of both ovaries (disorder)	1.56357E+16
SNOMED	Malignant melanoma of gum (disorder)	698287002
SNOMED	Primary malignant neoplasm of inner aspect of lip (disorder)	93835002
SNOMED	Malignant neoplasm of connective and soft tissue of thumb (disorder)	187997005
SNOMED	Primary malignant neoplasm of appendix (disorder)	93679002
SNOMED	Keratinizing squamous cell carcinoma of nasopharynx (disorder)	698011002
SNOMED	Pleuropulmonary blastoma type II (disorder)	707672001
SNOMED	Malignant melanoma of vestibule of mouth (disorder)	698041000
SNOMED	Malignant melanoma of tongue (disorder)	698042007
SNOMED	Secondary malignant neoplasm of laryngeal surface of epiglottis (disorder)	94369001
SNOMED	Secondary malignant neoplasm of ovary (disorder)	94455000
SNOMED	Malignant melanoma of skin of upper arm (disorder)	449637003
SNOMED	Primary spindle cell carcinoma of lung (disorder)	707457009
SNOMED	Non-small cell carcinoma of lung TNM stage 4 (disorder)	423121009
SNOMED	Follicular center B-cell lymphoma (nodal/systemic with skin involvement) (disorder)	404147001
SNOMED	Malignant melanoma of sphenoidal sinus (disorder)	707362006
SNOMED	Primary malignant neoplasm of sclera primary (disorder)	93998003
SNOMED	Malignant melanoma of maxillary sinus (disorder)	698288007
SNOMED	Secondary malignant neoplasm of palatine bone (disorder)	94457008
SNOMED	Leukemic reticuloendotheliosis of lymph nodes of inguinal region and lower limb (disorder)	188649008
SNOMED	Primary malignant neoplasm of ethmoid bone (disorder)	93786001
SNOMED	Malignant tumor involving ureter by separate metastasis from bladder (disorder)	369464004
SNOMED	Pediatric follicular lymphoma (disorder)	736322001
SNOMED	Malignant cylindroma of skin (disorder)	403941005
SNOMED	Primary adenocarcinoma of fallopian tube (disorder)	9.9101E+13
SNOMED	Malignant neoplasm of exocervix (disorder)	372099007
SNOMED	Primary malignant neoplasm of body of uterus (disorder)	371971003
SNOMED	Malignant neoplasm of squamocolumnar junction of cervix (disorder)	188184006
SNOMED	Primary neuroendocrine carcinoma of pyloric antrum of stomach (disorder)	721641003
SNOMED	Secondary malignant neoplasm of uterus (disorder)	94665001
SNOMED	Familial malignant neoplasm of prostate (disorder)	715412008
SNOMED	Overlapping malignant neoplasm of female breast (disorder)	109886000
SNOMED	Subacute leukemia in remission (disorder)	427056005
SNOMED	Primary angiosarcoma of breast (disorder)	721576006
SNOMED	T-cell large granular lymphocytic leukemia (disorder)	699818003
SNOMED	Malignant tumor of lung (disorder)	363358000
SNOMED	Malignant tumor involving right ovary by separate metastasis from fallopian tube (disorder)	369569007
SNOMED	Primary malignant neoplasm of bronchus of left lower lobe (disorder)	93729006
SNOMED	Primary mixed adenocarcinoma of endometrium (disorder)	722681008
SNOMED	Generalized pagetoid reticulosis (disorder)	404121005

SNOMED	Minimal deviation malignant melanoma (disorder)	403920004
SNOMED	Malignant tumor involving urethra by direct extension from prostate (disorder)	369466002
SNOMED	Malignant lymphoma mixed lymphocytic-histiocytic nodular (disorder)	307636001
SNOMED	Primary urothelial carcinoma of overlapping lesion of urinary organ (disorder)	724468006
SNOMED	Secondary malignant neoplasm of transverse colon (disorder)	94643001
SNOMED	Secondary malignant neoplasm of hypopharyngeal aspect of aryepiglottic fold (disorder)	94332004
SNOMED	Kaposis sarcoma of soft tissue (disorder)	188029000
SNOMED	Secondary malignant neoplasm of para-aortic body (disorder)	94461002
SNOMED	Primary malignant neoplasm of talus (disorder)	94083008
SNOMED	Anaplastic large cell lymphoma T/Null cell primary systemic type (disorder)	703626001
SNOMED	Malignant melanoma stage IV M1b (finding)	9.56531E+14
SNOMED	Pancoast tumor (disorder)	254638002
SNOMED	Carcinoma of lip oral cavity and/or pharynx (disorder)	255069008
SNOMED	Carcinosarcoma of uterine adnexa (disorder)	723173003
SNOMED	Secondary malignant neoplasm of cerebrum (disorder)	94248000
SNOMED	Malignant tumor involving right fallopian tube by direct extension from uterus (disorder)	369551003
SNOMED	Malignant neoplasm of breast upper outer quadrant (disorder)	373083005
SNOMED	Malignant melanoma of chin (disorder)	188038003
SNOMED	Primary adenocarcinoma of trachea (disorder)	707475005
SNOMED	Classical Hodgkin lymphoma (disorder)	762690000
SNOMED	Metastatic malignant melanoma (disorder)	443493003
SNOMED	Malignant melanoma of skin of scrotum (disorder)	448273006
SNOMED	Malignant tumor of lipstick area of lip (disorder)	275399006
SNOMED	Secondary malignant neoplasm of anus (disorder)	94172001
SNOMED	Secondary malignant neoplasm of parietal pleura (disorder)	94473002
SNOMED	Acute myeloid leukemia and myelodysplastic syndrome related to radiation (disorder)	766048008
SNOMED	Primary bone lymphoma (disorder)	766935007
SNOMED	Primary malignant neoplasm of myocardium (disorder)	93914000
SNOMED	Primary squamous cell carcinoma of frontal sinus (disorder)	707356001
SNOMED	Primary squamous cell carcinoma of laryngeal cartilage (disorder)	707357005
SNOMED	Myeloid leukemia co-occurrent with Down syndrome (disorder)	724644005
SNOMED	Malignant tumor of vermilion border of lip (disorder)	421249001
SNOMED	Primary Ewing sarcoma of articular cartilage of limb (disorder)	723854000
SNOMED	Mycosis fungoides of lymph nodes of head face AND/OR neck (disorder)	94711005
SNOMED	Secondary malignant neoplasm of soft tissues of head (disorder)	94586008
SNOMED	Carcinoma of Bartholins gland (disorder)	276876007
SNOMED	Therapy related acute myeloid leukemia and myelodysplastic syndrome (disorder)	721306009
SNOMED	Primary adenocarcinoma of rectosigmoid junction (disorder)	1.84881E+14
SNOMED	Secondary malignant neoplasm of cardia of stomach (disorder)	94237007
SNOMED	Well-differentiated neuroendocrine carcinoma of thymus (disorder)	717922007
SNOMED	Primary cutaneous diffuse large cell B-cell lymphoma of lower extremity (disorder)	735332000
SNOMED	Secondary malignant neoplasm of muscle of shoulder (disorder)	94428009
SNOMED	Carcinoma of body of stomach (disorder)	254557000
SNOMED	Primary adenocarcinoma of jejunum (disorder)	724537009
SNOMED	Primary malignant neoplasm of maxilla (disorder)	93888008
SNOMED	Non-Hodgkins lymphoma of lung (disorder)	448372003
SNOMED	Malignant neoplasm of cartilage of nose (disorder)	187829004
SNOMED	Chronic lymphocytic prolymphocytic leukemia syndrome (disorder)	277549009
SNOMED	Malignant epithelial neoplasm of nasal cavity (disorder)	448990005
SNOMED	Ewings sarcoma of soft tissue (disorder)	447951009
SNOMED	Malignant tumor of acoustic vestibular nerve (disorder)	254980001
SNOMED	Malignant neoplasm of pelvic peritoneum (disorder)	449377002

SNOMED	Acute myelofibrosis (disorder)	109991003
SNOMED	Malignant epithelial neoplasm of renal pelvis (disorder)	448215006
SNOMED	Primary mucinous adenocarcinoma of oropharynx (disorder)	707400004
SNOMED	Malignant neoplasm of mesocecum (disorder)	187810005
SNOMED	Primary adenocarcinoma of oropharynx (disorder)	707402007
SNOMED	Primary undifferentiated carcinoma of lung (disorder)	707456000
SNOMED	Chondrosarcoma of bone (disorder)	447792005
SNOMED	Primary malignant neoplasm of orbit (disorder)	93932000
SNOMED	Malignant lymphoma - mixed small and large cell (disorder)	188676008
SNOMED	Gestational trophoblastic disease (disorder)	416402001
SNOMED	Malignant tumor of face (disorder)	363501002
SNOMED	Hodgkins granuloma of lymph nodes of axilla and upper limb (disorder)	188537004
SNOMED	Primary solid carcinoma of lung (disorder)	707410008
SNOMED	Primary malignant neoplasm of radius (disorder)	93979000
SNOMED	Malignant Triton tumor (disorder)	404040002
SNOMED	Nodular lymphoma of intrathoracic lymph nodes (disorder)	95188007
SNOMED	Primary basaloid carcinoma of larynx (disorder)	707423001
SNOMED	Primary adenocarcinoma of skin (disorder)	7.9281E+13
SNOMED	Squamous cell carcinoma of bronchus in right middle lobe (disorder)	313356004
SNOMED	Adenocarcinoma of lung (disorder)	254626006
SNOMED	Malignant tumor of rectum (disorder)	363351006
SNOMED	Squamous cell carcinoma of corpus uteri (disorder)	764737005
SNOMED	Overlapping squamous cell carcinoma of laryngeal cartilage (disorder)	707430007
SNOMED	Primary adenocarcinoma of parotid gland (disorder)	721557009
SNOMED	Primary mucinous adenocarcinoma of lung (disorder)	707452003
SNOMED	Malignant melanoma of ankle (disorder)	188073001
SNOMED	Non-seminomatous germ cell neoplasm of testis (disorder)	1.07691E+14
SNOMED	Superficial spreading malignant melanoma of skin (disorder)	254730000
SNOMED	Malignant neoplasm of colon (disorder)	363406005
SNOMED	Reticulosarcoma (disorder)	373168002
SNOMED	Malignant germ cell neoplasm of mediastinum (disorder)	713293002
SNOMED	Malignant neoplasm of connective and soft tissue of hip and lower limb (disorder)	187999008
SNOMED	Primary adenosquamous carcinoma of oropharynx (disorder)	707583001
SNOMED	Secondary malignant neoplasm of adrenal cortex (disorder)	94160007
SNOMED	Malignant neoplasm of peripheral nerve of abdomen (disorder)	188326001
SNOMED	Malignant tumor involving an organ by direct extension from fallopian tube (disorder)	369596005
SNOMED	Primary adenocarcinoma of head of pancreas (disorder)	6.81721E+14
SNOMED	Squamous cell carcinoma of bronchus in left upper lobe (disorder)	313354001
SNOMED	Primary malignant neoplasm of parathyroid gland (disorder)	93943008
SNOMED	Endometrioid carcinoma ovary (disorder)	254852002
SNOMED	Secondary malignant neoplasm of pineal gland (disorder)	94489004
SNOMED	Primary mucinous adenocarcinoma of trachea (disorder)	707473003
SNOMED	Primary malignant neoplasm of broad ligament (disorder)	93728003
SNOMED	Malignant tumor involving vulva by separate metastasis from uterus (disorder)	369592007
SNOMED	Primary carcinosarcoma of lung (disorder)	707596000
SNOMED	Primary lymphoepithelial carcinoma of oropharynx (disorder)	707584007
SNOMED	Primary undifferentiated carcinoma of hypopharynx (disorder)	707483004
SNOMED	Sarcoma of humerus (disorder)	449207003
SNOMED	Primary squamous cell carcinoma of pharynx (disorder)	733345003
SNOMED	Malignant melanoma of skin of left lower limb (disorder)	1.08102E+15
SNOMED	Malignant tumor of tonsillar fossa (disorder)	363394001
SNOMED	Primary spindle cell squamous cell carcinoma of hypopharynx (disorder)	707489000

SNOMED	Hodgkins disease mixed cellularity of spleen (disorder)	93509007
SNOMED	Metastatic malignant melanoma with diffuse hypermelanosis (disorder)	402563000
SNOMED	Primary squamous cell carcinoma of hypopharynx (disorder)	707492001
SNOMED	Primary lymphoepithelial carcinoma of trachea (disorder)	707493006
SNOMED	Malignant tumor of anorectal junction (disorder)	254586002
SNOMED	Primary squamous cell adenoid carcinoma of trachea (disorder)	707495004
SNOMED	Primary malignant neoplasm of vagina (disorder)	372025005
SNOMED	Pigmented dermatofibrosarcoma protuberans of skin (disorder)	398670003
SNOMED	Lymphosarcoma of intrathoracic lymph nodes (disorder)	188501009
SNOMED	Secondary malignant neoplasm of bone of upper limb (disorder)	94221001
SNOMED	Malignant tumor of lip (disorder)	363348004
SNOMED	Primary malignant neoplasm of lacrimal gland duct (disorder)	423005002
SNOMED	Primary squamous cell carcinoma of vallecula (disorder)	707538002
SNOMED	Familial malignant neoplasm of pancreas (disorder)	715414009
SNOMED	Malignant melanoma of skin of neck (disorder)	93642000
SNOMED	Malignant tumor involving rectum by direct extension from prostate (disorder)	369451000
SNOMED	Secondary malignant neoplasm of abducens nerve (disorder)	94153001
SNOMED	Acquired cystic disease associated renal cell carcinoma (disorder)	764856008
SNOMED	Malignant tumor of nerve sheath origin (disorder)	402876004
SNOMED	Diffuse sclerosing papillary thyroid carcinoma (disorder)	708971008
SNOMED	Metastasis from malignant tumor of thyroid (disorder)	315007008
SNOMED	Malignant neoplasm of bronchus or lung (disorder)	1.09088E+15
SNOMED	Primary malignant neoplasm of thigh (disorder)	94093001
SNOMED	Diffuse non-Hodgkins lymphoma of bone (disorder)	449177007
SNOMED	Malignant tumor of retina (disorder)	363465007
SNOMED	Malignant melanoma stage IV M1a (finding)	9.56511E+14
SNOMED	Secondary malignant neoplasm of inguinal region (disorder)	94340005
SNOMED	Primary acinar cell carcinoma of oropharynx (disorder)	707590006
SNOMED	Primary mucoepidermoid carcinoma of oropharynx (disorder)	707591005
SNOMED	Peripheral T-cell lymphoma - pleomorphic medium and large cell (disorder)	277653002
SNOMED	Hodgkins disease nodular sclerosis - lymphocytic depletion (disorder)	277612005
SNOMED	Malignant melanoma of scalp (disorder)	188045003
SNOMED	Multiple myeloma in remission (disorder)	94704006
SNOMED	Primary salivary gland type carcinoma of hypopharynx (disorder)	707627009
SNOMED	Metastasis to heart of unknown primary (disorder)	285606005
SNOMED	Burkitts lymphoma of intra-abdominal lymph nodes (disorder)	188512009
SNOMED	Primary malignant neoplasm of perihilar bile duct (disorder)	446807009
SNOMED	Malignant tumor involving left ovary by separate metastasis from fallopian tube (disorder)	369561005
SNOMED	Squamous cell carcinomatosis (disorder)	425303004
SNOMED	Metastasis to liver of unknown primary (disorder)	285613005
SNOMED	Malignant neoplasm of connective and soft tissue of upper limb and shoulder (disorder)	187991006
SNOMED	Primary adenosquamous carcinoma of trachea (disorder)	707393007
SNOMED	Primary malignant epithelial neoplasm of trachea (disorder)	707674000
SNOMED	Secondary malignant neoplasm of cystic duct (disorder)	94270002
SNOMED	Malignant tumor involving left ovary by direct extension from vagina (disorder)	369559001
SNOMED	Primary mucinous cystic neoplasm with associated invasive carcinoma of distal bile duct (disorder)	733349009
SNOMED	Secondary malignant neoplasm of accessory nerve (disorder)	94154007
SNOMED	Malignant tumor of tonsillar pillar (disorder)	187675005
SNOMED	Secondary malignant neoplasm of anterior wall of nasopharynx (disorder)	94170009
SNOMED	Malignant tumor of exocrine pancreas (disorder)	255088001
SNOMED	[X]Malignant neoplasm of female genital organs (disorder)	4.62461E+14
SNOMED	Non-Hodgkins lymphoma of nose (disorder)	448384001

SNOMED	Secondary malignant neoplasm of ethmoid bone (disorder)	94287000
SNOMED	Malignant melanoma stage IIIB (finding)	9.56451E+14
SNOMED	Overlapping malignant neoplasm of brain and other parts of the central nervous system (disorder)	109911004
SNOMED	Subacute lymphoid leukemia (disorder)	188726003
SNOMED	Dermatofibrosarcoma protuberans with granular cell change (disorder)	404009009
SNOMED	Primary malignant neoplasm of metacarpal bone (disorder)	93892001
SNOMED	Primary malignant neoplasm of soft tissues (disorder)	372010005
SNOMED	Primary malignant neoplasm of olfactory nerve (disorder)	93930008
SNOMED	Malignant melanoma (disorder)	372244006
SNOMED	Diffuse high grade B-cell lymphoma (disorder)	277626001
SNOMED	Malignant neoplasm of chest wall (disorder)	712750007
SNOMED	Carcinoma of urinary bladder invasive (disorder)	425066001
SNOMED	Primary malignant neoplasm of stomach (disorder)	372014001
SNOMED	Primary adenocarcinoma of anus (disorder)	1.84741E+14
SNOMED	Primary malignant neoplasm of acoustic nerve (disorder)	93660000
SNOMED	Malignant neoplasm of head neck and face (disorder)	188353002
SNOMED	Primary cerebral lymphoma co-occurrent with human immunodeficiency virus infection (disorder)	713325002
SNOMED	Malignant meningioma of meninges of brain (disorder)	713327005
SNOMED	Malignant neoplasm of fifth metacarpal bone (disorder)	187949009
SNOMED	Invasive carcinoma of breast (disorder)	713609000
SNOMED	Primary carcinoma of frontal sinus (disorder)	707349007
SNOMED	Secondary malignant neoplasm of soft tissues of face (disorder)	94585007
SNOMED	Primary adenocarcinoma of ileum (disorder)	724536000
SNOMED	[X]Malignant neoplasm of eye brain and other parts of central nervous system (disorder)	3.97141E+14
SNOMED	Malignant neoplasm of second metatarsal bone (disorder)	187974007
SNOMED	Diffuse non-Hodgkin immunoblastic lymphoma co-occurrent with human immunodeficiency virus infection (disorder)	713718006
SNOMED	Burkitt lymphoma co-occurrent with human immunodeficiency virus infection (disorder)	713897006
SNOMED	Somatostatinoma (disorder)	253006001
SNOMED	Primary malignant neoplasm of muscle of perineum (disorder)	93908007
SNOMED	Mast cell malignancy of lymph nodes of multiple sites (disorder)	188669003
SNOMED	Primary adenoid cystic carcinoma of hypopharynx (disorder)	707539005
SNOMED	Primary malignant neoplasm of bronchus of right lower lobe (disorder)	93731002
SNOMED	Ependymoblastoma (disorder)	715901002
SNOMED	Primary malignant neoplasm of apex of urinary bladder (disorder)	371969003
SNOMED	Primary myoepithelial carcinoma of lung (disorder)	707464006
SNOMED	Letterer-Siwe disease of intra-abdominal lymph nodes (disorder)	93133006
SNOMED	Primary non-gestational choriocarcinoma of ovary (disorder)	716588005
SNOMED	Primary adenocarcinoma of gallbladder (disorder)	9.541E+12
SNOMED	Mixed oligoastrocytoma (disorder)	716647001
SNOMED	Bowel cancer detected by national screening programme (disorder)	2.94301E+14
SNOMED	Primary angiosarcoma of heart (disorder)	721575005
SNOMED	Hodgkins disease lymphocytic-histiocytic predominance of intrapelvic lymph nodes (disorder)	93494007
SNOMED	Primary malignant neoplasm of upper third of esophagus (disorder)	372023003
SNOMED	Primary malignant neuroendocrine neoplasm of pyloric antrum of stomach (disorder)	721637002
SNOMED	Mast cell malignancy of intra-abdominal lymph nodes (disorder)	188664008
SNOMED	Indolent systemic mastocytosis (disorder)	70910003
SNOMED	Malignant neoplasm of ectopic site of female breast (disorder)	188159008
SNOMED	Secondary malignant neoplasm of buccal mucosa (disorder)	94234000
SNOMED	Primary malignant neoplasm of lunate bone (disorder)	93879004
SNOMED	Malignant neoplasm of posterior mediastinum (disorder)	448670003
SNOMED	Malignant tumor of seminal vesicle (disorder)	188234005
SNOMED	Primary malignant neoplasm of blood vessel of lower leg (disorder)	400074007

SNOMED	Secondary malignant neoplasm of blood vessel (disorder)	94211009
SNOMED	Primary adenocarcinoma of overlapping lesion of stomach (disorder)	721633003
SNOMED	Refractory anemia with excess blasts-2 (disorder)	415284008
SNOMED	Melanoma and neural system tumor syndrome (disorder)	717968005
SNOMED	Primary malignant neuroendocrine neoplasm of cardia of stomach (disorder)	721635005
SNOMED	Primary acinar cell carcinoma of lung (disorder)	707409003
SNOMED	Small cell neuroendocrine carcinoma of bladder (disorder)	718604008
SNOMED	Secondary malignant neoplasm of skin of forehead (disorder)	94558000
SNOMED	Secondary malignant neoplasm of bronchus of left lower lobe (disorder)	94228007
SNOMED	Malignant tumor involving bladder by separate metastasis from prostate (disorder)	369479006
SNOMED	Refractory thrombocytopenia (disorder)	721304007
SNOMED	Adenocarcinoma of large intestine (disorder)	408645001
SNOMED	Malignant tumor of hypothalamus (disorder)	188286001
SNOMED	Acute leukemia of ambiguous lineage (disorder)	721308005
SNOMED	Secondary malignant neoplasm of soft tissues of thorax (disorder)	94594001
SNOMED	Primary malignant neoplasm of alveolar ridge mucosa (disorder)	93667002
SNOMED	Secondary malignant neoplasm of cecum (disorder)	94235004
SNOMED	Malignant epithelial neoplasm of thyroid (disorder)	448216007
SNOMED	Malignant tumor involving an organ by direct extension from bladder (disorder)	369594008
SNOMED	Secondary malignant neoplasm of coccygeal body (disorder)	94258001
SNOMED	Primary adenocarcinoma of ciliary epithelium (disorder)	721546004
SNOMED	Primary adenocarcinoma of epithelium of iris (disorder)	721547008
SNOMED	Carcinoma of ribs and/or sternum and/or clavicle (disorder)	372121004
SNOMED	Primary adenocarcinoma of lacrimal apparatus (disorder)	721549006
SNOMED	Follicular lymphoma of small intestine (disorder)	721555001
SNOMED	B-cell lymphoma unclassifiable with features intermediate between classical Hodgkin lymphoma and diffuse large B-cell lymphoma (disorder)	722954005
SNOMED	Primary malignant neoplasm of carina (disorder)	93739000
SNOMED	Malignant neoplasm of metatarsal bone of foot (disorder)	405945003
SNOMED	Secondary malignant neoplasm of thoracic esophagus (disorder)	94631002
SNOMED	Clinical stage B chronic lymphocytic leukaemia (disorder)	8.63761E+14
SNOMED	WaldenstrÃƒ Â¶m macroglobulinemia (disorder)	190818004
SNOMED	Sarcoma of bone and connective tissue (disorder)	255067005
SNOMED	Primary malignant neoplasm of thyroid gland metastatic to bone (disorder)	424887002
SNOMED	Secondary neoplasm of left broad ligament (disorder)	369535006
SNOMED	Primary malignant neoplasm of trapezoid bone (disorder)	94107008
SNOMED	Malignant neoplasm overlapping lesion of breast (disorder)	188157005
SNOMED	Alpha heavy chain disease (disorder)	109982002
SNOMED	Extraovarian primary peritoneal carcinoma (disorder)	716649003
SNOMED	Primary adenocarcinoma of subglottis (disorder)	707479004
SNOMED	Carcinoma of midline of tongue (disorder)	275397008
SNOMED	Primary undifferentiated carcinoma of endometrium (disorder)	733360000
SNOMED	Primary malignant neoplasm of urinary bladder neck (disorder)	94124002
SNOMED	Primary myosarcoma of omentum (disorder)	721580001
SNOMED	Malignant melanoma of great toe (disorder)	188077000
SNOMED	Malignant tumor of sphenoid sinus (disorder)	363428005
SNOMED	Secondary malignant neoplasm of intrapelvic lymph nodes (disorder)	94350006
SNOMED	Primary squamous cell carcinoma of overlapping lesion of accessory sinuses (disorder)	721605003
SNOMED	Secondary malignant neoplasm of greater curvature of stomach (disorder)	94320003
SNOMED	Secondary malignant neoplasm of maxillary sinus (disorder)	94406009
SNOMED	Reticulosarcoma associated with acquired immunodeficiency syndrome (disorder)	420302007
SNOMED	Primary malignant neoplasm of peripheral nerves of lower limb (disorder)	109937001
SNOMED	Primary squamous cell carcinoma of middle third of esophagus (disorder)	721619003

SNOMED	Malignant tumor of posterior margin of nasal septum and choanae (disorder)	254484001
SNOMED	Giant cell carcinoma of lung (disorder)	254631008
SNOMED	Burkitts tumor of intra-abdominal lymph nodes (disorder)	92508006
SNOMED	Primary malignant neoplasm of greater curvature of stomach (disorder)	93818001
SNOMED	Malignant tumor involving right ovary by separate metastasis from vagina (disorder)	369573005
SNOMED	Anaplastic large T-cell systemic malignant lymphoma (disorder)	404134006
SNOMED	Myxoid dermatofibrosarcoma protuberans (disorder)	253042009
SNOMED	Primary malignant neoplasm of pericardium (disorder)	93957005
SNOMED	Secondary malignant neoplasm of bronchus of right lower lobe (disorder)	94230009
SNOMED	Malignant melanoma of external auditory meatus (disorder)	188034001
SNOMED	Secondary malignant neoplasm of carina (disorder)	94238002
SNOMED	Primary malignant neoplasm of pituitary gland (disorder)	93964007
SNOMED	Malignant tumor of roof of nasopharynx (disorder)	363397008
SNOMED	Infiltrating ductal carcinoma of upper inner quadrant of right female breast (disorder)	1.08023E+15
SNOMED	Hormone receptor positive malignant neoplasm of breast (disorder)	417181009
SNOMED	Malignant neoplasm of anterior and lateral floor of mouth (disorder)	449034009
SNOMED	Non-Hodgkins lymphoma of skin (disorder)	448447004
SNOMED	Primary malignant neoplasm of breast (disorder)	372137005
SNOMED	Malignant teratoma of pineal region (disorder)	448250003
SNOMED	Primary malignant neoplasm of glomus jugulare (disorder)	93814004
SNOMED	Malignant tumor involving an organ by direct extension from endometrium (disorder)	369595009
SNOMED	Malignant tumor of posterior wall of nasopharynx (disorder)	187693006
SNOMED	Malignant tumor of sublingual gland (disorder)	363381003
SNOMED	Hypergranular promyelocytic leukemia (disorder)	278189009
SNOMED	Malignant neoplasm of subcutaneous fibrous tissue (disorder)	402871009
SNOMED	Malignant epithelial neoplasm of pineal gland (disorder)	448863000
SNOMED	Primary malignant neuroendocrine neoplasm of small intestine (disorder)	721670007
SNOMED	Diffuse non-Hodgkins lymphoma small cell (disorder)	109968002
SNOMED	Infiltrating lobular carcinoma of left female breast (disorder)	1.08026E+15
SNOMED	Malignant epithelial neoplasm of brain (disorder)	448989001
SNOMED	Primary adenocarcinoma of ascending colon and right flexure (disorder)	721695008
SNOMED	Non-Hodgkins lymphoma of prostate (disorder)	449318001
SNOMED	Primary malignant neoplasm of blood vessel of lower limb (disorder)	93703006
SNOMED	Follicular lymphoma grade 3b (disorder)	8.47701E+14
SNOMED	Siewert type III adenocarcinoma of esophagogastric junction (disorder)	439478008
SNOMED	Primary adenocarcinoma of lower lobe of left lung (disorder)	1.07888E+15
SNOMED	Hairy cell leukemia of spleen (disorder)	93151007
SNOMED	Primary cloacogenic carcinoma of anal canal (disorder)	721709000
SNOMED	Malignant tumor of cloacogenic zone (disorder)	363491008
SNOMED	Secondary malignant neoplasm of left lower lobe of lung (disorder)	94375005
SNOMED	Primary adenocarcinoma of peritoneum (disorder)	721725005
SNOMED	Hodgkins disease in remission (disorder)	426071002
SNOMED	Plasma cell myeloma/plasmacytoma (disorder)	415110002
SNOMED	Primary malignant neoplasm of soft tissues of hip (disorder)	94055006
SNOMED	Primary sarcoma of retroperitoneum (disorder)	722229001
SNOMED	Reactive oxygen species 1 positive non-small cell lung cancer (disorder)	722425009
SNOMED	Malignant neoplasm of anal canal (& anal carcinoma)	187762000
SNOMED	Secondary malignant neoplasm of intra-abdominal organs (disorder)	94348003
SNOMED	Primary papillary serous cystadenocarcinoma of endometrium (disorder)	1.07781E+14
SNOMED	Primary adenocarcinoma of ascending colon (disorder)	6.81601E+14
SNOMED	Malignant eccrine spiradenoma of skin (disorder)	403942003
SNOMED	Large cell anaplastic lymphoma T cell and Null cell type (disorder)	702785000

SNOMED	Malignant tumor of descending colon (disorder)	363409003
SNOMED	Burkitts lymphoma of lymph nodes of head face and neck (disorder)	188510001
SNOMED	Alveolar soft part sarcoma (disorder)	404056007
SNOMED	Hodgkins disease mixed cellularity of lymph nodes of axilla and upper limb (disorder)	188578002
SNOMED	Primary malignant nerve sheath neoplasm of peripheral nervous system structure (disorder)	722517009
SNOMED	Reticulosarcoma of lymph nodes of axilla and upper limb (disorder)	188492005
SNOMED	Hodgkins paragranuloma of extranodal AND/OR solid organ site (disorder)	93546006
SNOMED	Primary polymorphous low grade adenocarcinoma of oropharynx (disorder)	707398003
SNOMED	Familial malignant melanoma of skin (disorder)	726019003
SNOMED	Mu heavy chain disease (disorder)	61493004
SNOMED	Malignant neoplasm of testis: [NOS] or [seminoma] or [teratoma]	188228003
SNOMED	Primary squamous cell carcinoma of pharyngeal tonsil (disorder)	722530005
SNOMED	Malignant melanoma of skin of umbilicus (disorder)	93652001
SNOMED	Primary malignant neoplasm of prostate (disorder)	93974005
SNOMED	Diffuse non-Hodgkins lymphoma of intestine (disorder)	449176003
SNOMED	Compound leukemias (disorder)	190030009
SNOMED	Secondary malignant neoplasm of superficial cervical lymph nodes (disorder)	1.09014E+15
SNOMED	Primary thymic carcinoma (disorder)	722670005
SNOMED	Overlapping malignant neoplasm of colon (disorder)	109838007
SNOMED	Hodgkins granuloma of intrathoracic lymph nodes (disorder)	93531004
SNOMED	Malignant neoplasm of sclera (disorder)	188266000
SNOMED	Metastasis to peritoneum of unknown primary (disorder)	285616002
SNOMED	Mycosis fungoides with systemic infiltration (disorder)	404116007
SNOMED	Primary malignant neoplasm of bladder (disorder)	93689003
SNOMED	Anaplastic glioma of brain (disorder)	424151006
SNOMED	Spongiotic mycosis fungoides (disorder)	404117003
SNOMED	Large granular lymphocytic leukemia (disorder)	277569004
SNOMED	Acute promyelocytic leukemia FAB M3 in remission (disorder)	425869007
SNOMED	Carcinoma of anal canal (disorder)	285310000
SNOMED	Carcinoma of uterine cervix invasive (disorder)	423973006
SNOMED	Burkitts tumor of intrathoracic lymph nodes (disorder)	92510008
SNOMED	Reticulosarcoma of lymph nodes of inguinal region and lower limb (disorder)	188493000
SNOMED	Metastatic carcinoid tumor (disorder)	705176003
SNOMED	Embryonal sarcoma of liver (disorder)	716648006
SNOMED	Malignant neoplasm of connective and soft tissue of inguinal region (disorder)	188021002
SNOMED	Primary mucoepidermoid carcinoma of lacrimal apparatus (disorder)	722714004
SNOMED	Primary malignant meningioma (disorder)	722718001
SNOMED	Secondary malignant neoplasm of internal mammary lymph nodes (disorder)	1.09009E+15
SNOMED	Secondary malignant neoplasm of femur (disorder)	94300004
SNOMED	Secondary malignant neoplasm of blood vessel of axilla (disorder)	94189009
SNOMED	Metastasis to breast of unknown primary (disorder)	285634003
SNOMED	Non-small cell lung cancer with mutation in epidermal growth factor receptor (disorder)	703228009
SNOMED	Myelodysplastic syndrome: Refractory anemia without ringed sideroblasts without excess blasts (disorder)	109996008
SNOMED	Secondary malignant neoplasm of internal iliac lymph nodes (disorder)	1.09022E+15
SNOMED	Secondary malignant neoplasm of retroperitoneum (disorder)	94628003
SNOMED	Secondary malignant neoplasm of the mesocolon (disorder)	94625000
SNOMED	Primary malignant neoplasm of vestibule of mouth (disorder)	94138009
SNOMED	Diffuse malignant lymphoma - centroblastic (disorder)	302842009
SNOMED	Letterer-Siwe disease of lymph nodes of head face AND/OR neck (disorder)	93137007
SNOMED	Malignant tumor involving right fallopian tube by direct extension from ovary (disorder)	369549002
SNOMED	Monoclonal gammopathy of uncertain significance	277577000
SNOMED	Malignant tumor of undescended testis (disorder)	188219004

SNOMED	Primary chondrosarcoma of articular cartilage of pelvis (disorder)	723845003
SNOMED	Primary spindle cell squamous cell carcinoma of oropharynx (disorder)	707582006
SNOMED	Metastasis from malignant tumor of stomach (disorder)	315002002
SNOMED	Carcinoma of lung parenchyma (disorder)	254628007
SNOMED	Primary osteosarcoma of articular cartilage of jaw (disorder)	723849009
SNOMED	Primary malignant neuroendocrine neoplasm of pancreas (disorder)	735735001
SNOMED	Malignant melanoma of skin of wrist (disorder)	93654000
SNOMED	Primary osteosarcoma of articular cartilage of pelvis (disorder)	723852001
SNOMED	Paget disease of anal canal (disorder)	2.36811E+14
SNOMED	Primary malignant neoplasm of third cuneiform bone of foot (disorder)	94094007
SNOMED	Malignant infiltration of soft tissue (disorder)	278433008
SNOMED	Hodgkins paragranuloma of lymph nodes of axilla AND/OR upper limb (disorder)	93541001
SNOMED	Malignant tumor of nasal vestibule (disorder)	187831008
SNOMED	Secondary malignant neoplasm of endocardium (disorder)	94278009
SNOMED	Acute myelomonocytic leukemia FAB M4 in remission (disorder)	427658007
SNOMED	Primary malignant neoplasm of spinal cord (disorder)	94068003
SNOMED	Malignant neoplasm of connective and soft tissue of perineum (disorder)	188022009
SNOMED	Secondary malignant neoplasm of kidney (disorder)	94360002
SNOMED	Malignant neoplasm of peripheral nerves and autonomic nervous system (disorder)	188321006
SNOMED	Malignant neoplasm of intrahepatic canaliculi (disorder)	187776004
SNOMED	Primary sarcoma of right lower limb (disorder)	1.08175E+15
SNOMED	Malignant neoplasm of soft tissues of lower limb (disorder)	1.09096E+15
SNOMED	Secondary malignant neoplasm of blood vessel of perineum (disorder)	94203004
SNOMED	Primary poorly differentiated carcinoma of thyroid gland (disorder)	724552002
SNOMED	Malignant neoplasm of anterior wall of urinary bladder (disorder)	188242006
SNOMED	Primary malignant epithelial neoplasm of endocrine gland (disorder)	724554001
SNOMED	Primary malignant neoplasm of buccal mucosa (disorder)	371976008
SNOMED	Primary chondrosarcoma of bone of pelvis (disorder)	9.1061E+13
SNOMED	Malignant tumor involving bladder by separate metastasis from uterine cervix (disorder)	369480009
SNOMED	Malignant melanoma of buttock (disorder)	188051008
SNOMED	Primary squamous cell carcinoma of middle ear (disorder)	722676004
SNOMED	Carcinoma of ascending colon (disorder)	312111009
SNOMED	Secondary malignant neoplasm of nipple of female breast (disorder)	94443006
SNOMED	Lymphoma of sigmoid colon (disorder)	449218003
SNOMED	Primary liposarcoma of soft tissue of limb (disorder)	721577002
SNOMED	Primary malignant neuroendocrine neoplasm of anal canal (disorder)	733163007
SNOMED	Primary malignant nerve sheath neoplasm of autonomic nerve (disorder)	722518004
SNOMED	Primary malignant neoplasm of retromolar area (disorder)	93989001
SNOMED	Primary malignant neoplasm of peripheral nerves of shoulder (disorder)	109933002
SNOMED	Non-Hodgkins lymphoma of oral cavity (disorder)	448386004
SNOMED	Malignant neoplasm of hand bones (disorder)	269467007
SNOMED	Kaposi sarcoma of viscus (disorder)	7.2891E+13
SNOMED	Secondary malignant neoplasm of mediastinal lymph nodes (disorder)	94408005
SNOMED	Secondary malignant neoplasm of hamate bone (disorder)	94322006
SNOMED	Materno-fetal metastatic malignant melanoma (disorder)	402560002
SNOMED	Letterer-Siwe disease of lymph nodes of inguinal region AND/OR lower limb (disorder)	93138002
SNOMED	Primary malignant neoplasm of ciliary body primary (disorder)	93756008
SNOMED	Primary adenocarcinoma of chest wall (disorder)	9.1161E+13
SNOMED	Primary malignant neuroendocrine neoplasm of anus (disorder)	733162002
SNOMED	Malignant neoplasm of endocervical gland (disorder)	188177003
SNOMED	Malignant mast cell tumor of intrathoracic lymph nodes (disorder)	93202002
SNOMED	Primary squamous cell carcinoma of lip (disorder)	733344004

SNOMED	Primary malignant neoplasm of uterine adnexa (disorder)	94126000
SNOMED	Primary neuroendocrine carcinoma of body of stomach (disorder)	721640002
SNOMED	Primary mucinous cystic neoplasm with associated invasive carcinoma of cystic duct (disorder)	733347006
SNOMED	Adenoid cystic carcinoma of submandibular gland (disorder)	423189008
SNOMED	Philadelphia chromosome-negative precursor B-cell acute lymphoblastic leukemia (disorder)	714251006
SNOMED	Primary malignant neoplasm of minor salivary gland (disorder)	372000001
SNOMED	Carcinoma of submandibular gland (disorder)	254465004
SNOMED	Primary malignant neoplasm of cuboid (disorder)	93769007
SNOMED	Adenocarcinoma of sigmoid colon (disorder)	301756000
SNOMED	Malignant hemangiopericytoma of orbit (disorder)	254995004
SNOMED	Splenic lymphoma with villous lymphocytes (disorder)	277551008
SNOMED	Malignant neoplasm of cardiac orifice of stomach (disorder)	187733001
SNOMED	Primary malignant neoplasm of sebaceous gland (disorder)	94000008
SNOMED	Leukemic infiltrate of kidney (disorder)	236512004
SNOMED	Malignant melanoma of skin of upper lip (disorder)	424487008
SNOMED	Malignant tumor of pelvis (disorder)	363484005
SNOMED	Malignant neoplasm of cauda equina (disorder)	363477002
SNOMED	Malignant melanoma of skin of axilla (disorder)	93213004
SNOMED	Malignant histiocytosis of lymph nodes of inguinal region and lower limb (disorder)	188642004
SNOMED	Malignant lymphoma of intrathoracic lymph nodes (disorder)	93193008
SNOMED	Secondary malignant neoplasm of urethra (disorder)	94661005
SNOMED	Hodgkins disease lymphocytic depletion (disorder)	118610003
SNOMED	Malignant carcinoid tumor of kidney (disorder)	713574000
SNOMED	Seminoma of testis (disorder)	255107005
SNOMED	Malignant tumor involving uterine corpus by direct extension from uterine cervix (disorder)	369495006
SNOMED	Malignant melanoma of skin of hip (disorder)	93637008
SNOMED	Adenoid basal carcinoma of cervix uteri (disorder)	763063001
SNOMED	Diffuse large B-cell lymphoma of central nervous system (disorder)	734066005
SNOMED	Sarcoma of sternum (disorder)	449101009
SNOMED	Secondary malignant neoplasm of fundus of stomach (disorder)	94311007
SNOMED	Adenocarcinoma of nasopharynx (disorder)	423106003
SNOMED	Metastasis from malignant tumor of buccal cavity (disorder)	315008003
SNOMED	Secondary malignant neoplasm of islets of Langerhans (disorder)	94354002
SNOMED	Primary chondrosarcoma of articular cartilage (disorder)	735678002
SNOMED	Malignant tumor of neck (disorder)	363489000
SNOMED	Primary malignant neoplasm of blood vessel of foot (disorder)	93696001
SNOMED	Langerhans cell histiocytosis unifocal (disorder)	129000002
SNOMED	Malignant skin tumor with adnexal differentiation (disorder)	255093003
SNOMED	Primary mucoepidermoid carcinoma of trachea (disorder)	707379000
SNOMED	Secondary malignant neoplasm of facial nerve (disorder)	94294002
SNOMED	Primary malignant neuroepithelial neoplasm of iris (disorder)	735919002
SNOMED	Malignant neoplasm of mandible (disorder)	448668007
SNOMED	Primary malignant neuroepithelial neoplasm of orbit (disorder)	735921007
SNOMED	Overlapping malignant neoplasm of urinary system (disorder)	109870007
SNOMED	Secondary malignant neoplasm of scapula (disorder)	94529003
SNOMED	Carcinoma of pyloric antrum (disorder)	254559002
SNOMED	Malignant neoplasm of malar bone (disorder)	187903000
SNOMED	Leukemic infiltration of skin (T-cell lymphoblastic leukemia) (disorder)	404124002
SNOMED	Hodgkins disease nodular sclerosis of lymph nodes of multiple sites (disorder)	188572001
SNOMED	Secondary malignant neoplasm of blood vessel of hip (disorder)	94197002
SNOMED	Primary neuroendocrine carcinoma of duodenum (disorder)	721645007
SNOMED	Secondary malignant neoplasm of brain and spinal cord (disorder)	188462001

SNOMED	Small cell eccrine carcinoma of skin (disorder)	403944002
SNOMED	Malignant tumor of anterior two-thirds of tongue (disorder)	363360003
SNOMED	Malignant peripheral nerve sheath neoplasm with perineurial differentiation (disorder)	761958009
SNOMED	Spindle cell squamous cell carcinoma (disorder)	403900000
SNOMED	Primary malignant neoplasm of pelvic bones sacrum and coccyx (disorder)	372115003
SNOMED	Primary malignant neoplasm of round ligament of uterus (disorder)	93994001
SNOMED	Primary malignant neoplasm of pubis (disorder)	93975006
SNOMED	Primary malignant neuroendocrine neoplasm of perihilar bile duct (disorder)	762665002
SNOMED	Malignant neoplasm of short bone of lower limb (disorder)	712525007
SNOMED	Infiltrating ductal carcinoma of upper outer quadrant of left female breast (disorder)	1.08016E+15
SNOMED	Adenoid cystic carcinoma of cervix uteri (disorder)	763064007
SNOMED	Clear cell adenocarcinoma of ovary (disorder)	763131005
SNOMED	Sarcoma of scapula (disorder)	449294002
SNOMED	Rhabdomyosarcoma of cervix uteri (disorder)	763408003
SNOMED	Rhabdomyosarcoma of corpus uteri (disorder)	763409006
SNOMED	B-cell non-Hodgkins lymphoma (disorder)	1.09192E+15
SNOMED	Malignant plasma cell neoplasm extramedullary plasmacytoma (disorder)	188718006
SNOMED	Hodgkins sarcoma of lymph nodes of multiple sites (disorder)	188551004
SNOMED	Primary mucinous bronchiolo-alveolar carcinoma of lung (disorder)	707470000
SNOMED	Primary malignant neoplasm of cloacogenic zone (disorder)	109840002
SNOMED	Primary adenocarcinoma of digestive organ (disorder)	733355004
SNOMED	Malignant neoplasm of maxillofacial bone (disorder)	448558006
SNOMED	Primary small cell malignant neoplasm of lung TNM stage 3 (disorder)	6.7831E+13
SNOMED	Malignant melanoma of skin of groin (disorder)	93230002
SNOMED	Overlapping malignant neoplasm of bone and articular cartilage of limbs (disorder)	109348004
SNOMED	Primary malignant neoplasm of phalanx of hand (disorder)	93960003
SNOMED	Malignant tumor of tip of tongue (disorder)	254412006
SNOMED	Adenosarcoma of cervix uteri (disorder)	764847000
SNOMED	Kaposis sarcoma of lung (disorder)	109390005
SNOMED	Vaccine-induced fibrosarcoma (disorder)	370511006
SNOMED	Primary malignant neoplasm of coccyx (disorder)	93760006
SNOMED	Primary malignant neoplasm of tonsillar fossa (disorder)	94102002
SNOMED	Philadelphia chromosome-positive acute lymphoblastic leukemia (disorder)	425688002
SNOMED	Mantle cell B-cell lymphoma (nodal/systemic with skin involvement) (disorder)	404150003
SNOMED	Reticulosarcoma co-occurrent with human immunodeficiency virus infection (disorder)	713483007
SNOMED	Primary malignant neoplasm of middle ear (disorder)	93894000
SNOMED	Malignant tumor of vocal cord (disorder)	363486007
SNOMED	Secondary malignant neoplasm of soft tissues of abdomen (disorder)	94582005
SNOMED	Classic mycosis fungoides (disorder)	765328000
SNOMED	Primary malignant mixed Mullerian neoplasm of endometrium (disorder)	1.07751E+14
SNOMED	Secondary malignant neoplasm of nasal cavity (disorder)	94436000
SNOMED	Primary malignant neoplasm of upper respiratory tract (disorder)	94118008
SNOMED	Primary malignant neuroendocrine neoplasm of biliary tract (disorder)	733353006
SNOMED	Nonsquamous nonsmall cell neoplasm of lung (disorder)	440173001
SNOMED	Gestational choriocarcinoma (disorder)	417570003
SNOMED	Primitive neuroectodermal tumor of cervix uteri (disorder)	766248004
SNOMED	Neurolymphomatosis (disorder)	766752000
SNOMED	Malignant sex cord tumor of ovary (disorder)	254860001
SNOMED	Undifferentiated carcinoma of corpus uteri (disorder)	766758001
SNOMED	Malignant melanoma of hip (disorder)	188068007
SNOMED	Malignant neoplasm of connective and soft tissue of abdomen (disorder)	188015001
SNOMED	Malignant neoplasm of nail apparatus (disorder)	402636006

SNOMED	Secondary malignant neoplasm of muscle of upper limb (disorder)	94431005
SNOMED	Primary malignant neoplasm of ascending colon (disorder)	93683002
SNOMED	Secondary malignant neoplasm of rib (disorder)	94521000
SNOMED	Malignant neoplasm of lower lip buccal aspect (disorder)	187613005
SNOMED	Primary malignant neoplasm of muscle of thorax (disorder)	93910009
SNOMED	Pineoblastoma (disorder)	767448007
SNOMED	Malignant lymphoma follicular center cell non-cleaved (disorder)	303057009
SNOMED	Immature teratoma of ovary (disorder)	254871000
SNOMED	Malignant neoplasm of cricoid cartilage (disorder)	187844003
SNOMED	Overlapping malignant melanoma of skin (disorder)	109267002
SNOMED	Secondary malignant neoplasm of liver and intrahepatic bile duct (disorder)	8.13671E+14
SNOMED	Anaplastic lymphoma kinase positive anaplastic large cell lymphoma (disorder)	8.15011E+14
SNOMED	Malignant tumor of body of pancreas (disorder)	187791002
SNOMED	Refractory anaemia with multilineage dysplasia (disorder)	8.20601E+14
SNOMED	Anaplastic astrocytoma of central nervous system (disorder)	8.2501E+13
SNOMED	Malignant tumor of soft tissue of hip (disorder)	363364007
SNOMED	Malignant tumor involving vagina by separate metastasis from ovary (disorder)	369584003
SNOMED	Mycosis fungoides of intra-abdominal lymph nodes (disorder)	94707004
SNOMED	Secondary malignant neoplasm of upper respiratory tract (disorder)	94656008
SNOMED	Secondary malignant neoplasm of pyloric antrum (disorder)	94505005
SNOMED	Malignant tumor of anterior two-thirds of tongue - lateral margin (disorder)	254408000
SNOMED	Adenocarcinoma of stomach (disorder)	408647009
SNOMED	Local recurrence of malignant tumor of breast (disorder)	314955001
SNOMED	Primary verrucous carcinoma of larynx (disorder)	707427000
SNOMED	Malignant melanoma of meninges (disorder)	277530005
SNOMED	Primary malignant neoplasm of dorsal surface of tongue (disorder)	93773005
SNOMED	Malignant glioma of hypothalamus (disorder)	8.7111E+13
SNOMED	Malignant tumor of lateral wall of nasopharynx (disorder)	363398003
SNOMED	Myelofibrosis	52967002
SNOMED	Neuroblastoma	87364003
SNOMED	Malignant neoplasm of second metacarpal bone (disorder)	187946002
SNOMED	Hydroa vacciniforme-like lymphoma (disorder)	763719001
SNOMED	Overlapping malignant neoplasm of penis (disorder)	109875002
SNOMED	Malignant tumor of pleura (disorder)	363433009
SNOMED	Primary rhabdomyosarcoma of intrathoracic organ (disorder)	722512003
SNOMED	Overlapping malignant neoplasm of larynx (disorder)	109369002
SNOMED	Osteosarcoma of bone (disorder)	307576001
SNOMED	Malignant melanoma of ciliary body (disorder)	255015006
SNOMED	Malignant tumor of soft tissue of head face and neck (disorder)	302816009
SNOMED	Secondary malignant neoplasm of supraglottis (disorder)	94616006
SNOMED	Abdominothoracic neuroblastoma (disorder)	281566005
SNOMED	Sarcoma of female breast (disorder)	448449001
SNOMED	Secondary malignant neoplasm of vulva (disorder)	94681006
SNOMED	Malignant neoplasm of intraabdominal organ (disorder)	448882009
SNOMED	Primary malignant neoplasm of head (disorder)	93824007
SNOMED	Acute monocytic leukemia in remission (disorder)	91858008
SNOMED	Primary malignant neoplasm of renal pelvis (disorder)	93985007
SNOMED	Acute myeloid leukemia disease (disorder)	91861009
SNOMED	Squamous cell carcinoma of lung (disorder)	254634000
SNOMED	Malignant immunoproliferative disease (disorder)	109980005
SNOMED	Primary synovial sarcoma of respiratory organ (disorder)	722827008
SNOMED	Malignant melanoma of esophagus (disorder)	721627007

SNOMED	Malignant tumor of eye (disorder)	363461003
SNOMED	Transitional cell carcinoma of left renal pelvis (disorder)	1.08228E+15
SNOMED	Primary adenocarcinoma of ampulla of Vater (disorder)	721718003
SNOMED	Prethymic and thymic T-cell lymphoma/leukemia (disorder)	277614006
SNOMED	Primary chondrosarcoma of bone of rib (disorder)	723846002
SNOMED	Chronic leukemia in remission (disorder)	92811003
SNOMED	Retinoblastoma (disorder)	370967009
SNOMED	Primary malignant neoplasm of bronchus of left upper lobe (disorder)	93730001
SNOMED	Malignant adenomatous neoplasm (disorder)	443961001
SNOMED	Malignant teratoma of testis (disorder)	416769008
SNOMED	Primary malignant neoplasm of esophagus (disorder)	371984007
SNOMED	Blastic plasmacytoid dendritic cell neoplasm (disorder)	445105005
SNOMED	Secondary malignant neoplasm of anal canal (disorder)	94165002
SNOMED	Malignant neoplasm of connective and soft tissue of hand (disorder)	187995002
SNOMED	Letterer-Siwe disease of lymph nodes of axilla AND/OR upper limb (disorder)	93136003
SNOMED	Secondary malignant neoplasm of foot (disorder)	94305009
SNOMED	Secondary malignant neoplasm of lower third of esophagus (disorder)	94388008
SNOMED	Mixed cell type lymphosarcoma of lymph nodes of head face and neck (disorder)	94690004
SNOMED	Letterer-Siwe disease of spleen (disorder)	93140007
SNOMED	Mixed glioma (disorder)	443937008
SNOMED	Leukemic infiltration of skin (T-cell prolymphocytic leukemia) (disorder)	404123008
SNOMED	Non-Hodgkins lymphoma of extranodal site (disorder)	447989004
SNOMED	Non-Hodgkins lymphoma of stomach (disorder)	448709005
SNOMED	Primary follicular dendritic cell sarcoma (disorder)	724650000
SNOMED	Malignant melanoma of skin of ear (disorder)	93220006
SNOMED	Leukemic infiltration of skin (chronic T-cell lymphocytic leukemia) (disorder)	404122003
SNOMED	Metastasis to mediastinum of unknown primary (disorder)	285607001
SNOMED	Secondary malignant neoplasm of skin (disorder)	94579000
SNOMED	Primary neuroendocrine carcinoma of rectum (disorder)	721701002
SNOMED	Local recurrence of malignant tumor of tongue (disorder)	314951005
SNOMED	Primary squamous cell carcinoma of vagina (disorder)	722678003
SNOMED	Tubulocystic renal cell carcinoma (disorder)	733603009
SNOMED	Malignant histiocytosis of intra-abdominal lymph nodes (disorder)	93182006
SNOMED	Secondary malignant neoplasm of gum (disorder)	94321004
SNOMED	Malignant histiocytosis of intrathoracic lymph nodes (disorder)	93184007
SNOMED	SÃƒ Â©zarys disease of extranodal AND/OR solid organ site (disorder)	95264000
SNOMED	Lymphomatoid granulomatosis of the lung (disorder)	239297008
SNOMED	Malignant tumor involving urethra by separate metastasis from prostate (disorder)	369468001
SNOMED	Sebaceous adenocarcinoma of eyelid (disorder)	231833004
SNOMED	Sarcoma of bone (disorder)	448710000
SNOMED	Malignant histiocytosis of extranodal AND/OR solid organ site (disorder)	93190006
SNOMED	Malignant neoplasm of lower lobe bronchus or lung (disorder)	187868006
SNOMED	Malignant melanoma of thumb (disorder)	188065005
SNOMED	Secondary malignant neoplasm of preauricular lymph nodes (disorder)	94501001
SNOMED	Primary malignant inflammatory neoplasm of female breast (disorder)	9.6291E+13
SNOMED	Malignant lymphoma of lymph nodes of head face AND/OR neck (disorder)	93195001
SNOMED	Diffuse follicle center lymphoma (disorder)	449220000
SNOMED	Malignant tumor of infrahyoid epiglottis (disorder)	254520006
SNOMED	Primary malignant neoplasm of laryngeal commissure (disorder)	93857009
SNOMED	Primary malignant neoplasm of phalanx of foot (disorder)	93959008
SNOMED	Malignant mast cell tumor of intra-abdominal lymph nodes (disorder)	93200005
SNOMED	Overlapping malignant neoplasm of small intestine (disorder)	109837002

SNOMED	Secondary malignant neoplasm of tonsillar fossa (disorder)	94639000
SNOMED	Malignant mast cell tumor of lymph nodes of axilla AND/OR upper limb (disorder)	93203007
SNOMED	Follicular non-Hodgkins lymphoma of stomach (disorder)	449222008
SNOMED	Malignant tumor of esophagus (disorder)	363402007
SNOMED	Neutrophilic leukemia (disorder)	307617006
SNOMED	Malignant melanoma of neck (disorder)	188046002
SNOMED	Malignant mast cell tumor of extranodal AND/OR solid organ site (disorder)	93208003
SNOMED	Overlapping malignant neoplasm of biliary tract (disorder)	109847004
SNOMED	Malignant melanoma of skin of shoulder (disorder)	93647006
SNOMED	Clear cell (mesonephric) neoplasm of ovary (disorder)	416712009
SNOMED	Metastatic adenocarcinoma of unknown origin (disorder)	307226002
SNOMED	Hormone sensitive prostate cancer (disorder)	722103009
SNOMED	Malignant melanoma of skin of breast (disorder)	93215006
SNOMED	Primary malignant neoplasm of anterior portion of floor of mouth (disorder)	93672006
SNOMED	Malignant melanoma of skin of cheek (disorder)	93217003
SNOMED	Malignant neoplasm of cerebellopontine angle (disorder)	448218008
SNOMED	Malignant melanoma of skin of chin (disorder)	93219000
SNOMED	Primary malignant neoplasm of omentum (disorder)	94090003
SNOMED	Malignant melanoma of skin of elbow (disorder)	93221005
SNOMED	Malignant germ cell tumor of testis (disorder)	713646001
SNOMED	Squamous cell carcinoma of mucous membrane of upper lip (disorder)	419240004
SNOMED	Malignant tumor of anus and anal canal (disorder)	255083005
SNOMED	Primary lymphoepithelial carcinoma of hypopharynx (disorder)	707491008
SNOMED	Myeloid leukemia in remission (disorder)	94716000
SNOMED	Secondary malignant neoplasm of cervical esophagus (disorder)	94249008
SNOMED	Extraskeletal myxoid chondrosarcoma (disorder)	404079008
SNOMED	Secondary malignant neoplasm of small intestine (disorder)	94580002
SNOMED	Squamous cell carcinoma of small intestine (disorder)	764735002
SNOMED	Malignant melanoma of skin of upper eyelid (disorder)	423494003
SNOMED	Malignant tumor involving an organ by separate metastasis from ovary (disorder)	369605005
SNOMED	Sarcoma of orbit (disorder)	699354006
SNOMED	Hodgkins disease lymphocytic depletion of lymph nodes of head face AND/OR neck (disorder)	93488004
SNOMED	Malignant neoplasm of middle lobe bronchus or lung (disorder)	187864008
SNOMED	Hodgkins disease lymphocytic depletion of extranodal AND/OR solid organ site (disorder)	93492006
SNOMED	Metastasis to colon of unknown primary (disorder)	285611007
SNOMED	Malignant tumor involving vulva by separate metastasis from endometrium (disorder)	369588000
SNOMED	Malignant melanoma of skin of forearm (disorder)	93228004
SNOMED	Hodgkins disease lymphocytic-histiocytic predominance of lymph nodes of axilla AND/OR upper limb (disorder)	93496009
SNOMED	Nodular malignant lymphoma lymphocytic - well differentiated (disorder)	302845006
SNOMED	Primary giant cell sarcoma of retroperitoneum (disorder)	722444002
SNOMED	Carcinoma of base of tongue (disorder)	271943005
SNOMED	Malignant tumor involving vulva by separate metastasis from uterine cervix (disorder)	369591000
SNOMED	Secondary malignant neoplasm of soft tissues of hip (disorder)	94587004
SNOMED	Primary malignant neoplasm of glossopharyngeal nerve (disorder)	93815003
SNOMED	Carcinoma of urinary bladder superficial (disorder)	425231005
SNOMED	Secondary malignant neoplasm of axillary tail of female breast (disorder)	94182000
SNOMED	Secondary malignant neoplasm of scaphoid bone (disorder)	94440009
SNOMED	Hodgkins disease nodular sclerosis of lymph nodes of axilla AND/OR upper limb (disorder)	93514006
SNOMED	Hodgkins disease nodular sclerosis of lymph nodes of head face AND/OR neck (disorder)	93515007
SNOMED	Local recurrence of malignant tumor of soft tissue (disorder)	314974009
SNOMED	Large cell carcinoma of lung TNM stage 1 (disorder)	424938000
SNOMED	Burkitts lymphoma of intrapelvic lymph nodes (disorder)	188515006

SNOMED	Primary malignant neuroendocrine neoplasm of jejunum (disorder)	737312002
SNOMED	Kaposis sarcoma (disorder)	109385007
SNOMED	Primary malignant neoplasm of pisiform bone of hand (disorder)	93963001
SNOMED	Secondary malignant neoplasm of thyroglossal duct (disorder)	94633004
SNOMED	Subcutaneous panniculitic cutaneous T-cell lymphoma (disorder)	404133000
SNOMED	Malignant melanoma of umbilicus (disorder)	188055004
SNOMED	Hodgkins disease of lymph nodes of multiple sites (disorder)	93526001
SNOMED	Hodgkins disease of spleen (disorder)	93527005
SNOMED	Malignant tumor of anterior wall of nasopharynx (disorder)	187700006
SNOMED	Primary malignant neoplasm of subglottis (disorder)	94075002
SNOMED	Malignant tumor of urethral stump (disorder)	254934003
SNOMED	Malignant neoplasm of upper eyelid (disorder)	313249007
SNOMED	Hodgkins granuloma of lymph nodes of head face AND/OR neck (disorder)	93533001
SNOMED	Hodgkins granuloma of lymph nodes of inguinal region AND/OR lower limb (disorder)	93534007
SNOMED	Hodgkins granuloma of spleen (disorder)	93536009
SNOMED	Malignant tumor of external ear (disorder)	277156006
SNOMED	Primary malignant neoplasm of laryngeal cartilage (disorder)	109370001
SNOMED	Malignant neoplasm, overlapping lesion of connective and soft tissue	188026007
SNOMED	Collecting duct carcinoma of kidney (disorder)	733470002
SNOMED	Secondary malignant neoplasm of skin of abdomen (disorder)	94539009
SNOMED	[X]Malignant neoplasms of lymphoid haematopoietic and related tissue (disorder)	4.62401E+14
SNOMED	Aleukemic monocytic leukemia (disorder)	188748009
SNOMED	Adenoid cystic carcinoma of salivary gland (disorder)	422833009
SNOMED	Delta heavy chain disease (disorder)	20224008
SNOMED	Hodgkins sarcoma of lymph nodes of axilla AND/OR upper limb (disorder)	93550004
SNOMED	Hodgkins sarcoma of lymph nodes of head face AND/OR neck (disorder)	93551000
SNOMED	[X]Malignant neoplasm of respiratory and intrathoracic organs (disorder)	3.97641E+14
SNOMED	Primary malignant neoplasm of maxillary sinus (disorder)	93889000
SNOMED	Hodgkins sarcoma of extranodal AND/OR solid organ site (disorder)	93555009
SNOMED	Clinical stage C chronic lymphocytic leukaemia (disorder)	8.63781E+14
SNOMED	Ganglioneuroblastoma of central nervous system (disorder)	733926004
SNOMED	Recurrent malignant neoplasm of prostate (disorder)	1.09898E+15
SNOMED	Secondary malignant neoplasm of anterior mediastinum (disorder)	94167005
SNOMED	Malignant melanoma of skin of lower limb (disorder)	93641007
SNOMED	Hodgkins sarcoma of intra-abdominal lymph nodes (disorder)	93547002
SNOMED	Primary cutaneous plasmacytoma (disorder)	404142007
SNOMED	Secondary malignant neoplasm of skin of hip and leg (disorder)	188459004
SNOMED	Malignant tumor of transverse colon (disorder)	363408006
SNOMED	Secondary malignant neoplasm of occipital bone (disorder)	94447007
SNOMED	Malignant melanoma of skin of abdomen (disorder)	93210001
SNOMED	Malignant tumor of soft tissue of thorax (disorder)	363365008
SNOMED	Hereditary clear cell renal cell carcinoma (disorder)	764961009
SNOMED	Primary malignant neoplasm of frontal sinus (disorder)	93808006
SNOMED	Primary malignant neoplasm of vallecula (disorder)	94132005
SNOMED	Malignant neoplasm of skin	372130007
SNOMED	Carcinoma of central portion of breast (disorder)	708921005
SNOMED	Leukemic infiltration of skin (disorder)	404156009
SNOMED	Carcinoma of lip (disorder)	269515006
SNOMED	Eccrine ductal carcinoma of skin (disorder)	403939009
SNOMED	Primary malignant neoplasm of brain stem (disorder)	93726004
SNOMED	Primary malignant neoplasm of hepatic flexure of colon (disorder)	93826009
SNOMED	Secondary malignant neoplasm of parietal bone (disorder)	94470004

SNOMED	Primary malignant neoplasm of acromion (disorder)	93661001
SNOMED	Diffuse malignant lymphoma - large non-cleaved cell (disorder)	277629008
SNOMED	CD-30 negative pleomorphic large T-cell cutaneous lymphoma (disorder)	404130002
SNOMED	Malignant neoplasm of bone and articular cartilage of limb (disorder)	1.09087E+15
SNOMED	Low grade T-cell lymphoma (disorder)	277642008
SNOMED	Secondary malignant neoplasm of sacrum (disorder)	94527001
SNOMED	Malignant tumor of Islets of Langerhans (disorder)	187794005
SNOMED	Malignant epithelial neoplasm of peritoneum (disorder)	447781009
SNOMED	Secondary malignant neoplasm of blood vessel of lower limb (disorder)	94200001
SNOMED	Malignant carcinoid tumor of rectum (disorder)	713573006
SNOMED	Primary malignant neoplasm of aortic body (disorder)	93677000
SNOMED	Hodgkins disease lymphocytic-histiocytic predominance of lymph nodes of head face and neck (disorder)	188554007
SNOMED	Malignant tumor of lesser curve of stomach (disorder)	269459004
SNOMED	Malignant odontogenic neoplasm of lower jaw (disorder)	710195004
SNOMED	Primary carcinoma of sphenoidal sinus (disorder)	707348004
SNOMED	Lymphoproliferative disorder (disorder)	277466009
SNOMED	Diffuse non-Hodgkins lymphoma lymphoblastic (disorder)	109965004
SNOMED	Epithelioid malignant nerve sheath tumor (disorder)	404038007
SNOMED	Secondary malignant neoplasm of areola of male breast (disorder)	94177007
SNOMED	Primary epithelial-myoepithelial carcinoma of oropharynx (disorder)	707588005
SNOMED	Secondary malignant neoplasm of tonsillar pillar (disorder)	94640003
SNOMED	Granulocytic sarcoma affecting skin (disorder)	404155008
SNOMED	Secondary malignant neoplasm of blood vessel of head (disorder)	94196006
SNOMED	Multiple solitary plasmacytomas (disorder)	414785000
SNOMED	Neuroblastoma (disorder)	432328008
SNOMED	Pagetoid reticulosis (disorder)	404119000
SNOMED	Malignant melanoma animal-type (disorder)	402562005
SNOMED	Primary malignant neoplasm of blood vessel of hand (disorder)	93698000
SNOMED	Mesothelial neoplasm	115232000
SNOMED	Primary squamous cell carcinoma of naris (disorder)	440527006
SNOMED	Primary neuroendocrine carcinoma of esophagus (disorder)	721625004
SNOMED	Primary malignant neoplasm of long bone of lower limb (disorder)	93871001
SNOMED	Erythroleukemia FAB M6 (disorder)	93451002
SNOMED	Malignant neoplasm of adrenal medulla (disorder)	371965009
SNOMED	Primary malignant neoplasm of uvula (disorder)	94129007
SNOMED	Primary malignant neoplasm of the mesocolon (disorder)	94089007
SNOMED	Primary neuroendocrine carcinoma of colon (disorder)	721697000
SNOMED	Primary malignant neoplasm of blood vessel of thigh (disorder)	93709005
SNOMED	Malignant tumor of soft tissue of neck (disorder)	363441009
SNOMED	Dermatofibrosarcoma protuberans with myoid differentiation (disorder)	404008001
SNOMED	Malignant epithelial neoplasm of oropharynx (disorder)	448214005
SNOMED	Carcinoma of glans penis (disorder)	254904008
SNOMED	Malignant neoplasm overlapping lesion of cervix uteri (disorder)	188180002
SNOMED	Adenocarcinoma of anus (disorder)	423607006
SNOMED	Malignant tumor of trigone of urinary bladder (disorder)	188239000
SNOMED	Primary malignant sarcoma of skin (disorder)	721541009
SNOMED	Extraskeletal mesenchymal chondrosarcoma (disorder)	404080006
SNOMED	Malignant melanoma of eye (disorder)	274087000
SNOMED	Secondary malignant neoplasm of muscle of head (disorder)	94421003
SNOMED	Squamous cell carcinoma of nose (disorder)	448930008
SNOMED	Metastasis from malignant tumor of soft tissues (disorder)	314989000
SNOMED	Primary malignant neoplasm of bone (disorder)	93725000

SNOMED	Primary adenocarcinoma of ethmoidal sinus (disorder)	707342003
SNOMED	Glioblastoma multiforme of brain (disorder)	276828006
SNOMED	Adenocarcinoma of pancreas (disorder)	700423003
SNOMED	Primary malignant neoplasm of head of pancreas (disorder)	372119009
SNOMED	Hodgkins granuloma of extranodal AND/OR solid organ site (disorder)	93537000
SNOMED	Malignant tumor of meninges (disorder)	363497007
SNOMED	Malignant tumor of urethra (disorder)	363459007
SNOMED	Primary squamous cell carcinoma of nasopharynx (disorder)	707528007
SNOMED	Malignant neoplasm of lacrimal sac (disorder)	188273005
SNOMED	Primary malignant neoplasm of calcaneus (disorder)	93737003
SNOMED	Primary malignant neoplasm of cardia of stomach (disorder)	93738008
SNOMED	Metastasis to digestive organs (disorder)	275266006
SNOMED	Primary malignant neoplasm of carotid body (disorder)	93740003
SNOMED	Lymphomatoid papulosis type B - mycosis fungoides-like (disorder)	404104001
SNOMED	Primary malignant neoplasm of cartilage of nose (disorder)	93742006
SNOMED	Secondary malignant neoplasm of orbit proper (disorder)	94453007
SNOMED	Hodgkins disease lymphocytic-histiocytic predominance of intra-abdominal lymph nodes (disorder)	93493001
SNOMED	Malignant neoplasm of lower eyelid (disorder)	313250007
SNOMED	Primary adenocarcinoma of esophagogastric junction (disorder)	721628002
SNOMED	Primary malignant neoplasm of cerebral meninges (disorder)	93747000
SNOMED	Malignant neoplasm of isthmus of uterine body (disorder)	188195000
SNOMED	Malignant tumor of vermilion border of lower lip (disorder)	363373004
SNOMED	Leukemic reticuloendotheliosis of lymph nodes of head face and neck (disorder)	188645002
SNOMED	Malignant neoplasm of upper lobe of lung (disorder)	187862007
SNOMED	Metastasis to large intestine of unknown primary (disorder)	285610008
SNOMED	Choriocarcinoma of ovary (disorder)	254870004
SNOMED	Primary malignant neoplasm of vermilion border of lip (disorder)	94135007
SNOMED	Fibroblastic reticular cell neoplasm (disorder)	721314003
SNOMED	Primary malignant neoplasm of coccygeal body (disorder)	93759001
SNOMED	Malignant melanoma of upper limb (disorder)	269580008
SNOMED	Primary malignant neoplasm of colon (disorder)	93761005
SNOMED	Fibrolamellar hepatocellular carcinoma (disorder)	253018005
SNOMED	Chromophobe renal cell carcinoma (disorder)	733471003
SNOMED	Malignant tumor of iris (disorder)	188264002
SNOMED	Primary malignant neoplasm of urethra (disorder)	94123008
SNOMED	Primary malignant neoplasm of septum of nose (disorder)	94002000
SNOMED	Malignant teratoma of descended testis (disorder)	417554000
SNOMED	Secondary malignant neoplasm of accessory sinus (disorder)	94155008
SNOMED	Secondary malignant neoplasm of retromolar area (disorder)	94518002
SNOMED	Carcinoma of hepatic flexure (disorder)	312114001
SNOMED	Familial papillary thyroid carcinoma with renal papillary neoplasia syndrome (disorder)	716657000
SNOMED	Primary malignant neoplasm of duodenum (disorder)	93775003
SNOMED	Primary malignant neoplasm of ectopic female breast tissue (disorder)	93776002
SNOMED	Hodgkins disease lymphocytic depletion of intra-abdominal lymph nodes (disorder)	188587006
SNOMED	Primary malignant neoplasm of second cuneiform bone of foot (disorder)	94001007
SNOMED	Secondary malignant neoplasm of upper limb (disorder)	94654006
SNOMED	Carcinoma of upper gum (disorder)	254424004
SNOMED	Overlapping malignant neoplasm of floor of mouth (disorder)	109830000
SNOMED	Malignant neoplasm of mesorectum (disorder)	187811009
SNOMED	Primary malignant neoplasm of epiglottis (disorder)	93784003
SNOMED	Primary malignant neoplasm of nasal bone (disorder)	93916003
SNOMED	Malignant tumor involving right fallopian tube by direct extension from uterine cervix (disorder)	369550002

SNOMED	Malignant melanoma of skin of foot (disorder)	93227009
SNOMED	Carcinoma of cervical part of esophagus (disorder)	254535007
SNOMED	Primary adenocarcinoma of middle third of esophagus (disorder)	721623006
SNOMED	Malignant tumor of glans penis (disorder)	363451005
SNOMED	Acute leukemia in remission (disorder)	91854005
SNOMED	Plasma cell leukemia in remission (disorder)	95209008
SNOMED	Primary squamous cell carcinoma of overlapping lesion of male genital organ (disorder)	721604004
SNOMED	Overlapping malignant neoplasm of body of uterus (disorder)	109879008
SNOMED	Anaplastic lymphoma kinase negative anaplastic large cell lymphoma (disorder)	448212009
SNOMED	Primary malignant neoplasm of submaxillary gland (disorder)	94077005
SNOMED	Secondary malignant neoplasm of posterior hypopharyngeal wall (disorder)	94496002
SNOMED	Secondary malignant neoplasm of lymph nodes of upper limb (disorder)	94398002
SNOMED	Primary malignant neoplasm of pylorus (disorder)	93977003
SNOMED	Asymptomatic multiple myeloma (disorder)	440422002
SNOMED	Primary malignant neoplasm of frontal lobe (disorder)	93807001
SNOMED	Primary malignant neoplasm of soft tissues of upper limb (disorder)	372012002
SNOMED	Malignant tumor involving bladder by separate metastasis from fallopian tube (disorder)	369477008
SNOMED	Primary adenocarcinoma of overlapping lesion of small intestine (disorder)	721669006
SNOMED	Malignant tumor of soft tissue of pelvis (disorder)	363366009
SNOMED	Primary neuroendocrine carcinoma of small intestine (disorder)	721671006
SNOMED	Follicular non-Hodgkins lymphoma diffuse follicle center sub-type grade 1 (disorder)	702786004
SNOMED	Primary adenosquamous cell carcinoma of larynx (disorder)	707424007
SNOMED	Primary malignant neoplasm of vertebral column (disorder)	372028007
SNOMED	Malignant neoplasm of labia minora (disorder)	363447008
SNOMED	Malignant neoplasm of medial cuneiform (disorder)	187968007
SNOMED	Secondary malignant neoplasm of carotid body (disorder)	94239005
SNOMED	Primary malignant neoplasm of heart (disorder)	93825008
SNOMED	Primary malignant neoplasm of thyroglossal duct (disorder)	94097000
SNOMED	Metastatic sarcoma (disorder)	443144000
SNOMED	Secondary malignant neoplasm of body of stomach (disorder)	94214001
SNOMED	Malignant lymphoma large cell polymorphous immunoblastic (disorder)	371134001
SNOMED	Primitive neuroectodermal tumor (disorder)	699028006
SNOMED	Primary malignant neoplasm of hypopharynx (disorder)	93831006
SNOMED	Local recurrence of malignant melanoma of skin (disorder)	314976006
SNOMED	Primary leiomyosarcoma of retroperitoneum (disorder)	722513008
SNOMED	Sarcoma of clavicle (disorder)	449268006
SNOMED	Carcinoma of genital organ (disorder)	286899003
SNOMED	Primary malignant neoplasm of inner aspect of lower lip (disorder)	93836001
SNOMED	Carcinoma of breast - upper inner quadrant (disorder)	286893002
SNOMED	Small cell carcinoma (disorder)	1.10105E+16
SNOMED	Primary malignant neoplasm of spermatic cord (disorder)	372013007
SNOMED	Local recurrence of malignant tumor of bone (disorder)	314973003
SNOMED	Primary malignant neoplasm of islets of Langerhans (disorder)	93843007
SNOMED	Malignant tumor of epididymis (disorder)	363452003
SNOMED	Secondary malignant neoplasm of blood vessel of buttock (disorder)	94190000
SNOMED	B-cell prolymphocytic leukemia (disorder)	277619001
SNOMED	Secondary malignant neoplasm of renal pelvis (disorder)	94514000
SNOMED	Malignant tumor involving right fallopian tube by separate metastasis from uterus (disorder)	369557004
SNOMED	Malignant neoplasm overlapping lesion of accessory sinuses (disorder)	187838002
SNOMED	Metastatic neuroblastoma of orbit proper (disorder)	414676007
SNOMED	Primary malignant neoplasm of large intestine (disorder)	93854002
SNOMED	Malignant tumor of extrahepatic bile duct (disorder)	363416002

SNOMED	Primary malignant neoplasm of fundus of stomach (disorder)	93809003
SNOMED	Primary malignant neoplasm of lateral portion of floor of mouth (disorder)	93860002
SNOMED	Anaplastic thyroid carcinoma (disorder)	255031003
SNOMED	Primary malignant neoplasm of lateral wall of oropharynx (disorder)	93862005
SNOMED	Malignant melanoma of anus (disorder)	276821000
SNOMED	Malignant neoplasm of upper-inner quadrant of female breast (disorder)	188152004
SNOMED	Carcinoma of frenum of upper lip (disorder)	254398008
SNOMED	Sarcoma of upper inner quadrant of female breast (disorder)	448408001
SNOMED	Secondary malignant neoplasm of skin of eyelid (disorder)	94553009
SNOMED	Secondary malignant neoplasm of fallopian tube (disorder)	94295001
SNOMED	Primary malignant neoplasm of long bone of upper limb (disorder)	93872008
SNOMED	Clear cell eccrine hidradenocarcinoma of skin (disorder)	403940006
SNOMED	Malignant melanoma of skin of right lower limb (disorder)	3.52001E+14
SNOMED	Secondary malignant neoplasm of duodenum (disorder)	94275007
SNOMED	Malignant ascites (disorder)	236005001
SNOMED	Malignant neoplasm of long bone of lower limb (disorder)	449627008
SNOMED	Primary malignant neoplasm of lung (disorder)	93880001
SNOMED	Primary malignant neoplasm of lacrimal apparatus (disorder)	721548003
SNOMED	Malignant tumor of salivary gland (disorder)	255072001
SNOMED	Primary malignant neoplasm of muscle of upper limb (disorder)	93912001
SNOMED	Malignant neoplasm of rectum rectosigmoid junction and anus (disorder)	187760008
SNOMED	Primary malignant neoplasm of upper lobe bronchus or lung (disorder)	372135002
SNOMED	Primary malignant neoplasm of cornea primary (disorder)	93766000
SNOMED	Diffuse malignant lymphoma - small non-cleaved cell (disorder)	188674006
SNOMED	Malignant melanoma stage IIIC (finding)	9.56471E+14
SNOMED	Malignant neoplasm of anterior mediastinum (disorder)	449224009
SNOMED	Malignant mesothelioma of pelvic peritoneum (disorder)	109855006
SNOMED	Secondary malignant neoplasm of skin of forearm (disorder)	94557005
SNOMED	Papillary thyroid carcinoma (disorder)	255029007
SNOMED	Renal mucinous tubular and spindle cell carcinoma (disorder)	764990003
SNOMED	Primary malignant neoplasm of multiple endocrine glands (disorder)	93898002
SNOMED	Myeloid sarcoma disease (disorder)	94719007
SNOMED	Primary squamous cell carcinoma of sublingual gland (disorder)	737311009
SNOMED	CD-30 negative T-immunoblastic cutaneous lymphoma (disorder)	404131003
SNOMED	Primary malignant neoplasm of muscle of head (disorder)	93902008
SNOMED	Sarcoma upper outer quadrant of female breast (disorder)	448451002
SNOMED	Primary malignant neoplasm of muscle of inguinal region (disorder)	93904009
SNOMED	Primary malignant neoplasm of muscle of lower limb (disorder)	93905005
SNOMED	Malignant neoplasm of axillary tail of breast (disorder)	372094002
SNOMED	Malignant neoplasm of pituitary gland and craniopharyngeal duct (disorder)	188339002
SNOMED	Malignant tumor of ovary (disorder)	363443007
SNOMED	Squamous cell carcinoma arising in chronic ulcer (disorder)	448165009
SNOMED	Inflammatory carcinoma of breast (disorder)	254840009
SNOMED	Primary malignant neoplasm of lumbar vertebral column (disorder)	93878007
SNOMED	Primary malignant neoplasm of lacrimal gland (disorder)	371993008
SNOMED	Malignant teratoma of retroperitoneum (disorder)	448559003
SNOMED	Follicular lymphoma grade 3a (disorder)	8.47691E+14
SNOMED	Primary mucinous adenocarcinoma of endometrium (disorder)	722679006
SNOMED	Primary adenocarcinoma of lung (disorder)	707451005
SNOMED	Epsilon heavy chain disease (disorder)	60620005
SNOMED	Secondary malignant neoplasm of mastoid lymph nodes (disorder)	1.09015E+15
SNOMED	Primary clear cell adenocarcinoma of lung (disorder)	711414003

SNOMED	Primary seminoma of right testis (disorder)	1.08181E+15
SNOMED	Secondary malignant neoplasm of retroperitoneum and peritoneum (disorder)	188445006
SNOMED	Primary malignant neoplasm of nipple of female breast (disorder)	93924008
SNOMED	Primary malignant neoplasm of nose (disorder)	93926005
SNOMED	Malignant tumor of nasal sinuses (disorder)	363506007
SNOMED	Primary malignant neoplasm of blood vessel of thorax (disorder)	93710000
SNOMED	Adamantinoma of long bone (disorder)	307609003
SNOMED	Secondary malignant neoplasm of soft tissues of pelvis (disorder)	94591009
SNOMED	Malignant mast cell tumor of intrapelvic lymph nodes (disorder)	93201009
SNOMED	Primary malignant neoplasm of optic nerve (disorder)	93931007
SNOMED	Malignant neoplasm of upper respiratory tract (disorder)	449066004
SNOMED	Malignant tumor involving an organ by separate metastasis from uterine cervix (disorder)	369607002
SNOMED	Secondary malignant neoplasm of submental lymph nodes (disorder)	94611001
SNOMED	Primary malignant neoplasm of palatine bone (disorder)	93936002
SNOMED	Malignant neoplasm of cervical vertebra (disorder)	187916000
SNOMED	Malignant tumor of urinary system (disorder)	419052002
SNOMED	Local recurrence of malignant tumor of rectum (disorder)	314966008
SNOMED	Malignant tumor involving uterine corpus by separate metastasis from fallopian tube (disorder)	369575003
SNOMED	Malignant mast cell tumors (disorder)	188660004
SNOMED	Primary malignant neoplasm of endocervix (disorder)	93779009
SNOMED	Malignant neoplasm of hepatic duct (disorder)	187784000
SNOMED	Primary malignant neoplasm of parietal lobe (disorder)	93946000
SNOMED	Secondary malignant neoplasm of muscle of trunk (disorder)	94430006
SNOMED	Primary malignant neoplasm of parietal pleura (disorder)	93948004
SNOMED	Secondary malignant neoplasm of glossopharyngeal nerve (disorder)	94317006
SNOMED	Acute myeloid leukemia with normal karyotype (disorder)	703387000
SNOMED	Adenosarcoma of corpus uteri (disorder)	765740002
SNOMED	Molls gland adenocarcinoma (disorder)	403950007
SNOMED	Malignant tumor of scrotum (disorder)	363454002
SNOMED	Malignant neoplasm of inguinal region (disorder)	1.09094E+15
SNOMED	Primary malignant neoplasm of perirenal tissue (disorder)	93958000
SNOMED	Malignant tumor of tail of pancreas (disorder)	187792009
SNOMED	Hairy cell leukemia (disorder)	118613001
SNOMED	Primary malignant neoplasm of pharynx (disorder)	93961004
SNOMED	Malignant neoplasm of respiratory tract (disorder)	1.09023E+15
SNOMED	Overlapping primary malignant neoplasm of bone and articular cartilage of lower limb (disorder)	3.53661E+14
SNOMED	Malignant melanoma of scalp AND/OR neck (disorder)	188044004
SNOMED	Primary malignant neoplasm of pleura (disorder)	93966009
SNOMED	Primary malignant neoplasm of postcricoid region (disorder)	93967000
SNOMED	Primary rhabdomyosarcoma of oral cavity (disorder)	722509001
SNOMED	Adenocarcinoma of pigmented epithelium of ciliary body (disorder)	255016007
SNOMED	Malignant tumor of frontal sinus (disorder)	363427000
SNOMED	Malignant epithelial neoplasm of hypothalamus (disorder)	449253005
SNOMED	Spindle cell rhabdomyosarcoma (disorder)	404055006
SNOMED	Primary squamous cell carcinoma of anal canal (disorder)	722542000
SNOMED	Adenocarcinoma of appendix (disorder)	413445002
SNOMED	Lymphoma of cardia of stomach (disorder)	447806008
SNOMED	Primary malignant melanoma of cornea (disorder)	722665003
SNOMED	Primary malignant neoplasm of intrahepatic bile duct (disorder)	447109003
SNOMED	Metastasis to pancreas of unknown primary (disorder)	285614004
SNOMED	Secondary malignant neoplasm of blood vessel of thorax (disorder)	94207003
SNOMED	Primary malignant neoplasm of rectouterine pouch (disorder)	93981003

SNOMED	Primary malignant neoplasm of rectovaginal septum (disorder)	93982005
SNOMED	Primary malignant neoplasm of forearm (disorder)	93804008
SNOMED	Malignant tumor involving vagina by separate metastasis from fallopian tube (disorder)	369583009
SNOMED	Malignant tumor of lower labial sulcus (disorder)	187662005
SNOMED	Malignant tumor of spinal cord (disorder)	363475005
SNOMED	Kaposis sarcoma of palate (disorder)	109388009
SNOMED	Malignant teratoma of mediastinum (disorder)	278042005
SNOMED	Primary malignant neoplasm of peripheral nerves of head (disorder)	109923005
SNOMED	Secondary malignant neoplasm of male breast (disorder)	94401004
SNOMED	Primary malignant neoplasm of eustachian tube (disorder)	93788000
SNOMED	Acantholytic squamous cell carcinoma (disorder)	403901001
SNOMED	Primary malignant neoplasm of blood vessel of face (disorder)	93694003
SNOMED	Acute myeloid leukemia with t(6;9)(p23;q34) translocation (disorder)	733598001
SNOMED	Sarcoma of breast (disorder)	278050001
SNOMED	Primary malignant neoplasm of scapula (disorder)	93997008
SNOMED	Primary cutaneous diffuse large cell B-cell lymphoma of the leg (disorder)	404146005
SNOMED	Malignant neoplasm of border of tongue (disorder)	371974006
SNOMED	Primary malignant neoplasm of periadrenal tissue (disorder)	93955002
SNOMED	Primary malignant neoplasm of oral cavity (disorder)	372001002
SNOMED	Overlapping malignant neoplasm of pancreas (disorder)	109848009
SNOMED	Primary malignant neoplasm of short bone of upper limb (disorder)	94004004
SNOMED	Primary cutaneous CD30 antigen positive large T-cell lymphoma (disorder)	128875000
SNOMED	Non-small cell lung cancer without mutation in epidermal growth factor receptor (disorder)	703230006
SNOMED	Primary malignant neoplasm of carpal bone (disorder)	93741004
SNOMED	Malignant melanoma (vertical growth phase) (disorder)	402558004
SNOMED	Malignant neoplasm of cartilage of trachea (disorder)	187853005
SNOMED	Primary malignant neoplasm of soft tissues of axilla (disorder)	94051002
SNOMED	Secondary malignant neoplasm of cervix uteri (disorder)	188469005
SNOMED	Malignant immunoproliferative small intestinal disease (disorder)	188691005
SNOMED	Secondary malignant neoplasm of rectovesical septum (disorder)	94512001
SNOMED	Secondary neoplasm of right broad ligament (disorder)	369536007
SNOMED	Acute myeloid leukemia with maturation FAB M2 in remission (disorder)	426124006
SNOMED	Primary embryonal carcinoma of testis (disorder)	721602000
SNOMED	Malignant neoplasm of interlobular bile ducts (disorder)	187773007
SNOMED	Secondary malignant neoplasm of hand (disorder)	94323001
SNOMED	Primary malignant neoplasm of flank (disorder)	93801000
SNOMED	Primary malignant neoplasm of anus (disorder)	93676009
SNOMED	Malignant melanoma of skin of knee (disorder)	93638003
SNOMED	Secondary malignant neoplasm of right lower lobe of lung (disorder)	94522007
SNOMED	Malignant carcinoid tumor of bronchus (disorder)	726653000
SNOMED	Primary malignant neoplasm of sphenoidal sinus (disorder)	94067008
SNOMED	Malignant melanoma stage IIA (finding)	9.56371E+14
SNOMED	Malignant histiocytosis of spleen (disorder)	93189002
SNOMED	Primary malignant neoplasm of spleen (disorder)	94071006
SNOMED	IgA monoclonal gammopathy of uncertain significance	285423008
SNOMED	Primary malignant neoplasm of sternum (disorder)	94073009
SNOMED	Malignant hemangiopericytoma of skin (disorder)	254797000
SNOMED	Verrucous carcinoma of oral cavity (disorder)	403889000
SNOMED	Primary malignant neoplasm of nipple of male breast (disorder)	93925009
SNOMED	Primary cholangiocarcinoma of intrahepatic biliary tract (disorder)	721716004
SNOMED	Primary malignant neoplasm of supraclavicular region (disorder)	94079008
SNOMED	Malignant tumor involving vulva by separate metastasis from vagina (disorder)	369593002

SNOMED	Primary malignant neoplasm of nasal cavity (disorder)	93917007
SNOMED	Primary malignant neoplasm of tail of pancreas (disorder)	94082003
SNOMED	Malignant neoplasm of anorectum (disorder)	443488001
SNOMED	Primary malignant neoplasm of pelvis (disorder)	93953009
SNOMED	Primary malignant neoplasm of temporal bone (disorder)	94085001
SNOMED	B-cell lymphoma unclassifiable with features intermediate between Burkitt lymphoma and diffuse large B-cell lymphoma (disorder)	722953004
SNOMED	Primary malignant neoplasm of testis (disorder)	94087009
SNOMED	Malignant lymphoma of lymph nodes of axilla AND/OR upper limb (disorder)	93194002
SNOMED	Pulmonary blastoma (disorder)	189815007
SNOMED	Secondary malignant neoplasm of lower inner quadrant of female breast (disorder)	94385006
SNOMED	Primary adenocarcinoma of maxillary sinus (disorder)	707339009
SNOMED	Tumor stage mycosis fungoides (disorder)	404113004
SNOMED	Malignant neoplasm of nipple and areola of female breast (disorder)	188147009
SNOMED	Non-small cell carcinoma of lung TNM stage 1 (disorder)	424132000
SNOMED	Primary malignant neoplasm of thyroid gland (disorder)	94098005
SNOMED	Primary malignant neoplasm of floor of mouth (disorder)	93802007
SNOMED	Malignant tumor involving left fallopian tube by direct extension from uterine cervix (disorder)	369518007
SNOMED	Plasmablastic lymphoma (disorder)	724648008
SNOMED	Primary malignant neoplasm of tonsillar pillar (disorder)	94103007
SNOMED	Primary malignant neoplasm of trachea (disorder)	94104001
SNOMED	Adenocarcinoma of ileum (disorder)	304545002
SNOMED	Sarcoma of endometrium (disorder)	447266004
SNOMED	Primary malignant neoplasm of trigeminal nerve (disorder)	94108003
SNOMED	Secondary malignant neoplasm of bone of skull (disorder)	94220000
SNOMED	Malignant tumor involving vagina by separate metastasis from endometrium (disorder)	369582004
SNOMED	Primary malignant neoplasm of trunk (disorder)	94111002
SNOMED	Primary undifferentiated carcinoma of larynx (disorder)	707421004
SNOMED	Burkitts lymphoma (disorder)	118617000
SNOMED	Malignant neoplasm of fifth metatarsal bone (disorder)	187977000
SNOMED	Primary malignant neoplasm of peripheral nerves of neck (disorder)	109927006
SNOMED	Neurotropic malignant melanoma (disorder)	403925009
SNOMED	Metastasis from malignant tumor of gallbladder (disorder)	315001009
SNOMED	Adenocarcinoma of small intestine (disorder)	424440001
SNOMED	Primary malignant neoplasm of ureter (disorder)	94121005
SNOMED	Primary malignant neoplasm of Meckels diverticulum (disorder)	93890009
SNOMED	Primary malignant neoplasm of cranial nerve (disorder)	93767009
SNOMED	Theca steroid producing cell malignant neoplasm of ovary (disorder)	716855006
SNOMED	Secondary malignant neoplasm of blood vessel of toe (disorder)	94208008
SNOMED	Primary malignant neoplasm of temporal lobe (disorder)	94086000
SNOMED	Neuroendocrine carcinoma of appendix (disorder)	717916003
SNOMED	Secondary malignant neoplasm of supratrochlear lymph nodes (disorder)	1.0901E+15
SNOMED	Malignant melanoma of skin of penis (disorder)	448298007
SNOMED	Choriocarcinoma (disorder)	188188009
SNOMED	Carcinoma of middle lobe bronchus or lung (disorder)	372113005
SNOMED	Malignant neoplasm of midline of tongue (disorder)	187635002
SNOMED	Letterer-Siwe disease of extranodal AND/OR solid organ site (disorder)	93141006
SNOMED	Malignant astrocytoma of optic nerve (disorder)	254973003
SNOMED	Chronic monocytic leukemia (disorder)	188745007
SNOMED	Primary malignant neoplasm of soft tissues of face (disorder)	94053004
SNOMED	Primary malignant neoplasm of vomer (disorder)	94142007
SNOMED	Reactive immunoproliferative disease (disorder)	82546001
SNOMED	Infiltrating duct carcinoma of female breast (disorder)	448952004

SNOMED	Primary malignant neoplasm of zygomatic bone (disorder)	94145009
SNOMED	Megakaryocytic leukemia in remission (disorder)	94148006
SNOMED	Sarcoma of bladder (disorder)	278046008
SNOMED	Astrocytoma of brain (disorder)	254938000
SNOMED	Primary adenocarcinoma of common bile duct (disorder)	721559007
SNOMED	Mucoepidermoid carcinoma of submandibular gland (disorder)	423424005
SNOMED	Acute myeloid leukemia with CCAAT/enhancer binding protein alpha somatic mutation (disorder)	764855007
SNOMED	Malignant neoplasm of phalanges of foot (disorder)	187978005
SNOMED	Malignant tumor involving left ovary by direct extension from uterine cervix (disorder)	369527008
SNOMED	Secondary malignant neoplasm of pylorus (disorder)	94506006
SNOMED	Malignant tumor involving bladder by separate metastasis from uterus (disorder)	369481008
SNOMED	Secondary malignant neoplasm of adrenal gland (disorder)	94161006
SNOMED	Primary malignant neoplasm of small intestine (disorder)	94048009
SNOMED	Secondary malignant neoplasm of alveolar ridge mucosa (disorder)	94163009
SNOMED	Secondary malignant neoplasm of ampulla of Vater (disorder)	94164003
SNOMED	Letterer-Siwe disease of intrapelvic lymph nodes (disorder)	93134000
SNOMED	Non-Hodgkins lymphoma of testis (disorder)	448387008
SNOMED	Primary clear cell adenocarcinoma of trachea (disorder)	707471001
SNOMED	Malignant neoplasm of metacarpal bones (disorder)	255091001
SNOMED	Primary squamous cell carcinoma of anus (disorder)	723265000
SNOMED	Sarcoma of rib (disorder)	449269003
SNOMED	Secondary malignant neoplasm of anterior wall of urinary bladder (disorder)	94171008
SNOMED	Carcinoma ex pleomorphic adenoma of parotid gland (disorder)	425127006
SNOMED	Undifferentiated adnexal carcinoma of skin (disorder)	403955002
SNOMED	Primary malignant neuroendocrine neoplasm of lung (disorder)	722528008
SNOMED	Serous papillary cystadenocarcinoma ovary (disorder)	254850005
SNOMED	Primary malignant neoplasm of supraglottis (disorder)	94080006
SNOMED	Primary malignant neoplasm of face (disorder)	93792007
SNOMED	Overlapping malignant neoplasm of male breast (disorder)	109887009
SNOMED	Burkitts tumor of lymph nodes of inguinal region AND/OR lower limb (disorder)	92513005
SNOMED	Primary small cell neoplasm of thymus (disorder)	440525003
SNOMED	Nodular lymphoma of intra-abdominal lymph nodes (disorder)	95186006
SNOMED	Secondary malignant neoplasm of intra-abdominal lymph nodes (disorder)	94347008
SNOMED	Relapsing chronic myeloid leukemia (disorder)	415287001
SNOMED	CD-30 positive pleomorphic large T-cell cutaneous lymphoma (disorder)	404126000
SNOMED	Secondary malignant neoplasm of blood vessel of abdomen (disorder)	94187006
SNOMED	Infiltrating duct carcinoma of breast (disorder)	408643008
SNOMED	Malignant tumor involving an organ by separate metastasis from bladder (disorder)	369602008
SNOMED	Secondary malignant neoplasm of blood vessel of face (disorder)	94191001
SNOMED	Primary malignant neoplasm of peripheral nerves of trunk (disorder)	109947003
SNOMED	Hodgkins disease mixed cellularity (disorder)	118609008
SNOMED	Secondary malignant neoplasm of pubis (disorder)	94504009
SNOMED	Malignant neoplasm of abdomen (disorder)	188366002
SNOMED	Malignant mast cell tumor of lymph nodes of inguinal region AND/OR lower limb (disorder)	93205000
SNOMED	Secondary malignant neoplasm of skin of upper arm (disorder)	449631002
SNOMED	Secondary malignant neoplasm of mandible (disorder)	94403001
SNOMED	Pre B-cell acute lymphoblastic leukemia (disorder)	277572006
SNOMED	Malignant tumor of muscle (disorder)	363495004
SNOMED	Secondary malignant neoplasm of blood vessel of pelvis (disorder)	94202009
SNOMED	Malignant neoplasm of connective and soft tissue of toe (disorder)	188005007
SNOMED	Secondary malignant neoplasm of acromion (disorder)	94157000
SNOMED	Malignant tumor involving an organ by separate metastasis from uterus (disorder)	369608007

SNOMED	Malignant mixed tumor of salivary gland (disorder)	425225007
SNOMED	Malignant neoplasm of peripheral nerve of thorax (disorder)	188325002
SNOMED	Malignant tumor of nasal cavity (disorder)	363422006
SNOMED	Parathyroid carcinoma (disorder)	255037004
SNOMED	Malignant tumor of hard palate (disorder)	363387004
SNOMED	SÃƒ Â©zary disease of skin (disorder)	255101006
SNOMED	Mucinous adenocarcinoma of gastrointestinal tract (disorder)	9.4291E+13
SNOMED	Secondary malignant neoplasm of body of penis (disorder)	94213007
SNOMED	Secondary malignant neoplasm of perianal skin (disorder)	94483003
SNOMED	Overlapping malignant neoplasm of multiple endocrine glands (disorder)	109951001
SNOMED	Primary malignant neoplasm of lower gum (disorder)	371997009
SNOMED	Secondary malignant neoplasm of anterior portion of floor of mouth (disorder)	94168000
SNOMED	Secondary malignant neoplasm of anterior aspect of epiglottis (disorder)	94166001
SNOMED	Primary malignant neoplasm of ear nose AND/OR throat (disorder)	415177008
SNOMED	Adenocarcinoma of uterus (disorder)	309245001
SNOMED	Primary chondrosarcoma of bone of lower limb (disorder)	9.1081E+13
SNOMED	Primary malignant neoplasm of rectovesical septum (disorder)	93983000
SNOMED	SÃƒ Â©zarys disease of lymph nodes of multiple sites (disorder)	188637007
SNOMED	Malignant melanoma of chest wall (disorder)	313248004
SNOMED	Diffuse non-Hodgkins lymphoma of uterine cervix (disorder)	448607004
SNOMED	Primary lymphoepithelial carcinoma of larynx (disorder)	707360003
SNOMED	Malignant tumor involving vagina by direct extension from endometrium (disorder)	369505004
SNOMED	Secondary malignant neoplasm of iliac lymph nodes (disorder)	94336001
SNOMED	Secondary malignant neoplasm of jejunum (disorder)	94357009
SNOMED	Primary squamous cell carcinoma of submandibular gland (disorder)	737310005
SNOMED	Secondary malignant neoplasm of bronchus (disorder)	94233006
SNOMED	Secondary malignant neoplasm of navicular bone of foot (disorder)	94439007
SNOMED	Secondary malignant neoplasm of left fallopian tube (disorder)	369514009
SNOMED	Primary malignant neoplasm of trochlear nerve (disorder)	94110001
SNOMED	Primary adenocarcinoma of upper lobe of left lung (disorder)	1.0789E+15
SNOMED	Malignant neoplasm of connective and soft tissue of great toe (disorder)	188006008
SNOMED	Malignant tumor of lower limb (disorder)	363504005
SNOMED	Malignant neoplasm of carpal bone - lunate (disorder)	187938003
SNOMED	Malignant tumor involving left ovary by direct extension from endometrium (disorder)	369524001
SNOMED	Secondary malignant neoplasm of cauda equina (disorder)	94242004
SNOMED	Secondary malignant neoplasm of Waldeyers ring (disorder)	94682004
SNOMED	Malignant tumor involving left ovary by separate metastasis uterus (disorder)	369565001
SNOMED	Infantile fibrosarcoma (disorder)	403996004
SNOMED	Leiomyosarcoma of lower esophagus (disorder)	448229007
SNOMED	Diffuse non-Hodgkins lymphoma of nose (disorder)	449065000
SNOMED	Malignant neoplasm of ectopic site of male breast (disorder)	188168005
SNOMED	Diffuse non-Hodgkins lymphoma of stomach (disorder)	448663003
SNOMED	Immunoproliferative neoplasm in remission (disorder)	427374007
SNOMED	Secondary malignant neoplasm of cheek (disorder)	94252000
SNOMED	Malignant tumor involving right fallopian tube by separate metastasis from endometrium (disorder)	369553000
SNOMED	Malignant tumor involving right ovary by separate metastasis from endometrium (disorder)	369568004
SNOMED	Malignant neoplasm of phalanges of hand (disorder)	187950009
SNOMED	Secondary malignant neoplasm of palate (disorder)	94456004
SNOMED	Malignant fibrous histiocytoma (disorder)	443439001
SNOMED	Primary malignant neoplasm of soft palate (disorder)	94049001
SNOMED	Primary mesothelioma overlapping lesion of retroperitoneum peritoneum and omentum (disorder)	722677008
SNOMED	Primary papillary adenocarcinoma of lung (disorder)	707411007

SNOMED	Secondary malignant neoplasm of commissure of lip (disorder)	94261000
SNOMED	Secondary malignant neoplasm of common bile duct (disorder)	94262007
SNOMED	Primary squamous cell carcinoma of ear (disorder)	722112006
SNOMED	Carcinoma ventral surface of tongue (disorder)	275394001
SNOMED	Secondary malignant neoplasm of cornea (disorder)	94265009
SNOMED	Secondary malignant neoplasm of patella (disorder)	94476005
SNOMED	Malignant neoplasm of myometrium of corpus uteri (disorder)	188193007
SNOMED	Plasmacytoma (disorder)	415112005
SNOMED	Primary malignant neoplasm of sweat gland (disorder)	94081005
SNOMED	Lymphosarcoma of lymph nodes of inguinal region and lower limb (disorder)	188504001
SNOMED	Carcinoma of subglottis (disorder)	372104008
SNOMED	Sarcoma of liver (disorder)	254601002
SNOMED	Hodgkins disease mixed cellularity of lymph nodes of inguinal region AND/OR lower limb (disorder)	93507009
SNOMED	Malignant neoplasm of connective and soft tissue of sacrum or coccyx (disorder)	188023004
SNOMED	Primary papillary squamous cell carcinoma of hypopharynx (disorder)	707482009
SNOMED	Undifferentiated carcinoma of nasopharynx (disorder)	422541001
SNOMED	Secondary malignant neoplasm of ectopic male breast tissue (disorder)	94277004
SNOMED	Secondary malignant neoplasm of skin of nose (disorder)	94567000
SNOMED	Malignant neoplasm of carotid body (disorder)	447883002
SNOMED	Malignant neoplasm of liver and intrahepatic bile ducts (disorder)	187767006
SNOMED	Secondary malignant neoplasm of endometrium (disorder)	94281004
SNOMED	Primary malignant neoplasm of liver (disorder)	95214007
SNOMED	Malignant neoplasm of connective and soft tissues of trunk (disorder)	1.09089E+15
SNOMED	Secondary malignant neoplasm of frontal sinus (disorder)	94310008
SNOMED	Primary adenoid cystic carcinoma of nasopharynx (disorder)	7.391E+12
SNOMED	Secondary malignant neoplasm of esophagus (disorder)	94286009
SNOMED	Malignant neoplasm of connective and soft tissue of hip (disorder)	188000002
SNOMED	B-cell lymphoma (disorder)	109979007
SNOMED	Local recurrence of malignant tumor of buccal cavity (disorder)	314952003
SNOMED	Indolent multiple myeloma (disorder)	441313008
SNOMED	Malignant tumor of lacrimal gland (disorder)	188272000
SNOMED	Malignant ameloblastoma of mandible (disorder)	713290004
SNOMED	Malignant tumor involving left ovary by direct extension from uterus (disorder)	369528003
SNOMED	Non-Hodgkins lymphoma of central nervous system (disorder)	448254007
SNOMED	Microsatellite instability-high colorectal cancer (disorder)	737058005
SNOMED	Malignant glioma of brain (disorder)	276826005
SNOMED	Secondary malignant neoplasm of false vocal cord (disorder)	94296000
SNOMED	Malignant melanoma of forehead (disorder)	188040008
SNOMED	Secondary malignant neoplasm of female genital organ (disorder)	94298004
SNOMED	Secondary malignant neoplasm of femoral lymph nodes (disorder)	94299007
SNOMED	Carcinoid bronchial adenoma (disorder)	253003009
SNOMED	Primary malignant neoplasm of muscle of face (disorder)	93901001
SNOMED	Primary malignant neoplasm (disorder)	372087000
SNOMED	NK/T-cell lymphoma, nasal and nasal-type	128805001
SNOMED	Human epidermal growth factor 2 positive carcinoma of breast (disorder)	427685000
SNOMED	Malignant carcinoid tumor of small intestine (disorder)	709517003
SNOMED	Primary squamous cell carcinoma of oral cavity (disorder)	733343005
SNOMED	Primary solid papillary carcinoma with invasion of breast (disorder)	722832009
SNOMED	Malignant melanoma of accessory sinus (disorder)	707350007
SNOMED	Secondary malignant neoplasm of visceral pleura (disorder)	94678001
SNOMED	Non-Hodgkins lymphoma of bone (disorder)	448220006
SNOMED	Aleukemic leukemia (disorder)	302856006

SNOMED	Malignant tumor involving vulva by separate metastasis from ovary (disorder)	369590004
SNOMED	Primary malignant neoplasm of soft tissues of lower limb (disorder)	94057003
SNOMED	Hodgkins disease of extranodal AND/OR solid organ site (disorder)	93528000
SNOMED	Malignant tumor involving rectum by separate metastasis from prostate (disorder)	369458006
SNOMED	Secondary malignant neoplasm of glomus jugulare (disorder)	94316002
SNOMED	Malignant lymphoma - small lymphocytic (disorder)	302841002
SNOMED	Hodgkins granuloma of intrapelvic lymph nodes (disorder)	93530003
SNOMED	Squamous cell carcinoma of trachea (disorder)	254620000
SNOMED	Primary squamous cell carcinoma of paraurethral gland (disorder)	733134009
SNOMED	Primary malignant neoplasm of rectosigmoid junction (disorder)	93980002
SNOMED	Squamous cell carcinoma of lung TNM stage 4 (disorder)	425376008
SNOMED	Malignant neoplasm of ear (disorder)	443648003
SNOMED	Squamous cell carcinoma of lung TNM stage 1 (disorder)	423295000
SNOMED	Secondary malignant neoplasm of head of pancreas (disorder)	94325008
SNOMED	Secondary malignant neoplasm of head (disorder)	94326009
SNOMED	Secondary malignant neoplasm of heart (disorder)	94327000
SNOMED	Primary malignant neoplasm of anterior mediastinum (disorder)	93671004
SNOMED	Secondary malignant neoplasm of muscle of hip (disorder)	94422005
SNOMED	Lymphomatoid papulosis type C (anaplastic large-cell lymphoma-like) (disorder)	404105000
SNOMED	Secondary malignant neoplasm of hypoglossal nerve (disorder)	94331006
SNOMED	Lymphomatoid papulosis-associated mycosis fungoides (disorder)	404111002
SNOMED	Malignant medulloepithelioma of ciliary body (disorder)	416901002
SNOMED	Small cell carcinoma carcinomatosis (disorder)	424052001
SNOMED	Secondary malignant neoplasm of uveal tract (disorder)	94666000
SNOMED	Secondary malignant neoplasm of right ovary (disorder)	369530001
SNOMED	Burkitts tumor of lymph nodes of axilla AND/OR upper limb (disorder)	92511007
SNOMED	Secondary malignant neoplasm of skin of lip (disorder)	94564007
SNOMED	Malignant melanoma stage IIB (finding)	9.56391E+14
SNOMED	Secondary malignant neoplasm of olfactory nerve (disorder)	94451009
SNOMED	Secondary malignant neoplasm of large intestine and rectum (disorder)	448922007
SNOMED	Squamous cell carcinoma of gallbladder and extrahepatic biliary tract (disorder)	766978002
SNOMED	Malignant neoplasm of cerebral dura mater (disorder)	188312005
SNOMED	Aleukemic lymphoid leukemia in remission (disorder)	426248008
SNOMED	Carcinoma of upper labial sulcus (disorder)	254450000
SNOMED	Secondary malignant neoplasm of intestinal tract (disorder)	94346004
SNOMED	Secondary malignant neoplasm of parotid gland (disorder)	94474008
SNOMED	Leukemic reticuloendotheliosis of intrathoracic lymph nodes (disorder)	93146001
SNOMED	Primary malignant neoplasm of unknown site (disorder)	310504009
SNOMED	Primary clear cell adenocarcinoma of oropharynx (disorder)	707401000
SNOMED	Malignant tumor involving an organ by direct extension from vagina (disorder)	369601001
SNOMED	Malignant melanoma of skin of trunk (disorder)	93651008
SNOMED	Primary liposarcoma of male genital organ (disorder)	721578007
SNOMED	Psoralen and long-wave ultraviolet radiation therapy-associated squamous cell carcinoma (disorder)	403713003
SNOMED	Secondary malignant neoplasm of trigeminal nerve (disorder)	94646009
SNOMED	Hodgkins disease mixed cellularity of lymph nodes of head face AND/OR neck (disorder)	93506000
SNOMED	Malignant tumor of testis (disorder)	363449006
SNOMED	Malignant lymphoma of thyroid gland (disorder)	278051002
SNOMED	Malignant lymphoma of the eye region (disorder)	420519005
SNOMED	Primary malignant neoplasm of lip (disorder)	371996000
SNOMED	Malignant tumor involving uterine cervix by separate metastasis from ovary (disorder)	369501008
SNOMED	Primary malignant neuroendocrine neoplasm of duodenum (disorder)	721644006
SNOMED	Secondary malignant neoplasm of labia minora (disorder)	94362005

SNOMED	Secondary malignant neoplasm of lacrimal gland (disorder)	94364006
SNOMED	Malignant tumor involving left fallopian tube by direct extension from uterus (disorder)	369519004
SNOMED	Primary cutaneous large T-cell lymphoma (disorder)	402880009
SNOMED	Malignant tumor of base of tongue (disorder)	363376007
SNOMED	Secondary malignant neoplasm of laryngeal commissure (disorder)	94368009
SNOMED	Secondary malignant neoplasm of upper arm (disorder)	449633004
SNOMED	Secondary malignant neoplasm of larynx (disorder)	94370000
SNOMED	Secondary malignant neoplasm of lateral portion of floor of mouth (disorder)	94371001
SNOMED	Carcinoma anterior 2/3 tongue ventrum (disorder)	275395000
SNOMED	Secondary malignant neoplasm of lateral wall of oropharynx (disorder)	94373003
SNOMED	Malignant neoplasm of auditory tube middle ear and mastoid air cells (disorder)	187833006
SNOMED	Alveolar rhabdomyosarcoma (disorder)	404053004
SNOMED	Primary malignant neoplasm of mastoid air cells (disorder)	93887003
SNOMED	Secondary malignant neoplasm of lesser curvature of stomach (disorder)	94378007
SNOMED	Secondary malignant neoplasm of pericardium (disorder)	94484009
SNOMED	Aleukemic lymphoid leukemia (disorder)	188728002
SNOMED	Secondary malignant neoplasm of cuboid (disorder)	94269003
SNOMED	Primary malignant neoplasm of short bone of lower limb (disorder)	94003005
SNOMED	Local tumor spread (disorder)	255127006
SNOMED	Primary malignant neoplasm of lower leg (disorder)	449635006
SNOMED	Splenic marginal zone B-cell lymphoma (disorder)	763666008
SNOMED	Malignant melanoma of toe (disorder)	188076009
SNOMED	Secondary malignant neoplasm of lower outer quadrant of female breast (disorder)	94387003
SNOMED	Malignant neoplasm of Gartners duct (disorder)	188208002
SNOMED	Malignant tumor of body of penis (disorder)	188230001
SNOMED	Secondary malignant neoplasm of shoulder (disorder)	94537006
SNOMED	Malignant tumor of spinal cord extramedullary (disorder)	424549003
SNOMED	Secondary malignant neoplasm of lymph node (disorder)	94392001
SNOMED	Secondary malignant neoplasm of lymph nodes of face (disorder)	94393006
SNOMED	Subacute lymphoid leukemia in remission (disorder)	426370008
SNOMED	Secondary malignant neoplasm of ulna (disorder)	94650002
SNOMED	Large cell anaplastic lymphoma (disorder)	277637000
SNOMED	Small lymphocytic B-cell lymphoma involving skin (disorder)	404138009
SNOMED	Primary malignant neoplasm of prostate metastatic to bone (disorder)	712849003
SNOMED	Subacute myeloid leukemia (disorder)	188736006
SNOMED	Malignant optic glioma of adulthood (disorder)	703429003
SNOMED	Malignant granular cell tumor (disorder)	404041003
SNOMED	Neuroendocrine carcinoma of thymus (disorder)	716653001
SNOMED	Carcinoma of commissure of lip (disorder)	254404003
SNOMED	Mycosis fungoides of lymph nodes of axilla AND/OR upper limb (disorder)	94710006
SNOMED	Overlapping malignant neoplasm of vulva (disorder)	109885001
SNOMED	Mycosis fungoides of intrathoracic lymph nodes (disorder)	94709001
SNOMED	Primary malignant neoplasm of ribs and/or sternum and/or clavicle (disorder)	372107001
SNOMED	Hodgkins disease lymphocytic depletion of spleen (disorder)	188592008
SNOMED	Primary malignant neoplasm of right ovary (disorder)	369529006
SNOMED	Malignant tumor of female genital organ (disorder)	363514001
SNOMED	Secondary malignant neoplasm of metacarpal bone (disorder)	94411006
SNOMED	Aleukemic leukemia in remission (disorder)	426217000
SNOMED	Secondary malignant neoplasm of dorsal surface of tongue (disorder)	94273000
SNOMED	Malignant tumor of vagina (disorder)	363445000
SNOMED	Secondary malignant neoplasm of minor salivary gland (disorder)	94415002
SNOMED	Alpha heavy chain disease respiratory form (disorder)	25050002

SNOMED	Malignant tumor of trachea (disorder)	363432004
SNOMED	CD-30 positive T-immunoblastic cutaneous lymphoma (disorder)	404127009
SNOMED	Malignant histiocytosis of lymph nodes of axilla and upper limb (disorder)	188641006
SNOMED	Secondary malignant neoplasm of metatarsal bone (disorder)	94412004
SNOMED	Secondary malignant neoplasm of inguinal lymph nodes (disorder)	94339008
SNOMED	Malignant epithelial neoplasm of nose (disorder)	448988009
SNOMED	Malignant melanoma of thigh (disorder)	188069004
SNOMED	Secondary malignant neoplasm of muscle of lower limb (disorder)	94424006
SNOMED	Malignant epithelial neoplasm of mandible (disorder)	449487002
SNOMED	Malignant tumor of tympanic antrum (disorder)	187836003
SNOMED	Primary malignant neoplasm of soft tissues of perineum (disorder)	94060005
SNOMED	Acute monocytic leukemia FAB M5b (disorder)	413441006
SNOMED	Secondary malignant neoplasm of skin of face (disorder)	94554003
SNOMED	Non-small cell lung cancer positive for epidermal growth factor receptor expression (disorder)	426964009
SNOMED	Squamous cell carcinoma of bronchus (disorder)	254622008
SNOMED	Secondary malignant neoplasm of paraurethral glands (disorder)	94468008
SNOMED	Proliferating angioendotheliomatosis (disorder)	254792006
SNOMED	Malignant melanoma of skin (disorder)	93655004
SNOMED	Secondary malignant neoplasm of soft tissues (disorder)	94264008
SNOMED	Metastasis from malignant tumor of colon (disorder)	314998002
SNOMED	Primary malignant neuroendocrine neoplasm of ampulla of Vater (disorder)	733351008
SNOMED	Hepatosplenic gamma-delta cell lymphoma (disorder)	699657009
SNOMED	Primary malignant neoplasm of clavicle (disorder)	93757004
SNOMED	Epithelioid trophoblastic tumor (disorder)	609515005
SNOMED	Secondary malignant neoplasm of skin of hip (disorder)	94561004
SNOMED	Malignant melanoma of skin of back (disorder)	93214005
SNOMED	Secondary malignant neoplasm of soft tissues of neck (disorder)	94590005
SNOMED	Secondary malignant neoplasm of rectovaginal septum (disorder)	94511008
SNOMED	Malignant neoplasm of pyloric canal of stomach (disorder)	187738005
SNOMED	Primary malignant neoplasm of intra-abdominal organs (disorder)	93839008
SNOMED	Primary rhabdomyosarcoma of respiratory organ (disorder)	722511005
SNOMED	Malignant melanoma of skin of face (disorder)	93225001
SNOMED	Large cell carcinoma of lung TNM stage 3 (disorder)	424970000
SNOMED	Malignant tumor of urinary bladder (disorder)	399326009
SNOMED	Secondary malignant neoplasm of optic nerve (disorder)	94452002
SNOMED	Primary malignant neoplasm of articular cartilage (disorder)	128466006
SNOMED	Malignant melanoma of ethmoid sinus (disorder)	698285005
SNOMED	Choriocarcinoma of placenta (disorder)	448401007
SNOMED	Primary basaloid squamous cell carcinoma of oropharynx (disorder)	707579001
SNOMED	Leukemic infiltration of skin in myeloid leukemia (disorder)	404151004
SNOMED	Primary adenocarcinoma of submandibular gland (disorder)	737309000
SNOMED	Primary adenocarcinoma of cervix uteri (disorder)	1.84781E+14
SNOMED	Secondary malignant neoplasm of posterior wall of oropharynx (disorder)	94499009
SNOMED	Malignant tumor of tympanic cavity (disorder)	187835004
SNOMED	Secondary malignant neoplasm of paraganglion (disorder)	94462009
SNOMED	Secondary malignant neoplasm of gastrointestinal tract (disorder)	94313005
SNOMED	Sertoli cell tumor of testis (disorder)	278057003
SNOMED	Secondary malignant neoplasm of parametrium (disorder)	94465006
SNOMED	Malignant tumor of optic nerve (disorder)	363498002
SNOMED	Secondary malignant neoplasm of adrenal medulla (disorder)	94162004
SNOMED	Leukemic reticuloendotheliosis of lymph nodes of multiple sites (disorder)	93150008
SNOMED	Diffuse low grade B-cell lymphoma (disorder)	277616008

SNOMED	Primary malignant neoplasm of inguinal region (disorder)	93834003
SNOMED	Primary malignant neoplasm of blood vessel of upper limb (disorder)	93713003
SNOMED	Malignant epithelial neoplasm of nasal septum (disorder)	449417005
SNOMED	Malignant tumor of anterior commissure (disorder)	254509006
SNOMED	Siewert type III adenocarcinoma (disorder)	3.42561E+14
SNOMED	Malignant tumor of larynx (disorder)	363429002
SNOMED	Primary malignant neoplasm of intrathoracic organs (disorder)	93841009
SNOMED	Secondary malignant neoplasm of nose (disorder)	94445004
SNOMED	Glioblastoma multiforme of central nervous system (disorder)	1.47131E+14
SNOMED	Malignant tumor of nasal cavity and nasopharynx (disorder)	255074000
SNOMED	Secondary malignant neoplasm of major salivary gland (disorder)	94400003
SNOMED	Malignant neoplasm of endocervical canal (disorder)	188176007
SNOMED	Secondary malignant neoplasm of periadrenal tissue (disorder)	94482008
SNOMED	Leiomyosarcoma of orbit (disorder)	699355007
SNOMED	Overlapping squamous cell carcinoma of oropharynx (disorder)	707529004
SNOMED	Angiosarcoma of spleen (disorder)	187821001
SNOMED	Secondary malignant neoplasm of phalanx of foot (disorder)	94486006
SNOMED	Anaplastic lymphoma kinase positive anaplastic large cell lymphoma (disorder)	738770003
SNOMED	Secondary malignant neoplasm of second cuneiform bone of foot (disorder)	94533005
SNOMED	Secondary malignant neoplasm of vocal cord (disorder)	94679009
SNOMED	Secondary malignant neoplastic disease (disorder)	128462008
SNOMED	Secondary malignant neoplasm of subglottis (disorder)	94607007
SNOMED	Secondary malignant neoplasm of skin of neck (disorder)	94566009
SNOMED	Overlapping malignant neoplasm of accessory sinuses (disorder)	109366009
SNOMED	Malignant tumor of ill-defined site (disorder)	363357005
SNOMED	Malignant tumor of mesothelial tissue (disorder)	254824006
SNOMED	Secondary malignant neoplasm of zygomatic bone (disorder)	94683009
SNOMED	Secondary malignant neoplasm of posterior mediastinum (disorder)	94497006
SNOMED	Refractory neutropenia (disorder)	721303001
SNOMED	Secondary malignant neoplasm of pancreatic duct (disorder)	94460001
SNOMED	Primary malignant neoplasm of major salivary gland (disorder)	93883004
SNOMED	Secondary malignant neoplasm of jaw (disorder)	94356000
SNOMED	Malignant neoplasm of connective and soft tissues of lumbar spine (disorder)	188017009
SNOMED	Secondary malignant neoplasm of pelvic bone (disorder)	94478006
SNOMED	Poorly-differentiated neuroendocrine carcinoma of thymus (disorder)	717921000
SNOMED	Mixed follicular and papillary thyroid carcinoma (disorder)	255030002
SNOMED	Myelomonocytic leukemia (disorder)	188768003
SNOMED	Bronchioloalveolar carcinoma (disorder)	373627005
SNOMED	Granulosa cell tumor of ovary (disorder)	254863004
SNOMED	Adenoid cystic eccrine carcinoma of skin (disorder)	254711000
SNOMED	Secondary malignant neoplasm of rectouterine pouch (disorder)	94510009
SNOMED	Malignant neuroendocrine tumor (disorder)	1.33531E+14
SNOMED	Secondary malignant neoplasm of right lung (disorder)	3.53561E+14
SNOMED	Malignant epithelial neoplasm of urethra (disorder)	448954003
SNOMED	Monosomy 7 syndrome	307340003
SNOMED	Secondary malignant neoplasm of respiratory tract (disorder)	94515004
SNOMED	Secondary malignant neoplasm of retina (disorder)	94516003
SNOMED	Sarcoma of head and neck (disorder)	423812005
SNOMED	Malignant lipomatous tumor (disorder)	254828009
SNOMED	Leiomyosarcoma of stomach (disorder)	447785000
SNOMED	Squamous cell carcinoma of nasopharynx (disorder)	422691006
SNOMED	Hodgkins disease nodular sclerosis - mixed cellularity (disorder)	277611003

SNOMED	Primary malignant neoplasm of soft tissues of trunk (disorder)	94063007
SNOMED	Palate carcinoma (disorder)	274084007
SNOMED	Secondary malignant neoplasm of tail of pancreas (disorder)	94618007
SNOMED	Malignant tumor of anterior pillar of fauces (disorder)	254459004
SNOMED	Fibrosarcoma of spleen (disorder)	187822008
SNOMED	Carcinoma of sigmoid colon (disorder)	285312008
SNOMED	Secondary malignant neoplasm of scalene lymph nodes (disorder)	94528006
SNOMED	Malignant tumor involving bladder by separate metastasis from vagina (disorder)	369482001
SNOMED	Malignant neoplasm of sphincter of Oddi (disorder)	187786003
SNOMED	Secondary malignant neoplasm of scrotum (disorder)	94531007
SNOMED	Secondary malignant neoplasm of sebaceous gland (disorder)	94532000
SNOMED	Leiomyosarcoma of cardioesophageal junction (disorder)	449055006
SNOMED	Primary malignant neoplasm of glottis (disorder)	93816002
SNOMED	Secondary malignant neoplasm of vermilion border of upper lip (disorder)	94675003
SNOMED	Immunoglobulin A myeloma (disorder)	285420006
SNOMED	Primary malignant neoplasm of hypoglossal nerve (disorder)	93828005
SNOMED	Primary malignant neuroepithelial neoplasm of ciliary body (disorder)	735920008
SNOMED	Carcinoma of supraglottis (disorder)	372105009
SNOMED	Overlapping malignant neoplasm of major salivary gland (disorder)	109824000
SNOMED	Hodgkins disease mixed cellularity of extranodal AND/OR solid organ site (disorder)	93510002
SNOMED	Granulocytic sarcoma (disorder)	188738007
SNOMED	Secondary malignant neoplasm of skin of breast (disorder)	94544002
SNOMED	Secondary malignant neoplasm of skin of buttock (disorder)	94545001
SNOMED	Malignant neoplasm of central nervous system (disorder)	372062007
SNOMED	Secondary malignant neoplasm of skin of chest (disorder)	94547009
SNOMED	Secondary malignant neoplasm of skin of chin (disorder)	94548004
SNOMED	Malignant tumor of appendix (disorder)	363411007
SNOMED	Secondary malignant neoplasm of hard palate (disorder)	94324007
SNOMED	Malignant neoplasm of upper lobe bronchus or lung (disorder)	269464000
SNOMED	Amelanotic malignant melanoma of skin (disorder)	276751004
SNOMED	Hodgkins paragranuloma of spleen (disorder)	93545005
SNOMED	Malignant tumor of spermatic cord (disorder)	363453008
SNOMED	Secondary malignant neoplasm of skin of finger (disorder)	94555002
SNOMED	Carcinoma of gallbladder (disorder)	372140005
SNOMED	Follicular thyroid carcinoma (disorder)	255028004
SNOMED	Siewert type I adenocarcinoma (disorder)	3.42571E+14
SNOMED	Primary invasive pleomorphic lobular carcinoma of breast (disorder)	722524005
SNOMED	Mucinous cystadenocarcinoma of ovary (disorder)	254851009
SNOMED	Trichilemmal carcinoma (disorder)	403929003
SNOMED	Diffuse large B-cell lymphoma (disorder)	8.47741E+14
SNOMED	Secondary malignant neoplasm of flank (disorder)	94303002
SNOMED	Primary adenocarcinoma of middle ear (disorder)	721561003
SNOMED	Secondary malignant neoplasm of isthmus of uterus (disorder)	94355001
SNOMED	Desmoplastic malignant melanoma (disorder)	403924008
SNOMED	Acute myeloid leukaemia with 11q23 abnormality (disorder)	8.15361E+14
SNOMED	Secondary malignant neoplasm of skin of popliteal area (disorder)	94569002
SNOMED	Pleuropulmonary blastoma (disorder)	707670009
SNOMED	Hereditary diffuse carcinoma of stomach (disorder)	716859000
SNOMED	Malignant neoplasm of nasal conchae (disorder)	187830009
SNOMED	[X]Malignant neoplasm of urinary tract (disorder)	4.09171E+14
SNOMED	Malignant melanoma of rectum (disorder)	276822007
SNOMED	Malignant neoplasm of cerebral arachnoid mater (disorder)	188313000

SNOMED	Primary malignant neoplasm of uveal tract primary (disorder)	94128004
SNOMED	Secondary malignant neoplasm of skin of upper limb (disorder)	94577003
SNOMED	Secondary malignant neoplasm of epiglottis (disorder)	94284007
SNOMED	Carcinoma of upper third of esophagus (disorder)	254547001
SNOMED	Liver cell carcinoma (disorder)	109841003
SNOMED	Adenocarcinoma of esophagus (disorder)	276803003
SNOMED	Lymphoma of pyloric antrum of stomach (disorder)	447766003
SNOMED	Lymphangiosarcoma (disorder)	403986008
SNOMED	Malignant neoplasm of canthus (disorder)	310599006
SNOMED	Primary basaloid squamous cell carcinoma of hypopharynx (disorder)	707481002
SNOMED	Primary adenoid squamous cell carcinoma of hypopharynx (disorder)	707484005
SNOMED	Malignant neoplasm of eyelid (disorder)	231829006
SNOMED	Secondary malignant neoplasm of soft tissues of inguinal region (disorder)	94588009
SNOMED	Primary malignant neoplasm of hard palate (disorder)	371991005
SNOMED	Primary malignant neoplasm of bone of lower limb (disorder)	93722002
SNOMED	Pleuropulmonary blastoma type III (disorder)	707673006
SNOMED	Sarcoma of uterus (disorder)	254877001
SNOMED	Secondary malignant neoplasm of coccyx (disorder)	94259009
SNOMED	Malignant insulinoma (disorder)	713189001
SNOMED	Malignant tumor of lower third of esophagus (disorder)	187727005
SNOMED	Malignant tumor of cervix (disorder)	363354003
SNOMED	Primary malignant neoplasm of soft tissues of shoulder (disorder)	94061009
SNOMED	Anaplastic astrocytoma of brain (disorder)	277461004
SNOMED	Primary malignant neuroendocrine neoplasm of appendix (disorder)	721673009
SNOMED	Malignant neoplasm of nasal cavities middle ear and accessory sinuses (disorder)	187828007
SNOMED	Primary malignant neoplasm of gallbladder (disorder)	372139008
SNOMED	Undifferentiated nonkeratinizing squamous cell carcinoma of nasopharynx (disorder)	698048006
SNOMED	Primary high grade serous adenocarcinoma of ovary (disorder)	722685004
SNOMED	Local recurrence of malignant tumor of pancreas (disorder)	314964006
SNOMED	Secondary malignant neoplasm of sternum (disorder)	94605004
SNOMED	Primary salivary gland-type tumour of oropharynx (disorder)	707593008
SNOMED	Pulmonary tumor embolism (disorder)	233940007
SNOMED	Aggressive natural killer-cell leukemia (disorder)	721310007
SNOMED	Malignant tumor of choroid (disorder)	363466008
SNOMED	Secondary malignant neoplasm of submaxillary gland (disorder)	94610000
SNOMED	Overlapping primary malignant neoplasm of bone and articular cartilage of left upper limb (disorder)	3.54621E+14
SNOMED	Malignant tumor involving vulva by direct extension from uterine cervix (disorder)	369510000
SNOMED	Primary myoepithelial carcinoma of oropharynx (disorder)	707586009
SNOMED	Primary Ewing sarcoma of articular cartilage of pelvis (disorder)	723855004
SNOMED	Cancer en cuirasse (disorder)	254841008
SNOMED	Thoracic neuroblastoma (disorder)	281563002
SNOMED	Secondary malignant neoplasm of sweat gland (disorder)	94617002
SNOMED	Kaposis sarcoma - endemic (disorder)	403979000
SNOMED	Secondary malignant neoplasm of talus (disorder)	94619004
SNOMED	Secondary malignant neoplasm of central portion of female breast (disorder)	94244003
SNOMED	Metastatic malignant neoplasm to nasopharynx (disorder)	241861008
SNOMED	Follicular non-Hodgkins lymphoma of ovary (disorder)	449307001
SNOMED	Secondary malignant neoplasm of testis (disorder)	94623007
SNOMED	Yolk sac tumor (disorder)	404081005
SNOMED	Primary chondrosarcoma of bone of upper limb (disorder)	9.1031E+13
SNOMED	Primary squamous cell carcinoma of thymus (disorder)	1.05111E+14
SNOMED	Malignant neoplasm of spinal dura mater (disorder)	188317004

SNOMED	Primary squamous cell carcinoma of larynx (disorder)	707358000
SNOMED	Metastasis from malignant tumor of breast (disorder)	315004001
SNOMED	Secondary malignant neoplasm of third cuneiform bone of foot (disorder)	94630001
SNOMED	Refractory cytopenia with multilineage dysplasia (disorder)	415285009
SNOMED	Anaplastic astrocytoma of spinal cord (disorder)	370987005
SNOMED	Primary malignant neoplasm of brain (disorder)	93727008
SNOMED	Secondary malignant neoplasm of thyroid gland (disorder)	94634005
SNOMED	Verrucous squamous cell carcinoma (disorder)	403904009
SNOMED	Malignant neoplasm of prepylorus of stomach (disorder)	1.09029E+15
SNOMED	Familial cancer of breast (disorder)	254843006
SNOMED	Overlapping malignant neoplasm of colon and rectum (disorder)	9.6281E+13
SNOMED	CD-30 negative cutaneous T-cell lymphoma (disorder)	404128004
SNOMED	Primary malignant neuroendocrine neoplasm of ileum (disorder)	737313007
SNOMED	Secondary malignant neoplasm of trachea (disorder)	94641004
SNOMED	Primary malignant neoplasm of peripheral nerves of abdomen (disorder)	109943004
SNOMED	Metastasis to lung of unknown primary (disorder)	285604008
SNOMED	Follicular non-Hodgkins lymphoma of lung (disorder)	448672006
SNOMED	Secondary malignant neoplasm of left lung (disorder)	3.53741E+14
SNOMED	Malignant peripheral nerve sheath tumor (disorder)	404037002
SNOMED	Malignant neoplastic disease co-occurrent with human immunodeficiency virus infection (disorder)	713572001
SNOMED	Secondary malignant neoplasm of trochlear nerve (disorder)	94648005
SNOMED	Ring melanoma of ciliary body (disorder)	399660006
SNOMED	Burkitts lymphoma of lymph nodes of inguinal region and lower limb (disorder)	188514005
SNOMED	Medulloblastoma (disorder)	443333004
SNOMED	Malignant tumor of unknown origin or ill-defined site (disorder)	302817000
SNOMED	Secondary malignant neoplasm of upper inner quadrant of female breast (disorder)	94653000
SNOMED	Malignant neoplasm of cerebellum (disorder)	449420002
SNOMED	Secondary malignant neoplasm of upper outer quadrant of female breast (disorder)	94655007
SNOMED	Follicular lymphoma grade 3 (disorder)	8.47651E+14
SNOMED	Malignant Leydig cell tumor of testis (disorder)	278055006
SNOMED	Primary malignant neoplasm of frontal bone (disorder)	93806005
SNOMED	Secondary malignant neoplasm of ureter (disorder)	94659001
SNOMED	Secondary malignant neoplasm of ureteric orifice of urinary bladder (disorder)	94660006
SNOMED	Primary adenocarcinoma of endocervix (disorder)	2.08041E+14
SNOMED	Myeloid leukemia (disorder)	188732008
SNOMED	Primary adenocarcinoma of lower lobe of right lung (disorder)	1.07893E+15
SNOMED	Sarcoma of coccyx (disorder)	449208008
SNOMED	Lambda light chain myeloma (disorder)	313427003
SNOMED	Adenocarcinoma of rectosigmoid junction (disorder)	425178004
SNOMED	Secondary malignant neoplasm of uvula (disorder)	94667009
SNOMED	Pelvic neuroblastoma (disorder)	281564008
SNOMED	Secondary malignant neoplasm of vagus nerve (disorder)	94669007
SNOMED	Infiltrating lobular carcinoma of right female breast (disorder)	1.08034E+15
SNOMED	Balloon cell malignant melanoma (disorder)	403922007
SNOMED	Primary malignant neoplasm of spinal meninges (disorder)	94069006
SNOMED	Secondary malignant neoplasm of skin of toe (disorder)	94574005
SNOMED	Follicular non-Hodgkins lymphoma diffuse follicle center cell sub-type grade 2 (disorder)	702977001
SNOMED	Malignant glioma of eye (disorder)	2.34941E+14
SNOMED	Secondary malignant neoplasm of vestibule of mouth (disorder)	94676002
SNOMED	Secondary malignant neoplasm of muscle (disorder)	94432003
SNOMED	Malignant neoplasm of digestive system (disorder)	448675008
SNOMED	Secondary malignant neoplasm of right middle lobe of lung (disorder)	94523002

SNOMED	Liposarcoma of connective tissue (disorder)	448554008
SNOMED	Malignant tumor of posterior wall of hypopharynx (disorder)	303012000
SNOMED	Malignant neoplasm of superior wall of nasopharynx (disorder)	709031009
SNOMED	Malignant tumor involving right ovary by direct extension from endometrium (disorder)	369531002
SNOMED	Acute monocytic/monoblastic leukemia (disorder)	413442004
SNOMED	Primary adenocarcinoma of parametrium (disorder)	721568009
SNOMED	Hodgkins disease lymphocytic-histiocytic predominance of extranodal AND/OR solid organ site (disorder)	93501005
SNOMED	Squamous cell carcinoma of larynx (disorder)	405822008
SNOMED	Secondary malignant neoplasm of pelvic peritoneum (disorder)	94479003
SNOMED	Adenoid cystic carcinoma of oropharynx (disorder)	423318000
SNOMED	Nodular lymphoma of extranodal AND/OR solid organ site (disorder)	95194004
SNOMED	Secondary malignant neoplasm of glottis (disorder)	94318001
SNOMED	Secondary malignant neoplasm of oropharynx (disorder)	94454001
SNOMED	Primary malignant neoplasm of descending colon (disorder)	93771007
SNOMED	Secondary malignant neoplasm of bone of face (disorder)	94218003
SNOMED	Malignant tumor involving vasa deferentia by separate metastasis from prostate (disorder)	369484000
SNOMED	Small cell carcinoma of lung (disorder)	254632001
SNOMED	Malignant neoplasm of nervous system (disorder)	372063002
SNOMED	Malignant tumor of lower buccal sulcus (disorder)	187660002
SNOMED	Malignant melanoma of skin of perineum (disorder)	93644004
SNOMED	Primary malignant neoplasm of pineal gland (disorder)	93962006
SNOMED	Kaposis sarcoma of gastrointestinal tract (disorder)	109389001
SNOMED	Acute myeloid leukemia minimal differentiation FAB M0 (disorder)	359631009
SNOMED	Malignant tumor of pharyngeal recess (disorder)	187697007
SNOMED	Malignant neoplasm of soft tissues of lower leg (disorder)	1.09099E+15
SNOMED	Oat cell carcinoma of lung (disorder)	254633006
SNOMED	Atypical chronic myeloid leukemia (disorder)	277589003
SNOMED	Vulval verrucous carcinoma of Buschke-LÃƒ Â¶wenstein (disorder)	402912009
SNOMED	Leukemic reticuloendotheliosis of intra-abdominal lymph nodes (disorder)	93144003
SNOMED	Secondary malignant neoplasm of colon (disorder)	94260004
SNOMED	Burkitts tumor of extranodal AND/OR solid organ site (disorder)	92516002
SNOMED	Hodgkins disease lymphocytic-histiocytic predominance of spleen (disorder)	93500006
SNOMED	Scirrhous carcinoma of breast (disorder)	254839007
SNOMED	Reticulosarcoma of spleen (disorder)	95231000
SNOMED	SÃƒ Â©zarys disease of lymph nodes of head face AND/OR neck (disorder)	95260009
SNOMED	Primary malignant neoplasm of accessory sinus (disorder)	93659005
SNOMED	Malignant tumor of stomach (disorder)	363349007
SNOMED	Primary malignant neoplasm of breast upper inner quadrant (disorder)	373089009
SNOMED	Carcinoma of salivary gland type of breast (disorder)	716593008
SNOMED	Secondary malignant neoplasm of laryngeal aspect of aryepiglottic fold (disorder)	94366008
SNOMED	Acquired hemoglobin H disease	307343001
SNOMED	Mucosa-associated lymphoma (disorder)	277622004
SNOMED	Adenocarcinoma of non-pigmented epithelium of ciliary body (disorder)	255017003
SNOMED	Cutaneous leiomyosarcoma with granular cell change (disorder)	404047004
SNOMED	Malignant tumor of olfactory tract (disorder)	254969001
SNOMED	Malignant thymoma (disorder)	444596001
SNOMED	Essential thrombocythemia (disorder)	109994006
SNOMED	Pathological fracture due to metastatic bone disease	134421000
SNOMED	Secondary malignant neoplasm of long bone of upper limb (disorder)	94383004
SNOMED	Malignant neoplasm of carpal bone - trapezoid (disorder)	187942000
SNOMED	Secondary malignant neoplasm of skin of foot (disorder)	94556001
SNOMED	Secondary malignant neoplasm of pisiform bone of hand (disorder)	94490008

SNOMED	Anogenital verrucous carcinoma of Buschke-LÃƒ Â¶wenstein (disorder)	402910001
SNOMED	Secondary malignant neoplasm of female breast (disorder)	94297009
SNOMED	Malignant tumor involving ureter by direct extension from bladder (disorder)	369463005
SNOMED	Primary adenocarcinoma of body of stomach (disorder)	6.81631E+14
SNOMED	Astrocytoma of cerebrum (disorder)	9.9131E+13
SNOMED	Metastasis to vertebral column of unknown primary (disorder)	285619009
SNOMED	Familial renal cell carcinoma (disorder)	717736007
SNOMED	Papillary cystadenocarcinoma of kidney (disorder)	254917006
SNOMED	Malignant tumor of thymus (disorder)	363434003
SNOMED	Secondary malignant neoplasm of tibia (disorder)	94635006
SNOMED	Malignant neoplasm of mastoid (disorder)	448299004
SNOMED	T-cell prolymphocytic leukemia (disorder)	277567002
SNOMED	Malignant neoplasm of cornu of corpus uteri (disorder)	188190005
SNOMED	Secondary malignant neoplasm of retroperitoneal lymph nodes (disorder)	94519005
SNOMED	Malignant tumor involving right ovary by direct extension from uterus (disorder)	369566000
SNOMED	Burkitts lymphoma of lymph nodes of axilla and upper limb (disorder)	188513004
SNOMED	Malignant neoplastic disease in pregnancy (disorder)	1.07499E+16
SNOMED	Malignant tumor involving left ovary by separate metastasis from uterine cervix (disorder)	369563008
SNOMED	Malignant tumor involving left ovary by separate metastasis from right ovary (disorder)	369562003
SNOMED	Lymphoma of kidney (disorder)	236513009
SNOMED	Secondary malignant neoplasm of fibula (disorder)	94301000
SNOMED	Malignant tumor of spleen (disorder)	363499005
SNOMED	Metastasis to eye of unknown primary (disorder)	285642002
SNOMED	Primary low grade serous adenocarcinoma of ovary (disorder)	722684000
SNOMED	Acute monoblastic leukemia (disorder)	277601005
SNOMED	Early gastric cancer (disorder)	276809004
SNOMED	Siewert type I adenocarcinoma of esophagogastric junction (disorder)	438946002
SNOMED	Primary malignant neoplasm of areola of male breast (disorder)	93681000
SNOMED	Primary malignant neoplasm of independent multiple sites (disorder)	109359008
SNOMED	Malignant neoplasm of fourth metatarsal bone (disorder)	187976009
SNOMED	Malignant tumor of lingual tonsil (disorder)	363377003
SNOMED	Mixed cell type lymphosarcoma of intrathoracic lymph nodes (disorder)	94688000
SNOMED	Chronic lymphoid leukemia in remission (disorder)	92813000
SNOMED	Primary malignant neoplasm of cerebral ventricle (disorder)	93748005
SNOMED	Primary neuroendocrine carcinoma of overlapping lesion of stomach (disorder)	721642005
SNOMED	Malignant tumor involving rectum by separate metastasis from uterine cervix (disorder)	369459003
SNOMED	Malignant tumor involving left fallopian tube by separate metastasis from right fallopian tube (disorder)	369544007
SNOMED	Malignant neoplasm of frontal lobe (disorder)	363467004
SNOMED	Malignant melanoma of skin of vulva (disorder)	448300007
SNOMED	Malignant meningitis (disorder)	230156002
SNOMED	Diffuse non-Hodgkins lymphoma of prostate (disorder)	448213004
SNOMED	Malignant tumor involving an organ by separate metastasis from fallopian tube (disorder)	369604009
SNOMED	Malignant mast cell tumor of lymph nodes of head face AND/OR neck (disorder)	93204001
SNOMED	SÃƒ Â©zarys disease of lymph nodes of head face and neck (disorder)	188630009
SNOMED	Primary glioblastoma multiforme of frontal lobe (disorder)	6.84911E+14
SNOMED	Malignant tumor involving right ovary by direct extension from left ovary (disorder)	369533004
SNOMED	Acute megakaryoblastic leukemia (disorder)	277602003
SNOMED	Primary squamous cell carcinoma of parotid gland (disorder)	722674001
SNOMED	Acute myeloid leukemia with nucleophosmin 1 somatic mutation (disorder)	763309005
SNOMED	Secondary malignant neoplasm of mediastinum (disorder)	94409002
SNOMED	IgD monoclonal gammopathy of uncertain significance	285428004
SNOMED	Lymphoreticular tumor	277606000

SNOMED	Secondary malignant neoplasm of paravaginal lymph nodes (disorder)	94469000
SNOMED	Secondary malignant neoplasm of bilateral adrenal glands (disorder)	1.22466E+16
SNOMED	Mast cell leukemia (disorder)	110002002
SNOMED	Primary squamous cell carcinoma of posterior wall of hypopharynx (disorder)	707686002
SNOMED	Primary malignant neoplasm of left ovary (disorder)	369522002
SNOMED	Secondary malignant neoplasm of right fallopian tube (disorder)	369521009
SNOMED	Secondary malignant neoplasm of peritoneum (disorder)	94627008
SNOMED	Primary malignant neuroendocrine neoplasm of body of stomach (disorder)	721636006
SNOMED	Sebaceous adenocarcinoma (disorder)	307599002
SNOMED	Secondary malignant neoplasm of infraclavicular lymph nodes (disorder)	94338000
SNOMED	Malignant melanoma of unknown origin (disorder)	424190005
SNOMED	Diffuse non-Hodgkins lymphoma of central nervous system (disorder)	449221001
SNOMED	Malignant tumor of ureter (disorder)	363458004
SNOMED	Secondary malignant neoplasm of lymph nodes of neck (disorder)	94397007
SNOMED	Subacute myeloid leukemia in remission (disorder)	425749006
SNOMED	Malignant tumor involving vulva by direct extension from uterus (disorder)	369511001
SNOMED	Secondary malignant neoplasm of skin of shoulder (disorder)	94571002
SNOMED	Secondary malignant neoplasm of parathyroid gland (disorder)	94467003
SNOMED	Malignant tumor involving vulva by direct extension from fallopian tube (disorder)	369508002
SNOMED	Malignant tumor of oral cavity (disorder)	363505006
SNOMED	Secondary malignant neoplasm of left upper lobe of lung (disorder)	94376006
SNOMED	Malignant tumor of peritoneum (disorder)	363492001
SNOMED	Hodgkins disease of lymph nodes of axilla AND/OR upper limb (disorder)	93523009
SNOMED	Malignant neoplasm of connective tissue of orbit (disorder)	188268004
SNOMED	Squamous cell carcinoma of rectum (disorder)	766979005
SNOMED	Hodgkins disease lymphocytic-histiocytic predominance of lymph nodes of multiple sites (disorder)	188562004
SNOMED	Primary malignant neuroepithelial neoplasm of retina (disorder)	735916009
SNOMED	Primary malignant neoplasm of thymus (disorder)	94096009
SNOMED	Primary squamous cell carcinoma of respiratory system (disorder)	733358002
SNOMED	Immunoproliferative small intestinal disease (disorder)	109985000
SNOMED	Malignant tumor involving uterine corpus by direct extension from vagina (disorder)	369496007
SNOMED	Lymphoid leukemia (disorder)	188725004
SNOMED	Secondary malignant neoplasm of diaphragm (disorder)	94272005
SNOMED	Primary malignant neoplasm of lingual tonsil (disorder)	93868009
SNOMED	Primary malignant neoplasm of lower limb (disorder)	93875005
SNOMED	Malignant neoplasm of spinal arachnoid mater (disorder)	188318009
SNOMED	Lymphoma of greater curvature of stomach (disorder)	447805007
SNOMED	Malignant epithelial tumor of ovary (disorder)	254849005
SNOMED	Recurrent squamous cell carcinoma (disorder)	719052006
SNOMED	Carcinoma of lower lobe bronchus or lung (disorder)	372111007
SNOMED	Malignant tumor involving prostate by separate metastasis from bladder (disorder)	369486003
SNOMED	Cholangiocarcinoma of biliary tract (disorder)	312104005
SNOMED	Undifferentiated carcinoma of stomach (disorder)	766757006
SNOMED	Secondary malignant neoplasm of ilium (disorder)	94337005
SNOMED	Secondary malignant neoplasm of inferior epigastric lymph nodes (disorder)	1.09018E+15
SNOMED	Hodgkins paragranuloma of lymph nodes of inguinal region AND/OR lower limb (disorder)	93543003
SNOMED	Primary osteosarcoma of articular cartilage of limb (disorder)	723851008
SNOMED	Primary clear cell squamous cell carcinoma of trachea (disorder)	707389001
SNOMED	Metastatic squamous cell carcinoma (disorder)	403906006
SNOMED	Malignant mesothelioma of pleura (disorder)	254645002
SNOMED	Malignant neoplasm of pons (disorder)	188298006
SNOMED	Secondary malignant neoplasm of extrahepatic bile ducts (disorder)	94291005

SNOMED	Hodgkins sarcoma of lymph nodes of inguinal region AND/OR lower limb (disorder)	93552007
SNOMED	Malignant neoplasm after immunosuppressive therapy (disorder)	708054009
SNOMED	Secondary malignant neoplasm of forearm (disorder)	94306005
SNOMED	Acquired renal cyst with neoplastic change	236441006
SNOMED	Malignant neoplasm of midbrain (disorder)	188297001
SNOMED	Carcinoma of lower third of esophagus (disorder)	254551004
SNOMED	Secondary malignant neoplasm of skin of eyebrow (disorder)	94552004
SNOMED	Malignant neoplasm of endocervix (disorder)	372097009
SNOMED	Malignant tumor of opening of auditory tube (disorder)	187698002
SNOMED	Malignant tumor involving urethra by direct extension from bladder (disorder)	369465003
SNOMED	Follicular non-Hodgkins lymphoma of soft tissue (disorder)	448317000
SNOMED	Papillary renal cell carcinoma (disorder)	733608000
SNOMED	Adenoma malignum (disorder)	385478001
SNOMED	Adenocarcinoma of lacrimal gland (disorder)	254988008
SNOMED	Local recurrence of malignant tumor of esophagus (disorder)	314960002
SNOMED	Secondary malignant neoplasm of vermilion border of lip (disorder)	94673005
SNOMED	Malignant tumor of minor salivary gland (disorder)	363485006
SNOMED	Malignant tumor involving rectum by separate metastasis from fallopian tube (disorder)	369456005
SNOMED	Intraepithelial squamous cell carcinoma of anogenital region (disorder)	767544007
SNOMED	Leukemic reticuloendotheliosis of lymph nodes of head face AND/OR neck (disorder)	93148000
SNOMED	Malignant tumor involving rectum by direct extension from uterus (disorder)	369453002
SNOMED	Hodgkins granuloma of lymph nodes of axilla AND/OR upper limb (disorder)	93532006
SNOMED	Undifferentiated carcinoma of nasal sinus (disorder)	697993003
SNOMED	Malignant neoplasm of carpal bone - scaphoid (disorder)	187937008
SNOMED	Malignant tumor of head of pancreas (disorder)	363419009
SNOMED	Letterer-Siwe disease of lymph nodes of multiple sites (disorder)	93139005
SNOMED	Primary malignant neoplasm of gastrointestinal tract (disorder)	363745004
SNOMED	Rhabdomyosarcoma of connective or soft tissue (disorder)	423610004
SNOMED	Primary squamous cell carcinoma of base of tongue (disorder)	722672002
SNOMED	Secondary malignant neoplasm of splenic flexure of colon (disorder)	94604000
SNOMED	Malignant tumor of male genital organ (disorder)	363515000
SNOMED	Malignant epithelial neoplasm of upper rectum (disorder)	448994001
SNOMED	Malignant neoplasm of soft tissue of orbit (disorder)	448669004
SNOMED	Secondary adenocarcinoma of bilateral lungs (disorder)	1.59562E+16
SNOMED	Primary malignant neoplasm of labia minora (disorder)	93851005
SNOMED	Secondary malignant neoplasm of skin of wrist (disorder)	94578008
SNOMED	Malignant tumor of lower gingiva (disorder)	363384006
SNOMED	Overlapping malignant neoplasm of bronchus and lung (disorder)	109371002
SNOMED	Diffuse malignant lymphoma - large cleaved cell (disorder)	277628000
SNOMED	Sarcoma of soft tissue (disorder)	424952003
SNOMED	Follicular lymphoma cutaneous follicle centre (disorder)	1.09186E+15
SNOMED	Recurrent primary malignant neoplasm of vulva (disorder)	7.1111E+13
SNOMED	Multiple malignancy (disorder)	363500001
SNOMED	Primary malignant neoplasm of kidney (disorder)	93849006
SNOMED	Marginal zone lymphoma (disorder)	447100004
SNOMED	Primary malignant neoplasm of blood vessel of head (disorder)	93699008
SNOMED	Malignant tumor of soft tissue of abdomen (disorder)	363496003
SNOMED	Malignant tumor involving left fallopian tube by separate metastasis from uterus (disorder)	369545008
SNOMED	Gamma heavy chain disease (disorder)	109984001
SNOMED	Secondary malignant neoplasm of tongue (disorder)	94638008
SNOMED	Refractory anemia with excess blasts-1 (disorder)	415283002
SNOMED	Carcinoma of upper lobe bronchus or lung (disorder)	372136001

SNOMED	Primary adenocarcinoma of paraurethral gland (disorder)	733362008
SNOMED	Malignant neoplasm of jaw (disorder)	1.09092E+15
SNOMED	Primary malignant neoplasm of male genital organ (disorder)	93885006
SNOMED	Peripheral neuroepithelioma (disorder)	254764001
SNOMED	Hodgkins paragranuloma of intrathoracic lymph nodes (disorder)	188524002
SNOMED	Secondary malignant neoplasm of appendix (disorder)	94175004
SNOMED	Malignant tumor of nasopharyngeal soft palate surface (disorder)	187702003
SNOMED	Malignant neoplastic disease (disorder)	363346000
SNOMED	Malignant tumor of pituitary gland (disorder)	363482009
SNOMED	Malignant tumor of parathyroid gland (disorder)	363481002
SNOMED	Primary malignant neoplasm of junctional zone of tongue (disorder)	93848003
SNOMED	Hodgkins disease nodular sclerosis of extranodal AND/OR solid organ site (disorder)	93519001
SNOMED	Metastatic malignant neoplasm to dome of urinary bladder (disorder)	359785002
SNOMED	Carcinoma of cervix stage 0 (disorder)	254889004
SNOMED	Malignant neoplasm of cerebral meninges (disorder)	363474009
SNOMED	Malignant neoplasm of brainstem (disorder)	363473003
SNOMED	Primary malignant neoplasm of dome of urinary bladder (disorder)	449803009
SNOMED	Mycosis fungoides (disorder)	118618005
SNOMED	Primary malignant neoplasm of esophagogastric junction (disorder)	722533007
SNOMED	Metastasis from malignant tumor of liver (disorder)	315000005
SNOMED	Metastasis to lymph node from adenocarcinoma (disorder)	1.681E+12
SNOMED	Adenocarcinoma of endometrium (disorder)	123845008
SNOMED	Primary signet ring cell carcinoma of trachea (disorder)	707377003
SNOMED	Acute myeloid leukemia due to recurrent genetic abnormality (disorder)	721305008
SNOMED	Squamous cell carcinoma of bronchus in right lower lobe (disorder)	313355000
SNOMED	Malignant tumor of orbit (disorder)	363462005
SNOMED	Malignant tumor involving uterine corpus by separate metastasis from vagina (disorder)	369578001
SNOMED	Malignant melanoma of back (disorder)	310498001
SNOMED	Nodal marginal zone B-cell lymphoma (disorder)	726721002
SNOMED	Primary papillary squamous cell carcinoma of oropharynx (disorder)	707581004
SNOMED	Primary malignant neoplasm of urachus (disorder)	94120006
SNOMED	Hodgkins disease mixed cellularity of intrathoracic lymph nodes (disorder)	188576003
SNOMED	Kaposis sarcoma of multiple organs (disorder)	109392002
SNOMED	Primary squamous cell carcinoma of lingual tonsil (disorder)	722673007
SNOMED	Primary malignant neoplasm of uterus (disorder)	1.07085E+16
SNOMED	Transglottic malignant neoplasm of larynx (disorder)	448509007
SNOMED	Malignant optic glioma (disorder)	404664002
SNOMED	Monocytoid B-cell lymphoma (disorder)	277623009
SNOMED	Primary malignant neoplasm of hypopharyngeal aspect of aryepiglottic fold (disorder)	93829002
SNOMED	Secondary malignant neoplasm of lacrimal gland duct (disorder)	423384009
SNOMED	Renal medullary carcinoma (disorder)	765095002
SNOMED	Plasma cell leukemia disease (disorder)	95210003
SNOMED	Squamous cell carcinoma of floor of mouth (disorder)	276954004
SNOMED	Carcinoma of greater curve of stomach (disorder)	254567005
SNOMED	Malignant tumor of soft tissue of face (disorder)	363440005
SNOMED	Primary malignant neoplasm of nervous system (disorder)	93923002
SNOMED	Secondary malignant neoplasm of cartilage of nose (disorder)	94241006
SNOMED	Malignant tumor aryepiglottic fold - hypopharyngeal aspect (disorder)	187708004
SNOMED	Malignant melanoma of skin of finger (disorder)	93226000
SNOMED	Malignant tumor of heart (disorder)	363435002
SNOMED	Carcinoma of endocrine pancreas (disorder)	254612002
SNOMED	Primary malignant neoplasm of the peritoneum (disorder)	372016004

SNOMED	Malignant melanoma of perianal skin (disorder)	93209006
SNOMED	Ewing sarcoma of bone structure of upper limb (disorder)	9.1041E+13
SNOMED	Peripheral T-cell lymphoma (disorder)	109977009
SNOMED	Lymphoplasmacytic B-cell lymphoma nodal/systemic with skin involvement (disorder)	404149003
SNOMED	Malignant neoplasm of connective and soft tissue of axilla (disorder)	188010006
SNOMED	Primary malignant neoplasm of posterior wall of nasopharynx (disorder)	93970001
SNOMED	Eccrine porocarcinoma of skin (disorder)	254708001
SNOMED	Invasive carcinoma of uterine cervix co-occurrent with human immunodeficiency virus infection (disorder)	733834006
SNOMED	Secondary malignant neoplasm of neck (disorder)	94441008
SNOMED	Intraductal papillary mucinous carcinoma in situ of pancreas (disorder)	473419009
SNOMED	Malignant tumor of pancreas (disorder)	363418001
SNOMED	Malignant tumor of body of stomach (disorder)	187742008
SNOMED	Carcinoma of middle third of esophagus (disorder)	254549003
SNOMED	Primary verrucous carcinoma of trachea (disorder)	707494000
SNOMED	Metastasis involving oral cavity (disorder)	404094007
SNOMED	Malignant tumor of ampulla of Vater (disorder)	363417006
SNOMED	Squamous cell carcinoma of vagina (disorder)	1.05121E+14
SNOMED	Malignant mesothelioma of mesentery (disorder)	109856007
SNOMED	Leiomyosarcoma of small intestine (disorder)	716651004
SNOMED	Secondary malignant neoplasm of large intestine (disorder)	94365007
SNOMED	Malignant tumor of rectosigmoid junction (disorder)	363414004
SNOMED	Sarcoma of peritoneum (disorder)	447783007
SNOMED	Malignant tumor of sigmoid colon (disorder)	363410008
SNOMED	Chronic lymphocytic leukemia genetic mutation variant (disorder)	725437002
SNOMED	Diffuse malignant lymphoma - centroblastic polymorphic (disorder)	277632006
SNOMED	Malignant tumor of hepatic flexure (disorder)	363407001
SNOMED	Primary urothelial carcinoma of paraurethral gland (disorder)	733135005
SNOMED	Juvenile myelomonocytic leukemia (disorder)	445227008
SNOMED	Malignant tumor involving vasa deferentia by direct extension from prostate (disorder)	369483006
SNOMED	Primary malignant neoplasm of superior wall of nasopharynx (disorder)	94078000
SNOMED	Secondary malignant neoplasm of first cuneiform bone of foot (disorder)	94302007
SNOMED	Malignant neoplasm of lateral wall of urinary bladder (disorder)	188241004
SNOMED	Malignant tumor of postcricoid region (disorder)	363400004
SNOMED	Sarcoma of sacrum (disorder)	448377009
SNOMED	Malignant lymphoma of testis (disorder)	277664004
SNOMED	Overlapping malignant neoplasm of nasopharynx (disorder)	109367000
SNOMED	Precursor B-cell lymphoblastic lymphoma involving skin (disorder)	404137004
SNOMED	Sarcoma of kidney (disorder)	254918001
SNOMED	Glandular malignant peripheral nerve sheath tumor (disorder)	699659007
SNOMED	Malignant tumor of tonsil (disorder)	363393007
SNOMED	Malignant tumor of oropharynx (disorder)	363392002
SNOMED	Malignant tumor involving rectum by direct extension from fallopian tube (disorder)	369449004
SNOMED	Malignant neoplasm of peripheral nerves of upper limb including shoulder (disorder)	188323009
SNOMED	Primary malignant epithelial neoplasm of nasopharynx (disorder)	722529000
SNOMED	Primary sarcoma of peritoneum (disorder)	722519007
SNOMED	Malignant neoplasm of third metatarsal bone (disorder)	187975008
SNOMED	Leukemic reticuloendotheliosis of lymph nodes of axilla AND/OR upper limb (disorder)	93147005
SNOMED	Malignant tumor of floor of mouth (disorder)	363385007
SNOMED	Secondary malignant neoplasm of vertebral column (disorder)	94602001
SNOMED	Malignant tumor of upper gingiva (disorder)	363383000
SNOMED	Malignant tumor of gum (disorder)	363382005
SNOMED	Primary giant cell sarcoma of peritoneum (disorder)	722445001

SNOMED	Malignant tumor of submandibular gland (disorder)	363380002
SNOMED	Malignant tumor of parotid gland (disorder)	363379000
SNOMED	SÃƒ Â©zarys disease of lymph nodes of inguinal region and lower limb (disorder)	188634000
SNOMED	Malignant carcinoid tumor (disorder)	445238008
SNOMED	Carcinoma of main bronchus (disorder)	372120003
SNOMED	Chronic lymphoid leukemia disease (disorder)	92814006
SNOMED	Chordoma of sacrum (disorder)	447730004
SNOMED	Secondary malignant neoplasm of lip (disorder)	94380001
SNOMED	Carcinoma of breast - lower inner quadrant (disorder)	286894008
SNOMED	Primary malignant neoplasm of labia majora (disorder)	93850006
SNOMED	Secondary malignant neoplasm of skin of knee (disorder)	94562006
SNOMED	Secondary malignant neoplasm of skin of thigh (disorder)	94573004
SNOMED	Metastatic malignant neoplasm to apex of urinary bladder (disorder)	359782004
SNOMED	Refractory acute lymphoid leukemia (disorder)	1.2311E+13
SNOMED	Basophilic leukemia (disorder)	307592006
SNOMED	Secondary malignant neoplasm of male genital organ (disorder)	94402006
SNOMED	Primary giant cell carcinoma of larynx (disorder)	707660007
SNOMED	Inherited acute myeloid leukemia (disorder)	764940002
SNOMED	Malignant tumor of middle ear (disorder)	363359008
SNOMED	Renal cell carcinoma (disorder)	702391001
SNOMED	Diffuse non-Hodgkins lymphoma undifferentiated (disorder)	109964000
SNOMED	Malignant germ cell neoplasm of posterior mediastinum (disorder)	447705002
SNOMED	Secondary malignant neoplasm of tibial lymph nodes (disorder)	94636007
SNOMED	Malignant tumor of gallbladder (disorder)	363353009
SNOMED	Hodgkins paragranuloma of intrapelvic lymph nodes (disorder)	188529007
SNOMED	Chondrosarcoma (disorder)	443520009
SNOMED	Malignant lymphoma - lymphocytic intermediate differentiation (disorder)	274905008
SNOMED	Secondary malignant neoplasm of obturator lymph nodes (disorder)	94446003
SNOMED	Overlapping malignant neoplasm of eye and adnexa primary (disorder)	109948008
SNOMED	Leiomyosarcoma of cardia of stomach (disorder)	447707005
SNOMED	Chronic myeloid leukemia disease (disorder)	92818009
SNOMED	Metastasis to skin of unknown primary (disorder)	285631006
SNOMED	Nodular lymphoma of lymph nodes of multiple sites (disorder)	95192000
SNOMED	Angiocentric natural killer/T-cell malignant lymphoma involving skin (disorder)	404135007
SNOMED	Tongue carcinoma (disorder)	269516007
SNOMED	Primary mucinous cystadenocarcinoma of lung (disorder)	707595001
SNOMED	Polymorphous low grade adenocarcinoma of salivary gland (disorder)	423038006
SNOMED	Reticulosarcoma of lymph nodes of head face and neck (disorder)	188489006
SNOMED	Primary malignant neoplasm of soft tissues of abdomen (disorder)	94050001
SNOMED	Malignant tumor of branchial cleft (disorder)	363396004
SNOMED	Carcinoma of tail of pancreas (disorder)	363369002
SNOMED	Follicular malignant lymphoma - mixed cell type (disorder)	277624003
SNOMED	Acute myeloid leukemia and myelodysplastic syndrome related to topoisomerase type 2 inhibitor (disorder)	766046007
SNOMED	Malignant tumor of lung parenchyma (disorder)	254625005
SNOMED	Low grade endometrial stromal sarcoma (disorder)	699357004
SNOMED	Paratesticular malignant neoplasm (disorder)	699317002
SNOMED	Acute promyelocytic leukemia FAB M3 (disorder)	110004001
SNOMED	Primary adenosquamous carcinoma of lung (disorder)	707405009
SNOMED	Primary malignant neoplasm of cheek (disorder)	93753000
SNOMED	Malignant tumor of palate (disorder)	363390005
SNOMED	Malignant neoplasm of flank (disorder)	1.0903E+15
SNOMED	Secondary malignant neoplasm of brain stem (disorder)	94224009

SNOMED	Malignant neoplasm of connective and soft tissue of abdominal wall (disorder)	188016000
SNOMED	Non-secretory myeloma (disorder)	277580004
SNOMED	Malignant melanoma of skin of left upper limb (disorder)	3.52201E+14
SNOMED	Primary malignant neoplasm of soft tissue of left lower limb (disorder)	3.52321E+14
SNOMED	Polymorphic lymphoproliferative disorder following transplant (disorder)	762316003
SNOMED	Secondary malignant neoplasm of lymph nodes of head (disorder)	94394000
SNOMED	Chronic phase chronic myeloid leukemia (disorder)	413847001
SNOMED	Secondary malignant neoplasm of sclera (disorder)	94530008
SNOMED	Hodgkins disease mixed cellularity of lymph nodes of head face and neck (disorder)	188575004
SNOMED	Secondary malignant neoplasm of Meckels diverticulum (disorder)	94407000
SNOMED	Malignant tumor of kidney parenchyma (disorder)	188250002
SNOMED	Malignant carcinoid tumor of thymus (disorder)	726652005
SNOMED	Carcinoma of parotid gland (disorder)	254462001
SNOMED	B-cell acute lymphoblastic leukemia (disorder)	277571004
SNOMED	Primary malignant neoplasm of trapezium (disorder)	372021001
SNOMED	Primary malignant neoplasm of ventral surface of tongue (disorder)	94134006
SNOMED	Malignant tumor involving seminal vesicle by separate metastasis from prostate (disorder)	369492009
SNOMED	Primary malignant neoplasm of diaphragm (disorder)	93772000
SNOMED	Malignant tumor involving vulva by direct extension from endometrium (disorder)	369507007
SNOMED	Donor derived melanoma (disorder)	720587009
SNOMED	Metastasis to brain of unknown primary (disorder)	285641009
SNOMED	Metastasis from malignant tumor of pancreas (disorder)	314999005
SNOMED	Primary osteosarcoma (disorder)	735680008
SNOMED	Primary malignant neoplasm of muscle of neck (disorder)	93906006
SNOMED	Primary malignant neoplasm of cerebrum (disorder)	93749002
SNOMED	Malignant neuroma of mediastinum (disorder)	278044006
SNOMED	Secondary malignant neoplasm of adnexa of skin (disorder)	94159002
SNOMED	Secondary malignant neoplasm of eye (disorder)	94292003
SNOMED	Acute lymphoblastic leukemia transitional pre-B-cell (disorder)	371012000
SNOMED	Metastasis from malignant tumor of adrenal gland (disorder)	314991008
SNOMED	Mantle cell lymphoma of spleen (disorder)	441559006
SNOMED	Primary malignant neoplasm of upper lobe of right lung (disorder)	93993007
SNOMED	Metastasis from malignant tumor of skin (disorder)	314988008
SNOMED	Metastasis from malignant melanoma of skin (disorder)	314987003
SNOMED	Malignant mesothelioma of pericardium (disorder)	109383000
SNOMED	Mast cell malignancy (disorder)	397009000
SNOMED	Malignant tumor of thyroid gland (disorder)	363478007
SNOMED	Primary malignant neoplasm of vagus nerve (disorder)	94131003
SNOMED	Local recurrence of malignant tumor of prostate (disorder)	314969001
SNOMED	Rhabdomyosarcoma of vulva and vagina (disorder)	766759009
SNOMED	Glioblastoma multiforme (disorder)	393563007
SNOMED	Primary carcinoma of liver (disorder)	187769009
SNOMED	Mixed seminoma teratoma of testis (disorder)	278491007
SNOMED	Malignant tumor of cervical part of esophagus (disorder)	187722004
SNOMED	Secondary malignant neoplasm of tonsil (disorder)	94458003
SNOMED	Secondary malignant neoplasm of skin of external auditory canal (disorder)	94551006
SNOMED	Primary squamous cell carcinoma of overlapping lesion of esophagus (disorder)	721621008
SNOMED	Malignant tumor involving right fallopian tube by direct extension from endometrium (disorder)	369547000
SNOMED	Epithelioid cell sarcoma of skin (disorder)	254800003
SNOMED	Malignant sacral teratoma (disorder)	416842003
SNOMED	Mucoepidermoid carcinoma of salivary gland (disorder)	423708008
SNOMED	Langerhans cell sarcoma (disorder)	724649000

SNOMED	Acute lymphoid leukemia relapse (disorder)	1.2301E+13
SNOMED	SÃƒ Â©zarys disease of lymph nodes of axilla and upper limb (disorder)	188633006
SNOMED	Leukemic infiltrate of choroid (disorder)	314408000
SNOMED	Squamous non-small cell lung cancer (disorder)	723301009
SNOMED	Malignant neoplasm of connective and soft tissue of finger (disorder)	187996001
SNOMED	Secondary malignant neoplasm of soft palate (disorder)	94581003
SNOMED	Squamous cell carcinoma of right lung (disorder)	1.2241E+16
SNOMED	Nodular malignant melanoma of skin (disorder)	254731001
SNOMED	Malignant tumor of paraurethral gland (disorder)	363460002
SNOMED	Malignant melanoma of skin of eyelid (disorder)	93224002
SNOMED	Tibial adamantinoma (disorder)	281702006
SNOMED	Secondary malignant neoplasm of soft tissues of perineum (disorder)	94592002
SNOMED	Malignant lymphoma of spleen (disorder)	93198004
SNOMED	Malignant melanoma of skin of upper limb (disorder)	93653006
SNOMED	Malignant tumor involving vagina by direct extension from fallopian tube (disorder)	369506003
SNOMED	Carcinoma of thoracic part of esophagus (disorder)	254539001
SNOMED	Carcinoma of splenic flexure (disorder)	312115000
SNOMED	Squamous cell carcinoma of stomach (disorder)	766980008
SNOMED	Malignant neoplasm of costal cartilage (disorder)	187925006
SNOMED	Carcinoma of transverse colon (disorder)	312112002
SNOMED	Primary adenocarcinoma of uterine ligament (disorder)	721569001
SNOMED	Overlapping malignant neoplasm of oropharynx (disorder)	109832008
SNOMED	Primary chondrosarcoma of bone of limb (disorder)	723843005
SNOMED	Malignant tumor of soft tissue of back (disorder)	310526005
SNOMED	Malignant lymphoma of lymph nodes of inguinal region AND/OR lower limb (disorder)	93196000
SNOMED	Carcinoma of fundus of stomach (disorder)	254555008
SNOMED	Primary malignant neoplasm of descended testis (disorder)	109876001
SNOMED	Melanotic malignant nerve sheath tumor (disorder)	404039004
SNOMED	Malignant neoplasm of occipital lobe (disorder)	363470000
SNOMED	Malignant tumor involving right fallopian tube by direct extension from vagina (disorder)	369552005
SNOMED	Kaposis sarcoma of conjunctiva (disorder)	255114007
SNOMED	Secondary malignant neoplasm of endocrine gland (disorder)	94280003
SNOMED	Malignant lymphoma centroblastic type follicular (disorder)	307647008
SNOMED	Metastasis to vagina of unknown primary (disorder)	285638000
SNOMED	Malignant lymphoma centroblastic-centrocytic follicular (disorder)	307637005
SNOMED	Superficial malignant fibrous histiocytoma of skin (disorder)	404015009
SNOMED	Hodgkins disease nodular sclerosis - cellular phase (disorder)	307635002
SNOMED	Metastasis from malignant tumor of bronchus (disorder)	315005000
SNOMED	Hodgkins disease lymphocytic depletion diffuse fibrosis (disorder)	307633009
SNOMED	Malignant tumor of labial mucosa (disorder)	187622006
SNOMED	Diffuse malignant lymphoma - centroblastic-centrocytic (disorder)	307624007
SNOMED	Carcinosarcoma of uterus (disorder)	702369008
SNOMED	Primary spindle cell carcinoma of trachea (disorder)	707394001
SNOMED	Langerhans cell histiocytosis (disorder)	65399007
SNOMED	Pilomatrix carcinoma of skin (disorder)	307610008
SNOMED	Malignant lymphoma convoluted cell type (disorder)	303017006
SNOMED	Ewings sarcoma of bone (disorder)	307608006
SNOMED	Secondary malignant neoplasm of intrathoracic organs (disorder)	94352003
SNOMED	Sarcoma of mesentery (disorder)	448258005
SNOMED	Hodgkins paragranuloma of lymph nodes of multiple sites (disorder)	188531003
SNOMED	Primary malignant neoplasm of lower lobe of left lung (disorder)	93864006
SNOMED	Mycosis fungoides of lymph nodes of inguinal region AND/OR lower limb (disorder)	94712003

SNOMED	Malignant tumor of hypopharynx (disorder)	363399006
SNOMED	Squamous cell carcinoma of mouth (disorder)	307502000
SNOMED	Malignant tumor of ear nose and throat (disorder)	255073006
SNOMED	Atypical hairy cell leukemia (disorder)	307341004
SNOMED	Hodgkins sarcoma of intrapelvic lymph nodes (disorder)	93548007
SNOMED	Malignant neoplasm of endometrium of corpus uteri (disorder)	188192002
SNOMED	SÃƒ Â©zarys disease of intrathoracic lymph nodes (disorder)	188631008
SNOMED	Malignant neoplasm of breast lower inner quadrant (disorder)	373080008
SNOMED	Secondary malignant neoplasm of pararectal lymph nodes (disorder)	94466007
SNOMED	Malignant neoplasm of peripheral nerve of pelvis (disorder)	188327005
SNOMED	Primary malignant neoplasm of transverse colon (disorder)	94105000
SNOMED	Adenoid cystic carcinoma of lacrimal gland (disorder)	254987003
SNOMED	Malignant neoplasm of connective and soft tissue of buttock (disorder)	188020001
SNOMED	Primary malignant neuroendocrine neoplasm of esophagus (disorder)	721626003
SNOMED	Primary malignant clear cell tumor of ovary (disorder)	416274001
SNOMED	Secondary malignant neoplasm of hilus of lung (disorder)	94329002
SNOMED	Primary cutaneous diffuse large cell B-cell lymphoma (disorder)	404144008
SNOMED	Squamous cell carcinoma of buccal mucosa (disorder)	254437001
SNOMED	Primary synovial sarcoma of soft tissue of limb (disorder)	721579004
SNOMED	Synovial sarcoma (disorder)	302851001
SNOMED	Malignant neoplasm of nasolacrimal duct (disorder)	188274004
SNOMED	Nodular malignant lymphoma lymphocytic - intermediate differentiation (disorder)	302848008
SNOMED	Primary malignant neoplasm of left fallopian tube (disorder)	369513003
SNOMED	Secondary nodes - axilla/arm (disorder)	269616004
SNOMED	Juvenile chronic myeloid leukemia (disorder)	277587001
SNOMED	Malignant tumor involving uterine cervix by direct extension from vagina (disorder)	369499000
SNOMED	Lentigo maligna melanoma (disorder)	302837001
SNOMED	Plasmacytoma	10639003
SNOMED	Primary malignant melanoma of anus (disorder)	723281005
SNOMED	Angiosarcoma of skin (disorder)	254794007
SNOMED	Malignant tumor of frenum of lip (disorder)	302815008
SNOMED	Extranodal natural killer/T-cell lymphoma nasal type (disorder)	414166008
SNOMED	Smoldering chronic lymphocytic leukemia (disorder)	430338009
SNOMED	Malignant epithelial neoplasm of hypopharynx (disorder)	448665005
SNOMED	Adenocarcinoma in adenomatous polyp (disorder)	2.9421E+13
SNOMED	Primary malignant neoplasm of oculomotor nerve (disorder)	93929003
SNOMED	Secondary malignant neoplasm of thigh (disorder)	94629006
SNOMED	Carcinoma of body of pancreas (disorder)	363368005
SNOMED	Primary carcinoma of maxillary sinus (disorder)	707347009
SNOMED	Squamous cell carcinoma of esophagus (disorder)	276804009
SNOMED	Familial polycythemia vera (disorder)	127066000
SNOMED	Squamous cell carcinoma of shoulder (disorder)	448931007
SNOMED	Malignant neoplasm of alveolus of mandible (disorder)	449578008
SNOMED	Carcinoma of lower limb bones (disorder)	286890004
SNOMED	Carcinoma of genitourinary organ (disorder)	255066001
SNOMED	Primary malignant neoplasm of sigmoid colon (disorder)	94006002
SNOMED	Acute myelomonocytic leukemia - eosinophilic variant (disorder)	285839005
SNOMED	Adenocarcinoma of anal canal (disorder)	764845008
SNOMED	Carcinoma of hard palate (disorder)	254434008
SNOMED	Malignant tumor of pituitary and hypothalamus (disorder)	255112006
SNOMED	Squamous cell carcinoma of vulva due to lichen sclerosus (disorder)	403890009
SNOMED	Primary malignant neoplasm of tarsal bone (disorder)	94084002

SNOMED	Malignant tumor involving right fallopian tube by separate metastasis from vagina (disorder)	369558009
SNOMED	Malignant melanoma of trunk (disorder)	269579005
SNOMED	Primary adenocarcinoma of upper third of esophagus (disorder)	721622001
SNOMED	Squamous cell carcinoma of pharynx (disorder)	408649007
SNOMED	Malignant tumor of urachus (disorder)	363456000
SNOMED	Metastasis to ovary of unknown primary (disorder)	285637005
SNOMED	T-cell acute lymphoblastic leukemia in remission (disorder)	427642009
SNOMED	Secondary malignant neoplasm of blood vessel of popliteal space (disorder)	94204005
SNOMED	Primary chondrosarcoma of articular cartilage of limb (disorder)	723844004
SNOMED	Botryoid rhabdomyosarcoma (disorder)	404052009
SNOMED	Secondary malignant neoplasm of acoustic nerve (disorder)	94156009
SNOMED	Metastasis to bone of unknown primary (disorder)	285618001
SNOMED	Metastasis to retroperitoneum of unknown primary (disorder)	285617006
SNOMED	Primary malignant neoplasm of peripheral nerves of hip (disorder)	109939003
SNOMED	Adamantinoma of femur (disorder)	433067002
SNOMED	Adenosarcoma of uterus (disorder)	447390000
SNOMED	Malignant tumor involving bladder by direct extension from ovary (disorder)	369471009
SNOMED	Metastasis to rectum of unknown primary (disorder)	285612000
SNOMED	Primary malignant neoplasm of foot (disorder)	93803002
SNOMED	Primary malignant neoplasm of vulva (disorder)	94143002
SNOMED	Metastasis to small intestine of unknown primary (disorder)	285609003
SNOMED	Malignant neoplasm of carpal bone - trapezium (disorder)	187941007
SNOMED	Hodgkins granuloma of lymph nodes of head face and neck (disorder)	188534006
SNOMED	Primary mucinous adenocarcinoma of ovary (disorder)	733361001
SNOMED	Metastasis to pleura of unknown primary (disorder)	285605009
SNOMED	Malignant epithelial neoplasm of uterus (disorder)	446022000
SNOMED	Metastasis to bronchus of unknown primary (disorder)	285603002
SNOMED	Malignant granulosa cell tumor of testis (disorder)	702405001
SNOMED	Carcinoma of colon (disorder)	269533000
SNOMED	Malignant neoplasm of endocrine gland (disorder)	371982006
SNOMED	Adenocarcinoma of lower esophagus (disorder)	449153001
SNOMED	IgM monoclonal gammopathy of uncertain significance	285426000
SNOMED	IgG monoclonal gammopathy of uncertain significance	285424002
SNOMED	Glassy cell carcinoma of cervix uteri (disorder)	766930002
SNOMED	Malignant neoplasm of anterior epiglottis (disorder)	187681002
SNOMED	Parapsoriasis lichenoides (disorder)	16341002
SNOMED	Secondary malignant neoplasm of nasal bone (disorder)	94435001
SNOMED	Primary giant cell carcinoma of hypopharynx (disorder)	707487003
SNOMED	Splenic diffuse red pulp small B-cell lymphoma (disorder)	763884007
SNOMED	Malignant tumor involving bladder by separate metastasis from endometrium (disorder)	369476004
SNOMED	Adenocarcinoma carcinomatosis (disorder)	423595004
SNOMED	Primary squamous cell carcinoma of trachea (disorder)	707388009
SNOMED	Primary malignant neoplasm of fallopian tube (disorder)	371987000
SNOMED	Primary rhabdomyosarcoma of pharynx (disorder)	722510006
SNOMED	Primary leiomyosarcoma (disorder)	423807009
SNOMED	Hodgkins disease nodular sclerosis of lymph nodes of inguinal region AND/OR lower limb (disorder)	93516008
SNOMED	Malignant lymphoma - lymphoplasmacytic (disorder)	307623001
SNOMED	Malignant infiltration of oral cavity by underlying tumor (disorder)	404090003
SNOMED	[X]Malignant neoplasm of thyroid and other endocrine glands (disorder)	4.08911E+14
SNOMED	Malignant neoplasm of olfactory bulb (disorder)	188308004
SNOMED	Malignant neoplasm of visceral pleura (disorder)	449308006
SNOMED	Malignant neoplasm of brain (disorder)	428061005

SNOMED	Primary malignant neoplasm of first cuneiform bone of foot (disorder)	93800004
SNOMED	Primary malignant neuroendocrine of distal bile duct (disorder)	733350009
SNOMED	Secondary malignant neoplasm of urinary bladder neck (disorder)	94662003
SNOMED	Primary choriocarcinoma of testis (disorder)	721603005
SNOMED	Malignant lymphoma of breast (disorder)	278052009
SNOMED	Primary malignant neoplasm of vocal cord (disorder)	372030009
SNOMED	Carcinoma of breast - upper outer quadrant (disorder)	286895009
SNOMED	Primary malignant neoplasm of femur (disorder)	93798006
SNOMED	Secondary malignant neoplasm of vallecula (disorder)	94670008
SNOMED	Primary adenocarcinoma of intestinal tract (disorder)	9.9111E+13
SNOMED	Primary adenocarcinoma of overlapping lesion of accessory sinuses (disorder)	721606002
SNOMED	Rhabdomyosarcoma of bladder (disorder)	278024000
SNOMED	Secondary malignant neoplasm of bronchus of right upper lobe (disorder)	94232001
SNOMED	Secondary malignant neoplasm of cranial nerve (disorder)	94266005
SNOMED	Malignant tumor involving right ovary by separate metastasis from left ovary (disorder)	369570008
SNOMED	Secondary malignant neoplasm of soft tissues of lower limb (disorder)	94589001
SNOMED	Malignant melanoma of palatine arch (disorder)	698044008
SNOMED	Primary undifferentiated carcinoma of thyroid gland (disorder)	724553007
SNOMED	Malignant neoplasm of posterior pharynx (disorder)	187709007
SNOMED	Secondary malignant neoplasm of ischium (disorder)	94353008
SNOMED	Overlapping malignant neoplasm of male genital organs (disorder)	109874003
SNOMED	Localized skin involvement by breast carcinoma (disorder)	403458008
SNOMED	Primary mucinous carcinoma of uterine adnexa (disorder)	722687007
SNOMED	Malignant neoplasm of augmented bladder (disorder)	702467006
SNOMED	Malignant melanoma of skin of eyebrow (disorder)	93223008
SNOMED	Primary adenocarcinoma of transverse colon (disorder)	721696009
SNOMED	Mucinous carcinoma of breast (disorder)	444712000
SNOMED	Malignant infiltration of peripheral nerve plexus (disorder)	255129009
SNOMED	Malignant tumor of pyloric antrum (disorder)	187740000
SNOMED	Primary malignant neuroepitheliomatous neoplasm of peripheral nerve (disorder)	724805000
SNOMED	Malignant tumor of corpus spongiosum (disorder)	276419004
SNOMED	Follicular low grade B-cell lymphoma (disorder)	277618009
SNOMED	Spindle cell malignant melanoma (disorder)	403923002
SNOMED	Secondary malignant neoplasm of submandibular lymph nodes (disorder)	94609005
SNOMED	Malignant lymphoma follicular center cell (disorder)	303055001
SNOMED	Primary malignant neoplasm of presacral region (disorder)	93973004
SNOMED	Malignant tumor involving uterine cervix by direct extension from ovary (disorder)	369498008
SNOMED	Malignant neoplasm of lip oral cavity and/or pharynx (disorder)	271323007
SNOMED	Malignant neoplasm of genital labia (disorder)	1.09028E+15
SNOMED	Primary clear cell squamous cell carcinoma of lung (disorder)	707453008
SNOMED	Metastasis to soft tissue of unknown primary (disorder)	285633009
SNOMED	Null cell acute lymphoblastic leukemia (disorder)	277574007
SNOMED	Tumor lysis syndrome (disorder)	277605001
SNOMED	Specific skin infiltration in Hodgkins disease (disorder)	404157000
SNOMED	Diaphyseal medullary stenosis with bone malignancy (disorder)	389216001
SNOMED	Malignant tumor involving right fallopian tube by separate metastasis from uterine cervix (disorder)	369556008
SNOMED	Myelodysplastic syndrome with isolated del(5q) (disorder)	277597005
SNOMED	Secondary malignant neoplasm of axillary tail of breast (disorder)	372093008
SNOMED	Malignant tumor of anus (disorder)	363490009
SNOMED	Angioimmunoblastic T-cell lymphoma with dysproteinaemia (disorder)	1.09024E+15
SNOMED	Malignant neoplasm of mucosa of trachea (disorder)	187854004
SNOMED	Malignant neoplasm of thyroid cartilage (disorder)	187846001

SNOMED	Secondary malignant neoplasm of lower limb (disorder)	94386007
SNOMED	Hodgkins disease nodular sclerosis (disorder)	118608000
SNOMED	Malignant germ cell neoplasm of anterior mediastinum (disorder)	447708000
SNOMED	Common acute lymphoblastic leukemia (disorder)	277573001
SNOMED	T-cell acute lymphoblastic leukemia (disorder)	277575008
SNOMED	Burkitts lymphoma of lymph nodes of multiple sites (disorder)	188517003
SNOMED	Secondary malignant neoplasm of liver (disorder)	94381002
SNOMED	Metastasis to trachea of unknown primary (disorder)	285598005
SNOMED	Mast cell malignancy of lymph nodes of head face and neck (disorder)	188662007
SNOMED	Lymphoma of lower esophagus (disorder)	449053004
SNOMED	Secondary malignant neoplasm of skin of axilla (disorder)	94542003
SNOMED	Monoclonal paraproteinemia	267440005
SNOMED	Malignant neoplasm of connective and soft tissue upper arm (disorder)	187993009
SNOMED	Squamous cell carcinoma of bronchus in right upper lobe (disorder)	313357008
SNOMED	Squamous cell carcinoma of cornea (disorder)	255008003
SNOMED	Primary infiltrating duct carcinoma of oropharynx (disorder)	707592003
SNOMED	Primary adenocarcinoma of palate (disorder)	721556000
SNOMED	Primary malignant neoplasm of para-aortic body (disorder)	93940006
SNOMED	Secondary malignant neoplasm of muscle of abdomen (disorder)	94418000
SNOMED	Malignant tumor involving uterine corpus by separate metastasis from bladder (disorder)	369502001
SNOMED	Dermatofibrosarcoma protuberans of skin of shoulder (disorder)	447710003
SNOMED	Hodgkins disease of intrathoracic lymph nodes (disorder)	93522004
SNOMED	Adenocarcinoma of prostate (disorder)	399490008
SNOMED	Malignant tumor of anterior two-thirds of tongue - ventral surface (disorder)	187640005
SNOMED	Lymphomatoid papulosis (disorder)	31047003
SNOMED	Metastasis to uterus of unknown primary (disorder)	285635002
SNOMED	Secondary malignant neoplasm of bronchopulmonary lymph nodes (disorder)	94227002
SNOMED	Secondary malignant neoplasm of lumbar vertebral column (disorder)	94389000
SNOMED	Primary malignant neoplasm of muscle of hip (disorder)	93903003
SNOMED	Primary cerebral lymphoma (disorder)	276836002
SNOMED	Inflammatory malignant fibrous histiocytoma of skin (disorder)	404017001
SNOMED	Secondary malignant neoplasm of inner aspect of upper lip (disorder)	94343007
SNOMED	Primary malignant neoplasm of blood vessel of abdomen (disorder)	93690007
SNOMED	Primary adenocarcinoma of biliary tract (disorder)	733354000
SNOMED	Primary rhabdomyosarcoma of male genital organ (disorder)	721574009
SNOMED	Pheochromocytoma (disorder)	302835009
SNOMED	Primary leiomyosarcoma of peritoneum (disorder)	722514002
SNOMED	Secondary malignant neoplasm of skin of ear (disorder)	94549007
SNOMED	Malignant neoplasm of trunk	1.09031E+15
SNOMED	Core binding factor acute myeloid leukemia (disorder)	702446006
SNOMED	Carcinoma breast - lower outer quadrant (disorder)	286896005
SNOMED	Malignant epithelial neoplasm of cheek (disorder)	447949005
SNOMED	Secondary malignant neoplasm of blood vessel of neck (disorder)	94201002
SNOMED	Malignant tumor of fibrous tissue (disorder)	276797002
SNOMED	Astroblastoma of brain (disorder)	762457009
SNOMED	Secondary malignant neoplasm of calcaneus (disorder)	94236003
SNOMED	Malignant neoplasm of the pouch of Douglas (disorder)	187814001
SNOMED	Infiltrating ductal carcinoma of central portion of right female breast (disorder)	1.08019E+15
SNOMED	Primary malignant neoplasm of inner aspect of upper lip (disorder)	93837005
SNOMED	Malignant neoplasm of upper limb bones and scapula (disorder)	372131006
SNOMED	Pleuropulmonary blastoma type I (disorder)	707671008
SNOMED	Malignant tumor involving left ovary by direct extension from fallopian tube (disorder)	369525000

SNOMED	Malignant neoplasm of back	1.09032E+15
SNOMED	Primary malignant neoplasm of common bile duct (disorder)	93763008
SNOMED	Malignant tumor of splenic flexure (disorder)	363413005
SNOMED	Primary squamous cell carcinoma of oropharynx (disorder)	707585008
SNOMED	Primary malignant neoplasm of epididymis (disorder)	93783009
SNOMED	Primary malignant neoplasm of paraurethral glands (disorder)	93944002
SNOMED	Malignant tumor of ascending colon (disorder)	363412000
SNOMED	Primary squamous cell carcinoma of postcricoid region (disorder)	707703001
SNOMED	Malignant tumor of axilla (disorder)	363502009
SNOMED	Mycosis fungoides of intrapelvic lymph nodes (disorder)	94708009
SNOMED	Seminoma of descended testis (disorder)	313429000
SNOMED	Malignant tumor of lower labial mucosa (disorder)	271568003
SNOMED	Malignant tumor of frenum of lower lip (disorder)	187614004
SNOMED	Malignant neoplasm of bone, connective tissue, skin and breast	271467005
SNOMED	Malignant neoplasm of skeletal system (disorder)	443679004
SNOMED	Malignant melanoma of auricle (ear) (disorder)	188033007
SNOMED	Osteosarcoma limb anomalies erythroid macrocytosis syndrome (disorder)	733064004
SNOMED	Secondary nodes - inguinal/leg (disorder)	269617008
SNOMED	Primary adenocarcinoma of pancreas (disorder)	1.651E+12
SNOMED	Primary malignant neoplasm of ischium (disorder)	93842002
SNOMED	Malignant tumor involving vagina by direct extension from uterine cervix (disorder)	369580007
SNOMED	Local recurrence of malignant tumor of cervix (disorder)	314970000
SNOMED	Malignant melanoma of head and neck (disorder)	269578002
SNOMED	Malignant neoplasm of spinal meninges (disorder)	363476006
SNOMED	Secondary malignant neoplasm of anterior two-thirds of tongue (disorder)	94169008
SNOMED	Primary giant cell carcinoma of trachea (disorder)	707392002
SNOMED	Overlapping malignant neoplasm of rectum anus and anal canal (disorder)	109839004
SNOMED	Secondary malignant neoplasm of back (disorder)	94183005
SNOMED	Metastasis to thymus of unknown primary (disorder)	285608006
SNOMED	Secondary malignant neoplasm of respiratory and digestive systems (disorder)	269473008
SNOMED	Reticulosarcoma of intra-abdominal lymph nodes (disorder)	95224004
SNOMED	Carcinoma of vagina (disorder)	254893005
SNOMED	Malignant fibrous histiocytoma of skin (disorder)	404014008
SNOMED	Primary malignant neoplasm of muscle of buttock (disorder)	93900000
SNOMED	Primary adenocarcinoma of colon (disorder)	1.701E+12
SNOMED	Primary malignant neoplasm of areola of female breast (disorder)	93680004
SNOMED	Erythrodermic mycosis fungoides (disorder)	404114005
SNOMED	Primary squamous cell carcinoma of subglottis (disorder)	707576008
SNOMED	Mast cell malignancy of lymph nodes of inguinal region and lower limb (disorder)	188666005
SNOMED	Malignant fibromatous neoplasm (disorder)	443250000
SNOMED	Adrenal neuroblastoma (disorder)	281562007
SNOMED	Odontogenic ghost cell carcinoma (disorder)	134312002
SNOMED	Primary cutaneous T-cell lymphoma (disorder)	400122007
SNOMED	Metastasis to nervous system and eye (disorder)	255123005
SNOMED	Multiple primary malignant melanomata (disorder)	402564006
SNOMED	Malignant melanoma of nasal cavity (disorder)	698040004
SNOMED	Light chain monoclonal gammopathy of uncertain significance	285430002
SNOMED	Kaposis sarcoma of cornea (disorder)	255115008
SNOMED	Primary adenocarcinoma of overlapping lesion of esophagus (disorder)	721624000
SNOMED	Diffuse large B-cell lymphoma (nodal/systemic with skin involvement) (disorder)	404148006
SNOMED	Myeloid and/or lymphoid neoplasm associated with platelet derived growth factor receptor alpha rearrangement (disorder)	738527001
SNOMED	Adenocarcinoma of bladder (disorder)	255110003

SNOMED	Malignant tumor of endocardium (disorder)	363436001
SNOMED	Follicular non-Hodgkins lymphoma of testis (disorder)	449418000
SNOMED	Lymphosarcoma of intra-abdominal lymph nodes (disorder)	188502002
SNOMED	Angioendotheliomatosis (disorder)	255102004
SNOMED	Cystadenocarcinoma of kidney (disorder)	254916002
SNOMED	Prolymphocytic lymphosarcoma (disorder)	307622006
SNOMED	Hypernephroma (disorder)	188251003
SNOMED	Malignant neoplasm of carpal bones (disorder)	255090000
SNOMED	Secondary malignant neoplasm of supraclavicular lymph nodes (disorder)	94614009
SNOMED	Malignant tumor involving left fallopian tube by direct extension from endometrium (disorder)	369515005
SNOMED	Malignant tumor involving vulva by direct extension from ovary (disorder)	369509005
SNOMED	Squamous cell carcinoma of anal margin (disorder)	255084004
SNOMED	Malignant lymphoma of lymph nodes (disorder)	127220001
SNOMED	Carcinoma of cecum (disorder)	255081007
SNOMED	Overlapping malignant neoplasm of uterine cervix (disorder)	109880006
SNOMED	Hodgkins disease lymphocytic depletion of intrathoracic lymph nodes (disorder)	188586002
SNOMED	Primary malignant neoplasm of left kidney (disorder)	3.54351E+14
SNOMED	Glioblastoma multiforme of spinal cord (disorder)	276829003
SNOMED	Chronic neutrophilic leukemia (disorder)	188734009
SNOMED	Malignant neoplasm of thalamus (disorder)	188287005
SNOMED	Squamous cell carcinoma of eyelid (disorder)	231831002
SNOMED	Malignant neoplasm of lower lobe bronchus (disorder)	187869003
SNOMED	Lymphoma of retina (disorder)	232075002
SNOMED	Primary malignant neoplasm of body of pancreas (disorder)	93715005
SNOMED	Large cell carcinoma of lung TNM stage 2 (disorder)	423050000
SNOMED	Primary malignant neoplasm of rectum (disorder)	93984006
SNOMED	[X]Malignant neoplasm of ill-defined, secondary and unspecified sites	4.62341E+14
SNOMED	Secondary malignant neoplasm of lateral wall of nasopharynx (disorder)	94372008
SNOMED	Lymphoma of colon (disorder)	1.33751E+14
SNOMED	Medullary thyroid carcinoma (disorder)	255032005
SNOMED	Malignant tumor of Waldeyers ring (disorder)	187716008
SNOMED	Malignant tumor of laryngeal ventricle (disorder)	254526000
SNOMED	Hodgkins disease mixed cellularity of lymph nodes of inguinal region and lower limb (disorder)	188579005
SNOMED	Malignant epithelial neoplasm of fundus of uterus (disorder)	449054005
SNOMED	Erythroleukemia FAB M6 in remission (disorder)	426642002
SNOMED	Malignant neoplasm of ectopic pancreatic tissue (disorder)	187798008
SNOMED	Primary solid carcinoma of trachea (disorder)	707384006
SNOMED	Metastasis from malignant tumor of rectum (disorder)	314997007
SNOMED	Malignant neoplasm of crystalline lens (disorder)	188265001
SNOMED	Malignant white blood cell disorder (disorder)	277543005
SNOMED	Malignant melanoma of conjunctiva (disorder)	255004001
SNOMED	Squamous cell carcinoma of conjunctiva (disorder)	255003007
SNOMED	Follicular non-Hodgkins lymphoma of skin (disorder)	448865007
SNOMED	Primary Ewing sarcoma of bone of limb (disorder)	723853006
SNOMED	Precursor T-cell lymphoblastic lymphoma (disorder)	421246008
SNOMED	Malignant tumor of upper buccal sulcus (disorder)	187659007
SNOMED	Primary mediastinal (thymic) large B-cell lymphoma (disorder)	444910004
SNOMED	Carcinoma ex pleomorphic adenoma of lacrimal gland (disorder)	254989000
SNOMED	Secondary malignant neoplasm of urachus (disorder)	94658009
SNOMED	Non-Hodgkin lymphoma associated with Human immunodeficiency virus infection (disorder)	442537007
SNOMED	Secondary malignant neoplasm of placenta (disorder)	94492000
SNOMED	Malignant tumor of spinal nerve and sheath (disorder)	254983004

SNOMED	Malignant tumor of soft palate (disorder)	363388009
SNOMED	Linitis plastica of stomach (disorder)	721629005
SNOMED	Malignant tumor of optic nerve sheath (disorder)	254974009
SNOMED	Primary malignant neoplasm of right middle lobe of lung (disorder)	93992002
SNOMED	Secondary malignant neoplasm of gingival mucosa (disorder)	94314004
SNOMED	Dermatofibrosarcoma protuberans (disorder)	276799004
SNOMED	Secondary malignant neoplasm of ectopic female breast tissue (disorder)	94276008
SNOMED	Malignant melanoma of upper arm (disorder)	188061001
SNOMED	Astrocytoma of spinal cord (disorder)	254948003
SNOMED	Secondary malignant neoplasm of urinary system (disorder)	94663008
SNOMED	Malignant glioma of brainstem (disorder)	424276002
SNOMED	Malignant teratoma of undescended testis (disorder)	417417007
SNOMED	Malignant neoplasm of middle lobe bronchus (disorder)	187865009
SNOMED	Malignant histiocytosis involving skin (disorder)	404169008
SNOMED	Secondary malignant neoplasm of mesenteric lymph nodes (disorder)	94410007
SNOMED	Malignant tumor involving bladder by direct extension from vagina (disorder)	369475000
SNOMED	Smoldering myeloma (disorder)	413587002
SNOMED	Malignant tumor of choroid plexus (disorder)	188292007
SNOMED	Astrocytoma of brain stem (disorder)	1.07581E+14
SNOMED	B-cell chronic lymphocytic leukemia (disorder)	277473004
SNOMED	Malignant melanoma of vulva (disorder)	254896002
SNOMED	Malignant skin tumor with apocrine differentiation (disorder)	254726003
SNOMED	Malignant neoplasm of genitourinary organ (& [carcinoma])	188172009
SNOMED	Systemic Epstein-Barr virus positive T-cell lymphoproliferative disease of childhood (disorder)	721311006
SNOMED	Secondary malignant neoplasm of hypogastric lymph nodes (disorder)	94330007
SNOMED	Psoralen and long-wave ultraviolet radiation therapy-associated malignant melanoma (disorder)	403714009
SNOMED	Metastasis from malignant tumor of kidney (disorder)	314996003
SNOMED	Malignant tumor of upper third of esophagus (disorder)	187725002
SNOMED	Late gastric cancer (disorder)	276810009
SNOMED	Endodermal sinus tumor of ovary (disorder)	254876005
SNOMED	Dysgerminoma of ovary (disorder)	254874008
SNOMED	Primary malignant neoplasm of thoracic esophagus (disorder)	372017008
SNOMED	Primary malignant neoplasm of bronchus (disorder)	93734005
SNOMED	Primary squamous cell carcinoma of intrathoracic organ (disorder)	733357007
SNOMED	Ganglioneuroblastoma (disorder)	1.16381E+14
SNOMED	Hairy cell leukemia variant (disorder)	277568007
SNOMED	Primary malignant neoplasm of seminal vesicle (disorder)	369487007
SNOMED	Follicular non-Hodgkins lymphoma of bone (disorder)	448666006
SNOMED	Transitional cell carcinoma of right ureter (disorder)	1.08231E+15
SNOMED	Secondary malignant neoplasm of epididymis (disorder)	94283001
SNOMED	Primary malignant neoplasm of male breast (disorder)	93884005
SNOMED	Reticulosarcoma of intrathoracic lymph nodes (disorder)	95226002
SNOMED	Chondroma	1.09091E+15
SNOMED	Malignant tumor involving left fallopian tube by direct extension from vagina (disorder)	369541004
SNOMED	Malignant phyllodes tumor of breast (disorder)	254844000
SNOMED	Primary endometrioid carcinoma of endometrium of body of uterus (disorder)	762458004
SNOMED	Hodgkins sarcoma of spleen (disorder)	93554008
SNOMED	Adenosquamous carcinoma (disorder)	403902008
SNOMED	High grade B-cell lymphoma (disorder)	277617004
SNOMED	Squamous cell carcinoma of palate (disorder)	276962007
SNOMED	Malignant neoplasm of breast (disorder)	254837009
SNOMED	Liposarcoma (disorder)	254829001

SNOMED	Primary malignant neoplasm of gum (disorder)	371990006
SNOMED	Malignant neoplasm of alveolus of maxilla (disorder)	449223003
SNOMED	Primary malignant neoplasm of splenic flexure of colon (disorder)	94072004
SNOMED	Secondary malignant neoplasm of frontal lobe (disorder)	94309003
SNOMED	Primary carcinoma of ethmoidal sinus (disorder)	707346000
SNOMED	Lymphosarcoma of lymph nodes of head face and neck (disorder)	188500005
SNOMED	Epithelioid leiomyosarcoma of skin (disorder)	404045007
SNOMED	Primary malignant neoplasm of parietal peritoneum (disorder)	93947009
SNOMED	Cutaneous fibrosarcoma (disorder)	254748009
SNOMED	Chloroma (disorder)	188737002
SNOMED	Myelosclerosis with myeloid metaplasia (disorder)	307651005
SNOMED	Primary adenocarcinoma of frontal sinus (disorder)	707343008
SNOMED	Large cell Ewing sarcoma of bone (disorder)	698200003
SNOMED	Drash syndrome	236385009
SNOMED	Secondary malignant neoplasm of junctional zone of tongue (disorder)	94359007
SNOMED	Malignant neoplasm of nose (disorder)	1.09025E+15
SNOMED	Primary transitional cell carcinoma of urethra (disorder)	1.07601E+14
SNOMED	Microcystic adnexal carcinoma of skin (disorder)	254712007
SNOMED	Lymphoma of retroperitoneal space (disorder)	422853008
SNOMED	Digital papillary eccrine carcinoma of skin (disorder)	254709009
SNOMED	Carcinoid crisis (disorder)	237833006
SNOMED	Overlapping malignant neoplasm of gastrointestinal tract (disorder)	109821008
SNOMED	Primary malignant neoplasm of larynx (disorder)	371995001
SNOMED	Secondary malignant neoplasm of aortic body (disorder)	94173006
SNOMED	Malignant neoplasm of overlapping lesion of corpus uteri (disorder)	188198003
SNOMED	Non-small cell lung cancer (disorder)	254637007
SNOMED	Epithelioid hemangioendothelioma of lung (disorder)	254635004
SNOMED	CD-30 negative anaplastic large T-cell cutaneous lymphoma (disorder)	404129007
SNOMED	Malignant polyp of biliary tract (disorder)	255087006
SNOMED	Malignant tumor of retromolar area (disorder)	363391009
SNOMED	Acute promyelocytic leukemia - hypogranular variant (disorder)	285769009
SNOMED	Malignant melanoma of skin of lip (disorder)	93640008
SNOMED	Primary ganglioneuroblastoma of brain (disorder)	735757008
SNOMED	Carcinoid tumor of lung (disorder)	254627002
SNOMED	Follicular lymphoma grade 1 (disorder)	8.47481E+14
SNOMED	Malignant neoplasm of floor of nasopharynx (disorder)	187701005
SNOMED	Carcinoma of pylorus (disorder)	254561006
SNOMED	Mycosis fungoides of lymph nodes of multiple sites (disorder)	188627002
SNOMED	Malignant tumor of biliary tract (disorder)	363415003
SNOMED	Malignant melanoma of popliteal fossa area (disorder)	188071004
SNOMED	Acute biphenotypic leukemia (disorder)	278453007
SNOMED	Secondary malignant neoplasm of gallbladder (disorder)	94312000
SNOMED	Malignant tumor involving right fallopian tube by separate metastasis from left fallopian tube (disorder)	369554006
SNOMED	Malignant neoplasm of alveolus dentalis (disorder)	449260004
SNOMED	[X]Malignant neoplasm of mesothelial and soft tissue (disorder)	4.09231E+14
SNOMED	Carcinoma of duodenum (disorder)	254570009
SNOMED	Kaposi sarcoma not associated with acquired immunodeficiency syndrome (disorder)	703625002
SNOMED	Non-Hodgkins lymphoma of soft tissue (disorder)	448738008
SNOMED	Primary malignant neoplasm of blood vessel of perineum (disorder)	93706003
SNOMED	Primary malignant neoplasm of tonsil (disorder)	372020000
SNOMED	Primary acinar cell carcinoma of trachea (disorder)	707386008
SNOMED	Nodular lymphoma of spleen (disorder)	95193005

SNOMED	Malignant mesothelioma of peritoneum (disorder)	109853004
SNOMED	Primary malignant neoplasm of prepuce (disorder)	372006007
SNOMED	Malignant retroperitoneal tumor (disorder)	363420003
SNOMED	Secondary malignant neoplasm of pancreas (disorder)	94459006
SNOMED	Primary malignant neoplasm of bone of left upper limb (disorder)	3.54641E+14
SNOMED	Malignant tumor involving right ovary by separate metastasis from uterine cervix (disorder)	369571007
SNOMED	Metastasis to adrenal gland of unknown primary (disorder)	285643007
SNOMED	Malignant tumor of parapharyngeal space (disorder)	254530002
SNOMED	Carcinosarcoma of corpus uteri (disorder)	764952009
SNOMED	Malignant lymphoma of lymph nodes of multiple sites (disorder)	93197009
SNOMED	Carcinoma of soft palate (disorder)	254435009
SNOMED	Malignant sex cord tumor of testis (disorder)	700488005
SNOMED	Adrenal carcinoma (disorder)	255035007
SNOMED	Secondary malignant neoplasm of skin of scalp (disorder)	94570001
SNOMED	Leukemic infiltration of skin in chronic myeloid leukemia (disorder)	404153001
SNOMED	Bowel scope (flexible sigmoidoscopy) screen: cancer detected (finding)	8.84601E+14
SNOMED	Malignant neoplasm of connective and soft tissue of foot (disorder)	188004006
SNOMED	Malignant tumor of vallecula (disorder)	363395000
SNOMED	Carcinoma of sublingual gland (disorder)	254466003
SNOMED	Malignant tumor of unknown origin (disorder)	255052006
SNOMED	Primary invasive malignant neoplasm of female breast (disorder)	4.5221E+13
SNOMED	Lymphoepithelioid lymphoma (disorder)	109976000
SNOMED	Carcinoma of retromolar area (disorder)	254457002
SNOMED	Malignant melanoma of skin of lower lip (disorder)	424302003
SNOMED	Myxofibrosarcoma of skin (disorder)	400175006
SNOMED	Primary malignant neoplasm of retina primary (disorder)	93987004
SNOMED	Secondary malignant neoplasm of the mesentery (disorder)	94624001
SNOMED	Malignant neoplasm of thymus heart and mediastinum (disorder)	187881004
SNOMED	Carcinoma of uvula (disorder)	254436005
SNOMED	Intrahepatic bile duct carcinoma (disorder)	109842005
SNOMED	Primary papillary adenocarcinoma of oropharynx (disorder)	707399006
SNOMED	Primary malignant neoplasm of the mesentery (disorder)	372015000
SNOMED	Leukemia: [lymphoid] or [monocytic] or [myeloid] or [specific cell type]	269631008
SNOMED	Malignant tumor of peritoneum and retroperitoneum (disorder)	187801002
SNOMED	Pleomorphic rhabdomyosarcoma (disorder)	404054005
SNOMED	Malignant granulosa cell tumor of ovary (disorder)	254861002
SNOMED	Primary malignant neoplasm of breast upper outer quadrant (disorder)	373088001
SNOMED	Malignant melanoma of skin of chest (disorder)	93218008
SNOMED	Malignant tumor involving vagina by separate metastasis from uterus (disorder)	369586001
SNOMED	Metastatic malignant neoplasm of peripheral nervous system (disorder)	722707001
SNOMED	Carcinoma of frenum of lower lip (disorder)	254402004
SNOMED	Malignant acanthosis nigricans (disorder)	238636003
SNOMED	Carcinoma of frenum of lip (disorder)	254393004
SNOMED	Carcinoma of vermilion border of lower lip (disorder)	254390001
SNOMED	Primary papillary squamous cell carcinoma of larynx (disorder)	707426009
SNOMED	Metastasis from malignant tumor of tongue (disorder)	315009006
SNOMED	Interdigitating dendritic cell sarcoma (disorder)	715664005
SNOMED	Malignant neoplasm of posterior wall of stomach (disorder)	1.09097E+15
SNOMED	Malignant tumor involving rectum by direct extension from vagina (disorder)	369454008
SNOMED	Carcinoma of upper buccal sulcus (disorder)	254441002
SNOMED	Malignant tumor of false cord (disorder)	363488008
SNOMED	Lobular carcinoma of breast (disorder)	278054005

SNOMED	Neuroendocrine carcinoma (disorder)	253000007
SNOMED	Secondary malignant neoplasm of mouth (disorder)	94416001
SNOMED	Primary squamous cell carcinoma of vermilion border of lip (disorder)	2.42951E+14
SNOMED	Secondary malignant neoplasm of retrocecal tissue (disorder)	94517007
SNOMED	Malignant neoplasm of third metacarpal bone (disorder)	187947006
SNOMED	SÃƒ Â©zarys disease of lymph nodes of inguinal region AND/OR lower limb (disorder)	95261008
SNOMED	Leukemic reticuloendotheliosis of lymph nodes of axilla and upper limb (disorder)	188648000
SNOMED	Secondary malignant neoplasm of ciliary body (disorder)	94255003
SNOMED	Overlapping malignant neoplasm of peripheral nerves and autonomic nervous system (disorder)	109919002
SNOMED	Leiomyosarcoma of cervix uteri (disorder)	763771009
SNOMED	Malignant tumor involving vagina by separate metastasis from uterine cervix (disorder)	369585002
SNOMED	Placental site trophoblastic tumor (disorder)	237252008
SNOMED	Primary adenocarcinoma of hypopharynx (disorder)	707395000
SNOMED	B-cell Hodgkins lymphoma (disorder)	1.09189E+15
SNOMED	Malignant tumor of anal canal (disorder)	363352004
SNOMED	Sarcoma of pelvic peritoneum (disorder)	449497006
SNOMED	Secondary malignant neoplasm of skin of trunk (disorder)	94575006
SNOMED	Oxyphilic adenocarcinoma (disorder)	443261008
SNOMED	Cystadenocarcinoma of pancreas (disorder)	235966007
SNOMED	Malignant cystic tumor of exocrine pancreas (disorder)	235965006
SNOMED	Primary squamous cell carcinoma of endometrium (disorder)	733359005
SNOMED	Mixed cell type lymphosarcoma of intra-abdominal lymph nodes (disorder)	94686001
SNOMED	Kaposis sarcoma associated with acquired immunodeficiency syndrome (disorder)	420524008
SNOMED	Hodgkins sarcoma (disorder)	118606001
SNOMED	Malignant melanoma of eyelid (disorder)	231834005
SNOMED	Merkel cell carcinoma of left lower limb (disorder)	3.52071E+14
SNOMED	Primary malignant neoplasm of urinary system (disorder)	94125001
SNOMED	Carcinoma of lingual tonsil (disorder)	254423005
SNOMED	Secondary malignant neoplasm of muscle of pelvis (disorder)	94426008
SNOMED	Malignant pheochromocytoma (disorder)	2.1851E+13
SNOMED	Secondary malignant neoplasm of bronchus of right middle lobe (disorder)	94231008
SNOMED	Malignant melanoma of skin of ankle (disorder)	93211002
SNOMED	Carcinoma of pelvic bones sacrum and coccyx (disorder)	372116002
SNOMED	Macroglobulinemia	190817009
SNOMED	Malignant neoplasm of gum and contiguous sites (disorder)	423691004
SNOMED	Adenocarcinoma of left lung (disorder)	1.59563E+16
SNOMED	Hodgkins disease affecting skin (disorder)	402882001
SNOMED	Subacute myelomonocytic leukemia (disorder)	188770007
SNOMED	Secondary malignant neoplasm of intrahepatic bile ducts (disorder)	94349006
SNOMED	Leukemic reticuloendotheliosis of lymph nodes of inguinal region AND/OR lower limb (disorder)	93149008
SNOMED	Primary fetal adenocarcinoma of lung (disorder)	707403002
SNOMED	Subacute monocytic leukemia (disorder)	188746008
SNOMED	Hodgkins disease lymphocytic-histiocytic predominance of lymph nodes of axilla and upper limb (disorder)	188558005
SNOMED	Monocytic leukemia (disorder)	188744006
SNOMED	Endometrioid carcinoma of prostate (disorder)	278060005
SNOMED	Primary malignant neoplasm of myometrium (disorder)	93915004
SNOMED	Malignant neoplasm of cerebral ventricles (disorder)	363471001
SNOMED	Sarcoma of tibia (disorder)	449206007
SNOMED	Acute myelomonocytic leukemia FAB M4 (disorder)	110005000
SNOMED	Squamous cell carcinoma of back (disorder)	422599000
SNOMED	Primary malignant neoplasm of laryngeal surface of epiglottis (disorder)	93858004
SNOMED	Mucoepidermoid carcinoma of parotid gland (disorder)	423793008

SNOMED	Carcinoma of stomach due to Epstein-Barr virus disease (disorder)	716586009
SNOMED	Malignant melanoma of choroid (disorder)	255021005
SNOMED	Anaplasia of cervix (disorder)	80914001
SNOMED	Malignant tumor of soft tissue of upper limb (disorder)	372011009
SNOMED	Malignant tumor of bladder neck (disorder)	188244007
SNOMED	Primary malignant neoplasm of facial nerve (disorder)	93793002
SNOMED	Malignant mast cell tumor (disorder)	118615008
SNOMED	Non-small cell carcinoma of lung TNM stage 2 (disorder)	425048006
SNOMED	Secondary malignant neoplasm of vermilion border of lower lip (disorder)	94674004
SNOMED	Renal cell carcinoma of kidney except renal pelvis (disorder)	717735006
SNOMED	Follicular lymphoma grade 2 (disorder)	8.47631E+14
SNOMED	Erythroleukemia in remission (disorder)	93450001
SNOMED	Mast cell malignancy of intrapelvic lymph nodes (disorder)	188667001
SNOMED	Primary adenocarcinoma of pyloric antrum of stomach (disorder)	721632008
SNOMED	Malignant tumor involving left fallopian tube by direct extension from right fallopian tube (disorder)	369517002
SNOMED	Primary malignant neoplasm of axillary tail of breast (disorder)	372092003
SNOMED	Malignant neoplasm of intrahepatic gall duct (disorder)	187777008
SNOMED	Malignant meningioma of optic nerve sheath (disorder)	254975005
SNOMED	Malignant tumor of renal pelvis (disorder)	363457009
SNOMED	Secondary malignant neoplasm of thoracic vertebral column (disorder)	94274006
SNOMED	Malignant neoplasm of uncus (disorder)	188290004
SNOMED	Malignant melanoma of skin of lower eyelid (disorder)	423447006
SNOMED	Malignant tumor of foreskin	363450006
SNOMED	Malignant neoplasm of upper lobe, bronchus or lung: (& [Pancoast's syndrome])	187860004
SNOMED	Follicular non-Hodgkins lymphoma of oral cavity (disorder)	449063007
SNOMED	Malignant melanoma of skin of forehead (disorder)	93229007
SNOMED	Non-Hodgkin lymphoma of central nervous system metastatic to lymph node of lower limb (disorder)	1.16811E+14
SNOMED	Hurthle cell carcinoma of thyroid (disorder)	423158009
SNOMED	Malignant tumor of suprahyoid epiglottis (disorder)	254517003
SNOMED	Secondary malignant neoplasm of bronchus of left upper lobe (disorder)	94229004
SNOMED	Secondary lymphangitic carcinoma (disorder)	255118005
SNOMED	Secondary malignant neoplasm of parietal peritoneum (disorder)	94472007
SNOMED	Malignant lymphoma lymphocytic poorly differentiated nodular (disorder)	307646004
SNOMED	Malignant tumor of peripheral nerve (disorder)	254986007
SNOMED	Childhood myelodysplastic syndrome (disorder)	705061009
SNOMED	Nodular lymphoma of lymph nodes of head face and neck (disorder)	188609000
SNOMED	Primary effusion lymphoma (disorder)	713516007
SNOMED	Secondary malignant neoplasm of occipital lobe (disorder)	94448002
SNOMED	Hodgkins disease lymphocytic depletion of intrapelvic lymph nodes (disorder)	188591001
SNOMED	Secondary malignant neoplasm of trapezoid bone (disorder)	94645008
SNOMED	Secondary malignant neoplasm of omentum (disorder)	94626004
SNOMED	Malignant tumor of subglottis (disorder)	363430007
SNOMED	Carcinoma of the rectosigmoid junction (disorder)	269544008
SNOMED	Primary malignant neoplasm of undescended testis (disorder)	94113004
SNOMED	Hodgkins disease mixed cellularity of lymph nodes of multiple sites (disorder)	188582000
SNOMED	Secondary malignant neoplasm of presacral region (disorder)	94502008
SNOMED	Primary malignant neoplasm of muscle of trunk (disorder)	93911008
SNOMED	Primary malignant neoplasm of bone of face (disorder)	93721009
SNOMED	Primary adenocarcinoma of overlapping lesion of urinary organ (disorder)	723879002
SNOMED	Primary mucinous cystic neoplasm with associated invasive carcinoma of biliary tract (disorder)	733352001
SNOMED	Primary malignant neoplasm of jejunum (disorder)	93846004
SNOMED	Squamous cell carcinoma of cervix (disorder)	254886006

SNOMED	Malignant epithelial neoplasm of alveolus dentalis (disorder)	449472007
SNOMED	Malignant melanoma stage IIC (finding)	9.56411E+14
SNOMED	Malignant neoplasm overlapping lesion of digestive system (disorder)	187824009
SNOMED	Hodgkins disease nodular sclerosis of intra-abdominal lymph nodes (disorder)	188567005
SNOMED	Primary malignant neoplasm of soft tissue of right upper extremity (disorder)	3.52301E+14
SNOMED	Hodgkins disease nodular sclerosis of lymph nodes of head face and neck (disorder)	188565002
SNOMED	Hodgkins disease of lymph nodes of head face AND/OR neck (disorder)	93524003
SNOMED	Malignant neoplasm of greater vestibular (Bartholins) gland (disorder)	188211001
SNOMED	Primary malignant neoplasm of main bronchus (disorder)	93882009
SNOMED	Malignant neoplasm of middle lobe of lung (disorder)	187866005
SNOMED	Metastatic renal cell carcinoma (disorder)	702392008
SNOMED	Hodgkins sarcoma of lymph nodes of inguinal region and lower limb (disorder)	188548006
SNOMED	Secondary malignant neoplasm of muscle of face (disorder)	94420002
SNOMED	Hodgkins sarcoma of lymph nodes of head face and neck (disorder)	188544008
SNOMED	Primary malignant neoplasm of gingival mucosa (disorder)	93812000
SNOMED	Carcinoma of anterior two-thirds of tongue - dorsal surface (disorder)	275396004
SNOMED	Overlapping primary malignant neoplasm of bone and articular cartilage of upper limb (disorder)	713038003
SNOMED	Primary adenoid cystic carcinoma of lung (disorder)	707466008
SNOMED	Malignant neoplasm of floor of cerebral ventricle (disorder)	188293002
SNOMED	Malignant tumor of cornea (disorder)	363464006
SNOMED	Malignant tumor involving seminal vesicle by direct extension from prostate (disorder)	369490001
SNOMED	Primary malignant neoplasm of blood vessel of upper arm (disorder)	399884001
SNOMED	Hodgkins disease lymphocytic depletion of lymph nodes of head face and neck (disorder)	188585003
SNOMED	Carcinoma of tongue base - dorsal surface (disorder)	275490009
SNOMED	Burkitts lymphoma of spleen (disorder)	188516007
SNOMED	Primary malignant neoplasm of isthmus of uterus (disorder)	93844001
SNOMED	Primary malignant neoplasm of peripheral nerves of pelvis region (disorder)	109945006
SNOMED	Malignant epithelial neoplasm of pharynx (disorder)	449254004
SNOMED	Malignant neoplasm of long bone of lower leg (disorder)	449628003
SNOMED	Malignant neoplasm of ventral surface of tongue (disorder)	1.09027E+15
SNOMED	Diffuse non-Hodgkins lymphoma of ovary (disorder)	448609001
SNOMED	Metastasis to kidney of unknown primary (disorder)	285639008
SNOMED	Malignant neoplasm of specified parts of peritoneum (disorder)	187808008
SNOMED	Secondary malignant neoplasm of bone (disorder)	94222008
SNOMED	Lymphosarcoma of lymph nodes of axilla and upper limb (disorder)	188503007
SNOMED	Primary squamous cell carcinoma of ethmoidal sinus (disorder)	707359008
SNOMED	Primary mixed mucinous and non-mucinous bronchiolo-alveolar carcinoma of lung (disorder)	707468009
SNOMED	Primary malignant neoplasm of placenta (disorder)	721567004
SNOMED	Primary malignant neoplasm of paraganglion (disorder)	93941005
SNOMED	Primary malignant neoplasm of vestibule of nose (disorder)	94139001
SNOMED	Oligodendroglioma of spinal cord (disorder)	254950006
SNOMED	Primary squamous cell carcinoma of accessory sinus (disorder)	707353009
SNOMED	Malignant tumor involving left ovary by separate metastasis from endometrium (disorder)	369560006
SNOMED	Lymphosarcoma and reticulosarcoma (disorder)	188487008
SNOMED	Primary malignant neoplasm of peripheral nerves and peripheral autonomic nervous system (disorder)	109918005
SNOMED	Secondary malignant neoplasm of ascending colon (disorder)	94179005
SNOMED	Malignant tumor involving right ovary by direct extension from uterine cervix (disorder)	369534005
SNOMED	Malignant tumor of nasal septum (disorder)	363423001
SNOMED	Malignant neoplasm of spinal pia mater (disorder)	188319001
SNOMED	Secondary malignant neoplasm of lymph node from neoplasm of female breast (disorder)	1.62606E+16
SNOMED	Primary malignant neoplasm of respiratory tract (disorder)	93986008
SNOMED	Malignant neoplasm overlapping lesion of bladder (disorder)	188247000

SNOMED	Primary malignant neoplasm of soft tissues of buttock (disorder)	94052009
SNOMED	Malignant neoplasm of thorax (disorder)	188361007
SNOMED	Primary malignant neoplasm of extrahepatic bile duct (disorder)	446189008
SNOMED	Mast cell malignancy of spleen (disorder)	188668006
SNOMED	Monoclonal gammopathy of undetermined significance	35601003
SNOMED	Follicular non-Hodgkins lymphoma (disorder)	308121000
SNOMED	Mixed eccrine/pilar adnexal carcinoma of skin (disorder)	403954003
SNOMED	Primary mucinous cystic neoplasm with associated invasive carcinoma of perihilar bile duct (disorder)	733346002
SNOMED	Malignant neoplasm of main bronchus (disorder)	372065009
SNOMED	Malignant tumor of endocrine pancreas (disorder)	254611009
SNOMED	Malignant neoplasm of peripheral nerves of head face and neck (disorder)	188322004
SNOMED	Malignant infiltration of peripheral nerve (disorder)	255128001
SNOMED	Embryonal carcinoma of ovary (disorder)	254872007
SNOMED	Leukemic reticuloendotheliosis of intrapelvic lymph nodes (disorder)	93145002
SNOMED	Endometrial stromal sarcoma (disorder)	699356008
SNOMED	Secondary malignant neoplasm of pectoral axillary lymph nodes (disorder)	94477001
SNOMED	Primary osteosarcoma of bone of jaw (disorder)	723848001
SNOMED	Diffuse non-Hodgkins lymphoma of testis (disorder)	448465000
SNOMED	Hodgkins disease lymphocytic depletion reticular type (disorder)	307634003
SNOMED	Malignant tumor involving rectum by separate metastasis from uterus (disorder)	369460008
SNOMED	Secondary malignant neoplasm of blood vessel of inguinal region (disorder)	94198007
SNOMED	Secondary malignant neoplasm of vagina (disorder)	94668004
SNOMED	Primary malignant neoplasm of breast lower outer quadrant (disorder)	373091001
SNOMED	Philadelphia chromosome negative chronic myelogenous leukemia (disorder)	449386007
SNOMED	Primary malignant neoplasm of mandible (disorder)	93886007
SNOMED	Malignant neoplasm of cerebral peduncle (disorder)	188295009
SNOMED	Malignant tumor of spinal cord intramedullary (disorder)	424334007
SNOMED	Solitary osseous myeloma (disorder)	426336007
SNOMED	Metastasis to spleen of unknown primary (disorder)	285615003
SNOMED	Overlapping malignant neoplasm of esophagus (disorder)	109835005
SNOMED	Malignant neoplasm of lumbar vertebra (disorder)	187918004
SNOMED	Primary malignant neoplasm of anterior two-thirds of tongue (disorder)	371968006
SNOMED	Primary malignant neoplasm of great vessels (disorder)	93817006
SNOMED	Secondary malignant neoplasm of skin of umbilicus (disorder)	94576007
SNOMED	Metastasis from malignant tumor of cervix (disorder)	314992001
SNOMED	Primary malignant neoplasm of right lower lobe of lung (disorder)	93991009
SNOMED	Malignant tumor of myocardium (disorder)	363437005
SNOMED	Secondary malignant neoplasm of vas deferens (disorder)	94671007
SNOMED	Malignant neoplasm of mesentery (disorder)	363370001
SNOMED	Malignant tumor involving uterine corpus by direct extension from bladder (disorder)	369493004
SNOMED	Primary seminoma of left testis (disorder)	1.08178E+15
SNOMED	Primary serous adenocarcinoma of endometrium (disorder)	722680009
SNOMED	Primary malignant neoplasm of ampulla of Vater (disorder)	371967001
SNOMED	Malignant melanoma of perineum (disorder)	188054000
SNOMED	Carcinomatous metastasis in skin (disorder)	404092006
SNOMED	Primary malignant clear cell neoplasm of endometrium (disorder)	1.07771E+14
SNOMED	Malignant neoplasm of eyeball excluding conjunctiva cornea retina and choroid (disorder)	188261005
SNOMED	Malignant neoplasm of overlapping lesion of urinary organs (disorder)	188256008
SNOMED	Secondary malignant neoplasm of prepuce (disorder)	94307001
SNOMED	Malignant neoplasm of first metacarpal bone (disorder)	187945003
SNOMED	Malignant histiocytosis of lymph nodes of head face and neck (disorder)	188640007
SNOMED	Clear cell papillary renal cell carcinoma (disorder)	734015000

SNOMED	Papillary thyroid carcinoma with renal papillary neoplasia (disorder)	717734005
SNOMED	Carcinoma of fallopian tube (disorder)	276870001
SNOMED	Primary malignant neoplasm of fibula (disorder)	93799003
SNOMED	Secondary malignant neoplasm of skin of head (disorder)	188454009
SNOMED	Malignant tumor of thoracic part of esophagus (disorder)	187723009
SNOMED	Secondary malignant neoplasm of spinal meninges (disorder)	94601008
SNOMED	Secondary malignant neoplasm of lumbosacral plexus (disorder)	709285002
SNOMED	Malignant tumor of lacrimal drainage structure (disorder)	280959007
SNOMED	Malignant melanoma of skin of external auditory canal (disorder)	93222003
SNOMED	Malignant glioma of cerebrum (disorder)	8.7091E+13
SNOMED	Malignant tumor of middle third of esophagus (disorder)	187726001
SNOMED	Malignant neoplasm of hippocampus (disorder)	188289008
SNOMED	Primitive neuroectodermal tumor of corpus uteri (disorder)	766247009
SNOMED	Perforated carcinoma of esophagus (disorder)	307216009
SNOMED	Secondary malignant neoplasm of oculomotor nerve (disorder)	94450005
SNOMED	Secondary malignant neoplasm of muscle of perineum (disorder)	94427004
SNOMED	Primary vulval cancer (disorder)	275419009
SNOMED	Primary squamous cell carcinoma of nasal cavity (disorder)	723182009
SNOMED	Malignant glioma of cerebellum (disorder)	8.7121E+13
SNOMED	Klatskins tumor (disorder)	253017000
SNOMED	Primary malignant neoplasm of blood vessel of neck (disorder)	93704000
SNOMED	Sarcoma of skull (disorder)	449295001
SNOMED	Malignant neoplasm of fundus of corpus uteri (disorder)	188191009
SNOMED	Malignant tumor involving vulva by direct extension from vagina (disorder)	369587005
SNOMED	Carcinosarcoma of skin (disorder)	404087009
SNOMED	Secondary malignant neoplasm of abdominal esophagus (disorder)	94152006
SNOMED	Malignant neoplasm of thoracic vertebra (disorder)	187917009
SNOMED	Primary malignant neoplasm of thoracic vertebral column (disorder)	372019006
SNOMED	Burkitts lymphoma of intrathoracic lymph nodes (disorder)	188511002
SNOMED	Primary malignant neoplasm of abdominal esophagus (disorder)	371962007
SNOMED	Pituitary carcinoma (disorder)	254955001
SNOMED	Hodgkins disease nodular sclerosis of lymph nodes of axilla and upper limb (disorder)	188568000
SNOMED	Transitional cell carcinoma of bladder (disorder)	255109008
SNOMED	Primary malignant neoplasm of parietal bone (disorder)	93945001
SNOMED	Malignant neoplasm of nipple and areola of male breast (disorder)	188163001
SNOMED	Secondary malignant neoplasm of circumflex iliac lymph nodes (disorder)	1.09021E+15
SNOMED	Malignant neoplasm of extraocular muscle of orbit (disorder)	188269007
SNOMED	Malignant tumor of maxillary sinus (disorder)	363425008
SNOMED	Malignant neoplasm of lower-outer quadrant of female breast (disorder)	188155002
SNOMED	Malignant tumor involving an organ by direct extension from ovary (disorder)	369597001
SNOMED	Malignant blue nevus of skin (disorder)	307603002
SNOMED	Pleomorphic malignant fibrous histiocytoma of skin (disorder)	398903003
SNOMED	Malignant neoplasm of central part of female breast (disorder)	188151006
SNOMED	Secondary malignant neoplasm of upper gum (disorder)	94652005
SNOMED	Malignant tumor involving seminal vesicle by separate metastasis from bladder (disorder)	369491002
SNOMED	Malignant neoplasm of round ligament (disorder)	188204000
SNOMED	Mucinous eccrine carcinoma of skin (disorder)	254714008
SNOMED	Malignant melanoma of foot (disorder)	188075008
SNOMED	Malignant melanoma of heel (disorder)	188074007
SNOMED	Leiomyosarcoma of connective tissue (disorder)	447804006
SNOMED	Malignant melanoma of lower leg (disorder)	188072006
SNOMED	Primary malignant neoplasm of blood vessel of trunk (disorder)	93712008

SNOMED	Malignant melanoma of knee (disorder)	188070003
SNOMED	Acute lymphoid leukemia disease (disorder)	91857003
SNOMED	Enteropathy-associated T-cell lymphoma (disorder)	277654008
SNOMED	Malignant neoplasm of broad ligament of uterus (disorder)	449259009
SNOMED	Primary malignant neoplasm of bronchus of right middle lobe (disorder)	93732009
SNOMED	Metastasis from malignant tumor of lung (disorder)	315006004
SNOMED	Malignant melanoma of hand (disorder)	188063003
SNOMED	Malignant melanoma of forearm (disorder)	188062008
SNOMED	Leiomyosarcoma of uterus (disorder)	447389009
SNOMED	Malignant melanoma of shoulder (disorder)	188060000
SNOMED	Primary malignant neoplasm of vermilion border of lower lip (disorder)	372026006
SNOMED	Malignant neoplasm of arytenoid cartilage (disorder)	187843009
SNOMED	Malignant neoplasm of lower lip external (disorder)	187604008
SNOMED	Primary adenosquamous carcinoma of hypopharynx (disorder)	707485006
SNOMED	Malignant tumor of pyriform fossa (disorder)	363401000
SNOMED	Malignant melanoma of axilla (disorder)	188049009
SNOMED	Malignant mast cell tumor of spleen (disorder)	93207008
SNOMED	Primary basaloid squamous cell carcinoma of lung (disorder)	707454002
SNOMED	Lymphoma of small intestine (disorder)	449074003
SNOMED	Malignant tumor of bronchus (disorder)	363493006
SNOMED	Malignant melanoma of ear and/or external auditory canal (disorder)	188032002
SNOMED	Primary malignant neoplasm of adrenal cortex (disorder)	371963002
SNOMED	Malignant tumor of soft tissue of head (disorder)	363439008
SNOMED	Malignant epithelial neoplasm of vulva (disorder)	447882007
SNOMED	Malignant neoplasm of anterior wall of stomach (disorder)	1.09098E+15
SNOMED	Malignant neoplasm of urinary organ (disorder)	448233000
SNOMED	T-zone lymphoma (disorder)	109975001
SNOMED	Sarcoma of vulva (disorder)	254897006
SNOMED	Carcinoma of lower gum (disorder)	254425003
SNOMED	Malignant neoplasm of upper lobe of left lung (disorder)	724058006
SNOMED	Overlapping squamous cell carcinoma of larynx (disorder)	707429002
SNOMED	Adenocarcinoma of penis (disorder)	764846009
SNOMED	Malignant neoplasms of independent (primary) multiple sites (disorder)	188478004
SNOMED	Malignant neoplasm of tarsus of eyelid (disorder)	187988006
SNOMED	Chronic myelomonocytic leukemia (disorder)	127225006
SNOMED	Chronic myeloid leukemia in myeloid blast crisis (disorder)	413843002
SNOMED	Malignant germ cell tumor of ovary (disorder)	254869000
SNOMED	Malignant tumor of glottis (disorder)	187841006
SNOMED	Malignant neoplasm of connective and soft tissue of thorax (disorder)	188009001
SNOMED	Primary malignant neoplasm of female genital organ (disorder)	93797001
SNOMED	Overlapping malignant neoplasm of brain (disorder)	109912006
SNOMED	Primary malignant neoplasm of right kidney (disorder)	3.54361E+14
SNOMED	Malignant neoplasm of connective and soft tissue of lower leg (disorder)	188003000
SNOMED	Primary malignant neoplasm of unspecified site (disorder)	109356001
SNOMED	Malignant neoplasm of connective and soft tissue of thigh and upper leg (disorder)	188001003
SNOMED	Primary malignant neoplasm of vas deferens (disorder)	94133000
SNOMED	Malignant epithelial neoplasm of appendix (disorder)	448992002
SNOMED	Myeloid sarcoma in remission (disorder)	94718004
SNOMED	Primary malignant melanoma of vagina (disorder)	721563000
SNOMED	Seminoma (disorder)	443675005
SNOMED	Primary malignant neoplasm of chest wall (disorder)	93754006
SNOMED	Large cell carcinoma of lung (disorder)	254629004

SNOMED	Malignant neoplasm of connective and soft tissue of shoulder (disorder)	187992004
SNOMED	Primary malignant neoplasm of retroperitoneum (disorder)	94092006
SNOMED	Primary malignant neoplasm of pelvic peritoneum (disorder)	93952004
SNOMED	Malignant tumor of middle ear and mastoid (disorder)	269463006
SNOMED	Malignant epithelial neoplasm of lung (disorder)	448993007
SNOMED	Secondary malignant neoplasm of posterior wall of nasopharynx (disorder)	94498001
SNOMED	Primary adenocarcinoma of descending colon and splenic flexure (disorder)	721699002
SNOMED	Primary papillary squamous cell carcinoma of lung (disorder)	707455001
SNOMED	Malignant tumor of supraglottis (disorder)	187842004
SNOMED	Malignant neoplasm of connective and soft tissue of popliteal space (disorder)	188002005
SNOMED	Malignant neoplasm of first metatarsal bone (disorder)	187973001
SNOMED	Primary adenocarcinoma of vulva (disorder)	9.1131E+13
SNOMED	Malignant neoplasm of lateral cuneiform (disorder)	187970003
SNOMED	Primary malignant neoplasm of sacrococcygeal region (disorder)	93995000
SNOMED	Mast cell malignancy of intrathoracic lymph nodes (disorder)	188663002
SNOMED	Secondary malignant neoplasm of blood vessel of shoulder (disorder)	94205006
SNOMED	Malignant neoplasm of coccygeal vertebra (disorder)	187957007
SNOMED	Malignant neoplasm of sacral vertebra (disorder)	187956003
SNOMED	Malignant neoplasm of pelvic bones sacrum and coccyx (disorder)	187952001
SNOMED	Malignant neoplasm of carpal bone - pisiform (disorder)	187940008
SNOMED	Secondary malignant neoplasm of seminal vesicle (disorder)	369488002
SNOMED	Primary oxyphilic adenocarcinoma of oropharynx (disorder)	707396004
SNOMED	Secondary malignant neoplasm of temporal bone (disorder)	94621009
SNOMED	Rhabdomyosarcoma (disorder)	302847003
SNOMED	Secondary malignant neoplasm of cerebral ventricle (disorder)	94247005
SNOMED	Systemic mastocytosis with associated clonal hematological non-mast cell lineage disease (disorder)	397015000
SNOMED	Malignant neoplasm of lower lobe of lung (disorder)	187870002
SNOMED	Paraspinal neuroblastoma (disorder)	281565009
SNOMED	Squamous cell carcinoma of lung TNM stage 3 (disorder)	425230006
SNOMED	Carcinoma of frenum linguae (disorder)	254417000
SNOMED	Metastasis to lymph node of unknown primary (disorder)	285644001
SNOMED	Secondary malignant neoplasm of soft tissues of trunk (disorder)	94595000
SNOMED	Malignant tumor of inferior surface of soft palate (disorder)	254503007
SNOMED	Malignant melanoma of skin of hand (disorder)	93636004
SNOMED	Secondary malignant neoplasm of posterior wall of urinary bladder (disorder)	94500000
SNOMED	Malignant neoplasm of scapula and long bones of upper arm (disorder)	187929000
SNOMED	Primary papillary squamous cell carcinoma of trachea (disorder)	707391009
SNOMED	Malignant neoplasm of costovertebral joint (disorder)	187926007
SNOMED	Transitional cell carcinoma of left ureter (disorder)	1.08229E+15
SNOMED	Malignant neoplasm of epiglottis free border (disorder)	187682009
SNOMED	Hodgkins disease lymphocytic-histiocytic predominance of lymph nodes of inguinal region and lower limb (disorder)	188559002
SNOMED	Malignant neoplasm of supraclavicular fossa (disorder)	1.09095E+15
SNOMED	Acute leukemia disease (disorder)	91855006
SNOMED	Primary chondrosarcoma of bone (disorder)	735679005
SNOMED	Malignant tumor of digestive organ (disorder)	255077007
SNOMED	Malignant neoplasm of bones of skull and face (disorder)	187900002
SNOMED	Secondary malignant neoplasm of parametrial lymph nodes (disorder)	94464005
SNOMED	Secondary malignant neoplasm of skin of temporal region (disorder)	94572009
SNOMED	Secondary malignant neoplasm of occipital lymph nodes (disorder)	94449005
SNOMED	Malignant tumor involving uterine cervix by separate metastasis from vagina (disorder)	369574004
SNOMED	Secondary malignant neoplasm of pharynx (disorder)	94488007
SNOMED	Primary cutaneous marginal zone B-cell lymphoma (disorder)	404140004

SNOMED	Malignant neoplasm of omentum (disorder)	363421004
SNOMED	Hodgkins disease lymphocytic depletion of lymph nodes of axilla AND/OR upper limb (disorder)	93487009
SNOMED	Malignant tumor of uvula (disorder)	363389001
SNOMED	Malignant neoplasm of lower lobe of right lung (disorder)	724056005
SNOMED	Overlapping malignant neoplasm of ill-defined site (disorder)	109358000
SNOMED	Indeterminate dendritic cell neoplasm (disorder)	721313009
SNOMED	Malignant tumor of greater curve of stomach (disorder)	269460009
SNOMED	Malignant epithelial neoplasm of ureter (disorder)	448864006
SNOMED	Secondary malignant neoplasm of left ovary (disorder)	369523007
SNOMED	Lymphoma of cardioesophageal junction (disorder)	449075002
SNOMED	Primary malignant neoplasm of tongue (disorder)	94101009
SNOMED	Carcinoma of upper limb bones/scapula (disorder)	286889008
SNOMED	Malignant melanoma of lip (disorder)	188030005
SNOMED	Osteosclerotic myeloma (disorder)	425657001
SNOMED	Malignant tumor of frenum of upper lip (disorder)	187608006
SNOMED	Malignant neoplasm of basal ganglia (disorder)	188281006
SNOMED	Lymphoepithelial carcinoma (disorder)	764938007
SNOMED	Gastric lymphoma (disorder)	276811008
SNOMED	Squamous cell carcinoma of lung TNM stage 2 (disorder)	423468007
SNOMED	Leiomyosarcoma (disorder)	443719001
SNOMED	Large cell lymphoma of intrapelvic lymph nodes (disorder)	441962003
SNOMED	Malignant neoplasm of xiphoid process (disorder)	187927003
SNOMED	Primary myosarcoma of uterus (disorder)	723077004
SNOMED	Adenocarcinoma of cervix (disorder)	254887002
SNOMED	Malignant tumor of cecum (disorder)	363350007
SNOMED	Lymphomatoid papulosis type A (CD-30 positive type) (disorder)	404103007
SNOMED	Tonsil carcinoma (disorder)	274085008
SNOMED	Nasopharyngeal carcinoma (disorder)	449248000
SNOMED	Secondary malignant neoplasm of middle ear (disorder)	94413009
SNOMED	Malignant neoplasm of mesocolon (disorder)	187809000
SNOMED	Lymphosarcoma of spleen (disorder)	188506004
SNOMED	Overlapping malignant lesion of retroperitoneum and peritoneum (disorder)	187807003
SNOMED	Malignant neoplasm of retrocecal tissue (disorder)	187804005
SNOMED	Malignant neoplasm of perinephric tissue (disorder)	187803004
SNOMED	Secondary malignant neoplasm of endocervix (disorder)	94279001
SNOMED	Primary malignant neoplasm of metatarsal bone (disorder)	93893006
SNOMED	Overlapping malignant neoplasm of hypopharynx (disorder)	109368005
SNOMED	Malignant tumor of pancreatic duct (disorder)	187793004
SNOMED	Hodgkins disease mixed cellularity of intrapelvic lymph nodes (disorder)	188580008
SNOMED	Secondary malignant neoplasm of lateral wall of urinary bladder (disorder)	94374009
SNOMED	Non-Hodgkins lymphoma of uterine cervix (disorder)	448774004
SNOMED	Ewing sarcoma of bone of pelvis (disorder)	7.8411E+13
SNOMED	Secondary malignant neoplasm of short bone of upper limb (disorder)	94536002
SNOMED	Malignant mesothelioma of omentum (disorder)	109858008
SNOMED	Secondary malignant neoplasm of sublingual gland (disorder)	94608002
SNOMED	Primary squamous cell carcinoma of overlapping lesion of urinary organs (disorder)	724467001
SNOMED	Supratentorial primitive neuroectodermal tumor (disorder)	699318007
SNOMED	Leukemic infiltration of skin in acute myeloid leukemia (disorder)	404152006
SNOMED	Malignant neoplasm of rectum (& carcinoma)	187761007
SNOMED	Primary squamous cell carcinoma of posterior wall of oropharynx (disorder)	707532001
SNOMED	Malignant neoplasm overlapping lesion of colon (disorder)	187757001
SNOMED	Malignant tumor of Meckels diverticulum (disorder)	187752007

SNOMED	Burkitts tumor of lymph nodes of multiple sites (disorder)	92514004
SNOMED	Malignant tumor of fundus of stomach (disorder)	187741001
SNOMED	Malignant neoplasm of upper lobe bronchus (disorder)	187861000
SNOMED	Lymphoma with spill (disorder)	277570003
SNOMED	Local recurrence of malignant tumor of thyroid gland (disorder)	314953008
SNOMED	Malignant neoplasm of cardioesophageal junction of stomach (disorder)	187734007
SNOMED	Metaplastic carcinoma of breast (disorder)	763479005
SNOMED	Malignant neoplasm of carpal bone - hamate (disorder)	187944004
SNOMED	Malignant tumor of ileum (disorder)	363405009
SNOMED	Malignant tumor of inferior turbinate (disorder)	254478004
SNOMED	Endometrial carcinoma (disorder)	254878006
SNOMED	Secondary malignant neoplasm of pelvis (disorder)	94480000
SNOMED	Malignant neoplasm of orbital bone (disorder)	187906008
SNOMED	Malignant neoplasm of cuneiform cartilage (disorder)	187845002
SNOMED	Primary malignant melanoma of anal canal (disorder)	722543005
SNOMED	Malignant melanoma of skin of temporal region (disorder)	93648001
SNOMED	Malignant tumor involving bladder by direct extension from prostate (disorder)	369472002
SNOMED	Malignant neoplasm of fourth metacarpal bone (disorder)	187948001
SNOMED	Medulloepithelioma (disorder)	715903004
SNOMED	Malignant neoplasm of connective tissue (disorder)	448274000
SNOMED	Reticulosarcoma of intrapelvic lymph nodes (disorder)	95225003
SNOMED	Secondary malignant neoplasm of hepatic flexure of colon (disorder)	94328005
SNOMED	Krukenberg tumor (disorder)	359987004
SNOMED	Hodgkins disease mixed cellularity of intra-abdominal lymph nodes (disorder)	188577007
SNOMED	Malignant tumor of nasopharynx (disorder)	187692001
SNOMED	Malignant tumor of posterior wall of oropharynx (disorder)	187688008
SNOMED	Malignant melanoma of skin of nose (disorder)	93643005
SNOMED	Malignant neoplasm of glossoepiglottic fold (disorder)	187683004
SNOMED	Local recurrence of malignant tumor of liver (disorder)	314963000
SNOMED	Malignant lymphoma of intrapelvic lymph nodes (disorder)	93192003
SNOMED	Lethal midline granuloma (disorder)	58961005
SNOMED	Malignant tumor involving vulva by separate metastasis from fallopian tube (disorder)	369589008
SNOMED	Hereditary nonpolyposis colon cancer (disorder)	315058005
SNOMED	Malignant tumor involving right fallopian tube by direct extension from left fallopian tube (disorder)	369548005
SNOMED	Primary carcinoma ex pleomorphic adenoma of oropharynx (disorder)	707587000
SNOMED	Liposarcoma of orbit (disorder)	254993006
SNOMED	Malignant tumor of vestibule of mouth (disorder)	187658004
SNOMED	Primary basaloid carcinoma of oropharynx (disorder)	707580003
SNOMED	Hodgkins disease lymphocytic-histiocytic predominance of intrathoracic lymph nodes (disorder)	93495008
SNOMED	Hodgkins disease lymphocytic depletion of lymph nodes of inguinal region and lower limb (disorder)	188590000
SNOMED	Malignant tumor of frenum linguae (disorder)	187641009
SNOMED	Eccrine carcinoma of skin (disorder)	400173004
SNOMED	Histiocytic medullary reticulosis (disorder)	307650006
SNOMED	Carcinoma of cervix (disorder)	285432005
SNOMED	Malignant tumor of anterior two-thirds of tongue - dorsal surface (disorder)	187634003
SNOMED	Malignant neoplasm of dorsal surface of tongue (disorder)	187633009
SNOMED	Malignant neoplasm of base of tongue dorsal surface (disorder)	187631006
SNOMED	Malignant neoplasm of overlapping lesion of lip (disorder)	187624007
SNOMED	Primary malignant neoplasm of cauda equina (disorder)	93743001
SNOMED	Secondary malignant neoplasm of lunate bone (disorder)	94390009
SNOMED	Overlapping malignant neoplasm of tongue (disorder)	109823006
SNOMED	Metastasis from malignant tumor of prostate (disorder)	314994000

SNOMED	Adenoid cystic carcinoma of parotid gland (disorder)	423615009
SNOMED	Primary malignant neoplasm of ureteric orifice of urinary bladder (disorder)	94122003
SNOMED	Primary malignant neoplasm of sacrum (disorder)	93996004
SNOMED	Malignant neoplasm: [lip] or [oral cavity] or [pharynx]	187598005
SNOMED	Monoclonal gammopathy (clinical)	109983007
SNOMED	Primary malignant neoplasm of base of tongue (disorder)	93687001
SNOMED	Malignant neoplasm of tongue tip and lateral border (disorder)	187637005
SNOMED	Retroperitoneal sarcoma (disorder)	307219002
SNOMED	Burkitts tumor of intrapelvic lymph nodes (disorder)	92509003
SNOMED	Primary malignant neuroendocrine neoplasm of stomach (disorder)	721634009
SNOMED	Primary small cell malignant neoplasm of lung TNM stage 1 (disorder)	6.7811E+13
SNOMED	Primary malignant neoplasm of ileum (disorder)	93832004
SNOMED	Malignant neoplasm of peripheral nerves of lower limb including hip (disorder)	188324003
SNOMED	Metastasis to lymph node from squamous cell carcinoma (disorder)	1.671E+12
SNOMED	Metastasis to lung from adenocarcinoma (disorder)	1.661E+12
SNOMED	Carcinoid syndrome (disorder)	35868009
SNOMED	Secondary malignant neoplasm of nipple of male breast (disorder)	94444000
SNOMED	Malignant tumor involving uterine cervix by direct extension from fallopian tube (disorder)	369497003
SNOMED	Hodgkins disease mixed cellularity of lymph nodes of axilla AND/OR upper limb (disorder)	93505001
SNOMED	Malignant tumor involving bladder by direct extension from endometrium (disorder)	369469009
SNOMED	High grade endometrial stromal sarcoma (disorder)	699358009
SNOMED	Primary malignant neoplasm of bilateral female breasts (disorder)	1.56358E+16
SNOMED	Squamous cell carcinoma of colon (disorder)	766981007
SNOMED	Secondary malignant neoplasm of lymph nodes of multiple sites (disorder)	94396003
SNOMED	Malignant tumor of pineal gland (disorder)	363483004
SNOMED	Primary malignant germ cell neoplasm (disorder)	1.45831E+14
SNOMED	Primary salivary gland type carcinoma of trachea (disorder)	707380002
SNOMED	Mucosa-associated lymphoid tissue lymphoma of orbit (disorder)	414780005
SNOMED	Merkel cell carcinoma of lower limb (disorder)	1.33881E+14
SNOMED	Carcinoma of lower labial sulcus (disorder)	254454009
SNOMED	Secondary malignant neoplasm of diaphragmatic lymph nodes (disorder)	1.09016E+15
SNOMED	Secondary malignant neoplasm of base of tongue (disorder)	94184004
SNOMED	Malignant tumor of nasal skeleton (disorder)	254474002
SNOMED	Secondary malignant neoplasm of septum of nose (disorder)	94534004
SNOMED	Acute myeloid leukemia with t(9:11)(p22;q23); MLLT3-MLL (disorder)	444911000
SNOMED	Primary malignant neoplasm of patella (disorder)	93950007
SNOMED	Primary malignant neoplasm of adrenal medulla (disorder)	371966005
SNOMED	Malignant melanoma of breast (disorder)	188050009
SNOMED	Secondary malignant neoplasm of rectum (disorder)	94513006
SNOMED	Squamous cell carcinoma of tongue (disorder)	276952000
SNOMED	Primary synovial sarcoma of intrathoracic organ (disorder)	722828003
SNOMED	Malignant tumor of vulva (disorder)	363367000
SNOMED	SÃƒ Â©zarys disease (disorder)	118611004
SNOMED	Primary malignant neoplasm of middle third of esophagus (disorder)	371999007
SNOMED	Malignant neoplasm of tapetum (disorder)	188302003
SNOMED	Malignant carcinoid tumor of stomach (disorder)	709830006
SNOMED	Neoplasm of stomach	126824007
SNOMED	Neoplasm of respiratory tract	126667002
SNOMED	Extranodal marginal zone lymphoma of mucosa-associated lymphoid tissue of stomach (disorder)	444597005
SNOMED	Endocervical adenocarcinoma (disorder)	123842006
SNOMED	Malignant poorly differentiated neuroendocrine carcinoma (disorder)	1.23631E+14
SNOMED	Alpha heavy chain disease enteric form (disorder)	123313007

SNOMED	Primary adenocarcinoma of sphenoidal sinus (disorder)	707344002
SNOMED	Megakaryoblastic acute myeloid leukemia with t(1;22)(p13;q13) (disorder)	763796007
SNOMED	Kaposis sarcoma - sporadic (disorder)	403978008
SNOMED	Malignant tumor of upper labial mucosa (disorder)	187606005
SNOMED	Malignant neoplasm of peripheral nerves of trunk (disorder)	1.0909E+15
SNOMED	Hyperviscosity syndrome	11888009
SNOMED	Secondary malignant neoplasm of hypopharynx (disorder)	94334003
SNOMED	Hodgkins granuloma of lymph nodes of multiple sites (disorder)	188541000
SNOMED	Dermatofibrosarcoma protuberans with giant cell fibroblastoma (disorder)	404010004
SNOMED	Langerhans cell histiocytosis disseminated (disorder)	118614007
SNOMED	Malignant epithelial neoplasm of spinal cord (disorder)	448314007
SNOMED	Malignant tumor of ureteric orifice (disorder)	188245008
SNOMED	[Malignant neoplasm] or [carcinoma] of bone (& [sarcoma]), connective tissue, skin and breast	187898004
SNOMED	Malignant tumor of upper labial sulcus (disorder)	187661003
SNOMED	Malignant neoplasm of bone of lower limb (disorder)	372108006
SNOMED	Secondary malignant neoplasm of body of pancreas (disorder)	94212002
SNOMED	Malignant neoplasm of corpus striatum (disorder)	188283009
SNOMED	Primary malignant neoplasm of nasal concha (disorder)	93918002
SNOMED	Malignant tumor involving vagina by direct extension from uterus (disorder)	369503006
SNOMED	Hodgkins disease of lymph nodes of inguinal region AND/OR lower limb (disorder)	93525002
SNOMED	Malignant neoplasm of posterior wall of urinary bladder (disorder)	188243001
SNOMED	Squamous cell carcinoma of head and neck (disorder)	716659002
SNOMED	Hodgkins disease (disorder)	118599009
SNOMED	Non-Hodgkin lymphoma of central nervous system metastatic to lymph node of upper limb (disorder)	1.16821E+14
SNOMED	Malignant neoplasm of upper-outer quadrant of female breast (disorder)	188154003
SNOMED	Primary malignant neoplasm of soft tissue of right lower extremity (disorder)	3.52311E+14
SNOMED	Squamous cell carcinoma of anogenital area (disorder)	402816008
SNOMED	Secondary malignant neoplasm of myometrium (disorder)	94434002
SNOMED	Primary small cell carcinoma of endometrium (disorder)	722682001
SNOMED	Primary myoepithelial carcinoma of trachea (disorder)	707378008
SNOMED	Prolymphocytic leukemia (disorder)	110006004
SNOMED	Primary malignant neoplasm of visceral pleura (disorder)	94140004
SNOMED	Overlapping malignant neoplasm of bone and articular cartilage (disorder)	109347009
SNOMED	Primary malignant neoplasm of navicular bone of foot (disorder)	93920004
SNOMED	Angiosarcoma of cheek (disorder)	447757002
SNOMED	Refractory anemia with ringed sideroblasts (disorder)	109998009
SNOMED	Primary adenocarcinoma of pelvis (disorder)	1.84871E+14
SNOMED	Myelodysplastic syndrome (disorder)	109995007
SNOMED	Malignant tumor involving left fallopian tube by separate metastasis from uterine cervix (disorder)	369610009
SNOMED	Polycythemia vera (disorder)	109992005
SNOMED	Gorlin syndrome (disorder)	69408002
SNOMED	Multiple myeloma (disorder)	109989006
SNOMED	Primary malignant neoplasm of adenoid (disorder)	93662008
SNOMED	Secondary malignant neoplasm of junctional region of epiglottis (disorder)	94358004
SNOMED	Secondary malignant neoplasm of vestibule of nose (disorder)	94677006
SNOMED	Hereditary breast and ovarian cancer syndrome (disorder)	718220008
SNOMED	Acral lentiginous malignant melanoma of skin (disorder)	254732008
SNOMED	Secondary malignant neoplasm of clavicle (disorder)	94256002
SNOMED	Merkel cell carcinoma of right lower limb (disorder)	3.51461E+14
SNOMED	Malignant melanoma of iris (disorder)	255012009
SNOMED	Primary malignant neoplasm of hypopharyngeal aspect of interarytenoid fold (disorder)	93830007
SNOMED	Malignant tumor of vault of bladder (disorder)	188240003

SNOMED	Primary malignant neoplasm of rib (disorder)	93990005
SNOMED	Follicular non-Hodgkins lymphoma large cell (disorder)	109972003
SNOMED	Secondary malignant neoplasm of bladder (disorder)	94186002
SNOMED	Burkitts tumor of lymph nodes of head face AND/OR neck (disorder)	92512000
SNOMED	Secondary malignant neoplasm of blood vessel of upper limb (disorder)	94210005
SNOMED	Primary malignant neoplasm of posterior mediastinum (disorder)	93969002
SNOMED	Diffuse non-Hodgkins lymphoma small cleaved cell (disorder)	109967007
SNOMED	Diffuse non-Hodgkins lymphoma immunoblastic (disorder)	109966003
SNOMED	Leukemic infiltration of skin in monocytic leukemia (disorder)	404154007
SNOMED	Metastasis to bladder of unknown primary (disorder)	285640005
SNOMED	Primary adenocarcinoma of nasal cavity (disorder)	721560002
SNOMED	Malignant neoplasm of parietal pleura (disorder)	449067008
SNOMED	Carcinoma of lower buccal sulcus (disorder)	254445006
SNOMED	Primary malignant neoplasm of biliary tract (disorder)	371970002
SNOMED	Acute myeloid leukemia with maturation FAB M2 (disorder)	359648001
SNOMED	Neuroblastoma of brain (disorder)	281560004
SNOMED	Primary malignant neoplasm of occipital bone (disorder)	93927001
SNOMED	Secondary malignant neoplasm of skin of lower limb (disorder)	94565008
SNOMED	Localized malignant reticulohistiocytoma (disorder)	255191003
SNOMED	Primary lymphoma of conjunctiva (disorder)	763477007
SNOMED	Primary malignant neoplasm of peripheral nerves of upper limb (disorder)	109931000
SNOMED	Primary malignant neoplasm of vulva with widespread metastatic disease (disorder)	423987006
SNOMED	Primary malignant neoplasm of peripheral nerves of face (disorder)	109925003
SNOMED	Follicular non-Hodgkins mixed small cleaved and large cell lymphoma (disorder)	188672005
SNOMED	Primary malignant neoplasm of upper inner quadrant of female breast (disorder)	94115006
SNOMED	Anaplastic lymphoma kinase positive large B-cell lymphoma (disorder)	715950008
SNOMED	Metastasis from malignant tumor of bladder (disorder)	314995004
SNOMED	Primary malignant neoplasm of meninges (disorder)	109915008
SNOMED	Malignant tumor of prostate (disorder)	399068003
SNOMED	Malignant tumor of large intestine (disorder)	363510005
SNOMED	Clinical stage A chronic lymphocytic leukaemia (disorder)	8.63741E+14
SNOMED	Primary adenocarcinoma of cardia of stomach (disorder)	721630000
SNOMED	Primary malignant neoplasm of jaw (disorder)	93845000
SNOMED	Hodgkins disease nodular sclerosis of spleen (disorder)	93518009
SNOMED	Primary malignant mesenchymal neoplasm of stomach (disorder)	721643000
SNOMED	Secondary malignant neoplasm of parietal lobe (disorder)	94471000
SNOMED	Pre B-cell acute lymphoblastic leukemia in remission (disorder)	425941003
SNOMED	Light chain myeloma (disorder)	277579002
SNOMED	B-cell chronic lymphocytic leukemia variant (disorder)	277474005
SNOMED	Secondary malignant neoplasm of tarsal bone (disorder)	94620005
SNOMED	Primary adnexal carcinoma of skin (disorder)	721540005
SNOMED	Lymphosarcoma of lymph nodes of multiple sites (disorder)	188507008
SNOMED	Hodgkins disease lymphocytic depletion of lymph nodes of inguinal region AND/OR lower limb (disorder)	93489007
SNOMED	Primary malignant neoplasm of blood vessel of forearm (disorder)	93697005
SNOMED	Myelodysplastic/myeloproliferative disease	445738007
SNOMED	Siewert type II adenocarcinoma of esophagogastric junction (disorder)	440501006
SNOMED	Adenosquamous carcinoma of cervix (disorder)	254888007
SNOMED	Overlapping malignant neoplasm of retroperitoneum and peritoneum (disorder)	109851002
SNOMED	Primary adenoid squamous cell carcinoma of larynx (disorder)	707425008
SNOMED	Primary pleomorphic carcinoma of lung (disorder)	707458004
SNOMED	Malignant epithelial neoplasm of small intestine (disorder)	448664009
SNOMED	Hepatoblastoma (disorder)	109843000

SNOMED	Malignant tumor of junctional zone of tongue (disorder)	187644001
SNOMED	Secondary adenocarcinoma of bone (disorder)	9.1281E+13
SNOMED	Secondary malignant neoplasm of frontal bone (disorder)	94308006
SNOMED	Malignant tumor involving rectum by direct extension from ovary (disorder)	369450004
SNOMED	Malignant melanoma of retina (disorder)	423673009
SNOMED	Secondary malignant neoplasm of retropharyngeal lymph nodes (disorder)	94520004
SNOMED	Malignant tumor of buccal mucosa (disorder)	363386008
SNOMED	Primary non-mucinous bronchiolo-alveolar carcinoma of lung (disorder)	707469001
SNOMED	Mycosis fungoides of spleen (disorder)	94714002
SNOMED	Secondary malignant neoplasm of superficial inguinal lymph nodes (disorder)	94612008
SNOMED	Malignant tumor involving urethra by separate metastasis from bladder (disorder)	369467006
SNOMED	Nodular lymphoma (disorder)	269476000
SNOMED	Primary malignant neoplasm of back (disorder)	93686005
SNOMED	Metastatic malignant neoplasm to lateral axillary lymph nodes (disorder)	359780007
SNOMED	Hodgkins disease nodular sclerosis of intrathoracic lymph nodes (disorder)	188566001
SNOMED	Primary malignant neoplasm of tibia (disorder)	94099002
SNOMED	Overlapping malignant neoplasm of lip (disorder)	109822001
SNOMED	Malignant neoplasm of lower respiratory tract (disorder)	430621000
SNOMED	Sarcoma of central portion of female breast (disorder)	448436006
SNOMED	Secondary malignant neoplasm of sphenoid bone (disorder)	94598003
SNOMED	Adipocytic liposarcoma (disorder)	404067008
SNOMED	Human epidermal growth factor 2 negative carcinoma of breast (disorder)	431396003
SNOMED	Malignant melanoma stage IV M1c (finding)	9.56551E+14
SNOMED	Secondary malignant neoplasm of postcricoid region (disorder)	94495003
SNOMED	Malignant lymphoma in remission (disorder)	427141003
SNOMED	Reticulosarcoma of lymph nodes of multiple sites (disorder)	95230004
SNOMED	Primary malignant neoplasm of lower lobe bronchus or lung (disorder)	372110008
SNOMED	Mesothelioma (malignant clinical disorder) (disorder)	109378008
SNOMED	Overlapping malignant neoplasm of mediastinum and pleura (disorder)	109374005
SNOMED	Malignant tumor involving left fallopian tube by separate metastasis from vagina (disorder)	369546009
SNOMED	Secondary malignant neoplasm of supraclavicular region (disorder)	94615005
SNOMED	Primary malignant astrocytoma of central nervous system (disorder)	1.47101E+14
SNOMED	Primary malignant neoplasm of soft tissues of thorax (disorder)	94062002
SNOMED	Malignant tumor involving rectum by separate metastasis from ovary (disorder)	369457001
SNOMED	Secondary malignant neoplasm of pleura (disorder)	94493005
SNOMED	Malignant tumor of mastoid air cells (disorder)	363424007
SNOMED	Primary cystadenocarcinoma of oropharynx (disorder)	707589002
SNOMED	Hodgkins disease of intrapelvic lymph nodes (disorder)	93521006
SNOMED	Hodgkins disease lymphocytic depletion of lymph nodes of head (disorder)	426885008
SNOMED	Follicular malignant lymphoma - small cleaved cell (disorder)	277625002
SNOMED	Primary adenocarcinoma of sublingual gland (disorder)	737308008
SNOMED	Malignant melanoma stage IIIA (finding)	9.56431E+14
SNOMED	Metastasis to peripheral nerve (disorder)	255124004
SNOMED	Malignant tumor of posterior commissure (disorder)	254513004
SNOMED	Overlapping malignant neoplasm of skin (disorder)	109264009
SNOMED	Spindle cell squamous carcinoma of skin (disorder)	254653005
SNOMED	Secondary malignant neoplasm of areola of female breast (disorder)	94176003
SNOMED	Carcinoma of anterior part of floor of mouth (disorder)	254427006
SNOMED	Secondary malignant neoplasm of perirenal tissue (disorder)	94485005
SNOMED	Invasive hydatidiform mole (disorder)	416669000
SNOMED	Orbital lymphoma (disorder)	13048006
SNOMED	Mast cell leukemia affecting skin (disorder)	404172001

SNOMED	Malignant melanoma of skin of popliteal area (disorder)	93645003
SNOMED	Patch/plaque stage mycosis fungoides (disorder)	404107008
SNOMED	Extranodal marginal zone B-cell lymphoma of mucosa-associated lymphoid tissue (disorder)	445269007
SNOMED	Local recurrence of malignant tumor of gallbladder (disorder)	314962005
SNOMED	Oligodendroglioma of brain (disorder)	254940005
SNOMED	Adult T-cell leukemia/lymphoma of skin (disorder)	721762007
SNOMED	Primary chondrosarcoma of articular cartilage of rib (disorder)	723847006
SNOMED	Malignant tumor involving bladder by direct extension from fallopian tube (disorder)	369470005
SNOMED	Secondary malignant neoplasm of rectosigmoid junction (disorder)	94509004
SNOMED	Primary undifferentiated carcinoma of oropharynx (disorder)	721607006
SNOMED	Adenocarcinoma of pelvis (disorder)	423746001
SNOMED	Accelerated phase chronic myeloid leukemia (disorder)	413389003
SNOMED	Overlapping primary malignant neoplasm of bone and articular cartilage of right upper limb (disorder)	3.53671E+14
SNOMED	Secondary malignant neoplasm of trapezium (disorder)	94644007
SNOMED	Malignant tumor involving right ovary by separate metastasis from uterus (disorder)	369572000
SNOMED	Primary salivary gland type carcinoma of lung (disorder)	707467004
SNOMED	Malignant neoplasm of descended testis (disorder)	1.09026E+15
SNOMED	Primary malignant neoplasm of posterior hypopharyngeal wall (disorder)	93968005
SNOMED	Secondary malignant neoplasm of axilla (disorder)	94180008
SNOMED	Primary liposarcoma of peritoneum (disorder)	722516000
SNOMED	Extraskeletal osteosarcoma (disorder)	404077005
SNOMED	Secondary malignant neoplasm of mastoid air cells (disorder)	94404007
SNOMED	Adenocarcinoma of right lung (disorder)	1.59564E+16
SNOMED	Secondary malignant neoplasm of coeliac lymph nodes (disorder)	1.09019E+15
SNOMED	Primary papillary adenocarcinoma of trachea (disorder)	707472008
SNOMED	Primary squamous cell carcinoma of branchial cleft (disorder)	707531008
SNOMED	Secondary malignant neoplasm of brain (disorder)	94225005
SNOMED	Malignant mast cell tumor of lymph nodes of multiple sites (disorder)	93206004
SNOMED	Primary sarcoma of tongue (disorder)	424849005
SNOMED	Malignant neoplasm of lower-inner quadrant of female breast (disorder)	188153009
SNOMED	Malignant neoplasm of connective and soft tissues of thoracic spine (disorder)	188013008
SNOMED	Clear cell carcinoma of kidney (disorder)	254915003
SNOMED	Primary malignant neoplasm of lower outer quadrant of female breast (disorder)	93876006
SNOMED	Secondary malignant neoplasm of paratracheal lymph nodes (disorder)	1.09008E+15
SNOMED	Ceruminous gland adenocarcinoma of skin (disorder)	403951006
SNOMED	Transitional cell carcinoma of right renal pelvis (disorder)	1.0823E+15
SNOMED	Secondary malignant neoplasm of choroid (disorder)	94254004
SNOMED	Secondary malignant neoplasm of cervical vertebral column (disorder)	94250008
SNOMED	Primary squamous cell carcinoma of left ear (disorder)	1.08207E+15
SNOMED	Diffuse non-Hodgkins lymphoma large cell (disorder)	109969005
SNOMED	Malignant melanoma arising in congenital nevus (disorder)	254734009
SNOMED	Adenocarcinoma of head and neck (disorder)	441535001
SNOMED	Primary sarcoma of left lower limb (disorder)	1.08171E+15
SNOMED	Recurrent primary malignant neoplasm of right female breast (disorder)	1.08156E+15
SNOMED	Recurrent primary malignant neoplasm of left female breast (disorder)	1.08155E+15
SNOMED	Malignant neoplasm of genital structure (disorder)	430556008
SNOMED	Primary neuroendocrine carcinoma of cervix uteri (disorder)	722683006
SNOMED	Primary malignant neoplasm of muscle of shoulder (disorder)	93909004
SNOMED	Hodgkins paragranuloma of lymph nodes of head face AND/OR neck (disorder)	93542008
SNOMED	Primary squamous cell carcinoma of lateral wall of oropharynx (disorder)	707535004
SNOMED	Infiltrating ductal carcinoma of upper outer quadrant of right female breast (disorder)	1.08024E+15
SNOMED	Malignant neoplasm of breast lower outer quadrant (disorder)	373081007

SNOMED	Mast cell malignancy of lymph nodes (disorder)	397011009
SNOMED	Peripheral T-cell lymphoma - pleomorphic small cell (disorder)	277651000
SNOMED	Infiltrating ductal carcinoma of upper inner quadrant of left female breast (disorder)	1.08015E+15
SNOMED	Malignant tumor involving an organ by direct extension from uterus (disorder)	369600000
SNOMED	Primary adenocarcinoma of upper lobe of right lung (disorder)	1.07896E+15
SNOMED	Malignant tumor involving prostate by direct extension from bladder (disorder)	369485004
SNOMED	Acute monoblastic leukemia in remission (disorder)	698646006
SNOMED	Primary basaloid squamous cell carcinoma of trachea (disorder)	707390005
SNOMED	Primary adenosquamous carcinoma of endometrium (disorder)	1.07791E+14
SNOMED	Primary basaloid carcinoma of hypopharynx (disorder)	707486007
SNOMED	Malignant tumor of ciliary body (disorder)	188263008
SNOMED	Malignant melanoma of finger (disorder)	188064009
SNOMED	Secondary malignant neoplasm of thymus (disorder)	94632009
SNOMED	Neuroblastoma of central nervous system (disorder)	734099007
SNOMED	Bullous mycosis fungoides (disorder)	404115006
SNOMED	Secondary malignant neoplasm of skin of shoulder and arm (disorder)	188458007
SNOMED	Sarcoma of femur (disorder)	448712008
SNOMED	Malignant neoplastic disease in mother complicating childbirth (disorder)	1.07453E+16
SNOMED	Secondary malignant neoplasm of blood vessel of trunk (disorder)	94209000
Read	Burkitts lymphoma	B602.
Read	Plasma cell disorder	Xa0SI
Read	Sezarys disease of lymph nodes of head face and neck	B6221
Read	Cerebral metastasis	XM1Pr
Read	Malignant neoplasm of unspecified site	B59..
Read	Acute leukaemia	Xa9AM
Read	Secondary and unspecified malignant neoplasm of supratrochlear lymph nodes	B5631
Read	Lymphocyte depleted classical Hodgkin lymphoma	B61A.
Read	Leukaemia: [lymphoid] or [monocytic] or [myeloid] or [specific cell type]	XE20R
Read	Multiple myeloma etc.	XE20N
Read	Malignant neoplasm of colon NOS	XE1vV
Read	Secondary malignant neoplasm of unknown site	B594.
Read	Malignant neoplasm of retroperitoneum and peritoneum NOS	B18z.
Read	Follicular lymphoma grade 3	XaZdD
Read	Acute myeloid leukaemia with 11q23 abnormality	XaYj9
Read	Malignant neoplasms of independent (primary) multiple sites	B592.
Read	Malignant neoplasm overlapping lesion of brain and other part of central nervous system	B52W.
Read	Malignant neoplasm of phalanges of foot	B308D
Read	Mantle cell lymphoma	XaPQD
Read	Malignant neoplasm of cerebral dura mater	B5210
Read	Malignant neoplasm of descended testis NOS	XaMeL
Read	Secondary malignant neoplasm of retroperitoneum	B5760
Read	Lambda light chain myeloma	XaELI
Read	Malignant neoplasm of spleen NOS	B1z1z
Read	Secondary and unspecified malignant neoplasm of intrathoracic lymph nodes NOS	B561z
Read	Malignant neoplasm of lower lip inner aspect NOS	B003z
Read	Malignant neoplasm of eye NOS	B50z.
Read	Malignant neoplasm of nasal cavities	B200.
Read	Nodular lymphoma of intra-abdominal lymph nodes	B6203
Read	Lymphoid and histiocytic malignancy NOS	XE1vq
Read	Malignant neoplasm of anal canal	B142.
Read	Malignant neoplasm of frontal sinus	B204.
Read	Malignant neoplasm of second metacarpal bone	B3059

Read	Mycosis fungoides of intra-abdominal lymph nodes	B6213
Read	Malignant neoplasm of epiglottis NOS	XaBLv
Read	Myelosclerosis with myeloid metaplasia	XaBBt
Read	Hodgkins disease mixed cellularity of lymph nodes of multiple sites	B6158
Read	Malignant melanoma of upper limb or shoulder NOS	B326z
Read	[X]Malignant neoplasm of male genital organ unspecified	Byu82
Read	Myelodysplastic syndrome	Xa0SY
Read	Bowel scope (flexible sigmoidoscopy) screen: cancer detected	XaabR
Read	Clinical stage B chronic lymphocytic leukaemia	Xaa1O
Read	Malignant histiocytosis of intrapelvic lymph nodes	B6236
Read	Malignant neoplasm of trigone of urinary bladder	B490.
Read	Malignant neoplasm of cheek mucosa	B050.
Read	Hepatosplenic T-cell lymphoma	B62E5
Read	Secondary malignant neoplasm of brain	B5830
Read	Granulocytic sarcoma	B6531
Read	Secondary malignant neoplasm of other specified sites	XE2vT
Read	Malignant neoplasm of oropharynx	B06..
Read	Hyperviscosity syndrome	Xa0Sp
Read	Malignant neoplasm of the pouch of Douglas	B18y6
Read	Malignant neoplasm of kidney parenchyma	B4A0.
Read	IgG monoclonal gammopathy of uncertain significance	Xa36e
Read	5Q minus syndrome	Xa0Sh
Read	Lymphocyte-rich classical Hodgkin lymphoma	B61B.
Read	Refractory anaemia with excess blasts in transformation	Xa0Sg
Read	Common acute lymphoblastic leukaemia	Xa0SF
Read	Pre B-cell acute lymphoblastic leukaemia	Xa0SE
Read	Large granular lymphocytic leukaemia	Xa0SB
Read	Reticulosarcoma of intrapelvic lymph nodes	B6006
Read	Splenic lymphoma with villous lymphocytes	Xa0Rp
Read	Richters syndrome	Xa0Ro
Read	T-cell chronic lymphocytic leukaemia	Xa0Rk
Read	Malignant neoplasm of upper lip external	B0000
Read	Malignant tumour of unknown origin	X78e9
Read	Hodgkins disease lymphocytic depletion of spleen	B6167
Read	Lambda light chain myeloma	B6303
Read	Malignant neoplasm of middle third of oesophagus	B104.
Read	Malignant neoplasm of thorax	B551.
Read	Malignant neoplasm overlapping lesion of brain	B51y2
Read	[X]Non-Hodgkins lymphoma unspecified type	ByuDF
Read	Malignant neoplasm of cloacogenic zone	B1420
Read	Malignant neoplasm of other site of female breast	B34y.
Read	Malignant neoplasm of broad ligament	B442.
Read	Malignant neoplasm of palatine tonsil	B0601
Read	[X]Other specified types of non-Hodgkins lymphoma	ByuD3
Read	[X]Other types of diffuse non-Hodgkins lymphoma	ByuD2
Read	[X]Other Hodgkins disease	ByuD0
Read	Malignant neoplasm of other specified site of digestive tract and peritoneum	B1zy.
Read	Malignant neoplasm of tympanic antrum	B2012
Read	Malignant melanoma of ear and external auricular canal NOS	B322z
Read	[X]Malignant neoplasm of thyroid and other endocrine glands	ByuB.
Read	[X]Kaposis sarcoma unspecified	Byu53
Read	[X]Malignant neoplasm of overlapping lesion of male genital organs	Byu81

Read	[X]Malignant neoplasm of female genital organ unspecified	Byu73
Read	Malignant neoplasm of supraglottis	B211.
Read	Hodgkins disease lymphocytic depletion	B616.
Read	[X]Malignant neoplasm of female genital organs	Byu7.
Read	Malignant melanoma of scalp and neck NOS	B324z
Read	Malignant neoplasm of mesocolon	B18y0
Read	Secondary malignant neoplasm of skin of face	B5821
Read	Malignant melanoma stage IIIB	XacIa
Read	[X]Mesothelioma of other sites	Byu50
Read	Malignant neoplasm of connective and soft tissue of perineum	B3152
Read	Hodgkins granuloma of unspecified site	B6110
Read	[X]Malignant melanoma of other and unspecified parts of face	Byu40
Read	[X]Melanoma and other malignant neoplasms of skin	Byu4.
Read	[X]Malignant neoplasm of overlapping lesion of bone and articular cartilage	Byu32
Read	Malignant neoplasm of vagina	B450.
Read	Secondary malignant neoplasm of colon	B5750
Read	Malignant neoplasm of bronchus or lung NOS	XE1vc
Read	True histiocytic lymphoma	B62x6
Read	[X]Additional neoplasm classification terms	Byu..
Read	Hodgkins disease nodular sclerosis of lymph nodes of axilla and upper limb	B6144
Read	Malignant neoplasm of lymphatic or haematopoietic tissue otherwise specified	B6y..
Read	Juvenile myelomonocytic leukaemia	B693.
Read	Chronic myelomonocytic leukaemia	B691.
Read	Acute myelomonocytic leukaemia	B690.
Read	Chronic leukaemia NOS	B681.
Read	Malignant neoplasm of retained testis	B4701
Read	Lymphosarcoma cell leukaemia	B67y0
Read	Other specified leukaemia	B67..
Read	Other monocytic leukaemia NOS	B66yz
Read	Malignant neoplasm of lower gum	B031.
Read	Other specified reticulosarcoma or lymphosarcoma	B60y.
Read	Other myeloid leukaemia	B65y.
Read	Malignant neoplasm of other site of cervix	B41y.
Read	Malignant neoplasm of endocardium	B2410
Read	Malignant neoplasm of eye	B50..
Read	Chronic erythraemia	XE1vr
Read	Clinical stage B chronic lymphocytic leukaemia	B6412
Read	Malignant neoplasm of rectum	B141.
Read	B-cell chronic lymphocytic leukaemia	B6410
Read	Mycosis fungoides of the lymph nodes of head face and neck	B6211
Read	Hepatocellular carcinoma	B1503
Read	Sarcoma of dendritic cells	B62A.
Read	Lymphoid and histiocytic malignancy NOS	B62zz
Read	Unspecified malignant neoplasm of lymphoid and histiocytic tissue of intra-abdominal lymph nodes	B62z3
Read	Malignant neoplasm of fixed part of tongue NOS	B010z
Read	Follicular non-Hodgkins mixed small cleaved and large cell lymphoma	B6271
Read	Malignant neoplasm of lip inner aspect NOS	B004z
Read	Malignant melanoma of upper arm	B3261
Read	Chronic myeloid leukaemia	B651.
Read	Malignant melanoma of hand	B3263
Read	Malignant lymphoma NOS of unspecified site	B62y0
Read	Malignant neoplasm of genitourinary organ	B4...

Read	Lymphoepithelioid lymphoma	B62x1
Read	Malignant neoplasm overlapping lesion of major salivary glands	B023.
Read	Other non-follicular lymphoma	B62Fy
Read	Hodgkins sarcoma of spleen	B6127
Read	Enteropathy-associated T-cell lymphoma	B62E6
Read	Cutaneous T-cell lymphoma	B62E3
Read	Malignant neoplasm of other specified site of stomach	B11y.
Read	Malignant neoplasm of connective and soft tissue of finger	B3114
Read	Hodgkins paragranuloma of lymph nodes of axilla and upper limb	B6104
Read	Malignant neoplasm overlapping lesion of heart mediastinum and pleura	B25..
Read	Follicular lymphoma grade 3	B6282
Read	Hodgkins paragranuloma	B610.
Read	Malignant neoplasm of cranial nerves	B520.
Read	Chloroma	B6530
Read	Extranodal marginal zone B-cell lymphoma of mucosa-associated lymphoid tissue	B627F
Read	Malignant neoplasm of orbit NOS	B501z
Read	Diffuse non-Hodgkins lymphoblastic (diffuse) lymphoma	B6277
Read	Diffuse non-Hodgkins immunoblastic (diffuse) lymphoma	B6276
Read	Follicular non-Hodgkins small cleaved cell lymphoma	B6270
Read	Malignant neoplasm of pituitary gland and craniopharyngeal duct	B542.
Read	Malignant neoplasm of ulna	B3044
Read	Mast cell malignancy of spleen	B6267
Read	Mast cell malignancy of lymph nodes of inguinal region and lower limb	B6265
Read	Malignant neoplasm of dorsal surface of tongue	B011.
Read	Mast cell malignancy of lymph nodes of head face and neck	B6261
Read	Malignant neoplasm of other gallbladder and extrahepatic bile ducts	B16y.
Read	Malignant neoplasm of overlapping lesion of vulva	B45y0
Read	Letterer-Siwe disease of spleen	B6257
Read	Hodgkins disease lymphocytic-histiocytic predominance of lymph nodes of head face and neck	B6131
Read	Letterer-Siwe disease of lymph nodes of head face and neck	B6251
Read	Malignant neoplasm overlapping lesion of eye and adnexa	B508.
Read	Malignant neoplasm of thoracic vertebra	B3021
Read	Leukaemic reticuloendotheliosis of spleen	B6247
Read	Malignant neoplasm of other major salivary glands	B02y.
Read	Malignant histiocytosis of lymph nodes of inguinal region and lower limb	B6235
Read	Malignant neoplasm of prostate	B46..
Read	Malignant histiocytosis of lymph nodes of multiple sites	B6238
Read	Malignant histiocytosis of spleen	B6237
Read	Malignant neoplasm of prepylorus of stomach	B1110
Read	Leukaemic reticuloendotheliosis of unspecified sites	B6240
Read	Malignant neoplasm of uvula	B054.
Read	Malignant histiocytosis of lymph nodes of head face and neck	B6231
Read	Malignant neoplasm overlapping lesion of larynx	B214.
Read	Malignant neoplasm of postcricoid region	B080.
Read	Hodgkins sarcoma NOS	B612z
Read	[X]Other sarcomas of the liver	Byu10
Read	Burkitts lymphoma of intrapelvic lymph nodes	B6026
Read	Acquired renal cyst with neoplastic change	K1323
Read	Sezarys disease of unspecified site	B6220
Read	Letterer-Siwe disease of intrapelvic lymph nodes	B6256
Read	Mycosis fungoides of spleen	B6217
Read	Malignant neoplasm of anterior portion of floor of mouth	B040.

Read	Mycosis fungoides of lymph nodes of axilla and upper limb	B6214
Read	Malignant neoplasm of vestibule of mouth	B051.
Read	Hereditary nonpolyposis colon cancer	B139.
Read	Nodular lymphoma of intrapelvic lymph nodes	B6206
Read	Malignant neoplasm of penis and other male genital organs	B48..
Read	Malignant neoplasm of corpus uteri excluding isthmus	B430.
Read	Hodgkins disease NOS	B61zz
Read	Hodgkins disease NOS of spleen	B61z7
Read	Malignant neoplasm of frontal lobe	B511.
Read	Secondary malignant neoplasm of skin of hip and leg	B5825
Read	Hodgkins disease NOS of lymph nodes of head face and neck	B61z1
Read	Hodgkins disease lymphocytic depletion NOS	B616z
Read	Malignant ascites	X30AD
Read	Malignant neoplasm overlapping lesion of penis	B487.
Read	[X]Malignant neoplasm of bones and articular cartilage of limb unspecified	Byu31
Read	[X]Malignant neoplasm of respiratory and intrathoracic organs	Byu2.
Read	Malignant neoplasm of parametrium	B443.
Read	Hodgkins disease mixed cellularity NOS	B615z
Read	Other leukaemia of unspecified cell type	B68y.
Read	Unifocal Langerhans-cell histiocytosis	B62C.
Read	Malignant neoplasm soft tissues of cervical spine	B3105
Read	Macroglobulinaemia	C333.
Read	Malignant neoplasm of rectum rectosigmoid junction and anus	B14..
Read	Hodgkins disease mixed cellularity of intrapelvic lymph nodes	B6156
Read	Secondary and unspecified malignant neoplasm of intra-abdominal lymph nodes	B562.
Read	Malignant neoplasm of sigmoid colon	B133.
Read	Hodgkins disease lymphocytic-histiocytic predominance of intrapelvic lymph nodes	B6136
Read	Primary malignant neoplasm of liver	B150.
Read	Hodgkins disease lymphocytic-histiocytic predominance	B613.
Read	Hodgkins sarcoma of lymph nodes of inguinal region and lower limb	B6125
Read	Hodgkins sarcoma of intra-abdominal lymph nodes	B6123
Read	Hodgkins sarcoma of intrathoracic lymph nodes	B6122
Read	Malignant neoplasm of tonsillar pillar	B062.
Read	Secondary and unspecified malignant neoplasm of lymph nodes	XE2xB
Read	Hodgkins granuloma of intrapelvic lymph nodes	B6116
Read	Primary cutaneous CD30-positive T-cell proliferations	B62EA
Read	Hodgkins granuloma of lymph nodes of head face and neck	B6111
Read	Hodgkins paragranuloma of spleen	B6107
Read	Hodgkins paragranuloma of intra-abdominal lymph nodes	B6103
Read	Mycosis fungoides of intrathoracic lymph nodes	B6212
Read	Malignant neoplasm of adenoid	B0710
Read	Follicular lymphoma grade 1	B6280
Read	Malignant neoplasm of liver and intrahepatic bile ducts NOS	B15z.
Read	Hodgkins disease lymphocytic depletion of lymph nodes of inguinal region and lower limb	B6165
Read	Malignant melanoma of foot	B3277
Read	Solitary plasmacytoma	B6304
Read	Burkitts lymphoma of intra-abdominal lymph nodes	B6023
Read	Burkitts lymphoma of intrathoracic lymph nodes	B6022
Read	Hodgkins sarcoma of lymph nodes of head face and neck	B6121
Read	Malignant neoplasm of lower-inner quadrant of female breast	B343.
Read	Disseminated malignancy NOS	B590.
Read	Malignant neoplasm of tongue	B01..

Read	Hodgkins disease NOS of lymph nodes of multiple sites	B61z8
Read	Malignant ascites	B5762
Read	Secondary and unspecified malignant neoplasm of deep parotid lymph nodes	B5604
Read	Reticulosarcoma of unspecified site	B6000
Read	Malignant neoplasm of intrahepatic canaliculi	B1513
Read	Malignant neoplasm of middle lobe bronchus	B2230
Read	Kaposis sarcoma unspecified	B59zX
Read	Malignant tumour of unknown origin	B595.
Read	Reticulosarcoma of lymph nodes of axilla and upper limb	B6004
Read	Secondary malignant neoplasm of penis	B58y7
Read	Hodgkins disease NOS of intra-abdominal lymph nodes	B61z3
Read	Mucosa-associated lymphoma	B6279
Read	Malignant neoplasm overlapping lesion of bladder	B49y0
Read	Juvenile chronic myeloid leukaemia	Xa0SV
Read	Malignant neoplasm of malar bone	B3002
Read	Secondary malignant neoplasm of spinal cord	B5831
Read	Malignant histiocytosis	B623.
Read	Secondary malignant neoplasm of skin NOS	B582z
Read	Secondary malignant neoplasm of cervix uteri	B58y2
Read	[X]Other types of follicular non-Hodgkins lymphoma	ByuD1
Read	Drash syndrome	X30I1
Read	Siewert type III adenocarcinoma	B119.
Read	Secondary malignant neoplasm of skin	B582.
Read	Nodular sclerosis classical Hodgkin lymphoma	B618.
Read	Malignant melanoma of external surface of nose	B3234
Read	Malignant melanoma stage IIB	B32B.
Read	Secondary malignant neoplasm of other urinary organs	B581.
Read	Acute myeloid leukaemia	B650.
Read	Malignant neoplasm of urethra	B4A3.
Read	Malignant neoplasm of other specified site of stomach NOS	B11yz
Read	[X]Malignant melanoma of skin unspecified	Byu41
Read	Secondary malignant neoplasm of duodenum	B5740
Read	Malignant neoplasm of pyriform sinus	B081.
Read	Secondary and unspecified malignant neoplasm of external iliac lymph nodes	B5624
Read	Mast cell leukaemia	B673.
Read	Diffuse non-Hodgkins centroblastic lymphoma	B627D
Read	Secondary and unspecified malignant neoplasm of circumflex iliac lymph nodes	B5652
Read	Secondary and unspecified malignant neoplasm of internal iliac lymph nodes	B5650
Read	Burkitts lymphoma of lymph nodes of axilla and upper limb	B6024
Read	Malignant neoplasm of connective and soft tissue of lower leg	B3123
Read	Malignant neoplasm of seminal vesicle	B48y0
Read	Malignant neoplasm of cervical stump	B41y0
Read	Malignant neoplasm of anterior wall of stomach NEC	B11y0
Read	Secondary and unspecified malignant neoplasm of axilla and upper limb lymph nodes	B563.
Read	Secondary and unspecified malignant neoplasm of inferior mesenteric lymph nodes	B5622
Read	Enteropathy-associated T-cell lymphoma	Xa0Tu
Read	Follicular lymphoma	B628.
Read	Chronic myeloid leukaemia BCR/ABL positive	B6511
Read	Hodgkins disease lymphocytic depletion of lymph nodes of head face and neck	B6161
Read	Malignant neoplasm of glossopalatine fold	B0621
Read	Secondary and unspecified malignant neoplasm of lymph nodes of head face and neck NOS	B560z
Read	Peripheral T-cell lymphoma	B62x2

Read	Follicular lymphoma grade 3b	B6284
Read	Secondary and unspecified malignant neoplasm of lymph nodes of head face and neck	B560.
Read	Malignant neoplasm of hepatic duct	B1611
Read	Malignant neoplasm of back NOS	B55y0
Read	Malignant neoplasm of lower limb NOS	B555.
Read	Malignant neoplasm of abdomen	B552.
Read	Malignant neoplasm of cardio-oesophageal junction of stomach	B1101
Read	Malignant neoplasm overlapping lesion of stomach	B117.
Read	Malignant neoplasm of axilla NOS	B5510
Read	Malignant neoplasm of jaw NOS	B5503
Read	Seminoma of descended testis	XaELK
Read	Malignant neoplasm of head neck and face	B550.
Read	Malignant neoplasm of endocrine gland or related structure NOS	B54z.
Read	Neuroblastoma	B546.
Read	Malignant neoplasm of carotid body	B544.
Read	Malignant neoplasm of lower lip vermilion border	B001.
Read	Refractory anaemia with multilineage dysplasia	XaYv2
Read	Malignant neoplasm of nervous system NOS	B52z.
Read	Other lymphoid leukaemia NOS	B64yz
Read	Malignant neoplasm of peripheral nerve of pelvis	B5245
Read	Malignant neoplasm of peripheral nerve of thorax	B5243
Read	Malignant lymphoma NOS	B62yz
Read	Malignant neoplasm of cerebral meninges NOS	B521z
Read	Secondary malignant neoplasm of skin of breast	B5826
Read	Secondary malignant neoplasm of bone and bone marrow	B585.
Read	Malignant neoplasm of lower lip buccal aspect	B0030
Read	Malignant neoplasm of cerebral peduncle	B5170
Read	Mature T/NK-cell lymphoma	B62E0
Read	Malignant neoplasm of medial cuneiform	B3083
Read	Acute myeloid leukaemia with myelodysplasia-related changes	XaYj4
Read	Malignant neoplasm of hippocampus	B5120
Read	Chronic eosinophilic leukaemia	B6510
Read	Malignant neoplasm of mesorectum	B18y2
Read	Malignant neoplasm of corpus striatum	B5102
Read	Malignant neoplasm of cerebrum (excluding lobes and ventricles)	B510.
Read	Other and unspecified leukaemia NOS	B67yz
Read	Malignant lymphoma NOS of lymph nodes of multiple sites	B62y8
Read	Malignant neoplasm of carpal bone - triquetrum	B3052
Read	Malignant neoplasm of lacrimal duct NOS	B507z
Read	Malignant neoplasm of retina	B505.
Read	Malignant neoplasm of other and unspecified site NOS	B5z..
Read	Teratoma of undescended testis	B4703
Read	Malignant neoplasm of corpus callosum	B51y0
Read	Malignant neoplasm of crystalline lens	B5002
Read	Malignant neoplasm of iris	B5001
Read	Malignant neoplasm of peripheral nerves and autonomic nervous system unspecified	B524W
Read	Lymphoid leukaemia NOS	B64z.
Read	Myeloproliferative disorder	Xa0SP
Read	Siewert type II adenocarcinoma	XaOqX
Read	Malignant neoplasm of renal calyces	B4A10
Read	Malignant neoplasm of kidney and other unspecified urinary organs	B4A..
Read	Malignant neoplasm of other and unspecified site otherwise specified	B5y..

Read	Secondary malignant neoplasm of other specified sites	B58y.
Read	Malignant neoplasm of urachus	B497.
Read	[X]Malignant neoplasm of peripheral nerves and autonomic nervous system unspecified	Byu56
Read	Teratoma of undescended testis	XaELM
Read	Acute erythroid leukaemia	B676.
Read	Malignant neoplasm of other male genital organ NOS	B48yz
Read	Malignant neoplasm of posterior wall of nasopharynx NOS	B071z
Read	Malignant neoplasm of carpal bone - capitate	B3056
Read	Myelosclerosis with myeloid metaplasia	B6y1.
Read	Malignant neoplasm of body of penis	B482.
Read	Malignant neoplasm of testis NOS	B47z.
Read	Malignant neoplasm of arytenoid cartilage	B2130
Read	Malignant neoplasm of overlapping lesion of urinary organs	B4Ay0
Read	Acute myelomonocytic leukaemia - eosinophilic variant	Xa3HG
Read	Malignant neoplasm of clitoris	B453.
Read	Malignant neoplasm of descending colon	B132.
Read	Mycosis fungoides	B621.
Read	Secondary and unspecified malignant neoplasm of diaphragmatic lymph nodes	B5612
Read	Myeloid sarcoma	B653.
Read	Malignant neoplasm of kidney or urinary organs NOS	B4Az.
Read	Secondary malignant neoplasm of ovary	B586.
Read	Malignant melanoma stage IIIA	B32D.
Read	Malignant neoplasm of ovary	B440.
Read	Malignant neoplasm of body of uterus NOS	B43z.
Read	Malignant neoplasm of other site of uterine body	B43y.
Read	Malignant neoplasm of overlapping lesion of corpus uteri	B432.
Read	Acute biphenotypic leukaemia	Xa0jn
Read	Malignant neoplasm of isthmus of uterine body	B431.
Read	Malignant neoplasm of brain NOS	B51z.
Read	Malignant neoplasm of other site of cervix NOS	B41yz
Read	Chronic monocytic leukaemia	B661.
Read	Reticulosarcoma or lymphosarcoma NOS	B60z.
Read	Hodgkins disease lymphocytic-histiocytic predominance of intra-abdominal lymph nodes	B6133
Read	Malignant neoplasm of ciliary body	B5000
Read	Local recurrence of malignant tumour of breast	B36..
Read	Malignant neoplasm of peripheral nerves of upper limb including shoulder	B5241
Read	Secondary malignant neoplasm of liver	B577.
Read	Malignant neoplasm of costal cartilage	B3033
Read	Malignant neoplasm of rib sternum and clavicle NOS	B303z
Read	Malignant neoplasm of other sites of nasal cavity middle ear and accessory sinuses	B20y.
Read	Malignant neoplasm of occipital lobe	B514.
Read	Malignant neoplasm of lateral cuneiform	B3085
Read	Sezarys disease NOS	B622z
Read	Malignant neoplasm of humerus	B3042
Read	Malignant neoplasm of major salivary gland NOS	B02z.
Read	Malignant melanoma of skin NOS	B32z.
Read	Malignant neoplasm of endocervical gland	B4101
Read	Malignant neoplasm of cornu of corpus uteri	B4300
Read	Malignant melanoma stage IIA	B32A.
Read	Malignant melanoma stage IB	B329.
Read	Malignant neoplasm of overlapping lesion of lip	B006.
Read	Malignant neoplasm overlapping lesion of accessory sinuses	B206.

Read	Malignant neoplasm of long bones of leg	B307.
Read	Malignant melanoma of thumb	B3265
Read	Malignant melanoma of shoulder	B3260
Read	Malignant neoplasm of ureter	B4A2.
Read	Malignant melanoma of trunk excluding scrotum NOS	B325z
Read	Malignant melanoma of back	B3257
Read	Malignant melanoma of umbilicus	B3256
Read	Secondary malignant neoplasm of respiratory or digestive system NOS	B57z.
Read	Malignant melanoma of breast	B3251
Read	Secondary malignant neoplasm of other specified site NOS	B58yz
Read	Subcutaneous panniculitic T-cell lymphoma	XaYjf
Read	Malignant melanoma of scalp	B3240
Read	Acute myeloblastic leukaemia	B654.
Read	Malignant melanoma of forehead	B3233
Read	Malignant melanoma of eyebrow	B3232
Read	Malignant neoplasm of short bones of leg	B308.
Read	Malignant melanoma of auricle (ear)	B3220
Read	Malignant melanoma of eyelid including canthus	B321.
Read	Hepatosplenic T-cell lymphoma	XaYiN
Read	Malignant neoplasm of connective and soft tissue of trunk unspecified	B316.
Read	Malignant neoplasm of periadrenal tissue	B1800
Read	Malignant neoplasm of lip unspecified external	B00z0
Read	Malignant neoplasm of nipple and areola of female breast	B340.
Read	Basophilic leukaemia	XaBAm
Read	Malignant neoplasm of common bile duct	XE2vN
Read	Malignant melanoma of chest wall	XaEGU
Read	Secondary and unspecified malignant neoplasm of mastoid lymph nodes	B5601
Read	Malignant neoplasm of pyloric canal of stomach	B1111
Read	Malignant neoplasm of connective and soft tissue of abdomen	B314.
Read	Malignant neoplasm of lip oral aspect	B0043
Read	Malignant neoplasm of connective and soft tissue of thorax	B313.
Read	Malignant neoplasm of lower lobe of lung	B2241
Read	Malignant neoplasm of connective tissue of orbit	B5010
Read	Malignant neoplasm of connective and soft tissue of hip and lower limb	B312.
Read	Hodgkins paragranuloma of intrathoracic lymph nodes	B6102
Read	Malignant neoplasm of ischium	B3061
Read	Malignant neoplasm of gum	B03..
Read	Malignant neoplasm of other sites of lip	B00y.
Read	Malignant neoplasm of adrenal cortex	B5400
Read	Malignant neoplasm of nipple or areola of male breast NOS	B350z
Read	Malignant neoplasm of cartilage of ear	B3103
Read	Malignant neoplasm of soft tissue of neck	B3102
Read	Malignant neoplasm of soft tissue of face	B3101
Read	Malignant neoplasm of exocervix	B411.
Read	Malignant neoplasm of overlapping lesion of bone and articular cartilage	B30W.
Read	[X]Malignant neoplasm of eye brain and other parts of central nervous system	ByuA.
Read	Malignant neoplasm of calcaneum	B3082
Read	Malignant neoplasm of long bones of leg NOS	B307z
Read	Burkitts lymphoma NOS	B602z
Read	Malignant neoplasm of oropharynx other specified sites	B06y.
Read	Malignant mast cell tumour NOS	B626z
Read	Malignant neoplasm of pubis	B3062

Read	Malignant overlapping lesion of tongue	B017.
Read	Malignant neoplasm of cardia of stomach NOS	B110z
Read	Malignant neoplasm of pelvic bones sacrum and coccyx	B306.
Read	Adult T-cell leukaemia/lymphoma	XaZfR
Read	Malignant neoplasm of lower lip lipstick area	B0011
Read	Malignant neoplasm of carpal bone - trapezoid	B3055
Read	Multiple myeloma and immunoproliferative neoplasms	B63..
Read	Malignant melanoma of perineum	B3255
Read	Malignant neoplasm of connective and soft tissue of shoulder	B3110
Read	Malignant neoplasm of retroperitoneum and peritoneum	B18..
Read	Malignant neoplasm of acromion	B3041
Read	Mucosa-associated lymphoma	Xa0T8
Read	[X]Malignant neoplasm of overlapping lesion of peripheral nerves and autonomic nervous system	Byu55
Read	Malignant neoplasm of vertebral column NOS	B302z
Read	Malignant neoplasm of lumbar vertebra	B3022
Read	Malignant neoplasm of pelvis NOS	B553z
Read	Malignant neoplasm of sphenoid bone	B3007
Read	Unspecified malignant neoplasm of lymphoid and histiocytic tissue of lymph nodes of head face and neck	B62z1
Read	Malignant neoplasm of epicardium	B2411
Read	Diffuse large B-cell lymphoma	XaZdn
Read	Lymphosarcoma of intra-abdominal lymph nodes	B6013
Read	Malignant neoplasm of parietal bone	B3006
Read	Malignant neoplasm of renal pelvis NOS	B4A1z
Read	Malignant neoplasm of frontal bone	B3001
Read	Malignant melanoma of perianal skin	B3254
Read	Malignant neoplasm of specified parts of peritoneum	B18y.
Read	Neutrophilic leukaemia	XaBBC
Read	Malignant neoplasm of bone connective tissue skin and breast	B3...
Read	[X]Other leukaemia of unspecified cell type	ByuD9
Read	Seminoma of descended testis	B4710
Read	Malignant neoplasm of mediastinum part unspecified	B24X.
Read	Malignant melanoma stage IV M1a	B32G.
Read	Malignant neoplasm of female breast	B34..
Read	Malignant neoplasm of bronchus or lung NOS	B22z.
Read	Malignant neoplasm of first metatarsal bone	B3088
Read	Malignant neoplasm of nipple of male breast	B3500
Read	Acute erythraemia and erythroleukaemia	B670.
Read	Lymphosarcoma of intrathoracic lymph nodes	B6012
Read	Malignant neoplasm of middle lobe of lung	B2231
Read	Mast cell malignancy of lymph nodes of multiple sites	B6268
Read	Malignant neoplasm of mucosa of trachea	B2201
Read	Acute promyelocytic leukaemia	B65y1
Read	Other classical Hodgkin lymphoma	B61C.
Read	Secondary and unspecified malignant neoplasm of bronchopulmonary lymph nodes	B5618
Read	Secondary malignant neoplasm of peritoneum	B5761
Read	Malignant neoplasm of trachea bronchus and lung	B22..
Read	Malignant neoplasm of larynx other specified site	B21y.
Read	Malignant neoplasm of oropharynx NOS	B06z.
Read	Malignant neoplasm of glans penis	B481.
Read	Malignant neoplasm of nasopharynx NOS	B07z.
Read	Malignant neoplasm of subglottis	B212.
Read	Nodular lymphoma of lymph nodes of multiple sites	B6208

Read	Malignant neoplasm of larynx	B21..
Read	Malignant neoplasm of maxillary sinus	B202.
Read	Malignant neoplasm of upper lip vermilion border	B000.
Read	Malignant neoplasm of sphincter of Oddi	B1613
Read	Malignant neoplasm of nasal conchae	B2001
Read	Malignant neoplasm of respiratory tract and intrathoracic organs	B2...
Read	Burkitts lymphoma of unspecified site	B6020
Read	[X]Malignant neoplasm of connective and soft tissue unspecified	Byu59
Read	Malignant neoplasm of heart	B241.
Read	Malignant neoplasm of parietal peritoneum	B18y4
Read	Malignant neoplasm of connective and soft tissue of thorax NOS	B313z
Read	Overlapping malignant lesion of retroperitoneum and peritoneum	B182.
Read	Malignant neoplasm of anus unspecified	B143.
Read	Acute myeloblastic leukaemia without maturation	Xa3Ea
Read	Leukaemic reticuloendotheliosis of intrapelvic lymph nodes	B6246
Read	Malignant neoplasm of other specified sites of pancreas	B17y.
Read	Malignant neoplasm overlapping lesion of pancreas	B175.
Read	Malignant neoplasm of Islets of Langerhans	B174.
Read	[X]Malignant neoplasm of endocrine gland unspecified	ByuB1
Read	Hodgkins disease NOS	B61z.
Read	Hodgkins disease nodular sclerosis of spleen	B6147
Read	Malignant neoplasm of extrahepatic bile ducts NOS	B161z
Read	Hodgkins lymphocytic-histiocytic predominance NOS	B613z
Read	Malignant neoplasm of renal pelvis	B4A1.
Read	Malignant neoplasm of other specified hypopharyngeal site	B08y.
Read	Malignant neoplasm of gallbladder	B160.
Read	Malignant neoplasm of oesophagus NOS	B10z.
Read	Malignant neoplasm of sacrococcygeal region	B5532
Read	Clinical stage A chronic lymphocytic leukaemia	B6411
Read	Hodgkins disease nodular sclerosis of lymph nodes of multiple sites	B6148
Read	Malignant neoplasm of interlobular bile ducts	B1510
Read	Malignant neoplasm of other specified part of oesophagus	B10y.
Read	Malignant neoplasm of central part of female breast	B341.
Read	Malignant mast cell tumours	B626.
Read	Malignant melanoma stage IIC	XacIY
Read	[X]Secondary malignant neoplasm of other and unspecified parts of nervous system	ByuC6
Read	Malignant neoplasm of vestibule of mouth NOS	B051z
Read	Malignant neoplasm of other specified sites of colon	B13y.
Read	Nodular lymphoma of lymph nodes of axilla and upper limb	B6204
Read	Hodgkins paragranuloma NOS	B610z
Read	Malignant neoplasm of uterine adnexa NOS	B44z.
Read	Malignant neoplasm of common bile duct	B1612
Read	Malignant neoplasm of transverse colon	B131.
Read	Malignant neoplasm of other and unspecified female genital organs	B45..
Read	Polycythaemia rubra vera	B934.
Read	Malignant neoplasm of nose NOS	B5502
Read	Malignant neoplasm of connective and soft tissue of pelvis	B315.
Read	Malignant neoplasm of posterior wall of stomach NEC	B11y1
Read	Malignant neoplasm of cauda equina	B525.
Read	Malignant neoplasm of greater curve of stomach unspecified	B116.
Read	Malignant neoplasm of spermatic cord	B485.
Read	Malignant neoplasm of body of stomach	B114.

Read	Malignant neoplasm of fundus of stomach	B113.
Read	Secondary malignant neoplasm of other digestive organ	B57y.
Read	Malignant neoplasm of first metacarpal bone	B3058
Read	Malignant neoplasm of vulva unspecified	B454.
Read	Malignant melanoma of heel	B3276
Read	Malignant neoplasm overlapping lesion of oesophagus	B106.
Read	Malignant neoplasm of thoracic oesophagus	B101.
Read	Malignant neoplasm of oesophagus	B10..
Read	Malignant neoplasm of carpal bone - lunate	B3051
Read	Malignant neoplasm of laryngopharynx	B0z2.
Read	Solitary myeloma	B6301
Read	Malignant neoplasm of lingual tonsil	B016.
Read	Malignant neoplasm of aryepiglottic fold hypopharyngeal aspect	B082.
Read	Malignant neoplasm of lip unspecified buccal aspect	B0040
Read	Seminoma of undescended testis	B4702
Read	Follicular lymphoma grade 3a	B6283
Read	Malignant neoplasm of tympanic cavity	B2011
Read	Malignant neoplasm of pineal gland	B543.
Read	Malignant neoplasm of penis and other male genital organ NOS	B48z.
Read	Malignant neoplasm of extraocular muscle of orbit	B5011
Read	Malignant neoplasm of ethmoid sinus	B203.
Read	Malignant neoplasm of other sites of gum	B03y.
Read	Malignant neoplasm of splenic flexure of colon	B137.
Read	Malignant neoplasm of other and ill-defined sites	B55..
Read	Malignant neoplasm of anterior epiglottis	B064.
Read	Kaposis sarcoma of multiple organs	B592X
Read	[X]Malignant neoplasm of overlapping lesion of respiratory and intrathoracic organs	Byu23
Read	Unspecified malignant neoplasm of lymphoid and histiocytic tissue of lymph nodes of multiple sites	B62z8
Read	Malignant neoplasm of connective and soft tissue of thumb	B3115
Read	Secondary and unspecified malignant neoplasm of intrathoracic lymph nodes	B561.
Read	Anaplastic large cell lymphoma ALK-negative	B62E2
Read	Chronic leukaemia	Xa9AO
Read	Malignant neoplasm of floor of mouth NOS	B04z.
Read	Malignant neoplasm of connective and soft tissue of inguinal region	B3151
Read	Malignant neoplasm of nipple of female breast	B3400
Read	Mycosis fungoides of lymph nodes of inguinal region and lower limb	B6215
Read	Unspecified malignant neoplasm of lymphoid and histiocytic tissue of intrapelvic lymph nodes	B62z6
Read	Malignant neoplasm of phalanges of hand	B305D
Read	Malignant neoplasm of tongue junctional zone	B015.
Read	Malignant neoplasm of anterior 2/3 of tongue unspecified	B014.
Read	[X]Malignant neoplasm of other specified female genital organs	Byu71
Read	Malignant neoplasm of roof of mouth	B0551
Read	Malignant neoplasm of connective and soft tissue of popliteal space	B3122
Read	Malignant neoplasm of connective and soft tissues of lumbar spine	B3141
Read	Malignant melanoma stage IB	XacIV
Read	Malignant neoplasm of lip unspecified mucosa	B0042
Read	Malignant neoplasm of lower lip oral aspect	B0033
Read	Malignant neoplasm of lower lip frenulum	B0031
Read	Malignant neoplasm of omentum	B18y3
Read	Malignant neoplasm of upper lip frenulum	B0021
Read	Sezarys disease of lymph nodes of multiple sites	B6228
Read	[X]Secondary malignant neoplasm of other and unspecified digestive organs	ByuC4

Read	Malignant neoplasm of turbinate	B300B
Read	[X]Malignant neoplasm of urinary organ unspecified	Byu90
Read	Leukaemic reticuloendotheliosis	B624.
Read	Malignant neoplasm of cerebral arachnoid mater	B5211
Read	Malignant neoplasm of urinary bladder	B49..
Read	Malignant neoplasm of temporal lobe	B512.
Read	Malignant neoplasm of pharynx unspecified	B0z0.
Read	Malignant melanoma of neck	B3241
Read	Malignant neoplasm of conjunctiva	B503.
Read	Mesothelioma	B226.
Read	Malignant neoplasm of upper third of oesophagus	B103.
Read	Malignant neoplasm of ventral tongue surface NOS	B013z
Read	Malignant neoplasm overlapping lesion of connective and soft tissue	B317.
Read	Leukaemic reticuloendotheliosis of intra-abdominal lymph nodes	B6243
Read	Malignant neoplasm of patella	B3080
Read	Malignant neoplasm of pancreatic duct	B173.
Read	Malignant neoplasm of intermediate cuneiform	B3084
Read	Lymphoblastic (diffuse) lymphoma	B62F2
Read	Malignant neoplasm of sclera	B5003
Read	Secondary and unspecified malignant neoplasm of anterior cervical lymph nodes	B5608
Read	Somatostatinoma of pancreas	XaJgM
Read	T-zone lymphoma	Xa0Ti
Read	Malignant neoplasm of coccygeal vertebra	B3064
Read	Malignant melanoma of skin	B32..
Read	Malignant neoplasm of sphenoidal sinus	B205.
Read	Malignant neoplasm of nasolacrimal duct	B5071
Read	Hodgkins paragranuloma of intrapelvic lymph nodes	B6106
Read	Malignant neoplasm of endocervix NOS	B410z
Read	Malignant neoplasm of posterior pharynx	B083.
Read	Malignant neoplasm of retromolar area	B056.
Read	Malignant neoplasm of overlapping lesion of bronchus and lung	B225.
Read	Malignant neoplasm of lower third of oesophagus	B105.
Read	Malignant neoplasm of other and ill defined site NOS	B55z.
Read	IgA monoclonal gammopathy of uncertain significance	Xa36d
Read	Malignant neoplasm overlapping lesion of male genital organs	B48y2
Read	Malignant neoplasm of cuneiform cartilage	B2132
Read	Malignant neoplasm of sublingual gland	B022.
Read	Malignant neoplasm of spinal meninges NOS	B523z
Read	Malignant neoplasm of talus	B3081
Read	Malignant neoplasm of sacral vertebra	B3063
Read	Malignant neoplasm of bones of skull and face	B300.
Read	Malignant neoplasm of tonsil	B060.
Read	Malignant immunoproliferative small intestinal disease	B62x5
Read	Malignant neoplasm of accessory sinus NOS	B20z.
Read	Follicular lymphoma grade 2	XaZdF
Read	Sezarys disease of spleen	B6227
Read	Malignant neoplasm of genitourinary organ otherwise specified	B4y..
Read	Malignant neoplasm overlapping lesion of cervix uteri	B412.
Read	Malignant melanoma stage IIIC	B32F.
Read	Malignant neoplasm of caecum	B134.
Read	Malignant neoplasm of temporal bone	B3008
Read	Secondary malignant neoplasm of urethra	B5812

Read	Malignant neoplasm of lower uterine segment	B4310
Read	Malignant neoplasm of thymus heart and mediastinum	B24..
Read	Malignant neoplasm of connective and soft tissue of hip and lower limb NOS	B312z
Read	Malignant neoplasm of pleura NOS	B23z.
Read	[X]Malignant neoplasm of bone and articular cartilage unspecified	Byu33
Read	Malignant neoplasm overlapping lesion of small intestine	B124.
Read	Malignant neoplasm of aortic body or paraganglia NOS	B545z
Read	Malignant neoplasm of lateral wall of nasopharynx NOS	B072z
Read	Diffuse non-Hodgkins lymphoma undifferentiated (diffuse)	B6278
Read	Secondary malignant neoplasm of testis	B58y6
Read	Malignant neoplasm of ileum	B122.
Read	Hodgkins granuloma of intra-abdominal lymph nodes	B6113
Read	Malignant neoplasm of upper lip oral aspect	B0023
Read	Secondary and unspec malig neop pulmonary lymph nodes	B5619
Read	Malignant neoplasm of scapula and long bones of upper arm	B304.
Read	Heavy chain disease	C3333
Read	Malignant melanoma of external surface of cheek	B3230
Read	Malignant neoplasm of endocervical canal	B4100
Read	Malignant neoplasm of upper lobe bronchus or lung NOS	B222z
Read	Malignant neoplasm of pituitary gland or craniopharyngeal duct NOS	B542z
Read	Mediastinal (thymic) large B-cell lymphoma	B627G
Read	Malignant neoplasm of epiglottis free border	B0640
Read	Secondary and unspecified malignant neoplasm of internal mammary lymph nodes	B5610
Read	Secondary malignant neoplasm of liver	B153.
Read	Megakaryocytic leukaemia	B672.
Read	[X]Secondary malignant neoplasm of other specified sites	ByuC7
Read	Diffuse non-Hodgkins lymphoma unspecified	B627X
Read	Secondary and unspecified malignant neoplasm of superficial cervical lymph nodes	B5602
Read	Malignant neoplasm of anterior 2/3 of tongue ventral surface	B0130
Read	Malignant neoplasm of descended testis	B471.
Read	Malignant neoplasm of posterior mediastinum	B243.
Read	Malignant neoplasm of ectopic testis	B4700
Read	[X]Malignant neoplasm of uterine adnexa unspecified	Byu70
Read	B-cell chronic lymphocytic leukaemia variant	Xa0QQ
Read	Malignant neoplasm of craniopharyngeal duct	B5421
Read	Malignant neoplasm of acoustic nerve	B5202
Read	Malignant neoplasm of connective and soft tissue of pelvis NOS	B315z
Read	Secondary and unspecified malignant neoplasm of deep cervical lymph nodes	B5609
Read	Malignant melanoma of eye	XM0ps
Read	Malignant neoplasm of lateral wall of oropharynx	B066.
Read	Malignant neoplasm of midline of tongue	B0111
Read	[X]Unspecified B-cell non-Hodgkins lymphoma	ByuDE
Read	Secondary malignant neoplasm of mediastinum	B571.
Read	Myeloproliferative disorder	B6y0.
Read	[X]Malignant neoplasm of overlapping lesion of female genital organs	Byu72
Read	Malignant neoplasm of hilus of lung	B2211
Read	Unspecified malignant neoplasm of lymphoid and histiocytic tissue of spleen	B62z7
Read	Malignant neoplasm of rectosigmoid junction	B140.
Read	Malignant neoplasm of other site of uterine adnexa	B44y.
Read	Myeloid leukaemia NOS	B65z.
Read	Acute monoblastic leukaemia	Xa0Sl
Read	Hodgkins disease NOS of intrapelvic lymph nodes	B61z6

Read	T-cell prolymphocytic leukaemia	B64y4
Read	Secondary malignant neoplasm of rectum	B5751
Read	Malignant neoplasm of other and unspecified sites	B5...
Read	Malignant neoplasm of other urinary organs	B4Ay.
Read	Multiple endocrine neoplasia syndrome type 1	C184.
Read	Malignant melanoma of axilla	B3250
Read	Aleukaemic lymphoid leukaemia	B64y0
Read	[X]Other myeloid leukaemia	ByuD6
Read	Malignant neoplasm of intrathoracic site NOS	B5512
Read	Malignant neoplasm of mesentery	B18y7
Read	Waldenstroms macroglobulinaemia	C3330
Read	Secondary and unspecified malignant neoplasm of intra-abdominal lymph nodes NOS	B562z
Read	[X]Malignant neoplasm of lymphoid haematopoietic and related tissue unspecified	ByuDB
Read	Malignant neoplasm of nasal cavities middle ear and accessory sinuses	B20..
Read	Malignant neoplasm of cerebral cortex	B5101
Read	Malignant neoplasm of bladder neck	B495.
Read	Malignant neoplasm of spinal arachnoid mater	B5231
Read	Malignant neoplasm of other specified part of nervous system	B52y.
Read	Subacute monocytic leukaemia	B662.
Read	Secondary malignant neoplasm of lung	B570.
Read	Malignant neoplasm of third metatarsal bone	B308A
Read	Malignant neoplasm of bone connective tissue skin and breast NOS	B3z..
Read	Malignant neoplasm of bones of skull and face NOS	B300z
Read	Hodgkins disease NOS unspecified site	B61z0
Read	Malignant neoplasm of mouth NOS	B05z.
Read	Sarcoma of dendritic cells	XaYip
Read	Malignant neoplasm of upper limb NOS	B554.
Read	Secondary and unspecified malignant neoplasm of lymph nodes	B56..
Read	Malignant neoplasm of colon	B13..
Read	Malignant neoplasm of thorax NOS	B551z
Read	Malignant neoplasm of endometrium of corpus uteri	B4302
Read	Malignant neoplasm of fourth metacarpal bone	B305B
Read	Malignant neoplasm of palatopharyngeal arch	B0623
Read	Malignant neoplasm of cerebral ventricle NOS	B515z
Read	Clinical stage C chronic lymphocytic leukaemia	B6413
Read	Hodgkins granuloma NOS	B611z
Read	Malignant neoplasm of junctional region of epiglottis	B065.
Read	Malignant melanoma of lower limb or hip NOS	B327z
Read	Hodgkins disease lymphocytic-histiocytic predominance of lymph nodes of axilla and upper limb	B6134
Read	Hodgkins disease nodular sclerosis of lymph nodes of head face and neck	B6141
Read	Malignant neoplasm of lower labial sulcus	B0513
Read	Secondary malignant neoplasm of pleura	B572.
Read	Malignant neoplasm of palate NOS	B055z
Read	Malignant neoplasm of spinal dura mater	B5230
Read	Other malignant neoplasm of lymphoid and histiocytic tissue	B62..
Read	Malignant neoplasm of fifth metacarpal bone	B305C
Read	Malignant neoplasm tonsil NOS	B060z
Read	Mesothelioma of pericardium	B2414
Read	Malignant neoplasm of connective and soft tissue of axilla	B3130
Read	Cutaneous follicle centre lymphoma	XaYin
Read	Malignant neoplasm of tibia	B3072
Read	Malignant neoplasm of rib	B3030

Read	Malignant neoplasm of cervical vertebra	B3020
Read	Malignant neoplasm of pleura	B23..
Read	Mesothelioma	X77ou
Read	Light chain myeloma	Xa0SL
Read	Malignant neoplasm of male breast	B35..
Read	Malignant neoplasm overlapping lesion of bone and articular cartilage of limbs	B309.
Read	Malignant neoplasm of lacrimal sac	B5070
Read	Malignant neoplasm of gallbladder and extrahepatic bile ducts	B16..
Read	Siewert type III adenocarcinoma	XaOrB
Read	Malignant neoplasm of other sites of lip oral cavity and pharynx	B0zy.
Read	Secondary and unspecified malignant neoplasm of obturator lymph nodes	B5654
Read	Malignant neoplasm of colon NOS	B13z.
Read	Secondary malignant neoplasm of small intestine and duodenum	B574.
Read	Malignant neoplasm of fallopian tube	B441.
Read	Malignant neoplasm of female genital organ NOS	B45z.
Read	Monoclonal gammopathy of uncertain significance	C3310
Read	5Q minus syndrome	B9379
Read	Malignant neoplasm of lacrimal gland	B502.
Read	Malignant melanoma of ear and external auricular canal	B322.
Read	Malignant neoplasm of connective and soft tissue of head face and neck	B310.
Read	Malignant neoplasm of areola of female breast	B3401
Read	Blastic NK-cell lymphoma	B62E8
Read	Extranodal NK/T-cell lymphoma nasal type	B62E4
Read	Leukaemic reticuloendotheliosis of lymph nodes of inguinal region and lower limb	B6245
Read	Secondary malignant neoplasm of uterus	B58y1
Read	Malignant lymphoma otherwise specified	B62x.
Read	Malignant neoplasm of parotid gland	B020.
Read	Malignant melanoma of chin	B3231
Read	Diffuse follicle centre lymphoma	XaYi5
Read	Malignant neoplasm of frenulum linguae	B0131
Read	Cerebral metastasis	B5832
Read	Angioimmunoblastic T-cell lymphoma	B62E9
Read	Malignant neoplasm overlapping lesion of digestive system	B1z2.
Read	Malignant neoplasm of connective and soft tissue of hand	B3113
Read	B-cell acute lymphoblastic leukaemia	Xa0SD
Read	Malignant lymphoma NOS	B62y.
Read	Myelofibrosis	D41y1
Read	Malignant neoplasm of pons	B5173
Read	Malignant neoplasm of lip unspecified inner aspect	B004.
Read	Teratoma of descended testis	XaELL
Read	Malignant neoplasm of other site of male breast	B35z.
Read	Malignant neoplasm of chest wall NOS	B5511
Read	Malignant neoplasm of other specified mouth parts	B05y.
Read	Malignant neoplasm of upper lip inner aspect	B002.
Read	Reticulosarcoma of lymph nodes of multiple sites	B6008
Read	Secondary and unspecified malignant neoplasm of inferior epigastric lymph nodes	B5651
Read	Secondary malignant neoplasm of breast	B58y0
Read	Local recurrence of malignant tumour of breast	XaFr8
Read	Aleukaemic myeloid leukaemia	B65y0
Read	Malignant neoplasm of squamocolumnar junction of cervix	B41y1
Read	Malignant neoplasm of areola of male breast	B3501
Read	Malignant neoplasm of medulla oblongata	B5171

Read	Hodgkins disease NOS of lymph nodes of axilla and upper limb	B61z4
Read	Malignant melanoma of buttock	B3252
Read	Malignant neoplasm of connective and soft tissue of hip	B3120
Read	[X]Other specified leukaemias	ByuD8
Read	Aleukaemic monocytic leukaemia	B66y0
Read	Malignant melanoma stage IV M1b	B32H.
Read	Secondary malignant neoplasm of respiratory and digestive systems	B57..
Read	Malignant neoplasm of hypopharynx NOS	B08z.
Read	Malignant neoplasm of other male genital organ	B48y.
Read	Subacute leukaemia	Xa9AN
Read	Malignant neoplasm of spinal pia mater	B5232
Read	Malignant neoplasm of peripheral nerves of head face and neck	B5240
Read	Mast cell malignancy of intrathoracic lymph nodes	B6262
Read	Malignant neoplasm of lip unspecified frenulum	B0041
Read	Cutaneous follicle centre lymphoma	B6286
Read	Hepatoblastoma of liver	B1501
Read	Secondary malignant neoplasm of retroperitoneum and peritoneum	B576.
Read	[X]Malignant neoplasm of male genital organs	Byu8.
Read	[X]Secondary and unspecified malignant neoplasm lymph nodes of multiple regions	ByuC2
Read	Lymphosarcoma of intrapelvic lymph nodes	B6016
Read	Follicular lymphoma grade 3b	XaYim
Read	Malignant neoplasm of bone connective tissue skin and breast otherwise specified	B3y..
Read	Malignant melanoma of thigh	B3271
Read	Acute leukaemia NOS	B680.
Read	Malignant neoplasm of greater vestibular (Bartholins) gland	B4510
Read	Malignant melanoma of lower leg	B3274
Read	Subacute myeloid leukaemia	B652.
Read	Secondary and unspecified malignant neoplasm of common iliac lymph nodes	B5623
Read	Lymphosarcoma of spleen	B6017
Read	Malignant neoplasm of glossoepiglottic fold	B0641
Read	Kaposis sarcoma of soft tissue	B31z0
Read	[X]Malignant neoplasm of overlapping lesion of other and ill-defined sites	ByuC1
Read	[X]Diffuse non-Hodgkins lymphoma unspecified	ByuDC
Read	Malignant neoplasm of upper respiratory tract part unspecified	B2z0.
Read	Malignant lymphoma NOS of intra-abdominal lymph nodes	B62y3
Read	[X]Malignant neoplasm of ill-defined sites within the respiratory system	Byu24
Read	Follicular non-Hodgkins large cell lymphoma	B6272
Read	Malignant neoplasm of lower lip mucosa	B0032
Read	Malignant neoplasm of digestive tract and peritoneum NOS	B1zz.
Read	Malignant neoplasm of connective and soft tissue site NOS	B31z.
Read	Malignant neoplasm of heart thymus and mediastinum NOS	B24z.
Read	Mantle cell lymphoma	B62F1
Read	Nodular lymphoma of spleen	B6207
Read	Hodgkins lymphocytic depletion of unspecified site	B6160
Read	Malignant neoplasm of lip vermilion border NOS	B00zz
Read	Leukaemic reticuloendotheliosis of lymph nodes of axilla and upper limb	B6244
Read	Secondary and unspecified malignant neoplasm of deep inguinal lymph nodes	B5641
Read	Malignant melanoma stage IIIC	XacIb
Read	Secondary malignant neoplasm of large intestine and rectum	XaXQk
Read	Malignant neoplasm of intrahepatic biliary passages	B1512
Read	Malignant neoplasm of diaphragm	B3131
Read	Malignant neoplasm of maxilla	B300A

Read	[X]Malignant neoplasm of other and unspecified cranial nerves	ByuA0
Read	Secondary malignant neoplasm of brain or spinal cord NOS	B583z
Read	Secondary and unspecified malignant neoplasm of axilla and upper limb lymph nodes NOS	B563z
Read	Secondary malignant neoplasm of unknown site	XM0pv
Read	Malignant neoplasm of ectopic site of female breast	B34y0
Read	[X]Non-Hodgkins lymphoma unspecified type	XE2t9
Read	Angiosarcoma of spleen	B1z10
Read	Malignant neoplasm of female breast NOS	B34z.
Read	Unspecified malignant neoplasm of lymphoid and histiocytic tissue of unspecified site	B62z0
Read	Chronic erythraemia	B671.
Read	Malignant neoplasm of mesocaecum	B18y1
Read	Unspecified malignant neoplasm of lymphoid and histiocytic tissue of lymph nodes of inguinal region and lower limb	B62z5
Read	Malignant neoplasm of palatoglossal arch	B0622
Read	Hodgkins sarcoma of lymph nodes of multiple sites	B6128
Read	Myeloid leukaemia	B65..
Read	Siewert type II adenocarcinoma	B118.
Read	Lymphoproliferative disorder	Xa0QI
Read	Malignant neoplasm of other part of brain NOS	B51yz
Read	Malignant melanoma of other specified skin site	B32y.
Read	Malignant melanoma stage IIIB	B32E.
Read	Malignant neoplasm of anterior wall of nasopharynx	B073.
Read	Malignant melanoma of great toe	B3279
Read	Small cell B-cell lymphoma	B62F0
Read	Malignant neoplasm of cricoid cartilage	B2131
Read	Malignant neoplasm of other specified site of nasopharynx	B07y.
Read	Lymphosarcoma of lymph nodes of axilla and upper limb	B6014
Read	Pathological fracture due to metastatic bone disease	B5850
Read	Malignant neoplasm of adrenal gland NOS	B540z
Read	Malignant neoplasm of orbital bone	B3005
Read	Hodgkins paragranuloma of unspecified site	B6100
Read	Secondary and unspecified malignant neoplasm of coeliac lymph nodes	B5620
Read	Malignant neoplasm of lower-outer quadrant of female breast	B345.
Read	Prolymphocytic leukaemia	B64y1
Read	Malignant neoplasm of temporal lobe NOS	B512z
Read	Malignant neoplasm of upper labial sulcus	B0512
Read	Malignant neoplasm of vaginal vault	B4501
Read	Malignant neoplasm of main bronchus	B221.
Read	Reticulosarcoma of lymph nodes of head face and neck	B6001
Read	Malignant neoplasm of other and ill-defined sites within the respiratory and intrathoracic organs	B2z..
Read	Refractory anaemia with ringed sideroblasts	Xa0Se
Read	Follicular lymphoma grade 2	B6281
Read	Malignant neoplasm of thalamus	B5105
Read	[X]Malignant neoplasm of meninges unspecified	ByuA2
Read	Malignant neoplasm of jejunum	B121.
Read	Malignant melanoma stage IV M1b	XacIe
Read	Monoclonal paraproteinaemia (& gammopathy)	C331.
Read	Malignant neoplasm of mesentery	XaEY9
Read	Malignant neoplasm overlapping lesion of floor of mouth	B042.
Read	Burkitts lymphoma of lymph nodes of head face and neck	B6021
Read	Malignant neoplasm of presacral region	B5531
Read	Malignant neoplasm of auditory tube middle ear and mastoid air cells NOS	B201z
Read	Malignant neoplasm of lesser curve of stomach unspecified	B115.

Read	Malignant melanoma stage IV M1c	B32J.
Read	Secondary and unspecified malignant neoplasm of inguinal and lower limb lymph nodes	B564.
Read	Malignant neoplasm of head neck and face NOS	B550z
Read	Secondary malignant neoplasm of large intestine or rectum NOS	B575z
Read	Malignant neoplasm of prepuce (foreskin)	B480.
Read	Malignant neoplasm of submandibular gland	B021.
Read	Secondary malignant neoplasm of small intestine or duodenum NOS	B574z
Read	Mast cell malignancy of lymph nodes of axilla and upper limb	B6264
Read	Secondary and unspecified malignant neoplasm of superficial inguinal lymph nodes	B5640
Read	Malignant neoplasm of cerebellum	B516.
Read	Malignant melanoma of upper limb and shoulder	B326.
Read	[X]Malignant neoplasm overlapping lesion of brain and other part of central nervous system	ByuA3
Read	Local recurrence of malignant tumour of urinary bladder	XaFrL
Read	Malignant neoplasm of brain stem NOS	B517z
Read	Malignant neoplasm of zygomatic bone	B3009
Read	Malignant neoplasm of lateral wall of urinary bladder	B492.
Read	Hodgkins disease mixed cellularity of intrathoracic lymph nodes	B6152
Read	Malignant neoplasm of cervix uteri NOS	B41z.
Read	Lymphosarcoma NOS	B601z
Read	IgD monoclonal gammopathy of uncertain significance	Xa36k
Read	[X]Other lymphoid leukaemia	ByuD5
Read	Malignant neoplasm of vagina NOS	B450z
Read	[X]Malignant neoplasms of lymphoid haematopoietic and related tissue	ByuD.
Read	Malignant neoplasm of peripheral nerve of abdomen	B5244
Read	Burkitts lymphoma of lymph nodes of inguinal region and lower limb	B6025
Read	Malignant neoplasm of posterior wall of nasopharynx	B071.
Read	Malignant neoplasm of posterior wall of urinary bladder	B494.
Read	Malignant neoplasm lymphatic or haematopoietic tissue NOS	B6z..
Read	Malignant neoplasm of lower lip inner aspect	B003.
Read	Malignant neoplasm of midbrain	B5172
Read	Plasmacytoma - disorder	Xa9AA
Read	Aleukaemic leukaemia	Xa9AP
Read	Malignant neoplasm of testis	B47..
Read	Malignant melanoma stage IV M1a	XacId
Read	Other types of follicular non-Hodgkins lymphoma	B627B
Read	Malignant neoplasm of bones and articular cartilage of limb unspecified	B30X.
Read	Secondary malignant neoplasm of other urinary organ NOS	B581z
Read	Reticulosarcoma NOS	B600z
Read	Letterer-Siwe disease of lymph nodes of inguinal region and lower limb	B6255
Read	Sezarys disease of lymph nodes of inguinal region and lower limb	B6225
Read	Malignant neoplasm of ectopic site of male breast	B35z0
Read	Secondary malignant neoplasm of other specified site NOS	B58z.
Read	Malignant neoplasm of vomer	B300C
Read	Malignant neoplasm of fourth metatarsal bone	B308B
Read	Bowel scope (flexible sigmoidoscopy) screen: cancer detected	68W24
Read	Malignant neoplasm of adrenal gland	B540.
Read	Malignant neoplasm of ribs sternum and clavicle	B303.
Read	[X]Mesothelioma of other sites	XE1wg
Read	Atypical chronic myeloid leukaemia BCR/ABL negative	B6513
Read	Malignant sacral teratoma	XaKWz
Read	[X]Malignant neoplasm of peripheral nerves of trunk unspecified	Byu54
Read	Chronic lymphocytic prolymphocytic leukaemia syndrome	Xa0Rn

Read	Kaposis sarcoma of lymph nodes	B6z0.
Read	Malignant neoplasm of fibula	B3071
Read	Malignant neoplasm of opening of auditory tube	B0721
Read	Teratoma of descended testis	B4711
Read	Malignant white blood cell disorder	Xa0Ri
Read	Malignant neoplasm - pluriglandular involvement unspecified	B54X.
Read	Malignant neoplasm of tonsillar fossa NOS	B062z
Read	Mycosis fungoides of intrapelvic lymph nodes	B6216
Read	Leukaemia of unspecified cell type	B68..
Read	Hodgkins disease mixed cellularity of lymph nodes of axilla and upper limb	B6154
Read	Malignant neoplasm overlapping lesion of hypopharynx	B084.
Read	Malignant neoplasm of unspecified site NOS	B59z.
Read	Malignant neoplasm of stomach NOS	B11z.
Read	Malignant neoplasm of anterior wall of urinary bladder	B493.
Read	[X]Kaposis sarcoma of other sites	Byu5B
Read	Malignant neoplasm overlapping lesion of brain	XaC1Q
Read	Malignant histiocytosis NOS	B623z
Read	Malignant neoplasm of cheek NOS	B5501
Read	Chronic myeloid leukaemia NOS	B651z
Read	Malignant neoplasm of cartilage of nose	B2000
Read	Null cell acute lymphoblastic leukaemia	Xa0SG
Read	Malignant neoplasm of heart NOS	B241z
Read	Malignant neoplasm of pituitary gland	B5420
Read	Somatostatinoma of pancreas	B176.
Read	Malignant neoplasm of cervical oesophagus	B100.
Read	Malignant neoplasm of urinary bladder NOS	B49z.
Read	Follicular lymphoma grade 1	XaYij
Read	Malignant neoplasm of digestive organs and peritoneum	XE1vP
Read	Malignant neoplasm of visceral pleura	B231.
Read	[X]Malignant neoplasms of independent (primary) multiple sites	ByuE0
Read	[X]Malignant neoplasm of digestive organs	Byu1.
Read	Local recurrence of malignant tumour of urinary bladder	B498.
Read	(Myelodysplastic syndrome unspecified) or (myelodysplasia)	B937W
Read	Malignant neoplasm of uterus part unspecified	B40..
Read	Malignant mastocytosis	XaBAk
Read	Malignant reticulosis	B62x4
Read	Malignant neoplasm of auditory (Eustachian) tube	B2010
Read	Malignant neoplasm of lacrimal duct	B507.
Read	Letterer-Siwe disease	B625.
Read	Malignant neoplasm of laryngeal cartilage NOS	B213z
Read	[X]Secondary malignant neoplasm of other and unspecified respiratory organs	ByuC3
Read	Secondary malignant neoplasm of epididymis and vas deferens	B58y8
Read	Malignant neoplasm of specified site of pancreas NOS	B17yz
Read	Acute megakaryoblastic leukaemia	Xa0Sm
Read	Acute myelofibrosis	B675.
Read	Hodgkins sarcoma	B612.
Read	Multiple endocrine neoplasia syndrome type 1	X40OZ
Read	Acute erythraemia and erythroleukaemia	XE2vi
Read	Secondary and unspecified malignant neoplasm of facial lymph nodes	B5606
Read	Malignant neoplasm of brain stem	B517.
Read	Seminoma of undescended testis	XaELJ
Read	Secondary malignant neoplasm of other respiratory organs	B573.

Read	Disseminated malignancy NOS	XE1vn
Read	Hodgkins disease lymphocytic depletion of lymph nodes of axilla and upper limb	B6164
Read	Malignant neoplasm of connective and soft tissue of foot	B3124
Read	Malignant neoplasm of dome of urinary bladder	B491.
Read	Erythroleukaemia	XaBAl
Read	Myeloid sarcoma NOS	B653z
Read	Multiple myeloma	B630.
Read	Benign paraproteinaemia	XaIt4
Read	Reticulosarcoma of intrathoracic lymph nodes	B6002
Read	Malignant neoplasm of glomus jugulare	B5450
Read	Hodgkins disease nodular sclerosis of unspecified site	B6140
Read	Malignant neoplasm of overlapping lesion of female genital organs	B45X.
Read	Malignant neoplasm of pharyngeal tonsil	B0711
Read	Subcutaneous panniculitic T-cell lymphoma	B62E7
Read	Secondary malignant neoplasm of tongue	XaC0y
Read	Malignant neoplasm of nasopharyngeal soft palate surface	B0731
Read	Acute myeloid leukaemia with multilineage dysplasia	B6501
Read	Chronic myelogenous leukaemia BCR/ABL positive	XaYj0
Read	Mycosis fungoides of unspecified site	B6210
Read	[X]Malignant neoplasm of mediastinum part unspecified	Byu25
Read	Malignant neoplasm of navicular	B3087
Read	Clinical stage C chronic lymphocytic leukaemia	Xaa1P
Read	Malignant neoplasm of dorsum of tongue NOS	B011z
Read	Kaposis sarcoma of palate	B05z0
Read	Malignant neoplasm of upper lip buccal aspect	B0020
Read	Malignant neoplasm of radius	B3043
Read	Malignant neoplasm of lower lip external	B0010
Read	Hodgkins disease mixed cellularity of spleen	B6157
Read	Malignant neoplasm of pelvis sacrum or coccyx NOS	B306z
Read	Malignant neoplasm of anterior mediastinum	B242.
Read	Malignant neoplasm of basal ganglia	B5100
Read	Unspecified B-cell non-Hodgkins lymphoma	B627W
Read	Malignant neoplasm of hand bones NOS	B305z
Read	Secondary malignant neoplasm of retroperitoneum or peritoneum NOS	B576z
Read	[X]Malignant neoplasm without specification of site	ByuC8
Read	Hodgkins disease lymphocytic-histiocytic predominance of intrathoracic lymph nodes	B6132
Read	Burkitts lymphoma of spleen	B6027
Read	Monocytic leukaemia	B66..
Read	Malignant neoplasm of oesophagus NOS	XE1vQ
Read	Malignant neoplasm of upper gum	B030.
Read	Malignant neoplasm of cystic duct	B1610
Read	Malignant neoplasm of aortic body and other paraganglia	B545.
Read	Malignant neoplasm of other and unspecified parts of nervous system	B52..
Read	Acute panmyelosis	B674.
Read	Sezarys disease of intrathoracic lymph nodes	B6222
Read	Malignant neoplasm of optic nerve	B5201
Read	Malignant neoplasm of other and unspecified parts of mouth	B05..
Read	Malignant neoplasm of endocervix	B410.
Read	Other specified leukaemia NOS	B67z.
Read	Malignant neoplasm of adrenal medulla	B5401
Read	Secondary and unspecified malignant neoplasm of lymph nodes in multiple sites	B56y.
Read	Malignant neoplasm of specified parts of peritoneum NOS	B18yz

Read	IgD myeloma	Xa36c
Read	Malignant neoplasm of other parts of brain	B51y.
Read	Malignant neoplasm of connective and soft tissue of toe	B3125
Read	Chronic lymphoid leukaemia	B641.
Read	Malignant neoplasm of roof of nasopharynx	B070.
Read	[X]Other monocytic leukaemia	ByuD7
Read	T/NK-cell lymphoma	B62E.
Read	Malignant neoplasm of trunk NOS	B55y1
Read	Atypical chronic myeloid leukaemia BCR/ABL negative	XaYis
Read	Malignant neoplasm of vulva unspecified	XE1vj
Read	Acute eosinophilic leukaemia	Xa0Sn
Read	Acute lymphoid leukaemia	B640.
Read	Malignant neoplasm of parietal lobe	B513.
Read	Hodgkins disease nodular sclerosis	B614.
Read	[X]Malignant neoplasm of intestinal tract part unspecified	Byu12
Read	Malignant melanoma of knee	B3272
Read	Tumour lysis syndrome	Xa0Sq
Read	Malignant neoplasm of ureteropelvic junction	B4A11
Read	Malignant neoplasm of main bronchus NOS	B221z
Read	Malignant neoplasm of intrahepatic gall duct	B1514
Read	Nodular lymphoma of intrathoracic lymph nodes	B6202
Read	Malignant neoplasm of spleen NEC	B1z1.
Read	Malignant neoplasm of floor of nasopharynx	B0730
Read	Malignant melanoma of ankle	B3275
Read	Hodgkins disease lymphocytic-histiocytic predominance of lymph nodes of inguinal region and lower limb	B6135
Read	Malignant neoplasm of bone and articular cartilage	B30..
Read	Siewert type I adenocarcinoma	XaOrV
Read	Malignant neoplasm of liver unspecified	B152.
Read	Malignant neoplasm of larynx NOS	B21z.
Read	Malignant neoplasm of globus pallidus	B5103
Read	Sezarys disease of intrapelvic lymph nodes	B6226
Read	Malignant melanoma stage IA	XacIU
Read	[X]Malignant neoplasm of overlapping lesion of bone and articular cartilage of limbs	Byu30
Read	Malignant neoplasm of ureteric orifice	B496.
Read	Malignant neoplasm of penis part unspecified	B483.
Read	Secondary malignant neoplasm of skin of shoulder and arm	B5824
Read	Malignant neoplasm of other site of urinary bladder	B49y.
Read	Malignant neoplasm of middle lobe bronchus or lung NOS	B223z
Read	Other and unspecified leukaemia	B67y.
Read	Hypernephroma	B4A00
Read	Malignant neoplasm of vallecula	B063.
Read	Malignant neoplasm of upper lobe bronchus or lung	B222.
Read	Lymphosarcoma of lymph nodes of multiple sites	B6018
Read	Malignant neoplasm of cartilage of trachea	B2200
Read	Malignant neoplasm of hand bones	XE1ve
Read	Choriocarcinoma	B420.
Read	Malignant neoplasm of clavicle	B3032
Read	Malignant melanoma of popliteal fossa area	B3273
Read	Overlapping lesion of other and unspecified parts of mouth	B057.
Read	Malignant neoplasm of upper lip lipstick area	B0001
Read	Acute promyelocytic leukaemia - hypogranular variant	Xa3Ee
Read	Malignant neoplasm of other site of female breast NOS	B34yz

Read	Malignant neoplasm of ilium	B3060
Read	Monoclonal paraproteinaemia	XE11b
Read	Malignant neoplasm of major salivary glands	B02..
Read	Malignant melanoma of lip	B320.
Read	Sezarys disease	B622.
Read	Malignant neoplasm of other and unspecified sites	XE2vR
Read	T-cell prolymphocytic leukaemia	Xa0S9
Read	Hodgkins lymphocytic-histiocytic predominance of spleen	B6137
Read	Secondary malignant neoplasm of adrenal gland	B587.
Read	Secondary malignant neoplasm of brain and spinal cord	B583.
Read	Reticulosarcoma of spleen	B6007
Read	Malignant neoplasm of retrocaecal tissue	B1802
Read	Hodgkins disease lymphocytic depletion of intrapelvic lymph nodes	B6166
Read	Hodgkins granuloma of lymph nodes of multiple sites	B6118
Read	Malignant neoplasm of other site of heart thymus and mediastinum	B24y.
Read	Malignant neoplasm of faucial pillar	B0620
Read	Subacute myelomonocytic leukaemia	B692.
Read	Malignant neoplasm of upper lip vermilion border NOS	B000z
Read	Multifocal and unisystemic Langerhans-cell histiocytosis	B62B.
Read	Histiocytic sarcoma	B62D.
Read	Osteosarcoma	B30z0
Read	Malignant neoplasm of costo-vertebral joint	B3034
Read	Essential thrombocythaemia	X20FX
Read	Malignant neoplasm of intrahepatic bile ducts NOS	B151z
Read	Malignant neoplasm of myocardium	B2412
Read	Malignant neoplasm of lower lip vermilion border NOS	B001z
Read	Hodgkins disease NOS of intrathoracic lymph nodes	B61z2
Read	Malignant melanoma of lower limb and hip	B327.
Read	Malignant neoplasm of pancreas NOS	B17z.
Read	Malignant neoplasm of head NOS	B5500
Read	Malignant neoplasm of cuboid	B3086
Read	Hodgkins paragranuloma of lymph nodes of inguinal region and lower limb	B6105
Read	Diffuse non-Hodgkins large cell lymphoma	B627A
Read	Malignant neoplasm of lymphatic and haemopoietic tissue	B6...
Read	Malignant neoplasm overlapping lesion of nasopharynx	B074.
Read	Acute myeloblastic leukaemia	Xa0Sk
Read	Malignant neoplasm of paraurethral glands	B4A4.
Read	Malignant melanoma of other and unspecified parts of face	B323.
Read	Malignant neoplasm of tongue tip and lateral border	B012.
Read	Malignant neoplasm of eyeball NOS	B500z
Read	Malignant neoplasm of digestive organs and peritoneum	B1...
Read	Malignant neoplasm of cerebral ventricles	B515.
Read	Immunoproliferative neoplasm or myeloma NOS	B63z.
Read	Secondary and unspecified malignant neoplasm of sacral lymph nodes	B5653
Read	Hodgkins disease NOS of lymph nodes of inguinal region and lower limb	B61z5
Read	Hodgkins sarcoma of lymph nodes of axilla and upper limb	B6124
Read	Malignant neoplasm of connective and soft tissue of other specified site	B31y.
Read	Plasmacytoma NOS	B6302
Read	Malignant neoplasms of lymphoid and histiocytic tissue NOS	B62z.
Read	Nodular lymphoma of lymph nodes of inguinal region and lower limb	B6205
Read	Malignant melanoma of finger	B3264
Read	Malignant neoplasm of parathyroid gland	B541.

Read	[X]Malignant neoplasm of other specified male genital organs	Byu80
Read	Malignant neoplasm of Waldeyers ring	B0z1.
Read	Monoclonal gammopathy of uncertain significance	Xa0SJ
Read	Malignant neoplasm of short bones of leg NOS	B308z
Read	True histiocytic lymphoma	Xa0Ty
Read	[X]Mesothelioma unspecified	Byu51
Read	Malignant neoplasm of connective and soft tissue of sacrum or coccyx	B3153
Read	Sezarys disease of lymph nodes of axilla and upper limb	B6224
Read	Malignant neoplasm of base of tongue	B010.
Read	Malignant neoplasm of undescended testis NOS	B470z
Read	Primary carcinoma of liver	B1500
Read	Malignant neoplasm of brain	B51..
Read	Mycosis fungoides of lymph nodes of multiple sites	B6218
Read	Malignant neoplasm of intrahepatic bile ducts	B151.
Read	IgA myeloma	Xa36a
Read	Malignant neoplasm of testis NOS	XE1vk
Read	Malignant neoplasm of thyroid cartilage	B2133
Read	Malignant neoplasm of cornea	B504.
Read	[X]Malignant neoplasm of other specified sites	ByuC0
Read	Hodgkins disease lymphocytic-histiocytic predominance of lymph nodes of multiple sites	B6138
Read	Malignant neoplasm of laryngeal cartilage	B213.
Read	Other paraproteinaemias	C332.
Read	Malignant neoplasm of round ligament	B444.
Read	Reticulosarcoma of lymph nodes of inguinal region and lower limb	B6005
Read	Malignant neoplasm of carpal bone - trapezium	B3054
Read	Mesothelioma of peritoneum	B181.
Read	Hereditary nonpolyposis colon cancer	XaFsw
Read	Lymphosarcoma of lymph nodes of head face and neck	B6011
Read	Hypergranular promyelocytic leukaemia	Xa0eB
Read	Malignant neoplasm of other endocrine glands and related structures	B54..
Read	Malignant neoplasm of gallbladder and extrahepatic bile ducts NOS	B16z.
Read	Malignant neoplasm of junction of hard and soft palate	B0550
Read	Malignant neoplasm of myometrium of corpus uteri	B4303
Read	Hodgkins disease lymphocytic depletion of intrathoracic lymph nodes	B6162
Read	[X]Malignant neoplasms of independent (primary) multiple sites	ByuE.
Read	Hairy cell leukaemia variant	Xa0SA
Read	Secondary malignant neoplasm of vagina	B58y3
Read	Leukaemic reticuloendotheliosis NOS	B624z
Read	[X]Other specified carcinomas of liver	Byu11
Read	Mesothelioma of pleura	B232.
Read	Malignant neoplasm of tonsillar fossa	B061.
Read	Malignant melanoma of eye	B509.
Read	Other malignant neoplasm NOS	B591.
Read	Myelodysplastic and myeloproliferative disease	XaYiK
Read	Secondary and unspecified malignant neoplasm of intercostal lymph nodes	B5611
Read	Hodgkins sarcoma of intrapelvic lymph nodes	B6126
Read	Malignant neoplasm overlapping lesion of breast	B347.
Read	Malignant neoplasm of other specified endocrine gland	B54y.
Read	Diffuse non-Hodgkins small cleaved cell (diffuse) lymphoma	B6274
Read	Subacute lymphoid leukaemia	B642.
Read	Secondary malignant neoplasm of skin of trunk	B5823
Read	Secondary malignant neoplasm of kidney	B580.

Read	Other myeloid leukaemia NOS	B65yz
Read	Malignant neoplasm of other sites of bronchus or lung	B22y.
Read	Malignant melanoma of temple	B3235
Read	Malignant neoplasm of interlobular biliary canals	B1511
Read	Malignant neoplasm of other specified site of oropharynx NOS	B06yz
Read	Malignant neoplasm of hypothalamus	B5104
Read	Malignant neoplasm of peripheral nerves and autonomic nervous system	B524.
Read	Secondary malignant neoplasm of prostate	B58y5
Read	Malignant neoplasm of orbit	B501.
Read	Malignant neoplasm of nipple or areola of female breast NOS	B340z
Read	Malignant neoplasm of tongue NOS	B01z.
Read	Macroglobulinaemia NOS	C333z
Read	Acquired haemoglobin H disease	XaB49
Read	Malignant neoplasm of upper buccal sulcus	B0510
Read	Hodgkins disease nodular sclerosis NOS	B614z
Read	Malignant neoplasm of lower lobe bronchus	B2240
Read	Acute monocytic leukaemia	B660.
Read	Malignant neoplasm of eyeball excluding conjunctiva cornea retina and choroid	B500.
Read	Clinical stage A chronic lymphocytic leukaemia	Xaa1N
Read	Diffuse follicle centre lymphoma	B6285
Read	Secondary and unspecified malignant neoplasm of submental lymph nodes	B5607
Read	Tumour lysis syndrome	C37yD
Read	Lymphoepithelioid lymphoma	Xa0Tj
Read	Hodgkins disease lymphocytic depletion of lymph nodes of multiple sites	B6168
Read	Mast cell malignancy of intra-abdominal lymph nodes	B6263
Read	Malignant neoplasm of femur	B3070
Read	Malignant neoplasm of ampulla of Vater	B162.
Read	Monosomy 7 syndrome	XaB46
Read	T-zone lymphoma	B62x0
Read	Mast cell malignancy of unspecified site	B6260
Read	Erythraemia	Xa0eC
Read	[X]Malignant neoplasm of central nervous system unspecified	ByuA1
Read	Malignant neoplasm of mastoid air cells	B2013
Read	Malignant neoplasm of coccygeal body	B5452
Read	Secondary malignant neoplasm of liver and intrahepatic bile duct	B5770
Read	Malignant neoplasm of connective and soft tissue of head face and neck	B310z
Read	Malignant neoplasm of other specified site of eye	B50y.
Read	[X]Malignant neoplasm of bronchus or lung unspecified	Byu20
Read	Malignant neoplasm of cerebral meninges	B521.
Read	Acute myeloid leukaemia with 11q23 abnormality	B6500
Read	Secondary and unspecified malignant neoplasm of submandibular lymph nodes	B5605
Read	Letterer-Siwe disease of lymph nodes of multiple sites	B6258
Read	Malignant neoplasm of other specified sites	B55y.
Read	Secondary and unspecified malignant neoplasm of pectoral lymph nodes	B5633
Read	Secondary and unspecified malignant neoplasm of inguinal and lower limb lymph nodes NOS	B564z
Read	Letterer-Siwe disease of intra-abdominal lymph nodes	B6253
Read	Follicular lymphoma grade 3a	XaYil
Read	Malignant neoplasm of occipital bone	B3004
Read	Malignant neoplasm of upper-inner quadrant of female breast	B342.
Read	Malignant melanoma of trunk (excluding scrotum)	B325.
Read	[X]Secondary malignant neoplasm of bladder and other and unspecified urinary organs	ByuC5
Read	Paraproteinaemia NOS	C332z

Read	Malignant neoplasm of vestibule of nose	B2003
Read	Hodgkins disease mixed cellularity of intra-abdominal lymph nodes	B6153
Read	Hodgkins granuloma of lymph nodes of axilla and upper limb	B6114
Read	Follicular non-Hodgkins lymphoma	B627C
Read	Lymphosarcoma and reticulosarcoma	B60..
Read	Malignant neoplasm of thyroid gland	B53..
Read	Malignant neoplasm of upper lobe of lung	B2221
Read	Malignant neoplasm of glottis	B210.
Read	Malignant neoplasm of connective and soft tissue of great toe	B3126
Read	Malignant neoplasm of vertebral column	B302.
Read	Other lymphoid leukaemia	B64y.
Read	Malignant melanoma of back	XaCIv
Read	Malignant neoplasm of body of uterus	B43..
Read	Malignant neoplasm of pelvic peritoneum	B18y5
Read	[X]Other malignant immunoproliferative diseases	ByuD4
Read	Malignant neoplasm of posterior margin of nasal septum and choanae	B0732
Read	[X]Kaposis sarcoma of multiple organs	Byu52
Read	Malignant neoplasm overlapping lesion of respiratory and intrathoracic organs	B26..
Read	Hodgkins granuloma	B611.
Read	Malignant neoplasm of nasal bone	B3003
Read	Secondary and unspecified malignant neoplasm of lymph nodes NOS	B56z.
Read	Malignant neoplasm of thymus	B240.
Read	Other mature T/NK-cell lymphoma	B62Ew
Read	Malignant neoplasm of other and ill-defined sites within the lip oral cavity and pharynx	B0z..
Read	Hodgkins disease	B61..
Read	Malignant neoplasm of tapetum	B51y1
Read	Malignant neoplasm of choroid	B506.
Read	Malignant neoplasm of appendix	B135.
Read	[X]Malignant neoplasm of overlapping lesion of heart mediastinum and pleura	Byu21
Read	Nonfollicular lymphoma	B62F.
Read	Myelodysplastic and myeloproliferative disease	B677.
Read	Lymphosarcoma of lymph nodes of inguinal region and lower limb	B6015
Read	Hodgkins disease lymphocytic-histiocytic predominance of unspecified site	B6130
Read	Malignant neoplasm of anterior wall of nasopharynx NOS	B073z
Read	Nodular lymphoma of unspecified site	B6200
Read	[X]Malignant neoplasm - pluriglandular involvement unspecified	ByuB0
Read	Malignant neoplasm of nasopharynx	B07..
Read	Secondary malignant neoplasm of ureter	B5810
Read	Letterer-Siwe disease of unspecified sites	B6250
Read	Malignant neoplasm of nipple and areola of male breast	B350.
Read	Hodgkins disease mixed cellularity of lymph nodes of inguinal region and lower limb	B6155
Read	Malignant neoplasm of isthmus of uterine body NOS	B431z
Read	Overlapping malignant melanoma of skin	B32y0
Read	Malignant melanoma of scalp and neck	B324.
Read	Secondary and unspecified malignant neoplasm of occipital lymph nodes	B5603
Read	Non-secretory myeloma	Xa0SN
Read	Hodgkins granuloma of lymph nodes of inguinal region and lower limb	B6115
Read	Malignant neoplasm of tunica vaginalis	B48y1
Read	Malignant neoplasm of meninges unspecified	B52X.
Read	Diffuse large B-cell lymphoma	B627E
Read	Malignant neoplasm of Gartners duct	B4500
Read	Adult T-cell leukaemia	B64y2

Read	Atypical chronic myeloid leukaemia	Xa0SX
Read	Malignant neoplasm of xiphoid process	B3035
Read	Malignant neoplasm of connective and soft tissue of abdomen NOS	B314z
Read	Hodgkins paragranuloma of lymph nodes of multiple sites	B6108
Read	Malignant neoplasm of pancreas	B17..
Read	Malignant neoplasm of third metacarpal bone	B305A
Read	Secondary malignant neoplasm of large intestine and rectum	B575.
Read	Malignant neoplasm of neck NOS	B5504
Read	Hodgkins disease mixed cellularity of unspecified site	B6150
Read	Malignant neoplasm of uncus	B5121
Read	Malignant neoplasm of connective and soft tissue of buttock	B3150
Read	Malignant neoplasm of upper lip inner aspect NOS	B002z
Read	Malignant neoplasm of rectum rectosigmoid junction and anus NOS	B14z.
Read	Secondary and unspecified malignant neoplasm of superficial mesenteric lymph nodes	B5621
Read	Malignant neoplasm of faucial tonsil	B0600
Read	Malignant neoplasm of base of tongue dorsal surface	B0100
Read	Malignant melanoma of groin	B3253
Read	Malignant neoplasm of hepatic flexure of colon	B130.
Read	Malignant plasma cell neoplasm extramedullary plasmacytoma	B6300
Read	Malignant neoplasm of lateral portion of floor of mouth	B041.
Read	Leukaemia - specif. cell NOS	XE20T
Read	Malignant neoplasm of head of pancreas	B170.
Read	Malignant neoplasm of retroperitoneum	B180.
Read	Acute monoblastic leukaemia	B663.
Read	[X]Myelodysplastic syndrome unspecified	ByuHD
Read	Nodular lymphoma (Brill - Symmers disease)	B620.
Read	Anaplastic large cell lymphoma ALK-positive	B62E1
Read	Other and unspecified peripheral and cutaneous T-cell lymphomas	B62xX
Read	Malignant melanoma stage IIB	XacIX
Read	Malignant neoplasm of connective and soft tissue upper arm	B3111
Read	Malignant neoplasm of overlapping lesion of tonsil	B0602
Read	Plasmacytoma NOS	XaBLx
Read	Malignant neoplasm of axillary tail of female breast	B346.
Read	Malignant neoplasm of branchial cleft	B06y0
Read	Letterer-Siwe disease of intrathoracic lymph nodes	B6252
Read	Secondary and unspecified malignant neoplasm of posterior mediastinal lymph nodes	B5614
Read	Malignant neoplasm of other and ill-defined sites within the digestive organs and peritoneum	B1z..
Read	Secondary and unspecified malignant neoplasm of anterior mediastinal lymph nodes	B5613
Read	B-cell acute lymphoblastic leukaemia	B6400
Read	Acquired renal cyst with neoplastic change	X30J9
Read	[X]Malignant neoplasm of peritoneum unspecified	Byu57
Read	Malignant neoplasm of other sites of floor of mouth	B04y.
Read	Other immunoproliferative neoplasms	B63y.
Read	Unspecified malignant neoplasm of lymphoid and histiocytic tissue of lymph nodes of axilla and upper limb	B62z4
Read	Lymphoid leukaemia	B64..
Read	Malignant neoplasm of anterior 2/3 of tongue dorsal surface	B0110
Read	Acute myeloblastic leukaemia with maturation	Xa3Ec
Read	Hodgkins paragranuloma of lymph nodes of head face and neck	B6101
Read	Malignant neoplasm of inguinal region NOS	B5530
Read	Nodular lymphoma of lymph nodes of head face and neck	B6201
Read	Benign paraproteinaemia	C3322
Read	[X]Malignant neoplasm of upper respiratory tract part unspecified	Byu22

Read	Malignant neoplasm overlapping lesion of biliary tract	B163.
Read	Lymphosarcoma	B601.
Read	Malignant neoplasm of duodenum	B120.
Read	Malignant neoplasm of cervix uteri	B41..
Read	Malignant neoplasm of flank NOS	B55y2
Read	Malignant lymphoma NOS of intrathoracic lymph nodes	B62y2
Read	Malignant neoplasm of hand bones	B305.
Read	Malignant neoplasm of auditory tube middle ear and mastoid air cells	B201.
Read	Malignant neoplasm of upper lip mucosa	B0022
Read	Malignant neoplasm of cardiac orifice of stomach	B1100
Read	Heavy chain disease	Xa0SM
Read	Malignant neoplasm of middle lobe bronchus or lung	B223.
Read	Malignant neoplasm of pylorus of stomach NOS	B111z
Read	Subacute leukaemia NOS	B682.
Read	Secondary and unspecified malignant neoplasm of superficial parotid lymph nodes	B5600
Read	Hodgkins sarcoma of unspecified site	B6120
Read	Malignant sacral teratoma	B3065
Read	Malignant neoplasm of carina of bronchus	B2210
Read	Malignant neoplasm of spinal cord	B522.
Read	Malignant neoplasm of connective and soft tissue of upper limb and shoulder	B311.
Read	Refractory anaemia with multilineage dysplasia	B9378
Read	Malignant neoplasm of sternum	B3031
Read	Paraproteinaemia	Xa0l6
Read	Malignant neoplasm of cardia of stomach	B110.
Read	T-cell acute lymphoblastic leukaemia	Xa0SH
Read	Malignant neoplasm of male breast NOS	B35zz
Read	Malignant neoplasm of short bones of leg	XE1vf
Read	Malignant neoplasm of lip unspecified	B007.
Read	[X]Malignant neoplasm of lip oral cavity and pharynx	Byu0.
Read	Pathological fracture due to metastatic bone disease	XaIJA
Read	Follicular non-Hodgkins lymphoma	XaBLw
Read	Chronic neutrophilic leukaemia	B6512
Read	Primary malignant neoplasm of unknown site	B593.
Read	Malignant neoplasm of floor of mouth	B04..
Read	Secondary and unspecified malignant neoplasm of intrapelvic lymph nodes	B565.
Read	Leukaemia NOS	B68z.
Read	Malignant neoplasm of genitourinary organ NOS	B4z..
Read	Malignant neoplasm of pharyngeal recess	B0720
Read	Malignant neoplasm of scrotum	B486.
Read	Hodgkins disease nodular sclerosis of intrathoracic lymph nodes	B6142
Read	Malignant histiocytosis of intra-abdominal lymph nodes	B6233
Read	Sezarys disease of intra-abdominal lymph nodes	B6223
Read	Malignant neoplasm of other specified pleura	B23y.
Read	Malignant neoplasm of epididymis	B484.
Read	Malignant neoplasm of other specified site of small intestine	B12y.
Read	Malignant neoplasm of connective and soft tissue of fore-arm	B3112
Read	Secondary malignant neoplasm of tongue	B58y9
Read	Malignant neoplasm of placenta	B42..
Read	Acute myeloblastic leukaemia - undifferentiated	Xa3EW
Read	Letterer-Siwe disease of lymph nodes of axilla and upper limb	B6254
Read	B-cell prolymphocytic leukaemia	Xa0T5
Read	Malignant neoplasm of connective and other soft tissue	B31..

Read	Malignant neoplasm of small intestine NOS	B12z.
Read	Secondary malignant neoplasm of skin of head	B5820
Read	Malignant reticuloendotheliosis	B62x3
Read	Malignant neoplasm of connective and soft tissue of abdominal wall	B3140
Read	Diffuse non-Hodgkins small cell (diffuse) lymphoma	B6273
Read	Malignant neoplasm of nasal cavities NOS	B200z
Read	Malignant neoplasm of lip	B00..
Read	Lymphoma with spill	Xa0SC
Read	[X]Malignant neoplasm of mesothelial and soft tissue	Byu5.
Read	Secondary and unspecified malignant neoplasm of inferior tracheobronchial lymph nodes	B5617
Read	Mast cell malignancy of intrapelvic lymph nodes	B6266
Read	Other monocytic leukaemia	B66y.
Read	Malignant neoplasm of carpal bone - pisiform	B3053
Read	Reticulosarcoma of intra-abdominal lymph nodes	B6003
Read	Malignant neoplasm of lower lobe bronchus or lung	B224.
Read	Malignant neoplasm of body of pancreas	B171.
Read	Secondary malignant neoplasm of jejunum	B5741
Read	Drash syndrome	K01w1
Read	Malignant neoplasm of mandible	B301.
Read	Burkitts lymphoma of lymph nodes of multiple sites	B6028
Read	Malignant neoplasm of cerebral pia mater	B5212
Read	Malignant neoplasm of respiratory tract NOS	B2zz.
Read	(Aleukaemic leukaemia) or (leukaemia - unspec cell NOS)	XE20V
Read	[X]Malignant neoplasm of ill-defined secondary and unspecified sites	ByuC.
Read	Malignant neoplasm of corpus uteri NOS	B430z
Read	Multifocal and multisystemic (disseminated) Langerhans-cell histiocytosis	B629.
Read	Malignant neoplasm of other site of respiratory tract	B2zy.
Read	[X]Malignant neoplasm of connective and soft tissue of trunk unspecified	Byu58
Read	Malignant neoplasm of parietal pleura	B230.
Read	IgG myeloma	Xa36b
Read	Malignant lymphoma NOS of intrapelvic lymph nodes	B62y6
Read	Malignant neoplasm of cerebrum NOS	B510z
Read	Malignant neoplasm of labia minora	B452.
Read	Malignant neoplasm of soft palate	B053.
Read	Malignant neoplasm of liver and intrahepatic bile ducts	B15..
Read	Malignant neoplasm of trachea	B220.
Read	Malignant melanoma stage IIC	B32C.
Read	Malignant melanoma of toe	B3278
Read	Hodgkins granuloma of intrathoracic lymph nodes	B6112
Read	Malignant neoplasm of ventral surface of tongue	B013.
Read	Malignant neoplasm of upper lobe bronchus	B2220
Read	Malignant neoplasm of lip unspecified lipstick area	B00z1
Read	Malignant neoplasm of pelvis	B553.
Read	Malignant neoplasm overlapping lesion of colon	B138.
Read	Malignant neoplasm of fifth metatarsal bone	B308C
Read	Non - Hodgkins lymphoma	B627.
Read	Malignant melanoma stage IV M1c	XacIf
Read	Malignant neoplasm of extrahepatic bile ducts	B161.
Read	B-cell prolymphocytic leukaemia	B64y3
Read	Malignant neoplasm of Meckels diverticulum	B123.
Read	Malignant neoplasm of gum NOS	B03z.
Read	Malignant neoplasm of ethmoid bone	B3000

Read	Malignant neoplasm of soft tissue of head	B3100
Read	Plasma cell leukaemia	XaBB3
Read	Fibrosarcoma of spleen	B1z11
Read	Malignant neoplasm of trachea NOS	B220z
Read	Malignant neoplasm of supraclavicular fossa NOS	B5505
Read	Malignant neoplasm of floor of cerebral ventricle	B5151
Read	Malignant neoplasm of upper-outer quadrant of female breast	B344.
Read	Secondary and unspecified malignant neoplasm of axillary lymph nodes	B5630
Read	Malignant neoplasm of pylorus of stomach	B111.
Read	Reticulosarcoma	B600.
Read	Malignant neoplasm of lower buccal sulcus	B0511
Read	Hodgkins disease lymphocytic depletion of intra-abdominal lymph nodes	B6163
Read	Malignant neoplasm of scapula	B3040
Read	Neuroblastoma	Xa99D
Read	[X]Malignant neoplasm of bone and articular cartilage	Byu3.
Read	Malignant neoplasm of peripheral nerves of trunk unspecified	B524X
Read	Malignant neoplasm of upper lobe bronchus or lung	XE1vb
Read	Malignant lymphoma NOS of lymph nodes of inguinal region and lower limb	B62y5
Read	Malignant melanoma of fore-arm	B3262
Read	Malignant neoplasm of posterior wall of oropharynx	B067.
Read	Unspecified malignant neoplasm of lymphoid and histiocytic tissue of intrathoracic lymph nodes	B62z2
Read	Malignant neoplasm of second metatarsal bone	B3089
Read	Malignant melanoma stage IA	B328.
Read	Malignant neoplasm of cranial nerves NOS	B520z
Read	Malignant neoplasm of connective and soft tissues of thoracic spine	B3133
Read	Secondary malignant neoplasm of other specified sites	B58..
Read	IgM monoclonal gammopathy of uncertain significance	Xa36h
Read	Malignant neoplasm of palate unspecified	B055.
Read	Malignant neoplasm of tarsus of eyelid	B3104
Read	Malignant neoplasm of fundus of corpus uteri	B4301
Read	Malignant neoplasm of ascending colon	B136.
Read	Malignant neoplasm of lip unspecified	XaC2J
Read	Malignant lymphoma NOS of lymph nodes of head face and neck	B62y1
Read	Plasma cell leukaemia	B631.
Read	Malignant neoplasm of other specified female genital organ	B45y.
Read	Monocytic leukaemia NOS	B66z.
Read	Malignant neoplasm of hypopharynx	B08..
Read	[X]Other specified malignant neoplasms of lymphoid haematopoietic and related tissue	ByuDA
Read	Malignant neoplasm of scapula and long bones of upper arm NOS	B304z
Read	[X]Malignant neoplasm of urinary tract	Byu9.
Read	Secondary and unspecified malignant neoplasm of popliteal lymph nodes	B5642
Read	Letterer-Siwe disease NOS	B625z
Read	Malignant neoplasm of abdominal oesophagus	B102.
Read	Malignant neoplasm of genitourinary organ	XE2vP
Read	Mycosis fungoides NOS	B621z
Read	[X]Malignant neoplasm of breast	Byu6.
Read	Hodgkins disease nodular sclerosis of intra-abdominal lymph nodes	B6143
Read	Siewert type I adenocarcinoma	B107.
Read	Malignant neoplasm of labia majora	B451.
Read	[X]Malignant neoplasm of ill-defined sites within the digestive system	Byu13
Read	Secondary and unspecified malignant neoplasm of infraclavicular lymph nodes	B5632
Read	B-cell chronic lymphocytic leukaemia	Xa0QP

Read	Malignant neoplasm of olfactory bulb	B5200
Read	Malignant neoplasm of perinephric tissue	B1801
Read	Secondary and unspecified malignant neoplasm of paratracheal lymph nodes	B5615
Read	Malignant neoplasm of retroperitoneum NOS	B180z
Read	Adult T-cell lymphoma/leukaemia (HTLV-1-associated)	B64y5
Read	Leukaemic reticuloendotheliosis of intrathoracic lymph nodes	B6242
Read	Malignant neoplasm of lip oral cavity and pharynx NOS	B0zz.
Read	Malignant histiocytosis of unspecified site	B6230
Read	Malignant neoplasm of lip oral cavity and pharynx	XE2rk
Read	Malignant neoplasm of carpal bone - scaphoid	B3050
Read	Leukaemia	X78e2
Read	Refractory anaemia with excess blasts	Xa0Sf
Read	Malignant neoplasm of small intestine and duodenum	B12..
Read	Nodular lymphoma NOS	B620z
Read	Malignant neoplasm of undescended testis	B470.
Read	Myelomonocytic leukaemia	B69..
Read	Malignant neoplasm of specified site NOS	B55yz
Read	Hodgkins granuloma of spleen	B6117
Read	Malignant neoplasm of connective and soft tissue of thigh and upper leg	B3121
Read	Malignant neoplasm of intestinal tract part unspecified	B1z0.
Read	Malignant melanoma of face NOS	B323z
Read	Malignant neoplasm of lateral wall of nasopharynx	B072.
Read	Malignant melanoma of external auditory meatus	B3221
Read	Malignant neoplasm of lip oral cavity and pharynx	B0...
Read	Acute myelofibrosis	Xa0So
Read	Malignant neoplasm of hard palate	B052.
Read	Other types of follicular lymphoma	B6287
Read	Malignant neoplasm of pyloric antrum of stomach	B112.
Read	Malignant neoplasm of labia majora NOS	B451z
Read	[X]Malignant neoplasm overlapping lesion of skin	Byu5A
Read	Secondary malignant neoplasm of bladder	B5811
Read	Malignant neoplasm of ectopic pancreatic tissue	B17y0
Read	Juvenile myelomonocytic leukaemia	XaYdL
Read	Atypical hairy cell leukaemia	XaB47
Read	Malignant histiocytosis of intrathoracic lymph nodes	B6232
Read	Diffuse non-Hodgkins mixed small and large cell (diffuse) lymphoma	B6275
Read	Secondary malignant neoplasm of other part of nervous system	B584.
Read	Malignant neoplasm of pericardium	B2413
Read	Hodgkins disease nodular sclerosis of intrapelvic lymph nodes	B6146
Read	Malignant neoplasm overlapping lesion of peripheral nerves and autonomic nervous system	B5246
Read	Secondary and unspecified malignant neoplasm of intrapelvic lymph nodes NOS	B565z
Read	Malignant histiocytosis of lymph nodes of axilla and upper limb	B6234
Read	Malignant neoplasm of connective and soft tissue of upper limb and shoulder NOS	B311z
Read	Primary angiosarcoma of liver	B1502
Read	Malignant neoplasm of bone and articular cartilage NOS	B30z.
Read	Primary malignant neoplasm of unknown site	XaCJ1
Read	Malignant neoplasm of epiglottis NOS	B215.
Read	Leukaemic reticuloendotheliosis of lymph nodes of multiple sites	B6248
Read	Peripheral T-cell lymphoma	Xa0Th
Read	Secondary malignant neoplasm of vulva	B58y4
Read	Malignant neoplasm of vermilion border of lip unspecified	B00z.
Read	Nodular lymphocyte predominant Hodgkin lymphoma	B617.

Read	Malignant melanoma of chest wall	B3258
Read	Malignant lymphoma NOS of lymph nodes of axilla and upper limb	B62y4
Read	Secondary and unspecified malignant neoplasm of superficial tracheobronchial lymph nodes	B5616
Read	Malignant melanoma stage IIIA	XacIZ
Read	Malignant melanoma of hip	B3270
Read	Light chain monoclonal gammopathy of uncertain significance	Xa36m
Read	Malignant neoplasm of tail of pancreas	B172.
Read	Malignant neoplasm of anterior epiglottis NOS	B064z
Read	Malignant neoplasm of stomach	B11..
Read	[X]Other and unspecified peripheral and cutaneous T-cell lymphomas	ByuDD
Read	Malignant neoplasm of spinal meninges	B523.
Read	Malignant melanoma stage IIA	XacIW
Read	Malignant neoplasm of other sites of rectum rectosigmoid junction and anus	B14y.
Read	Malignant neoplasm of peripheral nerves of lower limb including hip	B5242
Read	Malignant neoplasm of septum of nose	B2002
Read	Hodgkins disease mixed cellularity of lymph nodes of head face and neck	B6151
Read	Malignant neoplasm of lower lobe bronchus or lung NOS	B224z
Read	Primary malignant neoplasm of liver NOS	B150z
Read	Lymphosarcoma of unspecified site	B6010
Read	Malignant neoplasm of aortic body	B5451
Read	Malignant neoplasm of descended testis NOS	B471z
Read	Malignant neoplasm of choroid plexus	B5150
Read	Mixed cellularity classical Hodgkin lymphoma	B619.
Read	Malignant neoplasm of carpal bone - hamate	B3057
Read	Malignant neoplasm of ovary and other uterine adnexa	B44..
Read	Malignant neoplasm of other sites of tongue	B01y.
Read	Hodgkins disease mixed cellularity	B615.
Read	Secondary malignant neoplasm of skin of neck	B5822
Read	Malignant lymphoma NOS of spleen	B62y7
Read	Secondary malignant neoplasm of ileum	B5742
Read	Leukaemic reticuloendotheliosis of lymph nodes of head face and neck	B6241
Read	Malignant neoplasm of great vessels	B3132
Read	Hodgkins disease nodular sclerosis of lymph nodes of inguinal region and lower limb	B6145
Read	Malignant neoplasm of commissure of lip	B005.
Peptic ulcer		
Read	Non steroidal anti inflammatory drug induced gastric ulcer NOS	XaMO6
Read	Unspecified peptic ulcer with haemorrhage and perforation	J13y3
Read	Unspecified peptic ulcer with unspecified haemorrhage and/or perforation	J13yy
Read	Gastrojejunal ulcer	XE0aS
Read	Unspecified gastrojejunal ulcer	J14y.
Read	Acute peptic ulcer with perforation	J1302
Read	Chronic gastric ulcer without mention of complication	J1110
Read	Deformed duodenal cap	XaBOE
Read	Unspecified peptic ulcer NOS	J13yz
Read	Gastric ulcer (& [prepyloric] or [pyloric])	J11..
Read	Chronic drug-induced ulcer of stomach	X301K
Read	Pyloric ulcer	Xa84h
Read	Stress ulcer of duodenum	X302B
Read	Acute gastric ulcer with haemorrhage	J1101
Read	Anti-platelet induced duodenal ulcer NOS	XaLWt
Read	Perforated DU (& [acute])	XE0c1
Read	Peptic ulcer of stomach	X302X

Read	Perforated peptic ulcer (& [acute PU])	XE0c5
Read	Chronic peptic ulcer with haemorrhage and perforation	J1313
Read	Chronic duodenal ulcer with obstruction	J1214
Read	Non-reflux ulcer of oesophagus	Xa9C0
Read	Acute gastric ulcer	J110.
Read	Acute duodenal ulcer without mention of complication	J1200
Read	Barretts ulcer of oesophagus	J1025
Read	Unspecified gastric ulcer NOS	J11yz
Read	Acute peptic ulcer with haemorrhage and perforation	J1303
Read	Unspecified gastrojejunal ulcer with unspecified haemorrhage or perforation	J14yy
Read	Chronic peptic ulcer of stomach	X301J
Read	Acute duodenal ulcer NOS	J120z
Read	Viral ulcer of oesophagus	X300N
Read	Unspecified gastric ulcer	J11y.
Read	Acute gastric ulcer without mention of complication	J1100
Read	Acute peptic ulcer with haemorrhage	J1301
Read	Acute gastrojejunal ulcer without mention of complication	J1400
Read	Acute drug-induced ulcer of stomach	X301F
Read	Unspecified peptic ulcer with perforation	J13y2
Read	Acute peptic ulcer without mention of complication	J1300
Read	Bacterial ulcer of oesophagus	X300M
Read	Unspecified duodenal ulcer with haemorrhage	J12y1
Read	Cushings ulcer of stomach	X301H
Read	Perforation of gastric ulcer	X301o
Read	Chronic gastric ulcer unspecified	J111y
Read	Chronic peptic ulcer unspecified	J131y
Read	Duodenal ulcer disease	J122.
Read	Unspecified duodenal ulcer NOS	J12yz
Read	Ulcer: [gastrojejunal] or [anastomotic] or [gastrocolic] or [jejunal] or [marginal] or [stomal]	J14..
Read	Duodenal ulcer - (DU)	J12..
Read	Multiple gastric ulcers	XaELE
Read	Chemical ulcer of oesophagus	J1023
Read	Perforation of duodenal ulcer	X302Q
Read	Chronic gastrojejunal ulcer unspecified	J141y
Read	Unspecified gastrojejunal ulcer with perforation	J14y2
Read	Unspecified gastric ulcer with unspecified haemorrhage and/or perforation	J11yy
Read	Chronic duodenal ulcer with haemorrhage	J1211
Read	Jejunal ulcer	Xa1qB
Read	Unspecified gastrojejunal ulcer without mention of complication	J14y0
Read	Duodenal ulcer disease	X302b
Read	Chronic duodenal ulcer without mention of complication	J1210
Read	Acute gastrojejunal ulcer with perforation	J1402
Read	Gastrocolic ulcer	Xa1qC
Read	Chronic peptic ulcer of duodenum	X302F
Read	Anti-platelet induced gastric ulcer NOS	XaLWr
Read	Unspecified gastrojejunal ulcer with haemorrhage and perforation	J14y3
Read	Gastric ulcer NOS	XE0aQ
Read	Peptic ulcer of oesophagus	J1020
Read	Acute gastrojejunal ulcer with haemorrhage and perforation	J1403
Read	Oesophageal ulcer due to aspirin	J1022
Read	Chronic gastrojejunal ulcer	J141.
Read	Chronic gastrojejunal ulcer with haemorrhage and perforation	J1413

Read	Anti-platelet induced duodenal ulcer	XaLWs
Read	Curlings ulcer of duodenum	X302D
Read	Acute gastrojejunal ulcer with haemorrhage	J1401
Read	Chronic gastrojejunal ulcer with perforation	J1412
Read	Unspecified duodenal ulcer with perforation	J12y2
Read	Chronic gastrojejunal ulcer with haemorrhage	J1411
Read	Unspecified peptic ulcer with haemorrhage	J13y1
Read	Chronic peptic ulcer with obstruction	J1314
Read	Chronic peptic ulcer without mention of complication	J1310
Read	Chronic peptic ulcer with perforation	J1312
Read	Peptic ulcer	XM0BZ
Read	Chronic peptic ulcer with haemorrhage	J1311
Read	Peptic ulcer - (PU) site unspecified	XE0aR
Read	Acute duodenal ulcer unspecified	J120y
Read	Chronic gastric ulcer NOS	J111z
Read	Infective ulcer of oesophagus	X300L
Read	Non steroidal anti inflammatory drug induced gastric ulcer	XaMO5
Read	Eosinophilic ulcer of oesophagus	X300K
Read	Chronic peptic ulcer NOS	J131z
Read	Acute gastrojejunal ulcer NOS	J140z
Read	Chronic duodenal ulcer with perforation	J1212
Read	Acute peptic ulcer	J130.
Read	Acute duodenal ulcer with perforation	J1202
Read	Duodenal ulcer NOS	J12z.
Read	Acute duodenal ulcer with haemorrhage	J1201
Read	Acute gastrojejunal ulcer unspecified	J140y
Read	Cushings ulcer of duodenum	X302C
Read	Unspecified duodenal ulcer with haemorrhage and perforation	J12y3
Read	Chronic peptic ulcer	J131.
Read	Recurrent duodenal ulcer	J124.
Read	Stress ulceration of oesophagus	X300Q
Read	Stress ulcer of stomach	X301G
Read	Unspecified peptic ulcer	J13y.
Read	Chronic gastrojejunal ulcer without mention of complication	J1410
Read	Ulcer of oesophagus NOS	J102z
Read	Acute peptic ulcer with obstruction	J1304
Read	Acute peptic ulcer of duodenum	X302A
Read	Chronic duodenal ulcer NOS	J121z
Read	Chronic duodenal ulcer unspecified	J121y
Read	Peptic ulcer - (PU) site unspecified	J13..
Read	Chronic duodenal ulcer with haemorrhage and perforation	J1213
Read	Anti-platelet induced duodenal ulcer	J125.
Read	Perforated GU (& [acute])	XE0bz
Read	Anti-platelet induced gastric ulcer NOS	J112z
Read	Perforated peptic ulcer	XM0sI
Read	Chronic gastrojejunal ulcer with obstruction	J1414
Read	Unspecified gastric ulcer with obstruction	J11y4
Read	Acute duodenal ulcer with obstruction	J1204
Read	Acute gastric ulcer with perforation	J1102
Read	Radiation ulcer of oesophagus	X300P
Read	Gastric ulcer NOS	J11z.
Read	Chronic gastric ulcer with haemorrhage	J1111

Read	Unspec duodenal ulcer; unspec haemorrhage and/or perforation	J12yy
Read	Bleeding stress ulcer of stomach	XaBel
Read	Peptic ulcer NOS	J13z.
Read	Gastric ulcer	XE0aP
Read	ADU - Acute duodenal ulcer	J120.
Read	Chronic gastrojejunal ulcer NOS	J141z
Read	Chronic duodenal ulcer	J121.
Read	Unspecified gastric ulcer with perforation	J11y2
Read	Bleeding peptic ulcer	XaBmb
Read	Unspecified gastric ulcer without mention of complication	J11y0
Read	Protozoal ulcer of oesophagus	X300O
Read	Non steroidal anti inflammatory drug induced gastric ulcer	J113.
Read	Chronic gastric ulcer	J111.
Read	Non steroidal anti inflammatory drug induced duodenal ulcer	J126.
Read	Oesophageal ulcer	J102.
Read	Acute peptic ulcer unspecified	J130y
Read	Unspecified gastrojejunal ulcer with haemorrhage	J14y1
Read	Unspecified duodenal ulcer with obstruction	J12y4
Read	Chronic gastric ulcer with perforation	J1112
Read	Curlings ulcer of stomach	X301I
Read	Acute gastrojejunal ulcer	J140.
Read	Unspecified peptic ulcer with obstruction	J13y4
Read	Prepyloric gastric ulcer	Xa6ot
Read	Acute gastric ulcer unspecified	J110y
Read	Unspecified gastric ulcer with haemorrhage	J11y1
Read	Unspecified duodenal ulcer without mention of complication	J12y0
Read	Acute peptic ulcer NOS	J130z
Read	Acute gastric ulcer with haemorrhage and perforation	J1103
Read	Chronic gastric ulcer with haemorrhage and perforation	J1113
Read	Acute gastric ulcer with obstruction	J1104
Read	Peptic ulcer of duodenum	X302c
Read	Acute gastrojejunal ulcer with obstruction	J1404
Read	Acute peptic ulcer of stomach	X301E
Read	Drug-induced ulcer of oesophagus	J1024
Read	Acute duodenal ulcer with haemorrhage and perforation	J1203
Read	Unspecified duodenal ulcer	J12y.
Read	Anti-platelet induced gastric ulcer	J112.
Read	Anti-platelet induced duodenal ulcer NOS	J125z
Read	Acute gastric ulcer NOS	J110z
Read	Unspecified gastric ulcer with haemorrhage and perforation	J11y3
Read	Anastomotic ulcer	Xa1qA
Read	Chronic gastric ulcer with obstruction	J1114
Read	Unspecified gastrojejunal ulcer NOS	J14yz
Read	Anti-platelet induced gastric ulcer	XaLWq
Read	Fungal ulcer of oesophagus	J1021
Read	Ulcerative oesophagitis	J1016
Read	Unspecified gastrojejunal ulcer with obstruction	J14y4
Read	Unspecified peptic ulcer without mention of complication	J13y0
Read	Gastrojejunal ulcer NOS	J14z.
SNOMED	Duodenal ulcer excision (procedure)	287828006
SNOMED	Duodenal ulcer with increased serum pepsinogen I	29755007
SNOMED	Gastrojejunal ulcer with perforation but without obstruction (disorder)	11818002

SNOMED	Non-reflux ulcer of oesophagus	302915007
SNOMED	Duodenal ulcer suture	307260009
SNOMED	Chronic gastric ulcer with obstruction	196639001
SNOMED	Anti-platelet induced gastric ulcer NOS (disorder)	2.06441E+14
SNOMED	Bleeding stress ulcer of stomach	308882008
SNOMED	Unspecified gastric ulcer with obstruction	196647001
SNOMED	Chronic gastric ulcer with perforation	196637004
SNOMED	Chronic peptic ulcer with haemorrhage (disorder)	155706006
SNOMED	Pyloric ulcer (disorder)	39204006
SNOMED	Duodenal ulcer	155691003
SNOMED	Acute gastrojejunal ulcer NOS	196714003
SNOMED	Gastrojejunal ulcer without haemorrhage, without perforation AND without obstruction	35517004
SNOMED	Esophagogastric ulcer (disorder)	10699001
SNOMED	Non-steroidal anti-inflammatory drug induced gastric ulcer NOS (disorder)	2.49321E+14
SNOMED	Unspecified peptic ulcer (disorder)	196697002
SNOMED	Chronic peptic ulcer NOS (disorder)	6.52941E+14
SNOMED	Gastric ulcer caused by antiplatelet agent	424301005
SNOMED	Acute gastric ulcer NOS	155685000
SNOMED	Chronic gastrojejunal ulcer without hemorrhage AND without perforation (disorder)	4269005
SNOMED	Gastric ulcer - (GU)	397825006
SNOMED	Acute duodenal ulcer NOS	6.18291E+14
SNOMED	Chronic duodenal ulcer without hemorrhage or perforation	40214005
SNOMED	Acute duodenal ulcer with hemorrhage AND with perforation but without obstruction	51847008
SNOMED	Chronic gastrojejunal ulcer without hemorrhage, without perforation AND without obstruction (disorder)	41626001
SNOMED	Acute duodenal ulcer with haemorrhage, with perforation AND with obstruction	41986000
SNOMED	Ulcer of stomach due to lymphocytic gastritis	724521003
SNOMED	Chronic peptic ulcer with haemorrhage	49232000
SNOMED	Unspecified gastrojejunal ulcer with unspecified haemorrhage or perforation	196728009
SNOMED	Acute marginal gastrojejunal ulcer (disorder)	1.83281E+14
SNOMED	Acute gastrojejunal ulcer	196707000
SNOMED	Helicobacter pylori associated duodenal ulcer	724528009
SNOMED	Peptic ulcer without hemorrhage AND without perforation but with obstruction (disorder)	54157007
SNOMED	Unspecified duodenal ulcer NOS (disorder)	6.12831E+14
SNOMED	Duodenal ulcer with haemorrhage	27281001
SNOMED	Acute gastric ulcer with hemorrhage AND obstruction (disorder)	46708007
SNOMED	Gastrojejunal ulcer with haemorrhage AND perforation	64094003
SNOMED	Incision of stomach and suture of bleeding gastric ulcer (procedure)	442805003
SNOMED	Duodenal ulcer NOS (disorder)	5.91721E+14
SNOMED	Unspecified gastric ulcer with perforation	6.06451E+14
SNOMED	Chronic peptic ulcer with perforation AND obstruction (disorder)	57871005
SNOMED	Unspecified gastrojejunal ulcer with haemorrhage	6.23201E+14
SNOMED	Gastrojejunal ulcer with hemorrhage but without obstruction (disorder)	50663005
SNOMED	Acute gastrojejunal ulcer without hemorrhage, without perforation AND without obstruction	77987006
SNOMED	Chronic gastrojejunal ulcer	128288009
SNOMED	Acute gastric ulcer with haemorrhage	196629007
SNOMED	Peptic ulcer, NOS with perforation and obstruction	48336009
SNOMED	Multiple gastric ulcers (disorder)	313425006
SNOMED	Gastric ulcer caused by alcohol	723103002
SNOMED	Chronic peptic ulcer without mention of complication	5.29371E+14
SNOMED	Acute duodenal ulcer NOS	155695007
SNOMED	Cushing ulcer of esophagus	738792004
SNOMED	Gastrojejunal ulcer, NOS with perforation	30183003

SNOMED	Unspecified peptic ulcer with haemorrhage and perforation	6.40821E+14
SNOMED	Normopepsinogenemic familial duodenal ulcer	76338009
SNOMED	Infectious gastric ulcer	762274007
SNOMED	Peptic ulcer of duodenum	553009
SNOMED	Peptic ulcer with hemorrhage AND perforation	55617001
SNOMED	Chronic peptic ulcer with hemorrhage AND obstruction	56461008
SNOMED	Chronic gastrojejunal ulcer with haemorrhage	62838000
SNOMED	Unspecified peptic ulcer with haemorrhage and perforation	196701004
SNOMED	Ulcer of upper gastrointestinal tract with obstruction	786068005
SNOMED	Unspecified duodenal ulcer with hemorrhage (disorder)	5.91661E+14
SNOMED	Duodenal ulcer without hemorrhage AND without perforation but with obstruction	18169007
SNOMED	Gastric ulcer, NOS with perforation but without obstruction	84038009
SNOMED	Chronic duodenal ulcer with perforation but without obstruction	34602004
SNOMED	Acute peptic ulcer NOS	155705005
SNOMED	Chronic gastric ulcer with hemorrhage and with perforation	76181002
SNOMED	Gastric ulcer with hemorrhage and perforation	62366003
SNOMED	Acute peptic ulcer NOS	5.91741E+14
SNOMED	Gastrojejunal ulcer, NOS with hemorrhage and perforation but without obstruction	87796008
SNOMED	Acute gastric ulcer NOS (disorder)	196634006
SNOMED	Unspecified peptic ulcer with obstruction	5.98481E+14
SNOMED	Chronic peptic ulcer with hemorrhage but without obstruction (disorder)	81518000
SNOMED	Acute duodenal ulcer with hemorrhage (disorder)	12847006
SNOMED	Acute duodenal ulcer with haemorrhage and perforation	155694006
SNOMED	Common duodenal ulcer	367474008
SNOMED	Acute duodenal ulcer without haemorrhage AND without perforation	32490005
SNOMED	Gastric ulcer, NOS without hemorrhage or perforation but with obstruction	31452001
SNOMED	Acute gastrojejunal ulcer without hemorrhage AND without perforation (disorder)	30514008
SNOMED	Acute gastric ulcer without hemorrhage AND without perforation	67964002
SNOMED	Unspecified gastric ulcer with haemorrhage	6.06441E+14
SNOMED	Chronic gastrojejunal ulcer without hemorrhage AND without perforation but with obstruction (disorder)	56579005
SNOMED	Unspecified duodenal ulcer with obstruction	196677007
SNOMED	Acute gastric ulcer with haemorrhage but without obstruction	70418001
SNOMED	Chronic gastric ulcer with haemorrhage and perforation (disorder)	155688003
SNOMED	Chronic duodenal ulcer with hemorrhage and perforation	36975000
SNOMED	Peptic ulcer of esophagus	6129004
SNOMED	Duodenal ulcer NOS	196680008
SNOMED	Chronic gastric ulcer NOS	196641000
SNOMED	Unspecified duodenal ulcer with haemorrhage and perforation	6.12781E+14
SNOMED	Unspecified duodenal ulcer with obstruction (disorder)	6.12791E+14
SNOMED	Chronic gastrojejunal ulcer with hemorrhage AND perforation (disorder)	45640006
SNOMED	Bleeding ulcer of esophagus	57748001
SNOMED	Acute gastric ulcer with hemorrhage	155682002
SNOMED	Peptic ulcer, NOS without hemorrhage or perforation and without obstruction	38365000
SNOMED	Gastric ulcer with hemorrhage and obstruction	53877005
SNOMED	Chronic gastric ulcer with hemorrhage (disorder)	57246001
SNOMED	Non-steroidal anti-inflammatory drug induced duodenal ulcer NOS	2.49331E+14
SNOMED	Chronic peptic ulcer with haemorrhage and perforation (disorder)	155708007
SNOMED	Acute peptic ulcer with hemorrhage AND with perforation but without obstruction (disorder)	47064007
SNOMED	Gastrojejunal ulcer, NOS with perforation and obstruction	21759003
SNOMED	Acute peptic ulcer with haemorrhage	155702008
SNOMED	Anti-platelet induced gastric ulcer (disorder)	2.05331E+14
SNOMED	Curling's ulcer of stomach	39755000

SNOMED	Chronic gastric ulcer unspecified	6.06401E+14
SNOMED	Ulcer of esophagus due to ingestion of chemicals (disorder)	62465000
SNOMED	Chronic gastric ulcer without haemorrhage AND without perforation but with obstruction	60531007
SNOMED	Gastrojejunal ulcer	266501005
SNOMED	Chronic duodenal ulcer unspecified	6.33191E+14
SNOMED	Peptic ulcer NOS	5.98511E+14
SNOMED	Unspecified duodenal ulcer	6.33211E+14
SNOMED	Unspecified duodenal ulcer NOS	196679005
SNOMED	Acute drug-induced ulcer of stomach (disorder)	235648004
SNOMED	Acute duodenal ulcer with perforation but without obstruction	22511002
SNOMED	Unspecified gastrojejunal ulcer with haemorrhage and perforation	6.42891E+14
SNOMED	Gastric ulcer caused by drug	713638002
SNOMED	Acute peptic ulcer without mention of complication	196683005
SNOMED	Acute duodenal ulcer without hemorrhage or perforation and without obstruction	23693000
SNOMED	Ulcer of esophagus NOS (disorder)	6.11561E+14
SNOMED	Duodenal ulcer with haemorrhage AND obstruction	18367003
SNOMED	Unspecified peptic ulcer	6.52951E+14
SNOMED	Chronic gastrojejunal ulcer with obstruction	196719008
SNOMED	Acute duodenal ulcer with hemorrhage but without obstruction (disorder)	66767006
SNOMED	Unspecified gastric ulcer without mention of complication	196643002
SNOMED	Unspecified gastrojejunal ulcer without mention of complication	6.11581E+14
SNOMED	Chronic gastric ulcer without haemorrhage, without perforation AND without obstruction	1567007
SNOMED	Chronic gastrojejunal ulcer unspecified	6.23181E+14
SNOMED	Deformed duodenal bulb	308212008
SNOMED	Acute duodenal ulcer without mention of complication	196653001
SNOMED	Protozoal ulcer of esophagus (disorder)	235612001
SNOMED	Gastrojejunal ulcer	155701001
SNOMED	Duodenal ulcer excision (procedure)	149381000
SNOMED	Unspecified gastric ulcer without mention of complication	6.06431E+14
SNOMED	Unspecified gastric ulcer with perforation	196645009
SNOMED	Viral duodenal ulcer	724533008
SNOMED	Acute gastrojejunal ulcer with perforation	196710007
SNOMED	Ulcer of esophagus caused by ingestion of aspirin (disorder)	33953007
SNOMED	Gastric ulcer due to parasitic infection (disorder)	723102007
SNOMED	Unspecified gastric ulcer with unspecified hemorrhage and/or perforation	196648006
SNOMED	Acute gastrojejunal ulcer unspecified	196713009
SNOMED	Chronic duodenal ulcer with perforation AND obstruction	60551006
SNOMED	Unspecified duodenal ulcer with hemorrhage and perforation (disorder)	196676003
SNOMED	Ulcer of oesophagus	155674002
SNOMED	Unspecified peptic ulcer with haemorrhage	6.52981E+14
SNOMED	Ulcer of esophagus due to gastro-esophageal reflux disease with complication	717848003
SNOMED	Perforated peptic ulcer (& [acute PU])	266502003
SNOMED	Unspecified gastrojejunal ulcer with haemorrhage and perforation	196726008
SNOMED	Eosinophilic ulcer of oesophagus	235608007
SNOMED	Chronic peptic ulcer	128287004
SNOMED	Ulcer of esophagus NOS	196610001
SNOMED	Acute duodenal ulcer	196652006
SNOMED	Acute gastrojejunal ulcer with perforation	66636001
SNOMED	Gastric ulcer without hemorrhage AND without perforation	73481001
SNOMED	Acute peptic ulcer unspecified	196688001
SNOMED	Acute duodenal ulcer with perforation (disorder)	196656009
SNOMED	Acute duodenal ulcer with haemorrhage and perforation	196657000

SNOMED	Acute peptic ulcer with haemorrhage AND obstruction	43406003
SNOMED	Chronic duodenal ulcer with haemorrhage (disorder)	155696008
SNOMED	Chronic gastric ulcer with hemorrhage	155686004
SNOMED	Gastric ulcer	196627009
SNOMED	Ulcer of duodenum due to infection (disorder)	762276009
SNOMED	Chronic peptic ulcer of stomach	235649007
SNOMED	Chronic gastric ulcer without hemorrhage AND without perforation	76796008
SNOMED	Duodenal ulcer, NOS with hemorrhage and perforation	23812009
SNOMED	Duodenal ulcer with perforation AND obstruction	77410006
SNOMED	Chronic duodenal ulcer without mention of complication (disorder)	196661006
SNOMED	Acute ulcerative gastroenteritis complicating pneumonia (disorder)	109814008
SNOMED	Chronic duodenal ulcer	128286008
SNOMED	Chronic gastrojejunal ulcer with perforation but without obstruction (disorder)	62477005
SNOMED	Acute peptic ulcer with perforation	79118000
SNOMED	Acute gastrojejunal ulcer with hemorrhage (disorder)	196709002
SNOMED	Duodenal ulcer, NOS with hemorrhage and perforation and with obstruction	12355008
SNOMED	Acute duodenal ulcer with haemorrhage	155692005
SNOMED	Acute gastrojejunal ulcer with haemorrhage and perforation	81387001
SNOMED	Chronic drug-induced ulcer of stomach (disorder)	235650007
SNOMED	Cushing ulcer of duodenum	235689001
SNOMED	Platelet aggregation inhibitor-induced gastric ulcer	840592004
SNOMED	Chronic gastric ulcer with hemorrhage AND with perforation but without obstruction (disorder)	74341002
SNOMED	Acute peptic ulcer with hemorrhage and perforation	111353003
SNOMED	Non-steroidal anti-inflammatory drug induced gastric ulcer	2.48891E+14
SNOMED	Duodenal ulcer caused by fungus	724534002
SNOMED	Eosinophilic duodenal ulcer	717878007
SNOMED	Chronic gastric ulcer with hemorrhage and with obstruction	85859006
SNOMED	Acute peptic ulcer with obstruction	196687006
SNOMED	Acute duodenal ulcer with haemorrhage AND perforation	86895006
SNOMED	Unspecified gastric ulcer with unspecified hemorrhage and/or perforation (disorder)	6.06481E+14
SNOMED	Acute duodenal ulcer with hemorrhage AND obstruction	87756006
SNOMED	Gastrojejunal ulcer with obstruction (disorder)	8.49621E+14
SNOMED	Fungal ulcer of esophagus	88700009
SNOMED	Chronic gastric ulcer without mention of complication	5.29341E+14
SNOMED	Acute peptic ulcer with perforation	155703003
SNOMED	Chronic gastrojejunal ulcer with hemorrhage and obstruction	90257004
SNOMED	Chronic duodenal ulcer with hemorrhage but without obstruction	62341002
SNOMED	Acute peptic ulcer with haemorrhage and perforation	196686002
SNOMED	Acute gastric ulcer	95529005
SNOMED	Ulcer of esophagus due to ingestion of medicines	64595005
SNOMED	Chronic gastric ulcer NOS (disorder)	155689006
SNOMED	Chronic duodenal ulcer NOS (disorder)	155699001
SNOMED	Peptic ulcer - (PU) site unspecified	6.24761E+14
SNOMED	Gastric ulcer caused by virus	723100004
SNOMED	Peptic ulcer with haemorrhage AND with perforation but without obstruction	90489006
SNOMED	Chronic peptic ulcer without hemorrhage AND without perforation	5492000
SNOMED	Acute peptic ulcer without hemorrhage AND without perforation	45485004
SNOMED	Acute gastric ulcer with haemorrhage and perforation	155684001
SNOMED	Deformed duodenal cap	196774008
SNOMED	Peptic ulcer of newborn	276525003
SNOMED	Chronic peptic ulcer with haemorrhage AND perforation	61300005
SNOMED	Chemical gastric ulcer	723105009

SNOMED	Fungal gastric ulcer	723101000
SNOMED	Bleeding chronic gastric ulcer	196636008
SNOMED	Acute gastric ulcer with hemorrhage AND perforation (disorder)	196631003
SNOMED	Chronic gastrojejunal ulcer without mention of complication	5.29321E+14
SNOMED	Chronic duodenal ulcer with haemorrhage AND with perforation but without obstruction	81142005
SNOMED	Peptic ulcer of esophagus	30811009
SNOMED	Acute gastric ulcer with perforation but without obstruction	90628007
SNOMED	Unspecified peptic ulcer without mention of complication	196698007
SNOMED	Chronic peptic ulcer of duodenum (disorder)	235691009
SNOMED	Gastric ulcer, NOS with hemorrhage but without obstruction	16694003
SNOMED	Chronic duodenal ulcer without mention of complication	5.29551E+14
SNOMED	Gastric stress ulcer	415624002
SNOMED	Gastrojejunal ulcer with haemorrhage	84124004
SNOMED	Non-steroidal anti-inflammatory drug induced duodenal ulcer	2.48901E+14
SNOMED	Unspecified gastric ulcer	6.06421E+14
SNOMED	Unspecified gastrojejunal ulcer	196722005
SNOMED	Anti-platelet induced gastric ulcer NOS	2.08721E+14
SNOMED	Unspecified duodenal ulcer	196672001
SNOMED	Unspecified gastric ulcer NOS	6.06491E+14
SNOMED	Unspecified gastrojejunal ulcer NOS (disorder)	6.17001E+14
SNOMED	Acute peptic ulcer with hemorrhage (disorder)	196684004
SNOMED	Acute gastrojejunal ulcer with hemorrhage but without obstruction (disorder)	59515005
SNOMED	Duodenal ulcer with haemorrhage but without obstruction	35560008
SNOMED	Sepsis-related gastrointestinal ulceration	238152004
SNOMED	Viral ulcer of esophagus	235611008
SNOMED	Gastric ulcer NOS (disorder)	155690002
SNOMED	Gastrocolic ulcer	24060004
SNOMED	Helicobacter pylori associated and drug-induced gastric ulcer	724519008
SNOMED	Acute peptic ulcer with haemorrhage and perforation (disorder)	155704009
SNOMED	Acute gastric ulcer without haemorrhage, without perforation AND without obstruction	54053008
SNOMED	Chronic gastrojejunal ulcer with perforation AND with obstruction (disorder)	10897002
SNOMED	Chronic peptic ulcer with perforation	3483000
SNOMED	Ulcer of esophagus caused by ingestion of aspirin	196607008
SNOMED	Chronic gastrojejunal ulcer with perforation	196717005
SNOMED	Duodenal ulcer with obstruction (disorder)	8.49601E+14
SNOMED	Gastrojejunal ulcer (GJU)	196706009
SNOMED	Peptic ulcer NOS	196705008
SNOMED	Bacterial gastric ulcer	723099007
SNOMED	Duodenal ulcer with haemorrhage AND with perforation but without obstruction	15115006
SNOMED	Duodenal ulcer disease (disorder)	51868009
SNOMED	Peptic ulcer without hemorrhage AND without perforation	37442009
SNOMED	Chronic gastric ulcer with perforation and obstruction	55483002
SNOMED	Acute peptic ulcer without hemorrhage AND without perforation but with obstruction	58085004
SNOMED	Chronic duodenal ulcer without hemorrhage or perforation but with obstruction	28082003
SNOMED	Drug-induced duodenal ulcer	724529001
SNOMED	Recurrent duodenal ulcer	196671008
SNOMED	Chronic gastrojejunal ulcer with hemorrhage and perforation but without obstruction	46523000
SNOMED	Non steroidal anti inflammatory drug induced gastric ulcer	2.74981E+14
SNOMED	Acute peptic ulcer with haemorrhage	12274003
SNOMED	Chronic duodenal ulcer with perforation	155697004
SNOMED	Barrett ulcer of oesophagus	196609006
SNOMED	Chronic gastrojejunal ulcer NOS	196721003

SNOMED	Acute duodenal ulcer without hemorrhage or perforation but with obstruction	75342000
SNOMED	Acute gastric ulcer without mention of complication	196628004
SNOMED	Cushings ulcer of oesophagus	235614000
SNOMED	Acute gastric ulcer with obstruction (disorder)	196632005
SNOMED	Chronic duodenal ulcer NOS	6.33201E+14
SNOMED	Acute gastric ulcer unspecified	196633000
SNOMED	Acute gastric ulcer without hemorrhage AND without perforation but with obstruction (disorder)	81225008
SNOMED	Stress ulcer of duodenum	235688009
SNOMED	Anti-platelet induced duodenal ulcer	2.08751E+14
SNOMED	Unspecified gastric ulcer	196642007
SNOMED	Parasitic infection causing ulcer of oesophagus	733147004
SNOMED	Infective ulcer of esophagus (disorder)	235609004
SNOMED	Gastric ulcer with haemorrhage AND perforation but without obstruction	2066005
SNOMED	Acute gastrojejunal ulcer with perforation AND obstruction (disorder)	72219001
SNOMED	Gastrojejunal ulcer NOS	196730006
SNOMED	Unspecified duodenal ulcer without mention of complication	196673006
SNOMED	Unspecified gastrojejunal ulcer with unspecified hemorrhage or perforation (disorder)	6.16991E+14
SNOMED	Unspecified duodenal ulcer with unspecified haemorrhage and/or perforation	196678002
SNOMED	Acute gastrojejunal ulcer without haemorrhage AND without perforation but with obstruction	10389003
SNOMED	Peptic ulcer with obstruction	8.49611E+14
SNOMED	Acute gastrojejunal ulcer with obstruction	196712004
SNOMED	Gastrojejunal ulcer with hemorrhage AND obstruction (disorder)	42698006
SNOMED	Chronic duodenal ulcer with hemorrhage and perforation	155698009
SNOMED	Anti-platelet induced duodenal ulcer NOS	2.08741E+14
SNOMED	Acute gastrojejunal ulcer with haemorrhage and perforation	196711006
SNOMED	Childhood duodenal ulcer	43035002
SNOMED	Unspecified gastrojejunal ulcer with perforation	196725007
SNOMED	Chronic gastrojejunal ulcer unspecified	196720002
SNOMED	Unspecified duodenal ulcer with hemorrhage	196674000
SNOMED	Acute peptic ulcer of stomach	235647009
SNOMED	Unspecified gastric ulcer with hemorrhage and perforation	196646005
SNOMED	Ulcer of esophagus due to gastro-esophageal reflux disease without complication	717849006
SNOMED	Ulcerative esophagitis (disorder)	439955006
SNOMED	Duodenal ulcer without haemorrhage, without perforation AND without obstruction	34580000
SNOMED	Acute gastrojejunal ulcer with perforation but without obstruction (disorder)	72395008
SNOMED	Acute peptic ulcer with perforation	196685003
SNOMED	Oesophageal bleeding due to ulcerative oesophagitis	439442003
SNOMED	Acute gastric ulcer unspecified	6.06381E+14
SNOMED	Unspecified gastrojejunal ulcer without mention of complication	196723000
SNOMED	Chronic gastric ulcer with perforation	155687008
SNOMED	Unspecified gastrojejunal ulcer with obstruction	196727004
SNOMED	Ulcer of oesophagus due to disorder of immune system	737190002
SNOMED	Gastroesophageal reflux disease with ulceration	245754007
SNOMED	Chronic peptic ulcer unspecified	6.47841E+14
SNOMED	Chronic peptic ulcer NOS	196696006
SNOMED	Gastric ulcer with obstruction	8.49591E+14
SNOMED	Anti-platelet induced gastric ulcer	2.08731E+14
SNOMED	Chronic gastrojejunal ulcer with perforation	2807004
SNOMED	Acute gastric ulcer with hemorrhage	89748001
SNOMED	Non steroidal anti inflammatory drug induced duodenal ulcer	2.75001E+14
SNOMED	Non steroidal anti inflammatory drug induced gastric ulcer NOS	2.74991E+14
SNOMED	Chronic duodenal ulcer with perforation (disorder)	196664003

SNOMED	Acute gastric ulcer with hemorrhage, with perforation AND with obstruction	53337006
SNOMED	Acute gastric ulcer with perforation	266499002
SNOMED	Acute duodenal ulcer unspecified	196659002
SNOMED	Acute duodenal ulcer with perforation	61347001
SNOMED	Unspecified peptic ulcer with perforation	6.52991E+14
SNOMED	Chronic duodenal ulcer with perforation	49916007
SNOMED	Peptic ulcer with hemorrhage but without obstruction	48658001
SNOMED	Chronic peptic ulcer with perforation (disorder)	155707002
SNOMED	Chronic gastrojejunal ulcer with hemorrhage and perforation and with obstruction	24001002
SNOMED	Chronic gastrojejunal ulcer without mention of complication	196715002
SNOMED	Stress ulcer, NOS	77282005
SNOMED	Chronic gastric ulcer NOS	6.06411E+14
SNOMED	Gastric ulcer, NOS with hemorrhage	15902003
SNOMED	Unspecified gastrojejunal ulcer with perforation	6.06351E+14
SNOMED	Acute gastric ulcer with haemorrhage and perforation	48974009
SNOMED	Peptic anastomotic ulcer (disorder)	717863000
SNOMED	Peptic ulcer of duodenum	235703009
SNOMED	Non steroidal anti inflammatory drug induced duodenal ulcer NOS	2.49341E+14
SNOMED	Duodenal ulcer caused by non-steroidal anti-inflammatory drug (disorder)	722200003
SNOMED	Chronic peptic ulcer with hemorrhage, with perforation AND with obstruction (disorder)	77661009
SNOMED	Gastric ulcer with haemorrhage, with perforation AND with obstruction	17593008
SNOMED	Acute peptic ulcer with haemorrhage, with perforation AND with obstruction	28945005
SNOMED	Chronic duodenal ulcer with haemorrhage AND obstruction	34021006
SNOMED	Drug-induced gastric ulcer	9.22751E+14
SNOMED	Chronic peptic ulcer NOS	155709004
SNOMED	Gastro-esophageal reflux disease with ulceration	196606004
SNOMED	Chemical duodenal ulcer	724532003
SNOMED	Gastric ulcer	6034002
SNOMED	Unspecified gastric ulcer with haemorrhage and perforation	6.06461E+14
SNOMED	Acute duodenal ulcer with perforation AND obstruction (disorder)	62936002
SNOMED	Acute peptic ulcer of duodenum	235687004
SNOMED	Duodenal ulcer caused by Helicobacter pylori and non-steroidal anti-inflammatory agent (disorder)	724530006
SNOMED	Acute gastrojejunal ulcer NOS (disorder)	6.23171E+14
SNOMED	Acute gastrojejunal ulcer with hemorrhage, with perforation and with obstruction (disorder)	58711008
SNOMED	Duodenal ulcer induced by anti-platelet agent (disorder)	423643000
SNOMED	Anti-platelet induced duodenal ulcer	2.05341E+14
SNOMED	Anastomotic ulcer, NOS	57962000
SNOMED	Acute peptic ulcer without mention of complication (disorder)	5.29361E+14
SNOMED	Prepyloric gastric ulcer	22620000
SNOMED	Unspecified gastrojejunal ulcer NOS	196729001
SNOMED	Chronic peptic ulcer with perforation but without obstruction	80953005
SNOMED	Acute peptic ulcer unspecified	5.91731E+14
SNOMED	Chronic duodenal ulcer with obstruction (disorder)	196666001
SNOMED	Exacerbation of peptic ulcer	865912001
SNOMED	Acute peptic ulcer with perforation but without obstruction (disorder)	34921009
SNOMED	Chronic gastric ulcer with haemorrhage and perforation	196638009
SNOMED	Chronic peptic ulcer with perforation (disorder)	196692008
SNOMED	Chronic gastrojejunal ulcer with haemorrhage and perforation	196718000
SNOMED	Acute gastrojejunal ulcer without mention of complication	5.30321E+14
SNOMED	Unspecified gastric ulcer with obstruction	6.06471E+14
SNOMED	Chronic gastric ulcer with hemorrhage, with perforation and with obstruction (disorder)	85787009
SNOMED	Ulcerative gastrointestinal mucositis (disorder)	1.4511E+13

SNOMED	Acute gastrojejunal ulcer without mention of complication	196708005
SNOMED	Acute peptic ulcer NOS	196689009
SNOMED	Gastric ulcer due to Zollinger-Ellison syndrome	717891008
SNOMED	Unspecified gastrojejunal ulcer	6.11571E+14
SNOMED	Unspecified peptic ulcer NOS	196704007
SNOMED	Unspecified peptic ulcer with unspecified hemorrhage and/or perforation	196703001
SNOMED	Gastric ulcer with perforation AND obstruction (disorder)	72486001
SNOMED	Curlings ulcer of duodenum (disorder)	235690005
SNOMED	Unspecified peptic ulcer with perforation	196700003
SNOMED	(Ulcerative esophagitis) or (Barretts esophagus)	196603007
SNOMED	Chronic gastrojejunal ulcer with hemorrhage but without perforation	59356009
SNOMED	Acute duodenal ulcer NOS	196660007
SNOMED	Eosinophilic gastric ulcer	724520002
SNOMED	Chronic peptic ulcer with obstruction (disorder)	196694009
SNOMED	Chronic peptic ulcer without mention of complication	196690000
SNOMED	Chronic peptic ulcer with haemorrhage	196691001
SNOMED	Acute duodenal ulcer unspecified	6.06501E+14
SNOMED	Chronic gastric ulcer	95530000
SNOMED	Gastric peptic ulcer	398177004
SNOMED	Peptic ulcer of stomach (disorder)	235701006
SNOMED	Unspecified peptic ulcer without mention of complication	6.52961E+14
SNOMED	Unspecified peptic ulcer with obstruction	196702006
SNOMED	Peptic ulcer - (PU) site unspecified	266437002
SNOMED	Acute peptic ulcer	196682000
SNOMED	Peptic ulcer - (PU) site unspecified	196681007
SNOMED	Gastric ulcer caused by Helicobacter pylori (disorder)	1.03691E+14
SNOMED	Acute peptic ulcer with haemorrhage but without obstruction	22157005
SNOMED	Acute gastrojejunal ulcer with hemorrhage AND obstruction	72408002
SNOMED	Duodenal ulcer caused by ionizing radiation	724531005
SNOMED	Gastric ulcer caused by ionising radiation	723104008
SNOMED	Acute duodenal ulcer without mention of complication	5.29351E+14
SNOMED	Chronic gastric ulcer with hemorrhage but without obstruction (disorder)	76078009
SNOMED	Gastrojejunal ulcer without hemorrhage AND without perforation (disorder)	2783007
SNOMED	Duodenal ulcer disease (disorder)	196669008
SNOMED	Acute gastrojejunal ulcer with hemorrhage and perforation but without obstruction	66673003
SNOMED	Chronic duodenal ulcer unspecified (disorder)	196667005
SNOMED	Bleeding peptic ulcer	64121000
SNOMED	Chronic duodenal ulcer with haemorrhage and perforation	196665002
SNOMED	Gastrointestinal ulcer	40845000
SNOMED	Unspecified gastric ulcer NOS	196649003
SNOMED	Gastric Cushing ulcer	738791006
SNOMED	Duodenal ulcer	196651004
SNOMED	Gastric ulcer NOS	196650003
SNOMED	Gastrojejunal ulcer (GJU)	16121001
SNOMED	Chronic peptic ulcer with haemorrhage AND with perforation but without obstruction	55746001
SNOMED	Chronic gastric ulcer with perforation (disorder)	31301004
SNOMED	Acute gastrojejunal ulcer with haemorrhage	63954007
SNOMED	Gastric ulcer NOS	6.74151E+14
SNOMED	Combined gastric AND duodenal ulcer	79806007
SNOMED	DU - Perforated duodenal ulcer	88968005
SNOMED	Chronic gastric ulcer unspecified (disorder)	196640004
SNOMED	Chronic gastric ulcer without mention of complication	196635007

SNOMED	Gastric ulcer due to nonsteroidal antiinflammatory drug in therapeutic use	1.29141E+14
SNOMED	Unspecified duodenal ulcer with perforation	196675004
SNOMED	Radiation ulcer of esophagus (disorder)	235613006
SNOMED	Unspecified duodenal ulcer with perforation	5.91671E+14
SNOMED	Lymphocytic duodenal ulcer	717879004
SNOMED	Cushing's ulcer of stomach	23649000
SNOMED	Acute gastric ulcer with perforation (disorder)	196630002
SNOMED	Chronic duodenal ulcer with hemorrhage, with perforation AND with obstruction (disorder)	86258000
SNOMED	Peptic ulcer with perforation but without obstruction	12625009
SNOMED	Acute duodenal ulcer with obstruction	196658005
SNOMED	(Ulcerative oesophagitis) or (Barrett's oesophagus)	302914006
SNOMED	Peptic ulcer, NOS with perforation	88169003
SNOMED	Unspecified peptic ulcer with hemorrhage	196699004
SNOMED	Gastrojejunal ulcer NOS	6.17011E+14
SNOMED	Chronic duodenal ulcer with hemorrhage (disorder)	89469000
SNOMED	Acute gastric ulcer NOS (disorder)	6.06391E+14
SNOMED	Perforated DU (& [acute])	155693000
SNOMED	Unspecified gastrojejunal ulcer with haemorrhage	196724006
SNOMED	Unspecified gastrojejunal ulcer with obstruction (disorder)	6.16971E+14
SNOMED	Giant duodenal ulcer	68834009
SNOMED	Gastric ulcer with perforation	9829001
SNOMED	Peptic ulcer NOS	155710009
SNOMED	Gastrojejunal ulcer with haemorrhage, with perforation AND with obstruction	54798007
SNOMED	Chronic gastric ulcer with perforation but without obstruction (disorder)	36246001
SNOMED	Acute gastric ulcer with hemorrhage AND with perforation but without obstruction	17067009
SNOMED	Anti-platelet induced duodenal ulcer NOS (disorder)	2.06451E+14
SNOMED	Duodenal ulcer due to Zollinger-Ellison syndrome	717892001
SNOMED	Chronic gastrojejunal ulcer NOS	6.23191E+14
SNOMED	Peptic ulcer with hemorrhage AND obstruction (disorder)	64398008
SNOMED	Duodenal ulcer NOS (disorder)	155700000
SNOMED	Duodenal ulcer caused by bacterium (disorder)	723884008
SNOMED	Peptic ulcer with hemorrhage, with perforation AND with obstruction (disorder)	26221006
SNOMED	Chronic peptic ulcer without hemorrhage or perforation and without obstruction	60400003
SNOMED	Unspecified duodenal ulcer without mention of complication (disorder)	6.12761E+14
SNOMED	Gastric ulcer NOS	266436006
SNOMED	Acute gastrojejunal ulcer unspecified	6.23161E+14
SNOMED	Perforated GU (& [acute]) (disorder)	155683007
SNOMED	Bacterial ulcer of esophagus (disorder)	235610009
SNOMED	Gastric ulcer	155681009
SNOMED	Chronic peptic ulcer without hemorrhage AND without perforation but with obstruction	12384004
SNOMED	Jejunal ulcer (disorder)	54822007
SNOMED	Gastrojejunal ulcer without hemorrhage AND without perforation but with obstruction	47152002
SNOMED	Anastomotic ulcer (disorder)	281678002
SNOMED	Gastroduodenal ulcer	13200003
SNOMED	Unspecified peptic ulcer with unspecified haemorrhage and/or perforation	6.23151E+14
SNOMED	Ulcer of esophagus due to allergic disorder (disorder)	737189006
SNOMED	Chronic peptic ulcer unspecified	196695005
SNOMED	Gastric ulcer without haemorrhage, without perforation AND without obstruction	59913009
SNOMED	Postbulbar duodenal ulcer	78054007
SNOMED	Acute gastric ulcer with perforation	19850005
SNOMED	Ulcer of esophagus caused by thermal agent (disorder)	724516001
SNOMED	Chronic peptic ulcer with hemorrhage and perforation	196693003

SNOMED	Duodenal ulcer without hemorrhage AND without perforation	56776001
SNOMED	Unspecified peptic ulcer NOS (disorder)	6.30931E+14
SNOMED	Acute duodenal ulcer with haemorrhage	196654007
SNOMED	Acute peptic ulcer without hemorrhage and perforation and without obstruction	3023008
SNOMED	Drug-induced peptic ulcer (disorder)	109813002
SNOMED	Acute gastric ulcer with perforation and obstruction	43694004
SNOMED	Acute duodenal ulcer with perforation	266500006
SNOMED	Acute peptic ulcer with perforation AND obstruction	35681000
SNOMED	Duodenal ulcer caused by platelet aggregation inhibitor (disorder)	840591006
SNOMED	Chronic duodenal ulcer with hemorrhage [dup] (disorder)	196663009
SNOMED	Chronic duodenal ulcer NOS	196668000
SNOMED	Duodenal ulcer, NOS with perforation but without obstruction	86983005
SNOMED	Acute gastric ulcer without mention of complication	5.29271E+14
SNOMED	Unspecified gastric ulcer with haemorrhage	196644008
SNOMED	Unspecified duodenal ulcer with unspecified haemorrhage and/or perforation	5.91691E+14
SNOMED	Chronic duodenal ulcer without haemorrhage, without perforation AND without obstruction	57940000
SNOMED	Chronic gastrojejunal ulcer with hemorrhage	196716001
Peripheral artery disease		
SNOMED	Endovascular stenting of suprarenal aortic aneurysm	4.88711E+14
SNOMED	Occlusion of anterior tibial artery	8.42731E+14
SNOMED	Carotid artery atherosclerosis	300920004
SNOMED	Intracranial bypass from carotid artery NEC	2.33151E+14
SNOMED	Other specified transluminal operation on carotid artery	5.59681E+14
SNOMED	Percutaneous transluminal balloon angioplasty of artery	2.60601E+14
SNOMED	Ischaemic foot	5.10171E+14
SNOMED	Claudication (finding)	275520000
SNOMED	Other specified peripheral vascular disease	6.44041E+14
SNOMED	Transluminal operation on carotid artery NOS	5.82981E+14
SNOMED	Percutaneous transluminal insertion of stent into vein NEC	2.90111E+14
SNOMED	Endovascular stenting for aortic aneurysm of bifurcation NEC	2.89971E+14
SNOMED	[X]Occlusion and stenosis of other precerebral arteries	4.19711E+14
SNOMED	Other specified peripheral vascular disease NOS	6.35831E+14
SNOMED	Aortic aneurysm without mention of rupture NOS	6.00621E+14
SNOMED	[X]Aortic aneurysm of unspecified site, ruptured	4.09791E+14
SNOMED	Percutaneous balloon angioplasty of vein	2.61241E+14
SNOMED	Aortic aneurysm monitoring	8.02941E+14
SNOMED	Percutaneous transluminal venoplasty	2.61231E+14
SNOMED	Insertion of stent into vena cava NEC	4.88771E+14
SNOMED	Peripheral vascular disease	8.55911E+14
SNOMED	H/O intermittent claudication	8.37591E+14
SNOMED	Arteriosclerotic vascular disease NOS	6.60711E+14
SNOMED	Transluminal aortic stent graft with fenestration NEC	2.84121E+14
SNOMED	Aneurysm of suprarenal aorta	7.56281E+14
SNOMED	Aortic aneurysm screening abnormal	7.54751E+14
SNOMED	HAVS - Hand-arm vibration syndrome	234034005
SNOMED	Insertion of stent into vena cava NEC	2.25491E+14
SNOMED	Peripheral ischemia (disorder)	233958001
SNOMED	Intracranial bypass from carotid artery NEC	2.60421E+14
SNOMED	Replacement of aneurysmal bifurcation of aorta NOS	5.93421E+14
SNOMED	Percutaneous transluminal venoplasty	2.25261E+14
SNOMED	Transluminal aortic stent graft with fenestration NEC	2.89941E+14
SNOMED	Endovascular insertion of stent for thoracic aortic aneurysm	4.88721E+14

SNOMED	Intermittent claudication (finding)	63491006
SNOMED	Peripheral vascular disease NOS	6.46351E+14
SNOMED	Endovascular stenting of suprarenal aortic aneurysm	2.25351E+14
SNOMED	[X]Aortic aneurysm of unspecified site, nonruptured	4.69871E+14
SNOMED	Endovascular repair of carotid artery	2.25071E+14
SNOMED	Aortic aneurysm NOS	6.60741E+14
SNOMED	Endovascular stenting of suprarenal aortic aneurysm	2.60331E+14
SNOMED	Occlusion of posterior tibial artery	8.42751E+14
SNOMED	Percutaneous balloon angioplasty of arteriovenous fistula	8.76311E+14
SNOMED	Aortic atherosclerosis	81817003
SNOMED	History of peripheral vascular disease	7.11321E+14
SNOMED	Profundoplasty of femoral artery NEC	5.95541E+14
SNOMED	Occlusion of dorsalis pedis artery	8.42141E+14
SNOMED	Percutaneous coronary intervention	8.41991E+14
SNOMED	Emergency percutaneous coronary intervention	8.90631E+14
SNOMED	Percutaneous transluminal angioplasty of aorta NEC	5.85751E+14
SNOMED	Endarterectomy of carotid artery NEC	5.89701E+14
SNOMED	Emergency percutaneous coronary intervention	8.81331E+14
SNOMED	Precerebral artery occlusion NOS	5.84161E+14
SNOMED	Angioplasty of femoral artery	7.6981E+13
SNOMED	Raynaud's syndrome NOS	6.00661E+14
SNOMED	Percutaneous transluminal insertion of stent into vein NEC	2.81381E+14
SNOMED	Transjugular intrahepatic angioplasty of portal vein	2.58021E+14
SNOMED	Abdominal aortic aneurysm without mention of rupture	5.30151E+14
SNOMED	Plastic repair of aorta and insertion of tube graft	2.60261E+14
SNOMED	Peripheral arterial occlusive disease (disorder)	399957001
SNOMED	Peripheral vascular disease monitoring invitation	3.76601E+14
SNOMED	Endovascular insertion of stent for thoracic aortic aneurysm	3.78491E+14
SNOMED	Ruptured aortic aneurysm NOS	6.60721E+14
SNOMED	Percutaneous transluminal insertion of stent in vein	2.25251E+14
SNOMED	Percutaneous transluminal angioplasty of vein NEC	2.24961E+14
SNOMED	Other peripheral vascular disease	6.82631E+14
SNOMED	Coronary angioplasty planned	7.52181E+14
SNOMED	[X]Other specified peripheral vascular diseases	4.00701E+14
SNOMED	Percutaneous balloon angioplasty of pulmonary vein	2.61401E+14
SNOMED	Single anastomosis of mammary artery to coronary artery NEC	6.91621E+14
SNOMED	Endovascular stenting of aorto-uniiliac aneurysm	2.89981E+14
SNOMED	Other specified reconstruction of carotid artery	5.45591E+14
SNOMED	Transluminal aortic branched stent graft NEC	3.78461E+14
SNOMED	Transluminal aortic branched stent graft NEC	4.88691E+14
SNOMED	Peripheral vascular disease (disorder)	400047006
SNOMED	Percutaneous transluminal angioplasty of artery NEC	5.86111E+14
SNOMED	Neurogenic claudication	5.10401E+14
SNOMED	Percutaneous transluminal balloon angioplasty of artery	2.25181E+14
SNOMED	Bypass to carotid artery NEC	5.53191E+14
SNOMED	Reconstruction of carotid artery NOS	5.45601E+14
SNOMED	Percutaneous transluminal angioplasty of vein NEC	2.61891E+14
SNOMED	Occlusion of dorsalis pedis artery	8.42151E+14
SNOMED	Infrarenal abdominal aortic aneurysm	7.15571E+14
SNOMED	Endovascular stenting for aortic aneurysm of bifurcation NEC	2.83271E+14
SNOMED	Infrarenal abdominal aortic aneurysm	7.56641E+14
SNOMED	Thoracoabdominal aortic aneurysm, without mention of rupture	5.30161E+14

SNOMED	Percutaneous angioplasty of pulmonary artery	2.32591E+14
SNOMED	Transjugular intrahepatic angioplasty of portal vein	2.39001E+14
SNOMED	HAVS - Hand-arm vibration syndrome	8.90271E+14
SNOMED	Ischaemic lower limb pain at rest (finding)	8.36711E+14
SNOMED	Profundoplasty of popliteal artery NEC	5.95551E+14
SNOMED	Lower limb ischemia (disorder)	233961000
SNOMED	Occlusion of artery of lower limb	8.42131E+14
SNOMED	Percutaneous transluminal stent reconstruction of vein	2.61901E+14
SNOMED	Occlusion of posterior tibial artery	8.42741E+14
SNOMED	Carotid artery atherosclerosis	9.4471E+13
SNOMED	Percutaneous transluminal insertion of stent in vein	2.61221E+14
SNOMED	Other open operations on carotid artery	5.45611E+14
SNOMED	Peripheral vascular disease monitoring invitation	3.76611E+14
SNOMED	Peripheral vascular disease monitoring invitation	3.76591E+14
SNOMED	Patch angioplasty of renal artery	2.60471E+14
SNOMED	Patch angioplasty of renal artery	2.25201E+14
SNOMED	Occlusion of artery of lower limb	8.42121E+14
SNOMED	Endovascular stenting of aorto-uniiliac aneurysm	2.60371E+14
SNOMED	Thromboangiitis obliterans NOS	6.44031E+14
SNOMED	Aneurysm of suprarenal aorta	7.58061E+14
SNOMED	Aortic aneurysm monitoring	8.02951E+14
SNOMED	Plastic repair of aorta and insertion of tube graft	2.23551E+14
SNOMED	Other precerebral artery occlusion	5.84151E+14
SNOMED	Percutaneous coronary intervention	8.41981E+14
SNOMED	Coronary angioplasty planned	7.52191E+14
SNOMED	Acroparaesthesia - unspecified	6.00691E+14
SNOMED	Percutaneous transluminal insertion of stent into aorta	2.89961E+14
SNOMED	Insertion of drug-eluting coronary artery stent	2.11371E+14
SNOMED	Percutaneous transluminal stent reconstruction of vein	2.25381E+14
SNOMED	[X]Peripheral angiopathy in diseases classified elsewhere	4.32021E+14
SNOMED	Endovascular repair of carotid artery	2.60431E+14
SNOMED	Endovascular stenting of aorto-uniiliac aneurysm	2.25041E+14
SNOMED	Percutaneous transluminal angioplasty of coeliac artery NEC	5.54761E+14
SNOMED	Repair of carotid artery NEC	5.46541E+14
SNOMED	Percutaneous transluminal insertion of stent into aorta	2.85071E+14
SNOMED	Peripheral angiopathic disease EC NOS	6.46531E+14
SNOMED	Endovascular stenting of aorto-uniiliac aneurysm	4.88751E+14
SNOMED	Occlusion of anterior tibial artery	8.42721E+14
SNOMED	Heart - arterial implant NOS	6.39231E+14
SNOMED	Insertion of drug-eluting coronary artery stent	2.03741E+14
Read	Percutaneous transluminal angioplasty of blood vessel liver	78083
Read	Placement of stent in ductus arteriosus	X012N
Read	Percutaneous transluminal angioplasty of renal artery	7A320
Read	Perc translumin balloon angioplasty stenting coronary artery	XaLgU
Read	LIMA sequential anastomosis	XM1M3
Read	LIMA single anastomosis	XM1LR
Read	Coronary A by-pass anastam NOS	XE2r7
Read	Emergency percutaneous coronary intervention	XaaUg
Read	Perc transl ins stent into maj system pulmon collater artery	7A0C5
Read	Intermittent claudication	XE0VR
Read	Thromboangiitis obliterans	G731.
Read	Angioplasty of superior vena cava	X00yJ

Read	Cerebral atherosclerosis	XE0VL
Read	Carotid artery occlusion	XE0VH
Read	Basilar artery occlusion	G630.
Read	Perc transluminal cutting balloon angioplasty pulmonary vein	XaLjG
Read	Emerg repl aneurysm bifurc aorta by anast aorta to iliac a	7A112
Read	Emerg repl aneurysm bifurc aorta by anast aorta to iliac a	XE0Ey
Read	Patch repair of coeliac artery	Xa9Ix
Read	Upper limb ischaemia	X203R
Read	Other open operations on carotid artery	7A21.
Read	Percutaneous coronary intervention	XaZSy
Read	Ischaemic lower limb pain at rest	XaZJa
Read	Percutaneous transluminal insertion of stent cerebral artery	XaMMj
Read	Intracranial bypass from carotid artery NEC	7A207
Read	Percutaneous transluminal insertion of stent into aorta	XaMvB
Read	Transjugular intrahepatic angioplasty of portal vein	780D0
Read	Endovas insertion of stent graft for aorto-uniiliac aneurysm	XaMmK
Read	History of peripheral vascular disease	XaVyB
Read	Perc transluminal insertion stent superior mesenteric artery	XaQIl
Read	Occlusion of dorsalis pedis artery	G7840
Read	Coarctation angioplasty	XaBYQ
Read	Endov insertion of stent graft for thoracic aortic aneurysm	XaMmJ
Read	Occlusion of posterior tibial artery	G7842
Read	Claudication	XM1Qu
Read	Insertion of popliteal artery stent	X016q
Read	Aortic aneurysm repair	X015i
Read	Placement of stent in pulmonary artery	X012Z
Read	Plastic repair of aorta and insertion of tube graft	XaLh9
Read	Patch repair of subclavian artery	XaCLU
Read	Percutaneous transluminal angioplasty of coeliac artery NEC	7A350
Read	High-flow inter extrac intrac byp ext carot art mid cer art	XaLhN
Read	Replace aneurysm abdominal aorta by anast aorta to aorta NEC	7A144
Read	Perc translum insert stent visceral branch abdominal aor NEC	XaMMl
Read	Endov ins stent graft for aortic dissection in any position	XaMmL
Read	Percutaneous transluminal angioplasty of axillary artery	7A28C
Read	Patch repair of femoral artery	XaCLV
Read	Transluminal aortic stent graft with fenestration NEC	XaMt7
Read	Endovas insert of stent graft for suprarenal aortic aneurysm	XaMmI
Read	Percutaneous balloon angioplasty of intracranial artery	X00Hc
Read	Patch repair of superior mesenteric artery	Xa9Iz
Read	Profundoplasty femoral artery & patch repair deep fem artery	7A494
Read	Transjugular intrahepatic ins stent graft into portal vein	XaMlb
Read	Byp carot art anastom superfic tempor artery middle cere art	7A206
Read	Insertion of drug-eluting coronary artery stent	XaLSD
Read	Abdominal aortic aneurysm which has ruptured	G713.
Read	Perc translum insert stent visceral branch abdominal aor NEC	7A35D
Read	Neurogenic claudication	N14A.
Read	Placement of stent in major aortopulmonary collateral artery	X010a
Read	Endovascular stenting of aorto-uniiliac aneurysm	XaLhK
Read	Patch angioplasty of renal artery	XaLhR
Read	Replacement of carotid artery using graft	7A200
Read	Ischaemic hand	XaB5W
Read	Percut translum cutting balloon angioplasty pulmonary artery	7A0A3

Read	Aortic atherosclerosis	XE0VO
Read	Insertion of stent in superior vena cava	X00yK
Read	Percutaneous balloon angioplasty of profunda femoris	X013t
Read	Percutaneous balloon angioplasty of artery	Xa0EZ
Read	Endovascul insert stent infrarenal abdominal aortic aneurysm	XaLhF
Read	Angioplasty of coronary artery	X00tS
Read	Percutaneous transluminal aortic stent graft branches NEC	XaLhD
Read	Percutaneous transluminal insertion of stent in vein	XaLia
Read	OS perc translumina balloon angioplast stenting coronary art	XaLgZ
Read	Percutaneous transluminal balloon angioplasty stenting aorta	XaLhB
Read	Atherosclerosis	G70..
Read	Percutaneous transluminal angioplasty of iliac artery	7A440
Read	Percutaneous transluminal insertion stent carotid artery	XaMI9
Read	Transluminal operations on carotid artery	7A22.
Read	Angioplasty of external iliac artery	X013m
Read	Ruptured suprarenal aortic aneurysm	G7130
Read	Percutaneous high speed rotational coronary atherectomy	X00tR
Read	Percutaneous transluminal insertion stent hepatic artery NEC	XaLe6
Read	Patch repair of brachial artery	Xa9Iv
Read	Transjugular intrahepatic angioplasty of portal vein	XaLdv
Read	Percutaneous transluminal angioplasty of blood vessel liver	XaLdm
Read	Percutaneous transluminal balloon angioplasty pulmonary art	XaLgv
Read	Coronary angioplasty planned	8L41.
Read	Angioplasty of coeliac artery	X013d
Read	Percutaneous transluminal insertion stent into renal artery	XaMmR
Read	Insertion of thoracic aorta stent	X016l
Read	Open insertion of iliac artery stent	XaDmh
Read	Percutaneous transluminal insertion stent carotid artery	7A223
Read	Patch repair of common iliac artery	XaCLX
Read	Other specified reconstruction of carotid artery	7A20y
Read	Angioplasty of subclavian artery	X013S
Read	Coronary artery bypass grafts x 3	X00tI
Read	Byp carot art anastom superfic tempor artery middle cere art	XaLhO
Read	Angioplasty of renal artery	X013h
Read	Aortocoronary bypass grafting	XaC2Q
Read	Angioplasty of anterior tibial artery	XaDzI
Read	Abdominal aortic aneurysm stenting	XaBD1
Read	Angioplasty of common iliac artery	X013i
Read	[X]Other specified peripheral vascular diseases	Gyu74
Read	H/O: Peripheral vascular disease procedure	XaBL7
Read	Insertion of stent in pulmonary vein	X00y9
Read	Prosthetic patch to artery	Xa3m7
Read	Percutaneous transluminal stent reconstruction of vein	7A6S1
Read	Neurogenic claudication	Xa9Fy
Read	Ischaemic foot	Xa84U
Read	Angioplasty of vertebral artery	Xa7y2
Read	Percutan transluminal insertion stent graft hepatic artery	XaLe5
Read	Lower limb ischaemia	X203T
Read	Angioplasty of profunda femoris artery	X013s
Read	Ruptured aortic aneurysm NOS	G715.
Read	Balloon dilatation of ductus arteriosus	X012M
Read	Bypass to carotid artery NEC	7A202

Read	Percutaneous balloon angioplasty of crural artery	X013w
Read	Intracranial bypass from carotid artery NEC	XaMMy
Read	Percutaneous transluminal insertion of stent in vein	7A6E5
Read	Single anastomosis of mammary artery to coronary artery NEC	XE2Px
Read	Perc transl ins stent into maj system pulmon collater artery	XaLh6
Read	Patch repair of carotid artery	Xa9Ir
Read	Percutaneous transluminal angioplasty of vein NEC	7A6S0
Read	Insertion of stent into iliac vein	X017R
Read	Claudication distance	X77Ut
Read	Percut transluminal balloon angioplasty stenting pulmon art	XaLgw
Read	Ureteroscopic balloon rupture and stenting	X30Dw
Read	Endovas insert stent for aortic aneurysm of bifurcation NEC	XaMrD
Read	Balloon dilatation of conduit	X011S
Read	Percutan transluminal aortic stent graft fenestration NEC	XaLhC
Read	Percutaneous transluminal insertion of iliac artery stent	XaDmi
Read	Percut transluminal insertion of stent into pulmonary vein	XaMmU
Read	Endovas insertion of stent graft for aortic bifurcation NEC	XaMmM
Read	Acroparaesthesia - Nothnagel's type	G73y5
Read	[X]Aortic aneurysm of unspecified site, nonruptured	Gyu72
Read	Occlusion of anterior tibial artery	G7841
Read	Aortic aneurysm screening abnormal	68B51
Read	Other precerebral artery occlusion	G63y.
Read	Transjugular intrahepatic ins stent graft into portal vein	780G1
Read	Patch repair of popliteal artery	XaCLR
Read	Percutan transluminal insertion stent graft hepatic artery	7.80E+02
Read	Patch repair of axillary artery	XaCLY
Read	Percutaneous transluminal insertion of stent into vein NEC	XaMmT
Read	Endovas insertion of stent graft for aorto-uniiliac aneurysm	7A1C5
Read	Percutaneous balloon angioplasty of pulmonary vein	Xa0F6
Read	Endovascular insertion stent for suprarenal aortic aneurysm	7A1B9
Read	[X]Occlusion and stenosis of other precerebral arteries	Gyu65
Read	Percutan transluminal aortic stent graft fenestration NEC	7A1A6
Read	Aortic aneurysm monitoring	66f3.
Read	Percutaneous balloon angioplasty of arteriovenous fistula	Xa0FB
Read	Profundoplasty	Xa8Ow
Read	Angioplasty of brachial artery	X013T
Read	Insertion of external iliac artery stent	X016o
Read	Endovascular stenting of aorto-uniiliac aneurysm	7A1B5
Read	Replace aneurysm abdominal aorta by anast aorta to aorta NEC	XE0F4
Read	Peripheral ischaemia	X203Q
Read	Patch repair of artery	X016E
Read	Percut translum cutting balloon angioplasty coronary artery	XaMKE
Read	Thoracoabdominal aortic aneurysm, ruptured	G7150
Read	Patch repair of adrenal artery	XaCLZ
Read	Peripheral vascular disease NOS	G73z.
Read	Coronary artery bypass grafts x 2	X00tH
Read	Percutaneous transluminal stent reconstruction of vein	XaLiu
Read	Angioplasty of inferior vena cava	X00yR
Read	Percutaneous balloon angioplasty of abdominal aorta	X013a
Read	Endov insertion of stent graft for thoracic aortic aneurysm	7A1C2
Read	Endovascular stenting for aortic aneurysm of bifurcation NEC	7A1B6
Read	Endovascular ins stent for aortic dissection in any position	XaLhI

Read	Percutaneous transluminal angioplasty of vertebral artery	7A282
Read	Juxtarenal aortic aneurysm	XaEef
Read	Endovascular repair of carotid artery	XaLhP
Read	Percutaneous transluminal insertion of iliac artery stent	7A444
Read	Endovascular insertion stent for suprarenal aortic aneurysm	XaLhG
Read	Percutaneous balloon angioplasty of vein	Xa0F4
Read	Claudication distance	16I..
Read	Spinal claudication	X70Cp
Read	Replacement of aneurysmal bifurcation of aorta OS	7A11y
Read	Erythromelalgia	G73y8
Read	Percutaneous transluminal stent implantation arterial duct	7A021
Read	Patch repair of coronary artery	X00tY
Read	Infrarenal abdominal aortic aneurysm	XaW51
Read	Gangrene of thumb	G7323
Read	Percutaneous transluminal balloon angioplasty of artery	XaLhc
Read	Perc transluminal insertion stent superior mesenteric artery	7A35E
Read	Insertion of stent into vena cava NEC	7A637
Read	Heart internal mammary artery implant	Xa3ks
Read	Spasm of peripheral artery	G73z1
Read	Erythrocyanosis	G73y7
Read	Occlusion of posterior tibial artery	XaZV1
Read	Angioplasty of vein	X0172
Read	Reconstruction of carotid artery NOS	7A20z
Read	Emerg repl aneurysm bifurc aorta by anast aorta to fem art	7A110
Read	Heart - arterial implant NOS	Xa3kl
Read	Percut trans balloon angiopl major systemic pulmon coll art	XaLh5
Read	Coronary artery bypass grafting	X00tE
Read	Perc transluminal cutting balloon angioplasty pulmonary vein	7A6K2
Read	Percutaneous transluminal angioplasty of artery NEC	7A540
Read	Endovas insert of stent graft for suprarenal aortic aneurysm	7A1C1
Read	Endarterectomy and patch repair of carotid artery	7A203
Read	Profundoplasty of femoral artery NEC	7A496
Read	Angioplasty of internal iliac artery	X013k
Read	Angioplasty of systemic venous pathway	X00yY
Read	Insertion abdominal aorta stent	X016m
Read	Gangrene of finger	G7322
Read	Transluminal operation on carotid artery NOS	7A22z
Read	Percutaneous balloon angioplasty of pulmonary venous pathway	Xa0F9
Read	Percutaneous balloon angioplasty extracranial carotid artery	X013R
Read	Repair of carotid artery NEC	7A210
Read	H/O: Peripheral vascular disease procedure	14NB.
Read	Percutaneous balloon angioplasty of arteriovenous fistula	7A607
Read	Transluminal aortic branched stent graft NEC	XaMt8
Read	Ischaemic finger	XaB5d
Read	Percutaneous transluminal balloon dilation cardiac conduit	XaLgc
Read	Percutan transluminal insertion of stent graft portal vein	7.80E+06
Read	Percutan transluminal insertion of stent graft portal vein	XaMlW
Read	Angioplasty of artery	X013N
Read	Rotary blade angioplasty	7A545
Read	Percutaneous transluminal venoplasty	7A6E6
Read	Percutaneous transluminal balloon angioplasty pulmonary art	7A0A4
Read	Peripheral vascular disease (& [NOS])	XE0XC

Read	Coronary angioplasty planned	XaX1p
Read	Percut transluminal insertion of stent into pulmonary artery	7A0A7
Read	Percutaneous balloon angioplasty of systemic venous pathway	Xa0FA
Read	Percut transluminal balloon angioplasty stenting pulmon art	7A0A5
Read	Replacement of aneurysmal bifurcation of aorta	7A11.
Read	Endarterectomy of carotid artery NEC	7A204
Read	Intermittent claudication	G73z0
Read	Insertion of stent into vena cava NEC	XaLiL
Read	Perc translum balloon angioplasty stenting coronary art NOS	XaLga
Read	Replacement of aneurysmal bifurcation of aorta NOS	7A11z
Read	Occlusion of dorsalis pedis artery	XaZT6
Read	Perc tran ball angio ins 3 or more drug elut stents cor art	XaLgW
Read	Angioplasty of external carotid artery	X013Q
Read	Percutaneous cor balloon angiop 3 more stents cor art NEC	XaLgY
Read	Replace aneurysm bifurc aorta by anast aorta to iliac artery	XE0Ez
Read	Percutaneous transluminal insertion of stent into aorta	7A1AA
Read	Percutaneous transluminal balloon angioplasty stenting aorta	7A1A5
Read	Percutan transluminal balloon angioplasty stenting renal art	7A324
Read	Percutaneous transluminal aortic stent graft branches NEC	7A1A7
Read	Transluminal aortic stent graft with fenestration NEC	7A1A8
Read	Transjugular intrahepatic insertion stent into portal vein	780G0
Read	Emerg replace aneurysm abdom aorta by anast aorta/aorta NEC	7A134
Read	HAVS - Hand-arm vibration syndrome	G735.
Read	Perc translum balloon angioplasty insert 1-2 stents cor art	XaLgX
Read	Peripheral angiopathic disease EC NOS	G73y1
Read	Other specified peripheral vascular disease	G73y.
Read	Percutaneous balloon angioplasty of common iliac artery	X013j
Read	Plastic repair of aorta using subclavian flap	7A181
Read	Endovascular stenting infrarenal abdominal aortic aneurysm	7A1B0
Read	DNA - Did not attend peripheral vascular disease c	9N4h.
Read	Endovas insert stent for aortic aneurysm of bifurcation NEC	7A1BC
Read	Replace aneurysm bifurc aorta by anast aorta to femoral art	7A111
Read	Emerg replace aneurysm abdom aorta by anast aorta/aorta NEC	XE0F2
Read	Peripheral vascular disease NOS	G73zz
Read	Endov ins stent graft for aortic dissection in any position	7A1C3
Read	Perc translum ball angio insert 1-2 drug elut stents cor art	XaLgV
Read	Endovascular stenting of suprarenal aortic aneurysm	7A1B1
Read	OS translum ins stent graft for aneurysmal segment of aorta	7A1Cy
Read	Peripheral vascular disease	Xa0lV
Read	Profundoplasty of popliteal artery NEC	7A497
Read	Percutaneous transluminal stent implantation arterial duct	XaLgn
Read	Percut transluminal balloon angioplasty stent pulmonary vein	7A6K3
Read	Insertion of stent in systemic venous pathway	X00yZ
Read	Mechanical complication of carotid artery bypass	SP012
Read	Cerebral atherosclerosis	G670.
Read	Ischaemic toe	XaB5X
Read	Angioplasty of posterior tibial artery	XaDzJ
Read	Peripheral vascular disease monitoring invitation	9m1..
Read	Percutaneous transluminal balloon angioplasty of artery	7A564
Read	Endovascular repair of carotid artery	7A222
Read	Percutaneous transluminal insertion of stent femoral artery	XaMNe
Read	Endovas ins stent graft for infrarenal abdom aortic aneurysm	7A1C0

Read	Coronary artery bypass grafts x 4	X00tJ
Read	Raynaud's phenomenon	XE0VQ
Read	Raynaud's syndrome NOS	G730z
Read	Percutaneous transluminal balloon dilation cardiac conduit	793H0
Read	Percutan transluminal balloon angioplasty stenting renal art	XaLhS
Read	Percut translum cutting balloon angioplasty pulmonary artery	XaLgu
Read	Percutaneous balloon angioplasty of external iliac artery	X013n
Read	Trash foot	XaB5V
Read	Occlusion of artery of lower limb	G784.
Read	Carotid artery atherosclerosis	G70y0
Read	Raynaud's disease	G7300
Read	Patch repair of vertebral artery	XaCLS
Read	Presenile gangrene	G7311
Read	Peripheral vascular disease monitoring invitation	XaPno
Read	OS translum ins stent graft for aneurysmal segment of aorta	XaMmN
Read	Insertion of stent into vein	X017M
Read	Ruptured suprarenal aortic aneurysm	XaEed
Read	Angioplasty of superior mesenteric artery	X013f
Read	Angioplasty of pulmonary vein	X00yB
Read	Raynaud's phenomenon	G7301
Read	RIMA sequential anastomosis	XM1M4
Read	Percutaneous transluminal insertion of stent portal vein	7.80E+05
Read	Insertion of iliac artery stent	7A443
Read	Infrarenal abdominal aortic aneurysm	G7142
Read	Aortic aneurysm repair	7A14.
Read	Other specified transluminal operation on carotid artery	7A22y
Read	High-flow inter extrac intrac byp ext carot art mid cer art	7A205
Read	Acrocyanosis	G73y2
Read	Patch repair of arterial graft	X016H
Read	Emergency repair of aortic aneurysm	7A13.
Read	Gangrene of foot	G7321
Read	Percutaneous transluminal angioplasty of popliteal artery	7A4B1
Read	Percutaneous transluminal angioplasty of aorta NEC	7A1A1
Read	H/O intermittent claudication	XaZLS
Read	Percutaneous transluminal insertion stent subclavian art	XaMMk
Read	Aneurysm of suprarenal aorta	G7143
Read	Percutaneous transluminal angioplasty of carotid artery	7A220
Read	Angioplasty of thoracic aorta	X013X
Read	Insertion of stent into subclavian vein	X017Q
Read	Plastic repair of aorta and insertion of tube graft	7A185
Read	Critical lower limb ischaemia	X203U
Read	Aortic aneurysm NOS	G71z.
Read	Percutaneous balloon angioplasty of common femoral artery	X013p
Read	Plastic repair of aorta using patch graft	7A182
Read	Angioplasty of crural artery	X013v
Read	Percutaneous transluminal venoplasty	XaLib
Read	Patch repair of pulmonary artery	XaCIC
Read	Thoracoabdominal aortic aneurysm, without mention of rupture	G7160
Read	Peripheral arterial disease	G734.
Read	Percutaneous transluminal angioplasty of femoral artery	7A4B0
Read	Aortic aneurysm monitoring	XaYMz
Read	Precerebral artery occlusion NOS	G63z.

Read	Raynaud's syndrome	G730.
Read	Occlusion/stenosis cerebral arts not result cerebral infarct	G677.
Read	Patch repair of iliac artery	XaCLW
Read	Percutaneous angioplasty of pulmonary artery	XaLsb
Read	Extremity artery atheroma	G702.
Read	Arteriosclerotic vascular disease NOS	G70z.
Read	Percutaneous transluminal balloon angioplasty of aorta	7A1A0
Read	Abdominal aortic aneurysm without mention of rupture	G714.
Read	RIMA single anastomosis	XM1LS
Read	Endovas insertion of stent graft for aortic bifurcation NEC	7A1C4
Read	Endovascular insertion of stent for thoracic aortic aneurysm	7A1BA
Read	Translum ins stent graft for aneurysmal segment of aorta NOS	XaMmO
Read	Insertion of renal artery stent	XaJsc
Read	Leaking abdominal aortic aneurysm	G718.
Read	Acroparaesthesia - unspecified	G73y6
Read	Angioplasty of pulmonary artery	X012Y
Read	Occlusion of anterior tibial artery	XaZV0
Read	Open insertion of iliac artery stent	7A433
Read	Profundoplasty and patch repair of popliteal artery	7A495
Read	Percutaneous balloon angioplasty of thoracic aorta	X013Y
Read	Vein patch repair of artery	XaBVH
Read	Percutaneous transluminal insertion of stent into vein NEC	7A6E8
Read	Percut transluminal balloon angioplasty pulmonary artery NEC	XaMrC
Read	Insertion common iliac artery stent	X016n
Read	Peripheral gangrene	G732.
Read	Transjugular intrahepatic insertion stent into portal vein	XaMla
Read	Insertion superficial femoral artery stent	X016p
Read	Gangrene of toe	G7320
Read	Translum ins stent graft for aneurysmal segment of aorta NOS	7A1Cz
Read	Ischaemic foot	G733.
Read	Peripheral vascular disease monitoring	662U.
Read	Angioplasty of femoral artery	XaIzW
Read	Ischaemia of feet	Xa7lT
Read	Diabetic peripheral angiopathy	G73y0
Read	Percut trans balloon angiopl major systemic pulmon coll art	7A0C3
Read	Percutaneous balloon angioplasty of coeliac artery	X013e
Read	Insertion of arterial stent	X016i
Read	Endovasc insert stent (AND/OR stenting [aneur aort]) bif NEC	XaLhJ
Read	Ischaemic toe	2G63.
Read	Percutaneous balloon angioplasty	X70Vw
Read	Other specified peripheral vascular disease NOS	G73yz
Read	Angioplasty of aorta	X013W
Read	Aortic aneurysm without mention of rupture NOS	G716.
Read	Percutaneous transluminal angioplasty suprarenal artery NEC	7A353
Read	Intracranial bypass to carotid artery	7A201
Read	Coronary artery bypass grafts greater than 5	X00tL
Read	Double anastomosis of mammary arteries to coronary arteries	XE0En
Read	Ligation of carotid artery	7A211
Read	Percutaneous balloon angioplasty of superior femoral artery	X013r
Read	Acroparaesthesia - Schultze's type	G73y4
Read	Insertion of stent into arteriovenous fistula	X018c
Read	Percut transluminal insertion of stent into pulmonary vein	7A6K4

Read	Percut transluminal balloon angioplasty stent pulmonary vein	XaLjH
Read	Other peripheral vascular disease	G73..
Read	Replace aneurysm bifurc aorta by anast aorta to iliac artery	7A113
Read	[X]Peripheral angiopathy in diseases classified elsewhere	Gyu7A
Read	Percutaneous balloon angioplasty of inferior vena cava	Xa0F8
Read	Angioplasty of axillary artery	Xa7y1
Read	Monckeberg's medial sclerosis	G7020
Read	Percutaneous transluminal angioplasty of brachial artery	7A281
Read	Percutaneous low speed rotational coronary atherectomy	X00tQ
Read	Percut transluminal balloon angioplasty pulmonary artery NEC	7A0A6
Read	Percutaneous transluminal angioplasty of subclavian artery	7A280
Read	Insertion of coronary artery stent	X00tU
Read	Percutaneous transluminal balloon angioplasty pulmonary vein	7A6K1
Read	Transluminal aortic branched stent graft NEC	7A1AB
Read	Aortic aneurysm screening abnormal	XaX4O
Read	Angioplasty of pulmonary venous pathway	X00yE
Read	Other peripheral vascular disease	XE0VP
Read	Angioplasty of intracranial artery	X00Hb
Read	Peroperative angioplasty	7A6G1
Read	Percutaneous transluminal insertion stent hepatic artery NEC	7.80E+03
Read	Endovascular ins stent for aortic dissection in any position	7A1BB
Read	Percutaneous transluminal insertion stent into renal artery	7A325
Read	Percutaneous transluminal insertion stent subclavian art	7A28G
Read	Endovascular insertion of stent for thoracic aortic aneurysm	XaLhH
Read	Endovas ins stent graft for infrarenal abdom aortic aneurysm	XaMmH
Read	Insertion of stent in inferior vena cava	X00yS
Read	Angioplasty of hepatic artery	X013b
Read	Percutaneous balloon angioplasty of hepatic artery	X013c
Read	Coronary artery bypass grafts x 5	X00tK
Read	Thromboangiitis obliterans NOS	G731z
Read	Buerger's disease	G7310
Read	Percutaneous balloon angioplasty of superior mesenteric arte	X013g
Read	Aneurysm of suprarenal aorta	XaX6B
Read	Endovascul insert stent infrarenal abdominal aortic aneurysm	7A1B8
Read	Percutaneous transluminal angioplasty of vein NEC	XaLit
Read	Percutaneous balloon angioplasty of internal iliac artery	X013l
Read	Peripheral vascular disease monitoring	XaIIV
Read	Angioplasty of arteriovenous fistula	X018T
Read	Occlusion of artery of lower limb	XaZT5
Read	[X]Aortic aneurysm of unspecified site, ruptured	Gyu71
Read	Angioplasty of superficial femoral artery	X013q
Read	Angioplasty of abdominal aorta	X013Z
Read	Percut transluminal insertion of stent into pulmonary artery	XaMyP
Read	Angioplasty of arterial graft	X013x
Read	Aortic aneurysm	G71..
Read	Carotid artery occlusion	G631.
Read	Angioplasty of systemic to pulmonary artery shunt	X90RO
Read	Percutaneous transluminal insertion of stent femoral artery	7A4B9
Read	Bronchoscopic placement of stent	X00r3
Read	Percutaneous transluminal insertion of stent portal vein	XaMlV
Read	Percutaneous balloon angioplasty of arterial graft	X013y
Read	Aortic atherosclerosis	G700.

	Read	HAVS - Hand-arm vibration syndrome	X205U
	Read	Percutaneous balloon angioplasty of superior vena cava	Xa0F7
	Read	Percutaneous transluminal angioplasty inf mesenteric art NEC	7A352
	Read	Juxtarenal aortic aneurysm	G7140
	Read	Coronary artery bypass graft x 1	X00tG
	Read	Insertion of carotid artery stent	X016j
	Read	Insertion of stent in pulmonary venous pathway	X00yC
	Read	Patch angioplasty of renal artery	7A305
	Read	Critical ischaemia of foot	XaE3G
	Read	Percutaneous transluminal insertion of stent cerebral artery	7A256
	Read	Percutaneous transluminal angioplasty sup mesenteric art NEC	7A351
	Read	Angioplasty of common femoral artery	X013o
	Read	Patch repair of inferior mesenteric artery	XaCDH
	Read	Gangrene of hand	G7324
	Read	Critical upper limb ischaemia	X203S
	Read	Carotid artery stenosis	G634.
Psychiatric disorders			
	SNOMED	Bipolar I disorder, most recent episode depressed, in partial remission	49512000
	SNOMED	[X]Bipolar affective disorder, current episode manic without psychotic symptoms	4.43561E+14
	SNOMED	Severe bipolar II disorder, most recent episode major depressive with psychotic features	30520009
	SNOMED	Bipolar I disorder, most recent episode depressed with atypical features (disorder)	29929003
	SNOMED	[X]Recurrent severe episodes of psychogenic depressive psychosis	192378005
	SNOMED	Recurrent manic episodes, unspecified (disorder)	6.45481E+14
	SNOMED	[X] (Bipolar affective disorders: [bipolar II] or [other]) or (recurrent manic episodes)	192364009
	SNOMED	Chronic paranoid schizophrenia (disorder)	31658008
	SNOMED	Unspecified bipolar affective disorder, moderate (disorder)	6.02531E+14
	SNOMED	Subchronic catatonic schizophrenia with acute exacerbations (disorder)	86817004
	SNOMED	[X]Other manic episodes (disorder)	192353006
	SNOMED	[X]Schizotypal disorder (disorder)	268692006
	SNOMED	Acute exacerbation of chronic hebephrenic schizophrenia (disorder)	191539009
	SNOMED	Femoral head bipolar component (physical object)	469999008
	SNOMED	Reactive psychoses	231437006
	SNOMED	Profile of mood states, bipolar	304757008
	SNOMED	[Chronic paranoid psychosis] or [Sanders disease]	191669007
	SNOMED	Latent schizophrenia NOS (disorder)	191566009
	SNOMED	Post-schizophrenic depression	231485007
	SNOMED	[X] (Other nonorganic psychotic disorders) or (chronic hallucinatory psychosis)	192346002
	SNOMED	Moderate depressed bipolar I disorder	66631006
	SNOMED	Mild manic bipolar I disorder (disorder)	71984005
	SNOMED	Hellers syndrome	71961003
	SNOMED	Bipolar I disorder, most recent episode depressed, in full remission	22121000
	SNOMED	Unspecified schizophrenia	5.89331E+14
	SNOMED	Catatonia (finding)	247917007
	SNOMED	Bipolar affective disorder, current episode depression (disorder)	191627008
	SNOMED	Residual schizophrenia (disorder)	26025008
	SNOMED	[X] (Unspecified organic or symptomatic mental disorder) or (organic psychosis NOS) or (symptomatic psychosis NOS)	192204008
	SNOMED	Unspecified manic-depressive psychoses	6.13631E+14
	SNOMED	Severe depressed bipolar I disorder with psychotic features, mood-incongruent	26203008
	SNOMED	Chronic paranoid schizophrenia	191553009
	SNOMED	[X]Bipolar affective disorder, current episode manic with psychotic symptoms	192357007
	SNOMED	Bipolar I disorder, most recent episode manic, in full remission	30935000
	SNOMED	Chronic schizophrenia with acute exacerbations	26847009

SNOMED	Simple schizophrenia	192325006
SNOMED	Schizophrenia clinic (environment)	702919008
SNOMED	[X]Persistent delusional disorder, unspecified (disorder)	192332002
SNOMED	Severe bipolar I disorder, single manic episode with psychotic features	41832009
SNOMED	Unspecified schizophrenia (disorder)	191528006
SNOMED	Bipolar II disorder, most recent episode major depressive with catatonic features	22407005
SNOMED	Non-organic psychosis NOS (disorder)	268625004
SNOMED	Bipolar affective disorder, currently depressed, NOS (disorder)	191635006
SNOMED	[X] Mania with mood-incongruent psychotic symptoms	192352001
SNOMED	Unspecified schizoaffective schizophrenia (disorder)	5.89381E+14
SNOMED	Hebephrenic schizophrenia in remission	191540006
SNOMED	Bipolar II disorder	8.08691E+14
SNOMED	Chronic catatonic schizophrenia	68995007
SNOMED	Schizoaffective disorder, bipolar type	38368003
SNOMED	[X]Schizoaffective disorder, unspecified (disorder)	4.39911E+14
SNOMED	Prepsychotic schizophrenia	247804008
SNOMED	Other and unspecified affective psychoses (finding)	191662003
SNOMED	Cotard's syndrome	357705009
SNOMED	Chronic catatonic schizophrenia (disorder)	191545001
SNOMED	[X]Other schizoaffective disorders	4.10341E+14
SNOMED	Schizoaffective disorder, mixed type	270901009
SNOMED	Severe mixed bipolar I disorder (disorder)	2.71E+11
SNOMED	Schizoaffective disorder, manic type	271428004
SNOMED	Severe bipolar I disorder, single manic episode with psychotic fearures, mood-congruent	13581000
SNOMED	Borderline schizophrenia	274952002
SNOMED	[X] Manic episode (& [bipolar disorder, single manic episode])	192349009
SNOMED	[X] (Schizophrenia: [cenesthopathic] or [other]) or (schizophreniform disorder [including psychosis] NOS) (disorder)	192326007
SNOMED	Severe bipolar I disorder, most recent episode manic, without psychotic features	162004
SNOMED	Acute exacerbation of chronic schizoaffective schizophrenia	191572009
SNOMED	[X] (Other acute predominantly delusional psychotic disorders) or (psychogenic paranoid psychosis) (disorder)	4.13991E+14
SNOMED	Mixed bipolar affective disorder, severe, without mention of psychosis	5.29851E+14
SNOMED	Simple schizophrenia (disorder)	154866008
SNOMED	Unspecified bipolar affective disorder, in full remission	191653000
SNOMED	[X]Acute polymorphic psychotic disorder without symptoms of schizophrenia (disorder)	4.52061E+14
SNOMED	History of - schizophrenia	161468000
SNOMED	[X]Undifferentiated schizophrenia	4.42251E+14
SNOMED	[X] Acute polymorphic psychotic disorder without symptoms of schizophrenia (& [bouffee delirante] or [cycloid psychosis])	192334001
SNOMED	Manic mood	1.90051E+14
SNOMED	Bipolar affective disorder, currently depressed, NOS	6.13511E+14
SNOMED	FH: Schizophrenia	137730004
SNOMED	Bipolar I disorder, single manic episode	9340000
SNOMED	[X] (Other nonorganic psychotic disorders) or (chronic hallucinatory psychosis)	4.80111E+14
SNOMED	Bipolar II disorder, most recent episode major depressive with melancholic features (disorder)	34315001
SNOMED	[X] (Unspecified organic or symptomatic mental disorder) or (organic psychosis NOS) or (symptomatic psychosis NOS) (disorder)	4.31811E+14
SNOMED	Senile and presenile organic psychotic conditions	268612007
SNOMED	Bipolar I disorder, single manic episode, in full remission	3530005
SNOMED	Bipolar affective disorder, currently manic, in partial or unspecified remission (disorder)	191624001
SNOMED	Recurrent manic episodes, severe, with psychosis (disorder)	191595000
SNOMED	[X]Bipolar affective disorder, currently in remission (disorder)	4.54161E+14
SNOMED	Bipolar I disorder, most recent episode manic (disorder)	767635003
SNOMED	Bipolar affective disorder, currently depressed, in partial or unspecified remission	191633004
SNOMED	Bipolar 1 disorder	371596008

SNOMED	[X]Other schizophrenia	4.26321E+14
SNOMED	Manic bipolar I disorder, NOS	68569003
SNOMED	Mixed bipolar affective disorder, mild (disorder)	191638008
SNOMED	Severe bipolar I disorder, most recent episode depressed with psychotic feature, mood-congruent	54761006
SNOMED	Catatonic schizophrenia	154868009
SNOMED	Bipolar affective disorder, currently depressed, in full remission (disorder)	191634005
SNOMED	Capgras' syndrome	44906001
SNOMED	Unspecified paranoid schizophrenia (disorder)	191551006
SNOMED	Unspecified bipolar affective disorder, unspecified (disorder)	191647000
SNOMED	Unspecified bipolar affective disorder, mild (disorder)	6.02521E+14
SNOMED	Schizoaffective schizophrenia NOS (disorder)	191575006
SNOMED	Paranoid schizophrenia, NOS	64905009
SNOMED	Insight present: [neurotic cond.] or [psychotic cond.] (finding)	269034001
SNOMED	Severe bipolar I disorder, most recent episode depressed with psychotic features	59617007
SNOMED	Recurrent major depressive episodes, severe, with psychosis (disorder)	191613003
SNOMED	Condition, insight present: [neurotic] or [psychotic]	163610003
SNOMED	Bipolar affective disorder, currently manic, moderate (disorder)	191621009
SNOMED	Paranoid schizophrenia in remission (disorder)	191556001
SNOMED	Acute exacerbation of chronic catatonic schizophrenia (disorder)	191548004
SNOMED	Unspecified bipolar affective disorder, in partial or unspecified remission	191652005
SNOMED	Recurrent manic episodes, unspecified (disorder)	191591009
SNOMED	Paranoid schizophrenia	268748000
SNOMED	[X] Acute polymorphic psychotic disorder without symptoms of schizophrenia (& [bouffee delirante] or [cycloid psychosis])	4.18931E+14
SNOMED	[X] (Bipolar affective disorders: [bipolar II] or [other]) or (recurrent manic episodes) (disorder)	4.19271E+14
SNOMED	Bipolar disorder	192355004
SNOMED	[X]Severe depressive episode with psychotic symptoms (disorder)	3.97711E+14
SNOMED	[X]Schizotypal disorder (disorder)	4.26331E+14
SNOMED	[X] Schizotypal disorder (& [latent] or [latent schizophrenic reaction] or [borderline] or [prepsychotic] or [prodromal] or [pseudoneurotic] or [pseudopsychopathic] (disorder)	192328008
SNOMED	Acute exacerbation of subchronic schizophrenia (disorder)	191530008
SNOMED	Paranoid disorder	191667009
SNOMED	[X]Involutional paranoid state	4.31361E+14
SNOMED	Unspecified affective psychoses NOS (finding)	191663008
SNOMED	Bipolar I disorder, most recent episode manic with postpartum onset	55516002
SNOMED	Other and unspecified affective psychoses	6.46051E+14
SNOMED	Chronic undifferentiated schizophrenia	29599000
SNOMED	Subchronic undifferentiated schizophrenia (disorder)	85861002
SNOMED	Bipolar affective disorder, currently manic, mild (disorder)	191620005
SNOMED	Schizoaffective schizophrenia NOS	6.45431E+14
SNOMED	Severe mixed bipolar I disorder with psychotic features	10981006
SNOMED	Severe bipolar II disorder, most recent episode major depressive without psychotic features	81319007
SNOMED	Subchronic schizophrenia with acute exacerbations, NOS	111482003
SNOMED	Acute exacerbation of subchronic latent schizophrenia	191563001
SNOMED	Unspecified bipolar affective disorder, moderate (disorder)	191649002
SNOMED	History of schizophrenia in child of subject (situation)	444180005
SNOMED	[X] (Mania with psychotic symptoms (& mood [congruent] or [incongruent])) or (manic stupor) (disorder)	4.67591E+14
SNOMED	Subchronic residual schizophrenia with acute exacerbations	70814008
SNOMED	Subchronic paranoid schizophrenia with acute exacerbations	41521002
SNOMED	Severe bipolar I disorder, most recent episode manic, with psychotic features	28663008
SNOMED	Bipolar affective disorder, currently manic, severe, without mention of psychosis (disorder)	5.30301E+14
SNOMED	Severe mixed bipolar disorder with psychotic features, mood-incongruent	10875004
SNOMED	Other senile and presenile organic psychoses (disorder)	268614008

SNOMED	[X]Bipolar affective disorder, current episode severe depression without psychotic symptoms	192359005
SNOMED	Mild mixed bipolar disorder	43769008
SNOMED	Recurrent manic episode NOS (disorder)	6.33671E+14
SNOMED	Bipolar partial shoulder prosthesis	467114009
SNOMED	Schizophrenic disorders (disorder)	191526005
SNOMED	[X] Manic-depressive psychosis, depressed type without psychotic symptoms	4.67361E+14
SNOMED	[X]Mania with psychotic symptoms	268699002
SNOMED	[X]Undifferentiated schizophrenia	192322009
SNOMED	Severe bipolar II disorder, most recent episode major depressive with psychotic features, mood-incongruent (disorder)	20960007
SNOMED	Late onset schizophrenia (disorder)	416340002
SNOMED	Bipolar 2 disorder, most recent episode rapid cycling	6.1771E+13
SNOMED	[X]Schizophrenia, unspecified (disorder)	4.70301E+14
SNOMED	Psychogenic paranoid psychosis (disorder)	191680007
SNOMED	[X]Bipolar affective disorder, current episode manic without psychotic symptoms	192356003
SNOMED	[X]Borderline schizophrenia	4.43791E+14
SNOMED	Endotherapy electrosurgical coagulator/cutter, bipolar, refurbished	468608004
SNOMED	Severe manic bipolar I disorder with psychotic features, mood-incongruent (disorder)	33380008
SNOMED	Unspecified manic-depressive psychoses (disorder)	191657004
SNOMED	Schizophrenia in remission (disorder)	4926007
SNOMED	[X]Mood affective disorder: [unspecified] or [psychosis NOS] (disorder)	4.13791E+14
SNOMED	[X]Bipolar affective disorder, currently in remission (disorder)	192363003
SNOMED	Severe major depression with psychotic features (disorder)	5.07861E+14
SNOMED	Mild bipolar I disorder, most recent episode depressed	74686005
SNOMED	Chronic bipolar I disorder, most recent episode depressed	51637008
SNOMED	Bipolar I disorder, most recent episode mixed with catatonic features	73471000
SNOMED	Mixed bipolar affective disorder, in full remission	191643001
SNOMED	Paranoid schizophrenia	268747005
SNOMED	Residual schizophrenia	192324005
SNOMED	Atypical manic disorder (disorder)	191658009
SNOMED	Other mixed manic-depressive psychoses (disorder)	191660006
SNOMED	Other mixed manic-depressive psychoses	6.13641E+14
SNOMED	Organic delusional disorder	5510009
SNOMED	Severe manic bipolar I disorder	2.3741E+13
SNOMED	Childhood schizophrenia NOS	231484006
SNOMED	Non-organic psychosis NOS	5.58821E+14
SNOMED	Psychotic depression, NOS	73867007
SNOMED	Severe bipolar I disorder, single manic episode without psychotic features	14495005
SNOMED	Bipolar cells of retina	72060002
SNOMED	Schizophrenia, NOS	58214004
SNOMED	Bipolar I disorder	8.08681E+14
SNOMED	Insight present: [neurotic cond.] or [psychotic cond.]	140817005
SNOMED	(Paranoid schizophrenia) or (paraphrenia)	154869001
SNOMED	Unspecified catatonic schizophrenia	5.89361E+14
SNOMED	Schizoaffective disorder, depressive type	84760002
SNOMED	Signposting to Bipolar UK	2.65225E+15
SNOMED	Affective psychoses (& [bipolar] or [depressive] or [manic])	191580002
SNOMED	Mixed bipolar affective disorder, in partial or unspecified remission (disorder)	6.02471E+14
SNOMED	Mild bipolar II disorder, most recent episode major depressive (disorder)	71294008
SNOMED	[X]Schizophrenia, schizotypal and delusional disorders (disorder)	192318004
SNOMED	Unspecified bipolar affective disorder, severe, without mention of psychosis	191650002
SNOMED	[X]Other schizophrenia	268691004
SNOMED	Single major depressive episode, severe, with psychosis	191604000

SNOMED	Bipolar affective disorder	154871001
SNOMED	Unspecified bipolar affective disorder, in partial or unspecified remission	6.02551E+14
SNOMED	Residual schizophrenia in remission (disorder)	51133006
SNOMED	Bipolar I disorder, most recent episode mixed with postpartum onset (disorder)	65042007
SNOMED	Moderate bipolar I disorder, single manic episode (disorder)	28884001
SNOMED	Unspecified bipolar affective disorder, severe, without mention of psychosis	6.13611E+14
SNOMED	Bipolar affective disorder, currently manic, in full remission (disorder)	191625000
SNOMED	Severe manic bipolar I disorder with psychotic features, mood-congruent (disorder)	78640000
SNOMED	Non-organic psychoses (disorder)	191525009
SNOMED	Undifferentiated schizophrenia, in remission	39610001
SNOMED	Chronic paranoid schizophrenia with acute exacerbations	17435002
SNOMED	Psychoses with origin in childhood (disorder)	191687005
SNOMED	Bipolar I disorder, most recent episode depression (disorder)	767636002
SNOMED	Organic bipolar disorder	231444002
SNOMED	Other and unspecified manic-depressive psychoses NOS (disorder)	191661005
SNOMED	Other specified non-organic psychoses	6.21261E+14
SNOMED	Bipolar II disorder (disorder)	231497008
SNOMED	Hysteroscopic bipolar electrosurgical excision of uterine fibroid	722022003
SNOMED	Coenesthopathic schizophrenia	191577003
SNOMED	[V]Personal history of schizophrenia (situation)	4.27491E+14
SNOMED	Manic mood	1.86611E+14
SNOMED	Unspecified hebephrenic schizophrenia	6.33351E+14
SNOMED	Severe bipolar II disorder, most recent episode major depressive with psychotic features, mood-congruent	19300006
SNOMED	Bipolar I disorder, most recent episode manic with catatonic features (disorder)	17782008
SNOMED	Mania (disorder)	231494001
SNOMED	(Other senile and presenile organic psychoses) or (presbyophrenic psychosis)	191468008
SNOMED	Hysteroscopic bipolar electrosurgical excision of uterine polyp (procedure)	8.69241E+14
SNOMED	Chronic residual schizophrenia with acute exacerbations	30336007
SNOMED	Hebephrenic schizophrenia (disorder)	192320001
SNOMED	Subchronic catatonic schizophrenia	191544002
SNOMED	Unspecified paranoid schizophrenia	6.33401E+14
SNOMED	Unspecified affective psychoses NOS (finding)	6.46061E+14
SNOMED	[X]Bipolar affective disorder, current episode severe depression without psychotic symptoms	4.65911E+14
SNOMED	Hebephrenic schizophrenia NOS (disorder)	191541005
SNOMED	Unspecified hebephrenic schizophrenia (disorder)	191535003
SNOMED	Schizophrenia NOS (disorder)	191579000
SNOMED	[X]Other manic episodes (disorder)	4.64001E+14
SNOMED	Severe bipolar II disorder, most recent episode major depressive, in partial remission	67002003
SNOMED	Childhood schizophrenia NOS	6.71541E+14
SNOMED	Catatonic schizophrenia (disorder)	191542003
SNOMED	Schizophrenia NOS	6.45451E+14
SNOMED	Robotic single-use bipolar electrosurgical instrument	763489009
SNOMED	Planar-resection electrosurgical diathermy bipolar conducting unit	700739009
SNOMED	Universal electrosurgical diathermy system bipolar electrode, reprocessed (physical object)	701439000
SNOMED	Schizoaffective disorder	68890003
SNOMED	Chronic undifferentiated schizophrenia with acute exacerbations	79204003
SNOMED	Bipolar clinic	702826007
SNOMED	Other schizophrenia (disorder)	5.58801E+14
SNOMED	Bipolar I disorder, most recent episode mixed, in remission	35481005
SNOMED	Other specified non-organic psychoses	191700002
SNOMED	Chronic schizophrenic	83746006
SNOMED	[X]Mania without psychotic symptoms (disorder)	4.28091E+14

SNOMED	Disorganised schizophrenia in remission	31373002
SNOMED	Bipolar I disorder, single manic episode with catatonic features	87950005
SNOMED	Other affective psychosis NOS	6.46071E+14
SNOMED	Subchronic schizophrenia	191529003
SNOMED	Bipolar disorder caused by drug	1.62387E+16
SNOMED	Cataleptic schizophrenia	1.08948E+15
SNOMED	Percutaneous electrosurgical probe, spinal-denervation, bipolar	717140007
SNOMED	Laparoscopic electrosurgical forceps, bipolar, reprocessed	718121003
SNOMED	[X]Other schizoaffective disorders (disorder)	192344004
SNOMED	Child psychosis NOS (& [childhood schizophrenia NOS]) (disorder)	191699003
SNOMED	Unspecified bipolar affective disorder, in full remission	6.02561E+14
SNOMED	Chronic disorganised schizophrenia with acute exacerbations	35218008
SNOMED	[X]Bipolar affective disorder, current episode manic with psychotic symptoms	4.31661E+14
SNOMED	Unspecified bipolar affective disorder, severe, with psychosis (disorder)	191651003
SNOMED	Subchronic disorganized schizophrenia with acute exacerbations	14291003
SNOMED	Moderate bipolar disorder	79584002
SNOMED	Bipolar (qualifier value)	260994008
SNOMED	Paranoid psychosis NOS (disorder)	6.20131E+14
SNOMED	Bipolar II disorder, most recent episode major depressive with atypical features	43568002
SNOMED	[X]Other persistent delusional disorders (disorder)	268693001
SNOMED	Bipolar I disorder, most recent episode depressed with catatonic features	21900002
SNOMED	Schizophrenia in remission	191533005
SNOMED	Hysteroscopic bipolar electrosurgical excision of uterine fibroid	9.55951E+14
SNOMED	Severe bipolar II disorder	371604007
SNOMED	Hypomania	231496004
SNOMED	Other and unspecified manic-depressive psychoses (disorder)	191656008
SNOMED	Subchronic residual schizophrenia	76566000
SNOMED	Endoscopic electrosurgical electrode, bipolar, single-use (physical object)	717133006
SNOMED	Bipolar disorder, most recent episode manic (disorder)	767632000
SNOMED	Bipolar disorder, full remission	41836007
SNOMED	Partial hip replacement with bipolar prosthesis (procedure)	713686009
SNOMED	Mixed bipolar affective disorder, severe, without mention of psychosis	191640003
SNOMED	Mixed bipolar affective disorder, severe, with psychosis (disorder)	191641004
SNOMED	Bipolar 1 disorder, single manic episode, in partial remission	78269000
SNOMED	Mild bipolar I disorder, single manic episode (disorder)	41552001
SNOMED	Bipolar affective disorder, currently depressed, severe, without mention of psychosis (disorder)	191631002
SNOMED	Severe bipolar disorder with psychotic features, mood-incongruent	26530004
SNOMED	Destruction of lesion or structure by biterminal bipolar fulguration	73223003
SNOMED	Manic disorder, single episode	268619003
SNOMED	Subchronic paranoid schizophrenia	79866005
SNOMED	Bipolar affective disorder, currently depressed, severe, with psychosis (disorder)	191632009
SNOMED	Bipolar humeral head prosthesis (physical object)	469222000
SNOMED	[X]Severe depressive episode with psychotic symptoms (disorder)	268704005
SNOMED	Moderate bipolar I disorder, most recent episode manic	82998009
SNOMED	Acute exacerbation of chronic latent schizophrenia (disorder)	191564007
SNOMED	[X]Other bipolar affective disorders	268701002
SNOMED	Latent schizophrenia (disorder)	191559008
SNOMED	Catatonic schizophrenia, NOS	76839006
SNOMED	Schizoaffective schizophrenia (disorder)	191567000
SNOMED	Unspecified bipolar affective disorder (disorder)	191646009
SNOMED	[X] (Other acute predominantly delusional psychotic disorders) or (psychogenic paranoid psychosis)	192338003
SNOMED	Bipolar II disorder, most recent episode major depressive with postpartum onset (disorder)	30687003

SNOMED	Bipolar I disorder, most recent episode depressed with postpartum onset	87203005
SNOMED	Bipolar affective disorder, current episode mixed	192362008
SNOMED	[X]Bipolar affective disorder, unspecified	192365005
SNOMED	Unspecified schizoaffective schizophrenia (disorder)	191568005
SNOMED	Rapid cycling bipolar I disorder (disorder)	1.33091E+14
SNOMED	Undifferentiated schizophrenia (disorder)	111484002
SNOMED	Robotic reusable bipolar electrosurgical instrument	763488001
SNOMED	(Manic disorder, single episode) or (hypomanic psychoses)	191581003
SNOMED	Sensitiver Beziehungswahn	192330005
SNOMED	Subchronic disorganised schizophrenia	27387000
SNOMED	[X]Other persistent delusional disorders (disorder)	4.40261E+14
SNOMED	Simple schizophrenia NOS	191534004
SNOMED	Bipolar I disorder	7.90711E+14
SNOMED	Recurrent manic episodes, mild (disorder)	191592002
SNOMED	Acute schizophrenic episode (disorder)	268617001
SNOMED	Paraphrenia, NOS	26472000
SNOMED	Manic	286571004
SNOMED	[X]Delusional dysmorphophobia	192331009
SNOMED	Schizophrenia: [other] or [cenesthopathic]	191576007
SNOMED	Disorganized schizophrenia (disorder)	35252006
SNOMED	Other schizophrenia	268618006
SNOMED	Subchronic paranoid schizophrenia (disorder)	191552004
SNOMED	[X]Mania with psychotic symptoms	4.01681E+14
SNOMED	[X] Psychosis: [unspecified nonorganic] or [NOS] (disorder)	4.71051E+14
SNOMED	Severe bipolar disorder (disorder)	371600003
SNOMED	Paranoid schizophrenia (disorder)	192319007
SNOMED	[X]Schizophrenia, unspecified	192327003
SNOMED	Manic mood (finding)	405273008
SNOMED	Subchronic latent schizophrenia	191561004
SNOMED	Schizophrenia NOS	154870000
SNOMED	Other senile and presenile organic psychoses (disorder)	5.44861E+14
SNOMED	[X](Schizoaffective disorder, mixed type) or (cyclic schizophrenia) or (mixed schizophrenic and affective psychosis) (disorder)	192343005
SNOMED	Endoscopic electrosurgical electrode, bipolar, reusable	717132001
SNOMED	Acute exacerbation of subchronic hebephrenic schizophrenia (disorder)	191538001
SNOMED	Bipolar II disorder, most recent episode major depressive	16295005
SNOMED	Moderate mixed bipolar disorder	40926005
SNOMED	Persistent delusional disorder	231487004
SNOMED	Unspecified bipolar affective disorder, mild (disorder)	191648005
SNOMED	Residual disintegrative psychoses (disorder)	191693002
SNOMED	Bipolar 1 disorder, single manic episode, in remission	75360000
SNOMED	Mixed bipolar affective disorder, NOS (disorder)	6.02481E+14
SNOMED	Non-organic psychosis NOS	191701003
SNOMED	Bipolar affective disorder, currently manic, severe, without mention of psychosis (disorder)	191622002
SNOMED	Chronic catatonic schizophrenia with acute exacerbations	21894002
SNOMED	Acute polymorphic psychotic disorder with symptoms of schizophrenia	712850003
SNOMED	Paranoid psychosis NOS (disorder)	191674004
SNOMED	Psychoses: [other nonorganic] or [reactive]	191675003
SNOMED	Moderate bipolar II disorder, most recent episode major depressive	35846004
SNOMED	Schizotypal personality disorder	31027006
SNOMED	Acute exacerbation of subchronic catatonic schizophrenia (disorder)	191547009
SNOMED	H/O: schizophrenia (situation)	138739000
SNOMED	Bipolar affective disorder, currently depressed, severe, with psychosis (disorder)	192361001

SNOMED	Catatonic schizophrenia NOS (disorder)	6.33391E+14
SNOMED	Acute exacerbation of chronic paranoid schizophrenia (disorder)	191555002
SNOMED	Other schizophrenia NOS	191578008
SNOMED	[X] Acute schizophrenia-like psychotic disorder (& [brief schizophreniform disorder] or [brief schizophrenifrm psych] or [oneirophrenia] or [schizophrenic reaction]) (disorder)	192336004
SNOMED	Unspecified bipolar affective disorder, NOS (disorder)	191654006
SNOMED	[X] Dementia: [unspecif] or [presenile NOS (including presenile psychosis NOS)] or [primary degenerative NOS] or [senile NOS (including senile psychosis NOS)] or [senile depressed or paranoid type] (disorder)	4.13681E+14
SNOMED	[X]Bipolar affective disorder, unspecified (disorder)	4.17731E+14
SNOMED	Bipolar type II disorder currently in full remission	723905008
SNOMED	[X]Other bipolar affective disorders (disorder)	4.26091E+14
SNOMED	Bipolar affective disorder, currently manic, in partial or unspecified remission (disorder)	6.15921E+14
SNOMED	Bipolar disorder in partial remission (disorder)	5703000
SNOMED	Bipolar II disorder, NOS	83225003
SNOMED	Chronic hebephrenic schizophrenia	12939007
SNOMED	Mixed bipolar affective disorder, NOS (disorder)	191644007
SNOMED	Acute exacerbation of chronic schizophrenia (disorder)	191531007
SNOMED	Mixed bipolar affective disorder, in partial or unspecified remission (disorder)	191642006
SNOMED	Endoscopic electrosurgical electrode, bipolar, reprocessed (physical object)	717116004
SNOMED	Simple schizophrenia NOS	5.89341E+14
SNOMED	Severe bipolar II disorder, most recent episode major depressive, in full remission (disorder)	12969000
SNOMED	Severe bipolar I disorder (disorder)	371599001
SNOMED	Severe mixed bipolar disorder without psychotic features	46229002
SNOMED	Bipolar affective disorder, currently depressed, unspecified (disorder)	6.15941E+14
SNOMED	Bipolar I disorder, single manic episode with postpartum onset (disorder)	1499003
SNOMED	Bipolar affective disorder, currently manic, unspecified	6.33731E+14
SNOMED	Bipolar affective disorder, currently depressed, severe, without mention of psychosis (disorder)	5.30311E+14
SNOMED	[X]Acute polymorphic psychotic disorder with symptoms of schizophrenia (disorder)	192335000
SNOMED	[X] Dementia: [unspecif] or [presenile NOS (including presenile psychosis NOS)] or [primary degenerative NOS] or [senile NOS (including senile psychosis NOS)] or [senile depressed or paranoid type]	192180006
SNOMED	Bipolar affective disorder, currently depressed, moderate (disorder)	191630001
SNOMED	[X]Manic episode, unspecified	192354000
SNOMED	Rapid cycling bipolar II disorder (disorder)	789061003
SNOMED	[X] Childhood disintegrative disorder: [other] or [dementia infantalis (including disintegrative psychosis and Hellers syndrome and symbiotic psychosis)] (disorder)	4.70961E+14
SNOMED	[X]Schizophrenia, schizotypal and delusional disorders	4.17601E+14
SNOMED	[X]Persistent delusional disorder, unspecified (disorder)	4.54771E+14
SNOMED	Unspecified bipolar affective disorder	6.02491E+14
SNOMED	Severe manic bipolar affective disorder, with psychosis	191623007
SNOMED	Chronic hebephrenic schizophrenia (disorder)	191537006
SNOMED	Acute polymorphic psychotic disorder without symptoms of schizophrenia (disorder)	712824002
SNOMED	Acute schizophrenic episode (& [oneirophrenia]) (disorder)	191558000
SNOMED	Laparoscopic electrosurgical forceps, bipolar	470560000
SNOMED	Bipolar affective disorder, current episode manic (disorder)	191618007
SNOMED	Unspecified bipolar affective disorder, severe, with psychosis (disorder)	6.02541E+14
SNOMED	[X] (Catatonic schizophrenia) (disorder)	192321002
SNOMED	Bipolar affective disorder, currently depressed, in partial or unspecified remission	6.15951E+14
SNOMED	Unspecified latent schizophrenia (disorder)	5.79821E+14
SNOMED	Bipolar affective disorder, currently manic, NOS	6.15931E+14
SNOMED	[X]Mania without psychotic symptoms (disorder)	192351008
SNOMED	Psychotic condition, insight present	268957000
SNOMED	Other affective psychosis NOS (finding)	191666000

SNOMED	Bipolar affective disorder, currently manic, unspecified (disorder)	191619004
SNOMED	Paranoid schizophrenia NOS (disorder)	5.79811E+14
SNOMED	[X]Manic episode, unspecified (disorder)	268700001
SNOMED	[X]Manic episode, unspecified (disorder)	4.41461E+14
SNOMED	Subchronic undifferentiated schizophrenia with acute exacerbations	7025000
SNOMED	Chronic paranoid psychosis	268622001
SNOMED	[X]Mood affective disorder: [unspecified] or [psychosis NOS] (disorder)	192391006
SNOMED	Subchronic hebephrenic schizophrenia (disorder)	191536002
SNOMED	Severe depressed bipolar I disorder (disorder)	2.61E+11
SNOMED	Bipolar I disorder, most recent episode depressed with melancholic features	75752004
SNOMED	Bipolar I disorder, most recent episode hypomanic (disorder)	31446002
SNOMED	Severe bipolar disorder with psychotic features (disorder)	4441000
SNOMED	Unspecified bipolar affective disorder, NOS (disorder)	6.23971E+14
SNOMED	Unspecified latent schizophrenia	191560003
SNOMED	Hebephrenic schizophrenia NOS	5.89351E+14
SNOMED	Bipolar affective disorder	13746004
SNOMED	Severe bipolar I disorder, most recent episode mixed, with psychotic features, mood-congruent	64731001
SNOMED	Manic bipolar I disorder in remission (disorder)	45479006
SNOMED	Chronic latent schizophrenia (disorder)	191562006
SNOMED	Mixed bipolar affective disorder, severe	7.64591E+14
SNOMED	Mixed bipolar affective disorder, unspecified (disorder)	191637003
SNOMED	Depressed bipolar I disorder, NOS	49468007
SNOMED	Endotherapy electrosurgical coagulator/cutter, bipolar, reusable	468330009
SNOMED	Paranoid schizophrenia NOS	191557005
SNOMED	Bipolar disorder, most recent episode depression	767631007
SNOMED	Schizophrenia, schizoaffective, in remission	191574005
SNOMED	[X]Acute polymorphic psychotic disorder without symptoms of schizophrenia (disorder)	268694007
SNOMED	Universal electrosurgical diathermy system bipolar electrode, single-use	701442006
SNOMED	Signposting to Bipolar UK (procedure)	1.05378E+15
SNOMED	Acute schizophrenia-like psychotic disorder	278853003
SNOMED	Bipolar type I disorder currently in full remission (disorder)	723903001
SNOMED	Schizophrenic psychoses (& [paranoid schizophrenia])	154865007
SNOMED	Bipolar affective disorder, most recent episode mixed (disorder)	767633005
SNOMED	Senile or presenile psychoses NOS	6.23941E+14
SNOMED	Catatonic schizophrenia NOS (disorder)	191550007
SNOMED	Mixed bipolar disorder in full remission	111485001
SNOMED	Affective psychosis	441704009
SNOMED	Recurrent manic episodes, moderate (disorder)	191593007
SNOMED	Subchronic catatonic schizophrenia	42868002
SNOMED	Other schizophrenia NOS (disorder)	6.45441E+14
SNOMED	Recurrent manic episode NOS (disorder)	191598003
SNOMED	Referral for bipolar disorder	416422002
SNOMED	Mixed bipolar affective disorder, unspecified	6.13581E+14
SNOMED	Subchronic schizoaffective schizophrenia	191569002
SNOMED	Acute exacerbation of subchronic paranoid schizophrenia (disorder)	191554003
SNOMED	[X] Childhood disintegrative disorder: [other] or [dementia infantalis (including disintegrative psychosis and Hellers syndrome and symbiotic psychosis)]	192584009
SNOMED	Other and unspecified manic-depressive psychoses NOS (disorder)	6.24011E+14
SNOMED	Schizophrenia assoc'n member	161103001
SNOMED	Severe bipolar disorder without psychotic features (disorder)	53049002
SNOMED	Bipolar affective disorder, currently manic, NOS (disorder)	191626004
SNOMED	Unspecified catatonic schizophrenia	191543008
SNOMED	Mixed bipolar affective disorder, in partial remission (disorder)	1.68363E+15

SNOMED	Severe bipolar II disorder, most recent episode major depressive, in remission (disorder)	35722002
SNOMED	[X] Psychosis: [unspecified nonorganic] or [NOS]	192347006
SNOMED	Senile or presenile psychoses NOS	191469000
SNOMED	Subchronic schizophrenia (disorder)	16990005
SNOMED	Mixed bipolar disorder in partial remission	36583000
SNOMED	Bipolar I disorder, most recent episode manic, in partial remission	63249007
SNOMED	Bipolar affective disorder, currently depressed, unspecified (disorder)	191628003
SNOMED	Mixed bipolar affective disorder, moderate (disorder)	191639000
SNOMED	[X]Bipolar affective disorder, current episode mild or moderate depression (disorder)	192358002
SNOMED	Mixed bipolar affective disorder, severe (disorder)	1.69618E+15
SNOMED	Psychosis and severe depression co-occurrent and due to bipolar affective disorder	765176007
SNOMED	Bipolar affective disorder, currently depressed, mild (disorder)	191629006
SNOMED	Poor insight into psychotic condition	163611004
SNOMED	Unspecified bipolar affective disorder, unspecified	6.02511E+14
SNOMED	Chronic schizoaffective schizophrenia (disorder)	191570001
SNOMED	[X]Acute polymorphic psychotic disorder with symptoms of schizophrenia	4.42891E+14
SNOMED	Catatonic schizophrenia in remission	191549007
SNOMED	Latent schizophrenia in remission	191565008
SNOMED	Catatonic schizophrenia in remission (disorder)	111483008
SNOMED	FH: Schizophrenia	160328009
SNOMED	Bipolar affective disorder	268749008
SNOMED	[X] Single episode of psychogenic depressive psychosis	4.01761E+14
SNOMED	Mixed bipolar I disorder	16506000
SNOMED	Bipolar diathermy	257797008
SNOMED	Hebephrenic schizophrenia	154867004
SNOMED	[X]Organic delusional [schizophrenia-like] disorder	192190003
SNOMED	Mixed bipolar affective disorder, in partial remission	7.60721E+14
SNOMED	Non-organic psychoses	154864006
SNOMED	Bipolar disorder, in remission	85248005
SNOMED	Recurrent manic episodes, severe	7.64621E+14
SNOMED	Acute exacerbation of subchronic schizoaffective schizophrenia	191571002
SNOMED	Latent schizophrenia NOS (disorder)	5.89371E+14
SNOMED	Bipolar II disorder, most recent episode hypomanic	48937005
SNOMED	Presbyophrenic psychosis (disorder)	231438001
SNOMED	Recurrent manic episodes (disorder)	191590005
SNOMED	Schizophrenia association member (finding)	138410004
SNOMED	Mixed bipolar affective disorder (disorder)	191636007
SNOMED	[X] Severe depressive episode with psychotic symptoms: (& single episode of [major depression] or [psychogenic depressive psychosis] or [psychotic depression] or [reactive depressive psychosis]) (disorder)	192370003
SNOMED	Severe bipolar disorder with psychotic features, mood-congruent (disorder)	70546001
SNOMED	Other and unspecified manic-depressive psychoses (disorder)	1.35636E+15
SNOMED	Mild bipolar disorder (disorder)	13313007
SNOMED	Chronic residual schizophrenia	71103003
SNOMED	Selective destruction of fetus by percutaneous intrafetal bipolar coagulation of umbilical cord (procedure)	1.21901E+15
SNOMED	[X]Bipolar affective disorder, current episode mild or moderate depression (disorder)	4.67121E+14
SNOMED	Severe depressed bipolar disorder without psychotic features	61403008
SNOMED	[X] (Schizophrenia: [cenesthopathic] or [other]) or (schizophreniform disorder [including psychosis] NOS)	4.70741E+14
SNOMED	Chronic bipolar II disorder, most recent episode major depressive	1196001
SNOMED	[X]Schizoaffective disorder, unspecified (disorder)	192345003
SNOMED	[X] Mania: [episode, unspecified] or [NOS]	4.55841E+14
SNOMED	Depressed bipolar I disorder in remission (disorder)	53607008
SNOMED	Schizophrenia simplex	191527001

SNOMED	Severe bipolar I disorder, single manic episode with psychotic features, mood-incongruent	86058007
SNOMED	Paranoid schizophrenia in remission (disorder)	63181006
Read	[X]Residual schizophrenia	Eu205
Read	[X]Bipolar affect disorder cur epi mild or moderate depressn	Eu313
Read	[V]Personal history of schizophrenia	ZV110
Read	[X]Mania with psychotic symptoms	XE1ZV
Read	Paranoid psychosis NOS	E12z.
Read	Other senile and presenile organic psychoses	E00y.
Read	Non-organic psychosis NOS	XE1Y5
Read	[X]Bipolar affective disorder	Eu31.
Read	Other senile and presenile organic psychoses	XE1Xt
Read	[X]Bipolar affective disorder, unspecified	Eu31z
Read	Paranoid schizophrenia NOS	E103z
Read	Bipolar diathermy	X79ul
Read	Catatonia	X763T
Read	H/O: schizophrenia	1464
Read	[X]Severe depressive episode with psychotic symptoms	XE1ZZ
Read	Schizophrenic psychoses (& [paranoid schizophrenia])	XE1aM
Read	Bipolar affect disord,currently manic, part/unspec remission	E1145
Read	[X]Bipolar affective disorder, currently in remission	Eu317
Read	[X]Bipolar affect dis cur epi severe depres with psyc symp	Eu315
Read	Bipolar affect disord, currently manic,severe with psychosis	E1144
Read	[X]Manic-depress psychosis,depressd,no psychotic symptoms	XaCHo
Read	Recurrent manic episodes, severe without mention psychosis	E1113
Read	[X]Other manic episodes	Eu30y
Read	Bipolar affective disorder, currently manic, unspecified	E1140
Read	Schizo-affective schizophrenia	E107.
Read	[X]Schizoaffective disorder, mixed type	Eu252
Read	Unspecified bipolar affective disorder, in full remission	E1176
Read	[X]Ac schizophrenia-like psychot disord (& [named variants])	Eu232
Read	[X]Bipolar affective disorder, current episode mixed	Eu316
Read	Hebephrenic schizophrenia NOS	E101z
Read	(Manic disorder, single episode) or (hypomanic psychoses)	E110.
Read	Non-organic psychoses	E1...
Read	[X]Undifferentiated schizophrenia	Eu203
Read	[X]Other persistent delusional disorders	Eu22y
Read	Schizophrenia NOS	E10z.
Read	Acute exacerbation of chronic schizo-affective schizophrenia	E1074
Read	[X]Organic delusional [schizophrenia-like] disorder	Eu052
Read	[X]Schizoaffective disorder, depressive type	Eu251
Read	Psychogenic paranoid psychosis	E134.
Read	[X]Simple schizophrenia	Eu206
Read	[X]Schizotypal disorder	Eu21.
Read	Subchronic catatonic schizophrenia	E1021
Read	Unspecified hebephrenic schizophrenia	E1010
Read	Paranoid schizophrenia in remission	E1035
Read	[X]Severe depressive episode with psychotic symptoms	Eu323
Read	Recurrent manic episodes	E111.
Read	Unspecified bipolar affective disorder, severe, no psychosis	E1173
Read	Non-organic psychosis NOS	E1z..
Read	Unspecified schizo-affective schizophrenia	E1070
Read	Mixed bipolar affective disorder, severe, without psychosis	E1163

Read	Acute exacerbation of subchronic schizophrenia	E1003
Read	Other schizophrenia NOS	E10yz
Read	Bipolar affect disord, now depressed, severe, no psychosis	E1153
Read	Paranoid schizophrenia	E103.
Read	Acute exacerbation of subchronic catatonic schizophrenia	E1023
Read	Bipolar affect disord, currently manic, severe, no psychosis	E1143
Read	Recurrent major depressive episodes, severe, with psychosis	E1134
Read	[X]Schizophrenia	Eu20.
Read	Subchronic hebephrenic schizophrenia	E1011
Read	Profile of mood states, bipolar	XaA6j
Read	Chronic schizo-affective schizophrenia	E1072
Read	Mixed bipolar affective disorder, in full remission	E1166
Read	Mixed bipolar affective disorder	E116.
Read	[X]Hebephrenic schizophrenia	Eu201
Read	Other schizophrenia	E10y.
Read	Coenesthopathic schizophrenia	E10y1
Read	Schizoaffective schizophrenia in remission	E1075
Read	Presbyophrenic psychosis	X00R0
Read	Subchronic latent schizophrenia	E1051
Read	Unspecified latent schizophrenia	E1050
Read	Acute schizophrenic episode	XE1Xw
Read	[X]Other bipolar affective disorders	Eu31y
Read	[X]Schizoaffective disorders	Eu25.
Read	Atypical manic disorder	E11y1
Read	Schizophrenia in remission	E1005
Read	Unspecified paranoid schizophrenia	E1030
Read	Bipolar affective disorder, currently manic, NOS	E114z
Read	Chronic paranoid psychosis	E121.
Read	Paraphrenia	E122.
Read	Unspecified catatonic schizophrenia	E1020
Read	Residual schizophrenia	E106.
Read	Other and unspecified manic-depressive psychoses NOS	E11yz
Read	[X]Cotard syndrome	Eu222
Read	Chronic hebephrenic schizophrenia	E1012
Read	Simple schizophrenia	E100.
Read	Unspecified bipolar affective disorder, unspecified	E1170
Read	Other and unspecified manic-depressive psychoses	E11y.
Read	Subchronic schizophrenia	E1001
Read	[X]Other schizophrenia	Eu20y
Read	Bipolar affective disorder, currently depressed, moderate	E1152
Read	Mixed bipolar affective disorder, moderate	E1162
Read	Senile or presenile psychoses NOS	E00z.
Read	FH: Schizophrenia	1284
Read	Catatonic schizophrenia	E102.
Read	Latent schizophrenia in remission	E1055
Read	Schizophrenic disorders	E10..
Read	[X] Psychosis: [unspecified nonorganic] or [NOS]	Eu2z.
Read	[X]Mania without psychotic symptoms	Eu301
Read	[X]Catatonic schizophrenia	Eu202
Read	[X]Paranoid schizophrenia	Eu200
Read	[X]Hypomania	Eu300
Read	Manic mood	Xa3WO

Read	Other schizophrenia	XE1Xx
Read	[X]Delusional disorder	Eu220
Read	[X]Schizoaffective disorder, unspecified	Eu25z
Read	Bipolar	X9078
Read	Mixed bipolar affective disorder, partial/unspec remission	E1165
Read	Disintegrative psychosis	E141.
Read	Catatonic schizophrenia in remission	E1025
Read	Recurrent manic episodes, mild	E1111
Read	Acute exacerbation of chronic schizophrenia	E1004
Read	Acute schizophrenic episode	E104.
Read	Organic bipolar disorder	X00RO
Read	Unspecified manic-depressive psychoses	E11y0
Read	Unspecified affective psychoses NOS	E11z0
Read	[X]Bipolar affective disorder type I	Eu318
Read	Acute exacerbation of subchronic paranoid schizophrenia	E1033
Read	Childhood schizophrenia NOS	X00S7
Read	[X]Manic episode, unspecified	XE1ZW
Read	[X]Delusional misidentification syndrome	Eu221
Read	[X]Schizoaffective disorder, manic type	Eu250
Read	Recurrent manic episodes, severe, with psychosis	E1114
Read	[X]Other schizophrenia	XE1ZM
Read	Bipolar disorder	X00SM
Read	Unspecified bipolar affect disord, partial/unspec remission	E1175
Read	Mixed bipolar affective disorder, unspecified	E1160
Read	Bipolar affective disorder, currently depressed	E115.
Read	Hebephrenic schizophrenia in remission	E1015
Read	[X]Persistent delusional disorders	Eu22.
Read	[X]Manic episode, unspecified	Eu30z
Read	[X]Schizophrenia, schizotypal and delusional disorders	Eu2..
Read	Bipolar affective disorder, currently manic, mild	E1141
Read	Recurrent manic episodes, unspecified	E1110
Read	Bipolar affective disorder, currently manic, full remission	E1146
Read	[X]Post-schizophrenic depression	Eu204
Read	Other nonorganic psychoses	E13..
Read	Bipolar II disorder	X00SN
Read	Acute exacerbation of chronic latent schizophrenia	E1054
Read	Latent schizophrenia NOS	E105z
Read	Mixed bipolar affective disorder, mild	E1161
Read	[X]Other nonorganic psychotic disorders	Eu2y.
Read	Other affective psychosis NOS	E11zz
Read	Acute schizophrenia-like psychotic disorder	Xa0s9
Read	Borderline schizophrenia	XM1GG
Read	Bipolar I disorder	XaY1Y
Read	[X]Bipolar affective disorder, current episode hypomanic	Eu310
Read	Hebephrenic schizophrenia	E101.
Read	Schizo-affective schizophrenia NOS	E107z
Read	Affective psychoses	E11..
Read	[X]Other persistent delusional disorders	XE1ZP
Read	Bipolar affective disorder, currently manic	E114.
Read	Other specified non-organic psychoses	E1y..
Read	Subchronic schizo-affective schizophrenia	E1071
Read	[X]Major depression, severe with psychotic symptoms	Eu328

Read	Simple schizophrenia NOS	E100z
Read	Acute exacerbation of chronic catatonic schizophrenia	E1024
Read	[X]Bipolar affect disorder cur epi manic wout psychotic symp	Eu311
Read	Acute exacerbation of chronic paranoid schizophrenia	E1034
Read	Chronic schizophrenic	E1002
Read	Chronic paranoid schizophrenia	E1032
Read	[X]Mania with psychotic symptoms	Eu302
Read	[X]Other bipolar affective disorders	XE1ZX
Read	Condition, insight present: [neurotic] or [psychotic]	285..
Read	Other mixed manic-depressive psychoses	E11y3
Read	[X]Schizotypal disorder	XE1ZN
Read	[X]Bipolar affect disorder cur epi manic with psychotic symp	Eu312
Read	Latent schizophrenia	E105.
Read	Acute exacerbation subchronic schizo-affective schizophrenia	E1073
Read	Bipolar affective disorder, now depressed, in full remission	E1156
Read	Unspecified bipolar affective disorder, mild	E1171
Read	Recurrent manic episodes, moderate	E1112
Read	Mixed bipolar affective disorder, NOS	E116z
Read	Acute exacerbation of chronic hebephrenic schizophrenia	E1014
Read	Atypical schizophrenia	E10y0
Read	Manic mood	1S42.
Read	Bipolar affective disorder, currently depressed, unspecified	E1150
Read	[X]Persistent delusional disorder, unspecified	Eu22z
Read	Chronic latent schizophrenia	E1052
Read	Unspecified bipolar affective disorder, NOS	E117z
Read	Bipolar affective disorder, currently depressed, mild	E1151
Read	Recurrent manic episode NOS	E111z
Read	Other and unspecified affective psychoses	E11z.
Read	(Paranoid schizophrenia) or (paraphrenia)	XE1aO
Read	Bipolar affect disord, now depressed, severe with psychosis	E1154
Read	Acute exacerbation of subchronic hebephrenic schizophrenia	E1013
Read	Unspecified bipolar affective disorder, moderate	E1172
Read	Chronic catatonic schizophrenia	E1022
Read	Unspecified schizophrenia	E1000
Read	Bipolar affect disord, now depressed, part/unspec remission	E1155
Read	[X]Schizophrenia, unspecified	Eu20z
Read	[X]Bipolar affective disorder type II	Eu319
Read	Acute exacerbation of subchronic latent schizophrenia	E1053
Read	Child psychosis NOS (& [childhood schizophrenia NOS])	E14z.
Read	[X]Manic episode	Eu30.
Read	Bipolar affective disorder, currently manic, moderate	E1142
Read	Catatonic schizophrenia NOS	E102z
Read	Unspecified bipolar affective disorder	E117.
Read	Bipolar affective disorder, currently depressed, NOS	E115z
Read	[X]Other schizoaffective disorders	Eu25y
Read	Subchronic paranoid schizophrenia	E1031
Read	Schizophrenia association member	13Y2.
Read	Mixed bipolar affective disorder, severe, with psychosis	E1164
Read	Unspecified bipolar affective disorder,severe with psychosis	E1174
Read	Residual disintegrative psychoses	E1411
Read	Chronic paranoid psychosis	XE1Y2
Read	Psychoses with origin in childhood	E14..
